# Molecular identification of hyaluronate lyase, not hyaluronidase, as an intrinsic hyaluronan-degrading enzyme in *Clostridium perfringens* strain ATCC 13124

**DOI:** 10.1038/s41598-024-73955-y

**Published:** 2024-10-22

**Authors:** Tomoya Kumon, Sayoko Oiki, Wataru Hashimoto

**Affiliations:** Laboratory of Basic and Applied Molecular Biotechnology, Division of Food Science and Biotechnology, Graduate School of Agriculture, Kyoto University, Uji, Kyoto 611-0011 Japan

**Keywords:** Bacteriology, Pathogens, Enzymes, Glycobiology

## Abstract

*Clostridium perfringens*, an opportunistic pathogen, produces mu-toxin hyaluronidases including endo-β-*N*-acetylglucosaminidases (Nags) as a virulence invasion factor. To clarify an intrinsic factor for degradation of host extracellular hyaluronan, we focused on hyaluronate lyase (HysA), distinct from endo-β-*N*-acetylglucosaminidases. *C. perfringens* strain ATCC 13124 was found to assimilate host-derived extracellular mucosubstances, hyaluronan and mucin, which induced expression of the hyaluronan-related genetic cluster, including hyaluronate lyase gene (*hysA*), but repressed endo-β-*N*-acetylglucosaminidase genes. This genetic cluster is conserved in some strains of *C. perfringens*. The recombinant strain ATCC 13124 hyaluronate lyase HysA showed an hyaluronan-degrading activity through β-elimination reaction. The hyaluronan-degrading enzyme in the culture supernatant of strain ATCC 13124 exhibited the lyase activity and was identical to the recombinant hyaluronate lyase on the native-PAGE gel, followed by activity straining. These results demonstrated that the intrinsic hyaluronan-degrading enzyme of *C. perfringens* strain ATCC 13124 is hyaluronate lyase HysA, but not hyaluronidases NagH, NagJ, and NagK.

**Supplementary Information:**

The online version contains supplementary material available at 10.1038/s41598-024-73955-y.

## Introduction

Approximately 38 trillion bacteria, more than all human cells, are estimated to be indigenous to the human gut^1^. Intestinal bacteria establish competitive and symbiotic relationships through interactions with the host and other bacteria in the gut, where various nutrients are intermittently supplied by the host’s diet^2–4^. The gut microbiota is suggested to be correlated with host health, such as regulation of the immune system, allergy, inflammatory bowel disease, obesity, diabetes, and cancer^5–11^. While some beneficial bacteria, e.g., genera of *Bifidobacterium* and *Lactobacillus*, are indigenous to the gut, opportunistic pathogens, including genera of *Clostridium* and *Streptococcus*, can also exist. However, the mechanism of bacterial colonization in the gut remains to be clarified.

All animal cells, including the gut, are covered with a noncellular component called extracellular matrices. Extracellular matrices, composed of water, proteins, and polysaccharides, provide a physical scaffold for cells and promote cell differentiation and homeostasis^12^. Proteoglycans, a part of extracellular matrices, comprise glycosaminoglycans (GAGs) linked to core proteins. GAGs are heteropolysaccharides with a disaccharide-repeating unit of uronic acid (or galactose) and amino sugar^13^. Chondroitin sulfate C (CSC) and heparin (HP) are representative sulfated GAGs that constitute proteoglycans, while hyaluronan (HA), a representative nonsulfated GAG, exists independently of proteoglycans. HA is the most abundant GAG in the gut epithelium^14^.

In *Streptococcus* species, polysaccharide lyase and phosphotransferase system (PTS) are involved in the degradation and import of HA, respectively^15,16^. Our previous studies have demonstrated the streptococcal action on HA as follows^17–21^: Hyaluronate lyase is inducibly expressed in the presence of HA and essential for the assimilation of nutrient HA, followed by pathogenic damage to host tissues. Unsaturated HA disaccharide generated from HA through the lyase reaction is imported into the cytoplasm in the specific PTS, followed by degradation by cytoplasmic unsaturated glucuronyl hydrolase (UGL). The resultant unsaturated uronic acid is metabolized into glycolysis by isomerase, dehydrogenase, and other enzymes (Supplementary Fig. 1)^18–21^. These enzymes and transporter are encoded as a GAG genetic cluster in the streptococcal genome^19^. Some pathogenic bacteria, such as *Streptococcus pneumoniae* and *Staphylococcus aureus*, degrade GAGs and infect host cells^16,22^.

Intestinal epithelial cells are covered with a mucin layer outside the extracellular matrices. Mucin is a macromolecule composed of core proteins linked to sugar chains, including sialic acid, fucose, galactose, and amino sugar. The mucin layer, formed of mucins secreted from goblet cells within the intestinal epithelium, contains a low-density outer mucus layer and a high-density inner mucus layer. Intestinal bacteria are present in the outer mucus layer and utilize the mucin^3,23^. In contrast, the inner mucus layer functions as a barrier to block interactions between bacteria and host, thus leaving the bacteria undetectable^23,24^. However, it remains unclear whether pathogens that infect and invade host epithelial cells degrade the mucin layer.

*Clostridium perfringens* is a Gram-positive, anaerobic, and spore-forming bacterium that belongs to the Bacillota phylum, one of the most abundant phyla among intestinal bacteria. *C. perfringens* is a major component of the adult human gut microbiota, increasing in abundance from the ileum, the cecum, and the rectum^25^. However, this bacterium can cause life-threatening gas gangrene and food poisoning in humans and animals^26^. The clostridial virulence is largely attributed to the production of approximately twenty different toxins. For example, alpha-toxins, such as phospholipase, degrade phosphatidylcholine and sphingomyelin in the plasma membrane and cause endocytosis and cell death through the formation of diacylglycerol and ceramide^27^. *C. perfringens* also produce degrading enzymes such as hyaluronidase (mu-toxin), collagenase (kappa-toxin), proteases, and sialidase, to decompose connective tissue, leading to colonization and infection of the surrounding tissues^28^. Furthermore, mu-toxin, while itself is non-lethal, facilitates the spread of the major alpha-toxin. An endo-β-*N*-acetylglucosaminidase, NagH, has been identified as the hyaluronidase by screening recombinant lambda phages for glucuronidase activity^28^. Although NagH has long been considered the HA-degrading enzyme and a virulence factor, the intrinsic enzyme crucial for HA degradation has not yet been elucidated. Two complete genome sequences of *C. perfringens* strains 13 and ATCC 13124 were described by Shimizu et al. and Garry et al., respectively^29,30^. Based on these studies, five candidate genes, *nagH* (CPE0191), *nagI* (CPE0881), *nagJ* (CPE1234), *nagK* (CPE1279), and *nagL* (CPE1523), coding for hyaluronidases and *nanJ* (CPE0553) coding for sialidase, have been thought to be virulence factors in *C. perfringens* strain 13^29^. The *nagH* gene (CPF_0184) coding for hyaluronoglucosaminidase, *nagJ* (CPF_0875) coding for putative *O*-linked *N*-acetylglucosaminidase annotated as *nagI* in *C. perfringens* strain 13, *nagJ* (CPF_1442) and *nagK* (CPF_1487) were conserved in the *C. perfringens* strain ATCC 13124 genome. However, the *nagL* gene was not conserved.

VirS and VirR, a two-component system involved in regulating the expression of virulence factors, have also been identified^31^. VirS, a membrane sensor protein, receives environmental and intracellular signals via Agr, an accessory gene regulator, causing autophosphorylation. VirR is activated in the cytoplasm by receiving a phosphate group from VirS, resulting in control of transcriptional expression. The VirS/VirR regulon positively regulates major toxins, such as phospholipase C and collagenase, suggesting an important role during host infection. At the same time, the system mainly acts as a global repressor that negatively regulates general genes^32^. In contrast, RevR, a virulence regulator, is a global activator^33^. Transcriptome analysis by RNA sequence (RNA-seq) and DNA microarray of these three regulators of virulence factors indicated that the hyaluronidase candidate genes (*nagH*, *nagI*, and *nagJ*) and sporulation protein (*sigF*, *ftsN*, *spoIIIAG*, *sigG*, and *spoIVA*) as virulence factors are positively regulated by RevR, contrary to the negative regulation by VirR^28,33–35^. Based on this observation, NagH also potentially degrades HA in *C. perfringens*. This study identifies the intrinsic HA-degrading enzyme in *C. perfringens* by transcriptome analysis and enzyme characterization.

## Results and discussion

### Degradation and assimilation of hyaluronan by *C. perfringens* strain ATCC 13124

**Fig. 1:**
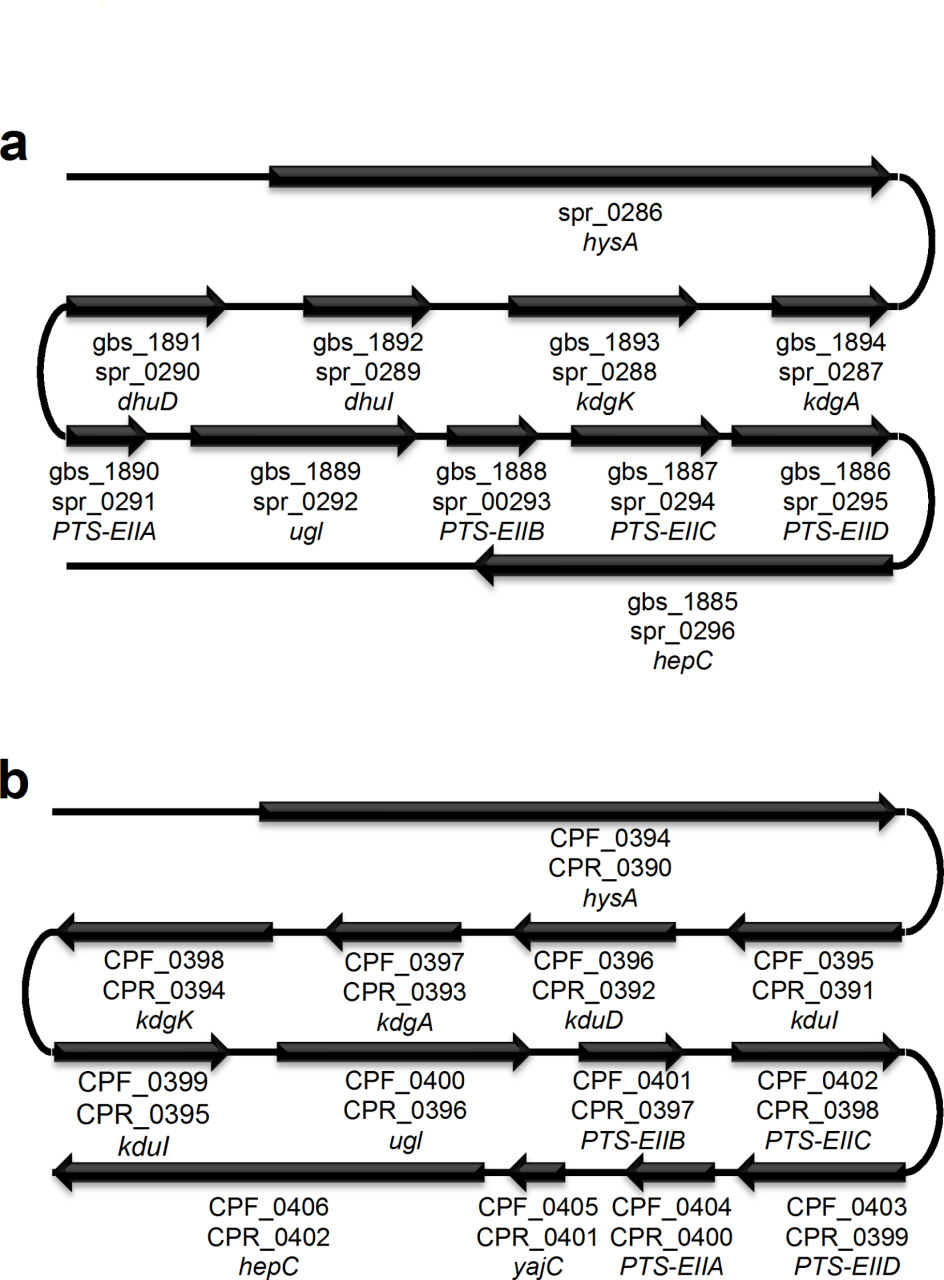
Glycosaminoglycan (GAG) genetic clusters. (**a**) *Streptococcus agalactiae* strain NEM316, gbs_XXXX; *Streptococcus pneumoniae* strain R6, spr_XXXX. (**b**) *Clostridium perfringens* strain ATCC 13124, CPF_XXXX; *C. perfringens* strain SM101, CPR_XXXX. Genes coding for proteins and enzymes for import, degradation, and metabolism of GAGs. XXXX, ID no. of each gene.

We previously found the GAG genetic cluster, in the *C. perfringens* strain ATCC 13124 genome as well as in the genomes of *Streptococcus agalactiae* and *S. pneumoniae* (Fig. 1)^21^. Complete genomes of *C. perfringens* strains 13, ATCC 13124, and SM101 are available on the database of KEGG Organisms: Complete Genomes (https://www.genome.jp/kegg/tables/br08606.html). The GAG genetic cluster is also completely conserved in the genome of *C. perfringens* strain SM101 (Fig. 1b), while *C. perfringens* strain 13 has no GAG genetic cluster. We further analyzed using the genome database of National Center for Biotechnology Information (NCBI) (https://www.ncbi.nlm.nih.gov/datasets/genome/). The GAG cluster was also detected in 28 out of 121 complete genomes of *C. perfringens* registered on the NCBI genome dataset as of August 23, 2024, indicating that the GAG cluster was conserved in *C. perfringens* strains to some extent. While *hysA* (CPF_0394), coding for hyaluronate lyase, and *hepC* (CPF_0406), coding for heparin lyase II/III-like protein, were found in the cluster, the HA-degrading enzyme in *C. perfringens* remained to be identified. *nagHIJK*, coding for hyaluronidases, which have been considered as candidate genes for HA degradation, were not found in the GAG genetic cluster. On the other hand, the cluster contains homologous genes coding for transporter and enzymes involved in the degradation and metabolism of HA. Therefore, *hysA* possibly plays a role in HA degradation as well as in virulence factors, because HA-degrading mu-toxin such as NagH has been considered a virulence factor^28^. In addition, it has been suggested that *C. perfringens* degrades and assimilates mucin by a sialidase, NanI, indicating that degradation of host mucosubstances such as HA and mucin is important for *C. perfringens* to colonize the human intestine^36,37^.

**Fig. 2:**
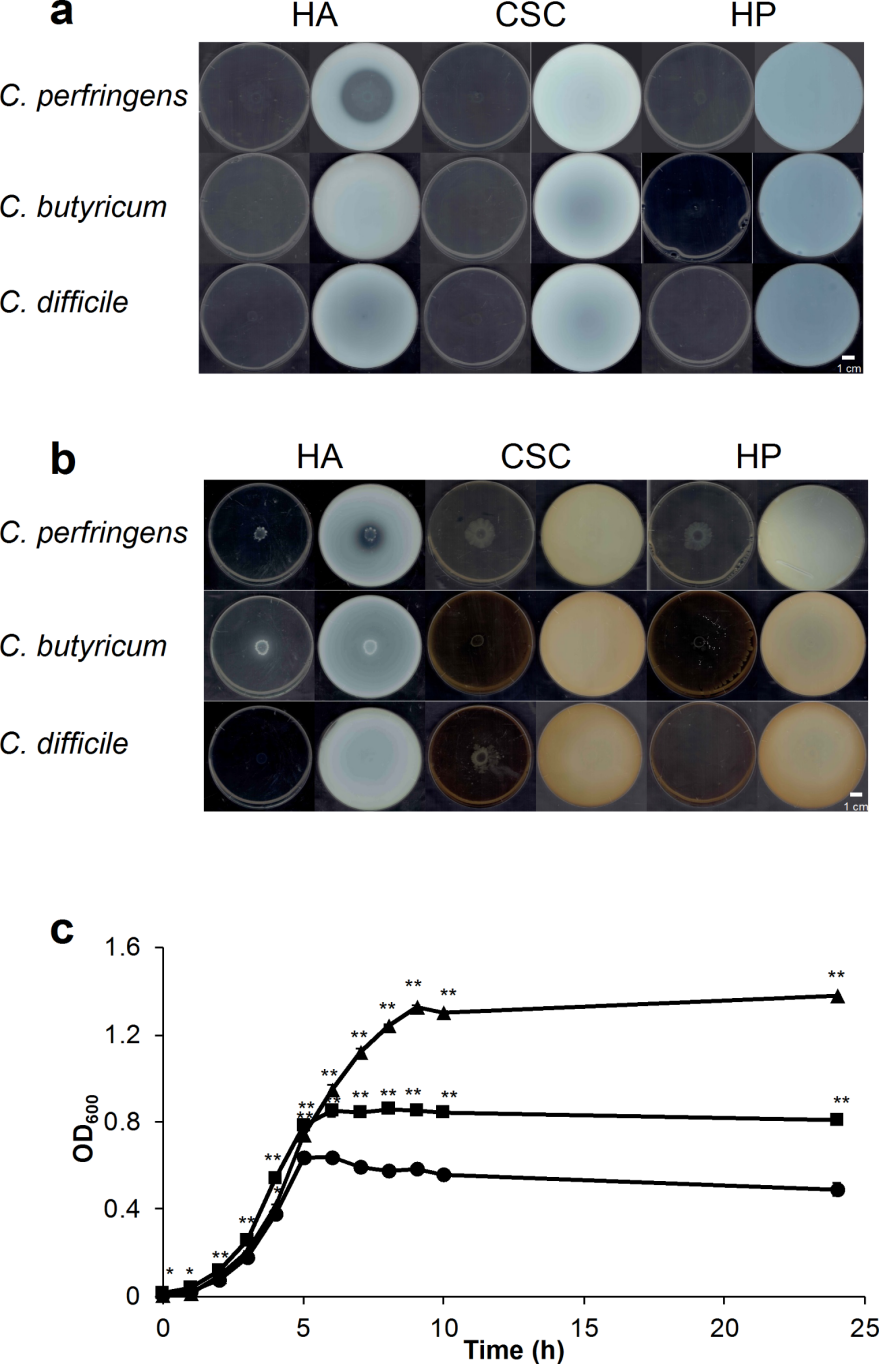
Degradation and assimilation of glycosaminoglycans (GAGs) and mucin by *C. perfringens* strain ATCC 13124. (**a**,**b**) Halo assay for the degradation of GAGs (HA, CSC, and HP). Plates before (left) and after (right) addition of acetic acid in nutrient-poor (**a**) and nutrient-rich medium (HA, glucose-rich medium; CSC and HP, GAM medium) (**b**). **c** growth of *C. perfringens* cells in the presence of HA or mucin. Circle, nutrient-poor medium; triangle, nutrient-poor medium containing HA; and square, nutrient-poor medium containing mucin. Each measurement represents the mean of three individual experiments. Significant differences from the control (nutrient-poor medium) were determined using Student’s *t*-test (**p < 0.01, *p < 0.05).

To clarify the clostridial molecular mechanism of HA degradation, we first examined the GAG degradation ability of *C. perfringens* strain ATCC 13124 and other related species, such as *Clostridium butyricum* and *Clostridioides difficile*, through the halo plate assay method (Fig. 2a,b). After clostridial cells were grown on halo plates containing GAGs and bovine serum albumin (BSA) in a nutrient-poor medium (Fig. 2a) or a nutrient-rich medium (Fig. 2b), acetic acid was added to confirm the presence or absence of a halo in a white precipitate. *C. perfringens* formed a halo in the presence of HA, while no halo was detected in the presence of CSC or HP, indicating HA degradation by *C. perfringens*. However, other species tested showed no halo, suggesting no GAG degradation. HA assimilation by *C. perfringens* was also examined by culturing in a nutrient-poor medium, excluding BSA and agar from the halo plate (Fig. 2c and Supplementary Fig. 2). *C. perfringens* cells in the presence of HA reached the stationary phase later than in the absence of HA and cell number at the stationary phase in the presence of HA was greater than that in the absence of HA. Moreover, *C. perfringens* also grew in the nutrient-poor medium containing mucin, indicating that the bacterial cells assimilate mucin and HA. Therefore, *C. perfringens* grown on the nutrient-poor medium in the presence of HA was expected to express genes involved in the degradation, import, and assimilation of GAGs in response to HA.

### Transcriptome analysis of *C. perfringens* strain ATCC 13124 in the presence of hyaluronan or mucin

**Fig. 3:**
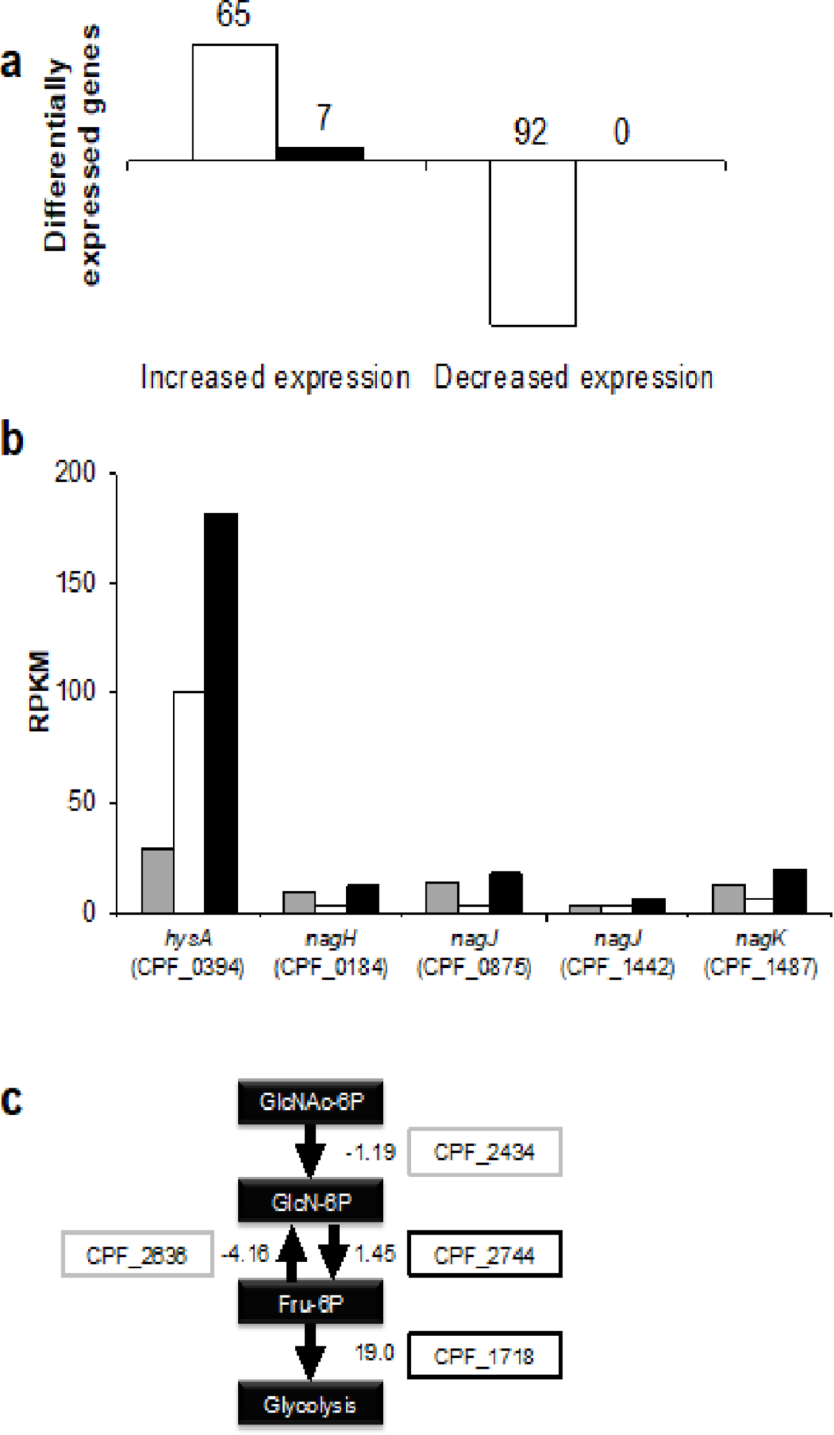
Transcriptome analysis of *C. perfringens* strain ATCC 13124 in the presence of hyaluronan or mucin. **a** the number of differentially expressed genes in nutrient-poor medium containing HA (white) or mucin (black). **b** RPKM of candidate genes for HA degradation. Nutrient-poor medium (gray) containing HA (white) or mucin (black). **c** the expression of genes involved in the utilization of amino sugar.

To investigate the gene expression in the presence of HA or mucin, we performed an RNA-seq analysis of *C. perfringens* strain ATCC 13124 cells grown in the nutrient-poor medium in the presence and absence of HA or mucin. The data are shown in Supplementary Fig. 3 and Supplementary Table 1. In the case of *C. perfringens* cells grown in the nutrient-poor medium, the dataset contained 49,915,882 reads with a length of 101 nucleotides. The reads obtained from the cells grown in the nutrient-poor medium containing HA and mucin were 49,913,574 and 68,868,196, respectively. The trimming data by sliding window method results were as follows: the nutrient-poor medium, 44,776,526; the nutrient-poor medium containing HA, 44,736,304; and the nutrient-poor medium containing mucin, 60,852,264. The mapping data occupied 90.70%, 90.53%, and 89.33%, respectively. Normalization was performed using RPKM (Reads Per Kilobase per Million mapped reads). Of the 2,921 genes, 2,735 genes for which RPKM could be calculated were analyzed. Figure 3a shows the number of differentially expressed genes by 10-fold or more (fold change) in the nutrient-poor medium containing HA compared with the nutrient-poor medium. In the nutrient-poor medium in the presence of HA, 65 genes were highly expressed, while the expression level of 92 genes was significantly decreased. No genes decreased in the nutrient-poor medium containing mucin, while seven genes showed a high expression level.

In response to both HA and mucin, the expression of *bioB* (CPF_1795), coding for biotin synthase, the product of which is an essential vitamin for animals, was elevated 49.3-fold and 12.5-fold in the nutrient-poor medium containing HA and mucin, respectively, compared with the nutrient-poor medium. This suggests the possibility that the gut-predominant *C. perfringens* provides the host with essential biotin through the assimilation of HA and mucin.

In the nutrient-poor medium containing HA, the following genes involved in cell proliferation were highly expressed: synthesis and transport of amino acid [CPF_0170 coding for cysteine synthase, CPF_0756 for the proton/sodium glutamate symporter, and *minC* (CPF_2394) for the probable septum site determining protein], purine-pyrimidine synthase [CPF_1001 for hypothetical protein and *xpt1* (CPF_0319) for xanthine phosphoribosyltransferase], and choline expression enzyme (CPF_0608 for MarR family/choline/ethanolamine kinase). In contrast, genes involved in the degradation of amino acids (CPF_1245 for l-serine dehydratase), transport of sugar (CPF_2652 for maltose/maltodextrin-binding protein and CPF_1113 for sugar-binding protein), and utilization of mucin [*gngC* (CPF_1119) for endo-β-galactosidase, *nanA* (CPF_0178) for *N*-acetylneuraminate lyase, and *fucP* (CPF_1052) for l-fucose: H^+^ symporter permease] had low expression levels. The expression of operon genes (CPF_0890–CPF_0903) responsible for the utilization of ethanolamine abundantly in the intestinal tract and available for various pathogenic bacteria were also decreased: *eutA* (CPF_0890) for ethanolamine utilization protein, CPF_0891 for ethanolamine ammonia-lyase large subunit, CPF_0892 for ethanolamine ammonia-lyase small subunit, *eutL* (CPF_0893) for ethanolamine utilization, CPF_0894 for ethanolamine utilization, CPF_0895 for ethanolamine utilization, *eutM* (CPF_0896) for ethanolamine utilization, CPF_0897 for ethanolamine utilization cobalamin adenosyltransferase, CPF_0898 for ethanolamine utilization, CPF_0899 for ethanolamine utilization protein, *eutN* (CPF_0900) for ethanolamine utilization, CPF_0901 for ethanolamine utilization, *eutH* (CPF_0902) for ethanolamine utilization, and *eutQ* (CPF_0903) for ethanolamine utilization^38^. These downregulated genes did not seem essential to assimilate HA as a carbon source.

In the nutrient-poor medium containing mucin, genes coding for endo-β-galactosidase (CPF_1119) involved in the utilization of mucin and constituents of GAG genetic cluster (CPF_0397 for 2-dehydro-3-deoxyphosphogluconate aldolase, CPF_0398 for 2-dehydro-3-deoxygluconokinase, and CPF_0406 for heparin lyase II/III-like protein) were up-regulated. Furthermore, changes in the expression of genes involved in *C. perfringens* strain 13 virulence are shown in Table 1. As a result, expression of *nagH* (CPF_0184), two *nagJ* (CPF_0875 and CPF_1442), and *nagK* (CPF_1487), which have been thought to encode major toxins as hyaluronidases, were unexpectedly lowly expressed in the nutrient-poor medium containing HA. On the other hand, *virS* (CPF_1751), a regulator of virulence factors, was highly expressed, indicating the high expression of *virR* (CPF_1752) due to the cotranscription of *virS* and *virR*^32^. A gene cluster homologous to the GAG genetic cluster of *S. pneumoniae* was found to be up-regulated in both nutrient-poor media containing each of HA and mucin. Table 2 shows changes in the expression level of each gene constituting the GAG genetic cluster in the nutrient-poor medium containing HA or mucin compared with the nutrient-poor medium.

**Table 1:** Expression change of virulence genes of *C. perfringens*.

*C. perfringens* strain ATCC 13124	Name	*C. perfringens* strain 13	Name	Nutrient-poor + HA/nutrient-poor	Nutrient-poor + mucin/nutrient-poor
CPF_0184	*nagH*	CPE_0191	*nagH*	− 4.45	1.08
CPF_1442	*nagJ*	CPE_1234	*nagJ*	− 1.92	1.29
CPF_0875	*nagJ*	CPE_0881	*nagI*	− 5.67	1.09
CPF_1487	*nagK*	CPE_1279	*nagK*	− 3.34	1.34
CPF_1751	*virS*	CPE_1500	*virS*	2.24	1.26

**Table 2:** Expression change of constituent genes of GAG genetic cluster.

Name	ATCC 13124	Nutrient-poor + HA/nutrient-poor	Nutrient-poor + mucin/nutrient-poor
*hysA*	CPF_0394	1.82	4.80
*kduI*	CPF_0395	9.62	10.99
*kduD*	CPF_0396	7.83	9.41
*kdgA*	CPF_0397	9.63	11.97
*kdgK*	CPF_0398	9.57	11.88
*kduI*	CPF_0399	2.28	6.87
*ugl*	CPF_0400	2.16	8.36
*PTS-EIIB*	CPF_0401	2.76	8.67
*PTS-EIIC*	CPF_0402	2.43	7.96
*PTS-EIID*	CPF_0403	2.41	8.03
*PTS-EIIA*	CPF_0404	6.59	9.27
*yajC*	CPF_0405	8.51	9.89
*hepC*	CPF_0406	10.02	13.64

HysA is the primary enzyme that degrades HA in *S. pneumoniae*. In *C. perfringens*, *nagH*, two *nagJ*, and *nagK* genes, which have been thought to encode degraders of HA, had repressed expression levels in the presence of HA, while expression of the *hysA* homologous gene (CPF_0394) increased. A comparison of RPKM obtained by RNA-seq suggested that the HA-degrading enzyme in *C. perfringens* may be a product of *hysA* but not the previous candidates (Fig. 3b). The GAG genetic cluster, including *hysA*, enables unsaturated uronic acid generated from GAG degradation to join glycolysis^21^. The expression of genes involved in the utilization of amino sugars was also investigated (Fig. 3c). The expression level (fold change) of *nagB* (CPF_2744) for glucosamine-6-phosphate deaminase and CPF_1718 for phosphofructokinase family protein responsible for metabolism of amino sugar into glycolysis, were up-regulated in the presence of hyaluronan, whereas *glmS* (CPF_2636) for fructose-6-phosphate aminotransferase for reverse pathway had decreased expression. Because *hysA* was more expressed in the presence of mucin rather than hyaluronan, further analysis of expression mechanism of the GAG genetic cluster in response to HA and mucin is required. Based on the above results, HysA, encoded in the GAG genetic cluster, likely is the enzyme in *C. perfringens* responsible for the degradation of HA. Moreover, unsaturated uronic acid and amino sugar, the resultant monosaccharides derived through HA degradation, were metabolized by glycolysis, unsaturated uronic acid were metabolized by GAG genetic cluster, and amino sugar by the Nag pathway.

### Characterization of recombinant CPF_0394, hyaluronate lyase CpeHysA

**Fig. 4:**
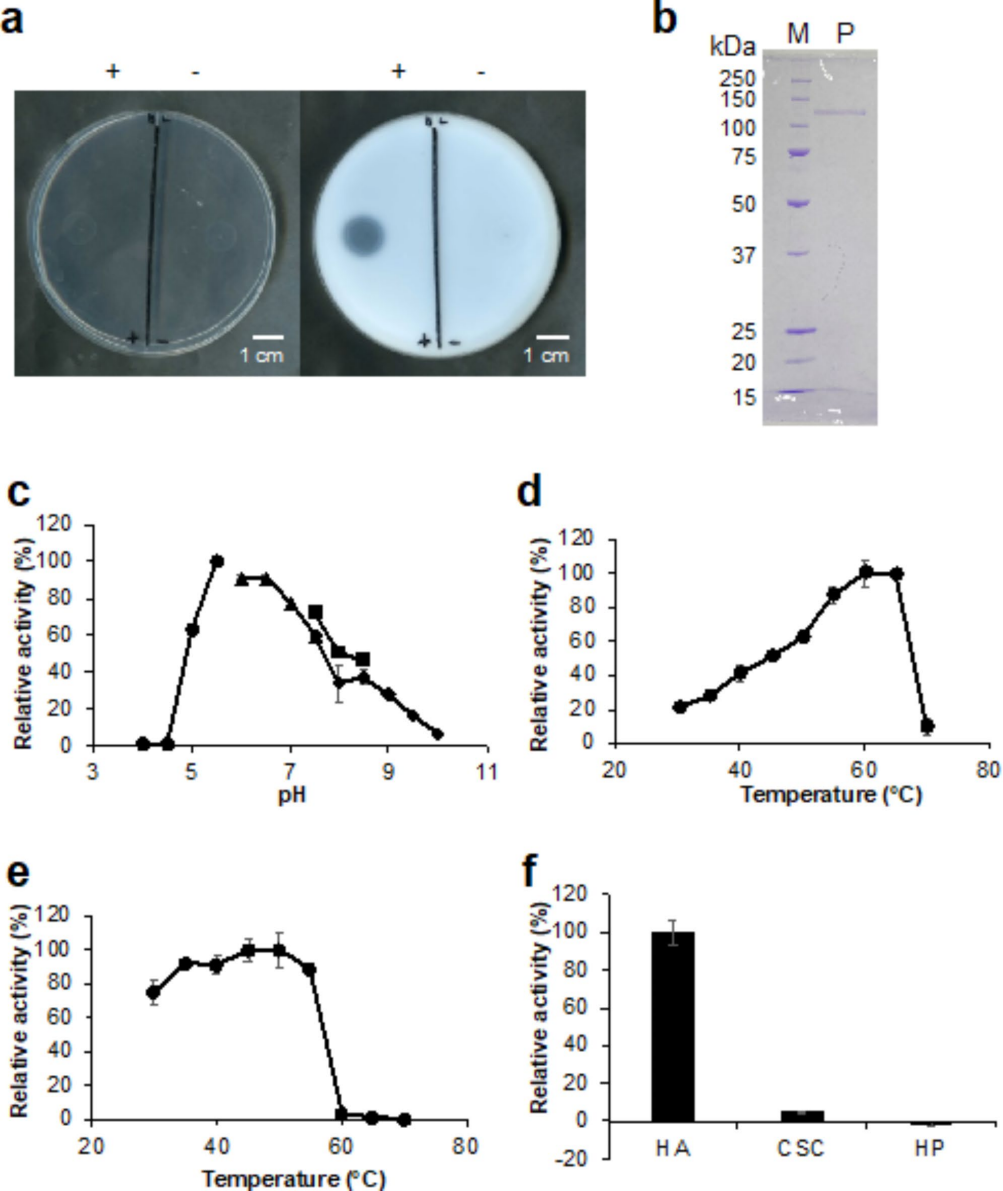
Expression, purification, and characterization of CPF_0394 (hyaluronate lyase CpeHysA). **a** halo assay for HA degradation using the cell extract obtained from the CpeHysA-expressing recombinant *E. coli*. Plates before (left) and after (right) addition of acetic acid. +, BL21-Gold(DE3)pLysS harboring pET21b-CPF_0394; and —, BL21-Gold(DE3)pLysS. **b** SDS-PAGE followed by CBB staining of the purified CpeHysA. Lane M, unstained marker of protein standard; lane P, purified CpeHysA. **c** Optimal pH. Buffers were sodium acetate (circle), potassium phosphate (triangle), Tris-HCl (square), and glycine-NaOH (rhombus). The activity measured in sodium acetate (pH 5.5) at 30 °C was taken as 100%. **d** Optimal temperature. The activity measured in Tris-HCl (pH 7.5) at 60 °C was taken as 100%. **e** Thermostability. The activity measured in Tris-HCl (pH 7.5) at 30 °C using CpeHysA preincubated at 45 °C was taken as 100%. **f** Substrate specificity. The activity measured in Tris-HCl (pH 7.5) at 30 °C using HA was taken as 100%. Each measurement represents the mean of three individual experiments.

To characterize HysA, the gene for CPF_0394 was cloned into the pET21b vector and expressed in *Escherichia coli* BL21-Gold(DE3)pLysS system. CPF_0394 is composed of 1,003 amino acid residues. The HA degradation ability was examined by the halo plate assay method using the cell extract obtained from the recombinant *E. coli* cells, resulting in the formation of a halo (Fig. 4a). Therefore, the recombinant enzyme was purified by metal affinity, anion exchange, and gel filtration chromatographies to homogeneity. As a result of SDS-PAGE followed by staining with coomassie brilliant blue (CBB), the molecular size of CPF_0394 indicated by a single band was approximately 113 kDa, consistent with the estimated value (Fig. 4b).

The absorbance at 235 nm increased in the CPF_0394 and HA reaction mixture. This indicates that CPF_0394 is a hyaluronate lyase (CpeHysA) directly acting on HA and producing unsaturated HA disaccharide with an absorbency at 235 nm, corresponding to C=C double bonds in unsaturated uronic acid residues. The specific activity of the purified CpeHysA was determined to be 76.3 units/mg. CpeHysA was further characterized by monitoring the absorbance increase at 235 nm (Fig. 4c-f). To determine the optimal pH, the enzyme activity was assayed in the presence of HA in different pH buffers at 30 °C. CpeHysA showed maximum activity at pH 5.5 and no activity below pH 4.5. The enzyme maintained more than 50% of activity in a pH range of 5.0–8.0. CpeHysA showed the highest activity at 60 °C, and almost all activity was lost at 70 °C. To assess the thermostability, CpeHysA was preincubated at different temperatures in Tris(hydroxymethyl) aminomethane-hydrochloride (Tris-HCl) for 10 min, followed by monitoring the enzyme activity in Tris-HCl at 30 °C. The activity was maintained at 55 °C, while the enzyme lost most activity at 60 °C, suggesting that the enzyme was more stable in the presence of the substrate. As shown in Fig. 4f, CpeHysA exhibited little degradation activity toward CSC and HP, indicating that the enzyme was specific for HA.

### Structural model of hyaluronate lyase CpeHysA

**Fig. 5:**
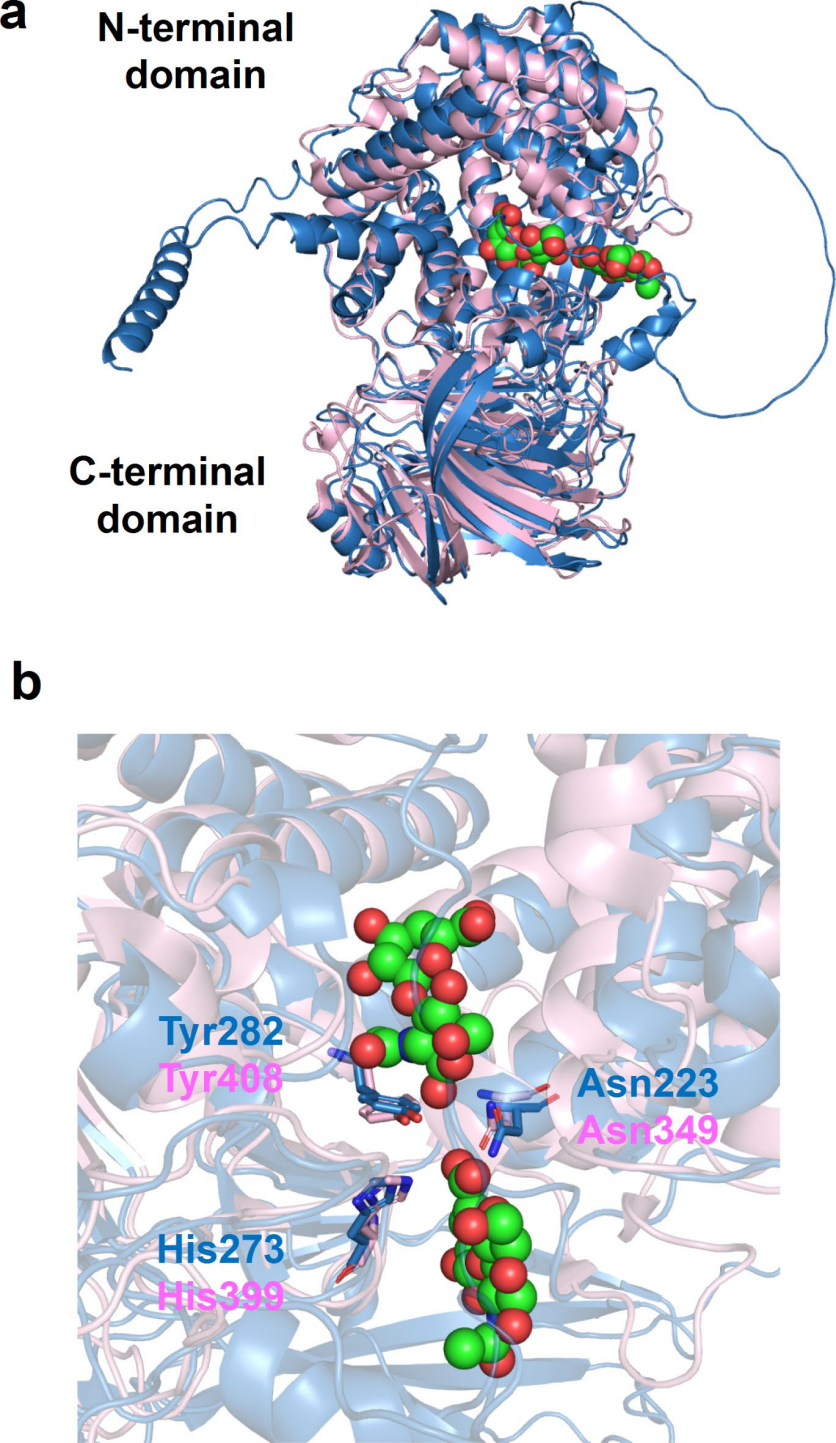
Structural model of hyaluronate lyase CpeHysA. **a** superimposition of CpeHysA and SpnHysA. Blue, the AlphaFold2 model of CpeHysA; pink, crystal structure of SpnHysA (PDB ID, 1C82). Ball models show unsaturated HA disaccharides. **b** the disaccharide-binding site of SpnHysA. Blue, the AlphaFold2 model of CpeHysA; pink, crystal structure of SpnHysA. Stick models indicate the conserved amino acid residues.

To investigate the structural conservation in HysA, we modeled the structure of CpeHysA using AlphaFold2^39^. The model structure was superimposed with the crystal structure of *S. pneumoniae* hyaluronate lyase (SpnHysA) in a complex with unsaturated HA disaccharide (PDB ID, 1C82) (Fig. 5a) because both CpeHysA and SpnHysA are categorized to polysaccharide lyase family 8 on the CAZy database^40^. While the sequence identity between CpeHysA and SpnHysA is 31%, the root-mean-square deviation is 1.5 Å, indicating that both structures are very similar. The length of amino acid residues of CpeHysA and SpnHysA are 1,003 and 1,066, respectively. However, the three-dimensional structure of SpnHysA consists of Ala168—Ala893, fully functional truncated length. Similar to SpnHysA, the CpeHysA model is divided into two domains (N- and C-terminal domains) linked by a short loop. Unsaturated HA disaccharide is bound to the cleft between N- and C-terminal domains. At the disaccharide-binding site of SpnHysA, Asn349 attracts electrons on the carboxylate group of uronic acid residue to make the C5 proton more acidic^41,42^. His399 removes a relatively acidic C5 proton by imidazole side chain, forming an unsaturated C = C (C4 and C5) double bond. Tyr408 provides a proton to the glycosidic bond oxygen to break the linkage. All three important residues (Asn349, His399, and Tyr408) are structurally conserved in the CpeHysA model (Asn223, His273, and Tyr282) (Fig. 5b), suggesting the physiological function of the enzyme for the degradation of HA through β-elimination reaction. Overall structure and three catalytically important residues of CpeHysA are also well conserved in our family 8 xanthan lyase (Supplementary Fig. 4)^43^.

### Identification of hyaluronan-degrading enzyme in *C. perfringens* strain ATCC 13124

**Fig. 6:**
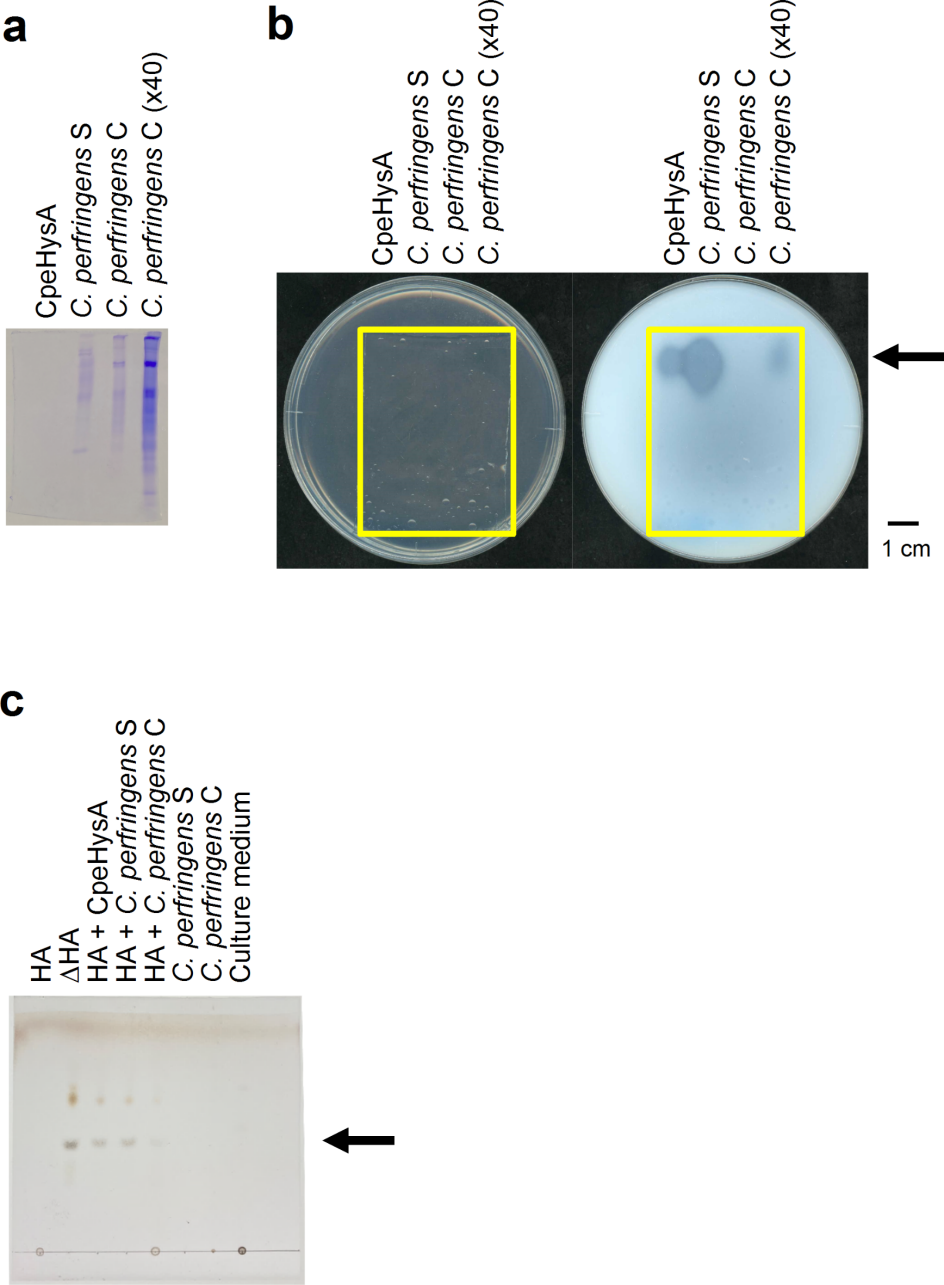
Hyaluronan-degrading activity of *C. perfringens* strain ATCC 13124. Native-PAGE followed by CBB staining (**a**) and halo-forming activity staining (Plates before (left) and after (right) addition of acetic acid. The yellow frame indicates the native-PAGE gel.) (**b**), and TLC (**c**). CpeHysA, the purified recombinant CpeHysA; *C. perfringens* S, the culture supernatant of *C. perfringens*; *C. perfringens* C, the cell extract of *C. perfringens*; *C. perfringens* C (x40), 40-fold concentrated *C. perfringens* C. HA, hyaluronan; ∆HA, unsaturated HA disaccharide.

To identify the intrinsic HA-degrading enzyme in *C. perfringens* strain ATCC 13124, the culture supernatant and cell extract of *C. perfringens* in addition to the purified recombinant CpeHysA were subjected to native-PAGE, followed by activity staining with halo plate assay method using HA as a substrate (Fig. 6a,b). Only one clear halo in the clostridial culture supernatant and cell extract was detected at the same position corresponding to CpeHysA on the native-PAGE gel, suggesting that the HA-degrading enzyme in *C. perfringens* is CpeHysA. Furthermore, the absorbance at 235 nm increased in the reaction mixtures of HA in the presence of the culture supernatant and cell extract, indicating that the HA-degrading enzyme was a hyaluronate lyase catalyzing a β-elimination reaction through production of unsaturated saccharides with C = C double bonds showing the absorbance at 235 nm. CpeHysA was predicted to contain a signal peptide cleaved by signal peptidase I with a program of SignalP-5.0 (https://services.healthtech.dtu.dk/services/SignalP-5.0/). In fact, the specific activity of HA-degrading enzyme in the culture supernatant (3.6 units/mg) was remarkably higher than that in the cell extract (0.0041 units/mg), demonstrating that *C. perfringens* secreted the enzyme extracellularly. Thin-layer chromatography (TLC) revealed that the enzyme released unsaturated HA disaccharide (Fig. 6c), indicating that unsaturated HA disaccharide was a main product, although there is a possibility that unsaturated tetra- or hexasaccharide was generated as an intermediate product during degradation of HA. According to the JSPS KAKENHI report (Grant number: 24590538, https://kaken.nii.ac.jp/ja/file/KAKENHI-PROJECT-24590538/24590538seika.pdf), the recombinant NagH, NagI, NagJ, NagK, and NagL of *C. perfringens* strain 13 genes have not shown HA-degrading activity and the recombinant HysA homolog of *C. perfringens* strain NCTC8237 (ATCC 13124) has exhibited HA-degrading activity. This report is consistent with our native-PAGE, followed by activity staining, enzyme assay, and TLC results. Therefore, CpeHysA was identified as the intrinsic HA-degrading enzyme in *C. perfringens*.

**Fig. 7:**
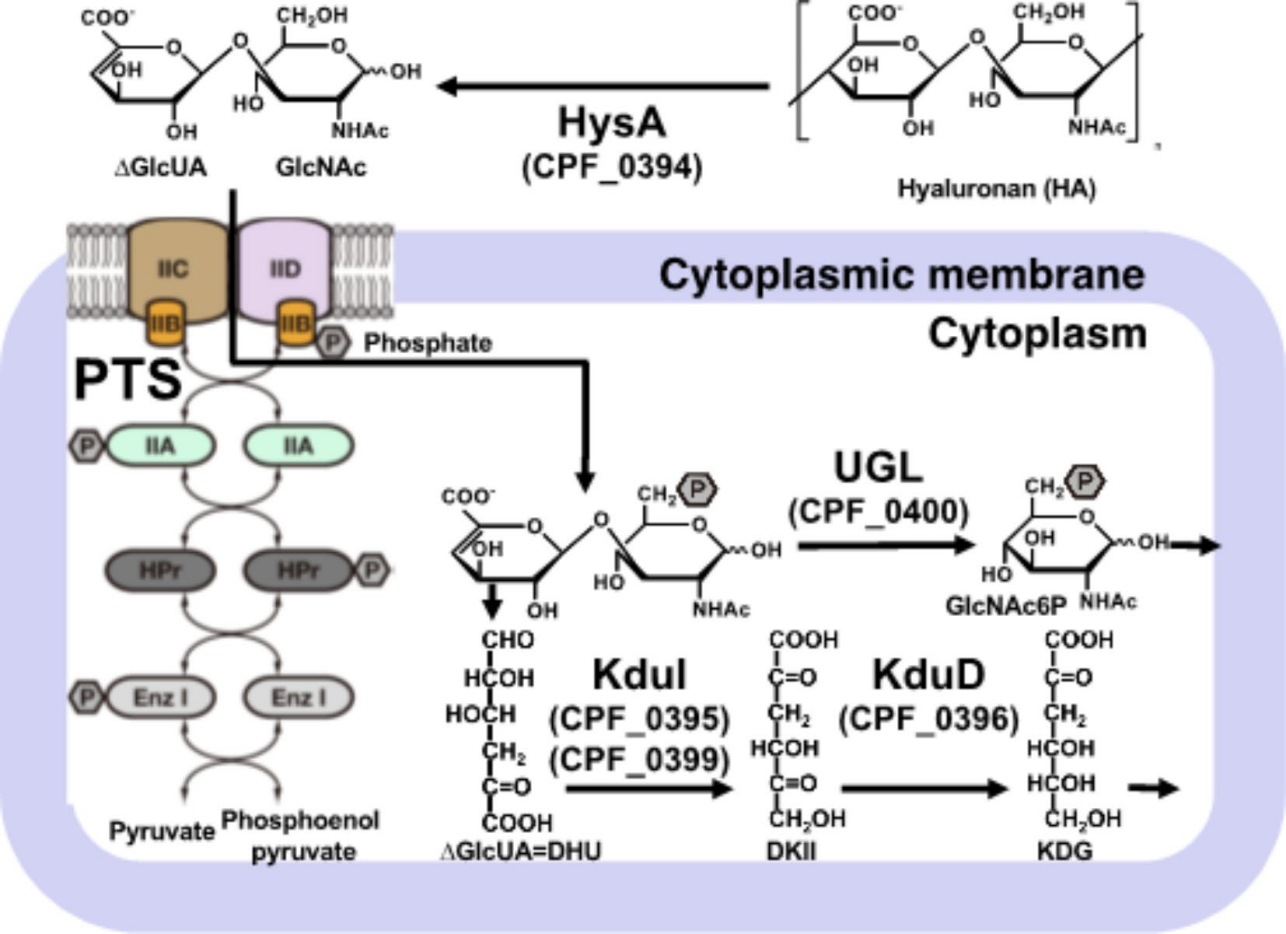
*C. perfringens* strain ATCC 13124 model for degradation, import, and metabolism of hyaluronan. HysA (CPF_0394) degrades HA to unsaturated HA disaccharides on the cell surface. PTS (CPF_0401—0404) in the cytoplasmic membrane imports unsaturated HA disaccharide into the cytoplasm through phosphorylation. In the cytoplasm, UGL (CPF_0400) degrades unsaturated HA disaccharides to unsaturated uronic acid and phosphorylated amino sugar. DHU, nonenzymatically converted from unsaturated uronic acid, is metabolized by KduI (CPF_0395 and CPF_0399) and KduD (CPF_0396). The resultant is finally metabolized into glycolysis.

In summary, we identified hyaluronate lyase (CpeHysA), not hyaluronidases, as an HA-degrading enzyme in *C. perfringens* strain ATCC 13124. Transcriptome analysis showed that HA increased the expression level of *hysA* and other constituent genes in the GAG genetic cluster but decreased *nagHJK*. A sole HA-degrading enzyme in the clostridial culture supernatant corresponded to CpeHysA, indicating that CpeHysA degrades HA as a virulence factor in *C. perfringens* (Fig. 7). Since HA induced the GAG genetic cluster, the resultant unsaturated HA disaccharide is likely degraded and metabolized as follows: PTS (CPF_0401—0404) imports unsaturated HA disaccharide into the cytoplasm through phosphorylation of the substrate. In the cytoplasm, UGL (CPF_0400) degrades unsaturated HA disaccharides to unsaturated uronic acid and phosphorylated amino sugar. 4-Deoxy-l-*threo*-5-hexosulose-uronic acid (DHU) is nonenzymatically converted from unsaturated uronic acid and is metabolized by KduI (CPF_0395 and CPF_0399), the complex structure of which with substrate analogs has recently reported in *Lactocaseibacillus*^44^, and KduD (CPF_0396). The resultant metabolites finally flow into glycolysis. This study is a significant finding that newly identified hyaluronate lyase as an HA-degrading enzyme distinct from conventional hyaluronidases in *C. perfringens*.

## Conclusion

In the current study, we focused on molecular identification of hyaluronan-degrading enzyme in *C. perfringens* strain ATCC 13124, an opportunistic pathogen causing gas gangrene in the human intestine and food poisoning by producing various toxins. A clostridial enzyme for direct degradation of hyaluronan, one of GAGs comprising the host extracellular matrices was found not to be well-known mu-toxin hyaluronidases including endo-β-*N*-acetylglucosaminidases such as NagH, NagJ, and NagK, but to be hyaluronate lyase HysA encoded in the GAG genetic cluster formed by the assembly of genes responsible for the degradation, import, and metabolism of GAGs. Based on the analysis of the primary structure and the higher enzyme activity in the extracellular fraction, CpeHysA is considered to be a secreted enzyme. On the other hand, a slight enzyme activity was observed in the cell extract. Intrinsic localization of CpeHysA is required to analyze by Western blotting and/or immunoelectron microscopy using anti-CpeHysA antibody. Interestingly, during this study, the GAG genetic cluster including *hysA* was demonstrated to be inducibly expressed in the presence of mucin as well as hyaluronan. Both of hyaluronan and mucin contain amino sugar and the GAG genetic cluster includes some genes coding for phosphotransferase system for import of amino sugar, suggesting that amino sugar may be a key molecule for expression of the GAG genetic cluster. This genetic cluster is conserved in some strains of *C. perfringens* and the cluster-deficient streptococci are known to reduce their colonization and/or infection to animal hosts^16,17^. The expression mechanism of the GAG genetic cluster in *C. perfringens* ATCC 13124 and involvement of the GAG genetic cluster in its pathogenicity are expected to be clarified in the future.

## Methods

### Materials

Sodium hyaluronate with a molecular weight of 1.5–1.8 × 10^6^ derived from *Streptococcus equi* was purchased from Sigma-Aldrich and its purity was extremely high (less than 1% protein). Sodium chondroitin sulfate C and sodium heparin were obtained from Nacalai Tesque. Mucin from the porcine stomach purchased from Fujifilm Wako Pure Chemical Co. was subjected to purification^45^. All other reagents of special grade were commercially available.

### Microorganisms and culture conditions

*C. perfringens* strain ATCC 13124 (JCM 1290), *C. butyricum* ATCC 25752 (JCM 1390), and *C. difficile* ATCC 9689 (JCM 1296) used for experiments were purchased from the RIKEN BioResource Center Microbial Materials Development Office (JCM). Gifu Anaerobic Medium (GAM) medium [1% peptone, 0.3% soy peptone, 1% protease peptone, 1.35% digested serum powder, 0.5% yeast extract, 0.22% meat extract, 0.12% liver extract, 0.3% glucose, 0.25% potassium dihydrogen phosphate, 0.3% sodium chloride, 0.5% soluble starch, 0.03% l-cysteine hydrochloride, and 0.03% sodium thioglycolate (pH 7.1)] was used as a standard medium under anaerobic conditions at 37 °C. The medium for assimilation assay and RNA-seq analysis of *C. perfringens* was prepared as follows: the nutrient-poor medium [0.1% potassium dihydrogen phosphate, 0.1% disodium hydrogen phosphate, 0.01% magnesium sulfate heptahydrate including 20-fold diluted GAM (0.05% peptone, 0.015% soy peptone, 0.05% protease peptone, 0.0675% digested serum powder, 0.025% yeast extract, 0.011% meat extract, 0.006% liver extract, 0.015% glucose, 0.015% sodium chloride, 0.025% soluble starch, 0.0015% l-cysteine hydrochloride, and 0.0015% sodium thioglycolate)] in the presence or absence of 0.2% HA or mucin. Briefly, *C. perfringens* cells grown in GAM medium overnight were harvested, washed, and suspended with saline to an optical density of 1 at 600 nm (OD_600_ = 1). The cell suspensions were inoculated into the nutrient-poor medium in the presence or absence of HA or mucin and cultured for a few days with periodical monitoring of OD_600_. For the degradation assay, 1% BSA and 1% agar were added to the nutrient-poor medium in the presence of HA. For the nutrient-rich halo plates, 0.2% CSC or HP, 1% BSA, and 1% agar were added to GAM medium. Because no white precipitate was detected in the nutrient-rich HA plate after the addition of acetic acid, a glucose-rich medium [0.1% potassium dihydrogenphosphate, 0.1% disodium hydrogenphosphate, 0.01% magnesium sulfate heptahydrate, 0.1% yeast extract, 5% glucose] containing 0.2% HA, 1% BSA, and 1% agar was alternatively used to detect HA degradation (Fig. 2b).

Luria-Bertani (LB) medium [1% tryptone, 0.5% yeast extract, and 1% sodium chloride (pH 7.2)] was used as a medium for *E. coli*. *E. coli* BL21-Gold(DE3)pLysS cells harboring the pET21b-CPF_0394 plasmid were cultured at 30 °C in LB medium containing 0.1 mg/mL sodium ampicillin to an OD_600_ = 0.3–0.7. Then, 1 mM isopropyl-β-d-thiogalactopyranoside (IPTG) was added and incubated at 16 °C for 2 days.

### Halo plate assay for hyaluronan degradation

The *C. perfringens* strain ATCC 13124 cell suspension was inoculated in the degradation assay medium and anaerobically cultured at 37 °C for 7 days^46^. After the addition of 2 M acetic acid 2 mL, the HA-degraded position formed a clear halo.

### RNA-seq analysis

RNA extraction from *C. perfringens* strain ATCC 13124 cells was subjected to the hot phenol method as follows. *C. perfringens* cells in the logarithmic growth phase were collected by centrifugation at 2600×*g* at 4 °C for 5 min and washed with saline. ISOGEN 300 µL (Nippon Gene) was added to the cell suspension, and the mixture was stirred with glass beads by vortexing for 4 min. After further addition of ISOGEN 700 µL, the mixture was incubated at 65 °C for 30 min. The supernatant was collected by centrifugation at 12,000×*g* at 4 °C for 15 min, followed by the addition of chloroform 200 µL. After vortexing for 15 s, the mixture was incubated at room temperature for 3 min and centrifuged at 12,000×*g* at 4 °C for 15 min. Isopropanol (500 µL) was added to the obtained aqueous layer and mixed by inversion, followed by incubation at room temperature for 5 min and centrifugation at 12,000×*g* at 4 °C for 10 min. Ethanol was added to the resulting precipitate and was centrifuged at 7500×*g* at 4 °C for 5 min to obtain a precipitate. The resultant precipitate was dried in a desiccator and dissolved with 100 µL of RNase-free water. Of the obtained RNA extract, 80 µL was used for RNA-seq, and 20 µL was used for purity and quality confirmation. The RNA extract was immediately frozen with liquid nitrogen and stored at − 80 °C.

The purity and quality of the RNA extract was confirmed by 0.9% agarose gel electrophoresis at 100 mV for 30 min. A quality check of RNA and RNA-seq analysis were performed by Macrogen Co. Japan. DNase treatment was added to the RNA extract to eliminate DNA contamination. To prepare the sequencing libraries, Ribo-Zero rRNA Removal Kit (Bacteria) and TruSeq Stranded Total RNA Sample Prep Kit (Illumina) were used. Paired-end (101 bp) RNA-seq analysis was performed on the Illumina NovaSeq 6000 sequencer. Trimmed reads were mapped to the reference genome (*C. perfringens* strain ATCC 13124) with Bowtie. After the read mapping, HTseq was used for expression profiling. The analysis of differentially expressed genes was performed on 3 comparison pairs using RPKM.

### Construction of plasmid for expression of CPF_0394, hyaluronate lyase CpeHysA

To construct the protein expression system in *E. coli*, the CPF_0394 (CpeHysA) gene was amplified via polymerase chain reaction (PCR) using *C. perfringens* strain ATCC 13124 genomic DNA as a template and oligonucleotides as In-Fusion primers (Supplementary Table 2). The reaction mixture contained PCR buffer for KOD FX Neo (TOYOBO), 4 nmol of dNTPs, 0.8 U of KOD FX Neo, 3 pmol of forward and reverse primers, and genomic DNA. The PCR reaction conditions were as follows: 94 °C for 2 min followed by 30 cycles of 98 °C for 10 s, 45.4 °C for 30 s, and 68 °C for 2 min. The CPF_0394 gene fragment and pET21b vector were digested with NdeI and XhoI mixed with In-Fusion HD Enzyme Premix Kit (Takara Bio), followed by incubation at 50 °C for 15 min. After the transformation of *E. coli* BL21-Gold(DE3)pLysS cells with the resultant plasmid, the nucleotide sequence of the objective gene was confirmed by DNA sequencing. DNA manipulations were carried out as described elsewhere^47^.

### Protein purification

*E. coli* BL21-Gold(DE3)pLysS cells harboring pET21b-CPF_0394 were cultured in LB containing ampicillin and IPTG, and harvested by centrifugation at 6800×*g* at 4 °C for 10 min. The obtained cells were suspended in 20 mM Tris-HCl (pH 7.5) and disrupted by an ultrasonic generator (Insonator Model 201 M, Kubota) at 9 kHz, 0 °C for 20 min. The disrupted cells were centrifuged at 20,000×*g* at 4 °C for 20 min to obtain supernatant as the cell extract. The cell extract was subjected to metal affinity chromatography using TALON resin (Clontech). The cell extract mixed with TALON resin was washed with equilibration buffer [20 mM Tris-HCl (pH 7.5), 500 mM NaCl, and 10 mM imidazole (pH 8.0)] and eluted with elution buffer [20 mM Tris-HCl (pH 7.5), 500 mM NaCl, and 500 mM imidazole (pH 8.0)]. Every eluted fraction was collected, and the purity of the protein in the fractions was assessed by SDS-PAGE^48^. Fractions containing partially purified protein were collected and applied to anion exchange chromatography using Resource Q (GE Healthcare). After washing with equilibration buffer [20 mM Tris-HCl (pH 7.5)], the proteins were eluted with gradient elution buffer [20 mM Tris-HCl (pH 7.5) and 0–1 M NaCl]. Fractions were selected by SDS-PAGE and applied to a gel filtration chromatography using HiLoad 16/60 Superdex 200 pg (GE Healthcare). The proteins were separated by molecular size with elution buffer [20 mM Tris-HCl (pH 7.5) and 0.15 M NaCl] and were subjected to SDS-PAGE, followed by protein staining with CBB. Collected fractions containing the purified protein were combined and dialyzed against 20 mM Tris-HCl (pH 7.5).

### Enzyme assay

The activity of hyaluronate lyase (CPF_0394, CpeHysA) was assayed by monitoring the increase in absorbance at 235 nm derived from the C = C double bonds in unsaturated HA disaccharide. The reaction mixture comprised 50 mM Tris-HCl (pH 7.5), 0.1% HA, and purified CpeHysA. To determine the optimal pH, the enzyme activity was measured in 50 mM buffer of sodium acetate (pH 4.0–5.5), potassium phosphate (pH 6.0–7.5), Tris-HCl (pH 7.5–8.5), and glycine-NaOH (pH 7.5–10.0) at 30 °C. The optimal temperature was determined by measuring the enzyme activity in 50 mM Tris-HCl (pH 7.5) at 30–70 °C. To examine the thermostability, the enzyme was preincubated for 10 min at 30–70 °C, followed by measuring the activity in 50 mM Tris-HCl (pH 7.5) at 30 °C.

To measure the intracellular and extracellular activity of HysA in *C. perfringens* strain ATCC 13124, the bacterial cells grown in 100 mL of the nutrient-poor medium containing HA were harvested to obtain cell pellet and culture supernatant. The resultant cell pellet was subjected to ultrasonication and centrifugation to obtain the cell extract. The culture supernatant and cell extract were subjected to concentration by ammonium sulfate precipitation, dialysis against 20 mM Tris-HCl (pH 7.5), and enzyme assay.

### TLC

The reaction mixture of HA in the presence of the culture supernatant or cell extract of *C. perfringens* strain ATCC 13124 was boiled and centrifuged. The resultant supernatant was subjected to TLC using a developing solvent of 1-butanol: acetic acid: water = 3:2:2. After spraying with 10% sulfuric acid in ethanol, the TLC plate was heated to visualize the HA degradation products.

### Native-PAGE

*C. perfringens* strain ATCC 13124 cells were grown in the nutrient-poor medium containing HA in the logarithmic growth phase and centrifuged to obtain the culture supernatant and bacterial cells. The resultant cells were washed and suspended in 20 mM Tris-HCl (pH 7.5). The cells were ultrasonically disrupted as described above, and the cell extract was obtained by centrifugation of the disrupted cells at 20,000×*g* at 4 °C for 20 min. The culture supernatant, cell extract, and the recombinant purified CpeHysA were subjected to native-PAGE with 6% separation gel at 0 °C in a running buffer consisting of 43 mM imidazole and 35 mM 4-(2-hydroxyethyl)-1-piperazineethanesulfonic acid (HEPES) (pH 7.4). After electrophoresis, the gel was placed on the halo assay plate and incubated at 37 °C for 24 h. The gel was removed, and acetic acid was added to confirm the degradation of HA.

### Statistics

Significant differences were statistically determined using Student’s *t*-test (**p < 0.01, *p < 0.05).

## Electronic supplementary material

Below is the link to the electronic supplementary material.

Supplementary Material 1

## Data Availability

RNA-seq data have been deposited to the GEO database under accession GSE240236 (https://www.ncbi.nlm.nih.gov/geo/query/acc.cgi?acc=GSE240236*).* All other data described are contained within the article.
