# Supplementary material for: Molecular identification of hyaluronate lyase, not hyaluronidase, as an intrinsic hyaluronan-degrading enzyme in Clostridium perfringens strain ATCC 13124
Source: Sci Rep. 2024 Oct 22;14:24266. doi: 10.1038/s41598-024-73955-y (PMC11496695; doi:10.1038/s41598-024-73955-y)
Supplement: Supplementary file 1 — Supplementary Material 1 [file 41598_2024_73955_MOESM1_ESM.pdf]

Supplementary Information

Molecular identification of hyaluronate lyase, not hyaluronidase, as an intrinsic hyaluronan-degrading enzyme in *Clostridium perfringens* strain ATCC 13124

Tomoya Kumon[1,2], Sayoko Oiki[1,2] & Wataru Hashimoto[1]

[1]Laboratory of Basic and Applied Molecular Biotechnology, Division of Food Science and Biotechnology, Graduate School of Agriculture, Kyoto University, Uji, Kyoto 611-0011, Japan

[2]These authors contributed equally to this work.

Correspondence and request for materials should be addressed to W.H. (e-mail: hashimoto.wataru.8c@kyoto-u.ac.jp).

Supplementary Fig. 1 Bacterial GAG degradation, import, and metabolism system.

Supplementary Fig. 2 Growth profile of *C. perfringens* cells by measuring OD_{600} in log10 form.

Supplementary Fig. 3 RNA-seq of *C. perfringens*.

Supplementary Fig. 4 Superimposition of CpeHysA and xanthan lyase.

Supplementary Table 1 Gene expression of *C. perfringens* through RNAseq.

Supplementary Table 2 Primers.

Supplementary Fig. 1

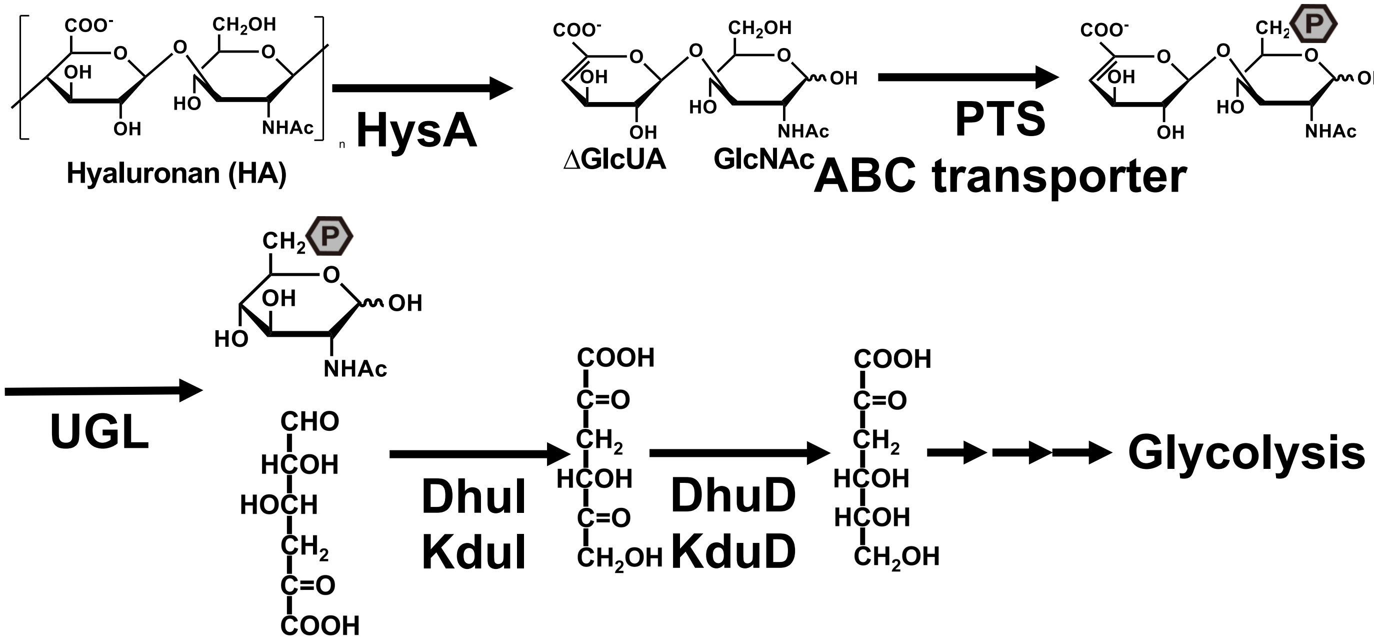

Supplementary Fig. 1 Bacterial GAG degradation, import, and metabolism system. Example of degradation enzymes, HysA and UGL; transporter, PTS and ABC transporter (non-phosphorylation of the substrate); metabolizing enzyme, DhuI/KduI and DhuD/KduD.

Supplementary Fig. 2

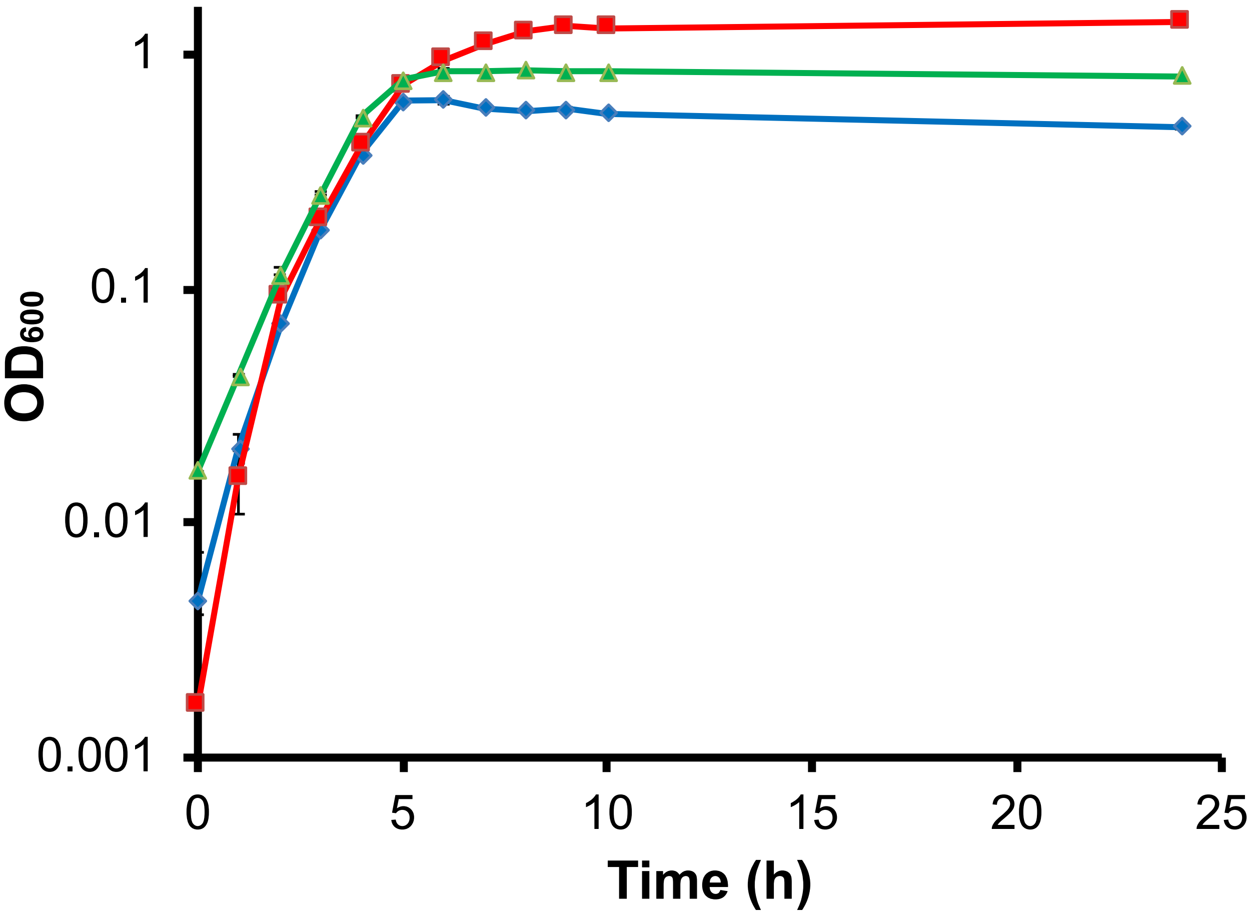

Supplementary Fig. 2 Growth profile of *C. perfringens* cells by measuring OD_{600} in log10 form. Blue, nutrient-poor medium; red, nutrient-poor medium containing HA; and green, nutrient-poor medium containing mucin. The profile is identical to Fig. 2c.

Supplementary Fig. 3

a

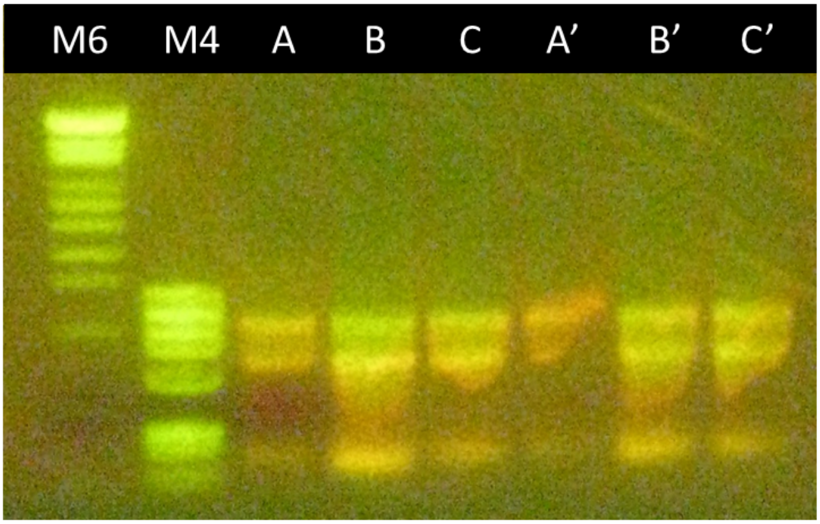

b

No.	growth medium	quantity (µg)	ratio(Abs260/Abs280)	RIN	rRNA ratio
A	Poor+	17.019	1.89	7.2	1.1
B	Poor+HA	36.716	1.9	7.3	1
C	Poor+mucin	33.961	1.97	7.2	1

No.	growth medium	total read bases	total reads
A	Poor+	5041504082	49915882
B	Poor+HA	5041270974	49913574
C	Poor+mucin	6955687796	68868196

No.	growth medium	total read bases	total reads
A	Poor+	4955895319	49366152
B	Poor+HA	4964571195	49416680
C	Poor+mucin	6842367613	68121182

No.	growth medium	processed data	mapped read		failed to aligned		multiple aligned	
A	Poor+	49366152	44776526	90.70%	2548788	5.16%	2040838	4.13%
B	Poor+HA	49416680	44736304	90.53%	2232878	4.52%	2447498	4.95%
C	Poor+mucin	68121182	60852264	89.33%	3693250	5.42%	3575668	5.25%

Supplementary Fig. 3 RNA-seq of *C. perfringens*. (**a**) Extracted RNA was applied to agarose gel electrophoresis. A, A ': Poor + medium B, B': Poor + HA medium C, C ': Poor + mucin medium. (**b**) Basic statistics of RNA-seq.

Supplementary Fig. 4

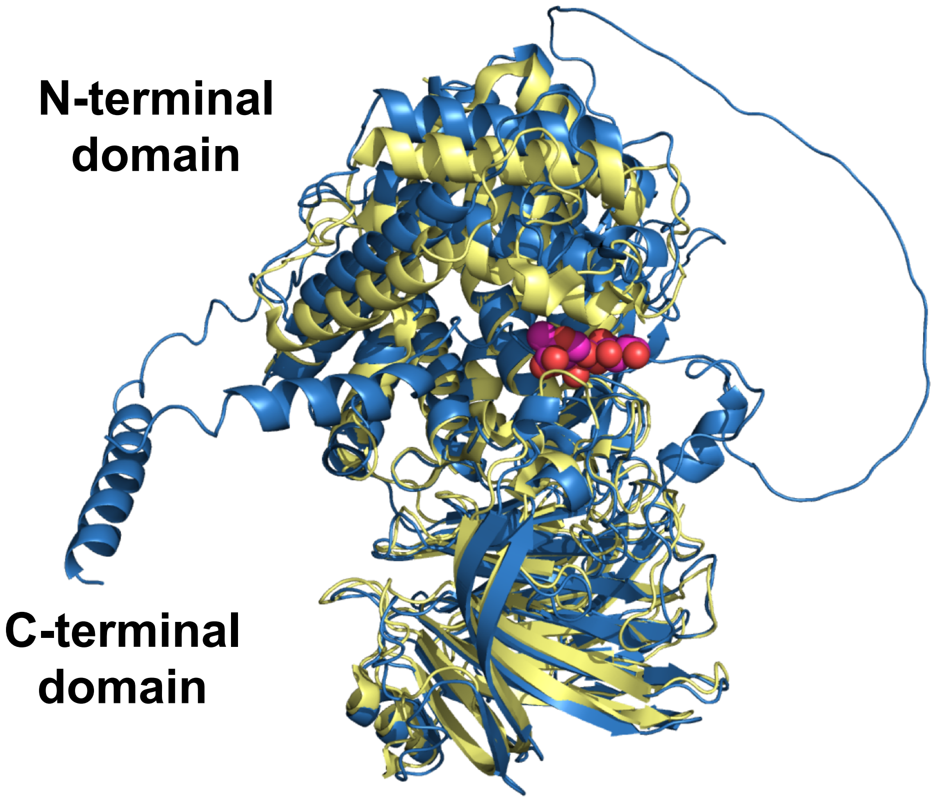

Supplementary Fig. 4 Superimposition of CpeHysA and xanthan lyase. Blue, the AlphaFold2 model of CpeHysA; yellow, crystal structure of *Bacillus* sp. GL1 xanthan lyase (PDB ID, 1X1J). Ball model shows xanthan disaccharide.

Supplementary Table 1 Gene expression of *C. perfringens* through RNAseq

Feature_ID	Chrom	Start	End	Strand	Type	Locus_Tag	Old_Locus_Tag	Gene_Symbol	Protein_ID	Product	B-1/A-1.fc	B-1/A-1.volum	B-1/C-1.fc	B-1/C-1.volum	C-1/A-1.fc	C-1/A-1.volum	A-1.mean	B-1.mean	C-1.mean	N_A-1	N_B-1	N_C-1	A-1_RPKM	B-1_RPKM	C-1_RPKM	A-1_Read_Coun	B-1_Read_Coun	C-1_Read_Count
gene0	NC_008261.1	411	1784	+	protein_coding	CPF_RS00010	CPF_0001	.	WP_003451025.	chromosomal re	1.098464	6.028179	-1.024235	6.113552	1.125084	6.045235	5.960817	6.096304	6.130850	5.960817	6.096304	6.130850	47.407668	98.280023	61.065514	1378	2731	2373
gene1	NC_008261.1	2079	3179	+	protein_coding	CPF_RS00015	CPF_0002	.	WP_003451044.	DNA polymerase	1.618265	7.657631	1.299002	7.821746	1.245776	7.475117	7.318275	8.012723	7.635320	7.318275	8.012723	7.635320	129.745782	303.636445	191.272434	3022	6761	5956
gene2	NC_008261.1	3305	3511	+	protein_coding	CPF_RS00020	CPF_0003	.	WP_003470354.	S4 domain-cont	1.262922	6.382229	-1.107484	6.626066	1.398666	6.453556	6.216067	6.552833	6.700118	6.216067	6.552833	6.700118	58.459585	131.377993	93.433366	256	550	547
gene3	NC_008261.1	3567	4652	+	protein_coding	CPF_RS00025	CPF_0004	.	WP_003450989.	DNA replication	1.984433	6.158424	1.836505	6.218677	1.080549	5.739481	5.683871	6.672598	5.795635	5.683871	6.672598	5.795635	39.261132	140.142245	47.371614	902	3078	1455
gene4	NC_008261.1	4666	4926	+	protein_coding	CPF_RS00030	CPF_0005	.	WP_003451015.	DUF370 domain	1.615977	6.717605	1.579727	6.734814	1.022947	6.396662	6.380317	7.072724	6.413049	6.380317	7.072724	6.413049	66.467855	173.913163	74.373266	367	918	549
gene5	NC_008261.1	5163	7079	+	protein_coding	CPF_RS00035	CPF_0006	.	WP_003466052.	DNA topoisomer	1.639879	7.579866	1.681323	7.560971	-1.025273	7.213437	7.231464	7.945053	7.195456	7.231464	7.945053	7.195456	123.932862	294.044472	133.278734	5026	11400	7226
gene6	NC_008261.1	7101	9620	+	protein_coding	CPF_RS00040	CPF_0007	.	WP_003470357.	DNA gyrase sub	1.796680	8.171432	1.744321	8.193867	1.030017	7.780994	7.759689	8.605023	7.802357	7.759689	8.605023	7.802357	177.656624	467.852391	219.302434	9471	23844	15630
gene8	NC_008261.1	11863	14775	+	rRNA	CPF_RS00050	CPF_2999	.	.	23S ribosomal R	-3.033647	5.312929	-4.338669	5.530594	1.430183	6.426346	6.173427	4.572374	6.689627	6.173427	4.572374	6.689627	56.357330	33.218546	93.219281	3473	1957	7680
gene9	NC_008261.1	14868	14986	+	rRNA	CPF_RS00055	CPF_3000	rrf	.	5S ribosomal RN	22.783309	7.285976	1.188258	9.756678	19.173704	7.193652	5.371990	9.881895	9.633047	5.371990	9.881895	9.633047	31.778188	1018.834834	795.995782	80	2452	2679
gene12	NC_008261.1	15422	15925	+	protein_coding	CPF_RS00070	CPF_0010	.	WP_011590026.	HD domain-con	2.703108	2.566998	1.854937	2.902946	1.457251	2.202967	1.948025	3.382644	2.491274	1.948025	3.382644	2.491274	2.157165	14.127400	4.139105	23	144	59
gene13	NC_008261.1	16274	19030	+	protein_coding	CPF_RS00075	CPF_0011	.	WP_011590027.	membrane prote	1.347566	4.915391	1.402141	4.885380	-1.040499	4.676195	4.704920	5.135276	4.647645	4.704920	5.135276	4.647645	19.751579	49.768724	20.699115	1152	2775	1614
gene14	NC_008261.1	19273	20667	+	protein_coding	CPF_RS00080	CPF_0012	.	WP_003458179.	FAD/NAD(P)-bi	-1.274682	5.745162	-1.118522	5.652979	-1.139613	5.827863	5.922897	5.572760	5.734353	5.922897	5.572760	5.734353	45.846871	68.728028	45.293446	1353	1939	1787
gene15	NC_008261.1	20680	20913	+	protein_coding	CPF_RS00085	CPF_0013	.	WP_003450903.	DUF1667 domai	-2.357753	5.539010	-2.054363	5.449405	-1.147680	6.091993	6.192164	4.954751	5.993443	6.192164	4.954751	5.993443	57.572503	43.951911	54.849893	285	208	363
gene16	NC_008261.1	21265	22542	+	protein_coding	CPF_RS00090	CPF_0014	.	WP_011590028.	serine--tRNA li	1.513378	7.747692	1.702984	7.658686	-1.125287	7.368931	7.454569	8.052341	7.284276	7.454569	8.052341	7.284276	141.181377	313.699023	143.616781	3817	8108	5191
gene17	NC_008261.1	22719	23138	+	protein_coding	CPF_RS00095	CPF_0015	.	WP_011590029.	hypothetical pro	-1.120441	0.847262	-1.000637	0.769650	-1.119728	0.847768	0.933257	0.769191	0.770109	0.933257	0.769191	0.770109	0.675287	1.059555	0.589296	6	9	7
gene20	NC_008261.1	23755	24021	+	protein_coding	CPF_RS00110	CPF_0018	.	WP_003450912.	hypothetical pro	1.461416	2.464852	2.053130	2.173691	-1.404890	1.945695	2.206316	2.753684	1.715859	2.206316	2.753684	1.715859	2.655621	8.333579	1.986392	15	45	15
gene24	NC_008261.1	24771	25652	+	protein_coding	CPF_RS00130	CPF_0022	.	WP_003450881.	hypothetical pro	-1.120611	1.067925	1.110009	0.910543	-1.243889	0.983270	1.153222	0.988936	0.838365	1.153222	0.988936	0.838365	0.911101	1.401528	0.681499	17	25	17
gene25	NC_008261.1	26114	29590	+	protein_coding	CPF_RS00135	CPF_0024	.	WP_011590030.	ATP-dependent	-1.456416	4.042558	-1.362623	3.997402	-1.068833	4.274569	4.322857	3.780434	4.226821	4.322857	3.780434	4.226821	14.410773	19.226591	15.375602	1060	1352	1512
gene26	NC_008261.1	29580	33395	+	protein_coding	CPF_RS00140	CPF_0025	.	WP_011590031.	ATP-dependent	-1.851039	4.531557	-1.816453	4.519203	-1.019041	4.983816	4.997440	4.109105	4.970229	4.997440	4.109105	4.970229	24.242007	24.761822	26.703650	1957	1911	2882
gene27	NC_008261.1	33434	34702	-	protein_coding	CPF_RS00145	CPF_0026	.	WP_041702735.	CCA tRNA nuck	2.125827	3.449314	2.978388	3.151863	-1.401049	2.693723	2.947938	4.035963	2.461431	2.947938	4.035963	2.461431	5.103229	23.690391	4.067956	137	608	146
gene28	NC_008261.1	34818	35810	+	protein_coding	CPF_RS00150	CPF_0027	.	WP_003472587.	3'-5' exoribonuc	1.168759	8.054497	1.278304	7.988692	-1.093728	7.877902	7.942794	8.167771	7.813540	7.942794	8.167771	7.813540	209.025994	338.104387	220.763594	4391	6790	6200
gene29	NC_008261.1	35842	37002	-	protein_coding	CPF_RS00155	CPF_0028	.	WP_003458221.	murein L,D-tran	3.678660	2.874327	2.547023	3.219304	1.444298	2.334583	2.084411	3.963592	2.614780	2.084411	3.963592	2.614780	2.402182	22.274079	4.598643	59	523	151
gene30	NC_008261.1	37194	38414	+	protein_coding	CPF_RS00160	CPF_0029	.	WP_003474863.	peptidase T	-2.114090	[illegible]	[illegible]	[illegible]	[illegible]	[illegible]	[illegible]	[illegible]	[illegible]	[illegible]	[illegible]	[illegible]	[illegible]	[illegible]	[illegible]	[illegible]	[illegible]	[illegible]
gene31	NC_008261.1	38520	39395	-	protein_coding	CPF_RS00165	CPF_0030	.	WP_011590034.	DegV family prot	1.295047	[illegible]	[illegible]	[illegible]	[illegible]	[illegible]	[illegible]	[illegible]	[illegible]	[illegible]	[illegible]	[illegible]	[illegible]	[illegible]	[illegible]	[illegible]	[illegible]	[illegible]
gene33	NC_008261.1	39832	40971	+	protein_coding	CPF_RS00175	CPF_0032	.	WP_011590035.	DUF1887 domai	2.181674	[illegible]	[illegible]	[illegible]	[illegible]	[illegible]	[illegible]	[illegible]	[illegible]	[illegible]	[illegible]	[illegible]	[illegible]	[illegible]	[illegible]	[illegible]	[illegible]	[illegible]
gene34	NC_008261.1	41086	41970	-	protein_coding	CPF_RS00180	CPF_0033	.	WP_011590036.	phosphatidylseri	2.559467	[illegible]	[illegible]	[illegible]	[illegible]	[illegible]	[illegible]	[illegible]	[illegible]	[illegible]	[illegible]	[illegible]	[illegible]	[illegible]	[illegible]	[illegible]	[illegible]	[illegible]
gene35	NC_008261.1	42133	42702	+	protein_coding	CPF_RS00185	CPF_0034	.	WP_011009579.	TetR/AcrR fami	-1.269635	[illegible]	[illegible]	[illegible]	[illegible]	[illegible]	[illegible]	[illegible]	[illegible]	[illegible]	[illegible]	[illegible]	[illegible]	[illegible]	[illegible]	[illegible]	[illegible]	[illegible]
gene36	NC_008261.1	42757	42831	+	tRNA	CPF_RS00190	CPF_0035	.	.	tRNA-Arg	-2.394466	[illegible]	[illegible]	[illegible]	[illegible]	[illegible]	[illegible]	[illegible]	[illegible]	[illegible]	[illegible]	[illegible]	[illegible]	[illegible]	[illegible]	[illegible]	[illegible]	[illegible]
gene37	NC_008261.1	42977	44242	+	protein_coding	CPF_RS00195	CPF_0036	.	WP_011590037.	HlyC/CorC famil	-1.127397	[illegible]	[illegible]	[illegible]	[illegible]	[illegible]	[illegible]	[illegible]	[illegible]	[illegible]	[illegible]	[illegible]	[illegible]	[illegible]	[illegible]	[illegible]	[illegible]	[illegible]
gene38	NC_008261.1	44245	44676	+	protein_coding	CPF_RS00200	CPF_0037	.	WP_003450857.	nucleoside dean	-1.286340	[illegible]	[illegible]	[illegible]	[illegible]	[illegible]	[illegible]	[illegible]	[illegible]	[illegible]	[illegible]	[illegible]	[illegible]	[illegible]	[illegible]	[illegible]	[illegible]	[illegible]
gene39	NC_008261.1	44739	45197	+	protein_coding	CPF_RS00205	CPF_0038	.	WP_003450846.	hypothetical pro	-1.428783	[illegible]	[illegible]	[illegible]	[illegible]	[illegible]	[illegible]	[illegible]	[illegible]	[illegible]	[illegible]	[illegible]	[illegible]	[illegible]	[illegible]	[illegible]	[illegible]	[illegible]
gene40	NC_008261.1	45310	45675	+	protein_coding	CPF_RS00210	CPF_0039	.	WP_003450965.	DUF2809 domai	1.027488	[illegible]	[illegible]	[illegible]	[illegible]	[illegible]	[illegible]	[illegible]	[illegible]	[illegible]	[illegible]	[illegible]	[illegible]	[illegible]	[illegible]	[illegible]	[illegible]	[illegible]
gene41	NC_008261.1	45785	46447	+	protein_coding	CPF_RS00215	CPF_0040	.	WP_003458203.	hypothetical pro	1.243154	[illegible]	[illegible]	[illegible]	[illegible]	[illegible]	[illegible]	[illegible]	[illegible]	[illegible]	[illegible]	[illegible]	[illegible]	[illegible]	[illegible]	[illegible]	[illegible]	[illegible]
gene42	NC_008261.1	46559	48031	-	protein_coding	CPF_RS00220	CPF_0041	.	WP_011590040.	YdiU family prote	1.007016	[illegible]	[illegible]	[illegible]	[illegible]	[illegible]	[illegible]	[illegible]	[illegible]	[illegible]	[illegible]	[illegible]	[illegible]	[illegible]	[illegible]	[illegible]	[illegible]	[illegible]
gene43	NC_008261.1	48363	49559	+	protein_coding	CPF_RS00225	CPF_0042	.	WP_011590041.	phospholipase C	2.190633	[illegible]	[illegible]	[illegible]	[illegible]	[illegible]	[illegible]	[illegible]	[illegible]	[illegible]	[illegible]	[illegible]	[illegible]	[illegible]	[illegible]	[illegible]	[illegible]	[illegible]
gene44	NC_008261.1	49747	50673	+	protein_coding	CPF_RS00230	CPF_0043	.	WP_003458162.	GTP-binding pro	1.025381	[illegible]	[illegible]	[illegible]	[illegible]	[illegible]	[illegible]	[illegible]	[illegible]	[illegible]	[illegible]	[illegible]	[illegible]	[illegible]	[illegible]	[illegible]	[illegible]	[illegible]
gene45	NC_008261.1	50693	51277	+	protein_coding	CPF_RS00235	CPF_0044	.	WP_011590042.	hypothetical pro	-1.195866	[illegible]	[illegible]	[illegible]	[illegible]	[illegible]	[illegible]	[illegible]	[illegible]	[illegible]	[illegible]	[illegible]	[illegible]	[illegible]	[illegible]	[illegible]	[illegible]	[illegible]
gene46	NC_008261.1	51280	52173	+	protein_coding	CPF_RS00240	CPF_0045	.	WP_003468662.	permease	1.019234	[illegible]	[illegible]	[illegible]	[illegible]	[illegible]	[illegible]	[illegible]	[illegible]	[illegible]	[illegible]	[illegible]	[illegible]	[illegible]	[illegible]	[illegible]	[illegible]	[illegible]
gene47	NC_008261.1	52186	52905	+	protein_coding	CPF_RS00245	CPF_0046	.	WP_003468577.	hypothetical pro	1.310191	[illegible]	[illegible]	[illegible]	[illegible]	[illegible]	[illegible]	[illegible]	[illegible]	[illegible]	[illegible]	[illegible]	[illegible]	[illegible]	[illegible]	[illegible]	[illegible]	[illegible]
gene48	NC_008261.1	53171	53959	+	protein_coding	CPF_RS00250	CPF_0047	.	WP_003468570.	diacylglyceryl tra	1.599585	[illegible]	[illegible]	[illegible]	[illegible]	[illegible]	[illegible]	[illegible]	[illegible]	[illegible]	[illegible]	[illegible]	[illegible]	[illegible]	[illegible]	[illegible]	[illegible]	[illegible]
gene50	NC_008261.1	54302	54566	+	SRP_RNA	CPF_RS14700	.	ffs	.	signal recognitio	-1.114748	[illegible]	[illegible]	[illegible]	[illegible]	[illegible]	[illegible]	[illegible]	[illegible]	[illegible]	[illegible]	[illegible]	[illegible]	[illegible]	[illegible]	[illegible]	[illegible]	[illegible]
gene51	NC_008261.1	54732	55235	+	protein_coding	CPF_RS00260	CPF_0049	.	WP_003468616.	hypothetical pro	-1.042532	[illegible]	[illegible]	[illegible]	[illegible]	[illegible]	[illegible]	[illegible]	[illegible]	[illegible]	[illegible]	[illegible]	[illegible]	[illegible]	[illegible]	[illegible]	[illegible]	[illegible]
gene52	NC_008261.1	55675	56961	+	protein_coding	CPF_RS00265	CPF_0050	.	WP_003458178.	branched-chain	4.145993	[illegible]	[illegible]	[illegible]	[illegible]	[illegible]	[illegible]	[illegible]	[illegible]	[illegible]	[illegible]	[illegible]	[illegible]	[illegible]	[illegible]	[illegible]	[illegible]	[illegible]
gene54	NC_008261.1	57783	59426	+	protein_coding	CPF_RS00275	CPF_0052	.	WP_003479629.	DNA polymerase	-1.040051	[illegible]	[illegible]	[illegible]	[illegible]	[illegible]	[illegible]	[illegible]	[illegible]	[illegible]	[illegible]	[illegible]	[illegible]	[illegible]	[illegible]	[illegible]	[illegible]	[illegible]
gene55	NC_008261.1	59538	59876	+	protein_coding	CPF_RS00280	CPF_0053	.	WP_003458189.	YbaB/EbfC famil	1.488014	[illegible]	[illegible]	[illegible]	[illegible]	[illegible]	[illegible]	[illegible]	[illegible]	[illegible]	[illegible]	[illegible]	[illegible]	[illegible]	[illegible]	[illegible]	[illegible]	[illegible]
gene56	NC_008261.1	59974	60570	+	protein_coding	CPF_RS00285	CPF_0054	.	WP_003450875.	recombination p	1.801324	[illegible]	[illegible]	[illegible]	[illegible]	[illegible]	[illegible]	[illegible]	[illegible]	[illegible]	[illegible]	[illegible]	[illegible]	[illegible]	[illegible]	[illegible]	[illegible]	[illegible]
[illegible]	[illegible]	[illegible]	[illegible]	[illegible]	[illegible]	[illegible]	[illegible]	[illegible]	[illegible]	[illegible]	[illegible]	[illegible]	[illegible]	[illegible]	[illegible]	[illegible]	[illegible]	[illegible]	[illegible]	[illegible]	[illegible]	[illegible]	[illegible]	[illegible]	[illegible]	[illegible]	[illegible]	[illegible]

gene279	NC_008261.1	326518	327867	-	protein_coding	CPF_RS01390	CPF_0270	.	WP_003476170. iron hydrogenas	-1.014335	0.549336	-1.165422	0.640137	1.148952	0.652213	0.559699	0.539165	0.760017	0.559699	0.539165	0.760017	0.350149	0.659279	0.576201	10	18	22
gene281	NC_008261.1	328565	328855	+	protein_coding	CPF_RS01400	CPF_0273	.	WP_003479074. hypothetical pro	-1.229361	4.405432	-1.092554	4.322376	-1.125218	4.470991	4.556903	4.258995	4.386699	4.556903	4.258995	4.386699	17.868406	27.696500	17.132121	110	163	141
gene282	NC_008261.1	329040	330338	+	protein_coding	CPF_RS01405	CPF_0274	.	WP_011590118. SagA protein	2.095511	10.039724	2.544625	9.890902	-1.214322	9.379124	9.520246	10.587548	9.240095	9.520246	10.587548	9.240095	659.888513	1709.632890	608.730899	18134	44914	22364
gene283	NC_008261.1	330512	331282	+	protein_coding	CPF_RS01410	CPF_0275	.	WP_041702538. tRNA1(Val) (ade	1.118786	4.024060	1.335530	3.891543	-1.193732	3.814030	3.943908	4.105842	3.688429	3.943908	4.105842	3.688429	10.974500	24.755016	10.226700	179	386	223
gene284	NC_008261.1	331285	332127	+	protein_coding	CPF_RS01415	CPF_0276	.	WP_003453470. 16S rRNA (cytic	-1.388392	4.056077	-1.290040	4.005769	-1.076240	4.246356	4.299686	3.826271	4.193687	4.299686	3.826271	4.193687	14.242697	19.883938	14.889695	254	339	355
gene285	NC_008261.1	332413	332652	-	protein_coding	CPF_RS01420	CPF_0277	.	WP_003476163. AbrB/MazE/Spc	1.170006	12.175077	1.124450	12.203958	1.040514	12.090960	12.062345	12.288861	12.119642	12.062345	12.288861	12.119642	3684.306874	5736.342400	4187.981958	18706	27843	28427
gene286	NC_008261.1	333343	333942	+	protein_coding	CPF_RS01425	CPF_0278	.	WP_003458091. DUF1836 domai	2.173360	8.373109	1.971464	8.447965	1.102409	7.901866	7.831848	8.951776	7.972509	7.831848	8.951776	7.972509	189.789271	592.773924	242.436526	2409	7193	4114
gene287	NC_008261.1	333978	334853	-	protein_coding	CPF_RS01430	CPF_0279	.	WP_003458008. aldose 1-epimer	1.258070	6.416563	1.780645	6.154048	-1.415378	5.997267	6.253093	6.584306	5.751906	6.253093	6.584306	5.751906	60.706406	134.677986	45.892464	1125	2386	1137
gene288	NC_008261.1	334964	335986	-	protein_coding	CPF_RS01435	CPF_0280	.	WP_011590120. LytR family trans	1.690999	7.373281	2.176206	7.179164	-1.286935	6.819677	7.004074	7.761950	6.640135	7.004074	7.761950	6.640135	105.167786	266.079840	88.688239	2276	5505	2566
gene289	NC_008261.1	336162	337013	+	protein_coding	CPF_RS01440	CPF_0281	.	WP_003457966. YihY/virulence f	1.239592	3.197260	1.548172	3.024272	-1.248936	2.881270	3.046079	3.355944	2.725379	3.046079	3.355944	2.725379	5.492647	13.754317	5.145968	99	237	124
gene290	NC_008261.1	337074	338060	-	protein_coding	CPF_RS01445	CPF_0282	.	WP_011590121. UDP-glucose 4-	1.142425	7.263560	1.629470	6.999187	-1.426326	6.907245	7.168145	7.360244	6.655842	7.168145	7.360244	6.655842	119.587971	212.161483	90.024424	2497	4235	2513
gene291	NC_008261.1	338214	338558	+	protein_coding	CPF_RS01450	CPF_0283	.	WP_003457980. single-stranded	-1.681141	1.147912	1.105596	0.756937	-1.858663	1.043338	1.582246	0.832805	0.687981	1.582246	0.832805	0.687981	1.507161	1.146572	0.512432	11	8	5
gene292	NC_008261.1	338634	339833	-	protein_coding	CPF_RS01455	CPF_0284	.	WP_003458024. MBL fold metallo	-1.068493	11.517205	1.066664	11.422867	-1.139723	11.470363	11.565093	11.469515	11.376410	11.565093	11.469515	11.376410	2628.845328	3382.552770	2580.027890	66736	82091	87563
gene293	NC_008261.1	340337	343744	-	protein_coding	CPF_RS01460	CPF_0285	.	WP_011590122. PKD domain-co	-2.764495	2.488346	-7.119102	2.954847	2.575191	3.951573	3.327713	1.860697	4.692392	3.327713	1.860697	4.692392	6.879679	3.830316	21.579867	496	264	2080
gene294	NC_008261.1	344115	344420	+	protein_coding	CPF_RS01465	CPF_0286	.	WP_003458000. hypothetical pro	2.942980	4.532880	-1.584954	5.700456	4.664488	4.804746	3.820630	5.377908	6.042349	3.820630	5.377908	6.042349	10.041025	59.141174	56.618671	65	366	490
gene295	NC_008261.1	344469	344870	+	protein_coding	CPF_RS01470	CPF_0287	.	WP_004457509. holo-ACP synth	2.148858	4.132667	-1.874411	5.154457	4.027843	4.511986	3.617556	4.721125	5.627563	3.617556	4.721125	5.627563	8.466279	37.022925	41.954383	72	301	477
gene296	NC_008261.1	344857	346356	+	protein_coding	CPF_RS01475	CPF_0288	.	WP_011590123. bifunctional ADF	1.735494	4.995613	-2.188712	5.947350	3.798498	5.492728	4.613743	5.409090	6.539172	4.613743	5.409090	6.539172	18.498348	60.719564	82.690066	587	1842	3508
gene297	NC_008261.1	346417	347010	+	protein_coding	CPF_RS01480	CPF_0289	.	WP_003458078. hypothetical pro	1.935669	4.780317	-1.817211	5.695002	3.517519	5.155640	4.327583	5.280415	6.142141	4.327583	5.280415	6.142141	14.483418	55.272857	61.310632	182	664	1030
gene298	NC_008261.1	347257	347499	+	protein_coding	CPF_RS01485	CPF_0290	.	WP_003465652. hypothetical pro	1.168533	7.823756	1.403525	7.688497	-1.201100	7.578883	7.712214	7.936912	7.447857	7.712214	7.936912	7.447857	172.350900	291.995332	168.349604	886	1435	1157
gene299	NC_008261.1	347505	347858	+	protein_coding	CPF_RS01490	CPF_0291	.	WP_003458025. type II toxin-ant	1.021892	7.837450	1.032603	7.829909	-1.010482	7.814319	7.821844	7.853087	7.806800	7.821844	7.853087	7.806800	187.477845	278.517298	219.937390	1404	1994	2202
gene300	NC_008261.1	348296	349120	+	protein_coding	CPF_RS01495	CPF_0292	.	WP_011590124. transketolase	1.035386	10.294017	1.739758	9.911644	-1.680298	9.887520	10.268963	10.319133	9.520246	10.268963	10.319133	9.520246	1049.395222	1417.688853	728.413168	18315	23654	16996
gene301	NC_008261.1	349120	350064	+	protein_coding	CPF_RS01500	CPF_0293	.	WP_003458019. transketolase	1.083802	10.483934	1.751283	10.129878	-1.615870	10.073943	10.426044	10.542145	9.733733	10.426044	10.542145	9.733733	1176.649212	1674.463144	863.665273	23523	32002	23083
gene302	NC_008261.1	350291	351148	+	protein_coding	CPF_RS01505	CPF_0295	.	WP_003458017. YitT family prote	-2.884326	4.785685	-1.583906	4.401445	-1.821022	5.159963	5.610421	4.082186	4.745673	5.610421	4.082186	4.745673	37.628727	24.434803	22.170730	683	424	538
gene303	NC_008261.1	351354	352040	+	protein_coding	CPF_RS01510	CPF_0296	.	WP_003458050. cell division ATP	1.762301	9.311226	2.344188	9.093637	-1.330186	8.703215	8.911462	9.728923	8.499835	8.911462	9.728923	8.499835	408.916939	940.767614	357.695175	5943	13071	6950
gene304	NC_008261.1	352030	352938	+	protein_coding	CPF_RS01515	CPF_0297	.	WP_003457959. ABC transporte	1.442942	9.654316	1.817667	9.481609	-1.259695	9.225392	9.393432	9.922446	9.060357	9.393432	9.922446	9.060357	581.073262	1055.879376	527.371627	11174	19411	13558
gene305	NC_008261.1	353027	354313	+	protein_coding	CPF_RS01520	CPF_0298	.	WP_011590126. S41 family pepti	1.388284	5.613301	1.380423	5.617572	1.005694	5.385730	5.381636	5.854939	5.389828	5.381636	5.854939	5.389828	31.954116	84.715002	35.659985	870	2205	1298
gene306	NC_008261.1	354336	355616	+	protein_coding	CPF_RS01525	CPF_0299	.	WP_011590127. membrane prote	1.828499	4.631991	1.776131	4.654958	1.029485	4.237982	4.217073	5.087733	4.258995	4.217073	5.087733	4.258995	13.284325	47.747523	15.594962	360	1237	565
gene307	NC_008261.1	355704	357683	+	protein_coding	CPF_RS01530	CPF_0300	.	WP_004457492. excinuclease AE	1.233343	4.933660	1.370886	4.854382	-1.111520	4.707807	4.784692	5.087267	4.632158	4.784692	5.087267	4.632158	20.961166	47.722785	20.482513	878	1911	1147
gene308	NC_008261.1	357949	358800	+	protein_coding	CPF_RS01535	CPF_0301	.	WP_003465642. DegV family prot	2.022597	7.688387	2.319588	7.582076	-1.146836	7.097536	7.197054	8.213263	6.999395	7.197054	8.213263	6.999395	121.781421	348.790910	117.195278	2195	6010	2824
gene309	NC_008261.1	359036	359761	+	protein_coding	CPF_RS01540	CPF_0302	.	WP_011590128. ABC transporte	-2.931653	5.887069	-3.514772	6.000716	1.198904	6.843438	6.713831	5.162117	6.975548	6.713831	5.162117	6.975548	83.145813	50.739938	115.618973	1277	745	2374
gene310	NC_008261.1	359754	361352	+	protein_coding	CPF_RS01545	CPF_0303	.	WP_011590129. ABC transporte	-2.053010	4.273297	-2.906493	4.489967	1.415723	5.068123	4.823553	3.785813	5.325092	4.823553	3.785813	5.325092	21.639575	19.326884	34.318498	732	625	1552
gene311	NC_008261.1	361573	362577	+	protein_coding	CPF_RS01550	CPF_0304	.	WP_011590130. alpha/beta hydr	-2.823854	4.769008	-4.004507	4.979849	1.418100	5.822804	5.576274	4.078609	6.080233	5.576274	4.078609	6.080233	36.922381	24.403150	58.437091	785	496	1661
gene312	NC_008261.1	362736	364301	+	protein_coding	CPF_RS01555	CPF_0305	.	WP_011590131. DNA mismatch r	-1.281477	4.903328	-1.637956	5.071158	1.278178	5.259560	5.085494	4.727687	5.439583	5.085494	4.727687	5.439583	25.808363	37.352809	37.006006	855	1183	1639
gene313	NC_008261.1	364316	365002	+	protein_coding	CPF_RS01560	CPF_0306	.	WP_003458079. FadR family tran	-1.425241	5.860452	-1.700747	5.981250	1.193305	6.247808	6.121627	5.610421	6.376589	6.121627	5.610421	6.376589	53.806671	70.534180	72.722774	782	980	1413
gene314	NC_008261.1	365240	366763	+	protein_coding	CPF_RS01565	CPF_0307	.	WP_003479050. L-lactate perme	-3.404729	4.038180	-3.067321	3.977132	-1.110001	4.941673	5.017527	3.249987	4.866966	5.017527	3.249987	4.866966	24.689609	12.621033	24.198272	796	389	1043
gene315	NC_008261.1	366858	367646	+	protein_coding	CPF_RS01570	CPF_0308	.	WP_011590133. electron transfe	-7.028771	2.964334	-7.879913	3.016024	1.121094	4.769521	4.687780	1.874507	4.852687	4.687780	1.874507	4.852687	19.591011	3.885483	23.975175	327	62	535
gene316	NC_008261.1	367659	368852	+	protein_coding	CPF_RS01575	CPF_0309	.	WP_011590134. electron transfe	-7.061347	3.197295	-7.524903	3.227057	1.065647	4.950007	4.904355	2.084411	4.996084	4.904355	2.084411	4.996084	22.645286	4.638141	27.273465	572	112	921
gene317	NC_008261.1	368854	370254	+	protein_coding	CPF_RS01580	CPF_0310	.	WP_011590135. FAD-binding pro	-7.369222	4.083884	-6.726169	4.037008	-1.095605	5.705088	5.771332	2.889819	5.639604	5.771332	2.889819	5.639604	41.669178	9.352722	42.348578	1235	265	1678
gene318	NC_008261.1	370338	370736	-	protein_coding	CPF_RS01585	CPF_0311	.	WP_003457995. TIGR03987 fami	-3.025787	6.884446	-2.739952	6.820391	-1.104321	7.657357	7.729271	6.131961	7.586111	7.729271	6.131961	7.586111	172.849650	99.511422	183.346494	1459	803	2069
gene319	NC_008261.1	371104	371613	+	protein_coding	CPF_RS01590	CPF_0312	.	WP_011590136. signal peptidase	-1.672136	6.104257	-1.534870	6.045829	-1.089432	6.424272	6.486357	5.744665	6.362781	6.486357	5.744665	6.362781	70.441651	77.756101	72.171473	760	802	1041
gene320	NC_008261.1	371637	372149	+	protein_coding	CPF_RS01595	CPF_0313	.	WP_011590137. hypothetical pro	-1.565851	6.547052	-1.367230	6.453249	-1.145272	6.779960	6.878511	6.231564	6.682820	6.878511	6.231564	6.682820	94.355821	104.964102	92.012942	1024	1089	1335
gene321	NC_008261.1	372276	372656	-	protein_coding	CPF_RS01600	CPF_0314	.	WP_011590138. DUF2809 domai	1.323402	4.677198	1.499002	4.582393	-1.132689	4.388642	4.479438	4.883688	4.299686	4.479438	4.883688	4.299686	16.625164	41.918702	16.054846	134	323	173
gene322	NC_008261.1	372975	373649	+	protein_coding	CPF_RS01605	CPF_0315	.	WP_003457977. DUF975 domain	1.229150	3.268344	1.136024	3.342281	1.104116	3.193547	3.122900	3.420561	3.265792	3.122900	3.420561	3.265792	5.882496	14.504130	7.542994	84	198	144
gene323	NC_008261.1	373842	374465	-	protein_coding	CPF_RS01610	CPF_0316	.	WP_003458062. DedA family prot	-1.608230	2.525907	-4.630061	3.121869	2.878978	3.574079	2.891791	2.206316	4.417347	2.891791	2.206316	4.417347	4.923964	5.229855	17.565565	65	66	310
gene324	NC_008261.1	374562	377219	-	protein_coding	CPF_RS01615	CPF_0317	.	WP_003458074. cation-transloca	-4.517922	4.451021	-12.150268	4.979829	2.689348	6.343469	5.669855	3.494196	7.097112	5.669855	3.494196	7.097112	38.911543	15.682052	125.734295	2188	843	9452
gene325	NC_008261.1	377756	378556	+	protein_coding	CPF_RS01620	CPF_0318	.	WP_003458097. TIGR00266 fami	-1.006296	7.730506	1.388359	7.485548	-1.397100	7.489934	7.735035	7.725980	7.252599	7.735035	7.725980	7.252599	173.500577	262.908971	139.665446	2940	4259	3164
gene326	NC_008261.1	379116	379688	+	protein_coding	CPF_RS01625	CPF_0319	.	WP_003458080. Xanthine phosph	43.809376	5.710798	57.844396	5.383565	-1.320366	3.395342	3.601723	9.054891	3.200786	3.601723	9.054891	3.200786	8.414565	622.949298	7.219652	102	7219	117
gene327	NC_008261.1	380174	381106	+	protein_coding	CPF_RS01630	CPF_0320	.	WP_003458020. EamA family trar	8.278188	2.730195	13.163460	2.083568	-1.590138	1.222890	1.602409	4.651724	0.933257	1.602409	4.651724	0.933257	1.519937	35.083800	0.795834	30	662	21
gene328	NC_008261.1	381297	382679	+	protein_coding	CPF_RS01635	CPF_0321	.	WP_011590140. amino acid perm	-1.171351	4.686718	-1.485063	4.850901	1.267821	4.970422	4.802193	4.574020	5.144544	4.802193	4.574020	5.144544	21.157024	33.321459	30.270147	619	932	1184
gene329	NC_008261.1	383008	384312	-	protein_coding	CPF_RS01640	CPF_0322	.	WP_011590141. TIGR00341 fami	2.446145	1.770890	2.387909	1.795550	1.024388	1.256789	1.239528	2.530038	1.274290	1.239528	2.530038	1.274290	1.014223	6.820124	1.246328	28	180	46
gene330	NC_008261.1	384584	385969	+	protein_coding	CPF_RS01645	CPF_0323	.	WP_011590142. TldD/PmbA fami	1.018487	6.244764	-1.082668	6.315028	1.102684	6.301679	6.231564	6.257992	6.372583	6.231564	6.257992	6.372583	59.582097	106.704679	72.679885	1747	2991	2849
gene331	NC_008261.1	385985	387334	+	protein_coding	CPF_RS01650	CPF_0324	.	WP_003467066. TldD/PmbA fami	-1.154173	5.686991	-1.298022	5.769592	1.124633	5.875478	5.791362	5.584502	5.960817	5.791362	5.584502	5.960817	41.877767	69.041126	53.560492	1196	1885	2045
gene332	NC_008261.1	387643	389229	+	protein_coding	CPF_RS01655	CPF_0325	.	WP_011590143. aspartate 4-dec	-1.684854	7.467299	-1.904725	7.550963	1.130498	7.941073	7.853087	7.100463	8.030046	7.853087	7.100463	8.030046	192.803444	177.469340	250.222590	6473	5696	11231
gene333	NC_008261.1	389547	390023	+	protein_coding	CPF_RS01660	CPF_0326	.	WP_011590144. methylated-DNA	-1.241624	3.154955	-1.432948	3.251857	1.154091	3.416745	3.314930	3.002701	3.521687	3.314930	3.002701	3.521687	6.837807	10.262356	9.043290	69	99	122
gene334	NC_008261.1	390072	392246	+	protein_coding	CPF_RS01665	CPF_0327	.	WP_011590145. DNA helicase Re	-1.053732	4.058468	-1.188351	4.143501	1.127755	4.182225	4.096398	4.020890	4.269851	4.096398	4.020890	4.269851	12.214148	23.438493	15.833784	562	1031	974
gene335	NC_008261.1	392477	393274	+	protein_coding	CPF_RS01670	CPF_0329	.	WP_011590146. amidinotransfera	-1.069087	0.597687	-1.306471	0.718859	1.222044	0.779151	0.647816	0.551437	0.937112	0.647816	0.551437	0.937112	0.414650	0.681585	0.797544	7	11	18
gene336	NC_008261.1	393400	394857	+	protein_coding	CPF_RS01675	CPF_0330	.	WP_041702578. MATE family effl	1.146380	1.623667	1.260125	1.549463	-1.099221	1.458275	1.528112	1.725197	1.391630	1.528112	1.725197	1.391630	1.426531	3.391351	1.406551	44	100	58
gene337	NC_008261.1	395023	395772	+	protein_coding	CPF_RS01680	CPF_0331	.	WP_003457974. MerR family tran	-1.087723	1.397379	-1.276944	1.504084	1.173960	1.570787	1.459350	1.338039	1.690734	1.459350	1.338039	1.690734	1.323562	2.175620	1.932892	21	33	41
gene338	NC_008261.1	395917	396303	+	protein_coding	CPF_RS01685	CPF_0332	.	WP_003458031. DUF2089 domai	-1.517640	4.583151	-1.880670	4.725815	1.239206	5.046272	4.893934	4.292104	5.203351	4.893934	4.292104	5.203351	22.596796	28.364314	31.520503	185	222	345
gene339	NC_008261.1	396319	396723	+	protein_coding	CPF_RS01690	CPF_0333	.	WP_003458015. hypothetical pro	-1.770520	4.295881	-2.127347	4.414585	1.201538	4.858323	4.727687	3.903514	4.992569	4.727687	3.903514	4.992569	20.075184	21.243423	27.238588	172	174	312
gene340	NC_008261.1	396713	396997	+	protein_coding	CPF_RS01695	CPF_0334	.	WP_003479024. hypothetical pro	1.093479	0.812625	1.278877	0.679413	-1.169549	0.627652	0.750715	0.879640	0.524762	0.750715	0.879640	0.524762	0.497580	1.214461	0.372187	3	7	3
gene341	NC_008261.1	397006	397377	+	protein_coding	CPF_RS01700	CPF_0335	.	WP_003458048. phage holin fami	-1.136059	0.325326	-1.225333	0.364270	1.078583	0.481595	0.430108	0.246071	0.539245	0.430108	0.246071	0.539245	0.254140	0.265838	0.380191	2	2	4
gene342	NC_008261.1	397452	398123	-	protein_coding	CPF_RS01705	CPF_0336	.	WP_003458068. transcriptional r	-1.045044	2.638805	1.120052	2.524107	-1.170504	2.554691	2.670779	2.607215	2.443649	2.670779	2.607215	2.443649	4.009514	7.358021	3.998797	57	100	76
gene343	NC_008261.1	399068	401887	+	protein_coding	CPF_RS01710	CPF_0337	.	WP_011590149. excinuclease AE	1.000000	5.868353	-1.470965	6.140427	1.470965	6.140427	5.868353	5.868353	6.425115	5.868353	5.868353	6.425115	43.816994	85.688692	75.254400	2614	4887	6002
gene344	NC_008261.1	402221	402655	+	protein_coding	CPF_RS01715	CPF_0338	.	WP_003473867. hypothetical pro	1.223687	6.986818	1.313020	6.934726	-1.073003	6.791701	6.842718	7.133953	6.741064	6.842718	7.133953	6.741064	92.040774	183.575006	95.913064	847	1615	1180
gene345	NC_008261.1	402669	403898	+	protein_coding	CPF_RS01720	CPF_0339	.	WP_003457997. FtsW/RodA/Sp	1.453948	6.059611	1.235749	6.181032	1.176572	5.911767	5.795635	6.335611	6.030225	5.795635	6.335611	6.030225	42.197171	111.675373	56.342482	1098	2778	1960
gene346	NC_008261.1	403899	405362	+	protein_coding	CPF_RS01725	CPF_0340	.	WP_003452191. penicillin-binding	1.629839	7.950429	1.470314	8.027722	1.108497	7.679812	7.605869	8.310599	7.754474	7.605869	8.310599	7.754474	157.308544	363.312527	212.484813	4872	10757	8798
gene347	NC_008261.1	405519	407381	+	protein_coding	CPF_RS01730	CPF_0341	.	WP_003458095. UvrABC system	-1.198071	5.387166	-1.514160	5.549582	1.263832	5.685493	5.519100	5.258386	5.856904	5.519100	5.258386	5.856904	35.116348	54.594855	49.724846	1384	2057	2620
gene348	NC_008261.1	407530	408444	+	protein_coding	CPF_RS01735	CPF_0342	.	WP_003457971. UDP-N-acetyle	1.678427	6.405020	1.340405	6.574729	1.252179	6.202448	6.042349	6.789459	6.366790	6.042349	6.789459	6.366790	50.576376	148.661933	72.493114	979	2751	1876
gene349	NC_008261.1	408684	409568	+	protein_coding	CPF_RS01740	CPF_0343	.	WP_003457961. RNase adaptor p	-1.555056	6.376623	-1.690733	6.433769	1.087248	6.763126	6.703055	6.066088	6.823736	6.703055	6.066088	6.823736	82.255237	96.321729	102.437687	1540	1724	2564
gene351	NC_008261.1	410913	411863	+	protein_coding	CPF_RS01750	CPF_0345	.	WP_003457993. sporulation prot	-1.199280	6.125505	-1.315237	6.190330	1.096689	6.324219	6.257992	5.995824	6.391147	6.257992	5.995824	6.391147	60.889398	92.184626	73.169416	1225	1773	1968
gene352	NC_008261.1	412037	412591	+	protein_coding	CPF_RS01755	CPF_0346	.	WP_011590150. hypothetical pro	1.284275	1.272007	1.029326	1.444223	1.247685	1.253776	1.104269	1.465224	1.423523	1.104269	1.465224	1.423523	0.851713	2.583660	1.465277	10	29	23
gene353	NC_008261.1	412991	414241	+	protein_coding	CPF_RS01760		.	WP_025648808. hypothetical pro	-1.949918	6.594988	-2.132691	6.654799	1.093734	7.158602	7.094263	6.130850	7.223525	7.094263	6.130850	7.223525	112.828445	99.484675	136.230623	2986	2517	4820
gene354	NC_008261.1	414446	415225	+	protein_coding	CPF_RS01765	CPF_0348	.	WP_004457554. hypothetical pro	1.118708	3.788903	-1.151578	3.971185	1.288279	3.887281	3.708851	3.870684	4.074296	3.708851	3.870684	4.074296	9.150998	20.539066	13.463155	151	324	297
gene356	NC_008261.1	418374	419333	+	protein_coding	CPF_RS01775	CPF_0350	.	WP_003455617. ATP-dependent	2.937085	9.244415	3.543032	9.096073	-1.206309	8.363441	8.499835	10.054220	8.229235	8.499835	10.054220	8.229235	306.122846	1149.668666	297.373679	6217	22321	8074
gene357	NC_008261.1	419427	420830	+	protein_coding	CPF_RS01780	CPF_0351	.	WP_011590151. pyruvate kinase	1.726898	10.349028	1.815168	10.311604	-1.051115	9.926412	9.962437	10.750620	9.890517	9.962437	10.750620	9.890517	864.159902	2064.014120	1001.249785	25667	58607	39758
gene358	NC_008261.1	421410	421766	+	protein_coding	CPF_RS01785	CPF_0352	.	WP_003479007. DUF1540 domai	1.768300	3.641743	1.712853	3.667374	1.032371	3.276601	3.253701	4.076064	3.299662	3.253701	4.076064	3.299662	6.488047	24.376690	7.725230	49	176	78
gene359	NC_008261.1	422075	422872	+	protein_coding	CPF_RS01790	CPF_0353	.	WP_011590152. NAD(P)-depend	1.638545	4.344370	1.789971	4.274608	-1.092415	3.938465	4.002741	4.715156	3.875220	4.002741	4.715156	3.875220	11.491718	36.929519	11.785927	194	596	266
gene360	NC_008261.1	423044	424579	+	protein_coding	CPF_RS01795	CPF_0354	.	WP_011590153. cardiolipin synth	1.695735	4.938658	1.996556	4.809737	-1.177399	4.453015	4.572374	5.334285	4.336771	4.572374	5.334285	4.336771	18.003244	57.364969	16.550941	585	1782	719
gene362	NC_008261.1	425211	426773	+	protein_coding	CPF_RS01800	CPF_0356	.	WP_011590154. PLP-dependent	1.569267	2.420836	1.586375	2.411878	-1.010902	2.109679	2.117515	2.767607	2.101872	2.117515	2.767607	2.101872	2.449696	8.383342	3.053935	81	265	135
gene363	NC_008261.1	427075	428442	+	protein_coding	CPF_RS01805	CPF_0357	.	WP_011590155. 23S rRNA (urac	2.007735	4.863564	2.056180	4.844459	-1.024129	4.369467	4.386699	5.392268	4.352302	4.386699	5.392268	4.352302	15.307481	59.927852	16.593345	443	1658	642
gene364	NC_008261.1	429345	431828	+	protein_coding	CPF_RS01810		.	WP_011590157. hypothetical pro	1.558965	3.076039	1.550671	3.080305	1.005349	2.776220	2.772375	3.412964	2.780071	2.772375	3.412964	2.780071	4.395887	14.471485	5.295127	231	727	372
gene366	NC_008261.1	432472	434523	+	protein_coding	CPF_RS01820	CPF_0361	.	WP_011590159. site-specific DN	2.975620	4.178731	2.791415	4.233950	1.065990	3.511319	3.465525	5.038716	3.557719	3.465525	5.038716	3.557719	7.578873	45.759144	9.373603	329	1899	544
gene367	NC_008261.1	434536	437700	+	protein_coding	CPF_RS01825	CPF_0362	.	WP_011590160. DEAD/DEAH bo	4.081991	3.870364	4.652082	3.746161	-1.139660	2.890674	2.986514	5.015787	2.797910	2.986514	5.015787	2.797910	5.242272	45.040293	5.384661	351	2883	482
gene368	NC_008261.1	438159	438710	+	protein_coding	CPF_RS01830	CPF_0363	.	WP_004457634. hypothetical pro	-1.180761	4.858660	-1.199926	4.869978	1.016231	4.991598	4.979997	4.740280	5.003225	4.979997	4.740280	5.003225	23.806296	37.890608	27.351038	278	423	427
gene369	NC_008261.1	438829	439407	+	protein_coding	CPF_RS01835	CPF_0364	.	WP_003455253. TetR family tran	-1.358671	4.341287	-1.379808	4.351857	1.015557	4.579134	4.568012	4.125816	4.590283	4.568012	4.125816	4.590283	17.960988	24.936447	19.907836	220	292	326
gene370	NC_008261.1	439756	441549	+	protein_coding	CPF_RS01840	CPF_0365	.	WP_003461039. oleate hydratase	-3.182092	6.105445	-2.916436	6.050326	-1.091089	6.934095	6.997265	5.327289	6.871496	6.997265	5.327289	6.871496	104.473672	57.163208	105.284990	3965	2074	5342
gene371	NC_008261.1	441948	443681	+	protein_coding	CPF_RS01845	CPF_0366	.	WP_011590161. ABC transporte	-1.826977	4.280046	-1.690197	4.229021	-1.080925	4.680326	4.736796	3.867338	4.624529	4.736796	3.867338	4.624529	20.145658	20.502654	20.288923	739	719	995
gene372	NC_008261.1	443674	445455	+	protein_coding	CPF_RS01850	CPF_0367	.	WP_011590162. ABC transporte	-1.566254	4.842355	-1.383133	4.757721	-1.132396	5.086339	5.176818	4.529501	4.997440	5.176818	4.529501	4.997440	27.401777	32.297995	27.282239	1033	1164	1375
gene373	NC_008261.1	445536	445934	-	protein_coding	CPF_RS01855	CPF_0368	.	WP_011590163. rubrerythrin	1.082961	6.456001	3.993843	5.423642	-3.687890	5.375560	6.398767	6.513748	4.515971	6.398767	6.513748	4.515971	67.291707	126.279126	18.875207	568	1019	213
gene374	NC_008261.1	446200	447228	+	protein_coding	CPF_RS01860	CPF_0369	.	WP_011590164. autolytic lysozyr	1.130854	0.386634	-1.179195	0.592474	1.333497	0.471935	0.307973	0.485385	0.723188	0.307973	0.485385	0.723188	0.183751	0.576629	0.549781	4	12	16
gene375	NC_008261.1	447486	447884	+	protein_coding	CPF_RS01865	CPF_0370	.	WP_011590165. hypothetical pro	-1.004425	1.286827	1.010675	1.275964	-1.015148	1.279126	1.290016	1.283646	1.268327	1.290016	1.283646	1.268327	1.066242	2.106718	1.240624	9	17	14
gene376	NC_008261.1	447929	448999	-	protein_coding	CPF_RS01870	CPF_0371	.	WP_011590166. oxidoreductase	1.918259	7.261450	2.713260	6.989535	-1.414439	6.551852	6.806739	7.746536	6.306509	6.806739	7.746536	6.306509	89.155473	264.911831	68.866791	2020	5738	2086
gene377	NC_008261.1	449260	450180	-	protein_coding	CPF_RS01875	CPF_0372	.	WP_011590167. polysaccharide c	2.633654	4.330991	3.030953	4.210300	-1.150855	3.585644	3.688429	5.085494	3.485723	3.688429	5.085494	3.485723	9.033148	47.674222	8.753067	176	888	228
gene378	NC_008261.1	450585	451403	+	protein_coding	CPF_RS01880	CPF_0373	.	WP_003455277. NAD(P)-depend	-2.451880	5.394636	-1.873283	5.219527	-1.308868	5.882870	6.080233	4.786345	5.691914	6.080233	4.786345	5.691914	52.002832	39.122030	44.121677	901	648	1022
gene379	NC_008261.1	451426	452157	-	protein_coding	CPF_RS01885	CPF_0374	.	WP_003471236. GntR family tran	-7.148440	3.702477	-9.203991	3.825802	1.287552	5.563161	5.383832	2.546204	5.748463	5.383832	2.546204	5.748463	32.029983	6.890062	45.791226	496	102	948
gene380	NC_008261.1	452392	453177	+	protein_coding	CPF_RS01890	CPF_0375	.	WP_003461217. uridine phospho	-5.538115	9.062752	-5.346560	9.040558	-1.035828	10.355747	10.381170	7.911775	10.330386	10.381170	7.911775	10.330386	1138.991823	289.629670	1364.108629	18939	4604	30324
gene381	NC_008261.1	453344	454534	+	protein_coding	CPF_RS01895	CPF_0376	.	WP_003450068. phosphopentom	-10.005042	8.736231	-11.656864	8.827000	1.165099	10.663776	10.554119	7.231464	10.774571	10.554119	7.231464	10.774571	1304.986910	196.039913	1721.695392	32880	4722	57994
gene382	NC_008261.1	454962	456407	-	protein_coding	CPF_RS01900	CPF_0377	.	WP_003455623. arginine:agmatin	-9.846580	8.174616	-7.854396	8.040069	-1.253639	9.824835	9.989249	6.689627	9.663127	9.989249	6.689627	9.663127	876.718953	140.849004	814.842828	26819	4119	33324
gene383	NC_008261.1	456453	457415	-	protein_coding	CPF_RS01905	CPF_0378	.	WP_003450230. histidine decarb	-6.059581	9.026787	-4.359468	8.818603	-1.389982	10.179167	10.419471	7.820253	9.944405	10.419471	7.820253	9.944405	1170.903316	274.596750	1051.260660	23854	5348	28632
gene384	NC_008261.1	457883	458716	+	protein_coding	CPF_RS01910	CPF_0379	.	WP_011590168. phosphoesteras	1.506246	3.070595	1.766144	2.941478	-1.172547	2.672011	2.789300	3.380257	2.559654	2.789300	3.380257	2.559654	4.477619	14.110460	4.366728	79	238	103
gene385	NC_008261.1	459461	460324	+	protein_coding	CPF_RS01915	CPF_0381	.	WP_011590169. NAD(P)-depend	-1.294192	6.718838	-1.365559	6.756396	1.055145	6.946051	6.907438	6.535387	6.984879	6.907438	6.535387	6.984879	96.837960	129.509341	116.958960	1770	2263	2858
gene386	NC_008261.1	460631	461536	+	protein_coding	CPF_RS01920	CPF_0382	.	WP_004457430. quinolinate synt	2.787601	6.247276	2.142714	6.457329	1.300967	5.738036	5.551381	7.030405	5.930966	5.551381	7.030405	5.930966	36.313420	170.058965	52.568358	696	3116	1347
gene387	NC_008261.1	461541	462860	+	protein_coding	CPF_RS01925	CPF_0383	.	WP_011590170. L-aspartate oxic	2.175239	6.706659	1.573597	6.955918	1.382335	6.398753	6.169460	7.290634	6.636568	6.169460	7.290634	6.636568	56.294338	203.252614	88.608745	1572	5426	3308
gene388	NC_008261.1	462817	463656	+	protein_coding	CPF_RS01930	CPF_0384	.	WP_011590171. nicotinate-nucl	1.634348	8.103712	1.416790	8.210655	1.153557	7.859467	7.757098	8.465813	7.963187	7.757098	8.465813	7.963187	177.037611	419.348318	242.158714	3146	7124	5753
gene389	NC_008261.1	463973	465331	-	protein_coding	CPF_RS01935	CPF_0385	.	WP_003455285. purine permease	53.757142	4.661780	61.292952	4.489063	-1.140182	2.505992	2.602411	8.350795	2.413146	2.602411	8.350795	2.413146	3.860910	379.449069	3.928642	111	10429	151
gene390	NC_008261.1	466007	466825	+	protein_coding	CPF_RS01940	CPF_0386	.	WP_011590172. purine-nucleosi	-6.944841	6.973081	-2.163315	6.245731	-3.210277	7.622162	8.509805	5.713863	6.827107	8.509805	5.713863	6.827107	307.688234	75.768747	102.662766	5331	1255	2378
gene391	NC_008261.1	466915	467592	+	protein_coding	CPF_RS01945	CPF_0387	.	WP_011590173. nicotinamide mo	-2.747965	4.973210	-1.568677	4.610550	-1.751773	5.335853	5.755564	4.297201	4.946749	5.755564	4.297201	4.946749	41.343868	28.369402	26.022910	593	389	499
gene392	NC_008261.1	467626	468267	+	protein_coding	CPF_RS01950	CPF_0388	.	WP_003455357. Crp/Fnr family t	-1.353526	4.174130	-1.760084	4.350223	1.300369	4.583745	4.398199	3.961476	4.777120	4.398199	3.961476	4.777120	15.462168	22.258357	22.635589	210	289	411
gene394	NC_008261.1	468822	469433	+	protein_coding	CPF_RS01960	CPF_0390	.	WP_011590175. hypothetical pro	-1.162125	4.434641	-1.194019	4.453659	1.027445	4.563836	4.544348	4.327583	4.583408	4.544348	4.327583	4.583408	17.687651	28.924235	19.816535	229	358	343
gene395	NC_008261.1	469640	470176	+	protein_coding	CPF_RS01965	CPF_0391	.	WP_011590176. hypothetical pro	2.415345	2.247680	2.033619	2.406175	1.187708	1.819709	1.699845	2.972075	1.948025	1.699845	2.972075	1.948025	1.672497	10.036505	2.567884	19	109	39
gene396	NC_008261.1	470911	472248	+	protein_coding	CPF_RS01970	CPF_0392	.	WP_003478897. branched-chain	1.448895	4.077144	-1.003491	4.355899	1.453954	4.079498	3.818432	4.353385	4.358413	3.818432	4.353385	4.358413	10.033405	29.490155	16.727561	284	798	633
gene397	NC_008261.1	472504	473376	+	protein_coding	CPF_RS01975	CPF_0393	.	WP_003455426. YitT family prote	1.127475	8.971634	-1.013923	9.068567	1.143173	8.981507	8.885503	9.058599	9.078547	8.885503	9.058599	9.078547	402.201568	625.238583	538.993543	7428	11039	13308
gene398	NC_008261.1	473690	476701	+	protein_coding	CPF_RS01980	CPF_0394	.	WP_003455341. silent informatio	1.818772	5.706963	-2.636667	6.818318	4.795495	6.322270	5.291769	6.154733	7.553449	5.291769	6.154733	7.553449	29.394692	101.107999	180.909289	1873	6159	15411
gene399	NC_008261.1	476808	477638	+	protein_coding	CPF_RS01985	CPF_0395	.	WP_003455501. 5-dehydro-4-de	9.623681	8.579677	-1.141736	10.462230	10.987706	8.658446	7.100463	10.367052	10.558281	7.100463	10.367052	10.558281	113.254729	1461.123351	1544.126676	1991	24556	36291
gene400	NC_008261.1	477654	478430	+	protein_coding	CPF_RS01990	CPF_0396	.	WP_003455308. 2-deoxy-D-gluc	7.831498	10.434445	-1.201371	12.155800	9.408534	10.548664	9.054891	12.024180	12.288861	9.054891	12.024180	12.288861	456.700519	4995.626759	4789.546042	7507	78502	105252
gene401	NC_008261.1	478427	479068	+	protein_coding	CPF_RS01995	CPF_0397	.	WP_011590177. bifunctional 2-k	9.629511	8.981517	-1.242959	10.918389	11.969091	9.111502	7.495164	10.762626	11.076405	7.495164	10.762626	11.076405	145.712522	2084.737701	2151.262187	1979	27068	39061
gene402	NC_008261.1	479072	480091	+	protein_coding	CPF_RS02000	CPF_0398	.	WP_003455275. sugar kinase	9.567366	8.466265	-1.241431	10.405464	11.877221	8.594145	6.992510	10.250632	10.562636	6.992510	10.250632	10.562636	103.623376	1345.994943	1549.398808	2236	27766	44697
gene403	NC_008261.1	480256	481086	+	protein_coding	CPF_RS02005	CPF_0399	.	WP_003455313. 5-dehydro-4-de	2.282894	9.997964	-3.009233	11.378090	6.869759	10.720624	9.420247	10.611111	12.200506	9.420247	10.611111	12.200506	592.496863	1784.396000	4383.514003	10416	29989	103024
gene404	NC_008261.1	481103	482293	+	protein_coding	CPF_RS02010	CPF_0400	.	WP_003455311. glucuronyl hydro	2.157468	10.158737	-3.873408	11.664512	8.356755	11.045001	9.619199	10.728538	12.682142	9.619199	10.728538	12.682142	705.716918	1998.137439	6268.094600	17781	48129	211136
gene405	NC_008261.1	482310	482798	+	protein_coding	CPF_RS02015	CPF_0401	.	WP_003455664. PTS mannose/f	2.764080	10.349548	-3.138337	11.905350	8.674614	11.091556	9.642101	11.108901	12.758901	9.642101	11.108901	12.758901	716.590835	2678.774572	6283.056176	7413	26492	86895
gene406	NC_008261.1	482846	483637	+	protein_coding	CPF_RS02020	CPF_0402	.	WP_003455334. PTS mannose/f	2.432757	10.962016	-3.271975	12.447801	7.959920	11.740867	10.339462	11.622054	13.332216	10.339462	11.622054	13.332216	1095.925191	3720.616632	9330.927608	18362	59595	209009
gene407	NC_008261.1	483627	484436	+	protein_coding	CPF_RS02025	CPF_0403	.	WP_011590178. PTS N-acetylglu	2.412980	10.640496	-3.329410	12.131506	8.033800	11.428672	10.024043	11.294859	13.030126	10.024043	11.294859	13.030126	881.674064	3132.672424	7395.625758	15108	51318	169424
gene408	NC_008261.1	484506	484919	+	protein_coding	CPF_RS02030	CPF_0404	.	WP_003455482. PTS mannose tr	6.589527	9.223535	-1.406748	10.926771	9.269803	9.433684	7.963187	10.683362	11.175726	7.963187	10.683362	11.175726	214.884646	1927.792902	2338.652525	1882	16141	27383
gene409	NC_008261.1	484922	485200	+	protein_coding	CPF_RS02035	CPF_0405	.	WP_003455254. preprotein trans	8.513176	8.795696	-1.161433	10.582582	9.887483	8.885878	7.385483	10.475180	10.691086	7.385483	10.475180	10.691086	135.371950	1594.674561	1683.106463	799	8998	13281
gene410	NC_008261.1	485340	487358	+	protein_coding	CPF_RS02040	CPF_0406	.	WP_003455377. heparinase	10.019864	5.311853	-1.361237	7.447442	13.639405	5.472891	3.903514	7.228305	7.673223	3.903514	7.228305	7.673223	10.629324	195.652942	196.945823	454	7989	11246
gene411	NC_008261.1	487392	488405	+	protein_coding	CPF_RS02045	CPF_0407	.	WP_003455628. LacI family DNA-	4.464136	7.341751	1.531099	8.186786	2.915641	7.071355	6.341454	8.499835	7.885267	6.341454	8.499835	7.885267	64.751584	429.603919	231.394716	1389	8810	6636
gene412	NC_008261.1	488700	490046	+	protein_coding	CPF_RS02050	CPF_0408	.	WP_011590179. FAD-binding oxi	1.564021	5.661627	1.573051	5.657230	-1.005773	5.344028	5.348182	5.993443	5.339878	5.348182	5.993443	5.339878	31.022070	91.880539	34.544054	884	2503	1316
gene413	NC_008261.1	490346	490699	+	protein_coding	CPF_RS02055	CPF_0409	.	WP_003455446. hypothetical pro	-4.198459	4.418238	-1.921827	3.946101	-2.184619	4.977251	5.572760	3.502900	4.445378	5.572760	3.502900	4.445378	36.721088	15.783578	17.778772	275	113	178
gene414	NC_008261.1	490894	491718	+	protein_coding	CPF_RS02060	CPF_0410	.	WP_011590180. Cof-type HAD-I	-5.679231	4.313559	-2.948900	3.942624	-1.925881	5.250665	5.744665	3.238969	4.799146	5.744665	3.238969	4.799146	40.910084	12.526295	23.228991	714	209	542
gene415	NC_008261.1	491866	493287	+	protein_coding	CPF_RS02065	CPF_0411	.	WP_011590181. FAD/NAD(P)-bi	-5.374427	4.737856	-2.886179	4.375915	-1.862125	5.637453	6.103738	3.677627	5.206788	6.103738	3.677627	5.206788	52.954429	17.803306	31.652923	1593	512	1273
gene416	NC_008261.1	493292	494182	+	protein_coding	CPF_RS02070	CPF_0412	.	WP_003461122. NAD(P)/FAD-de	-4.098210	4.500310	-2.531807	4.213539	-1.618689	5.272552	5.631399	3.596405	4.936572	5.631399	3.596405	4.936572	38.091919	16.925925	25.754434	718	305	649
gene417	NC_008261.1	494423	494800	+	protein_coding	CPF_RS02075	CPF_0413	.	WP_011590182. gamma-glutamyl	-1.043585	3.020817	1.365590	2.756289	-1.425109	2.784510	3.051747	2.990200	2.540675	3.051747	2.990200	2.540675	5.502334	10.203122	4.302799	44	78	46
gene418	NC_008261.1	495016	495588	+	protein_coding	CPF_RS02080	CPF_0414	.	WP_011009793. carbonic anhydr	-2.392316	7.827481	-1.419858	7.472121	-1.684898	8.096861	8.481933	7.223525	7.729271	8.481933	7.223525	7.729271	298.304571	194.159291	207.086765	3616	2250	3356
gene419	NC_008261.1	495732	496370	+	protein_coding	CPF_RS02085	CPF_0415	.	WP_011590183. hypothetical pro	-2.092556	4.034188	-2.036756	4.017060	-1.027397	4.582292	4.601831	3.535664	4.562837	4.601831	3.535664	4.562837	18.271837	16.095066	19.532546	247	208	353
gene420	NC_008261.1	496395	496784	+	protein_coding	CPF_RS02090	CPF_0416	.	WP_011590184. zinc ribbon doma	-2.181306	5.647904	-1.639388	5.458203	-1.330561	6.028915	6.238452	5.113259	5.826417	6.238452	5.113259	5.826417	59.875403	48.431625	48.684946	494	382	537
gene421	NC_008261.1	497117	498988	+	protein_coding	CPF_RS02095	CPF_0417	.	WP_003455303. molecular chape	-4.448293	6.310536	-2.570610	5.967409	-1.730443	7.071718	7.478344	5.325092	6.687203	7.478344	5.325092	6.687203	143.047469	57.158614	92.757515	5665	2164	4911
gene422	NC_008261.1	499216	500817	+	protein_coding	CPF_RS02100	CPF_0418	.	WP_011590185. zinc ribbon doma	-1.023021	0.281834	-1.015108	0.276499	-1.007795	0.293075	0.298729	0.265894	0.287528	0.298729	0.265894	0.287528	0.177041	0.277786	0.198639	6	9	9
gene424	NC_008261.1	501760	502752	+	protein_coding	CPF_RS02110	CPF_0420	.	WP_011590187. LacI family trans	1.745381	4.776900	1.804554	4.750670	-1.033903	4.367879	4.391995	5.195537	4.343895	4.391995	5.195537	4.343895	15.423462	52.035211	16.592877	324	1045	466
gene425	NC_008261.1	503188	504852	+	protein_coding	CPF_RS02115	CPF_0421	.	WP_011590188. alpha-glucosidas	-5.490662	6.161623	-3.309980	5.854493	-1.658821	7.136976	7.511387	5.054406	6.781229	7.511387	5.054406	6.781229	147.772154	46.030717	99.277856	5205	1550	4675
gene426	NC_008261.1	505111	506766	+	protein_coding	CPF_RS02120	CPF_0422	.	WP_011009797. PTS trehalose t	-8.624691	6.223141	-5.799922	5.995448	-1.487036	7.676975	7.968528	4.860055	7.396089	7.968528	4.860055	7.396089	216.169159	41.234775	159.665153	7573	1381	7478
gene427	NC_008261.1	506777	507568	+	protein_coding	CPF_RS02125	CPF_0423	.	WP_011590189. endonuclease	-6.539694	5.612868	-5.124521	5.472615	-1.276157	6.950499	7.128627	4.419404	6.776821	7.128627	4.419404	6.776821	115.489339	30.404234	99.153578	1935	487	2221
gene428	NC_008261.1	508224	508961	+	protein_coding	CPF_RS02130	CPF_0424	.	WP_011590190. glycerophospho	-17.362648	7.175080	-12.328020	6.986495	-1.408389	9.273300	9.523613	5.405698	9.029567	9.523613	5.405698	9.029567	662.997750	60.500877	523.659292	10351	903	10930
gene429	NC_008261.1	509058	510344	+	protein_coding	CPF_RS02135	CPF_0425	.	WP_011009800. MFS transporte	-37.690103	6.183015	-31.092473	6.090356	-1.212194	9.192653	9.332512	4.096398	9.054891	9.332512	4.096398	9.054891	556.883119	24.588481	525.806209	15162	640	19139
gene430	NC_008261.1	510845	513457	+	protein_coding	CPF_RS02140	CPF_0426	.	WP_011590191. ATP-dependent	-3.576614	3.844360	-2.874784	3.717948	-1.244133	4.711840	4.872044	3.033450	4.556903	4.872044	3.033450	4.556903	22.269207	10.521209	19.417687	1231	556	1435
gene431	NC_008261.1	513634	514293	+	protein_coding	CPF_RS02145	CPF_0427	.	WP_003450220. tRNA (guanosine	1.015455	6.325514	1.381952	6.087265	-1.381952	6.076628	6.314460	6.336587	5.847753	6.314460	6.336587	5.847753	63.384847	111.702782	49.393750	885	1491	922
gene432	NC_008261.1	514812	516095	+	protein_coding	CPF_RS02150	CPF_0428	.	WP_011009803. metal-independ	-4.993031	3.316954	-7.820855	3.539233	1.566354	4.987096	4.673885	2.353969	5.321296	4.673885	2.353969	5.321296	19.438153	5.930427	34.228756	528	154	1243
gene433	NC_008261.1	516563	517141	+	protein_coding	CPF_RS02155	CPF_0429	.	WP_011009804. hypothetical pro	-1.084977	0.208930	-1.707215	0.383570	1.573503	0.506497	0.275888	0.158223	0.929867	0.275888	0.158223	0.929867	0.163282	0.170798	0.793871	2	2	13
gene434	NC_008261.1	517919	518446	+	protein_coding	CPF_RS02160	CPF_0431	.	WP_003450157. hypothetical pro	1.414879	3.368893	1.305084	3.431077	1.084129	3.185577	3.127842	3.628520	3.244378	3.127842	3.628520	3.244378	5.908757	17.324794	7.433170	66	185	111
gene435	NC_008261.1	518534	519076	-	protein_coding	CPF_RS02165	CPF_0432	.	WP_011009806. signal peptidase	1.063363	0.211449	1.118830	0.160036	-1.052162	0.129971	0.171726	0.260360	0.098369	0.171726	0.260360	0.098369	0.087054	0.273182	0.065116	1	3	1
gene436	NC_008261.1	519284	520126	+	protein_coding	CPF_RS02170	CPF_0433	.	WP_011590192. hypothetical pro	1.704917	1.476557	1.400089	1.650218	1.217721	1.275236	1.141036	1.910738	1.425219	1.141036	1.910738	1.425219	0.897178	3.988519	1.467998	16	68	35
gene437	NC_008261.1	520409	521248	+	protein_coding	CPF_RS02175	CPF_0434	.	WP_003455658. alpha/beta hydr	-1.027498	1.214843	1.160997	1.082408	-1.192922	1.099982	1.234568	1.195433	0.980068	1.234568	1.195433	0.980068	1.012930	1.883653	0.841852	18	32	20
gene438	NC_008261.1	521332	522627	+	protein_coding	CPF_RS02180	CPF_0435	.	WP_003455362. hypothetical pro	-1.485182	4.139406	-1.419396	4.108787	-1.046348	4.401745	4.434547	3.863908	4.369185	4.434547	3.863908	4.369185	16.194371	20.488000	16.914988	444	537	620
gene439	NC_008261.1	523004	523492	+	protein_coding	CPF_RS02185	CPF_0436	.	WP_003455413. N-acetyltransfe	-1.780424	7.186914	-1.787738	7.189705	1.004108	7.618017	7.615061	6.782840	7.620975	7.615061	6.782840	7.620975	159.403519	148.236582	189.080982	1649	1466	2615
gene440	NC_008261.1	523551	523886	-	protein_coding	CPF_RS02190	CPF_0437	.	WP_003450245. hypothetical pro	-4.871085	9.889201	-3.773032	9.723611	-1.291027	10.911242	11.097057	8.812814	10.728538	11.097057	8.812814	10.728538	1766.296254	536.694033	1702.645575	12555	3647	16180
gene441	NC_008261.1	523892	524308	-	protein_coding	CPF_RS02195	CPF_0438	.	WP_003449994. YtxH domain-co	-4.337121	10.648001	-4.117048	10.613931	-1.053454	11.721215	11.758839	9.642101	11.683711	11.758839	9.642101	11.683711	3026.530455	883.504547	3171.431946	26699	7451	37403
gene442	NC_008261.1	524854	526191	+	protein_coding	CPF_RS02200	CPF_0439	.	WP_003455347. HlyC/CorC famil	4.193244	7.136705	7.734263	6.606842	-1.844458	5.718569	6.177192	8.245259	5.293995	6.177192	8.245259	5.293995	56.738198	353.623177	33.983638	1606	9569	1286
gene443	NC_008261.1	526466	527518	+	protein_coding	CPF_RS02205	CPF_0440	.	WP_011590193. Fe(3+) ABC tran	2.625952	3.639362	3.112384	3.487939	-1.185240	2.883781	3.008975	4.401816	2.763796	3.008975	4.401816	2.763796	5.342010	30.146503	5.238190	119	642	156
gene444	NC_008261.1	527518	529110	+	protein_coding	CPF_RS02210	CPF_0441	.	WP_041702739. iron ABC transp	1.999297	1.195100	1.730402	1.342513	1.155394	0.893774	0.795634	1.795127	1.004019	0.795634	1.795127	1.004019	0.534125	3.600580	0.887829	18	116	40
gene445	NC_008261.1	529113	529793	+	protein_coding	CPF_RS02215	CPF_0442	.	WP_003471458. ABC transporte	1.426377	1.508875	1.758349	1.318096	-1.232738	1.113171	1.274290	1.786645	0.972424	1.274290	1.786645	0.972424	1.041191	3.557781	0.830726	15	49	16
gene446	NC_008261.1	530086	530775	+	protein_coding	CPF_RS02220	CPF_0443	.	WP_041702592. nitrite reductase	-1.125663	2.611458	1.497295	2.217250	-1.685449	2.290932	2.698241	2.527466	1.945108	2.698241	2.527466	1.945108	4.110440	6.807769	2.562158	60	95	50
gene447	NC_008261.1	530965	531138	+	protein_coding	CPF_RS02225	CPF_0444	.	WP_003449944. (2Fe-2S)-bindin	-1.225376	12.025621	3.088100	11.036612	-3.784083	11.171987	12.173127	11.879903	10.253183	12.173127	11.879903	10.253183	4242.079941	4465.476222	1290.965456	15615	15714	6353
gene448	NC_008261.1	531404	531775	+	protein_coding	CPF_RS02230	CPF_0445	.	WP_003455483. GntR family tran	-1.650578	2.759672	-1.599931	2.739873	-1.031656	3.122170	3.144732	2.421761	3.099771	3.144732	2.421761	3.099771	5.972292	6.247197	6.748394	47	47	71
gene449	NC_008261.1	531809	532672	+	protein_coding	CPF_RS02235	CPF_0446	.	WP_011590196. ABC transporte	-1.188017	1.547637	-1.609486	1.738061	1.354767	1.883225	1.676897	1.428341	2.114941	1.676897	1.428341	2.114941	1.641321	2.460849	3.110175	30	43	76
gene451	NC_008261.1	533302	533970	+	protein_coding	CPF_RS02245	CPF_0448	.	WP_003455536. membrane prote	1.117643	0.640345	-1.445764	0.955172	1.615847	0.842966	0.565122	0.725581	1.257413	0.565122	0.725581	1.257413	0.353289	0.960832	1.215589	5	13	23
gene452	NC_008261.1	534086	534466	+	protein_coding	CPF_RS02250	CPF_0449	.	WP_003455401. lactoylglutathion	1.087448	8.168538	1.293849	8.041254	-1.189803	7.981943	8.108289	8.229235	7.857566	8.108289	8.229235	7.857566	239.451985	351.831583	227.273512	1930	2711	2449
gene453	NC_008261.1	534603	535460	-	protein_coding	CPF_RS02255	CPF_0450	.	WP_003450023. AraC family tran	2.074704	2.155850	2.627880	1.926510	-1.266629	1.512671	1.692746	2.745651	1.351752	1.692746	2.745651	1.351752	1.652799	8.240983	1.359915	30	143	33
gene454	NC_008261.1	535649	536797	+	protein_coding	CPF_RS02260	CPF_0451	.	WP_011590197. L-threonine del	-1.324657	6.866920	1.468738	6.383797	-1.945574	6.575122	7.072724	6.667105	6.112528	7.072724	6.667105	6.112528	110.913898	139.429690	60.006680	2696	3240	1950
gene455	NC_008261.1	536987	537310	+	protein_coding	CPF_RS02265	CPF_0452	.	WP_003455301. CGGC domain-c	1.753133	3.850751	2.116925	3.696593	-1.207510	3.328223	3.467019	4.276954	3.194984	3.467019	4.276954	3.194984	7.586552	28.080387	7.202511	52	184	66
gene456	NC_008261.1	537424	537816	+	protein_coding	CPF_RS02270	CPF_0453	.	WP_003455372. VOC family prot	1.567692	2.820235	2.224147	2.521423	-1.418740	2.248082	2.514501	3.163143	2.009891	2.514501	3.163143	2.009891	3.608401	11.575121	2.789036	30	92	31
gene457	NC_008261.1	538113	540347	+	protein_coding	CPF_RS02275	CPF_0454	.	WP_011590198. SH3 domain-co	1.212399	6.774075	1.541033	6.595110	-1.271061	6.461235	6.636568	6.914432	6.290534	6.636568	6.914432	6.290534	78.339277	159.443667	68.231818	3704	7207	4313
gene459	NC_008261.1	541443	543263	+	protein_coding	CPF_RS02285	CPF_0456	.	WP_011590199. FUSC family prot	1.297760	0.414762	1.185857	0.505697	1.094365	0.325994	0.267374	0.643397	0.397467	0.267374	0.643397	0.397467	0.155750	0.841748	0.271834	6	31	14
gene460	NC_008261.1	543568	544077	+	protein_coding	CPF_RS02290	CPF_0457	.	WP_003455400. DedA family prot	-1.181843	0.458948	-1.180886	0.458497	-1.000810	0.594443	0.595027	0.353989	0.593859	0.595027	0.353989	0.593859	0.370746	0.387811	0.415974	4	4	6
gene461	NC_008261.1	544136	544768	+	protein_coding	CPF_RS02295	CPF_0458	.	WP_003455619. hypothetical pro	-1.230704	0.423965	-1.130564	0.378190	-1.088575	0.534660	0.599374	0.299891	0.476934	0.599374	0.299891	0.476934	0.373381	0.312454	0.335145	5	4	6
gene462	NC_008261.1	544990	545694	+	protein_coding	CPF_RS02300	CPF_0459	.	WP_003471479. DNA-binding res	-1.669457	1.226473	-1.205859	1.037570	-1.384454	1.396431	1.650668	0.911289	1.181351	1.650668	0.911289	1.181351	1.609193	1.262448	1.103363	24	18	22
gene463	NC_008261.1	545669	547900	+	protein_coding	CPF_RS02305	CPF_0460	.	WP_011590200. sensor histidine	-1.132469	2.218521	1.058236	2.089369	-1.198420	2.175590	2.310071	2.130599	2.048938	2.310071	2.130599	2.048938	2.943789	4.873700	2.867275	139	220	181
gene465	NC_008261.1	548818	549855	+	protein_coding	CPF_RS02315	CPF_0462	.	WP_011590202. FUSC family prot	2.466516	3.619440	3.151376	3.401483	-1.277663	2.844083	3.026324	4.328799	2.672817	3.026324	4.328799	2.672817	5.419207	28.962531	4.939189	119	608	145
gene466	NC_008261.1	550623	551036	+	protein_coding	CPF_RS02320	CPF_0463	.	WP_011590203. hypothetical pro	1.455349	1.181124	1.125231	1.394721	1.293378	1.111245	0.941061	1.482426	1.312205	0.941061	1.482426	1.312205	0.685073	2.627560	1.281079	6	22	15
gene467	NC_008261.1	551513	552211	+	protein_coding	CPF_RS02325	CPF_0464	.	WP_011590204. capsular polysac	4.852078	4.458227	3.832691	4.672158	1.265972	3.628334	3.462198	5.740800	3.802443	3.462198	5.740800	3.802443	7.574029	77.670383	11.128343	112	1098	220
gene468	NC_008261.1	552224	552877	+	protein_coding	CPF_RS02330	CPF_0465	.	WP_003471542. tyrosine protein	3.858082	5.284558	4.917579	5.069962	-1.274618	4.220955	4.399615	6.347499	4.049550	4.399615	6.347499	4.049550	15.467572	112.273946	13.083461	214	1485	242
gene469	NC_008261.1	553078	553746	+	protein_coding	CPF_RS02335	CPF_0466	.	WP_011590205. sugar transferas	4.483175	3.829459	3.326489	4.104135	1.347720	3.105000	2.897192	5.061713	3.327713	2.897192	5.061713	3.327713	4.946045	46.415583	7.927754	70	628	150
gene471	NC_008261.1	554774	556003	+	protein_coding	CPF_RS02345	CPF_0468	.	WP_011590206. nucleotide suga	3.865472	4.108732	2.449411	4.505908	1.578123	3.561515	3.247584	5.198228	3.905793	3.247584	5.198228	3.905793	6.456398	52.058894	11.987150	168	1295	417
gene473	NC_008261.1	557181	557780	+	protein_coding	CPF_RS02355	CPF_0470	.	WP_011590207. hexapeptide rep	3.577850	2.558167	3.310903	2.636511	1.080627	1.853960	1.798869	3.637962	1.910738	1.798869	3.637962	1.910738	1.890802	17.388475	2.475045	24	211	42
gene474	NC_008261.1	557784	559688	+	protein_coding	CPF_RS02360	CPF_0471	.	WP_049752143. hypothetical pro	3.293199	2.728723	2.560769	2.965842	1.286020	2.175115	2.001215	3.720704	2.364127	2.001215	3.720704	2.364127	2.233231	18.376744	3.786345	90	708	204
gene475	NC_008261.1	559699	561078	+	protein_coding	CPF_RS02365	CPF_0472	.	WP_011590209. phenylacetate--	3.970501	2.486869	2.647347	2.886595	1.499804	1.954383	1.683747	3.673068	2.268520	1.683747	3.673068	2.268520	1.644176	17.771859	3.484535	48	496	136
gene476	NC_008261.1	561106	562176	+	protein_coding	CPF_RS02370	CPF_0473	.	WP_011590210. group 1 glycosyl	2.470563	1.472225	2.464625	1.474891	1.002409	0.959624	0.957890	2.262730	0.961362	0.957890	2.262730	0.961362	0.706182	5.493989	0.825345	16	119	25
gene478	NC_008261.1	563631	564848	+	protein_coding	CPF_RS02380	CPF_0475	.	WP_011590212. hypothetical pro	1.896270	5.059857	2.147938	4.960413	-1.132717	4.528499	4.619285	5.542450	4.439498	4.619285	5.542450	4.439498	18.628593	66.861573	17.678891	480	1647	609
gene479	NC_008261.1	564848	565999	+	protein_coding	CPF_RS02385	CPF_0476	.	WP_011590213. hypothetical pro	1.863491	2.297900	2.336080	2.090564	-1.253604	1.721608	1.892352	2.790360	1.566270	1.892352	2.790360	1.566270	2.051652	8.541436	1.688089	50	199	55
gene480	NC_008261.1	565971	567206	+	protein_coding	CPF_RS02390	CPF_0477	.	WP_080503682. hypothetical pro	1.778139	3.361717	1.816380	3.344311	-1.021506	2.956686	2.972075	3.802443	2.941377	2.972075	3.802443	2.941377	5.201236	19.522329	5.892964	136	488	206
gene481	NC_008261.1	567223	568551	+	protein_coding	CPF_RS02395	CPF_0478	.	WP_011590215. group 1 glycosyl	2.045006	1.568073	2.439289	1.381196	-1.192803	0.999518	1.134755	2.166860	0.880399	1.134755	2.166860	0.880399	0.889203	5.022721	0.744934	25	135	28
gene482	NC_008261.1	568563	569444	+	protein_coding	CPF_RS02400	CPF_0479	.	WP_011590216. glucose-1-phos	1.360932	5.187766	1.727562	5.004942	-1.269396	4.795071	4.970229	5.414824	4.626086	4.970229	5.414824	4.626086	23.635028	60.770244	20.324711	441	1084	507
gene485	NC_008261.1	571045	572094	+	protein_coding	CPF_RS02415	CPF_0482	.	WP_011590217. dTDP-glucose 4	-1.011275	0.792140	1.271476	0.585757	-1.285812	0.591769	0.800270	0.784094	0.437590	0.800270	0.784094	0.437590	0.540229	1.083101	0.303067	12	23	9
gene486	NC_008261.1	572270	573190	+	protein_coding	CPF_RS02420	CPF_0483	.	WP_011590218. UTP--glucose-	1.532355	6.636349	1.671809	6.570207	-1.091006	6.272467	6.335611	6.951361	6.209952	6.335611	6.951361	6.209952	64.515157	163.101675	64.112374	1257	3038	1670
gene487	NC_008261.1	573555	574202	+	protein_coding	CPF_RS02425	CPF_0484	.	WP_011590219. RNA polymerase	-2.355847	2.022026	-3.795498	2.262320	1.611097	3.057245	2.732518	1.496272	3.420561	2.732518	1.496272	3.420561	4.230962	2.670689	8.512059	58	35	156
gene488	NC_008261.1	574617	576845	+	protein_coding	CPF_RS02430	CPF_0485	.	WP_011590220. hypothetical pro	-1.182366	1.041429	-1.171495	1.035478	-1.009279	1.162572	1.169254	0.927578	1.155929	1.169254	0.927578	1.155929	0.933101	1.308797	1.078658	44	59	68
gene489	NC_008261.1	576923	577939	+	protein_coding	CPF_RS02435	CPF_0486	.	WP_011590221. UDP-glucose 4-	2.242122	4.941843	2.755055	4.771765	-1.228771	4.242404	4.393614	5.558479	4.096398	4.393614	5.558479	4.096398	15.431326	67.435066	13.802399	332	1387	397
gene490	NC_008261.1	578221	579165	+	protein_coding	CPF_RS02440	CPF_0487	.	WP_003455278. UTP--glucose-	3.179941	7.033897	3.053719	7.066708	1.041334	6.277874	6.248726	7.917726	6.307159	6.248726	7.917726	6.307159	60.575700	290.448876	68.919613	1211	5551	1842
gene492	NC_008261.1	580328	580403	+	tRNA	CPF_RS02450	CPF_0489	.	. tRNA-Met	1.220027	1.915820	1.333981	1.845153	-1.093329	1.712154	1.777727	2.064640	1.649000	1.777727	2.064640	1.649000	1.865923	4.554228	1.860936	3	7	4
gene493	NC_008261.1	580766	585439	+	protein_coding	CPF_RS02455	CPF_0490	.	WP_011590223. cell wall-binding	-1.336695	2.816346	-1.938214	3.055074	1.450005	3.290581	3.033450	2.614780	3.569508	3.033450	2.614780	3.569508	5.430898	7.394669	9.387892	537	699	1241
gene494	NC_008261.1	585866	588070	+	protein_coding	CPF_RS02460	CPF_0491	.	WP_011590224. alpha-galactosid	-2.179247	7.714862	-1.915060	7.627692	-1.137952	8.203464	8.297213	7.173383	8.110773	8.297213	7.173383	8.110773	269.299969	187.849569	267.356145	12562	8377	16673
gene495	NC_008261.1	588204	588953	-	protein_coding	CPF_RS02465	CPF_0492	.	WP_003455633. SrtB family sorta	1.367349	2.996021	1.102813	3.158818	1.239874	2.929813	2.778819	3.230200	3.089012	2.778819	3.230200	3.089012	4.411872	12.460367	6.647263	70	189	141
gene496	NC_008261.1	589192	589716	+	protein_coding	CPF_RS02470	CPF_0493	.	WP_011590225. signal peptidase	-1.471267	1.494608	-1.298180	1.417611	-1.133331	1.706198	1.798869	1.241810	1.618300	1.798869	1.241810	1.618300	1.890802	1.977836	1.818400	21	21	27
gene497	NC_008261.1	589770	590405	+	protein_coding	CPF_RS02475	CPF_0494	.	WP_011590226. hypothetical pro	-1.103864	2.603078	-1.269498	2.699420	1.150050	2.774351	2.675335	2.532773	2.877031	2.675335	2.532773	2.877031	4.013495	6.841571	5.559400	54	88	100
gene498	NC_008261.1	590453	591151	+	protein_coding	CPF_RS02480	CPF_0495	.	WP_011590227. hypothetical pro	-1.030230	0.847663	-1.119212	0.904051	1.086372	0.927253	0.869418	0.826452	0.988936	0.869418	0.826452	0.988936	0.608627	1.131809	0.859917	9	16	17
gene499	NC_008261.1	591175	591882	+	protein_coding	CPF_RS02485	CPF_0496	.	WP_011590228. hypothetical pro	-1.126722	1.024137	-1.143197	1.033720	1.014622	1.124235	1.113812	0.941681	1.134755	1.113812	0.941681	1.134755	0.867953	1.326938	1.048748	13	19	21
gene500	NC_008261.1	591902	593644	+	protein_coding	CPF_RS02490	CPF_0497	.	WP_011590229. VWA domain-co	-1.009516	1.060133	-1.012130	1.061985	1.002590	1.068851	1.066987	1.053323	1.070718	1.066987	1.053323	1.070718	0.813598	1.531887	0.973708	30	54	48
gene501	NC_008261.1	593925	594641	+	protein_coding	CPF_RS02495	CPF_0498	.	WP_011590230. DNA-binding res	1.417298	4.443755	1.147518	4.602113	1.235099	4.348945	4.199298	4.702442	4.503925	4.199298	4.702442	4.503925	13.185510	36.618930	18.591191	200	531	377
gene502	NC_008261.1	594646	595941	+	protein_coding	CPF_RS02500	CPF_0499	.	WP_011590231. GHKL domain-c	1.365569	3.755268	1.369044	3.753322	-1.002545	3.535403	3.537237	3.986739	3.533570	3.537237	3.986739	3.533570	7.914816	22.815315	9.248679	217	598	339
gene503	NC_008261.1	596119	596925	+	protein_coding	CPF_RS02505	CPF_0500	.	WP_011590232. SGNH/GDSL hy	1.090130	6.821795	1.248459	6.722350	-1.145238	6.661287	6.759829	6.884330	6.564181	6.759829	6.884330	6.564181	86.808205	157.957284	83.596838	1482	2578	1908
gene504	NC_008261.1	597293	597736	+	protein_coding	CPF_RS02510	CPF_0501	.	WP_003449981. PTS L-Ascorba	1.892416	3.003300	1.605627	3.138355	1.178615	2.694166	2.578226	3.498456	2.815319	2.578226	3.498456	2.815319	3.832707	15.702414	5.415156	36	141	68
gene505	NC_008261.1	597914	598186	+	protein_coding	CPF_RS02515	CPF_0502	.	WP_003455220. PTS ascorbate t	1.578701	3.020036	1.284539	3.181564	1.229001	2.853444	2.708575	3.367312	3.006061	2.708575	3.367312	3.006061	4.155609	13.946279	6.216753	24	77	48
gene506	NC_008261.1	598227	599498	+	protein_coding	CPF_RS02520	CPF_0503	.	WP_003455659. PTS ascorbate t	1.555539	2.396019	1.620198	2.362236	-1.041567	2.068829	2.098415	2.735829	2.039659	2.098415	2.735829	2.039659	2.415530	8.124366	2.835294	65	209	102
gene507	NC_008261.1	599856	600650	+	protein_coding	CPF_RS02525	CPF_0504	.	WP_011590233. Cof-type HAD-I	1.352177	5.811397	1.364726	5.804475	-1.009280	5.591162	5.597829	6.033113	5.584502	5.597829	6.033113	5.584502	37.518748	94.475876	40.339004	631	1519	907
gene508	NC_008261.1	600784	601230	-	protein_coding	CPF_RS02530	CPF_0505	.	WP_003478780. MarR family tran	1.540883	4.910408	-1.086861	5.291924	1.674726	4.966477	4.608424	5.232181	5.352349	4.608424	5.232181	5.352349	18.400424	53.428119	34.804081	174	483	440
gene509	NC_008261.1	601555	603852	+	protein_coding	CPF_RS02535	CPF_0506	.	WP_011590234. ABC transporte	-1.305724	5.779275	-3.589903	6.446428	2.749357	6.664639	5.974903	5.590052	7.433997	5.974903	5.590052	7.433997	48.031148	69.435125	164.895279	2335	3227	10717
gene510	NC_008261.1	603845	605701	+	protein_coding	CPF_RS02540	CPF_0507	.	WP_011590235. ABC transporte	-1.285979	6.767496	-3.033919	7.345587	2.359229	7.545158	6.951361	6.588495	8.189677	6.951361	6.588495	8.189677	100.292955	134.678169	287.489042	3940	5058	15099
gene511	NC_008261.1	606198	607079	+	protein_coding	CPF_RS02545	CPF_0508	.	WP_003478774. hypothetical pro	1.475657	1.304140	1.497128	1.291174	-1.014550	1.042851	1.053323	1.614680	1.032483	1.053323	1.614680	1.032483	0.803913	3.027300	0.922028	15	54	23
gene512	NC_008261.1	607334	608383	+	protein_coding	CPF_RS02550	CPF_0509	.	WP_003449992. malate permeas	13.967595	3.822877	21.127342	3.305994	-1.512597	2.047733	2.367891	6.171903	1.770863	2.367891	6.171903	1.770863	3.106318	101.717279	2.087793	69	2160	62
gene513	NC_008261.1	608710	609708	+	protein_coding	CPF_RS02555	CPF_0510	.	WP_011009879. lactate dehydro	14.224570	4.891428	24.487309	4.278873	-1.721480	2.919834	3.337832	7.168145	2.554183	3.337832	7.168145	2.554183	6.908336	187.835023	4.353361	146	3795	123
gene514	NC_008261.1	610004	611554	+	protein_coding	CPF_RS02560	CPF_0511	.	WP_011590236. sensor histidine	-2.383201	3.275661	-2.170002	3.219277	-1.098249	3.893286	3.961476	2.708575	3.826271	3.961476	2.708575	3.826271	11.154636	7.970003	11.375586	366	250	499
gene515	NC_008261.1	611554	612222	+	protein_coding	CPF_RS02565	CPF_0512	.	WP_003471331. response regula	-3.405126	3.308505	-3.078106	3.252094	-1.106241	4.234924	4.308383	2.540675	4.162718	4.308383	2.540675	4.162718	14.272872	6.873645	14.534215	202	93	275
gene516	NC_008261.1	612303	612482	+	protein_coding	CPF_RS02570	CPF_0513	.	WP_003450214. hypothetical pro	-2.265027	5.355283	-2.422488	5.398543	1.069518	6.025709	5.977424	4.797895	6.074385	5.977424	4.797895	6.074385	48.057889	39.282020	58.143908	183	143	296
gene517	NC_008261.1	612640	613170	+	protein_coding	CPF_RS02575	CPF_0514	.	WP_003461219. hypothetical pro	-1.467751	7.770609	-1.504899	7.787988	1.025310	8.070350	8.052341	7.498734	8.088401	8.052341	7.498734	8.088401	231.009026	228.419569	265.016897	2595	2453	3980
gene518	NC_008261.1	614301	614942	+	protein_coding	CPF_RS02580	CPF_0516	.	WP_011590238. membrane prote	1.861171	1.645598	1.821091	1.666023	1.022009	1.273019	1.257413	2.153623	1.288820	1.257413	2.153623	1.288820	1.030811	5.006205	1.266712	14	65	23
gene519	NC_008261.1	615154	615567	+	protein_coding	CPF_RS02585	CPF_0517	.	WP_003471360. hypothetical pro	-3.367573	6.081919	-3.022917	6.014070	-1.114014	6.942195	7.020516	5.268807	6.864748	7.020516	5.268807	6.864748	105.957998	54.820454	105.133888	928	459	1231
gene520	NC_008261.1	615878	616993	+	protein_coding	CPF_RS02590	CPF_0518	.	WP_003474476. NAD-dependent	-1.599836	5.107973	-1.992288	5.253984	1.245307	5.614190	5.458169	4.780245	5.774671	5.458169	4.780245	5.774671	33.758274	39.033904	46.605105	797	881	1471
gene521	NC_008261.1	617002	617190	+	protein_coding	CPF_RS02595	CPF_0519	.	WP_003449870. hypothetical pro	1.240896	2.159978	-1.204442	2.451787	1.494587	2.281423	2.009891	2.321273	2.589638	2.009891	2.321273	2.589638	2.250955	5.755607	4.489877	9	22	24
gene522	NC_008261.1	617226	617906	+	protein_coding	CPF_RS02600	CPF_0520	.	WP_003449949. potassium trans	-1.699186	5.795357	-1.860536	5.856298	1.094958	6.255478	6.190382	5.425539	6.321258	6.190382	5.425539	6.321258	57.543136	61.208360	69.677156	829	843	1342
gene523	NC_008261.1	618021	618473	-	protein_coding	CPF_RS02605	CPF_0521	.	WP_003455298. MarR family tran	1.395702	0.358845	1.343881	0.406788	1.038560	0.217069	0.191486	0.672476	0.246071	0.191486	0.672476	0.246071	0.104349	0.873217	0.156105	1	8	2
gene524	NC_008261.1	618727	619860	+	protein_coding	CPF_RS02610	CPF_0522	.	WP_003478759. AI-2E family tra	1.037188	0.445508	-1.042954	0.502046	1.081739	0.473242	0.419947	0.472624	0.533300	0.419947	0.472624	0.533300	0.250106	0.566840	0.374156	6	13	12
gene525	NC_008261.1	620264	621655	+	protein_coding	CPF_RS02615	CPF_0523	.	WP_003450292. MFS transporte	39.109780	5.889762	58.700518	5.418258	-1.500917	3.506442	3.811578	9.101035	3.225734	3.811578	9.101035	3.225734	9.983762	635.124052	7.290002	294	17880	287
gene526	NC_008261.1	621817	622032	-	protein_coding	CPF_RS02620	CPF_0524	.	WP_003449914. hypothetical pro	-1.566043	8.039901	-1.332253	7.927081	-1.175484	8.252519	8.369971	7.722847	8.136716	8.369971	7.722847	8.136716	281.650745	261.422301	272.876957	1287	1142	1667
gene527	NC_008261.1	622046	622276	-	protein_coding	CPF_RS02625	CPF_0525	.	WP_003450005. hypothetical pro	1.897700	4.783751	1.285579	5.083709	1.476144	4.616272	4.343895	5.268147	4.905728	4.343895	5.268147	4.905728	14.733524	54.797187	25.102495	72	256	164
gene528	NC_008261.1	622525	624075	+	protein_coding	CPF_RS02630	CPF_0526	.	WP_011590240. nitrite/sulfite re	-4.296373	7.823928	-3.748941	7.737462	-1.146023	8.846973	8.945837	6.842718	8.749201	8.945837	6.842718	8.749201	417.384529	153.055940	419.255299	13695	4801	18391
gene529	NC_008261.1	624388	625203	+	protein_coding	CPF_RS02635	CPF_0527	.	WP_011009885. S-adenosylmeth	-1.112427	6.813348	-1.281059	6.913286	1.151589	6.991709	6.890637	6.736926	7.094263	6.890637	6.736926	7.094263	95.930406	145.004948	125.225478	1656	2393	2890
gene530	NC_008261.1	625221	626675	+	protein_coding	CPF_RS02640	CPF_0528	.	WP_003461164. aminotransferas	-1.219746	7.705013	-1.398037	7.801012	1.146170	7.947437	7.849636	7.563054	8.046457	7.849636	7.563054	8.046457	191.679261	239.345340	254.551730	5900	7043	10475
gene531	NC_008261.1	626690	627541	+	protein_coding	CPF_RS02645	CPF_0529	.	WP_003455287. polyamine amino	-1.234642	8.282966	-1.529472	8.433264	1.238799	8.589490	8.436408	8.132315	8.745349	8.436408	8.132315	8.745349	293.274074	329.987540	417.155428	5286	5686	10052
gene532	NC_008261.1	627528	628385	+	protein_coding	CPF_RS02650	CPF_0530	.	WP_003455394. agmatinase	-1.162676	9.009263	-1.416697	9.149010	1.218480	9.260087	9.118643	8.901194	9.403725	9.118643	8.901194	9.403725	473.912603	575.485724	682.883224	8602	9986	16571
gene533	NC_008261.1	628699	628962	-	protein_coding	CPF_RS02655	CPF_0531	.	WP_003450079. DUF1294 domai	-1.156091	6.044290	-1.067496	5.987498	-1.082994	6.092039	6.149823	5.940568	6.034798	6.149823	5.940568	6.034798	54.969344	89.152455	56.518879	307	476	422
gene534	NC_008261.1	629171	632692	+	protein_coding	CPF_RS02660	CPF_0532	.	WP_011590241. exo-alpha-sialid	-54.781420	5.461703	-42.135945	5.346434	-1.300111	8.874629	9.065965	3.290350	8.687330	9.065965	3.290350	8.687330	459.279183	12.972178	401.966381	34220	924	40040
gene535	NC_008261.1	632838	633479	+	protein_coding	CPF_RS02665	CPF_0533	.	WP_011009887. hypothetical pro	-21.302901	6.487127	-20.810657	6.475039	-1.023653	9.041719	9.058599	4.645621	9.024871	9.058599	4.645621	9.024871	457.312012	34.966415	522.546162	6211	454	9488
gene536	NC_008261.1	633592	636261	-	protein_coding	CPF_RS02670	CPF_0534	.	WP_011590242. copper-transloc	-12.947091	4.650734	-7.729805	4.390923	-1.674957	6.468700	6.851453	3.156897	6.107329	6.851453	3.156897	6.107329	92.397909	11.537377	59.750679	5219	623	4512
gene538	NC_008261.1	636553	637209	+	protein_coding	CPF_RS02675	CPF_0535	.	WP_003455358. FMN-dependent	1.029425	0.270982	-1.335281	0.455827	1.374571	0.421995	0.250869	0.292707	0.709851	0.250869	0.292707	0.709851	0.143897	0.301040	0.538170	2	4	10
gene539	NC_008261.1	638514	639650	+	protein_coding	CPF_RS02680	CPF_0537	.	WP_003461046. ABC transporte	1.600412	2.292432	1.552831	2.317524	1.030641	1.999825	1.978173	2.656616	2.021715	1.978173	2.656616	2.021715	2.203441	7.610407	2.798769	53	175	90
gene540	NC_008261.1	639643	641196	+	protein_coding	CPF_RS02685	CPF_0538	.	WP_011590244. ABC transporte	-1.089371	1.650008	-1.500821	1.859366	1.377695	1.930251	1.712911	1.589415	2.175167	1.712911	1.589415	2.175167	1.703425	2.959118	3.185386	56	93	140
gene541	NC_008261.1	641455	642030	+	protein_coding	CPF_RS02690	CPF_0539	.	WP_003471190. DUF1492 domai	1.008193	0.155438	-1.278872	0.288704	1.289349	0.277979	0.149663	0.161435	0.516307	0.149663	0.161435	0.516307	0.082066	0.171687	0.368310	1	2	6
gene542	NC_008261.1	642023	643114	+	protein_coding	CPF_RS02695	CPF_0540	.	WP_003471460. DUF4179 domai	1.189833	1.386712	1.293082	1.319378	-1.086776	1.205468	1.266989	1.517748	1.146935	1.266989	1.517748	1.146935	1.038902	2.716808	1.068504	24	60	33
gene543	NC_008261.1	643462	644883	+	protein_coding	CPF_RS02700	CPF_0541	.	WP_003471351. PTS trehalose t	-6.960890	1.992893	-4.842592	1.851882	-1.437431	3.563573	3.834919	1.035647	3.311426	3.834919	1.035647	3.311426	10.072311	1.495199	7.807555	303	43	314
gene544	NC_008261.1	645087	646754	+	protein_coding	CPF_RS02705	CPF_0542	.	WP_011590246. alpha,alpha-phos	-2.877972	2.873708	-2.337422	2.755850	-1.231259	3.582471	3.735680	2.210627	3.435545	3.735680	2.210627	3.435545	9.465347	5.246957	8.542678	334	177	403

gene545	NC_008261.1	647019	647726	+	protein_coding	CPF_RS02710	CPF_0543	.	WP_003471465.	trehalose opero	1.199968	3.677729	1.602478	3.454720	-1.335434	3.333403	3.548582	3.811578	3.131273	3.548582	3.811578	3.131273	8.011874	19.694553	6.841831	120	282	137
gene546	NC_008261.1	648176	650362	-	protein_coding	CPF_RS02715	CPF_0544	.	WP_003471431.	stage V sporulat	-1.616466	1.149172	-1.541959	1.123596	-1.048320	1.512251	1.546673	0.853830	1.478594	1.546673	0.853830	1.478594	1.448145	1.175668	1.535889	67	52	95
gene547	NC_008261.1	650709	651377	+	protein_coding	CPF_RS02720	.	.	WP_003471344.	N-acetylmuramo	-2.019319	3.788095	-2.253745	3.856803	1.116092	4.407315	4.328799	3.314930	4.487254	4.328799	3.314930	4.487254	14.484845	13.229919	18.445241	205	179	349
gene548	NC_008261.1	651951	653048	+	protein_coding	CPF_RS02725	CPF_0546	.	WP_003471179.	bifunctional diam	3.306030	2.584470	5.684048	1.968497	-1.719297	1.418260	1.862056	3.587156	1.080237	1.862056	3.587156	1.080237	1.980348	16.797195	0.998262	46	373	31
gene549	NC_008261.1	653070	653723	+	protein_coding	CPF_RS02730	CPF_0547	.	WP_003471205.	riboflavin syntha	3.496290	3.096186	8.345967	2.098712	-2.387092	1.574104	2.322241	4.128066	1.066987	2.322241	4.128066	1.066987	2.963413	24.949766	0.973150	41	330	18
gene550	NC_008261.1	653875	655074	+	protein_coding	CPF_RS02735	CPF_0548	.	WP_011590250.	bifunctional 3,4-	4.183330	3.456209	8.087248	2.744688	-1.933208	2.044701	2.574761	4.639413	1.623764	2.574761	4.639413	1.623764	3.820996	34.900564	1.826819	97	847	62
gene551	NC_008261.1	655201	655662	+	protein_coding	CPF_RS02740	CPF_0549	.	WP_003471176.	6,7-dimethyl-8-	9.053384	4.491205	16.594070	3.823250	-1.832913	2.702681	3.174863	6.353321	2.300725	3.174863	6.353321	2.300725	6.138968	112.484070	3.597004	60	1051	47
gene552	NC_008261.1	655789	656781	-	protein_coding	CPF_RS02745	CPF_0550	.	WP_011590251.	cell wall-binding	-1.718250	6.799203	-1.516327	6.713513	-1.133166	7.110124	7.200875	6.419935	7.020516	7.200875	6.419935	7.020516	121.911995	118.560611	119.034205	2561	2381	3343
gene553	NC_008261.1	656885	657292	-	protein_coding	CPF_RS02750	CPF_0551	.	WP_003457667.	hypothetical pro	-1.805123	5.007324	-1.852661	5.024518	1.026335	5.470184	5.451465	4.599368	5.488967	5.451465	4.599368	5.488967	33.598814	34.297033	38.217603	290	283	441
gene554	NC_008261.1	657691	657939	+	protein_coding	CPF_RS02755	CPF_0552	.	WP_003457734.	hypothetical pro	-1.919240	10.461506	-1.324705	10.202623	-1.448805	10.671556	10.942338	10.001803	10.407475	10.942338	10.001803	10.407475	1605.473316	1117.000739	1415.305275	8457	5625	9967
gene555	NC_008261.1	658230	660434	+	protein_coding	CPF_RS02760	CPF_0553	.	WP_011590252.	1,3-beta-galact	-5.248494	2.739277	-10.319347	3.041793	1.966154	4.647089	4.184921	1.793017	5.160297	4.184921	1.793017	5.160297	13.012664	3.587911	30.595305	607	160	1908
gene556	NC_008261.1	660619	662370	+	protein_coding	CPF_RS02765	CPF_0554	.	WP_011590253.	sensor histidine	-1.994433	2.979647	-1.989780	2.978220	-1.002338	3.517279	3.518965	2.522986	3.515595	3.518965	2.522986	3.515595	7.851362	6.801633	9.021078	291	241	447
gene557	NC_008261.1	662383	663972	+	protein_coding	CPF_RS02770	CPF_0555	.	WP_011590254.	DNA-binding res	-2.702526	1.891817	-2.702526	1.891817	1.000000	2.740340	2.740340	1.306032	2.740340	2.740340	1.306032	2.740340	4.251332	2.145765	5.181361	143	69	233
gene558	NC_008261.1	664125	665510	+	protein_coding	CPF_RS02775	CPF_0556	.	WP_011590255.	carbohydrate AE	-10.833167	4.696905	-16.756211	4.911882	1.546751	7.027755	6.720173	3.282790	7.349414	6.720173	3.282790	7.349414	83.865127	12.843091	151.992544	2459	360	5958
gene559	NC_008261.1	665589	666515	+	protein_coding	CPF_RS02780	CPF_0557	.	WP_011590256.	sugar ABC trans	-7.199103	3.060182	-13.224417	3.328159	1.836953	5.219403	4.799146	1.951329	5.676461	4.799146	1.951329	5.676461	21.110898	4.107157	43.596489	414	77	1143
gene560	NC_008261.1	666517	667398	+	protein_coding	CPF_RS02785	CPF_0558	.	WP_003449831.	carbohydrate AE	-8.486208	3.254965	-15.265444	3.512732	1.798853	5.551951	5.144544	2.059424	5.991621	5.144544	2.059424	5.991621	26.636301	4.540950	54.840642	497	81	1368
gene561	NC_008261.1	667634	668329	+	protein_coding	CPF_RS02790	CPF_0559	.	WP_003450082.	TVP38/TMEM6	1.821302	3.009647	1.802254	3.018385	1.010569	2.615649	2.608077	3.473047	2.623244	2.608077	3.473047	2.623244	3.871255	15.416322	4.622928	57	217	91
gene562	NC_008261.1	668319	669431	+	protein_coding	CPF_RS02795	CPF_0560	.	WP_003471256.	(Fe-S)-binding	1.928171	2.628792	1.941303	2.622928	-1.006811	2.192598	2.197499	3.144732	2.187707	2.197499	3.144732	2.187707	2.633193	11.461853	3.240336	62	258	102
gene563	NC_008261.1	669595	670182	+	protein_coding	CPF_RS02800	CPF_0561	.	WP_003457789.	CDP-alcohol ph	1.702463	2.185147	1.570345	2.253477	1.084133	1.892161	1.834787	2.602411	1.951329	1.834787	2.602411	1.951329	1.929390	7.315975	2.585688	24	87	43
gene564	NC_008261.1	670467	671099	+	protein_coding	CPF_RS02805	CPF_0562	.	WP_003450071.	DUF47 domain-	2.871134	4.948091	2.879910	4.945524	-1.003057	4.243227	4.245429	5.767050	4.241026	4.245429	5.767050	4.241026	13.516398	78.972835	15.528378	181	1011	278
gene565	NC_008261.1	671092	672090	+	protein_coding	CPF_RS02810	CPF_0563	.	WP_003450180.	inorganic phosp	3.492267	6.087827	3.483915	6.089828	1.002397	5.253943	5.252216	7.056380	5.255671	5.252216	7.056380	5.255671	28.579693	173.332872	32.880260	604	3502	929
gene566	NC_008261.1	672372	674264	-	protein_coding	CPF_RS02815	CPF_0564	.	WP_011590257.	PTS fructose tr	-2.336337	3.464877	-2.080866	3.394085	-1.122772	4.046262	4.130656	2.906408	3.963592	4.130656	2.906408	3.963592	12.610342	9.481702	12.420985	505	363	665
gene567	NC_008261.1	674268	675188	-	protein_coding	CPF_RS02820	CPF_0565	.	WP_011590258.	1-phosphofruct	-2.000514	2.455449	-1.881143	2.418925	-1.063457	2.961348	3.006061	2.005690	2.917300	3.006061	2.005690	2.917300	5.337770	4.294975	5.758596	104	80	150
gene568	NC_008261.1	675188	675934	-	protein_coding	CPF_RS02825	CPF_0566	.	WP_003465612.	DeoR/GlpR tran	-3.483444	2.144150	-3.075582	2.083586	-1.132613	3.134620	3.225734	1.425219	3.046079	3.225734	1.425219	3.046079	6.391266	2.449128	6.484627	101	37	137
gene569	NC_008261.1	676225	676677	+	protein_coding	CPF_RS02830	CPF_0567	.	WP_011590259.	hypothetical pro	-1.237482	0.321452	-1.194882	0.305110	-1.035652	0.484084	0.510013	0.202606	0.459474	0.510013	0.202606	0.459474	0.313047	0.218304	0.312210	3	2	4
gene570	NC_008261.1	676728	677828	-	protein_coding	CPF_RS02835	CPF_0568	.	WP_011590260.	AI-2E family trar	2.046137	4.676851	3.097782	4.329966	-1.513966	3.878140	4.188828	5.221731	3.590495	4.188828	5.221731	3.590495	13.051859	53.128519	9.505816	304	1183	296
gene571	NC_008261.1	678086	678745	+	protein_coding	CPF_RS02840	CPF_0569	.	WP_041702615.	cysteine hydrola	-1.052989	5.691148	1.021495	5.638662	-1.075624	5.675684	5.728515	5.654024	5.623341	5.728515	5.654024	5.623341	40.537653	72.895243	41.840042	566	973	781
gene572	NC_008261.1	679123	680436	+	protein_coding	CPF_RS02845	CPF_0570	.	WP_003480303.	voltage-gated c	-1.301075	0.967026	-1.106534	0.865583	-1.175811	1.052043	1.175338	0.795634	0.941681	1.175338	0.795634	0.941681	0.935328	1.091272	0.807255	26	29	30
gene573	NC_008261.1	680740	681663	+	protein_coding	CPF_RS02850	CPF_0571	.	WP_011590262.	glutaminase A	-10.380251	8.225424	-7.968659	8.068370	-1.302635	9.892148	10.084703	6.708933	9.703270	10.084703	6.708933	9.703270	949.289114	142.611820	845.755085	18556	2665	22102
gene574	NC_008261.1	681964	682686	+	protein_coding	CPF_RS02855	CPF_0572	.	WP_003480305.	MgtC/SapB fam	-1.277433	3.158202	-1.009369	2.993233	-1.265575	3.165308	3.339761	2.986514	2.999968	3.339761	2.986514	2.999968	6.930326	10.190095	6.210841	106	149	127
gene576	NC_008261.1	684844	687755	+	rRNA	CPF_RS02865	CPF_3014	.		23S ribosomal R	-1.337470	0.357257	-1.370554	0.367210	1.024736	0.641419	0.624035	0.204528	0.659287	0.624035	0.204528	0.659287	0.389588	0.220741	0.485684	24	13	40
gene579	NC_008261.1	688429	689121	+	protein_coding	CPF_RS02880	CPF_0574	.	WP_003460225.	DNA/RNA nucle	-1.305549	5.695994	1.011628	5.498566	-1.320730	5.687361	5.891568	5.506911	5.490232	5.891568	5.506911	5.490232	44.609836	65.143010	38.265998	654	913	750
gene580	NC_008261.1	689535	690074	+	protein_coding	CPF_RS02885	CPF_0575	.	WP_003460223.	NUDIX domain-	-1.259528	6.632937	-1.039234	6.496284	-1.211977	6.661342	6.801466	6.468584	6.524104	6.801466	6.468584	6.524104	89.025270	122.332818	82.108627	1017	1336	1254
gene581	NC_008261.1	690485	691780	+	protein_coding	CPF_RS02890	CPF_0576	.	WP_003460208.	diaminopimelate	1.729619	5.712744	1.279243	5.941326	1.352065	5.544487	5.331172	6.121627	5.766336	5.331172	6.121627	5.766336	30.601525	99.197020	46.379807	839	2600	1700
gene582	NC_008261.1	692237	692455	+	protein_coding	CPF_RS02895	CPF_0577	.	WP_003460209.	DUF3006 domai	1.835014	6.273315	1.310292	6.528621	1.400462	6.088790	5.850684	6.726475	6.336587	5.850684	6.726475	6.336587	43.600690	143.596310	70.392662	202	636	436
gene583	NC_008261.1	692458	692988	+	protein_coding	CPF_RS02900	CPF_0578	.	WP_003460206.	signal peptidase	1.230302	7.623070	1.185188	7.650503	1.038065	7.501930	7.475030	7.774043	7.528927	7.475030	7.774043	7.528927	142.255269	266.411898	177.787718	1598	2861	2670
gene584	NC_008261.1	693013	695166	+	protein_coding	CPF_RS02905	CPF_0579	.	WP_011590264.	ATP-dependent	-1.197250	8.810493	-1.310601	8.874557	1.094676	9.006328	8.941312	8.681588	9.071816	8.941312	8.681588	9.071816	414.435928	489.638363	537.178931	18885	21330	32725
gene585	NC_008261.1	695386	697506	+	protein_coding	CPF_RS02910	CPF_0580	.	WP_011590265.	ATP-dependent	-1.381806	4.964596	-1.867465	5.167734	1.351467	5.416258	5.203351	4.736796	5.637877	5.203351	4.736796	5.637877	27.724634	37.859567	42.242631	1244	1624	2534
gene586	NC_008261.1	697962	698798	+	protein_coding	CPF_RS02915	CPF_0581	.	WP_011590266.	amino acid ABC	1.094999	7.849220	-1.972208	8.390574	2.159566	8.320885	7.784028	7.914958	8.894769	7.784028	7.914958	8.894769	181.568969	289.940829	462.354748	3215	4908	10945
gene587	NC_008261.1	698829	699500	+	protein_coding	CPF_RS02920	CPF_0582	.	WP_011590267.	amino acid ABC	-1.456411	8.343054	-2.981471	8.829168	2.047135	9.120843	8.618670	8.076252	9.652276	8.618670	8.076252	9.652276	328.850457	316.836373	811.966209	4675	4306	15432
gene588	NC_008261.1	699497	700219	+	protein_coding	CPF_RS02925	CPF_0583	glnQ	WP_011009923.	amino acid ABC	-1.451640	9.222180	-3.301936	9.781032	2.274625	10.070320	9.494939	8.957256	10.680568	9.494939	8.957256	10.680568	645.631803	593.829522	1672.183459	9875	8683	34193
gene589	NC_008261.1	700437	702245	+	protein_coding	CPF_RS02930	CPF_0584	.	WP_003460224.	LTA synthase fa	1.308957	6.604403	1.420685	6.543272	-1.085356	6.353690	6.413049	6.801466	6.294880	6.413049	6.801466	6.294880	67.991533	149.376363	68.389651	2602	5465	3499
gene590	NC_008261.1	702497	704341	+	protein_coding	CPF_RS02935	CPF_0585	.	WP_011590268.	LTA synthase fa	-1.741848	7.223928	-1.460094	7.102486	-1.192970	7.506962	7.635320	6.834701	7.380763	7.635320	6.834701	7.380763	160.615705	152.625689	158.180560	6269	5695	8254
gene591	NC_008261.1	704443	705624	-	protein_coding	CPF_RS02940	CPF_0586	.	WP_011590269.	cation transport	2.228586	5.501750	2.107059	5.546489	1.057676	4.994256	4.953971	6.110099	5.034868	4.953971	6.110099	5.034868	23.475061	98.766302	28.028969	587	2361	937
gene592	NC_008261.1	705867	707528	+	protein_coding	CPF_RS02945	CPF_0587	.	WP_011590270.	enterotoxin	-1.610587	4.890929	-1.214721	4.697421	-1.325891	5.039203	5.246791	4.559204	4.839829	5.246791	4.559204	4.839829	28.555436	32.904431	23.827146	1004	1106	1120
gene593	NC_008261.1	707670	708965	+	protein_coding	CPF_RS02950	CPF_0588	.	WP_011590271.	M18 family amin	-1.511572	8.415599	-1.195412	8.250599	-1.264477	8.547953	8.718900	8.122850	8.380358	8.718900	8.122850	8.380358	359.084639	328.952580	336.417292	9845	8622	12331
gene594	NC_008261.1	709058	709252	-	protein_coding	CPF_RS02955	CPF_0589	.	WP_003448762.	hypothetical pro	-1.121514	0.311138	-1.203880	0.348232	1.073442	0.452917	0.404671	0.239223	0.506914	0.404671	0.239223	0.506914	0.242411	0.253569	0.362644	1	1	2
gene595	NC_008261.1	709644	710483	+	protein_coding	CPF_RS02960	CPF_0590	.	WP_003460189.	Cof-type HAD-I	1.667023	4.631570	2.369705	4.348188	-1.421519	4.015857	4.277580	5.014854	3.770147	4.277580	5.014854	3.770147	13.843373	45.031087	10.859890	246	765	258
gene596	NC_008261.1	710691	714020	-	protein_coding	CPF_RS02965	CPF_0591	.	WP_011590272.	MMPL family tra	-2.415752	4.066601	-2.670858	4.128103	1.105601	4.824180	4.752308	3.479835	4.897138	4.752308	3.479835	4.897138	20.412714	15.516806	24.835391	1438	1045	2339
gene597	NC_008261.1	714281	715876	-	protein_coding	CPF_RS02970	CPF_0592	.	WP_003448736.	peptide chain re	3.657395	5.656481	3.146739	5.782959	1.162281	4.905175	4.797895	6.668712	5.014854	4.797895	6.668712	5.014854	21.087894	139.477092	27.648190	712	4502	1248
gene598	NC_008261.1	716090	716695	-	protein_coding	CPF_RS02975	CPF_0593	.	WP_003448743.	hypothetical pro	2.719485	5.022385	2.684029	5.033297	1.013210	4.361758	4.352302	5.795635	4.371235	4.352302	5.795635	4.371235	14.898648	81.022736	16.978737	191	993	291
gene599	NC_008261.1	717071	717871	+	protein_coding	CPF_RS02980	CPF_0594	.	WP_003460506.	Cof-type HAD-I	1.992682	5.661985	2.167924	5.595215	-1.087943	5.125269	5.186431	6.181143	5.064828	5.186431	6.181143	5.064828	27.500432	102.533881	28.604048	466	1661	648
gene600	NC_008261.1	717912	718574	+	protein_coding	CPF_RS02985	CPF_0595	.	WP_003476898.	sugar transferas	1.908511	5.756217	1.972420	5.730397	-1.033486	5.285030	5.308843	6.241290	5.261323	5.308843	6.241290	5.261323	29.873534	105.753070	33.011263	419	1418	619
gene601	NC_008261.1	718575	719489	+	protein_coding	CPF_RS02990	CPF_0596	.	WP_003460502.	rhamnosyltransf	2.822435	4.406054	2.181687	4.620785	1.293694	3.902034	3.720704	5.217645	4.092201	3.720704	5.217645	4.092201	9.299027	53.066528	13.679404	180	982	354
gene602	NC_008261.1	719512	720393	+	protein_coding	CPF_RS02995	CPF_0597	.	WP_011009932.	glucose-1-phos	1.892179	4.937666	2.035608	4.879479	-1.075801	4.446007	4.499025	5.419074	4.393614	4.499025	5.419074	4.393614	16.935757	61.106610	17.197833	316	1090	429
gene605	NC_008261.1	721994	723043	+	protein_coding	CPF_RS03010	CPF_0600	.	WP_003460529.	dTDP-glucose 4	2.340429	2.188714	2.125453	2.278529	1.101144	1.727758	1.659654	2.886427	1.798657	1.659654	2.886427	1.798657	1.620688	9.324084	2.155141	36	198	64
gene606	NC_008261.1	723068	724282	+	protein_coding	CPF_RS03015	CPF_0601	.	WP_003448723.	O-antigen ligase	2.321109	1.170150	2.237988	1.212671	1.037141	0.736835	0.710999	1.925813	0.763611	0.710999	1.925813	0.763611	0.466865	4.028925	0.582021	12	99	20
gene607	NC_008261.1	724279	725127	+	protein_coding	CPF_RS03020	CPF_0602	.	WP_003460503.	glycosyltransfer	3.554157	4.372215	3.004805	4.518837	1.182825	3.671250	3.552129	5.381636	3.794365	3.552129	5.381636	3.794365	8.017536	59.288487	11.077939	144	1018	266
gene608	NC_008261.1	725221	725961	+	protein_coding	CPF_RS03025	CPF_0603	.	WP_003448712.	hypothetical pro	3.063742	4.104895	2.564383	4.258090	1.194729	3.501937	3.375946	4.991241	3.632629	3.375946	4.991241	3.632629	7.080939	44.507982	9.829559	111	667	206
gene609	NC_008261.1	725970	727421	+	protein_coding	CPF_RS03030	CPF_0604	.	WP_011590273.	flippase	2.141937	2.973582	1.998084	3.033248	1.071995	2.524114	2.474463	3.573378	2.574761	2.474463	3.573378	2.574761	3.483399	16.584127	4.431898	107	487	182
gene610	NC_008261.1	727443	729866	+	protein_coding	CPF_RS03035	CPF_0605	.	WP_003460505.	cell wall-binding	1.531089	5.834458	1.627868	5.787668	-1.063209	5.490874	5.535265	6.149823	5.446839	5.535265	6.149823	5.446839	35.764555	100.890849	37.122754	1834	4946	2545
gene611	NC_008261.1	729995	731647	+	protein_coding	CPF_RS03040	CPF_0606	.	WP_003460499.	cell wall-binding	-1.781256	9.226435	-1.768737	9.221571	-1.007078	9.647188	9.652276	8.819382	9.642101	9.652276	8.819382	9.642101	718.573468	541.033262	802.726464	25128	18087	37528
gene612	NC_008261.1	731849	733213	+	protein_coding	CPF_RS03045	CPF_0607	.	WP_011590274.	cell wall-binding	-1.780459	4.278845	-2.252457	4.430100	1.265099	4.881835	4.715156	3.882907	5.054406	4.715156	3.882907	5.054406	19.877664	20.901307	28.493451	574	577	1100
gene613	NC_008261.1	733418	735286	+	protein_coding	CPF_RS03050	CPF_0608	.	WP_011590275.	choline kinase	65.917581	4.628109	25.266250	5.765894	2.608918	3.121700	2.505695	8.548286	3.889146	2.505695	8.548286	3.889146	3.591411	448.663995	11.880517	142	16959	628
gene614	NC_008261.1	735312	736280	+	protein_coding	CPF_RS03055	CPF_0609	.	WP_011590276.	EamA/RhaT fam	74.165284	5.430676	14.009639	7.211318	5.293875	4.182822	3.149986	9.362658	5.554310	3.149986	9.362658	5.554310	6.000224	752.149181	39.663477	123	14740	1087
gene615	NC_008261.1	736427	737110	+	protein_coding	CPF_RS03060	CPF_0610	.	WP_011009940.	lic-1 operon pro	101.992797	5.912597	12.652103	8.089837	8.061331	4.724127	3.452710	10.125034	6.463728	3.452710	10.125034	6.463728	7.532801	1194.364253	78.624542	109	16522	1521
gene616	NC_008261.1	737229	738521	-	protein_coding	CPF_RS03065	CPF_0611	.	WP_011590277.	cell wall-binding	3.191152	3.961697	1.963416	4.372512	1.625306	3.545205	3.212119	4.886197	3.912831	3.212119	4.886197	3.912831	6.288051	41.950620	12.086728	172	1097	442
gene617	NC_008261.1	738640	739650	-	protein_coding	CPF_RS03070	CPF_0612	.	WP_003453510.	LytR family trans	3.403350	5.826746	3.447571	5.815905	-1.012994	5.000545	5.009866	6.776821	4.991241	5.009866	6.776821	4.991241	24.546764	147.163908	27.174082	525	3009	777
gene618	NC_008261.1	739939	741213	-	protein_coding	CPF_RS03075	CPF_0613	.	WP_003453506.	branched-chain	7.365082	5.215881	7.113517	5.248708	1.035364	3.995742	3.970751	6.851453	4.020890	3.970751	6.851453	4.020890	11.233590	154.891704	12.895191	303	3994	465
gene619	NC_008261.1	741966	742964	+	protein_coding	CPF_RS03080	CPF_0614	.	WP_003476922.	tryptophan--tR	2.135832	5.927876	2.537854	5.789846	-1.188227	5.279827	5.405698	6.500496	5.156887	5.405698	6.500496	5.156887	32.507035	124.332432	30.473524	687	2512	861
gene620	NC_008261.1	743378	743917	+	protein_coding	CPF_RS03085	CPF_0615	.	WP_011590279.	hypothetical pro	-1.273333	1.182528	-1.076026	1.072555	-1.183367	1.242239	1.369611	1.021000	1.126713	1.369611	1.021000	1.126713	1.225520	1.465064	1.047638	14	16	16
gene621	NC_008261.1	744000	745112	+	protein_coding	CPF_RS03090	CPF_0616	.	WP_011590280.	anaerobic sulfat	-2.263344	5.588646	-1.971448	5.498264	-1.148062	6.108438	6.208850	5.030394	6.009650	6.208850	5.030394	6.009650	58.354947	45.447579	55.339853	1374	1023	1742
gene622	NC_008261.1	745323	746153	+	protein_coding	CPF_RS03095	CPF_0617	.	WP_011590281.	phosphate ABC	-1.505961	4.698610	-1.481908	4.687691	-1.016231	4.991598	5.003225	4.412541	4.979997	5.003225	4.412541	4.979997	24.459836	30.345859	26.890636	430	510	632
gene623	NC_008261.1	746377	747195	+	protein_coding	CPF_RS03100	CPF_0618	.	WP_011590282.	phosphate ABC	1.326432	1.298076	1.564697	1.150336	-1.179628	0.983841	1.110198	1.517748	0.871865	1.110198	1.517748	0.871865	0.865752	2.716808	0.733922	15	45	17
gene624	NC_008261.1	747277	748164	+	protein_coding	CPF_RS03105	CPF_0619	.	WP_003453504.	phosphate ABC	1.416141	1.569075	1.933296	1.278927	-1.365187	1.090613	1.338039	1.840003	0.888941	1.338039	1.840003	0.888941	1.171105	3.730715	0.756529	22	67	19
gene625	NC_008261.1	748167	748991	+	protein_coding	CPF_RS03110	CPF_0620	.	WP_003460575.	phosphate ABC	1.952933	1.291965	1.788561	1.380348	1.091901	0.957738	0.896419	1.862056	1.023256	0.896419	1.862056	1.023256	0.630267	3.835803	0.900016	11	64	21
gene626	NC_008261.1	749009	749770	+	protein_coding	CPF_RS03115	CPF_0621	.	WP_003453508.	phosphate ABC	1.731314	1.977974	1.852974	1.917278	-1.070270	1.571528	1.621279	2.413146	1.523303	1.621279	2.413146	1.523303	1.550855	6.164515	1.624045	25	95	35
gene627	NC_008261.1	749795	750448	+	protein_coding	CPF_RS03120	CPF_0622	.	WP_004460437.	phosphate trans	1.671151	2.059012	1.837094	1.975651	-1.099299	1.651943	1.721646	2.462488	1.585062	1.721646	2.462488	1.585062	1.734681	6.426455	1.730044	24	85	32
gene628	NC_008261.1	750608	751303	+	protein_coding	CPF_RS03125	CPF_0623	.	WP_011590283.	DNA-binding res	3.569834	3.038653	2.301192	3.438855	1.551298	2.553512	2.256344	4.092201	2.889819	2.256344	4.092201	2.889819	2.784587	24.580864	5.638957	41	346	111
gene629	NC_008261.1	751474	752097	-	protein_coding	CPF_RS03130	CPF_0624	.	WP_003464415.	DUF3793 domai	5.660651	8.970546	4.538748	9.152161	1.247733	7.964855	7.806800	10.307768	8.126110	7.806800	10.307768	8.126110	184.686529	1401.759551	271.246318	2438	17690	4787
gene630	NC_008261.1	752131	752547	-	protein_coding	CPF_RS03135	CPF_0625	.	WP_003480361.	flavodoxin	2.261655	11.569470	3.103869	11.326664	-1.372388	10.764982	10.995748	12.173127	10.539059	10.995748	12.173127	10.539059	1676.329914	5442.724764	1532.933673	14788	45901	18079
gene631	NC_008261.1	752961	754619	-	protein_coding	CPF_RS03140	CPF_0626	.	WP_003453104.	glutamine--tRN	1.162388	9.491571	1.434244	9.336969	-1.233878	9.230802	9.383646	9.600737	9.080447	9.383646	9.600737	9.080447	579.378722	848.597738	539.424631	20334	28472	25310
gene632	NC_008261.1	754873	755028	-	protein_coding	CPF_RS03145	CPF_0627	.	WP_003452998.	cytochrome c5	2.418254	13.837169	1.989019	13.983983	1.215802	13.355051	13.214840	14.488806	13.496749	13.214840	14.488806	13.496749	9934.286891	18896.257579	11574.460611	32785	59617	51067
gene634	NC_008261.1	756367	757788	+	protein_coding	CPF_RS03155	CPF_0629	.	WP_003461569.	spore germinati	-1.032086	0.729262	-1.048511	0.740219	1.015914	0.763704	0.752399	0.706836	0.775178	0.752399	0.706836	0.775178	0.498629	0.938846	0.596756	15	27	24
gene635	NC_008261.1	757763	758887	+	protein_coding	CPF_RS03160	CPF_0630	.	WP_003460753.	Ger(x)C family sp	-1.001495	0.962439	1.218534	0.806280	-1.220356	0.807184	0.963518	0.961362	0.676216	0.963518	0.961362	0.676216	0.714303	1.362509	0.502866	17	31	16
gene636	NC_008261.1	759081	760214	+	protein_coding	CPF_RS03165	CPF_0631	.	WP_011590285.	ATP-binding pro	-1.431580	3.359763	-1.132443	3.199374	-1.264152	3.455301	3.628520	3.110912	3.290350	3.628520	3.110912	3.290350	8.545292	11.162390	7.701386	205	256	247
gene637	NC_008261.1	760290	760703	+	protein_coding	CPF_RS03170	CPF_0632	.	WP_003453091.	hypothetical pro	-3.537753	3.830812	-3.794528	3.870535	1.072582	4.899440	4.849157	3.026324	4.950245	4.849157	3.026324	4.950245	21.922344	10.510239	26.048608	192	88	305
gene638	NC_008261.1	760987	762207	+	protein_coding	CPF_RS03175	CPF_0633	.	WP_011590286.	tyrosine--tRNA	1.167536	8.889013	1.266853	8.839178	-1.085065	8.728892	8.787981	9.011449	8.670200	8.787981	9.011449	8.670200	375.837588	611.493104	401.445481	9708	15100	13863
gene639	NC_008261.1	762463	763155	+	protein_coding	CPF_RS03180	CPF_0634	.	WP_011590287.	ser/threonine p	1.465802	5.608944	1.477774	5.602777	-1.008167	5.334007	5.339878	5.891568	5.328143	5.339878	5.891568	5.328143	30.899473	86.976265	34.388377	453	1219	674
gene640	NC_008261.1	763274	764065	+	protein_coding	CPF_RS03185	CPF_0635	.	WP_003453085.	GTP pyrophosph	1.250751	6.594891	1.124878	6.672839	1.111899	6.511532	6.435468	6.758263	6.588495	6.435468	6.758263	6.588495	68.696759	146.339884	85.135468	1151	2344	1907
gene641	NC_008261.1	764375	765160	+	protein_coding	CPF_RS03190	CPF_0636	.	WP_011590288.	hypothetical pro	2.043551	6.235063	3.339906	5.837515	-1.634364	5.374767	5.740800	6.771879	5.032071	5.740800	6.771879	5.032071	40.714793	147.079533	27.980331	677	2338	622
gene642	NC_008261.1	765278	766393	+	protein_coding	CPF_RS03195	CPF_0637	.	WP_011590289.	ATPase AAA	2.331600	5.382423	2.714549	5.258142	-1.164243	4.695315	4.806293	6.027613	4.586900	4.806293	6.027613	4.586900	21.220697	93.840874	19.833308	501	2118	626
gene643	NC_008261.1	766407	767744	+	protein_coding	CPF_RS03200	CPF_0638	.	WP_011590290.	hypothetical pro	2.229950	4.770377	2.471220	4.685995	-1.108195	4.152054	4.226821	5.383832	4.078609	4.226821	5.383832	4.078609	13.354320	59.423771	13.530033	378	1608	512
gene644	NC_008261.1	767902	769293	+	protein_coding	CPF_RS03205	CPF_0639	.	WP_011009952.	DUF2334 domai	2.535298	1.885899	2.660397	1.835999	-1.049343	1.295453	1.330661	2.672817	1.261176	1.330661	2.672817	1.261176	1.154585	7.708161	1.219234	34	217	48
gene645	NC_008261.1	769643	771136	+	protein_coding	CPF_RS03210	CPF_0640	.	WP_011009953.	YjjI family glycine	-4.339295	7.363323	-4.878490	7.436170	1.124259	8.581849	8.497778	6.380317	8.666752	8.497778	6.380317	8.666752	305.546802	115.009706	399.561870	9657	3475	16883
gene646	NC_008261.1	771145	771966	+	protein_coding	CPF_RS03215	CPF_0641	.	WP_011590292.	YjjW family glycin	-5.144221	6.473542	-5.466578	6.510005	1.062664	7.805670	7.761950	5.398998	7.849636	7.761950	5.398998	7.849636	177.924027	60.032856	227.158695	3094	998	5281
gene647	NC_008261.1	772148	772927	-	protein_coding	CPF_RS03220	CPF_0642	.	WP_003456934.	DeoR/GlpR tran	-2.483579	4.775552	-2.076657	4.661638	-1.195950	5.345999	5.476636	4.164216	5.218478	5.476636	4.164216	5.218478	34.058681	25.800617	31.867334	562	407	703
gene648	NC_008261.1	773373	774437	+	protein_coding	CPF_RS03225	CPF_0643	.	WP_003457064.	L-ascorbate 6-	-24.798850	4.311626	-27.442561	4.355101	1.106606	7.283132	7.210428	2.578226	7.356570	7.210428	2.578226	7.356570	122.591448	7.103495	153.117456	2762	153	4612
gene649	NC_008261.1	774462	774896	+	protein_coding	CPF_RS03230	CPF_0644	.	WP_011590293.	PTS sugar trans	-30.786333	3.744909	-36.802994	3.813570	1.195433	7.086984	6.959387	2.015169	7.216920	6.959387	2.015169	7.216920	100.842784	4.319412	135.660088	928	38	1669
gene650	NC_008261.1	775083	776549	+	protein_coding	CPF_RS03235	CPF_0645	.	WP_003456976.	PTS ascorbate	-43.311981	3.204364	-45.897866	3.223675	1.059704	6.962118	6.920413	1.483719	7.004074	6.920413	1.483719	7.004074	97.923447	2.629025	117.353129	3039	78	4869
gene651	NC_008261.1	776646	776936	+	protein_coding	CPF_RS03240	CPF_0646	.	WP_003457096.	PTS ascorbate	-35.690417	3.318300	-42.773757	3.381601	1.198466	6.910590	6.781229	1.623764	7.042418	6.781229	1.623764	7.042418	88.204948	3.058509	120.653875	543	18	993
gene652	NC_008261.1	777044	778078	+	protein_coding	CPF_RS03245	CPF_0647	.	WP_003457100.	hypothetical pro	-20.332470	3.884223	-19.889228	3.874888	-1.022286	6.607612	6.623530	2.277816	6.591732	6.623530	2.277816	6.591732	77.321935	5.541763	85.473595	1693	116	2502
gene653	NC_008261.1	778217	778915	+	protein_coding	CPF_RS03250	CPF_0648	.	WP_003456978.	L-ribulose-5-ph	-26.248800	4.049250	-25.025047	4.029400	-1.048901	7.007894	7.042418	2.328238	6.973539	7.042418	2.328238	6.973539	108.335663	5.800520	115.481855	1602	82	2283
gene654	NC_008261.1	778934	779782	+	protein_coding	CPF_RS03255	CPF_0649	.	WP_003456954.	L-ribulose-5-ph	-21.133256	3.966608	-24.519848	4.029242	1.160249	6.843304	6.736926	2.335483	6.951361	6.736926	2.335483	6.951361	85.186318	5.824016	113.028293	1530	100	2714
gene655	NC_008261.1	779906	780706	-	protein_coding	CPF_RS03260	CPF_0650	.	WP_011590294.	tetratricopeptid	-1.498616	5.799170	-1.333547	5.718558	-1.123782	6.013553	6.098323	5.514692	5.929961	6.098323	5.514692	5.929961	52.699325	65.680945	52.529039	893	1064	1190
gene656	NC_008261.1	781031	782341	-	protein_coding	CPF_RS03265	CPF_0652	.	WP_011590296.	carbohydrate-bi	-3.482629	1.960778	-4.217204	2.047397	1.210926	3.192661	3.057589	1.257413	3.333699	3.057589	1.257413	3.333699	5.516643	2.036673	7.983145	153	54	296
gene657	NC_008261.1	782631	782957	-	protein_coding	CPF_RS03270	CPF_0653	.	WP_011590297.	HesB-like prote	3.381885	10.072523	6.203447	9.583165	-1.834317	8.783366	9.231883	10.989710	8.356640	9.231883	10.989710	8.356640	516.501244	2471.236493	329.789710	3573	16343	3050
gene658	NC_008261.1	783059	783403	-	protein_coding	CPF_RS03275	CPF_0654	.	WP_003453095.	HesB-like prote	2.229684	10.103724	4.559519	9.541684	-2.044917	9.011063	9.541847	10.698687	8.509805	9.541847	10.698687	8.509805	670.138665	1957.770972	360.034459	4891	13660	3513
gene659	NC_008261.1	783761	784492	+	protein_coding	CPF_RS03280	.	.	WP_041702627.	hypothetical pro	1.620392	4.513435	2.523291	4.154051	-1.557210	3.845944	4.178673	4.875016	3.539709	4.178673	4.875016	3.539709	12.979892	41.475112	9.274172	201	614	192
gene660	NC_008261.1	785000	786013	+	protein_coding	CPF_RS03285	CPF_0656	.	WP_003457081.	hypothetical pro	-1.834659	8.929451	-1.658023	8.859646	-1.106534	9.304620	9.377930	8.502418	9.231883	9.377930	8.502418	9.231883	579.034866	430.530420	606.208127	12421	8829	17385
gene661	NC_008261.1	786159	788609	-	protein_coding	CPF_RS03290	CPF_0657	.	WP_003457020.	leucine--tRNA	3.757499	7.297118	4.386512	7.168777	-1.167402	6.291803	6.404444	8.314217	6.181143	6.404444	8.314217	6.181143	67.558957	364.580291	62.896748	3503	18072	4360
gene663	NC_008261.1	789763	791019	+	protein_coding	CPF_RS03300	CPF_0661	.	WP_011590299.	chloride channe	3.044996	2.859408	2.407616	3.074856	1.264735	2.330126	2.166860	3.773300	2.505695	2.166860	3.773300	2.505695	2.594776	19.078171	4.191177	69	485	149
gene664	NC_008261.1	791089	791655	-	protein_coding	CPF_RS03305	CPF_0662	.	WP_003456967.	NUDIX domain-	1.023283	4.341779	1.224880	4.209551	-1.197010	4.193485	4.325209	4.358413	4.065773	4.325209	4.358413	4.065773	14.422786	29.562892	13.282553	173	339	213
gene665	NC_008261.1	791947	792309	+	protein_coding	CPF_RS03310	CPF_0663	.	WP_003456924.	hypothetical pro	-1.806793	8.322873	-1.588870	8.234319	-1.137156	8.667309	8.760520	7.907089	8.575090	8.760520	7.907089	8.575090	370.998311	289.456022	374.324949	2849	2125	3843
gene666	NC_008261.1	792506	793492	+	protein_coding	CPF_RS03315	CPF_0664	.	WP_003457103.	biotin--[acetyl-	2.305140	4.000786	2.027630	4.106885	1.136864	3.534779	3.443460	4.648315	3.628520	3.443460	4.648315	3.628520	7.519148	35.068014	9.815636	157	700	274
gene667	NC_008261.1	793649	794734	+	protein_coding	CPF_RS03320	CPF_0665	.	WP_003457087.	aldolase	2.425417	2.408812	1.608702	2.767153	1.507685	2.128703	1.853040	3.131273	2.445375	1.853040	3.131273	2.445375	1.958704	11.382574	4.004611	45	250	123
gene668	NC_008261.1	795004	795738	+	protein_coding	CPF_RS03325	CPF_0667	.	WP_003456936.	DUF4184 domai	1.102439	4.281374	-1.013673	4.362087	1.117513	4.291000	4.211603	4.352302	4.371894	4.211603	4.352302	4.371894	13.248478	29.465720	16.981357	206	438	353
gene669	NC_008261.1	795928	796233	+	protein_coding	CPF_RS03330	CPF_0668	.	WP_003457108.	hypothetical pro	1.413078	3.071707	2.362020	2.639382	-1.671542	2.433753	2.832396	3.331237	2.091216	2.832396	3.331237	2.091216	4.634319	13.411796	3.004256	30	83	26
gene670	NC_008261.1	796480	797130	+	protein_coding	CPF_RS03335	CPF_0669	.	WP_003457021.	aminotransferas	-2.145749	4.449769	-1.661535	4.283610	-1.291425	4.846470	5.034462	3.932981	4.665498	5.034462	3.932981	4.665498	25.050951	21.722776	20.964829	345	286	386
gene671	NC_008261.1	797635	798945	+	protein_coding	CPF_RS03340	CPF_0670	.	WP_003456962.	phosphomethylp	7.817555	10.632271	11.288175	10.323229	-1.443952	8.982969	9.251889	12.218607	8.721866	9.251889	12.218607	8.721866	523.540198	5539.336227	411.428654	14520	146869	15255
gene672	NC_008261.1	799183	802983	+	protein_coding	CPF_RS03345	CPF_0671	.	WP_011590300.	phosphoribosylfo	20.361074	8.271325	27.405647	7.988552	-1.345982	6.160295	6.378353	10.726094	5.949693	6.378353	10.726094	5.949693	66.409391	1994.903330	53.162253	5340	153352	5715
gene673	NC_008261.1	803294	803773	+	protein_coding	CPF_RS03350	CPF_0672	.	WP_003453078.	5-(carboxyamino	22.678419	8.215526	28.902666	7.982893	-1.274457	6.089412	6.266806	10.770114	5.916983	6.266806	10.770114	5.916983	61.451072	2104.232055	52.300053	624	20427	710
gene674	NC_008261.1	803773	804480	+	protein_coding	CPF_RS03355	CPF_0673	.	WP_003456948.	phosphoribosyla	20.856040	7.913366	28.972569	7.595284	-1.389169	5.777962	6.019936	10.402330	5.545714	6.019936	10.402330	5.545714	50.007445	1524.093025	39.602713	749	21823	793
gene675	NC_008261.1	804502	805947	+	protein_coding	CPF_RS03360	CPF_0674	.	WP_011009969.	amidophosphorib	16.567032	8.711443	24.092324	8.364402	-1.454233	6.642992	6.918611	10.968854	6.378353	6.918611	10.968854	6.378353	97.907203	2465.695343	72.769543	2995	72107	2976
gene676	NC_008261.1	805948	806949	+	protein_coding	CPF_RS03365	CPF_0675	.	WP_003468449.	phosphoribosylfo	18.034840	8.290307	27.717838	7.882581	-1.536905	6.144618	6.462448	10.635163	5.842420	6.462448	10.635163	5.842420	69.112406	1816.075837	49.260962	1465	36802	1396
gene677	NC_008261.1	806937	807551	+	protein_coding	CPF_RS03370	CPF_0676	.	WP_003468450.	phosphoribosylg	20.528576	7.750047	28.775098	7.421572	-1.401641	5.622117	5.870949	10.230581	5.383832	5.870949	10.230581	5.383832	43.888132	1305.532543	35.472767	571	16238	617
gene678	NC_008261.1	807564	809069	+	protein_coding	CPF_RS03375	CPF_0677	.	WP_011590302.	bifunctional pho	16.653867	8.284568	22.240733	8.014212	-1.335470	6.288361	6.500496	10.558281	6.083149	6.500496	10.558281	6.083149	71.218961	1692.684822	58.577468	2269	51555	2495
gene679	NC_008261.1	809185	810435	+	protein_coding	CPF_RS03380	CPF_0678	.	WP_011590303.	phosphoribosyla	15.470305	8.296051	19.036381	8.104389	-1.230511	6.400974	6.552351	10.503781	6.253093	6.552351	10.503781	6.253093	73.342268	1633.019007	65.825959	1941	41316	2329
gene680	NC_008261.1	811348	811710	+	protein_coding	CPF_RS03390	CPF_0680	.	WP_003456972.	YccF domain-c	1.916158	5.044391	2.839320	4.722877	-1.481777	4.304046	4.597048	5.535265	4.029719	4.597048	5.535265	4.029719	18.230875	66.745153	12.954780	140	490	133
gene682	NC_008261.1	812243	812785	+	protein_coding	CPF_RS03395	CPF_0681	.	WP_003453016.	NADH peroxidas	-2.476084	14.285744	-2.476084	14.285744	1.000000	14.954737	14.954737	13.646677	14.954737	14.954737	13.646677	14.954737	37596.146881	13151.698625	38400.895455	431874	144428	589734
gene683	NC_008261.1	812918	814318	-	protein_coding	CPF_RS03400	CPF_0682	.	WP_011590304.	argininosuccinat	1.275899	7.581896	-1.707195	8.136361	2.178208	7.949937	7.408175	7.759689	8.531318	7.408175	7.759689	8.531318	137.187754	265.652594	364.278534	4066	7527	14434
gene684	NC_008261.1	814412	815623	-	protein_coding	CPF_RS03405	CPF_0683	.	WP_011590305.	argininosuccinat	1.644034	7.918732	-1.355430	8.502013	2.228373	8.125692	7.568228	8.285468	8.724219	7.568228	8.285468	8.724219	154.563720	357.789222	411.923995	3963	8770	14120
gene685	NC_008261.1	816248	816895	+	protein_coding	CPF_RS03410	CPF_0684	.	WP_011590306.	transaldolase	-5.844127	8.617615	-2.863795	8.161461	-2.040693	9.456180	9.984698	7.437710	8.955638	9.984698	7.437710	8.955638	872.745276	221.056745	488.352082	11964	2897	8950
gene686	NC_008261.1	817295	822355	+	protein_coding	CPF_RS03415	CPF_0685	.	WP_011590307.	hypothetical pro	-2.203311	1.123917	-2.208949	1.125049	1.002559	1.831800	1.829957	0.690284	1.833644	1.829957	0.690284	1.833644	1.924053	0.898839	2.284528	206	92	327
gene687	NC_008261.1	822827	823840	+	protein_coding	CPF_RS03420	CPF_0687	.	WP_003457101.	3-deoxy-7-pho	-1.262729	7.234366	-1.257481	7.231430	-1.004174	7.401590	7.404595	7.068050	7.398587	7.404595	7.068050	7.398587	137.148424	173.743333	159.842281	2942	3563	4584
gene688	NC_008261.1	823961	825013	+	protein_coding	CPF_RS03425	CPF_0688	.	WP_011590308.	3-dehydroquina	-2.196213	5.056205	-2.577259	5.158354	1.173502	5.769718	5.655462	4.520445	5.886282	5.655462	4.520445	5.886282	38.516341	32.212618	50.938039	858	686	1517
gene689	NC_008261.1	825206	826480	+	protein_coding	CPF_RS03430	CPF_0689	.	WP_011590309.	3-phosphoshikin	-2.160185	5.218478	-2.635071	5.345818	1.219836	5.945163	5.803547	4.692392	6.090234	5.803547	4.692392	6.090234	42.302065	36.143983	58.652321	1141	932	2115
gene690	NC_008261.1	826544	827617	+	protein_coding	CPF_RS03435	CPF_0690	.	WP_011590310.	chorismate synt	-1.881346	5.443290	-2.000282	5.483810	1.063219	5.962285	5.918230	5.006465	6.006668	5.918230	5.006465	6.006668	45.509532	44.841998	55.242419	1034	974	1678
gene691	NC_008261.1	827786	828079	+	protein_coding	CPF_RS03440	CPF_0691	.	WP_011590311.	chorismate mut	-1.280745	6.032755	-1.655655	6.209963	1.292728	6.396415	6.213887	5.856904	6.584306	6.213887	5.856904	6.584306	58.364048	84.764399	84.786518	363	504	705
gene692	NC_008261.1	828901	829716	+	protein_coding	CPF_RS03445	CPF_0693	.	WP_011590313.	shikimate dehyd	-1.177537	4.691473	-1.012636	4.584116	-1.162844	4.700752	4.810840	4.575067	4.593183	4.810840	4.575067	4.593183	21.317868	33.327506	19.932083	368	550	460
gene693	NC_008261.1	829970	830473	+	protein_coding	CPF_RS03450	CPF_0694	.	WP_003476990.	shikimate kinase	-1.083044	5.500632	-1.178092	5.560356	1.087760	5.618832	5.558479	5.443387	5.679840	5.558479	5.443387	5.679840	36.578019	61.905481	43.706147	390	631	623
gene694	NC_008261.1	830540	830974	+	protein_coding	CPF_RS03455	CPF_0695	.	WP_011590314.	3-dehydroquina	-1.370206	6.964555	-1.203195	6.873204	-1.138806	7.101076	7.195456	6.741064	7.007934	7.195456	6.741064	7.007934	121.598142	145.041305	117.696709	1119	1276	1448
gene695	NC_008261.1	830995	831336	-	protein_coding	CPF_RS03460	CPF_0696	.	WP_003457038.	transcriptional r	-1.007558	3.863576	1.283152	3.673903	-1.292850	3.679071	3.869011	3.858148	3.498456	3.869011	3.858148	3.498456	10.366240	20.385590	8.891138	75	141	86
gene696	NC_008261.1	831592	832698	+	protein_coding	CPF_RS03465	CPF_0697	.	WP_004459980.	oxidoreductase	-2.395959	4.719051	-2.298219	4.692679	-1.042529	5.361132	5.391259	4.130656	5.331172	5.391259	4.130656	5.331172	32.111185	24.968616	34.395577	752	559	1077
gene697	NC_008261.1	833435	833950	+	protein_coding	CPF_RS03470	CPF_0698	.	WP_011590315.	hypothetical pro	-1.725125	2.876923	-1.259379	2.671520	-1.369822	3.061642	3.297039	2.510339	2.843051	3.297039	2.510339	2.843051	6.779039	6.707777	5.481827	74	70	80
gene698	NC_008261.1	834077	834409	+	protein_coding	CPF_RS03475	CPF_0699	.	WP_011590316.	PadR family tran	-1.790104	1.460935	-2.313341	1.594144	1.292294	2.117039	1.940137	1.100093	2.310071	1.940137	1.100093	2.310071	2.129282	1.633348	3.610104	15	11	34
gene699	NC_008261.1	834411	835268	+	protein_coding	CPF_RS03480	CPF_0700	.	WP_011590317.	hypothetical pro	1.005398	1.009704	-1.286531	1.181441	1.293475	1.176906	1.005829	1.013595	1.377081	1.005829	1.013595	1.377081	0.771306	1.440731	1.401124	14	25	34
gene700	NC_008261.1	835480	835974	+	protein_coding	CPF_RS03485	CPF_0701	.	WP_011590318.	hypothetical pro	-1.092980	0.935482	-1.360225	1.072753	1.244510	1.148815	1.001812	0.873545	1.317390	1.001812	0.873545	1.317390	0.763960	1.198688	1.285738	8	12	18
gene702	NC_008261.1	836447	837337	+	protein_coding	CPF_RS03495	CPF_0703	.	WP_011590320.	DUF4162 domai	-1.166681	6.123667	-1.217776	6.153955	1.043795	6.266724	6.235881	6.013472	6.297720	6.235881	6.013472	6.297720	59.737466	93.120336	68.413935	1126	1678	1724
gene703	NC_008261.1	837339	838550	+	protein_coding	CPF_RS03500	CPF_0704	.	WP_011590321.	ABC transporte	-1.266249	5.517265	-1.397261	5.585703	1.103465	5.760755	5.690172	5.349612	5.832213	5.690172	5.349612	5.832213	39.352711	58.094852	48.923268	1009	1424	1677
gene704	NC_008261.1	838665	839654	+	protein_coding	CPF_RS03505	CPF_0705	.	WP_003461725.	choloylglycine h	-3.231075	8.792384	-2.325507	8.574173	-1.389406	9.438784	9.678999	7.986985	9.204530	9.678999	7.986985	9.204530	729.009293	299.422391	588.582069	15268	5995	16480
gene705	NC_008261.1	840440	840922	+	protein_coding	CPF_RS03510	CPF_0706	.	WP_003453052.	Lrp/AsnC family	2.226194	5.534827	2.197215	5.545305	1.013189	4.997004	4.987561	6.142141	5.006465	4.987561	6.142141	5.006465	24.075432	100.734503	27.451694	246	984	375
gene706	NC_008261.1	840919	842097	+	protein_coding	CPF_RS03515	CPF_0707	.	WP_003471875.	aminotransferas	2.541616	5.593962	2.189104	5.714107	1.161030	5.067971	4.961413	6.307159	5.176818	4.961413	6.307159	5.176818	23.614981	109.586204	30.889326	589	2613	1030
gene707	NC_008261.1	842212	843237	+	protein_coding	CPF_RS03520	CPF_0708	.	WP_003471891.	hypothetical pro	-4.688395	2.228452	-2.735842	1.973792	-1.713693	3.194074	3.606175	1.377081	2.829066	3.606175	1.377081	2.829066	8.431209	2.265066	5.444961	183	47	158
gene708	NC_008261.1	843294	843758	-	protein_coding	CPF_RS03525	CPF_0709	.	WP_003477004.	hypothetical pro	1.493932	0.738151	1.142152	0.981923	1.307998	0.669587	0.503356	1.082470	0.890716	0.503356	1.082470	0.890716	0.304968	1.595029	0.760382	3	15	10
gene709	NC_008261.1	843817	844959	-	protein_coding	CPF_RS03530	CPF_0710	.	WP_011590323.	galactosyldiacylg	-2.646945	7.380380	-2.179258	7.251722	-1.214609	7.974391	8.115870	6.711542	7.835379	8.115870	6.711542	7.835379	240.444532	142.670670	224.272896	5814	3298	7250
gene710	NC_008261.1	845450	845953	+	protein_coding	CPF_RS03535	CPF_0711	.	WP_011590324.	N-acetyltransfe	-5.863653	6.795027	-4.739877	6.666472	-1.237090	8.034736	8.189677	5.637877	7.882727	8.189677	5.637877	7.882727	249.574636	71.912389	230.527127	2661	733	3286
gene711	NC_008261.1	846174	846692	+	protein_coding	CPF_RS03540	CPF_0712	.	WP_041702629.	RNA polymerase	-1.361229	1.736375	-1.250436	1.681617	-1.088604	1.910802	1.973022	1.528112	1.850543	1.973022	1.528112	1.850543	2.185898	2.762873	2.316309	24	29	34
gene712	NC_008261.1	846769	848181	+	protein_coding	CPF_RS03545	CPF_0713	.	WP_011590326.	DUF4179 domai	1.066857	1.400536	-1.098154	1.514031	1.171573	1.464406	1.354630	1.447997	1.583077	1.354630	1.447997	1.583077	1.204333	2.519536	1.726601	36	72	69
gene713	NC_008261.1	848402	849604	+	protein_coding	CPF_RS03550	CPF_0714	.	WP_004458224.	hypothetical pro	1.302886	4.114257	-1.088881	4.370528	1.418687	4.172485	3.927826	4.309537	4.432383	3.927826	4.309537	4.432383	10.805707	28.607104	17.634804	275	696	600
gene714	NC_008261.1	849659	850663	+	protein_coding	CPF_RS03555	CPF_0715	.	WP_003457045.	capsular polysac	1.569393	3.180009	1.013107	3.512281	1.549089	3.171515	2.871480	3.521687	3.502900	2.871480	3.521687	3.502900	4.891628	15.989967	8.901014	104	325	253
gene715	NC_008261.1	850815	851990	+	protein_coding	CPF_RS03560	CPF_0716	.	WP_003453008.	AI-2E family trar	1.078901	5.546426	-1.222717	5.744692	1.319190	5.688233	5.491915	5.601477	5.891568	5.491915	5.601477	5.891568	34.447651	69.964266	51.142506	857	1664	1701
gene716	NC_008261.1	852189	853028	+	protein_coding	CPF_RS03565	CPF_0717	.	WP_003456958.	zinc transporter	2.072473	4.972623	2.239694	4.910026	-1.080686	4.418326	4.474655	5.526009	4.362707	4.474655	5.526009	4.362707	16.600793	66.045594	16.794946	295	1122	399
gene717	NC_008261.1	853216	853980	+	protein_coding	CPF_RS03570	CPF_0718	.	WP_011590328.	DeoR/GlpR tran	-1.255144	4.150571	-1.255144	4.150571	1.000000	4.317733	4.317733	3.989881	4.317733	4.317733	3.989881	4.317733	14.335494	22.816212	16.361640	232	353	354
gene718	NC_008261.1	854099	855887	+	protein_coding	CPF_RS03575	CPF_0719	.	WP_011590329.	HAD family hydr	-1.289357	5.075454	-1.223482	5.038834	-1.053842	5.224123	5.262089	4.895438	5.186431	5.262089	4.895438	5.186431	28.817359	42.301625	31.010881	481	675	692
gene719	NC_008261.1	855091	856113	+	protein_coding	CPF_RS03580	CPF_0720	.	WP_011590330.	spore photoproc	-1.219973	0.285396	-1.135170	0.251315	-1.074705	0.407562	0.462832	0.175983	0.358892	0.462832	0.175983	0.358892	0.277244	0.193337	0.241940	6	4	7
gene720	NC_008261.1	856777	858861	+	protein_coding	CPF_RS03585	CPF_0721	.	WP_011590331.	exo-alpha-sialid	-61.506508	8.941710	-38.520006	8.694758	-1.596742	12.051515	12.393807	6.451140	11.718676	12.393807	6.451140	11.718676	5087.074097	120.330213	3281.015633	224382	5074	193477
gene721	NC_008261.1	858909	860063	+	protein_coding	CPF_RS03590	CPF_0722	.	WP_003468923.	cyclically-permu	-290.744710	7.920905	-216.369704	7.790037	-1.343740	12.792260	13.007163	4.823553	12.580908	13.007163	4.823553	12.580908	8333.280978	39.942012	5698.113212	203616	933	186135
gene722	NC_008261.1	860407	861234	+	protein_coding	CPF_RS03595	CPF_0723	.	WP_011590332.	oxidoreductase	-2.674758	0.813849	-2.110920	0.732079	-1.267106	1.609732	1.789533	0.370125	1.447997	1.789533	0.370125	1.447997	1.883951	0.418021	1.494592	33	7	35
gene723	NC_008261.1	861338	861802	+	protein_coding	CPF_RS03600	CPF_0724	.	WP_011590333.	N-acetyltransfe	1.275779	1.886332	1.434058	1.791355	-1.124065	1.632265	1.718806	2.070185	1.550081	1.718806	2.070185	1.550081	1.728153	4.572417	1.672841	17	43	22
gene724	NC_008261.1	862176	862352	+	protein_coding	CPF_RS14730	CPF_0725	.	WP_003452895.	hypothetical pro	1.947457	2.523815	1.293155	2.858543	1.505973	2.365388	2.088408	3.050000	2.679104	2.088408	3.050000	2.679104	2.403562	10.615504	4.994037	9	38	25
gene725	NC_008261.1	862480	863163	+	protein_coding	CPF_RS03605	CPF_0726	.	WP_011590334.	ATPase	1.028937	5.294318	1.062217	5.271216	-1.032344	5.250768	5.273781	5.314935	5.227857	5.273781	5.314935	5.227857	29.094581	56.819411	32.049452	421	786	620
gene726	NC_008261.1	863377	864273	-	protein_coding	CPF_RS03610	CPF_0727	.	WP_003457094.	LysR family tran	1.288767	2.240174	1.354385	2.200962	-1.050915	2.028501	2.064640	2.430631	1.992994	2.064640	2.430631	1.992994	2.371407	6.284094	2.717829	45	114	69
gene727	NC_008261.1	864623	866323	+	protein_coding	CPF_RS03615	CPF_0728	.	WP_003472186.	pyridine nucleot	1.098792	7.124015	1.767132	6.769146	-1.608250	6.704880	7.056380	7.192299	6.370889	7.056380	7.192299	6.370889	109.407529	189.644355	72.607133	3937	6524	3493
gene728	NC_008261.1	866429	867715	+	protein_coding	CPF_RS03620	CPF_0729	.	WP_011590335.	YeeE/YedE fami	1.122535	6.023371	1.820771	5.658567	-1.622017	5.580778	5.940568	6.107329	5.242779	5.940568	6.107329	5.242779	46.682393	98.661282	32.418168	1271	2568	1180
gene729	NC_008261.1	867804	869114	+	protein_coding	CPF_RS03625	CPF_0730	.	WP_011590336.	APC family perm	1.301190	1.671119	1.463699	1.573160	-1.124893	1.404503	1.491960	1.871792	1.322172	1.491960	1.871792	1.322172	1.370417	3.884766	1.294564	38	103	48
gene730	NC_008261.1	869254	870372	+	protein_coding	CPF_RS03630	CPF_0731	.	WP_011590337.	ROK family tran	-1.447113	5.104362	-1.080962	4.900567	-1.338727	5.163191	5.377908	4.844730	4.957046	5.377908	4.844730	4.957046	31.851315	40.829322	26.131265	754	924	827
gene731	NC_008261.1	870578	871624	+	protein_coding	CPF_RS03635	CPF_0732	.	WP_011590338.	NAD(P)-depend	-4.861645	3.966911	-5.060244	4.011552	1.085506	5.288667	5.229814	3.008975	5.348182	5.229814	3.008975	5.348182	28.307855	10.342552	34.749912	627	219	1029
gene732	NC_008261.1	871747	873144	+	protein_coding	CPF_RS03640	CPF_0733	.	WP_011590339.	MFS transporte	-5.198917	2.342002	-7.157598	2.479522	1.376748	4.039744	3.815690	1.437478	4.276954	3.815690	1.437478	4.276954	10.008538	2.475832	15.883181	296	70	628
gene733	NC_008261.1	873165	874097	+	protein_coding	CPF_RS03645	CPF_0734	.	WP_003461370.	carbohydrate kir	-4.852049	2.597132	-5.930533	2.690059	1.222274	4.117571	3.975332	1.696738	4.264899	3.975332	1.696738	4.264899	11.247537	3.338791	15.689305	222	63	414
gene734	NC_008261.1	874183	874845	-	protein_coding	CPF_RS03650	CPF_0735	.	WP_011590340.	elongation facto	1.016037	2.264021	1.247701	2.109860	-1.228007	2.099192	2.252573	2.275527	1.956255	2.252573	2.275527	1.956255	2.780591	5.518848	2.613169	39	74	49
gene736	NC_008261.1	875747	876061	+	protein_coding	CPF_RS03660	CPF_0737	.	WP_011590341.	thioredoxin	1.418604	1.213792	1.057402	1.451140	1.341594	1.180583	0.987488	1.491960	1.411436	0.987488	1.491960	1.411436	0.750318	2.668509	1.459210	5	17	13
gene737	NC_008261.1	876369	876701	+	protein_coding	CPF_RS03665	CPF_0738	.	WP_011590342.	transcriptional r	-1.057924	0.618352	-1.014868	0.589617	-1.042424	0.629618	0.660302	0.579067	0.600360	0.660302	0.579067	0.600360	0.425856	0.742431	0.424718	3	5	4
gene738	NC_008261.1	876727	877938	+	protein_coding	CPF_RS03670	CPF_0739	.	WP_011590343.	cytochrome c b	1.074262	1.974253	-1.006887	2.031547	1.081661	1.979070	1.923256	2.026602	2.036504	1.923256	2.026602	2.036504	2.106092	4.406070	2.829789	54	108	97
gene739	NC_008261.1	877464	880185	+	protein_coding	CPF_RS03675	CPF_0740	.	WP_011590344.	lipase	-3.404600	4.815055	-4.951538	5.035143	1.454367	6.043384	5.779226	4.011740	6.319617	5.779226	4.011740	6.319617	41.725020	23.057524	69.668382	1520	803	3393
gene740	NC_008261.1	880647	882098	+	protein_coding	CPF_RS03680	CPF_0741	.	WP_003457086.	carbon starvatio	-35.005850	5.754786	-33.999885	5.741116	-1.029587	8.844146	8.865204	3.735680	8.823138	8.865204	3.735680	8.823138	397.628411	18.559239	440.876476	12214	545	18105
gene741	NC_008261.1	882229	882648	+	protein_coding	CPF_RS03685	CPF_0742	.	WP_003456940.	hypothetical pro	-71.200462	6.292768	-66.036516	6.258776	-1.078198	10.027182	10.081641	3.927826	9.973018	10.081641	3.927826	9.973018	943.937980	21.662013	1070.246388	8387	184	12713
gene742	NC_008261.1	882852	883154	+	protein_coding	CPF_RS03690	CPF_0743	.	WP_003457097.	hypothetical pro	1.210604	5.978694	1.759639	5.695939	-1.453522	5.566110	5.842420	6.118147	5.302867	5.842420	6.118147	5.302867	43.369885	99.054987	34.074166	278	607	292
gene743	NC_008261.1	883242	883796	-	protein_coding	CPF_RS03695	CPF_0744	.	WP_003457091.	TetR/AcrR fami	-1.279872	1.891288	-1.125610	1.804982	-1.137048	1.982836	2.077646	1.721646	1.892352	2.077646	1.721646	1.892352	2.384796	3.385485	2.420893	28	38	38
gene744	NC_008261.1	884193	884894	+	protein_coding	CPF_RS03700	CPF_0745	.	WP_003457044.	ABC transporte	1.992824	1.954802	-2.645323	3.138738	5.271664	2.440091	1.519686	2.514501	3.917945	1.519686	2.514501	3.917945	1.414061	6.761832	12.088131	21	96	240
gene745	NC_008261.1	884911	888309	+	protein_coding	CPF_RS03705	CPF_0746	.	WP_011590345.	ABC transporte	1.660393	1.784853	-2.732564	2.821134	4.537132	2.301637	1.456182	2.187707	3.637962	1.456182	2.187707	3.637962	1.321170	5.149705	9.882287	95	354	950
gene746	NC_008261.1	888715	889053	+	protein_coding	CPF_RS03710	CPF_0747	.	WP_011590346.	bacteriocin imm	-1.003370	4.027292	-1.702571	4.391981	1.696853	4.394628	4.029719	4.024866	4.792581	4.029719	4.024866	4.792581	11.712934	23.483156	23.050353	84	161	221
gene747	NC_008261.1	889155	890606	-	protein_coding	CPF_RS03715	CPF_0748	.	WP_011590347.	cardiolipin synth	-1.155166	5.482303	1.107310	5.305201	-1.279127	5.406846	5.587340	5.379240	5.232181	5.587340	5.379240	5.232181	37.210519	59.151190	32.240843	1143	1737	1324
gene748	NC_008261.1	891054	891548	+	protein_coding	CPF_RS03720	CPF_0749	.	WP_011590348.	NUDIX domain-	2.402052	4.081028	5.199972	3.368825	-2.164804	2.887183	3.497561	4.761829	2.383325	3.497561	4.761829	2.383325	7.735100	38.657703	3.857213	81	387	54
gene749	NC_008261.1	891712	892974	-	protein_coding	CPF_RS03725	CPF_0750	.	WP_011590349.	cytosine deamin	1.037703	2.895270	-1.168353	3.032252	1.212403	3.004421	2.868697	2.922090	3.146566	2.868697	2.922090	3.146566	4.865485	9.630793	6.970774	130	246	249
gene750	NC_008261.1	893422	894177	+	protein_coding	CPF_RS03730	CPF_0751	.	WP_003453056.	creatininase	-1.396703	6.073824	-1.290856	6.018939	-1.081998	6.262510	6.319617	5.837591	6.205919	6.319617	5.837591	6.205919	63.589479	83.521711	64.027518	1017	1277	1369
gene751	NC_008261.1	894226	895152	+	protein_coding	CPF_RS03735	CPF_0752	.	WP_003457034.	EamA family trar	1.046132	5.942281	1.099853	5.905848	-1.051352	5.873603	5.909837	5.974903	5.837591	5.909837	5.974903	5.837591	45.230355	91.157543	49.088960	887	1709	1287
gene752	NC_008261.1	895279	896478	-	protein_coding	CPF_RS03740	CPF_0753	.	WP_011590350.	PucR family trar	3.776579	3.509082	3.748118	3.516725	1.008131	2.684940	2.679104	4.596184	2.690788	2.679104	4.596184	2.690788	4.017955	34.200080	5.009019	102	830	170
gene753	NC_008261.1	896667	897077	+	protein_coding	CPF_RS03745	CPF_0754	.	WP_003477074.	peptide deformy	4.811308	3.707503	4.848436	3.700002	-1.007717	2.738057	2.743608	5.010037	2.732518	2.743608	5.010037	2.732518	4.255465	44.874259	5.161720	37	373	60
gene754	NC_008261.1	897410	898675	-	protein_coding	CPF_RS03750	CPF_0756	.	WP_003461701.	proton glutamat	41.140627	4.236626	45.996023	4.087850	-1.118019	2.250632	2.332543	7.695035	2.171597	2.332543	7.695035	2.171597	2.987049	258.673137	3.183876	80	6623	114
gene755	NC_008261.1	899251	899943	-	protein_coding	CPF_RS03755	CPF_0757	.	WP_011590352.	noncanonical py	-1.239370	5.916533	-1.205272	5.896896	-1.028290	6.053204	6.073361	5.763754	6.033113	6.073361	5.763754	6.033113	51.771965	78.770957	56.378570	759	1104	1105
gene756	NC_008261.1	900169	901122	+	protein_coding	CPF_RS03760	CPF_0758	.	WP_011590353.	hypothetical pro	-1.226118	2.502248	-1.074371	2.410707	-1.141242	2.556535	2.653614	2.359516	2.463009	2.653614	2.359516	2.463009	3.963946	5.960460	4.076893	80	115	110
gene757	NC_008261.1	901357	901986	-	protein_coding	CPF_RS03765	CPF_0759	.	WP_011590354.	N-acetyltransfe	-1.054224	0.337403	1.158046	0.164498	-1.220840	0.184113	0.377637	0.301456	0.089763	0.377637	0.301456	0.089763	0.225096	0.313942	0.056123	3	4	1
gene758	NC_008261.1	902138	902941	+	protein_coding	CPF_RS03770	CPF_0760	.	WP_011590355.	MerR family DNA	1.788873	2.912452	1.502347	3.054349	1.190719	2.645908	2.522986	3.362037	2.774819	2.522986	3.362037	2.774819	3.645203	13.837472	5.277281	62	225	120
gene759	NC_008261.1	903078	904433	-	protein_coding	CPF_RS03775	CPF_0761	.	WP_011590356.	DUF4317 domai	1.217810	4.681431	1.244566	4.665242	-1.021971	4.525739	4.541444	4.825732	4.510089	4.541444	4.825732	4.510089	17.673981	39.965122	18.800119	507	1096	721
gene760	NC_008261.1	904740	904895	+	protein_coding	CPF_RS03780	CPF_0762	.	WP_003452954.	cytochrome c5	3.166303	13.216622	2.182326	13.499480	1.450885	12.676970	12.411346	14.074145	12.948279	12.411346	14.074145	12.948279	5097.590616	15802.402299	7105.100970	16823	49856	31348
gene761	NC_008261.1	904953	905447	-	protein_coding	CPF_RS03785	CPF_0763	.	WP_003460841.	DUF2798 domai	-1.201395	1.326280	1.036659	1.174254	-1.245438	1.297271	1.465224	1.200513	1.148571	1.465224	1.200513	1.148571	1.336931	1.897923	1.071448	14	19	15
gene762	NC_008261.1	905799	907163	+	protein_coding	CPF_RS03790	CPF_0764	.	WP_003468848.	MFS transporte	-3.941717	2.588669	-14.325645	3.165212	3.634367	4.598299	3.760719	1.781895	5.622423	3.760719	1.781895	5.622423	9.627161	3.549962	41.807663	278	98	1614
gene763	NC_008261.1	907386	908225	-	protein_coding	CPF_RS03795	CPF_0765	.	WP_003468880.	AraC family tran	-3.687394	4.390102	-5.704228	4.637519	1.546954	5.737273	5.431183	3.548582	6.060613	5.431183	3.548582	6.060613	33.257860	16.187646	57.540581	591	275	1367
gene764	NC_008261.1	908589	911627	+	protein_coding	CPF_RS03800	CPF_0766	.	WP_011590357.	beta-galactosida	-2.155975	3.052945	-7.077923	3.700189	3.282934	4.432312	3.657004	2.548664	5.371990	3.657004	2.548664	5.371990	8.803834	6.898671	35.113454	566	424	3018
gene765	NC_008261.1	912042	913481	+	protein_coding	CPF_RS03805	CPF_0767	.	WP_011590358.	FAD-dependent	1.413227	1.821908	1.059011	2.046631	1.334478	1.785462	1.589415	2.088408	2.005690	1.589415	2.088408	2.005690	1.510016	4.669890	2.774604	46	136	113
gene766	NC_008261.1	913833	915212	-	protein_coding	CPF_RS03810	CPF_0768	.	WP_003476373.	GntP family perm	1.948404	3.050651	2.341091	2.891531	-1.201543	2.471224	2.607215	3.569508	2.342326	2.607215	3.569508	2.342326	3.870664	16.517797	3.740751	113	461	146
gene767	NC_008261.1	915626	916405	+	protein_coding	CPF_RS03815	CPF_0769	.	WP_011590359.	alpha/beta hydr	-2.201397	3.369793	-1.643400	3.186577	-1.339538	3.769979	3.986739	2.848320	3.565004	3.986739	2.848320	3.565004	11.393295	9.001689	9.383411	188	142	207
gene768	NC_008261.1	916586	917875	+	protein_coding	CPF_RS03820	CPF_0770	.	WP_011590360.	aspartate--tRN	1.146266	0.764633	-1.234916	1.010223	1.415542	0.888467	0.672476	0.869418	1.173831	0.672476	0.869418	1.173831	0.439721	1.188235	1.096365	12	31	40
gene769	NC_008261.1	918285	919934	+	protein_coding	CPF_RS03825	CPF_0771	.	WP_011590361.	isopeptide-form	2.042343	8.172117	2.437799	8.034988	-1.193629	7.544465	7.673223	8.703448	7.417868	7.673223	8.703448	7.417868	166.419242	498.684370	162.924374	5809	16641	7603
gene770	NC_008261.1	920024	920863	+	protein_coding	CPF_RS03830	CPF_0772	.	WP_011590362.	class C sortase	1.184316	5.288149	1.302416	5.217509	-1.099720	5.098501	5.167530	5.411584	5.030394	5.167530	5.411584	5.030394	27.180282	60.747819	27.949485	483	1032	664
gene771	NC_008261.1	920872	921732	+	protein_coding	CPF_RS03835	CPF_0773	.	WP_011590363.	class C sortase	-1.084556	4.132433	1.167315	3.961127	-1.266019	4.017650	4.191400	4.074296	3.851102	4.191400	4.074296	3.851102	13.066519	24.349665	11.580598	238	424	282
gene772	NC_008261.1	921885	924671	+	protein_coding	CPF_RS03840	CPF_0774	.	WP_011590364.	VWA domain-co	1.217736	4.322054	1.280384	4.284494	-1.051446	4.145944	4.182289	4.466490	4.109914	4.182289	4.466490	4.109914	13.009018	31.012355	13.904603	767	1748	1096
gene774	NC_008261.1	926776	927588	+	protein_coding	CPF_RS03850	CPF_0776	.	WP_011590366.	DUF438 domain	-2.415615	5.481744	1.846514	4.417844	-4.460469	4.960220	6.154733	4.882343	3.997538	6.154733	4.882343	3.997538	55.235612	41.904335	12.655737	950	689	291
gene775	NC_008261.1	927631	928329	+	protein_coding	CPF_RS03855	CPF_0777	.	WP_011590367.	iron-sulfur clust	-1.219802	5.388208	1.874257	4.772164	-2.286222	4.900789	5.533437	5.246791	4.340472	5.533437	5.246791	4.340472	35.638511	54.185349	16.591348	527	766	328
gene776	NC_008261.1	928482	929192	+	protein_coding	CPF_RS03860	CPF_0778	.	WP_003468866.	Crp/Fnr family t	1.075986	4.516625	1.247317	4.407467	-1.159231	4.356216	4.464104	4.569763	4.250935	4.464104	4.569763	4.250935	16.554492	33.172565	15.565381	249	477	313
gene777	NC_008261.1	929445	929606	-	protein_coding	CPF_RS03865	CPF_0779	.	WP_003453030.	rubredoxin	-1.460151	0.498073	-1.013512	0.304481	-1.440684	0.514162	0.841070	0.294953	0.314317	0.841070	0.294953	0.314317	0.583581	0.305222	0.218258	2	1	1
gene778	NC_008261.1	930188	930934	+	protein_coding	CPF_RS03870	CPF_0780	.	WP_003477104.	flavohemoprotei	1.062825	7.111866	2.192381	6.565331	-2.062785	6.524882	7.068050	7.155954	6.023456	7.068050	7.155954	6.023456	110.486635	186.928005	55.947655	1746	2824	1182
gene779	NC_008261.1	931153	931863	+	protein_coding	CPF_RS03875	CPF_0781	.	WP_003461384.	Crp/Fnr family t	-2.380212	4.590415	-2.003804	4.480703	-1.187847	5.132710	5.258386	4.007296	5.010037	5.258386	4.007296	5.010037	28.721046	23.019118	27.550227	432	331	554
gene780	NC_008261.1	931918	932079	-	protein_coding	CPF_RS03880	CPF_0782	.	WP_003452884.	Rubredoxin-2	-2.021638	3.991188	-1.283093	3.691030	-1.575598	4.190356	4.531120	3.515595	3.875220	4.531120	3.515595	3.875220	17.507428	15.871523	11.785927	60	52	54
gene781	NC_008261.1	932106	933326	-	protein_coding	CPF_RS03885	CPF_0783	.	WP_003453014.	FprA family A-ty	-1.035587	4.762532	1.775434	4.303413	-1.838617	4.326266	4.787824	4.737375	3.909203	4.787824	4.737375	3.909203	20.983104	37.863977	12.075508	542	935	417
gene782	NC_008261.1	933542	934105	+	protein_coding	CPF_RS03890	CPF_0784	.	WP_003453100.	peroxiredoxin	3.571445	8.903655	7.790396	8.256539	-2.181301	7.448817	8.032627	9.869135	6.907438	8.032627	9.869135	6.907438	229.729115	1013.816277	109.709430	2741	11564	1750
gene783	NC_008261.1	934592	936262	+	protein_coding	CPF_RS03895	CPF_0785	.	WP_011590368.	FAD-binding pro	2.624240	3.870663	2.913844	3.779284	-1.110357	3.160367	3.236781	4.628681	3.085757	3.236781	4.628681	3.085757	6.421486	34.828141	6.644131	227	1177	314
gene784	NC_008261.1	936752	937810	+	protein_coding	CPF_RS03900	CPF_0787	.	WP_003480523.	PTS sugar trans	3.271339	5.033790	2.968974	5.115962	1.101841	4.320327	4.250935	5.960817	4.390852	4.250935	5.960817	4.390852	13.569496	90.487397	17.161379	304	1938	514
gene785	NC_008261.1	937846	938775	+	protein_coding	CPF_RS03905	CPF_0788	.	WP_011590370.	2-dehydropanto	2.827668	6.861464	2.858895	6.910795	1.063475	6.196738	6.152504	7.652117	6.241290	6.152504	7.652117	6.241290	55.199226	252.599428	65.582983	1086	4751	1725
gene786	NC_008261.1	939036	939461	+	protein_coding	CPF_RS03910	CPF_0789	.	WP_003461590.	MarR family tran	1.405457	5.467867	1.437297	5.450939	-1.022655	5.211672	5.227857	5.718896	5.195537	5.227857	5.718896	5.195537	28.295455	76.258112	31.373807	255	657	378
gene787	NC_008261.1	939680	940183	+	protein_coding	CPF_RS03915	CPF_0790	.	WP_011590371.	hypothetical pro	1.837119	4.993506	1.591343	5.105355	1.154446	4.676472	4.574020	5.451465	4.781220	4.574020	5.451465	4.781220	18.007640	62.396016	22.659848	192	636	323
gene788	NC_008261.1	940286	940729	+	protein_coding	CPF_RS03920	CPF_0791	.	WP_003480535.	MarR family tran	-1.010369	4.853527	-1.041212	4.875136	1.030526	4.882616	4.860974	4.846092	4.904355	4.860974	4.846092	4.904355	22.144530	40.870822	25.084913	208	367	315
gene789	NC_008261.1	940814	941695	+	protein_coding	CPF_RS03925	CPF_0792	.	WP_011590372.	pirin family prote	-2.984302	4.330534	-3.029349	4.339541	1.015095	5.201261	5.190465	3.613071	5.212079	5.190465	3.613071	5.212079	27.547402	17.154700	31.669669	514	306	790
gene790	NC_008261.1	941729	942325	+	protein_coding	CPF_RS03930	CPF_0793	.	WP_004460381.	FMN-dependent	-2.110842	5.288643	-2.249507	5.329938	1.065692	5.900655	5.854939	4.777120	5.946729	5.854939	4.777120	5.946729	43.706986	38.927257	52.947834	552	470	894
gene791	NC_008261.1	942572	943087	+	protein_coding	CPF_RS03935	CPF_0794	.	WP_003466481.	NUDIX domain-	-1.595318	4.229316	-1.545094	4.207953	-1.032506	4.556504	4.579638	3.905793	4.533488	4.579638	3.905793	4.533488	18.138509	21.273236	19.117871	198	222	279
gene792	NC_008261.1	943237	943818	-	protein_coding	CPF_RS03940	CPF_0795	.	WP_011590373.	phosphatase PA	2.852476	5.915943	2.776721	5.937958	1.027282	5.227339	5.207959	6.720173	5.246791	5.207959	6.720173	5.246791	27.858469	143.410100	32.563181	343	1688	536
gene793	NC_008261.1	944042	944782	+	protein_coding	CPF_RS03945	CPF_0796	.	WP_011590374.	hypothetical pro	-1.453462	3.907630	-1.469256	3.914900	1.010866	4.194465	4.186676	3.647183	4.202268	4.186676	3.647183	4.202268	13.013618	17.549624	15.030636	204	263	315
gene794	NC_008261.1	944953	946197	+	protein_coding	CPF_RS03950	CPF_0797	.	WP_011590375.	hypothetical pro	1.051413	4.565522	-1.040733	4.630541	1.094241	4.594007	4.529501	4.601831	4.659431	4.529501	4.601831	4.659431	17.465241	34.433409	20.845471	460	867	734
gene795	NC_008261.1	946332	946859	-	protein_coding	CPF_RS03955	CPF_0798	.	WP_011590376.	hypothetical pro	-1.832148	1.755996	-1.340150	1.569786	-1.367121	2.008068	2.246268	1.372732	1.795127	2.246268	1.372732	1.795127	2.775325	2.247541	2.142896	31	24	32
gene796	NC_008261.1	947377	947844	+	protein_coding	CPF_RS03960	CPF_0799	.	WP_003455849.	hypothetical pro	2.287102	5.727874	2.022839	5.825322	1.130640	5.249939	5.162117	6.355637	5.339256	5.162117	6.355637	5.339256	27.069177	112.626771	34.526723	268	1066	457
gene797	NC_008261.1	947972	948685	+	protein_coding	CPF_RS03965	CPF_0800	.	WP_004456177.	M48 family pepti	1.208018	1.417405	1.092203	1.495293	1.106039	1.358381	1.287624	1.560266	1.433026	1.287624	1.560266	1.433026	1.059273	2.908582	1.485621	16	42	30
gene798	NC_008261.1	948869	950743	+	protein_coding	CPF_RS03970	CPF_0801	.	WP_003455757.	PHP domain-co	1.463166	5.955009	1.085030	6.176734	1.348503	5.898526	5.686788	6.235881	6.118147	5.686788	6.235881	6.118147	39.328686	105.220874	60.400520	1560	3990	3203
gene799	NC_008261.1	951039	952871	+	protein_coding	CPF_RS03975	CPF_0802	.	WP_011590377.	alpha-glycosidas	-3.766371	6.125120	-3.709368	6.115696	-1.015367	7.144945	7.155954	5.242779	7.133953	7.155954	5.242779	7.133953	118.832741	54.112643	127.079688	4608	2006	6588
gene800	NC_008261.1	953116	954399	+	protein_coding	CPF_RS03980	CPF_0803	.	WP_011590378.	serine dehydrata	-18.346870	7.952262	-18.012443	8.006541	1.101889	10.288917	10.219165	6.188223	10.359144	10.219165	6.188223	10.359144	1022.491051	102.550179	1387.214663	27774	2663	50376
gene801	NC_008261.1	954688	955191	-	protein_coding	CPF_RS03985	CPF_0804	.	WP_003449220.	hypothetical pro	2.020688	0.988215	2.321546	0.807788	-1.148899	0.493276	0.603454	1.618300	0.403215	0.603454	1.618300	0.403215	0.375159	3.041315	0.280617	4	31	4
gene802	NC_008261.1	955511	956056	+	protein_coding	CPF_RS03990	CPF_0805	.	WP_011590379.	hypothetical pro	-2.381469	7.011090	-2.034482	6.906403	-1.170553	7.550451	7.664901	6.413049	7.437710	7.664901	6.413049	7.437710	165.531768	118.000013	165.834485	1912	1303	2561
gene803	NC_008261.1	956096	956716	-	protein_coding	CPF_RS03995	CPF_0806	.	WP_011590380.	YdcF family prot	2.710217	6.758442	3.868781	6.510986	-1.353685	5.854877	6.077397	7.515805	5.640505	6.077397	7.515805	5.640505	51.913329	232.101122	42.361014	682	2915	744
gene804	NC_008261.1	956980	957924	+	protein_coding	CPF_RS04000	CPF_0807	.	WP_003477145.	ferrichrome AB	-1.307371	2.507164	-1.464296	2.581761	1.120030	2.788513	2.707942	2.321273	2.871480	2.707942	2.321273	2.871480	4.151761	5.755607	5.537515	83	110	148

gene805	NC_008261.1	958023	959036	+	protein_coding	CPF_RS04005	CPF_0808	.	WP_003455759.	iron ABC transp	-1.274499	0.502269	-1.426532	0.557046	1.119289	0.783924	0.706836	0.356906	0.869418	0.706836	0.356906	0.869418	0.466174	0.390106	0.732262	10	8	21
gene806	NC_008261.1	959040	960053	+	protein_coding	CPF_RS04010	CPF_0809	.	WP_003455696.	iron ABC transp	1.026256	0.264105	-1.012550	0.292320	1.039136	0.272359	0.246071	0.283462	0.301456	0.246071	0.283462	0.301456	0.139852	0.292579	0.209218	3	6	6
gene807	NC_008261.1	960211	960993	+	protein_coding	CPF_RS04015	CPF_0810	fecE	WP_011590381.	ABC transporter	1.097637	1.034890	-1.071533	1.153031	1.176153	1.080587	0.969869	1.104269	1.203945	0.969869	1.104269	1.203945	0.724445	1.641882	1.128920	12	26	25
gene808	NC_008261.1	961312	961800	+	protein_coding	CPF_RS04020	CPF_0811	.	WP_011590382.	GNAT family N-a	-1.486533	2.168212	-1.362627	2.112451	-1.090932	2.409367	2.472966	1.901014	2.347405	2.472966	1.901014	2.347405	3.480004	3.943538	3.759928	36	39	52
gene809	NC_008261.1	961868	962083	+	protein_coding	CPF_RS04025	CPF_0812	.	WP_011590383.	DUF4177 domai	-1.239481	2.026931	-1.449118	2.128806	1.169133	2.297663	2.187707	1.877970	2.413146	2.187707	1.877970	2.413146	2.626114	3.891575	3.928642	12	17	24
gene810	NC_008261.1	962076	962393	+	protein_coding	CPF_RS04030	CPF_0813	.	WP_011590384.	hypothetical pro	-1.078454	0.196741	-1.023880	0.165815	-1.053301	0.217973	0.258628	0.149663	0.183710	0.258628	0.149663	0.183710	0.148648	0.155490	0.111188	1	1	1
gene811	NC_008261.1	962605	962931	-	protein_coding	CPF_RS04035	CPF_0814	.	WP_003449181.	hypothetical pro	-1.778981	0.705460	-1.978079	0.747921	1.111917	1.308554	1.234265	0.403215	1.387315	1.234265	0.403215	1.387315	1.011897	0.453632	1.405661	7	3	13
gene812	NC_008261.1	963334	966717	+	protein_coding	CPF_RS04040	CPF_0815	.	WP_011590385.	endo-beta-N-a	-5.067493	11.071356	-5.804301	11.159125	1.145399	12.401248	12.303709	9.962437	12.499559	12.303709	9.962437	12.499559	4595.336611	1072.788987	5359.598218	328974	73420	512953
gene813	NC_008261.1	966918	967826	+	protein_coding	CPF_RS04045	CPF_0816	.	WP_011590386.	D-alanine--D-a	-3.064755	3.655498	-3.727779	3.767249	1.216338	4.690739	4.551593	2.935822	4.834138	4.551593	2.935822	4.834138	17.784773	9.736871	23.649650	342	179	608
gene814	NC_008261.1	967973	968800	+	protein_coding	CPF_RS04050	CPF_0817	.	WP_004456191.	Cof-type HAD-I	-1.317533	4.093684	-1.199218	4.028511	-1.098660	4.229017	4.297434	3.899595	4.161689	4.297434	3.899595	4.161689	14.215270	21.139913	14.518896	249	354	340
gene815	NC_008261.1	969127	970107	+	protein_coding	CPF_RS04055	CPF_0818	.	WP_003455841.	PTS mannose tr	-7.199324	11.417118	-4.077934	11.049137	-1.765434	12.512775	12.929502	10.081641	12.109479	12.929502	10.081641	12.109479	7556.559198	1153.183213	4177.228201	156822	22879	115897
gene816	NC_008261.1	970158	970961	+	protein_coding	CPF_RS04060	CPF_0819	.	WP_003455721.	PTS mannose/f	-7.099210	11.529936	-4.359431	11.214357	-1.628472	12.673486	13.030126	10.202467	12.326607	13.030126	10.202467	12.326607	8454.872558	1276.306902	4842.784774	143806	20753	110120
gene817	NC_008261.1	970981	971892	+	protein_coding	CPF_RS04065	CPF_0820	.	WP_011590387.	PTS mannose tr	-7.096034	11.680458	-4.466362	11.380630	-1.588773	12.840882	13.179181	10.352168	12.511268	13.179181	10.352168	12.511268	9484.954238	1450.033529	5372.056700	182997	26745	138564
gene818	NC_008261.1	972063	972437	+	protein_coding	CPF_RS04070	CPF_0821	.	WP_003449204.	DUF956 domain	-2.904051	7.002404	-1.909065	6.725752	-1.521190	7.504842	7.813540	6.275473	7.208339	7.813540	6.275473	7.208339	186.180990	107.726133	134.076709	1477	817	1422
gene819	NC_008261.1	972821	973168	+	protein_coding	CPF_RS04075	CPF_0822	.	WP_011590388.	hypothetical pro	-1.707432	8.011945	-1.281114	7.811975	-1.332772	8.197315	8.407148	7.635320	7.992718	8.407148	7.635320	7.992718	287.287835	251.918332	245.675623	2115	1773	2418
gene820	NC_008261.1	973394	974311	+	protein_coding	CPF_RS04080	CPF_0823	.	WP_003455717.	polysaccharide c	-1.463501	7.268918	-1.999159	7.482425	1.366011	7.770547	7.548819	6.999395	7.998788	7.548819	6.999395	7.998788	152.366114	166.758719	245.771250	2959	3096	6381
gene821	NC_008261.1	974770	975552	+	protein_coding	CPF_RS04085	CPF_0824	.	WP_011590389.	TIGR02206 fami	1.947792	3.260967	1.544535	3.449347	1.261086	2.977955	2.815319	3.777159	3.149986	2.815319	3.777159	3.149986	4.588153	19.134237	6.999306	76	303	155
gene822	NC_008261.1	975809	976750	+	protein_coding	CPF_RS04090	CPF_0825	.	WP_011590390.	hypothetical pro	1.405139	5.380451	1.210864	5.491646	1.160444	5.246926	5.140686	5.631399	5.355362	5.140686	5.631399	5.355362	26.595679	71.386861	34.869830	530	1360	929
gene823	NC_008261.1	977025	979439	+	protein_coding	CPF_RS04095	CPF_0826	.	WP_004456165.	hypothetical pro	-4.249350	5.546248	-5.036132	5.646964	1.185153	6.808637	6.687203	4.599960	6.932277	6.687203	4.599960	6.932277	81.288836	34.335724	112.178603	4153	1677	7662
gene824	NC_008261.1	980233	981186	+	protein_coding	CPF_RS04100	CPF_0827	.	WP_003455787.	serine protease	1.011115	3.164860	1.252252	3.006206	-1.238486	2.998642	3.156897	3.172844	2.848320	3.156897	3.172844	2.848320	6.045018	11.661769	5.485274	122	225	148
gene825	NC_008261.1	981513	983324	+	protein_coding	CPF_RS04105	CPF_0829	.	WP_003455858.	ABC transporter	-1.217023	3.007039	-1.558696	3.172753	1.280745	3.325758	3.152053	2.868697	3.509036	3.152053	2.868697	3.509036	6.026149	9.168776	8.995551	231	336	461
gene826	NC_008261.1	983324	985114	+	protein_coding	CPF_RS04110	CPF_0830	.	WP_003455695.	ABC transporter	-1.191547	3.222023	-1.539669	3.395152	1.292160	3.530975	3.350920	3.098084	3.720704	3.350920	3.098084	3.720704	6.994173	11.070802	10.482961	265	401	531
gene827	NC_008261.1	985331	985828	+	protein_coding	CPF_RS04115	CPF_0831	.	WP_003455803.	hypothetical pro	-1.298384	3.521156	-1.824764	3.746651	1.405411	3.952429	3.714549	3.337832	4.205542	3.714549	3.337832	4.205542	9.207220	13.503298	15.051907	97	136	212
gene828	NC_008261.1	985837	987465	+	protein_coding	CPF_RS04120	CPF_0832	.	WP_011590392.	membrane prote	1.093583	2.991772	-1.083609	3.114383	1.185016	3.047932	2.927937	3.057000	3.172844	2.927937	3.057000	3.172844	5.078121	10.654089	7.119308	175	351	328
gene829	NC_008261.1	987834	988118	+	protein_coding	CPF_RS04125	CPF_0833	.	WP_003449190.	ArsR family tran	2.005744	0.900746	1.131832	1.441194	1.772123	0.846643	0.529152	1.533290	1.354630	0.529152	1.533290	1.354630	0.331720	2.775910	1.364686	2	16	11
gene830	NC_008261.1	988237	988635	+	protein_coding	CPF_RS04130	CPF_0834	.	WP_003455733.	arsenate reduct	1.708235	3.341990	1.300977	3.555630	1.313040	3.168355	2.977984	3.750490	3.370895	2.977984	3.750490	3.370895	5.212738	18.836533	8.152672	44	152	92
gene831	NC_008261.1	988652	989968	+	protein_coding	CPF_RS04135	CPF_0835	.	WP_011590393.	MATE family effl	1.895553	2.824192	1.587012	2.971152	1.194416	2.525213	2.400310	3.322929	2.656616	2.400310	3.322929	2.656616	3.194408	13.290697	4.859346	89	354	181
gene832	NC_008261.1	990160	990828	+	protein_coding	CPF_RS04140	CPF_0836	.	WP_003449171.	DNA-binding res	-1.407449	4.523865	-1.222625	4.426654	-1.151169	4.674467	4.777120	4.284037	4.574020	4.777120	4.284037	4.574020	20.844045	28.233682	19.713681	295	382	373
gene833	NC_008261.1	990825	991835	+	protein_coding	CPF_RS04145	CPF_0837	.	WP_011590394.	ATP-binding pro	-1.040413	3.937678	1.055250	3.870216	-1.097896	3.898407	3.966360	3.909203	3.831618	3.966360	3.909203	3.831618	11.174622	21.274942	11.471170	239	435	328
gene834	NC_008261.1	992119	992829	+	protein_coding	CPF_RS04150	CPF_0838	.	WP_011590395.	Fic family protei	1.884891	2.103836	1.747803	2.170363	1.078434	1.749331	1.695710	2.610191	1.804648	1.695710	2.610191	1.804648	1.662098	7.371681	2.188105	25	106	44
gene836	NC_008261.1	993403	994971	+	protein_coding	CPF_RS04155	CPF_0840	.	WP_011590397.	clostripain	3.515347	11.285518	1.833426	11.783345	1.917365	10.874488	10.415059	12.228726	11.354184	10.415059	12.228726	11.354184	1170.302925	5558.361980	2545.692701	38845	176376	112965
gene837	NC_008261.1	995242	996009	+	protein_coding	CPF_RS04160	CPF_0841	.	WP_011590398.	ABC transporter	5.266987	3.253272	2.074016	4.105701	2.539512	2.862923	2.268520	4.665498	3.613071	2.268520	4.665498	3.613071	2.831279	35.603623	9.668144	46	553	210
gene839	NC_008261.1	998295	999833	+	protein_coding	CPF_RS04170	CPF_0843	.	WP_041702632.	glycoside hydrol	-1.922232	3.554699	-2.548550	3.728690	1.325828	4.255797	4.057210	3.114427	4.464104	4.057210	3.114427	4.464104	11.917337	11.245006	18.172843	388	350	791
gene840	NC_008261.1	1000085	1000387	-	protein_coding	CPF_RS04175	CPF_0844	.	WP_011590400.	stress responsiv	-1.696086	6.536361	-1.639006	6.513023	-1.034826	6.903828	6.928566	6.166357	6.879178	6.928566	6.166357	6.879178	98.596286	101.339616	106.656808	632	621	914
gene841	NC_008261.1	1000650	1001792	+	protein_coding	CPF_RS04180	CPF_0845	.	WP_011590401.	aminotransferas	18.563049	5.855543	6.019028	6.915401	3.084061	4.860963	4.115969	8.330331	5.740800	4.115969	8.330331	5.740800	12.448195	371.211953	45.597000	301	8581	1474
gene842	NC_008261.1	1001957	1002874	+	protein_coding	CPF_RS04185	CPF_0846	.	WP_003477206.	hypothetical pro	1.022873	0.595157	1.259377	0.413102	-1.231215	0.401934	0.579067	0.611694	0.278985	0.579067	0.611694	0.278985	0.360447	0.807940	0.192581	7	15	5
gene843	NC_008261.1	1003079	1003618	+	protein_coding	CPF_RS04190	CPF_0847	.	WP_003477208.	rubrerythrin fam	1.984659	1.432515	1.844434	1.504849	1.076026	1.072555	1.021000	2.009891	1.126713	1.021000	2.009891	1.126713	0.787834	4.303625	1.047638	9	47	16
gene844	NC_008261.1	1004144	1007212	+	protein_coding	CPF_RS04195	CPF_0848	.	WP_011590402.	alpha-mannosid	-6.299760	3.906096	-11.444242	4.203248	1.816616	5.868054	5.453207	2.797910	6.314460	5.453207	2.797910	6.314460	33.669710	8.603490	69.425218	2186	534	6026
gene845	NC_008261.1	1007469	1008026	-	protein_coding	CPF_RS04200	CPF_0849	.	WP_003480605.	N-acetyltransfe	-1.408253	7.660842	-1.191288	7.543073	-1.182126	7.790148	7.911775	7.417868	7.670391	7.911775	7.417868	7.670391	203.481491	218.607587	196.178662	2402	2467	3096
gene846	NC_008261.1	1008266	1009438	+	protein_coding	CPF_RS04205	CPF_0850	.	WP_003455707.	NADH-depende	-2.788417	8.062429	-2.076666	7.866061	-1.342738	8.620807	8.836016	7.356570	8.410839	8.836016	7.356570	8.410839	388.879822	211.862822	344.685625	9650	5026	11435
gene847	NC_008261.1	1009752	1010813	+	protein_coding	CPF_RS04210	CPF_0851	.	WP_003449241.	carbohydrate dia	-2.487955	3.947573	-2.342263	3.910521	-1.062201	4.615697	4.659431	3.344471	4.572374	4.659431	3.344471	4.572374	19.228497	13.595294	19.709799	432	292	592
gene848	NC_008261.1	1010960	1012219	+	protein_coding	CPF_RS04215	CPF_0853	.	WP_003465956.	GntP family perm	-7.560274	3.221815	-3.992820	2.909728	-1.893467	4.512129	4.996084	2.077646	4.075054	4.996084	2.077646	4.075054	24.235282	4.630648	13.469631	646	118	480
gene849	NC_008261.1	1012443	1013564	+	protein_coding	CPF_RS04220	CPF_0854	.	WP_004456183.	glycerate kinase	-5.243344	4.343662	-3.125541	4.049318	-1.677580	5.314074	5.700352	3.309865	4.953971	5.700352	3.309865	4.953971	39.602363	13.176759	26.092909	940	299	828
gene850	NC_008261.1	1014223	1015659	+	protein_coding	CPF_RS04225	CPF_0855	.	WP_003455781.	RNA helicase	1.506417	4.935033	1.888825	4.758631	-1.253852	4.482161	4.648315	5.239436	4.321947	4.648315	5.239436	4.321947	19.144866	53.815857	16.411719	582	1564	667
gene851	NC_008261.1	1015814	1016272	+	protein_coding	CPF_RS04230	CPF_0856	.	WP_003454082.	hypothetical pro	-5.251873	5.585692	-6.473101	5.706319	1.232532	7.058004	6.908803	4.515971	7.210428	6.908803	4.515971	7.210428	96.908762	32.102131	134.190101	941	298	1742
gene852	NC_008261.1	1016461	1017837	+	protein_coding	CPF_RS04235	CPF_0857	.	WP_011590405.	MATE family effl	-1.058463	2.286886	-1.093676	2.309958	1.033268	2.351727	2.328238	2.246268	2.375453	2.328238	2.246268	2.375453	2.986561	5.386264	3.825933	87	150	149
gene853	NC_008261.1	1017944	1018117	+	protein_coding	CPF_RS14740	CPF_0858	.	WP_003454088.	hypothetical pro	-1.583040	1.295474	-1.541150	1.280366	-1.027181	1.649068	1.668526	1.005829	1.629836	1.668526	1.005829	1.629836	1.630002	1.420859	1.828851	6	5	9
gene854	NC_008261.1	1018458	1024772	+	protein_coding	CPF_RS04240	CPF_0859	.	WP_041702633.	alpha-N-acetylg	-12.473607	8.080332	-12.663334	8.089036	1.015210	10.114138	10.103255	6.462448	10.125034	10.103255	6.462448	10.125034	959.952643	122.013531	1191.156925	128244	15583	212744
gene855	NC_008261.1	1025033	1025212	+	protein_coding	CPF_RS04245	CPF_0860	.	WP_003455738.	hypothetical pro	-1.069406	1.398754	1.084305	1.291483	-1.159562	1.336948	1.447997	1.351187	1.234417	1.447997	1.351187	1.234417	1.313057	2.197596	1.178593	5	8	6
gene856	NC_008261.1	1025324	1026484	-	protein_coding	CPF_RS04250	CPF_0861	.	WP_011590407.	alanine racemas	3.054749	4.671242	2.988360	4.690021	1.022216	3.950478	3.934660	5.545714	3.966360	3.934660	5.545714	3.966360	10.830176	66.907414	12.425474	266	1571	408
gene857	NC_008261.1	1027032	1027601	+	protein_coding	CPF_RS04255	CPF_0862	.	WP_011590408.	N-acetyltransfe	-1.321331	3.662504	-1.212095	3.603099	-1.090122	3.806257	3.869011	3.467019	3.744521	3.869011	3.467019	3.744521	10.366240	15.354253	10.607335	125	177	171
gene858	NC_008261.1	1027653	1028162	-	protein_coding	CPF_RS04260	.		WP_003453795.	ATP-binding pro	-2.079765	1.971292	-1.329269	1.705626	-1.564592	2.222820	2.569043	1.512623	1.923256	2.569043	1.512623	1.923256	3.800142	2.714677	2.495843	41	28	36
gene859	NC_008261.1	1029042	1029605	-	protein_coding	CPF_RS04265	CPF_0865	.	WP_003455702.	accessory gene	-1.097844	2.522254	1.693616	2.040674	-1.859327	2.095881	2.590490	2.455816	1.695710	2.590490	2.455816	1.695710	3.855359	6.399913	1.943424	46	73	31
gene860	NC_008261.1	1029967	1031742	+	protein_coding	CPF_RS04270	CPF_0866	.	WP_011590410.	aminopeptidase	-1.329671	6.983740	-1.282127	6.958189	-1.037083	7.165985	7.192299	6.781229	7.139768	7.192299	6.781229	7.139768	121.422292	147.753034	127.136710	4562	5307	6386
gene861	NC_008261.1	1031888	1032124	+	protein_coding	CPF_RS04275	.		WP_003455735.	hypothetical pro	1.141520	1.597380	1.493770	1.376113	-1.308579	1.296316	1.504753	1.695710	1.116752	1.504753	1.695710	1.116752	1.396162	3.338120	1.044323	7	16	7
gene862	NC_008261.1	1032227	1033354	+	protein_coding	CPF_RS04280	CPF_0868	.	WP_011590412.	aminopeptidase	-3.078942	8.103920	-2.394956	7.938243	-1.285595	8.772549	8.955638	7.333204	8.593203	8.955638	7.333204	8.593203	420.192236	208.698517	379.688665	10027	4761	12113
gene863	NC_008261.1	1033458	1033901	+	protein_coding	CPF_RS04285	CPF_0869	.	WP_003474296.	N-acetyltransfe	-2.022846	5.880779	-2.200951	5.936353	1.088046	6.471473	6.410889	5.394502	6.532629	6.410889	5.394502	6.532629	67.817624	60.025540	82.342222	637	539	1034
gene864	NC_008261.1	1034084	1034749	-	protein_coding	CPF_RS04290	CPF_0870	.	WP_003455751.	membrane prote	-1.804972	6.416351	1.246846	5.843180	-2.250521	6.243978	6.856465	6.004488	5.686206	6.856465	6.004488	5.686206	92.907661	92.506893	43.958324	1309	1246	828
gene865	NC_008261.1	1034911	1036083	-	protein_coding	CPF_RS04295	CPF_0871	.	WP_011590413.	transposase	-1.083666	5.484184	1.864771	4.956691	-2.020788	5.009353	5.542450	5.426530	4.527532	5.542450	5.426530	4.527532	35.865600	61.248842	19.020256	890	1453	631
gene867	NC_008261.1	1036945	1037487	+	protein_coding	CPF_RS04305	CPF_0873	.	WP_003455744.	N-acetyltransfe	-1.738539	3.666020	-1.430829	3.537724	-1.215057	3.943586	4.086600	3.288725	3.805576	4.086600	3.288725	3.805576	12.187491	12.930604	11.134771	140	142	171
gene869	NC_008261.1	1038706	1042596	+	protein_coding	CPF_RS04315	CPF_0875	.	WP_011590415.	hyaluronidase	-5.673333	2.802895	-6.205700	2.844545	1.093837	4.386169	4.321947	1.817751	4.451344	4.321947	1.817751	4.451344	14.408195	3.672543	17.928785	1186	289	1973
gene870	NC_008261.1	1042917	1043237	-	protein_coding	CPF_RS04320	CPF_0876	.	WP_003453892.	hypothetical pro	-2.357227	9.924834	-3.066926	10.101647	1.301074	10.750811	10.562636	9.325545	10.942338	10.562636	9.325545	10.942338	1311.780842	739.531971	1945.559203	8908	4801	17663
gene871	NC_008261.1	1043676	1043864	+	protein_coding	CPF_RS04325	CPF_0877	.	WP_003455852.	hypothetical pro	1.270160	7.230033	-1.105136	7.476359	1.403699	7.300105	7.059585	7.404595	7.548819	7.059585	7.404595	7.548819	109.796583	216.881749	179.408005	439	829	959
gene872	NC_008261.1	1043918	1044733	+	protein_coding	CPF_RS04330	CPF_0878	.	WP_041702634.	ABC transporter	1.104377	6.467159	-1.128697	6.625926	1.246508	6.552958	6.395939	6.539172	6.713831	6.395939	6.539172	6.713831	67.197627	129.977272	94.590733	1160	2145	2183
gene873	NC_008261.1	1044730	1046379	+	protein_coding	CPF_RS04335	CPF_0879	.	WP_011590417.	hypothetical pro	1.068679	6.291885	-1.199607	6.469931	1.281995	6.420849	6.244153	6.339981	6.602544	6.244153	6.339981	6.602544	60.247834	111.777700	86.508707	2103	3730	4037
gene874	NC_008261.1	1046533	1047318	-	protein_coding	CPF_RS04340	CPF_0880	.	WP_011590418.	LysM peptidogly	-1.341783	1.549951	-1.313355	1.536415	-1.021645	1.760954	1.776469	1.352317	1.745575	1.776469	1.352317	1.745575	1.864341	2.201789	2.024300	31	35	45
gene875	NC_008261.1	1047643	1048194	-	protein_coding	CPF_RS04345	CPF_0881	.	WP_011590419.	class I SAM-dep	1.469174	0.496255	1.379093	0.568773	1.065318	0.333606	0.291072	0.846077	0.382356	0.291072	0.846077	0.382356	0.171268	1.164487	0.256216	2	13	4
gene876	NC_008261.1	1048515	1049165	+	protein_coding	CPF_RS04350	CPF_0882	.	WP_011590420.	CoA pyrophosph	1.953154	2.030197	1.953154	2.030197	1.000000	1.603936	1.603936	2.569742	1.603936	1.603936	2.569742	1.603936	1.524840	7.063700	1.792330	21	93	33
gene877	NC_008261.1	1049256	1049675	+	protein_coding	CPF_RS04355	CPF_0883	.	WP_011590421.	hypothetical pro	2.384522	3.849984	1.966190	4.010284	1.212762	3.410143	3.273832	4.527532	3.552129	3.273832	4.527532	3.552129	6.640317	32.257563	9.344557	59	274	111
gene878	NC_008261.1	1050043	1050669	+	protein_coding	CPF_RS04360	CPF_0884	.	WP_011590422.	DedA family prot	4.638105	3.469289	5.167066	3.360972	-1.114047	2.455644	2.534785	4.748320	2.378975	2.534785	4.748320	2.378975	3.694151	38.168764	3.834656	49	484	68
gene879	NC_008261.1	1050937	1052067	+	protein_coding	CPF_RS04365	CPF_0885	.	WP_003455712.	alcohol dehydrog	-34.692986	5.637606	-31.393491	5.590962	-1.105101	8.676813	8.749201	3.632629	8.605023	8.749201	3.632629	8.605023	370.302995	17.356341	382.214185	8860	397	12226
gene880	NC_008261.1	1052421	1052771	+	protein_coding	CPF_RS04370	CPF_0886	.	WP_003453843.	ethanolamine ut	-5.309962	9.424927	-3.620650	9.178531	-1.466577	10.426030	10.705915	8.297213	10.153462	10.705915	8.297213	10.153462	1403.287674	360.349318	1217.576956	10420	2558	12087
gene881	NC_008261.1	1052775	1053209	+	protein_coding	CPF_RS04375	CPF_0887	.	WP_011590423.	ethanolamine ut	-7.987419	8.369789	-6.349677	8.230109	-1.257925	9.834888	10.001803	7.004074	9.670757	10.001803	7.004074	9.670757	879.440358	167.320377	819.894132	8093	1472	10087
gene882	NC_008261.1	1053227	1053802	+	protein_coding	CPF_RS04380	CPF_0888	.	WP_003453802.	ANTAR domain-	-8.281260	7.677230	-7.054144	7.581665	-1.173957	9.235723	9.352138	6.302287	9.120758	9.352138	6.302287	9.120758	567.404793	109.193028	559.279084	6914	1272	9111
gene883	NC_008261.1	1053806	1055233	+	protein_coding	CPF_RS04385	CPF_0889	.	WP_003455831.	sensor histidine	-6.914368	7.645195	-5.870473	7.546083	-1.177821	9.047356	9.166187	6.376589	8.930066	9.166187	6.376589	8.930066	492.992248	114.438864	483.074459	14893	3305	19510
gene884	NC_008261.1	1055449	1056882	+	protein_coding	CPF_RS04390	CPF_0890	.	WP_011590424.	ethanolamine am	-163.954885	7.040376	-99.682984	6.819473	-1.644763	11.257394	11.622054	4.264899	10.904175	11.622054	4.264899	10.904175	2778.978188	27.826249	1878.721201	84304	807	76195
gene885	NC_008261.1	1056901	1058268	+	protein_coding	CPF_RS04395	CPF_0891	.	WP_004457163.	ethanolamine am	-217.448986	7.433452	-113.759283	7.144678	-1.911483	11.791854	12.268458	4.503925	11.333765	12.268458	4.503925	11.333765	4522.997899	32.060316	2519.655511	130896	887	97486
gene886	NC_008261.1	1058285	1059172	+	protein_coding	CPF_RS04400	CPF_0892	.	WP_003454028.	ethanolamine am	-211.959803	7.295849	-107.599423	6.995249	-1.969897	11.620295	12.119642	4.391995	11.141522	12.119642	4.391995	11.141522	3920.273879	30.012770	2304.586787	73645	539	57879
gene887	NC_008261.1	1059194	1059847	+	protein_coding	CPF_RS04405	CPF_0893	.	WP_003461990.	ethanolamine ut	-240.743617	7.342834	-123.413445	7.049129	-1.950708	11.804381	12.296216	4.384862	11.332218	12.296216	4.384862	11.332218	4595.025199	29.939719	2517.268638	63574	396	46561
gene888	NC_008261.1	1059860	1060624	+	protein_coding	CPF_RS04410	CPF_0894	.	WP_011590425.	BMC domain-co	-233.144677	6.429704	-119.374218	6.153062	-1.953057	10.976032	11.469515	3.604433	10.503781	11.469515	3.604433	10.503781	2453.841080	17.128318	1490.989138	39712	265	32259
gene889	NC_008261.1	1060624	1062102	+	protein_coding	CPF_RS04415	CPF_0895	.	WP_011590426.	acetaldehyde de	-215.225036	7.522418	-123.955588	7.275678	-1.736308	11.931955	12.336603	4.586900	11.540580	12.336603	4.586900	11.540580	4794.922251	33.766300	2871.845599	150025	1010	120128
gene890	NC_008261.1	1062457	1062747	+	protein_coding	CPF_RS04420	CPF_0896	.	WP_003453976.	ethanolamine ut	-47.502520	9.930778	-38.980099	9.822047	-1.218635	12.955441	13.098859	7.528927	12.813593	13.098859	7.528927	12.813593	8848.759378	233.126371	6320.173163	54474	1372	52016
gene891	NC_008261.1	1062825	1063601	+	protein_coding	CPF_RS04425	CPF_0897	.	WP_011590427.	ethanolamine ut	-110.359841	6.760276	-61.328512	6.493541	-1.799487	10.524709	10.957030	4.170959	10.109445	10.957030	4.170959	10.109445	1632.915763	25.963870	1179.047790	26841	408	25910
gene892	NC_008261.1	1063611	1064246	+	protein_coding	CPF_RS04430	CPF_0898	.	WP_003455694.	phosphate propa	-102.485401	7.429872	-62.415021	7.194743	-1.641999	11.122089	11.485567	4.806293	10.770114	11.485567	4.806293	10.770114	2457.819891	39.494523	1714.185301	33069	508	30834
gene893	NC_008261.1	1064454	1065212	+	protein_coding	CPF_RS04435	CPF_0899	.	WP_003455851.	ethanolamine ut	-75.303967	7.293409	-45.405019	7.048400	-1.658494	10.677835	11.049007	4.814353	10.319133	11.049007	4.814353	10.319133	1733.609028	39.739129	1352.400224	27836	610	29031
gene894	NC_008261.1	1065359	1065631	+	protein_coding	CPF_RS04440	CPF_0900	.	WP_003453956.	ethanolamine ut	-98.460542	6.107630	-61.225106	5.900051	-1.608173	9.909339	10.257975	3.636501	9.572553	10.257975	3.636501	9.572553	1043.577369	17.387569	764.272058	6027	96	5901
gene895	NC_008261.1	1065633	1066187	+	protein_coding	CPF_RS04445	CPF_0901	.	WP_011590428.	BMC domain-co	-90.807970	4.769046	-55.348578	4.576433	-1.640656	8.660374	9.024871	2.520124	8.310599	9.024871	2.520124	8.310599	437.780330	6.770970	322.488455	5140	76	5062
gene896	NC_008261.1	1066238	1067335	+	protein_coding	CPF_RS04450	CPF_0902	.	WP_003454016.	ethanolamine ut	-114.301046	6.393505	-64.656282	6.142264	-1.767826	10.249088	10.668313	3.831618	9.846337	10.668313	3.831618	9.846337	1368.377687	19.904451	977.877748	31785	442	30367
gene897	NC_008261.1	1067338	1067811	+	protein_coding	CPF_RS04455	CPF_0903	.	WP_003453912.	DUF861 domain	-121.232326	6.662838	-74.600106	6.446567	-1.625096	10.612813	10.968854	4.047224	10.268329	10.968854	4.047224	10.268329	1645.676052	23.888420	1306.969829	16502	229	17521
gene898	NC_008261.1	1068180	1068656	+	protein_coding	CPF_RS04460	CPF_0904	.	WP_011590429.	glutathione pero	-1.533933	6.136729	1.328075	5.627478	-2.037178	5.917597	6.453103	5.835867	5.426530	6.453103	5.835867	5.426530	68.972659	83.446434	36.469662	696	805	492
gene899	NC_008261.1	1069088	1069633	+	protein_coding	CPF_RS04465	CPF_0905	.	WP_003474278.	N-acetyltransfe	-1.602114	6.340530	-1.047643	6.043130	-1.529256	6.375853	6.689627	6.009650	6.076797	6.689627	6.009650	6.076797	81.294106	92.914822	58.217300	939	1026	899
gene900	NC_008261.1	1069925	1070236	+	protein_coding	CPF_RS04470	CPF_0906	.	WP_003459816.	hypothetical pro	4.433871	2.187955	4.469135	2.178764	-1.007953	1.357454	1.363181	3.511748	1.351752	1.363181	3.511748	1.351752	1.212053	15.848045	1.359915	8	100	12
gene901	NC_008261.1	1070550	1072190	+	protein_coding	CPF_RS04475	CPF_0908	.	WP_011590430.	diguanylate phos	-2.229497	4.284016	-2.629962	4.386936	1.179621	5.018987	4.901240	3.744521	5.139563	4.901240	3.744521	5.139563	22.641233	18.771965	30.208172	786	623	1402
gene902	NC_008261.1	1072272	1072892	+	protein_coding	CPF_RS04480	CPF_0909	.	WP_003480649.	flavodoxin family	-3.083949	8.098743	-2.800758	8.035637	-1.101112	8.882023	8.951776	7.326997	8.812814	8.951776	7.326997	8.812814	418.960359	208.054968	439.153902	5504	2613	7713
gene903	NC_008261.1	1073139	1074983	+	protein_coding	CPF_RS04485	CPF_0910	.	WP_011590431.	polysaccharide b	1.039480	0.500442	1.085504	0.466228	-1.044276	0.440932	0.473290	0.529152	0.410786	0.473290	0.529152	0.410786	0.281827	0.643199	0.287462	11	24	15
gene904	NC_008261.1	1075096	1076289	+	protein_coding	CPF_RS04490	CPF_0911	.	WP_011590432.	DegT/DnrJ/Ery	1.258087	0.657614	1.164515	0.725629	1.080352	0.565542	0.512533	0.843764	0.624035	0.512533	0.843764	0.624035	0.316717	1.159535	0.444193	8	28	15
gene905	NC_008261.1	1076295	1076897	+	protein_coding	CPF_RS04495	CPF_0912	.	WP_011590433.	sugar transferas	-1.068316	0.580865	1.035095	0.509660	-1.105808	0.553199	0.630487	0.535148	0.485385	0.630487	0.535148	0.485385	0.391957	0.655999	0.351819	5	8	6
gene906	NC_008261.1	1076920	1077945	+	protein_coding	CPF_RS04500	CPF_0913	.	WP_011590434.	glycosyltransfer	-1.011459	0.232141	1.077756	0.161246	-1.090106	0.167055	0.240505	0.224068	0.116037	0.240505	0.224068	0.116037	0.138217	0.240964	0.068924	3	5	2
gene907	NC_008261.1	1077932	1079059	+	protein_coding	CPF_RS04505	CPF_0914	.	WP_011590435.	glycosyltransfer	1.530356	0.782614	1.527936	0.784286	1.001583	0.534857	0.533717	1.147584	0.536000	0.533717	1.147584	0.536000	0.335249	1.753401	0.376147	8	40	12
gene910	NC_008261.1	1081573	1082385	+	protein_coding	CPF_RS04520	CPF_0917	.	WP_011590438.	LicD family prote	1.065121	0.112993	1.016342	0.155189	1.047995	0.104800	0.076304	0.167322	0.143936	0.076304	0.167322	0.143936	0.058143	0.182457	0.086981	1	3	2
gene911	NC_008261.1	1082401	1083114	+	protein_coding	CPF_RS04525	CPF_0918	.	WP_011590439.	capsule chain le	1.583447	0.978675	1.399779	1.095704	1.131212	0.785688	0.701771	1.364840	0.879640	0.701771	1.364840	0.879640	0.463432	2.216063	0.742811	7	32	15
gene912	NC_008261.1	1083114	1083731	+	protein_coding	CPF_RS04530	CPF_0919	.	WP_011590440.	tyrosine protein	1.242472	0.643672	1.544349	0.396619	-1.242964	0.311691	0.505843	0.819056	0.192058	0.505843	0.819056	0.192058	0.305955	1.120134	0.114426	4	14	2
gene913	NC_008261.1	1083893	1084318	+	protein_coding	CPF_RS04535	CPF_0920	.	WP_011590441.	hypothetical pro	-1.527288	8.106533	-1.306571	7.997367	-1.168928	8.304416	8.417773	7.806800	8.192586	8.417773	7.806800	8.192586	290.167122	272.648868	287.676227	2615	2349	3466
gene914	NC_008261.1	1084498	1084986	+	protein_coding	CPF_RS04540	CPF_0921	.	WP_011590442.	hypothetical pro	-1.431001	1.823355	-1.084303	1.640423	-1.319743	1.889404	2.100102	1.583077	1.699845	2.100102	1.583077	1.699845	2.416669	2.932374	1.952270	25	29	27
gene915	NC_008261.1	1085226	1087037	+	protein_coding	CPF_RS04545	CPF_0922	.	WP_011590443.	ribonucleoside t	-4.125201	5.151802	-3.004669	4.960530	-1.372930	6.041518	6.274472	4.230007	5.817213	6.274472	4.230007	5.817213	61.618030	26.960568	48.509628	2362	988	2486
gene916	NC_008261.1	1087034	1087492	+	protein_coding	CPF_RS04550	CPF_0923	.	WP_011590444.	hypothetical pro	-4.921493	5.735786	-3.293535	5.493234	-1.494289	6.703407	6.999395	4.700299	6.419935	6.999395	4.700299	6.419935	104.632627	36.518867	74.644207	1016	339	969
gene919	NC_008261.1	1089077	1090141	-	protein_coding	CPF_RS04565	CPF_0926	.	WP_003477284.	site-specific int	-1.625398	1.283495	-1.518006	1.245275	-1.070746	1.630808	1.680862	0.980068	1.582246	1.680862	0.980068	1.582246	1.642246	1.392842	1.726389	37	30	52
gene920	NC_008261.1	1090234	1090698	-	protein_coding	CPF_RS04570	CPF_0927	.	WP_011590446.	ImmA/IrrE famil	-2.623973	1.236730	-2.094122	1.137616	-1.253018	1.945445	2.114941	0.723188	1.789533	2.114941	0.723188	1.789533	2.439745	0.957017	2.129071	24	9	28
gene921	NC_008261.1	1090714	1091217	-	protein_coding	CPF_RS04575	CPF_0928	.	WP_011590447.	XRE family trans	-1.970967	3.857345	-1.402445	3.634616	-1.405379	4.124948	4.377726	3.398823	3.886767	4.377726	3.398823	3.886767	15.193946	14.323614	11.856082	162	146	169
gene922	NC_008261.1	1091369	1091605	+	protein_coding	CPF_RS04580	CPF_0929	.	WP_011590448.	transcriptional r	-1.000436	1.646387	-1.032880	1.669246	1.032430	1.669565	1.646702	1.646073	1.692746	1.646702	1.646073	1.692746	1.595614	3.129487	1.939456	8	15	13
gene923	NC_008261.1	1091617	1092348	+	protein_coding	CPF_RS04585	CPF_0930	.	WP_011590449.	hypothetical pro	-1.358336	1.927105	-1.911635	2.135661	1.407336	2.394478	2.160647	1.718806	2.653614	2.160647	1.718806	2.653614	2.583063	3.377452	4.830298	40	50	100
gene925	NC_008261.1	1092597	1093286	-	protein_coding	CPF_RS04595	CPF_0931	.	WP_011590451.	hypothetical pro	1.384939	0.405585	1.329522	0.453821	1.041682	0.261596	0.233792	0.703615	0.292707	0.233792	0.703615	0.292707	0.137015	0.931589	0.204973	2	13	4
gene926	NC_008261.1	1093445	1093747	+	protein_coding	CPF_RS04600	CPF_0933	.	WP_011590452.	histidine kinase	-2.244623	3.247716	-2.264834	3.253120	1.009004	3.889368	3.882907	2.716434	3.895839	3.882907	2.716434	3.895839	10.452454	7.996202	11.902620	67	49	102
gene928	NC_008261.1	1093939	1094121	+	protein_coding	CPF_RS04610	CPF_0935	.	WP_003477299.	hypothetical pro	-2.450729	1.082872	-2.359386	1.067207	-1.038714	1.880240	1.907839	0.614628	1.853040	1.907839	0.614628	1.853040	2.066450	0.810589	2.318543	8	3	12
gene929	NC_008261.1	1094418	1094603	+	protein_coding	CPF_RS04615	CPF_0937	.	WP_011590455.	hypothetical pro	-1.517705	2.030847	-2.225808	2.256602	1.466562	2.615644	2.353969	1.752078	2.906408	2.353969	1.752078	2.906408	3.049681	3.455896	5.702868	12	13	30
gene930	NC_008261.1	1094603	1095514	+	protein_coding	CPF_RS04620	CPF_0938	.	WP_011590456.	hypothetical pro	-2.003597	2.020277	-1.709677	1.928691	-1.171916	2.465750	2.582838	1.580246	2.353969	2.582838	1.580246	2.353969	3.835509	2.927718	3.760641	74	54	97
gene931	NC_008261.1	1095516	1096385	+	protein_coding	CPF_RS04625	CPF_0939	.	WP_011590457.	hypothetical pro	-1.897732	2.693488	-2.066272	2.744743	1.088811	3.255783	3.194984	2.270708	3.317738	3.194984	2.270708	3.317738	6.194007	5.512934	7.884379	114	97	194
gene932	NC_008261.1	1096403	1097116	+	protein_coding	CPF_RS04630	CPF_0940	.	WP_011590458.	hypothetical pro	-1.659691	1.973688	-1.727698	1.997640	1.040976	2.401488	2.372695	1.641781	2.430631	2.372695	1.641781	2.430631	3.111614	3.116338	3.961656	47	45	80
gene933	NC_008261.1	1097284	1097670	+	protein_coding	CPF_RS04635	CPF_0942	.	WP_011590460.	RusA family cros	-1.667547	2.517148	-2.121555	2.663020	1.272261	3.081701	2.912895	2.175167	3.260289	2.912895	2.175167	3.260289	5.007939	5.110687	7.491830	41	40	82
gene934	NC_008261.1	1097820	1098467	+	protein_coding	CPF_RS04640	CPF_0943	.	WP_011590461.	hypothetical pro	1.461190	3.879512	1.237449	4.006086	1.180808	3.733537	3.615574	4.162718	3.855348	3.615574	4.162718	3.855348	8.461923	25.791225	11.622234	116	338	213
gene935	NC_008261.1	1098594	1098782	+	protein_coding	CPF_RS04645	.		WP_041702635.	hypothetical pro	-1.715622	0.737054	-1.686021	0.729449	-1.017556	1.210326	1.222945	0.444214	1.197837	1.222945	0.444214	1.197837	1.000424	0.523237	1.122469	4	2	6
gene936	NC_008261.1	1098850	1098933	-	tRNA	CPF_RS04650	CPF_0944		.	tRNA-Gly	-1.828763	1.776024	-1.786800	1.762839	-1.023485	2.247250	2.264057	1.393189	2.230567	2.264057	1.393189	2.230567	2.813694	2.354567	3.367408	5	4	8
gene937	NC_008261.1	1099026	1099499	+	protein_coding	CPF_RS04655	CPF_0945	.	WP_011590462.	hypothetical pro	1.238073	1.584054	-1.387995	1.967915	1.718439	1.785821	1.437478	1.745575	2.218577	1.437478	1.745575	2.218577	1.296436	3.442436	3.356751	13	33	45
gene938	NC_008261.1	1099769	1099843	+	tRNA	CPF_RS04660	CPF_0946		.	tRNA-Trp	2.284585	3.321746	1.548627	3.641615	1.475232	3.046406	2.778819	3.970751	3.339761	2.778819	3.970751	3.339761	4.411872	22.415474	8.014431	7	34	17
gene939	NC_008261.1	1099982	1101157	+	protein_coding	CPF_RS04665	CPF_0947	.	WP_011590463.	hypothetical pro	3.343534	1.758771	2.568728	2.042250	1.301630	1.267781	1.091804	2.833177	1.472123	1.091804	2.833177	1.472123	0.844108	8.913717	1.533373	21	212	51
gene940	NC_008261.1	1101226	1101693	+	protein_coding	CPF_RS04670	CPF_0948	.	WP_011590464.	terminase small	1.447704	3.658054	-1.121346	4.016427	1.623377	3.734072	3.400894	3.934660	4.099892	3.400894	3.934660	4.099892	7.171312	21.764648	13.825799	71	206	183
gene941	NC_008261.1	1101686	1103050	+	protein_coding	CPF_RS04675	CPF_0949	.	WP_011590465.	terminase	1.288431	2.518508	1.197714	2.574513	1.075742	2.394414	2.342326	2.707942	2.447659	2.342326	2.707942	2.447659	3.012817	7.969302	4.014986	87	220	155
gene942	NC_008261.1	1103106	1103873	+	protein_coding	CPF_RS04680	CPF_0950	.	WP_041702636.	hypothetical pro	2.539504	3.500086	1.803514	3.787114	1.408087	3.128935	2.891791	4.236337	3.385527	2.891791	4.236337	3.385527	4.923964	27.298257	8.286980	80	424	180
gene943	NC_008261.1	1103919	1105439	+	protein_coding	CPF_RS04685	CPF_0951	.	WP_041702637.	phage capsid pro	1.152073	3.680791	1.080168	3.728281	1.066568	3.626281	3.580091	3.784324	3.673068	3.580091	3.784324	3.673068	8.235743	19.277724	10.135433	265	593	436
gene945	NC_008261.1	1106563	1107060	+	protein_coding	CPF_RS14750	.		WP_080503691.	hypothetical pro	-1.592121	5.029219	-1.202850	4.836314	-1.323624	5.169669	5.375870	4.704920	4.971377	5.375870	4.704920	4.971377	31.798129	36.637624	26.766835	335	369	377
gene946	NC_008261.1	1107106	1107297	+	protein_coding	CPF_RS04695	CPF_0953	.	WP_041702747.	hypothetical pro	-2.100866	5.254505	-2.128206	5.262923	1.013014	5.826533	5.817213	4.746229	5.835867	5.817213	4.746229	5.835867	42.592289	38.114547	48.985261	173	148	266
gene947	NC_008261.1	1107325	1107942	+	protein_coding	CPF_RS04700	CPF_0954	.	WP_011590470.	hypothetical pro	-2.146746	4.007395	-2.052081	3.978929	-1.046132	4.563536	4.596184	3.494032	4.531120	4.596184	3.494032	4.531120	18.204325	15.681871	19.052009	238	196	333
gene948	NC_008261.1	1108036	1108641	+	protein_coding	CPF_RS04705	CPF_0955	.	WP_011590471.	hypothetical pro	1.061296	2.545512	1.041562	2.559244	1.018946	2.516463	2.502960	2.588787	2.530038	2.502960	2.588787	2.530038	3.588156	7.180263	4.259271	46	88	73
gene949	NC_008261.1	1108666	1109571	+	protein_coding	CPF_RS04710	CPF_0956	.	WP_011590472.	hypothetical pro	1.069670	2.510601	1.233973	2.403219	-1.153601	2.357163	2.462488	2.559654	2.256344	2.462488	2.559654	2.256344	3.443514	6.985734	3.473336	66	128	89
gene950	NC_008261.1	1109586	1109855	+	protein_coding	CPF_RS04715	CPF_0957	.	WP_003460293.	hypothetical pro	1.307251	2.104013	1.308285	2.103389	-1.000790	1.919033	1.919603	2.306139	1.918463	1.919603	2.306139	1.918463	2.100891	5.677122	2.488140	12	31	19
gene951	NC_008261.1	1109891	1110253	+	protein_coding	CPF_RS04720	CPF_0958	.	WP_011590473.	hypothetical pro	-1.090320	1.213997	-1.204503	1.280426	1.104724	1.347904	1.277974	1.153222	1.421660	1.277974	1.153222	1.421660	1.041764	1.770790	1.461065	8	13	15
gene952	NC_008261.1	1110257	1110583	+	protein_coding	CPF_RS04725	CPF_0959	.	WP_011590474.	hypothetical pro	-1.505608	1.555030	-1.379796	1.502005	-1.091182	1.813932	1.877970	1.287624	1.752078	1.877970	1.287624	1.752078	2.023794	2.116950	2.054428	14	14	19
gene953	NC_008261.1	1110583	1110966	+	protein_coding	CPF_RS04730	CPF_0960	.	WP_011590475.	hypothetical pro	1.110033	1.840482	1.050201	1.881659	1.056971	1.806247	1.766721	1.917323	1.846658	1.766721	1.917323	1.846658	1.846487	3.991726	2.301939	15	31	25
gene954	NC_008261.1	1110966	1111352	+	protein_coding	CPF_RS04735	CPF_0961	.	WP_011590476.	hypothetical pro	1.364741	1.074698	-1.283543	1.491407	1.751704	1.212257	0.873545	1.322172	1.682304	0.873545	1.322172	1.682304	0.610724	2.172042	1.918639	5	17	21
gene955	NC_008261.1	1111362	1111820	+	protein_coding	CPF_RS04740	CPF_0962	.	WP_003460307.	hypothetical pro	1.007882	2.369783	-1.227162	2.518791	1.236834	2.512779	2.364127	2.375453	2.670779	2.364127	2.375453	2.670779	3.089546	6.032615	4.930061	30	56	64
gene956	NC_008261.1	1111833	1112201	+	protein_coding	CPF_RS04745	CPF_0963	.	WP_003460275.	hypothetical pro	1.605599	1.056928	1.536340	1.099766	1.045080	0.800366	0.769191	1.452302	0.832805	0.769191	1.452302	0.832805	0.512413	2.545995	0.670744	4	19	7
gene957	NC_008261.1	1112161	1112523	+	protein_coding	CPF_RS04750	.		WP_041702638.	bacteriophage G	1.071334	2.030665	-1.084118	2.138445	1.161453	2.086743	1.981569	2.080978	2.197499	1.981569	2.080978	2.197499	2.213749	4.631296	3.311748	17	34	34
gene958	NC_008261.1	1112564	1115776	+	protein_coding	CPF_RS04755	CPF_0965	.	WP_011590478.	membrane prote	1.073200	1.746954	-1.000294	1.798869	1.073516	1.747160	1.696738	1.798657	1.799082	1.696738	1.798657	1.799082	1.662470	3.616491	2.156902	113	235	196
gene959	NC_008261.1	1115780	1116133	+	protein_coding	CPF_RS04760	CPF_0966	.	WP_003460303.	hypothetical pro	2.495462	1.297196	1.671660	1.704467	1.492805	1.045433	0.795634	2.114941	1.373660	0.795634	2.114941	1.373660	0.534125	4.749041	1.398330	4	34	14
gene960	NC_008261.1	1116156	1116422	+	protein_coding	CPF_RS04765	CPF_0967	.	WP_003460286.	hypothetical pro	1.530390	1.666228	1.218711	1.853052	1.255745	1.542866	1.387315	2.001215	1.715859	1.387315	2.001215	1.715859	1.239290	4.259385	1.986392	7	23	15
gene961	NC_008261.1	1116489	1116869	+	protein_coding	CPF_RS04770	CPF_0968	.	WP_011590479.	DUF1617 domai	1.139683	0.496675	-1.025335	0.617651	1.168557	0.511400	0.411235	0.599867	0.635962	0.411235	0.599867	0.635962	0.248137	0.778676	0.464013	2	6	5
gene962	NC_008261.1	1116905	1122013	+	protein_coding	CPF_RS04775	CPF_0969	.	WP_011590480.	KID repeat-con	1.088130	1.583057	1.080624	1.588237	1.006946	1.528288	1.523303	1.645154	1.533290	1.523303	1.645154	1.533290	1.415604	3.126057	1.640202	153	323	237
gene964	NC_008261.1	1122294	1122710	+	protein_coding	CPF_RS04785	CPF_0971	.	WP_011590482.	hypothetical pro	-1.122351	1.556293	1.230506	1.317159	-1.381061	1.389511	1.641781	1.475256	1.176004	1.641781	1.475256	1.176004	1.587004	2.608657	1.102281	14	22	13
gene965	NC_008261.1	1122751	1123167	+	protein_coding	CPF_RS04790	CPF_0972	.	WP_011590483.	hypothetical pro	1.228689	0.955239	-1.042988	1.145241	1.281508	0.980898	0.818162	1.115282	1.176004	0.818162	1.115282	1.176004	0.566787	1.660054	1.102281	5	14	13
gene966	NC_008261.1	1123253	1124281	+	protein_coding	CPF_RS04795	CPF_0973	.	WP_011590484.	autolytic lysozym	1.471189	1.407126	1.190018	1.582451	1.236274	1.299955	1.155929	1.712911	1.461927	1.155929	1.712911	1.461927	0.918757	3.363667	1.511897	20	70	44
gene967	NC_008261.1	1124699	1125361	+	protein_coding	CPF_RS04800	CPF_0974	.	WP_011590485.	hypothetical pro	-1.035372	0.105322	-1.063290	0.119525	1.026964	0.151321	0.133341	0.083191	0.171726	0.133341	0.083191	0.171726	0.071297	0.074579	0.106660	1	1	2
gene968	NC_008261.1	1125585	1126673	+	protein_coding	CPF_RS04805	CPF_0975	.	WP_011590486.	hypothetical pro	-1.039583	4.611326	-1.212148	4.720151	1.165994	4.748901	4.639413	4.583408	4.860974	4.639413	4.583408	4.860974	19.055605	33.554196	24.091345	439	739	742
gene969	NC_008261.1	1126603	1126959	+	protein_coding	CPF_RS04810	.		WP_041702639.	hypothetical pro	1.386459	5.245264	1.247232	5.324510	1.111629	5.090620	5.014854	5.486259	5.167530	5.014854	5.486259	5.167530	24.628096	64.127315	30.801877	186	463	311
gene970	NC_008261.1	1126966	1127691	+	protein_coding	CPF_RS04815	CPF_0977	.	WP_011590488.	hypothetical pro	1.478580	5.365607	1.165308	5.543670	1.268832	5.259859	5.090912	5.655124	5.434413	5.090912	5.655124	5.434413	25.848777	72.942918	36.624039	397	1071	752
gene971	NC_008261.1	1128751	1129536	-	protein_coding	CPF_RS04820	CPF_0978	.	WP_003477359.	phosphorylase	1.063659	2.700767	1.148259	2.644048	-1.079536	2.600824	2.656616	2.745651	2.546204	2.656616	2.745651	2.546204	3.969241	8.240983	4.318508	66	131	96
gene972	NC_008261.1	1129893	1130990	+	protein_coding	CPF_RS04825	CPF_0979	.	WP_011590489.	hypothetical pro	-1.269661	5.251485	-1.821294	5.497584	1.434472	5.680832	5.426530	5.082087	5.947050	5.426530	5.082087	5.947050	33.106259	47.374395	52.972269	769	1052	1645
gene973	NC_008261.1	1131187	1131705	+	protein_coding	CPF_RS04830	CPF_0980	.	WP_011590490.	ClbS/DfsB famil	-1.880453	5.147684	-2.715255	5.384811	1.443937	5.882379	5.623341	4.712261	6.153349	5.623341	4.712261	6.153349	37.797828	36.870064	61.927214	415	387	909
gene974	NC_008261.1	1131742	1132584	+	protein_coding	CPF_RS04835	CPF_0981	.	WP_004457245.	hypothetical pro	-1.993767	5.595894	-2.481177	5.738432	1.244466	6.271516	6.115736	5.120239	6.431263	6.115736	5.120239	6.431263	53.550300	48.800698	75.664815	955	832	1804
gene975	NC_008261.1	1132601	1132915	+	protein_coding	CPF_RS04840	CPF_0982	.	WP_011590491.	DUF3784 domai	1.190215	1.471446	-1.341822	1.802067	1.597057	1.654786	1.351187	1.602409	2.026602	1.351187	1.602409	2.026602	1.200509	2.982451	2.806173	8	19	25
gene976	NC_008261.1	1133107	1133844	-	protein_coding	CPF_RS04845	CPF_0983	.	WP_004457215.	sugar phosphate	-1.337459	1.997849	-1.017920	1.811848	-1.313914	2.012026	2.218577	1.799082	1.824705	2.218577	1.799082	1.824705	2.690166	3.617993	2.251783	42	54	47
gene978	NC_008261.1	1135175	1136323	-	protein_coding	CPF_RS04855	CPF_0985	.	WP_011590492.	exo-alpha-sialid	-3.421689	6.558586	-5.136332	6.809815	1.501110	7.793205	7.505697	5.730988	8.091726	7.505697	5.730988	8.091726	146.664705	76.858465	265.506478	3565	1786	8628
gene979	NC_008261.1	1137105	1138484	+	protein_coding	CPF_RS04860	CPF_0986	.	WP_011590493.	hypothetical pro	1.698355	1.975294	1.746612	1.950646	-1.028414	1.609499	1.629836	2.393974	1.589415	1.629836	2.393974	1.589415	1.575669	6.091162	1.742268	46	170	68
gene980	NC_008261.1	1138908	1139201	-	protein_coding	CPF_RS04865	CPF_0987	.	WP_011590494.	hypothetical pro	-2.804442	5.154564	-1.595885	4.789428	-1.757296	5.530206	5.951818	4.464104	5.138461	5.951818	4.464104	5.138461	46.948490	30.945733	30.186406	292	184	251
gene981	NC_008261.1	1139592	1140212	-	protein_coding	CPF_RS04870	.		WP_041702640.	CPBP family intr	-1.494679	0.583040	-1.031613	0.383019	-1.448876	0.618215	0.941061	0.361225	0.406127	0.941061	0.361225	0.406127	0.685073	0.398115	0.284684	9	5	5
gene982	NC_008261.1	1141378	1141770	+	protein_coding	CPF_RS04875	CPF_0989	.	WP_011590496.	DUF4440 domai	1.780191	6.169054	1.379637	6.362704	1.290333	5.948080	5.767050	6.599082	6.134793	5.767050	6.599082	6.134793	41.616893	134.875329	61.178860	346	1072	680
gene983	NC_008261.1	1142100	1143245	+	protein_coding	CPF_RS04880	CPF_0990	.	WP_011590497.	hypothetical pro	1.161991	3.246610	1.057617	3.316060	1.098688	3.207288	3.140116	3.356715	3.275898	3.140116	3.356715	3.275898	5.939693	13.763736	7.620744	144	319	247
gene985	NC_008261.1	1143881	1145644	+	protein_coding	CPF_RS04890	CPF_0992	.	WP_011590499.	ATPase AAA	-1.774659	4.081279	-1.412497	3.929664	-1.256399	4.348207	4.515971	3.688429	4.186676	4.515971	3.688429	4.186676	17.203728	18.023647	14.772497	642	643	737
gene986	NC_008261.1	1145648	1146961	+	protein_coding	CPF_RS04895	CPF_0993	.	WP_011590500.	ATP-dependent	-1.419144	4.098131	-1.064751	3.898388	-1.332842	4.145984	4.358413	3.853392	3.943908	4.358413	3.853392	3.943908	14.929279	20.320233	12.324098	415	540	458
gene987	NC_008261.1	1146987	1147370	+	protein_coding	CPF_RS04900	CPF_0994	.	WP_003462042.	membrane prote	-1.358889	4.835442	-1.310765	4.810531	-1.036714	5.035636	5.061713	4.619285	5.009694	5.061713	4.619285	5.009694	25.604613	34.637883	27.531190	208	269	299
gene988	NC_008261.1	1147461	1148018	-	protein_coding	CPF_RS04905	CPF_0995	.	WP_011590501.	flavin reductase	-1.704611	3.667512	-1.999149	3.769632	1.172788	4.185750	4.072357	3.302914	4.302300	4.072357	3.302914	4.302300	12.029297	13.114683	16.094761	142	148	254
gene989	NC_008261.1	1148063	1148452	-	protein_coding	CPF_RS04910	CPF_0996	.	WP_011590502.	hypothetical pro	-2.308794	1.299617	-1.799420	1.179304	-1.283077	1.847974	2.036504	0.829364	1.676897	2.036504	0.829364	1.676897	2.302900	1.141059	1.903881	19	9	21
gene990	NC_008261.1	1148755	1149531	+	protein_coding	CPF_RS04915	CPF_0997	.	WP_011590503.	DUF1311 domai	-2.787746	5.832412	-2.943018	5.866765	1.055698	6.657646	6.618662	5.139563	6.696860	6.618662	5.139563	6.696860	77.140836	49.891358	93.286298	1268	784	2050
gene991	NC_008261.1	1149913	1150716	-	protein_coding	CPF_RS04920	CPF_0998	.	WP_011590504.	CPBP family intr	1.108105	3.363583	1.038870	3.410827	1.066645	3.336566	3.290350	3.438445	3.383431	3.290350	3.438445	3.383431	6.702470	14.759970	8.267740	114	240	188
gene994	NC_008261.1	1151594	1151818	+	protein_coding	CPF_RS04935	CPF_1001	.	WP_011590505.	hypothetical pro	-1.016020	3.446991	1.775629	2.992859	-1.804075	3.002829	3.458474	3.435545	2.607215	3.458474	3.435545	2.607215	7.563209	14.723890	4.557225	36	67	29
gene995	NC_008261.1	1152107	1153333	-	protein_coding	CPF_RS04940	CPF_1002	.	WP_011590506.	amidohydrolase	1.182905	7.435118	2.048756	7.020861	-1.731969	6.907377	7.314938	7.557273	6.522524	7.314938	7.557273	6.522524	129.559246	238.404190	82.098061	3363	5916	2849
gene996	NC_008261.1	1153742	1154620	+	protein_coding	CPF_RS04945	CPF_1003	.	WP_011590507.	hypothetical pro	-2.155925	6.281664	-1.750736	6.142617	-1.231439	6.708360	6.860213	5.751906	6.559868	6.860213	5.751906	6.559868	92.926798	78.022142	83.305992	1728	1387	2071
gene997	NC_008261.1	1155215	1156168	-	protein_coding	CPF_RS04950	CPF_1005	.	WP_011590509.	methionyl-tRNA	-1.966632	6.355448	-2.041815	6.380464	1.038229	6.889019	6.862009	5.886282	6.916135	6.862009	5.886282	6.916135	93.202278	86.608069	110.113177	1881	1671	2971
gene998	NC_008261.1	1156381	1157175	-	protein_coding	CPF_RS04955	CPF_1006	.	WP_011590510.	hypothetical pro	-1.836865	5.254485	-1.688702	5.198373	-1.087738	5.650392	5.711384	4.834138	5.590052	5.711384	4.834138	5.590052	40.075493	40.489661	40.383479	674	651	908
gene999	NC_008261.1	1157270	1157641	-	protein_coding	CPF_RS04960	CPF_1007	.	WP_003475668.	hypothetical pro	1.631179	2.353238	2.225720	2.076676	-1.364485	1.788429	2.026602	2.732518	1.578246	2.026602	2.732518	1.578246	2.287261	8.108064	1.710860	18	61	18
gene1000	NC_008261.1	1158231	1159394	+	protein_coding	CPF_RS04965	CPF_1008	.	WP_011590511.	transposase	-1.335375	3.073029	-1.290106	3.049694	-1.035090	3.263752	3.288725	2.871480	3.238969	3.288725	2.871480	3.238969	6.700652	9.175528	7.381393	165	216	243
gene1001	NC_008261.1	1160156	1162279	-	protein_coding	CPF_RS04970	CPF_1009	.	WP_011590512.	ferrous iron trar	-1.163458	0.859815	-1.015366	0.768435	-1.145851	0.872212	0.975933	0.757513	0.779514	0.975933	0.757513	0.779514	0.734422	1.047583	0.599284	33	45	36
gene1003	NC_008261.1	1162940	1163290	+	protein_coding	CPF_RS04980	CPF_1011	.	WP_011590514.	hypothetical pro	-2.907337	2.598337	-1.491745	2.209885	-1.948950	2.959599	3.479835	1.940137	2.517138	3.479835	1.940137	2.517138	7.676334	4.085274	4.230846	57	29	42
gene1004	NC_008261.1	1163290	1164111	+	protein_coding	CPF_RS04985	CPF_1012	.	WP_011590515.	membrane prote	-1.379661	2.237103	-1.426653	2.258781	1.034061	2.505318	2.481274	2.016960	2.529594	2.481274	2.016960	2.529594	3.507875	4.331028	4.258419	61	72	99
gene1005	NC_008261.1	1164131	1164424	+	protein_coding	CPF_RS04990	CPF_1013	.	WP_011590516.	hypothetical pro	-1.524574	3.674276	-1.546595	3.683788	1.014444	4.001382	3.991051	3.382644	4.011740	3.991051	3.382644	4.011740	11.415558	14.127400	12.868308	71	84	107
gene1006	NC_008261.1	1164914	1166227	+	protein_coding	CPF_RS04995	CPF_1014	.	WP_011590517.	hypothetical pro	1.203833	3.794384	1.274975	3.751235	-1.059096	3.621271	3.662925	3.930560	3.580091	3.662925	3.930560	3.580091	8.849645	21.712545	9.471795	246	577	352
gene1007	NC_008261.1	1166292	1166957	-	protein_coding	CPF_RS05000	CPF_1015	.	WP_011590518.	hypothetical pro	-1.029497	4.892969	1.167748	4.758865	-1.202193	4.779304	4.913984	4.872044	4.648315	4.913984	4.872044	4.648315	22.854291	41.427645	20.705007	322	558	390
gene1008	NC_008261.1	1166971	1168458	-	protein_coding	CPF_RS05005	CPF_1016	.	WP_011590519.	type II restrictio	-1.157722	5.318846	1.016623	5.202344	-1.176967	5.306701	5.425539	5.214250	5.190465	5.425539	5.214250	5.190465	33.101746	53.001485	31.175679	1042	1595	1312
gene1009	NC_008261.1	1168761	1169984	+	protein_coding	CPF_RS05010	CPF_1017	.	WP_011590520.	DNA (cytosine-	1.797968	5.517396	2.313377	5.317479	-1.286662	4.925247	5.110418	5.956785	4.746785	5.110418	5.956785	4.746785	26.183903	90.287243	22.214162	678	2235	769
gene1010	NC_008261.1	1170044	1171384	+	protein_coding	CPF_RS05015	CPF_1018	.	WP_011590521.	DNA cytosine m	1.656703	6.189321	1.910479	6.079306	-1.153182	5.732135	5.835867	6.564181	5.630247	5.835867	6.564181	5.630247	43.145822	132.445690	42.028564	1224	3592	1594
gene1011	NC_008261.1	1171434	1171916	-	protein_coding	CPF_RS05020	CPF_1019	.	WP_011590522.	hypothetical pro	4.906157	7.763094	4.828541	7.776411	1.016075	6.711611	6.700118	8.994711	6.723124	6.700118	8.994711	6.723124	82.208791	605.635488	94.726646	840	5916	1294
gene1012	NC_008261.1	1172294	1172737	-	protein_coding	CPF_RS05025	CPF_1020	.	WP_041702642.	ImmA/IrrE famil	1.081114	0.402661	1.159676	0.339553	-1.072667	0.295409	0.350313	0.462832	0.249110	0.350313	0.462832	0.249110	0.212928	0.556823	0.159269	2	5	2
gene1013	NC_008261.1	1172854	1173396	-	protein_coding	CPF_RS05030	CPF_1021	.	WP_011590524.	XRE family trans	-1.024268	0.277117	1.025475	0.241533	-1.050361	0.257079	0.294953	0.260360	0.224068	0.294953	0.260360	0.224068	0.174107	0.273182	0.130231	2	3	2
gene1014	NC_008261.1	1173781	1174608	+	protein_coding	CPF_RS05035	CPF_1022	.	WP_041702748.	hypothetical pro	-1.156375	4.728172	1.163907	4.513713	-1.345913	4.614873	4.834138	4.624529	4.405553	4.834138	4.624529	4.405553	21.693987	34.815168	17.465378	380	583	409
gene1015	NC_008261.1	1175129	1175359	+	protein_coding	CPF_RS05040	CPF_1023	.	WP_003475677.	DUF4318 domai	1.336924	1.962497	1.267044	2.005113	1.055152	1.802494	1.764185	2.183102	1.841636	1.764185	2.183102	1.841636	1.841690	5.137236	2.295960	9	24	15
gene1016	NC_008261.1	1175800	1176030	+	protein_coding	CPF_RS05045	CPF_1024	.	WP_011590526.	DUF4318 domai	-2.254156	5.161526	-1.894539	5.048350	-1.189817	5.654251	5.781011	4.608424	5.530271	5.781011	4.608424	5.530271	41.744984	34.462293	39.031318	204	161	255
gene1017	NC_008261.1	1176353	1177627	+	protein_coding	CPF_RS05050	CPF_1025	.	WP_011590527.	GNAT family N-a	-1.076229	3.810547	1.044657	3.726275	-1.124290	3.778456	3.863908	3.757923	3.694894	3.863908	3.757923	3.694894	10.306726	18.963957	10.260690	278	489	370
gene1018	NC_008261.1	1178112	1178423	-	protein_coding	CPF_RS05055	CPF_1026	.	WP_011590528.	DUF3784 domai	-3.700584	3.342768	-4.773666	3.478984	1.289977	4.597352	4.417347	2.529594	4.784692	4.417347	2.529594	4.784692	15.756685	6.814659	22.778571	104	43	201
gene1019	NC_008261.1	1178726	1179541	+	protein_coding	CPF_RS05060	CPF_1027	.	WP_011590529.	hypothetical pro	1.831061	2.052578	2.153927	1.902421	-1.176327	1.540513	1.662105	2.534785	1.427816	1.662105	2.534785	1.427816	1.622012	6.847288	1.473241	28	113	34
gene1020	NC_008261.1	1179642	1180868	-	protein_coding	CPF_RS05065	CPF_1028	.	WP_011590530.	tetracycline res	-1.358171	0.654189	-1.365551	0.656989	1.005434	0.915190	0.911289	0.469624	0.919107	0.911289	0.469624	0.919107	0.654923	0.564175	0.778044	17	14	27
gene1021	NC_008261.1	1181122	1181595	-	protein_coding	CPF_RS05070	CPF_1029	.	WP_011590531.	transcriptional r	-1.559850	5.024434	-1.967301	5.179113	1.261211	5.520230	5.355362	4.713954	5.690172	5.355362	4.713954	5.690172	31.114467	36.927950	44.085336	312	354	591
gene1022	NC_008261.1	1181874	1182305	+	protein_coding	CPF_RS05075	CPF_1030	.	WP_003475672.	hypothetical pro	-1.361122	2.692394	1.320898	2.269543	-1.797904	2.464743	2.923962	2.479165	2.077646	2.923962	2.479165	2.077646	5.033385	6.524112	2.946482	46	57	36
gene1023	NC_008261.1	1182338	1182733	-	protein_coding	CPF_RS05080	CPF_1031	.	WP_003453973.	(deoxy)nucleosi	1.438837	5.070016	2.400263	4.665099	-1.668196	4.429854	4.814353	5.339256	4.076064	4.814353	5.339256	4.076064	21.367020	57.562019	13.482387	179	461	151
gene1024	NC_008261.1	1183107	1183316	+	protein_coding	CPF_RS05085	CPF_1032	.	WP_003453830.	transcriptional r	1.071834	3.072452	-1.261879	3.286407	1.352525	3.233317	3.022819	3.122900	3.458474	3.022819	3.122900	3.458474	5.402292	11.301920	8.586890	24	48	51
gene1025	NC_008261.1	1183309	1183815	+	protein_coding	CPF_RS05090	CPF_1033	.	WP_003459130.	hypothetical pro	-1.174969	2.744613	-1.503976	2.910305	1.280014	3.036250	2.863388	2.630765	3.219547	2.863388	2.630765	3.219547	4.848211	7.509535	7.252878	52	77	104
gene1026	NC_008261.1	1184257	1184709	+	protein_coding	CPF_RS05095	CPF_1034	.	WP_003480756.	DUF1232 domai	-4.124442	7.476663	-3.213140	7.317815	-1.283617	8.386261	8.568303	6.524104	8.208087	8.568303	6.524104	8.208087	317.116330	127.707952	293.243239	3039	1170	3757
gene1027	NC_008261.1	1185165	1186094	+	protein_coding	CPF_RS05100	CPF_1035	.	WP_011590532.	hypothetical pro	1.129326	4.310991	-1.148502	4.498383	1.297033	4.407770	4.224153	4.399615	4.599368	4.224153	4.399615	4.599368	13.316940	30.146048	19.960038	262	567	525
gene1028	NC_008261.1	1186126	1186515	+	protein_coding	CPF_RS05105	CPF_1036	.	WP_011590533.	hypothetical pro	-1.104684	5.622739	-1.543078	5.855933	1.396851	5.931206	5.695014	5.551381	6.177192	5.695014	5.551381	6.177192	39.512918	67.068925	62.828059	326	529	693
gene1029	NC_008261.1	1186516	1187205	+	protein_coding	CPF_RS05110	CPF_1037	.	WP_011590534.	hypothetical pro	1.191119	5.753288	-1.223550	6.024608	1.457394	5.893948	5.628512	5.880830	6.171903	5.628512	5.880830	6.171903	37.953059	86.422833	62.670388	554	1206	1223
gene1030	NC_008261.1	1187379	1187696	-	protein_coding	CPF_RS05115	CPF_1038	.	WP_011590535.	hypothetical pro	-1.037863	3.467120	-1.319562	3.634946	1.271422	3.663160	3.494032	3.440416	3.840475	3.494032	3.440416	3.840475	7.729695	14.771574	11.563551	52	95	104
gene1031	NC_008261.1	1187903	1188598	-	protein_coding	CPF_RS05120	CPF_1039	.	WP_011590536.	radical SAM prot	1.027307	5.288205	1.001105	5.306878	1.026173	5.287411	5.268807	5.307674	5.306081	5.268807	5.307674	5.306081	29.000450	56.266023	34.138548	427	792	672
gene1032	NC_008261.1	1188620	1189324	-	protein_coding	CPF_RS05125	CPF_1040	.	WP_011590537.	hypothetical pro	-1.030192	6.166729	-1.263712	6.311886	1.226677	6.333886	6.188223	6.145309	6.482978	6.188223	6.145309	6.482978	57.528663	100.785471	79.692930	858	1437	1589
gene1033	NC_008261.1	1189698	1190255	+	protein_coding	CPF_RS05130	CPF_1041	.	WP_004456258.	hypothetical pro	-1.069703	0.378394	-1.382589	0.515647	1.292498	0.586688	0.430108	0.332897	0.800270	0.430108	0.332897	0.800270	0.254140	0.354451	0.633652	3	4	10
gene1035	NC_008261.1	1191279	1191758	-	protein_coding	CPF_RS05140	CPF_1043	.	WP_011590539.	NUDIX domain-	-1.201185	6.229149	1.008451	6.092249	-1.211336	6.222945	6.362781	6.098323	6.086182	6.362781	6.098323	6.086182	65.882639	98.479750	58.634989	669	956	796
gene1036	NC_008261.1	1192056	1192568	+	protein_coding	CPF_RS05145	CPF_1044	.	WP_003453965.	flavodoxin family	-2.826142	3.735351	-2.841691	3.738593	1.005502	4.563160	4.559204	3.060370	4.567120	4.559204	3.060370	4.567120	17.876005	10.698820	19.643212	194	111	285
gene1037	NC_008261.1	1192556	1193158	+	protein_coding	CPF_RS05150	CPF_1045	.	WP_011590540.	hypothetical pro	-3.905047	2.912582	-3.895471	2.911310	-1.002458	4.054785	4.056556	2.091216	4.053014	4.056556	2.091216	4.053014	11.915503	4.673990	13.134566	152	57	224
gene1038	NC_008261.1	1193329	1194477	+	protein_coding	CPF_RS05155	CPF_1046	.	WP_004457104.	lactaldehyde red	-8.815231	6.869510	-30.662919	7.552449	3.478402	9.473265	8.616634	5.476636	10.415059	8.616634	5.476636	10.415059	328.751097	63.431902	1419.450315	7991	1474	46127
gene1039	NC_008261.1	1194534	1195391	-	protein_coding	CPF_RS05160	CPF_1047	.	WP_003459136.	AraC family tran	-4.283363	4.163545	-5.893680	4.339228	1.375947	5.568578	5.343122	3.244378	5.803547	5.343122	3.244378	5.803547	30.907344	12.563177	47.885481	561	218	1162
gene1040	NC_008261.1	1195542	1197335	+	protein_coding	CPF_RS05165	CPF_1048	.	WP_003477484.	L-fucose isome	-48.324408	4.721698	-218.957676	5.306589	4.530996	9.311814	8.285468	2.690788	10.465296	8.285468	2.690788	10.465296	266.809686	7.799994	1470.915267	10126	283	74632
gene1041	NC_008261.1	1197356	1198858	+	protein_coding	CPF_RS05170	CPF_1049	.	WP_011590541.	rhamnulokinase	-44.805766	3.302407	-195.184227	3.787669	4.356230	8.027348	7.035693	1.550081	9.158773	7.035693	1.550081	9.158773	107.623505	2.829240	574.005244	3422	86	24400
gene1042	NC_008261.1	1198852	1199289	+	protein_coding	CPF_RS05175	CPF_1050	.	WP_004456284.	fucose isomeras	-54.073457	3.535783	-213.691411	3.979212	3.951873	8.370488	7.437710	1.680862	9.420247	7.437710	1.680862	9.420247	139.651715	3.273815	691.575614	1294	29	8567
gene1043	NC_008261.1	1199318	1200022	+	protein_coding	CPF_RS05180	CPF_1051	.	WP_003462064.	class II aldolase	-50.632726	3.148288	-202.674602	3.566376	4.002838	8.003157	7.064942	1.402944	9.065965	7.064942	1.402944	9.065965	110.229745	2.384625	531.670706	1644	34	10601
gene1044	NC_008261.1	1200057	1201391	+	protein_coding	CPF_RS05185	CPF_1052	.	WP_011590542.	L-fucose:H+ syr	-31.001713	3.200269	-116.916646	3.639785	3.771296	7.420107	6.524104	1.569828	8.439164	6.524104	1.569828	8.439164	71.878810	2.926012	350.823356	2030	79	13246
gene1045	NC_008261.1	1201696	1202742	+	protein_coding	CPF_RS05190	CPF_1053	.	WP_003459193.	transcriptional r	-1.803312	3.322170	-2.084599	3.412958	1.155984	3.877762	3.774610	2.923962	3.983732	3.774610	2.923962	3.983732	9.706840	9.634158	12.495109	215	204	370
gene1046	NC_008261.1	1202947	1203477	+	protein_coding	CPF_RS05195	CPF_1054	.	WP_011590543.	UBA/TS-N dom	-2.596778	6.243083	-1.687564	5.958082	-1.538773	6.651127	6.969279	5.592557	6.347499	6.969279	5.592557	6.347499	101.572755	69.466367	71.248261	1141	746	1070
gene1047	NC_008261.1	1204006	1204554	+	protein_coding	CPF_RS05200	CPF_1055	.	WP_004456268.	hypothetical pro	-1.690450	3.912548	-1.238167	3.703027	-1.365284	4.078757	4.309537	3.552129	3.860335	4.309537	3.552129	3.860335	14.292949	16.301836	11.657119	166	181	181
gene1048	NC_008261.1	1204584	1205264	+	protein_coding	CPF_RS05205	CPF_1056	.	WP_003459138.	DNA-binding res	-2.440217	5.401447	-2.016076	5.277735	-1.210379	5.943824	6.083149	4.796139	5.807689	6.083149	4.796139	5.807689	52.128945	39.280810	48.078276	751	541	926
gene1049	NC_008261.1	1205257	1206513	+	protein_coding	CPF_RS05210	CPF_1057	.	WP_003459145.	sensor histidine	-2.354081	5.576055	-2.108700	5.504500	-1.116366	6.147815	6.227733	4.992569	6.068923	6.227733	4.992569	6.068923	59.190984	44.528845	58.057647	1574	1132	2064
gene1050	NC_008261.1	1206647	1207432	+	protein_coding	CPF_RS05215	CPF_1058	.	WP_011590544.	HAD family hydr	-1.429590	5.198161	-1.276575	5.119859	-1.119864	5.380069	5.462350	4.946749	5.299027	5.462350	4.946749	5.299027	33.858831	43.721247	34.053233	563	695	757
gene1051	NC_008261.1	1207726	1208283	-	protein_coding	CPF_RS05220	CPF_1059	.	WP_011590545.	N-acetyltransfe	-1.160228	2.333984	-1.229631	2.373675	1.059819	2.485204	2.443649	2.229241	2.527466	2.443649	2.229241	2.527466	3.388534	5.316763	4.245468	40	60	67
gene1052	NC_008261.1	1208403	1208894	-	protein_coding	CPF_RS05225	CPF_1060	.	WP_003460378.	hypothetical pro	-1.041022	1.507316	-1.042794	1.508519	1.001703	1.537821	1.536595	1.478594	1.539049	1.536595	1.478594	1.539049	1.441160	2.612995	1.652904	15	26	23
gene1053	NC_008261.1	1209210	1209668	+	protein_coding	CPF_RS05230	CPF_1061	.	WP_003460381.	N-acetyltransfe	-1.084388	1.318322	1.025237	1.243067	-1.111755	1.299392	1.378057	1.261176	1.225218	1.378057	1.261176	1.225218	1.235818	2.046780	1.155483	12	19	15
gene1054	NC_008261.1	1209702	1210151	-	protein_coding	CPF_RS05235	CPF_1062	.	WP_003460384.	NUDIX domain-	-1.321115	2.173183	1.110810	1.904255	-1.467507	2.088392	2.383325	1.981569	1.829957	2.383325	1.981569	1.829957	3.151337	4.175432	2.278613	30	38	29
gene1055	NC_008261.1	1210835	1212460	+	protein_coding	CPF_RS05240	CPF_1064	.	WP_003460382.	diguanylate phos	4.514934	2.414032	4.852705	2.332123	-1.074812	1.507326	1.560266	3.734971	1.456182	1.560266	3.734971	1.456182	1.453569	18.549815	1.500422	50	610	69
gene1056	NC_008261.1	1212579	1212926	+	protein_coding	CPF_RS05245	CPF_1065	.	WP_003454002.	hypothetical pro	2.740492	4.344400	3.253091	4.195744	-1.187046	3.551786	3.677627	5.132062	3.430251	3.677627	5.132062	3.430251	8.965010	49.445899	8.534637	66	348	84
gene1058	NC_008261.1	1213607	1213858	+	protein_coding	CPF_RS05250	CPF_1067	.	WP_003480805.	spore coat prot	1.727853	1.324478	1.493432	1.458739	1.156968	1.087589	0.987488	1.776469	1.197837	0.987488	1.776469	1.197837	0.750318	3.531850	1.122469	4	18	8
gene1059	NC_008261.1	1213871	1214437	+	protein_coding	CPF_RS05255	CPF_1068	.	WP_003480806.	hypothetical pro	1.092829	3.417824	1.460676	3.197479	-1.336601	3.138135	3.354390	3.482457	2.935822	3.354390	3.482457	2.935822	7.002971	15.522699	5.861784	84	178	94
gene1060	NC_008261.1	1214613	1214831	-	protein_coding	CPF_RS05260	CPF_1069	.	WP_003454064.	DUF4318 domai	-2.322730	3.110399	-1.694544	2.917082	-1.370711	3.542402	3.777159	2.561338	3.322235	3.777159	2.561338	3.322235	9.713025	6.999191	7.911102	45	31	49
gene1061	NC_008261.1	1215484	1216935	+	protein_coding	CPF_RS05265	CPF_1070	.	WP_004458822.	PTS glucose tra	-6.468321	1.661881	-5.359491	1.595899	-1.206891	3.347328	3.485723	0.792332	3.214428	3.485723	0.792332	3.214428	7.715567	1.089717	7.232274	237	32	297
gene1062	NC_008261.1	1217212	1217538	+	protein_coding	CPF_RS05270	CPF_1071	.	WP_011590547.	DUF3784 domai	-1.118056	1.456330	-1.519971	1.652709	1.359477	1.746582	1.539049	1.378057	1.982100	1.539049	1.378057	1.982100	1.445567	2.268161	2.703194	10	15	25
gene1063	NC_008261.1	1217570	1217959	+	protein_coding	CPF_RS05275	CPF_1072	.	WP_041702644.	hypothetical pro	-4.048657	4.591911	-3.718347	4.542274	-1.088833	5.648398	5.710123	3.692679	5.587340	5.710123	3.692679	5.587340	39.997739	18.130163	40.344136	330	143	445
gene1064	NC_008261.1	1218551	1224979	+	protein_coding	CPF_RS05280	CPF_1073	.	WP_011590549.	DUF5011 domai	-8.041069	7.824239	-7.619540	7.792085	-1.055322	9.432194	9.471115	6.463728	9.393432	9.471115	6.463728	9.393432	632.532011	122.049615	675.136197	86028	15869	122758
gene1065	NC_008261.1	1225148	1225447	-	protein_coding	CPF_RS05285	CPF_1074	.	WP_011590550.	hypothetical pro	-1.116062	3.300899	-1.571977	3.533899	1.408503	3.619716	3.381058	3.222640	3.875220	3.381058	3.222640	3.875220	7.090508	12.361475	11.785927	45	75	100
gene1066	NC_008261.1	1225790	1226188	+	protein_coding	CPF_RS05290	CPF_1075	.	WP_011590551.	hypothetical pro	-1.440060	3.558844	-1.607024	3.631598	1.115942	3.909949	3.831618	3.305489	3.989881	3.831618	3.305489	3.989881	10.070062	13.136003	12.583471	85	106	142
gene1067	NC_008261.1	1226670	1228760	+	protein_coding	CPF_RS05295	CPF_1076	.	WP_011590552.	iron hydrogenas	-5.566438	8.745121	-3.416346	8.433783	-1.629354	9.712213	10.070745	7.593990	9.366445	10.070745	7.593990	9.366445	927.790688	244.935737	662.768181	41041	10358	39195
gene1068	NC_008261.1	1229188	1230621	+	protein_coding	CPF_RS05300	CPF_1077	.	WP_003480817.	DUF3502 domai	-21.298008	5.841930	-35.694210	6.093982	1.675941	8.815622	8.451002	4.038356	9.195974	8.451002	4.038356	9.195974	294.531340	23.722998	585.474571	8935	688	23745
gene1069	NC_008261.1	1230684	1230983	-	protein_coding	CPF_RS05305	CPF_1078	.	WP_003460376.	hypothetical pro	-1.357526	5.445843	1.097912	5.161993	-1.490445	5.375220	5.670794	5.229814	5.095051	5.670794	5.229814	5.095051	38.919012	53.401571	29.346959	247	324	249
gene1070	NC_008261.1	1231448	1232005	+	protein_coding	CPF_RS05310	CPF_1080	.	WP_003460380.	hypothetical pro	-1.686028	3.126564	-1.798992	3.167773	1.067000	3.572477	3.526003	2.772375	3.619563	3.526003	2.772375	3.619563	7.878343	8.418209	9.694876	93	95	153
gene1071	NC_008261.1	1232192	1233535	+	protein_coding	CPF_RS05315	CPF_1081	.	WP_003480819.	MATE family effl	-2.335306	1.567349	-2.191745	1.535766	-1.065501	2.248099	2.294330	1.070718	2.202799	2.294330	1.070718	2.202799	2.884036	1.581974	3.314792	82	43	126
gene1072	NC_008261.1	1233775	1234200	-	protein_coding	CPF_RS05320	CPF_1082	.	WP_003456311.	MarR family tran	-2.392299	5.767847	-2.245623	5.726768	-1.065316	6.385459	6.431263	5.172866	6.339981	6.431263	5.172866	6.339981	68.685831	50.954812	70.715564	619	439	852

gene1074	NC_008261.1	1234935	1235414	+	protein_coding	CPF_RS05330	CPF_1085	.	WP_003451929.	N-acetyltransfe	-2.602020	3.875355	-2.907724	3.941910	1.117487	4.705533	4.626086	3.246455	4.786345	4.626086	3.246455	4.786345	18.711063	12.567499	22.835234	190	122	310
gene1075	NC_008261.1	1235541	1236104	-	protein_coding	CPF_RS05335	CPF_1086	.	WP_011590555.	hypothetical pro	-1.237208	1.423260	-1.310895	1.460251	1.059559	1.626259	1.585062	1.277974	1.668526	1.585062	1.277974	1.668526	1.508619	2.104081	1.880733	18	24	30
gene1076	NC_008261.1	1236091	1236570	-	protein_coding	CPF_RS14760	.	.	WP_080503692.	hypothetical pro	-1.308325	1.287903	-1.166439	1.214539	-1.121641	1.411039	1.496272	1.108551	1.330661	1.496272	1.108551	1.330661	1.378710	1.648197	1.325917	14	16	18
gene1077	NC_008261.1	1237069	1245018	+	protein_coding	CPF_RS05340	CPF_1087	.	WP_041702645.	type I pullulanas	-39.216961	7.835088	-37.950518	7.818074	-1.033371	10.893042	10.916747	5.623341	10.869389	10.916747	5.623341	10.869389	1593.393286	71.065264	1820.703399	267981	11426	409375
gene1078	NC_008261.1	1245204	1245383	-	protein_coding	CPF_RS05345	CPF_1088	.	WP_011590557.	hypothetical pro	-2.253456	2.659997	-2.411536	2.699014	1.070150	3.358415	3.309865	2.137725	3.407678	3.309865	2.137725	3.407678	6.827897	4.944590	8.446581	26	18	43
gene1079	NC_008261.1	1245440	1246039	-	protein_coding	CPF_RS05350	CPF_1089	.	WP_003477519.	YdcF family prot	-2.112055	3.431284	-3.021812	3.645538	1.430745	4.263295	4.012734	2.934087	4.529501	4.012734	2.934087	4.529501	11.581163	9.724360	19.034273	147	118	323
gene1080	NC_008261.1	1246197	1246496	-	protein_coding	CPF_RS05355	CPF_1090	.	WP_011590558.	hypothetical pro	-1.287087	2.024428	-1.245147	2.002465	-1.033682	2.190626	2.214653	1.850543	2.166860	2.214653	1.850543	2.166860	2.678636	3.790852	3.182200	17	23	27
gene1082	NC_008261.1	1247365	1247949	+	protein_coding	CPF_RS05365	CPF_1092	.	WP_004457096.	sigma-70 family	1.342400	2.672928	1.335762	2.676797	1.004970	2.472520	2.468947	2.893762	2.476099	2.468947	2.893762	2.476099	3.474551	9.382042	4.109964	43	111	68
gene1083	NC_008261.1	1247942	1248724	+	protein_coding	CPF_RS05370	CPF_1093	.	WP_011590560.	membrane prote	1.146487	0.673726	1.149054	0.671856	-1.002239	0.580678	0.582294	0.779514	0.579067	0.582294	0.779514	0.579067	0.362223	1.073538	0.406411	6	17	9
gene1084	NC_008261.1	1248718	1249590	+	protein_coding	CPF_RS05375	CPF_1094	.	WP_011590561.	multidrug ABC t	-1.220423	3.593529	-1.179418	3.569763	-1.034767	3.715356	3.740091	3.452710	3.690785	3.740091	3.452710	3.690785	9.475670	15.065990	10.246872	175	266	253
gene1085	NC_008261.1	1249595	1250866	+	protein_coding	CPF_RS05380	CPF_1095	.	WP_011590562.	membrane prote	1.341800	2.542882	1.543356	2.430686	-1.150213	2.236399	2.339626	2.763796	2.137725	2.339626	2.763796	2.137725	3.010121	8.357601	3.141061	81	215	113
gene1086	NC_008261.1	1250901	1252049	+	protein_coding	CPF_RS05385	CPF_1096	.	WP_011590563.	hypothetical pro	1.101384	3.701633	1.280465	3.589186	-1.162596	3.522278	3.632629	3.771947	3.415280	3.632629	3.771947	3.415280	8.598295	19.063998	8.493253	209	443	276
gene1088	NC_008261.1	1253040	1253390	-	protein_coding	CPF_RS05395	CPF_1098	.	WP_011590565.	hypothetical pro	1.192142	0.317948	-1.075674	0.519029	1.282355	0.351812	0.215514	0.469070	0.574310	0.215514	0.469070	0.574310	0.134673	0.563486	0.402938	1	4	4
gene1090	NC_008261.1	1256095	1256430	+	protein_coding	CPF_RS05405	CPF_1100	.	WP_003451843.	hypothetical pro	2.441964	1.307887	2.469431	1.294855	-1.011248	0.805722	0.813830	2.101872	0.797693	0.813830	2.101872	0.797693	0.562739	4.709133	0.631389	4	32	6
gene1091	NC_008261.1	1256667	1258130	+	protein_coding	CPF_RS05410	CPF_1101	.	WP_011590568.	sulfatase	-1.570472	1.607381	-1.141020	1.406375	-1.376375	1.719820	1.965626	1.314428	1.504753	1.965626	1.314428	1.504753	2.163315	2.161569	1.593998	67	64	66
gene1092	NC_008261.1	1258382	1259071	-	protein_coding	CPF_RS05415	CPF_1102	.	WP_011590569.	membrane prote	3.035929	0.655153	2.914448	0.733069	1.041682	0.261596	0.233792	1.835930	0.292707	0.233792	1.835930	0.292707	0.137015	3.726358	0.204973	2	52	4
gene1093	NC_008261.1	1259269	1261191	+	protein_coding	CPF_RS05420	CPF_1103	.	WP_011590570.	beta-N-acetylh	-5.368663	7.722715	-3.820265	7.509860	-1.405312	8.780692	9.029567	6.605004	8.538677	9.029567	6.605004	8.538677	439.786040	135.352686	365.621163	17891	5264	19885
gene1094	NC_008261.1	1261496	1261717	+	protein_coding	CPF_RS05425	CPF_1104	.	WP_003456362.	hypothetical pro	-4.520158	5.573228	-3.941269	5.491203	-1.146879	6.667067	6.766657	4.590283	6.568943	6.766657	4.590283	6.568943	87.300552	33.854850	83.775646	410	152	526
gene1095	NC_008261.1	1262167	1262631	+	protein_coding	CPF_RS05430	CPF_1105	.	WP_003451945.	hypothetical pro	-1.164434	6.055547	1.363377	5.718767	-1.587562	5.823414	6.166357	5.946729	5.499544	6.166357	5.946729	5.499544	56.114130	89.746966	38.475350	552	844	506
gene1096	NC_008261.1	1262784	1263206	+	protein_coding	CPF_RS05435	CPF_1106	.	WP_011590571.	MarR family tran	-1.113953	5.868690	1.195757	5.660931	-1.332018	5.736517	5.947050	5.791362	5.533437	5.947050	5.791362	5.533437	46.934806	81.007112	39.035660	420	693	467
gene1097	NC_008261.1	1263374	1264051	+	protein_coding	CPF_RS05440	CPF_1107	.	WP_003451821.	hypothetical pro	1.194510	5.822197	1.533083	5.635177	-1.283441	5.512452	5.695399	5.951818	5.335382	5.695399	5.951818	5.335382	39.531152	89.994454	34.419080	567	1234	660
gene1098	NC_008261.1	1264229	1264960	+	protein_coding	CPF_RS05445	CPF_1108	.	WP_011590572.	hypothetical pro	1.538464	3.562494	1.677893	3.493551	-1.090629	3.202085	3.265276	3.886767	3.140116	3.265276	3.886767	3.140116	6.586811	20.940203	6.955629	102	310	144
gene1099	NC_008261.1	1265288	1265839	+	protein_coding	CPF_RS05450	CPF_1109	.	WP_003451892.	hypothetical pro	-1.415548	5.778683	-1.392824	5.767494	-1.016315	6.023113	6.034798	5.533437	6.011450	6.034798	5.533437	6.011450	50.267251	66.734049	55.342615	587	745	864
gene1100	NC_008261.1	1266078	1267112	-	protein_coding	CPF_RS05455	CPF_1110	.	WP_041702648.	acyltransferase	1.074005	0.221578	1.014210	0.268613	1.058957	0.213341	0.175983	0.278985	0.258628	0.175983	0.278985	0.258628	0.091343	0.286643	0.170811	2	6	5
gene1101	NC_008261.1	1267397	1268917	+	protein_coding	CPF_RS05460	CPF_1111	.	WP_011590574.	DNA-binding res	-2.644726	2.750075	-2.806379	2.783125	1.061123	3.582250	3.539709	2.136591	3.625301	3.539709	2.136591	3.625301	7.924960	4.941339	9.809983	255	152	422
gene1102	NC_008261.1	1268935	1270659	+	protein_coding	CPF_RS05465	CPF_1112	.	WP_011590575.	HAMP domain-c	-3.081593	2.859473	-3.690463	2.956117	1.197583	3.912225	3.784324	2.160647	4.044449	3.784324	2.160647	4.044449	9.782846	5.016251	13.077255	357	175	638
gene1103	NC_008261.1	1270941	1272200	+	protein_coding	CPF_RS05470	CPF_1113	.	WP_003456161.	sugar ABC trans	-62.658444	4.859139	-417.742675	5.572270	6.666981	9.962290	8.687330	2.717893	11.424364	8.687330	2.717893	11.424364	352.086878	8.005527	2649.476467	9385	204	94416
gene1104	NC_008261.1	1272288	1273193	+	protein_coding	CPF_RS05475	CPF_1114	.	WP_003451894.	sugar ABC trans	-36.217705	2.432706	-187.997044	2.864843	5.190750	7.233206	6.142141	0.963518	8.518084	6.142141	0.963518	8.518084	54.574479	1.364401	363.802698	1046	25	9322
gene1105	NC_008261.1	1273190	1274047	+	protein_coding	CPF_RS05480	CPF_1115	.	WP_003451790.	carbohydrate AE	-27.551485	2.171092	-165.983758	2.624064	6.024494	6.795472	5.622423	0.838365	8.213263	5.622423	0.838365	8.213263	37.794007	1.152585	294.524555	686	20	7147
gene1106	NC_008261.1	1274067	1274936	+	protein_coding	CPF_RS05485	CPF_1116	.	WP_011590576.	PIG-L family dea	-28.774344	2.520794	-180.643188	3.033007	6.277926	7.123005	5.920076	1.073365	8.570364	5.920076	1.073365	8.570364	45.640053	1.591362	373.410691	840	28	9188
gene1107	NC_008261.1	1274949	1277291	+	protein_coding	CPF_RS05490	CPF_1117	.	WP_003456339.	glycoside hydrol	-20.572578	3.382185	-153.040157	4.095809	7.439036	7.515337	6.205919	1.843269	9.101035	6.205919	1.843269	9.101035	58.124212	3.756453	552.414413	2881	178	36606
gene1108	NC_008261.1	1277372	1278247	+	protein_coding	CPF_RS05495	CPF_1118	.	WP_003456288.	hypothetical pro	-12.116997	3.215886	-118.534621	4.067682	9.782508	6.937303	5.484592	1.885632	8.774796	5.484592	1.885632	8.774796	34.211433	3.894711	427.320592	634	69	10587
gene1109	NC_008261.1	1278316	1279605	+	protein_coding	CPF_RS05500	CPF_1119	.	WP_003456415.	endo-beta-gala	-10.375872	5.804663	-346.203356	7.466193	33.366194	9.945960	7.732581	4.357421	12.792897	7.732581	4.357421	12.792897	173.213605	29.552549	6293.274066	4727	771	229605
gene1110	NC_008261.1	1280108	1280578	+	protein_coding	CPF_RS05505	CPF_1121	.	WP_003456199.	hypothetical pro	-1.790747	1.256888	-4.797693	1.693073	2.679157	2.351351	1.745575	0.905013	3.167354	1.745575	0.905013	3.167354	1.806499	1.259768	7.056542	18	12	94
gene1111	NC_008261.1	1280868	1281395	+	protein_coding	CPF_RS05510	CPF_1122	.	WP_003456144.	hypothetical pro	1.506051	5.068004	1.391414	5.128181	1.082388	4.837992	4.781220	5.371990	4.895438	4.781220	5.371990	4.895438	20.949229	58.997948	24.777234	234	630	370
gene1112	NC_008261.1	1281873	1286585	+	protein_coding	CPF_RS05515	CPF_1123	.	WP_011590578.	peptidase S8	1.377166	9.628500	1.757034	9.446802	-1.275833	9.223022	9.400415	9.862118	9.048976	9.400415	9.862118	9.048976	585.244002	1000.531951	524.672575	58351	95367	69936
gene1113	NC_008261.1	1287175	1287411	+	protein_coding	CPF_RS05520	CPF_1124	.	WP_003451889.	hypothetical pro	-3.411451	7.246675	-3.729213	7.303326	1.093146	8.249724	8.185731	6.415346	8.314217	8.185731	6.415346	8.314217	248.915733	118.294620	322.695707	1248	567	2163
gene1114	NC_008261.1	1287671	1289680	-	protein_coding	CPF_RS05525	CPF_1125	.	WP_003456318.	ferrous iron trar	2.180155	0.628450	1.922005	0.806527	1.134312	0.360642	0.281014	1.405445	0.462832	0.281014	1.405445	0.462832	0.164622	2.386195	0.316637	7	97	18
gene1117	NC_008261.1	1290415	1290681	-	protein_coding	CPF_RS05540	CPF_1128	.	WP_003456159.	HTH domain-co	1.464186	0.798287	1.331041	0.889509	1.100030	0.634347	0.569293	1.119391	0.706836	0.569293	1.119391	0.706836	0.354083	1.666716	0.529705	2	9	4
gene1118	NC_008261.1	1291849	1292337	-	protein_coding	CPF_RS05545	CPF_1129	.	WP_003456337.	N-acetyltransfe	1.243584	0.759905	1.247145	0.757368	-1.002863	0.616687	0.618753	0.933257	0.614628	0.618753	0.933257	0.614628	0.386667	1.314513	0.433838	4	13	6
gene1119	NC_008261.1	1292920	1293528	+	protein_coding	CPF_RS05550	CPF_1131	.	WP_003456400.	membrane prote	2.404591	1.620600	1.386724	2.123800	1.734008	1.450599	1.106904	2.372695	1.901014	1.106904	2.372695	1.901014	0.853811	6.008205	2.438468	11	74	42
gene1120	NC_008261.1	1293709	1294302	+	protein_coding	CPF_RS05555	CPF_1132	.	WP_011590582.	hypothetical pro	1.112680	7.499482	1.442987	7.307581	-1.296858	7.232918	7.422858	7.576896	7.047838	7.422858	7.576896	7.047838	138.149521	240.403632	121.014092	1736	2888	2033
gene1121	NC_008261.1	1295026	1295997	+	protein_coding	CPF_RS05560	CPF_1133	.	WP_011590583.	DUF4003 domai	-3.974669	7.228171	-2.782954	7.000456	-1.428220	8.030584	8.291808	6.300973	7.777590	8.291808	6.300973	7.777590	268.495858	109.065852	215.529690	5521	2144	5925
gene1122	NC_008261.1	1296246	1296425	+	protein_coding	CPF_RS05565	CPF_1134	.	WP_003456349.	DUF4177 domai	1.112553	6.413062	1.161500	6.381552	-1.043995	6.305453	6.336587	6.490460	6.274472	6.336587	6.490460	6.274472	64.602409	124.164147	67.179786	246	452	342
gene1123	NC_008261.1	1296514	1297332	-	protein_coding	CPF_RS05570	CPF_1135	.	WP_011590584.	4Fe-4S dicluste	-1.494592	4.755705	-3.892814	5.366237	2.604600	5.703286	5.054406	4.474655	6.435468	5.054406	4.474655	6.435468	25.453107	31.394222	76.155223	441	520	1764
gene1124	NC_008261.1	1297629	1297955	+	protein_coding	CPF_RS05575	CPF_1136	.	WP_003456232.	transcriptional r	1.737046	3.889724	2.379724	3.629500	-1.369983	3.276810	3.511748	4.308383	3.057589	3.511748	4.308383	3.057589	7.806064	28.578823	6.595794	54	189	61
gene1125	NC_008261.1	1298210	1300132	+	protein_coding	CPF_RS05580	CPF_1137	.	WP_011590585.	FUSC family pro	1.118425	2.392366	-1.079302	2.528913	1.207119	2.445010	2.312994	2.474463	2.584562	2.312994	2.474463	2.584562	2.949769	6.505363	4.467988	120	253	243
gene1126	NC_008261.1	1300286	1300687	+	protein_coding	CPF_RS05585	CPF_1138	.	WP_011590586.	hypothetical pro	-3.276594	5.354317	-3.137987	5.327661	-1.044171	6.247168	6.278424	4.566227	6.216067	6.278424	4.566227	6.216067	61.733281	33.086933	64.470782	525	269	733
gene1127	NC_008261.1	1300967	1301650	+	protein_coding	CPF_RS05590	CPF_1139	.	WP_003456343.	DNA-binding res	1.181914	0.758861	-1.078529	0.941896	1.274728	0.804067	0.647816	0.888941	0.998006	0.647816	0.888941	0.998006	0.414650	1.228919	0.878775	6	17	17
gene1128	NC_008261.1	1301654	1302910	+	protein_coding	CPF_RS05595	CPF_1140	.	WP_011590587.	sensor histidine	1.281903	2.050512	1.360794	2.002952	-1.061543	1.835593	1.879180	2.237467	1.793017	1.879180	2.237467	1.793017	2.030694	5.349755	2.137782	54	136	76
gene1129	NC_008261.1	1303158	1303844	+	protein_coding	CPF_RS05600	CPF_1141	.	WP_003456340.	VTC domain-co	-1.019624	0.445235	1.028735	0.410493	-1.048922	0.423621	0.459474	0.431437	0.390566	0.459474	0.431437	0.390566	0.275226	0.503816	0.257335	4	7	5
gene1130	NC_008261.1	1303940	1304629	+	protein_coding	CPF_RS05605	CPF_1142	.	WP_003456146.	DUF4956 domai	1.108466	0.298985	-1.079931	0.434298	1.197066	0.339600	0.233792	0.382356	0.493295	0.233792	0.382356	0.493295	0.137015	0.429964	0.358702	2	6	7
gene1131	NC_008261.1	1304725	1306764	+	protein_coding	CPF_RS05610	CPF_1143	.	WP_011590588.	hypothetical pro	-1.183728	0.706293	-1.432749	0.814093	1.210370	0.966323	0.838365	0.595027	1.113812	0.838365	0.595027	1.113812	0.579290	0.775622	1.039935	25	32	60
gene1132	NC_008261.1	1306960	1307829	+	protein_coding	CPF_RS05615	CPF_1144	.	WP_011590589.	cation transport	1.002725	1.902976	1.135474	1.810976	-1.132388	1.809108	1.901014	1.904940	1.721646	1.901014	1.904940	1.721646	2.064669	3.978406	1.991415	38	70	49
gene1133	NC_008261.1	1308049	1309422	+	protein_coding	CPF_RS05620	CPF_1145	.	WP_011590590.	pyridine nucleot	-1.408533	3.384655	-1.344260	3.353188	-1.047813	3.606912	3.640760	3.146566	3.573378	3.640760	3.146566	3.573378	8.669617	11.479798	9.418448	252	319	366
gene1134	NC_008261.1	1309503	1310750	-	protein_coding	CPF_RS05625	CPF_1146	.	WP_011590591.	hypothetical pro	-1.071715	0.264509	1.125680	0.103038	-1.206408	0.124321	0.319146	0.219225	0.048429	0.319146	0.219225	0.048429	0.189383	0.237721	0.028332	5	6	1
gene1135	NC_008261.1	1310920	1311867	+	protein_coding	CPF_RS05630	CPF_1147	.	WP_011590592.	hypothetical pro	-1.643428	4.071283	-1.594546	4.051286	-1.030656	4.423544	4.445378	3.728670	4.401816	4.445378	3.728670	4.401816	16.404903	18.411817	17.380513	329	353	466
gene1136	NC_008261.1	1312100	1315303	+	protein_coding	CPF_RS05635	CPF_1148	.	WP_041702650.	ATP-dependent	1.522426	2.731788	1.360060	2.821204	1.119381	2.525416	2.445375	3.051747	2.608077	2.445375	3.051747	2.608077	3.393294	10.617596	4.557667	230	688	413
gene1137	NC_008261.1	1315800	1316468	+	protein_coding	CPF_RS05640	CPF_1150	.	WP_004457147.	hypothetical pro	-1.083954	3.871972	1.044207	3.782924	-1.131872	3.840165	3.930560	3.814257	3.751849	3.930560	3.814257	3.751849	10.810640	19.734014	10.676042	153	267	202
gene1138	NC_008261.1	1316688	1317374	+	protein_coding	CPF_RS05645	CPF_1151	.	WP_003456143.	RNA pseudourid	1.824840	3.373244	1.803530	3.382863	1.011816	2.975610	2.967148	3.834919	2.984095	2.967148	3.834919	2.984095	5.160486	19.936702	6.124565	75	277	119
gene1139	NC_008261.1	1317389	1318063	+	protein_coding	CPF_RS05650	CPF_1152	.	WP_003456279.	hypothetical pro	1.831434	2.007892	1.959343	1.946533	-1.069841	1.568847	1.618300	2.491274	1.520904	1.618300	2.491274	1.520904	1.540654	6.592787	1.623839	22	90	31
gene1140	NC_008261.1	1318654	1321323	+	protein_coding	CPF_RS05655	CPF_1153	.	WP_011590594.	magnesium-trar	-24.017148	6.103293	-12.142451	5.752475	-1.977949	8.306253	8.812814	4.226821	7.828809	8.812814	4.226821	7.828809	383.259233	26.926718	222.767269	21648	1454	16822
gene1141	NC_008261.1	1321433	1322194	+	protein_coding	CPF_RS05660	CPF_1154	.	WP_011590595.	3-oxoacyl-ACP	-16.853838	7.538334	-9.547984	7.217693	-1.765172	9.427525	9.846337	5.771332	9.026528	9.846337	5.771332	9.026528	809.422150	79.360020	523.360136	13048	1223	11279
gene1142	NC_008261.1	1322470	1323156	+	protein_coding	CPF_RS05665	CPF_1155	.	WP_011590596.	MgtC/SapB fami	-3.649817	4.158035	-3.812331	4.183109	1.044526	5.226867	5.195537	3.327713	5.258386	5.195537	3.327713	5.258386	27.660207	13.387099	32.938836	402	186	640
gene1143	NC_008261.1	1323322	1324446	-	protein_coding	CPF_RS05670	CPF_1156	.	WP_025648860.	hypothetical pro	1.317151	0.662862	1.238352	0.720179	1.063632	0.535950	0.493295	0.890716	0.582294	0.493295	0.890716	0.582294	0.294125	1.230653	0.408579	7	28	13
gene1144	NC_008261.1	1324726	1325232	+	protein_coding	CPF_RS05675	CPF_1157	.	WP_003474578.	N-acetyltransfe	1.195289	2.422087	1.341255	2.332796	-1.122118	2.212150	2.296823	2.554183	2.130599	2.296823	2.554183	2.130599	2.890280	6.924376	3.138265	31	71	45
gene1145	NC_008261.1	1325410	1326423	+	protein_coding	CPF_RS05680	CPF_1158	.	WP_011590598.	alpha/beta hydro	1.616347	5.685439	1.811412	5.597410	-1.120682	5.266782	5.349612	6.042349	5.185234	5.349612	6.042349	5.185234	31.047196	95.332084	30.999081	666	1955	889
gene1146	NC_008261.1	1326587	1327231	-	protein_coding	CPF_RS05685	CPF_1159	.	WP_003480970.	DedA family prot	1.726344	3.911569	2.266516	3.688038	-1.312899	3.335334	3.537488	4.325209	3.144732	3.537488	4.325209	3.144732	7.914986	28.900937	6.961920	108	377	127
gene1147	NC_008261.1	1327488	1328204	+	protein_coding	CPF_RS05690	CPF_1160	.	WP_003456327.	nitrite reductase	-1.044639	1.422784	-1.099221	1.458275	1.052250	1.490921	1.454635	1.391630	1.528112	1.454635	1.391630	1.528112	1.318551	2.344715	1.627346	20	34	33
gene1148	NC_008261.1	1328241	1328576	+	protein_coding	CPF_RS05695	CPF_1161	.	WP_003456142.	hypothetical pro	-1.256572	0.258313	-1.122210	0.208850	-1.119730	0.380912	0.471124	0.141631	0.307973	0.471124	0.141631	0.307973	0.281369	0.147160	0.210463	2	1	2
gene1149	NC_008261.1	1328592	1328870	+	protein_coding	CPF_RS05700	CPF_1162	.	WP_003456354.	hypothetical pro	-1.152148	0.422895	-1.153764	0.423690	1.001402	0.538233	0.537224	0.332897	0.539245	0.537224	0.332897	0.539245	0.338853	0.354451	0.380191	2	2	3
gene1150	NC_008261.1	1328955	1329947	+	protein_coding	CPF_RS05705	CPF_1163	.	WP_003480974.	hypothetical pro	1.245356	0.969928	1.137823	1.043751	1.094507	0.887231	0.824478	1.141036	0.954760	0.824478	1.141036	0.954760	0.571239	1.742806	0.818962	12	35	23
gene1151	NC_008261.1	1330169	1331968	+	protein_coding	CPF_RS05710	CPF_1164	.	WP_011590600.	PAS domain S-b	-1.275344	2.760364	-1.281158	2.763441	1.004558	2.944656	2.941377	2.590490	2.947938	2.941377	2.590490	2.947938	5.094661	7.197125	5.932250	194	262	302
gene1152	NC_008261.1	1332038	1332583	+	protein_coding	CPF_RS05715	CPF_1165	.	WP_003456285.	NAD(P)H nitrore	-1.768154	3.386224	-1.517562	3.287104	-1.165128	3.710330	3.822212	2.999968	3.601723	3.822212	2.999968	3.601723	10.042722	10.233309	9.519403	116	113	147
gene1153	NC_008261.1	1332864	1333490	+	protein_coding	CPF_RS05720	CPF_1166	.	WP_003451994.	hypothetical pro	-2.823524	6.999348	-3.214120	7.082849	1.138337	7.880941	7.788031	6.290534	7.974958	7.788031	6.290534	7.974958	182.521217	108.670573	242.936723	2421	1378	4308
gene1154	NC_008261.1	1333517	1333930	+	protein_coding	CPF_RS05725	CPF_1167	.	WP_011590601.	hypothetical pro	-6.001966	7.092860	-6.459746	7.136955	1.076272	8.555275	8.502418	5.916983	8.608461	8.502418	5.916983	8.608461	306.798642	88.023256	382.359397	2687	737	4477
gene1155	NC_008261.1	1334008	1334829	+	protein_coding	CPF_RS05730	CPF_1168	.	WP_041702653.	MBL fold hydrola	-2.864288	8.389719	-3.441458	8.509840	1.201505	9.314558	9.183078	7.664901	9.447921	9.183078	7.664901	9.447921	498.233281	254.929102	699.843204	8664	4238	16270
gene1156	NC_008261.1	1335411	1336115	+	protein_coding	CPF_RS05735	CPF_1170	.	WP_003463638.	aquaporin family	4.383041	9.310797	3.922494	9.400140	1.117412	8.385350	8.305652	10.437584	8.465813	8.305652	10.437584	8.465813	269.673988	1547.481310	352.574763	4022	22064	7030
gene1157	NC_008261.1	1336302	1337534	+	protein_coding	CPF_RS05740	CPF_1171	.	WP_003477608.	sensor histidine	1.966654	4.624929	1.366685	4.907950	1.438995	4.417455	4.162718	5.138461	4.687780	4.162718	5.138461	4.687780	12.804703	49.846920	21.478490	334	1243	749
gene1158	NC_008261.1	1337554	1338306	+	protein_coding	CPF_RS05745	CPF_1172	.	WP_003451987.	DNA-binding res	1.973393	4.528012	1.339894	4.829154	1.472798	4.334437	4.064145	5.044824	4.622705	4.064145	5.044824	4.622705	11.990147	45.899978	20.237987	191	699	431
gene1159	NC_008261.1	1338644	1340308	+	protein_coding	CPF_RS05750	CPF_1173	pduC	WP_003451947.	propanediol dehy	-1.910728	3.992970	-1.489609	3.829826	-1.282704	4.303915	4.487254	3.553132	4.128066	4.487254	3.553132	4.128066	16.778740	16.303783	14.185585	591	549	668
gene1160	NC_008261.1	1340322	1340888	+	protein_coding	CPF_RS05755	CPF_1174	.	WP_003451972.	propanediol dehy	-1.770865	3.729274	-1.471554	3.607685	-1.203398	4.028445	4.164216	3.339761	3.897102	4.164216	3.339761	3.897102	12.838780	13.516957	11.910646	154	155	191
gene1161	NC_008261.1	1340908	1341333	+	protein_coding	CPF_RS05760	CPF_1175	.	WP_003451918.	propanediol dehy	-2.179392	2.886747	-1.508484	2.659039	-1.444756	3.226590	3.502900	2.378975	2.972075	3.502900	2.378975	2.972075	7.767380	6.035650	6.058963	70	52	73
gene1162	NC_008261.1	1341350	1343200	+	protein_coding	CPF_RS05765	CPF_1176	.	WP_003456360.	diol dehydratase	-1.946084	3.994870	-1.584287	3.861027	-1.228366	4.353027	4.503925	3.543351	4.207185	4.503925	3.543351	4.207185	17.033564	16.161410	15.071469	667	605	789
gene1163	NC_008261.1	1343193	1343543	+	protein_coding	CPF_RS05770	CPF_1177	.	WP_003451934.	glycerol dehydra	-1.712256	2.804896	-1.850081	2.853134	1.080493	3.274916	3.219547	2.443649	3.331237	3.219547	2.443649	3.331237	6.329609	6.339218	7.958019	47	45	79
gene1164	NC_008261.1	1343562	1344074	+	protein_coding	CPF_RS05775	CPF_1178	.	WP_003451906.	ATP:cob(I)alamir	-1.765631	3.925969	-1.666884	3.888384	-1.059240	4.315706	4.357421	3.537237	4.274391	4.357421	3.537237	4.274391	14.927386	16.096423	15.852417	162	167	230
gene1165	NC_008261.1	1344098	1344526	+	protein_coding	CPF_RS05780	CPF_1179	.	WP_003451873.	heme-binding pr	-4.392645	4.615463	-2.948629	4.380892	-1.489725	5.509840	5.804860	3.669770	5.229814	5.804860	3.669770	5.229814	42.311658	17.749810	32.143438	384	154	390
gene1166	NC_008261.1	1344620	1345777	+	protein_coding	CPF_RS05785	CPF_1180	.	WP_003451991.	alcohol dehydrog	-2.717168	6.817822	-2.102046	6.649129	-1.292630	7.389422	7.576896	6.134793	7.206587	7.576896	6.134793	7.206587	155.729931	99.788486	134.011490	3815	2337	4389
gene1167	NC_008261.1	1346362	1347831	+	protein_coding	CPF_RS05790	CPF_1181	.	WP_003456207.	amino acid perm	-1.259408	10.459447	-1.539381	10.601006	1.222305	10.770972	10.627143	10.294397	10.916747	10.627143	10.294397	10.916747	1347.968325	1397.536209	1894.744165	41919	41548	78774
gene1168	NC_008261.1	1348121	1348795	+	protein_coding	CPF_RS05795	CPF_1182	.	WP_003456389.	ferric reductase	5.982802	2.645831	4.358164	2.989289	1.372780	1.867945	1.653325	4.234146	2.110426	1.653325	4.234146	2.110426	1.610683	27.176931	3.090532	23	371	59
gene1169	NC_008261.1	1349143	1349655	+	protein_coding	CPF_RS05800	CPF_1183	.	WP_011590604.	N-acetyltransfe	2.985141	5.100140	3.855591	4.880070	-1.291594	4.183248	4.371894	5.949693	4.002741	4.371894	5.949693	4.002741	15.111675	89.927922	12.750857	164	933	185
gene1170	NC_008261.1	1349723	1350502	-	protein_coding	CPF_RS05805	CPF_1184	.	WP_003456323.	hypothetical pro	1.261920	1.942470	1.387378	1.866451	-1.099418	1.712160	1.781895	2.117515	1.645154	1.781895	2.117515	1.645154	1.878682	4.754413	1.858550	31	75	41
gene1173	NC_008261.1	1351938	1352282	-	protein_coding	CPF_RS05820	CPF_1187	.	WP_003456325.	DUF1622 domai	-1.160730	1.713820	-1.204890	1.738934	1.038045	1.851444	1.824705	1.609673	1.878575	1.824705	1.609673	1.878575	1.918205	3.009750	2.357185	14	21	23
gene1174	NC_008261.1	1352466	1353455	+	protein_coding	CPF_RS05825	CPF_1188	.	WP_003456141.	alpha/beta hydro	-1.241344	3.523397	-1.089101	3.431911	-1.139788	3.587174	3.682798	3.370895	3.494032	3.682798	3.370895	3.494032	9.024283	13.984699	8.821588	189	280	247
gene1175	NC_008261.1	1353512	1354168	-	protein_coding	CPF_RS05830	CPF_1189	.	WP_003464694.	pentapeptide rep	5.125327	1.630037	4.371311	1.841054	1.172492	0.940616	0.832805	3.190449	1.062383	0.832805	3.190449	1.062383	0.575587	11.966359	0.968706	8	159	18
gene1176	NC_008261.1	1354425	1355306	-	protein_coding	CPF_RS05835	CPF_1190	.	WP_003456226.	hypothetical pro	1.375351	0.166641	1.306352	0.256759	1.052818	0.083268	0.054042	0.513842	0.128299	0.054042	0.513842	0.128299	0.053594	0.616672	0.080176	1	11	2
gene1177	NC_008261.1	1356064	1357371	+	protein_coding	CPF_RS05840	CPF_1191	.	WP_003456155.	dicarboxylate/ar	180.466860	4.586449	41.104399	6.455733	4.390451	3.061693	2.175167	9.670757	4.309537	2.175167	9.670757	4.309537	2.602021	905.071653	16.219166	72	23942	600
gene1178	NC_008261.1	1357715	1358404	+	protein_coding	CPF_RS05845	CPF_1192	.	WP_003456183.	DNA-binding res	1.964963	4.046355	2.079697	3.999928	-1.058390	3.547164	3.588335	4.562837	3.506464	3.588335	4.562837	3.506464	8.289386	33.035594	8.967553	121	461	175
gene1179	NC_008261.1	1358614	1359534	+	protein_coding	CPF_RS05850	CPF_1193	.	WP_003456219.	ABC transporte	-1.076312	5.022997	-1.486811	5.248547	1.381394	5.304270	5.076325	4.970229	5.542450	5.076325	4.970229	5.542450	25.713678	44.130868	39.465581	501	822	1028
gene1180	NC_008261.1	1359527	1360279	+	protein_coding	CPF_RS05855	CPF_1194	.	WP_011590607.	ABC transporte	1.187211	2.436647	1.124749	2.477325	1.055534	2.354665	2.316001	2.563578	2.393974	2.316001	2.563578	2.393974	2.950455	7.026177	3.897339	47	107	83
gene1181	NC_008261.1	1360293	1361198	+	protein_coding	CPF_RS05860	CPF_1195	.	WP_003456262.	sensor histidine	1.280083	2.296257	1.184113	2.356219	1.081047	2.180527	2.125037	2.481274	2.237467	2.125037	2.481274	2.237467	2.452199	6.549126	3.395284	47	120	87
gene1182	NC_008261.1	1361302	1361529	-	protein_coding	CPF_RS05865	CPF_1196	.	WP_003450420.	DUF1413 domai	-1.033243	1.239560	-1.112199	1.290625	1.076416	1.315420	1.263374	1.216195	1.369611	1.263374	1.216195	1.369611	1.036624	1.951812	1.395702	5	9	9
gene1183	NC_008261.1	1361829	1363040	+	protein_coding	CPF_RS05870	CPF_1197	.	WP_003456357.	NAD(P)/FAD-de	1.375891	5.100007	1.538397	5.015052	-1.118109	4.793809	4.875016	5.335382	4.713954	4.875016	5.335382	4.713954	22.308970	57.482898	21.850643	572	1409	749
gene1184	NC_008261.1	1363257	1363793	+	protein_coding	CPF_RS05875	CPF_1198	.	WP_003456157.	glutathione perc	1.350704	2.771964	1.401480	2.743030	-1.037592	2.536819	2.563578	2.997290	2.510339	2.563578	2.997290	2.510339	3.785125	10.220661	4.213963	43	111	64
gene1185	NC_008261.1	1364004	1364669	+	protein_coding	CPF_RS05880	CPF_1199	.	WP_003456309.	ABC transporte	-1.875569	1.924211	-2.338260	2.046256	1.246694	2.584796	2.430631	1.523303	2.748739	2.430631	1.523303	2.748739	3.335875	2.746994	5.202797	47	37	98
gene1186	NC_008261.1	1364725	1365642	+	protein_coding	CPF_RS05885	CPF_1200	.	WP_003456383.	hypothetical pro	-2.615085	3.074362	-2.813390	3.116234	1.075831	3.897392	3.845023	2.458165	3.950474	3.845023	2.458165	3.950474	10.144010	6.409654	12.325153	197	119	320
gene1187	NC_008261.1	1365653	1366948	+	protein_coding	CPF_RS05890	CPF_1201	.	WP_003468551.	ABC transporte	-2.179425	3.011339	-2.700650	3.137193	1.239157	3.776815	3.625301	2.501354	3.934660	3.625301	2.501354	3.934660	8.498397	6.676723	12.277008	233	175	450
gene1188	NC_008261.1	1367043	1368245	-	protein_coding	CPF_RS05895	CPF_1202	.	WP_011590608.	collagen-like pro	1.110210	1.488945	1.479555	1.252200	-1.332680	1.190380	1.415438	1.566270	1.001107	1.415438	1.566270	1.001107	1.257391	2.918253	0.881740	32	71	30
gene1189	NC_008261.1	1368347	1368937	-	protein_coding	CPF_RS05900	CPF_1203	.	WP_003456331.	metal-depender	-1.030441	0.118034	-1.083268	0.145008	1.051267	0.173997	0.141631	0.098369	0.213759	0.141631	0.098369	0.213759	0.079983	0.083665	0.119654	1	1	2
gene1190	NC_008261.1	1369129	1369347	-	protein_coding	CPF_RS05905	CPF_1204	.	WP_003456139.	hypothetical pro	1.271854	2.120174	2.136371	1.665433	-1.679730	1.534737	1.953792	2.300725	1.205563	1.953792	2.300725	1.205563	2.158450	5.644509	1.130157	10	25	7
gene1191	NC_008261.1	1369560	1370174	-	protein_coding	CPF_RS05910	CPF_1205	.	WP_003456298.	ADP-ribose pyro	-1.673626	2.922735	-1.400177	2.807084	-1.195296	3.186456	3.317738	2.574761	3.060370	3.317738	2.574761	3.060370	6.840707	7.075186	6.611618	89	88	115
gene1192	NC_008261.1	1370553	1371194	-	protein_coding	CPF_RS05915	CPF_1207	.	WP_003456145.	pentapeptide rep	1.152611	0.867451	1.280919	0.777085	-1.111320	0.690707	0.771028	0.975933	0.618753	0.771028	0.975933	0.618753	0.515406	1.386334	0.440595	7	18	8
gene1193	NC_008261.1	1371401	1371832	+	protein_coding	CPF_RS05920	CPF_1208	.	WP_003456274.	MarR family tran	1.266350	4.572719	1.537315	4.425163	-1.213974	4.263390	4.405553	4.746229	4.125816	4.405553	4.746229	4.125816	15.537842	38.114547	14.159482	142	333	173
gene1194	NC_008261.1	1372186	1373922	+	protein_coding	CPF_RS05925	CPF_1210	.	WP_003456394.	potassium-trans	-1.084178	0.765936	-1.181158	0.821216	1.089451	0.886100	0.826452	0.709851	0.950053	0.826452	0.709851	0.950053	0.571486	0.939387	0.814226	21	33	40
gene1195	NC_008261.1	1373936	1376002	+	protein_coding	CPF_RS05930	CPF_1211	.	WP_003456252.	potassium-trans	-1.472173	1.301129	-1.072885	1.101306	-1.372162	1.362465	1.609673	1.051727	1.153222	1.609673	1.051727	1.153222	1.532218	1.530981	1.077668	67	64	63
gene1196	NC_008261.1	1376017	1376613	+	protein_coding	CPF_RS05935	CPF_1212	.	WP_003456358.	potassium-trans	-1.915095	3.906213	-1.773475	3.856734	-1.079854	4.347169	4.402941	3.465525	4.292104	4.402941	3.465525	4.292104	15.519147	15.322431	15.990957	196	185	270
gene1197	NC_008261.1	1376625	1379327	+	protein_coding	CPF_RS05940	CPF_1213	.	WP_003456245.	sensor histidine	-1.778289	4.255368	-1.778289	4.255368	1.000000	4.690825	4.690825	3.860335	4.690825	4.690825	3.860335	4.690825	19.604044	20.451541	21.557390	1121	1118	1648
gene1198	NC_008261.1	1379302	1380015	+	protein_coding	CPF_RS05945	CPF_1214	.	WP_003456175.	DNA-binding res	-2.048848	4.500496	-2.030350	4.494659	-1.009111	5.041001	5.047547	4.012734	5.034462	5.047547	4.012734	5.034462	25.356346	23.060903	28.028718	383	333	566
gene1199	NC_008261.1	1380017	1380388	+	protein_coding	CPF_RS05950	CPF_1215	.	WP_003456405.	hypothetical pro	-1.765911	4.636511	-2.100190	4.749610	1.189295	5.188375	5.064828	4.244416	5.314935	5.064828	4.244416	5.314935	25.668148	27.381331	34.217208	202	206	360
gene1200	NC_008261.1	1380631	1381590	+	protein_coding	CPF_RS05955	CPF_1216	.	WP_003456130.	hypothetical pro	3.460146	2.148069	3.260045	2.211596	1.061380	1.474152	1.431807	3.222640	1.517748	1.431807	3.222640	1.517748	1.280231	12.361475	1.620565	26	240	44
gene1201	NC_008261.1	1381694	1383316	-	protein_coding	CPF_RS05960	CPF_1217	.	WP_003456313.	glutamate/gamm	-11.006954	9.505765	-7.238433	9.250054	-1.520627	11.085655	11.392110	7.931767	10.787444	11.392110	7.931767	10.787444	2283.932950	291.648549	1733.882018	78418	9573	79589
gene1202	NC_008261.1	1384213	1385850	-	protein_coding	CPF_RS05965	CPF_1220	.	WP_011590611.	GGDEF domain-	1.853046	1.088931	2.154624	0.912733	-1.162748	0.613037	0.731380	1.621279	0.513842	0.731380	1.621279	0.513842	0.490593	3.048862	0.366961	17	101	17
gene1203	NC_008261.1	1386055	1390422	+	protein_coding	CPF_RS05970	CPF_1221	.	WP_011590612.	glycoside hydrol	-1.789193	1.390177	-1.713132	1.366706	-1.044399	1.840189	1.871792	1.032483	1.809119	1.871792	1.032483	1.809119	2.002051	1.482924	2.201767	185	131	272
gene1204	NC_008261.1	1390797	1391276	+	protein_coding	CPF_RS05975	CPF_1222	.	WP_003456222.	DUF4352 domai	8.660745	3.350790	13.310271	2.823392	-1.536851	1.801258	2.137725	5.252216	1.517748	2.137725	5.252216	1.517748	2.461982	54.390489	1.620565	25	528	22
gene1205	NC_008261.1	1391813	1392976	+	protein_coding	CPF_RS05980	CPF_1223	.	WP_003464716.	dicarboxylate/ar	29.913209	7.183047	30.046170	7.178574	-1.004445	5.135261	5.138461	10.041172	5.132062	5.138461	10.041172	5.132062	26.558948	1132.795359	30.102716	654	26667	991
gene1206	NC_008261.1	1393308	1394303	+	protein_coding	CPF_RS05985	CPF_1224	.	WP_011590613.	hypothetical pro	4.229731	1.710086	4.156785	1.732264	1.017548	0.973830	0.961362	3.041928	0.986459	0.961362	3.041928	0.986459	0.711898	10.574274	0.851995	15	213	24
gene1207	NC_008261.1	1394596	1396506	+	protein_coding	CPF_RS05990	CPF_1226	.	WP_003456307.	FUSC family prot	-1.825564	2.696262	-1.913176	2.724914	1.047992	3.198801	3.165166	2.296823	3.232793	3.165166	2.296823	3.232793	6.109735	5.640610	7.326887	247	218	396
gene1208	NC_008261.1	1396754	1398208	-	protein_coding	CPF_RS05995	CPF_1227	.	WP_011590614.	sodium:proton a	1.127992	5.206392	1.328968	5.084705	-1.178172	5.000565	5.120239	5.293995	4.883688	5.120239	5.293995	4.883688	26.380264	55.766818	24.641093	812	1641	1014
gene1209	NC_008261.1	1398237	1398857	-	protein_coding	CPF_RS06000	CPF_1228	.	WP_003474694.	methyltransfera	1.307896	0.962430	1.260737	0.994254	1.037406	0.814149	0.788090	1.175338	0.841070	0.788090	1.175338	0.841070	0.532835	1.831330	0.683242	7	23	12
gene1210	NC_008261.1	1399095	1399880	+	protein_coding	CPF_RS06005	CPF_1229	.	WP_011590615.	PHP domain-co	1.928758	3.615765	2.381415	3.438097	-1.234689	3.016940	3.172844	4.120516	2.868697	3.172844	4.120516	2.868697	6.134282	24.911674	5.533088	102	396	123
gene1211	NC_008261.1	1400550	1401569	-	protein_coding	CPF_RS06010	CPF_1232	.	WP_011590617.	lactonase family	1.110975	5.613746	1.858364	5.224065	-1.672733	5.153900	5.538346	5.690172	4.796139	5.538346	5.690172	4.796139	35.823287	74.653613	23.155881	773	1540	668
gene1212	NC_008261.1	1401770	1403710	+	protein_coding	CPF_RS06015	CPF_1233	.	WP_011590618.	GTP-binding pro	-1.246322	4.122139	-1.166728	4.076077	-1.068220	4.236165	4.284037	3.966360	4.188828	4.284037	3.966360	4.188828	13.905822	22.366563	14.791612	571	878	812
gene1213	NC_008261.1	1403875	1404228	+	protein_coding	CPF_RS06020	CPF_1234	.	WP_011590619.	hypothetical pro	1.924935	2.864853	1.514014	3.062175	1.271412	2.598585	2.431136	3.375946	2.777567	2.431136	3.375946	2.777567	3.338281	14.107446	5.293679	25	101	53
gene1214	NC_008261.1	1404332	1406329	-	protein_coding	CPF_RS06025	CPF_1235	.	WP_011590620.	potassium trans	3.617036	4.130049	3.644614	4.123198	-1.007624	3.300006	3.305489	5.160297	3.294531	3.305489	5.160297	3.294531	6.813702	50.708032	7.715712	288	2049	436
gene1215	NC_008261.1	1406857	1407369	-	protein_coding	CPF_RS06030	CPF_1236	.	WP_011590621.	ferritin	-1.165811	9.058356	1.139060	8.853944	-1.327930	8.962775	9.169699	8.948365	8.760520	9.169699	8.948365	8.760520	493.617320	592.097777	419.468736	5357	6143	6086
gene1216	NC_008261.1	1407519	1408337	-	protein_coding	CPF_RS06035	CPF_1237	.	WP_011590622.	AraC family tran	-1.804777	0.900066	-2.063138	0.959223	1.143154	1.515100	1.421660	0.569840	1.614680	1.421660	0.569840	1.614680	1.269769	0.724482	1.813220	22	12	42
gene1217	NC_008261.1	1408498	1410330	+	protein_coding	CPF_RS06040	CPF_1238	.	WP_011590623.	glycosyl hydrola	-51.455088	8.495391	-27.208422	8.157801	-1.891146	11.332030	11.800978	6.115736	10.881717	11.800978	6.115736	10.881717	3114.227560	98.945750	1830.792390	120761	3668	94911
gene1218	NC_008261.1	1410448	1410915	+	protein_coding	CPF_RS06045	CPF_1239	.	WP_011590624.	hypothetical pro	-22.938335	10.814229	-15.124889	10.567280	-1.516595	13.003782	13.307670	8.787981	12.706834	13.307670	8.787981	12.706834	10162.758823	527.845541	6276.384066	100617	4996	83075
gene1219	NC_008261.1	1411035	1411805	-	protein_coding	CPF_RS06050	CPF_1240	.	WP_011590625.	Tim44 domain-c	1.170461	4.782253	1.121555	4.813677	1.043605	4.700749	4.670062	4.897138	4.731637	4.670062	4.897138	4.731637	19.435289	42.327229	22.058487	317	660	481
gene1220	NC_008261.1	1411936	1413357	-	protein_coding	CPF_RS06055	CPF_1241	.	WP_011590626.	spore germinatic	-1.265184	0.738668	-1.290537	0.749979	1.020040	0.941782	0.927578	0.588231	0.956203	0.927578	0.588231	0.956203	0.664839	0.764986	0.820539	20	22	33
gene1221	NC_008261.1	1413590	1415098	-	protein_coding	CPF_RS06060	CPF_1242	.	WP_003448442.	YfcC family prote	-6.741385	9.050025	-3.964704	8.714737	-1.700350	10.140492	10.530635	7.777590	9.764803	10.530635	7.777590	9.764803	1280.081862	266.988196	891.465988	40864	8148	38046
gene1222	NC_008261.1	1415500	1417371	+	protein_coding	CPF_RS06065	CPF_1243	.	WP_011590627.	PAS domain-co	1.001736	3.614322	-1.220896	3.756786	1.223015	3.755486	3.613071	3.615574	3.903514	3.613071	3.615574	3.903514	8.459118	17.221542	11.955917	335	652	633
gene1223	NC_008261.1	1417595	1418662	+	protein_coding	CPF_RS06070	CPF_1244	.	WP_003460396.	PTS sugar trans	-9.155544	2.106252	-7.384244	2.027757	-1.239876	4.082713	4.240756	1.046110	3.930560	4.240756	1.046110	3.930560	13.410886	1.527823	12.216312	303	33	369
gene1224	NC_008261.1	1418694	1419374	+	protein_coding	CPF_RS06075	CPF_1245	.	WP_011590628.	L-serine ammor	-11.130631	2.293723	-10.090556	2.258287	-1.103074	4.544869	4.616185	1.139721	4.474655	4.616185	1.139721	4.474655	18.533193	1.742587	18.275975	267	24	352
gene1225	NC_008261.1	1419378	1420256	+	protein_coding	CPF_RS06080	CPF_1246	.	WP_011590629.	L-serine ammor	-14.876616	2.479721	-11.731786	2.394987	-1.268061	4.926247	5.100537	1.205563	4.757913	5.100537	1.205563	4.757913	26.081885	1.912583	22.324879	485	34	555
gene1226	NC_008261.1	1420324	1421058	-	protein_coding	CPF_RS06085	CPF_1247	.	WP_003448449.	tRNA 2-thiocyti	1.036292	4.427457	1.028617	4.432847	1.007462	4.407175	4.401816	4.453247	4.412541	4.401816	4.453247	4.412541	15.499433	30.743913	17.510521	241	457	364
gene1227	NC_008261.1	1421265	1422011	+	protein_coding	CPF_RS06090	CPF_1248	.	WP_011590630.	tRNA pseudouri	1.892103	4.308097	3.138015	3.880951	-1.658480	3.488624	3.872590	4.792581	3.142728	3.872590	4.792581	3.142728	10.377897	39.252233	6.957957	164	593	147
gene1228	NC_008261.1	1422078	1423145	-	protein_coding	CPF_RS06095	CPF_1249	.	WP_011590631.	nitronate mono	3.863600	9.090610	1.631256	9.758343	2.368482	8.767719	8.167771	10.117717	9.411734	8.167771	10.117717	9.411734	247.326842	1189.294254	685.735711	5588	25688	20713
gene1229	NC_008261.1	1423346	1424056	+	protein_coding	CPF_RS06100	CPF_1250	.	WP_011590632.	Crp/Fnr family t	1.021713	5.582312	-1.061454	5.640686	1.084502	5.625051	5.566839	5.597829	5.683871	5.566839	5.597829	5.683871	36.699114	69.961427	43.911282	552	1006	883
gene1230	NC_008261.1	1424113	1425024	-	protein_coding	CPF_RS06105	CPF_1251	.	WP_003481127.	radical SAM prot	1.807863	3.733423	2.232402	3.558782	-1.234830	3.174836	3.330636	4.184921	3.026324	3.330636	4.184921	3.026324	6.893550	26.241026	6.319428	133	484	163
gene1231	NC_008261.1	1425168	1426202	+	protein_coding	CPF_RS06110	CPF_1252	.	WP_003477685.	glucanase	-2.891470	8.193089	-3.080867	8.234671	1.065502	9.040362	8.994711	7.462908	9.086245	8.994711	7.462908	9.086245	431.824513	224.393613	540.854498	9455	4697	15832
gene1232	NC_008261.1	1426521	1427192	+	protein_coding	CPF_RS06115	CPF_1253	.	WP_011590633.	hypothetical pro	1.398819	2.578167	1.660379	2.438581	-1.186986	2.220313	2.347405	2.831614	2.100102	2.347405	2.831614	2.100102	3.024721	8.903205	3.051713	43	121	58
gene1233	NC_008261.1	1427208	1428884	+	protein_coding	CPF_RS06120	CPF_1254	.	WP_011590634.	hypothetical pro	1.594279	2.931990	1.644833	2.906630	-1.031710	2.592163	2.614780	3.287683	2.569742	2.614780	3.287683	2.569742	3.889844	12.914314	4.406545	138	438	209
gene1234	NC_008261.1	1429551	1431209	+	protein_coding	CPF_RS06125	CPF_1255	.	WP_011590635.	glutamate--tRN	3.146728	7.931202	3.987165	7.739398	-1.267083	6.974422	7.147268	8.801120	6.805757	7.147268	8.801120	6.805757	117.420070	529.956321	102.258371	4121	17781	4798
gene1235	NC_008261.1	1431385	1431540	+	protein_coding	CPF_RS06130	CPF_1256	.	WP_003481131.	cytochrome c5	1.679198	12.568833	1.646671	12.583360	1.019753	12.214608	12.200506	12.948279	12.228726	12.200506	12.948279	12.228726	4306.423221	9131.960298	4577.699512	14212	28811	20197
gene1236	NC_008261.1	1431768	1432382	+	protein_coding	CPF_RS06135	CPF_1257	.	WP_003472308.	DUF488 domain	1.281297	4.821109	-1.013427	5.012837	1.298500	4.830370	4.645621	5.003225	5.022467	4.645621	5.003225	5.022467	19.138608	44.702309	27.826287	249	556	484
gene1237	NC_008261.1	1432475	1434097	-	protein_coding	CPF_RS06140	CPF_1258	.	WP_011590636.	aspartate 4-dec	-1.023482	1.078996	1.011344	1.054215	-1.035091	1.070700	1.095868	1.062383	1.046110	1.095868	1.062383	1.046110	0.844628	1.553753	0.936774	29	51	43
gene1238	NC_008261.1	1434229	1435251	-	protein_coding	CPF_RS06145	CPF_1259	.	WP_011590637.	hypothetical pro	-1.393254	1.551556	-1.657217	1.655414	1.189458	1.930218	1.809119	1.330661	2.059424	1.809119	1.330661	2.059424	1.894499	2.175040	2.903278	41	45	84
gene1239	NC_008261.1	1435556	1435735	+	protein_coding	CPF_RS06150	CPF_1260	.	WP_003448677.	DUF896 family p	1.559251	7.847762	1.626445	7.815995	-1.043094	7.503378	7.533874	8.174728	7.473005	7.533874	8.174728	7.473005	149.951119	341.176706	171.878107	571	1242	875
gene1240	NC_008261.1	1435747	1436670	+	protein_coding	CPF_RS06155	CPF_1261	.	WP_003448672.	HPr kinase/pho	1.213793	8.884011	1.295089	8.836377	-1.066977	8.698459	8.745349	9.024871	8.651820	8.745349	9.024871	8.651820	369.258937	614.274328	395.593886	7218	11479	10338
gene1241	NC_008261.1	1436715	1437644	+	protein_coding	CPF_RS06160	CPF_1262	.	WP_011590638.	divalent cation t	-1.681888	2.995236	-1.828444	3.047963	1.087138	3.453407	3.393665	2.643583	3.514200	3.393665	2.643583	3.514200	7.166750	7.549804	9.010532	141	142	237
gene1242	NC_008261.1	1437711	1439117	+	protein_coding	CPF_RS06165	CPF_1263	.	WP_003472255.	aminopeptidase	-3.619651	9.778767	-2.662901	9.575240	-1.359288	10.526866	10.750620	8.894769	10.307768	10.750620	8.894769	10.307768	1443.937240	574.408831	1348.847868	42979	16345	53675
gene1243	NC_008261.1	1439242	1440765	+	protein_coding	CPF_RS06170	CPF_1264	.	WP_011590639.	GGDEF domain-	-1.644976	2.574601	-1.689748	2.591404	1.027218	2.977856	2.958548	2.240481	2.997290	2.958548	2.240481	2.997290	5.117821	5.353395	6.194572	165	165	267
gene1244	NC_008261.1	1440934	1441293	+	protein_coding	CPF_RS06175	CPF_1265	.	WP_003476019.	methylglyoxal sy	1.079148	9.486742	1.004895	9.538324	1.073892	9.483239	9.431954	9.541847	9.534802	9.431954	9.541847	9.534802	596.127917	832.064606	733.968625	4540	6058	7473
gene1245	NC_008261.1	1441486	1441746	+	protein_coding	CPF_RS06180	CPF_1266	.	WP_003458618.	hypothetical pro	-1.130148	6.196604	-1.794971	6.517311	1.588263	6.610795	6.285488	6.108977	6.952938	6.285488	6.108977	6.952938	61.940072	98.702351	113.117808	342	521	835
gene1246	NC_008261.1	1441830	1442210	+	protein_coding	CPF_RS06185	CPF_1267	.	WP_003458553.	RidA family prote	-3.887622	11.004411	-3.343974	10.939434	-1.102766	11.915173	11.985946	10.103255	11.844819	11.985946	10.103255	11.844819	3546.618881	1173.723658	3507.473418	28586	9044	37795
gene1247	NC_008261.1	1442254	1442499	+	protein_coding	CPF_RS06190	CPF_1268	.	WP_003448671.	TSCPD domain-	-3.099570	10.809318	-2.910061	10.767033	-1.065122	11.610513	11.656111	10.024043	11.565093	11.656111	10.024043	11.565093	2889.430154	1129.014702	2917.879403	15037	5617	20301
gene1248	NC_008261.1	1442606	1445242	-	protein_coding	CPF_RS06195	CPF_1269	.	WP_011590640.	FprA family A-ty	-2.939176	10.834532	-2.251887	10.654190	-1.305206	11.446363	11.640115	10.084703	11.255837	11.640115	10.084703	11.255837	2838.246832	1153.756403	2439.834452	158334	61531	181964
gene1249	NC_008261.1	1445447	1446289	-	protein_coding	CPF_RS06200	CPF_1270	.	WP_011590641.	PHP domain-co	-1.998892	8.213691	-1.295557	7.913854	-1.542882	8.409842	8.728472	7.729271	8.102844	8.728472	7.729271	8.102844	363.469154	263.124916	266.965934	6482	4486	6365
gene1250	NC_008261.1	1446556	1447146	+	protein_coding	CPF_RS06205	CPF_1271	.	WP_003458624.	type 1 glutamine	3.495567	4.840184	4.593002	4.596987	-1.313950	3.818859	4.020890	5.826417	3.626979	4.020890	5.826417	3.626979	11.597560	82.911821	9.811635	145	991	164
gene1251	NC_008261.1	1447152	1448498	+	protein_coding	CPF_RS06210	CPF_1272	.	WP_011590642.	aminodeoxychor	4.032989	3.389397	4.764654	3.224235	-1.181420	2.406330	2.529594	4.541444	2.289072	2.529594	4.541444	2.289072	3.684748	32.450019	3.569902	105	884	136
gene1252	NC_008261.1	1448516	1449286	+	protein_coding	CPF_RS06215	CPF_1273	.	WP_041702656.	4-amino-4-deo	2.357487	3.369506	3.929278	2.893571	-1.666723	2.410689	2.807199	4.044449	2.070185	2.807199	4.044449	2.070185	4.536944	23.793033	2.935017	74	371	64
gene1253	NC_008261.1	1449301	1449858	+	protein_coding	CPF_RS06220	CPF_1274	.	WP_011590644.	GTP cyclohydro	1.427724	5.554419	1.921453	5.325318	-1.345815	5.084745	5.303496	5.817213	4.875016	5.303496	5.817213	4.875016	29.734389	82.055381	24.522333	351	926	387
gene1254	NC_008261.1	1449884	1450357	+	protein_coding	CPF_RS06225	CPF_1275	.	WP_003458609.	HD domain-cont	1.774064	4.735981	2.159915	4.578469	-1.217495	4.196113	4.340472	5.167530	4.056556	4.340472	5.167530	4.056556	14.659701	50.802011	13.203222	147	487	177
gene1255	NC_008261.1	1450370	1451179	+	protein_coding	CPF_RS06230	CPF_1276	.	WP_011590645.	dihydropteroate	1.226840	6.089192	1.438523	5.970399	-1.172543	5.827553	5.943504	6.238452	5.713863	5.943504	6.238452	5.713863	46.919906	105.240408	44.830175	804	1724	1027
gene1256	NC_008261.1	1451192	1452013	+	protein_coding	CPF_RS06235	CPF_1277	.	WP_011590646.	2-amino-4-hyd	1.615534	5.126924	1.595216	5.136681	1.012737	4.801702	4.792581	5.484592	4.810840	4.792581	5.484592	4.810840	20.989745	64.123271	23.399797	365	1066	544
gene1258	NC_008261.1	1452669	1453109	+	protein_coding	CPF_RS06245	CPF_1279	.	WP_003477720.	hypothetical pro	1.027199	0.370703	1.199362	0.223850	-1.167604	0.212466	0.351850	0.390566	0.128299	0.351850	0.390566	0.128299	0.214377	0.448489	0.080176	2	4	1
gene1259	NC_008261.1	1453478	1454560	+	protein_coding	CPF_RS06250	CPF_1281	.	WP_011590647.	pyruvate kinase	1.154857	2.018510	1.276239	1.941130	-1.105106	1.843822	1.917323	2.125037	1.773138	1.917323	2.125037	1.773138	2.095072	4.839580	2.089472	48	106	64
gene1260	NC_008261.1	1454557	1455114	-	protein_coding	CPF_RS06255	CPF_1280	.	WP_003448881.	phosphatase PA	1.193085	3.721575	1.208309	3.712098	-1.012761	3.587246	3.596405	3.851102	3.578112	3.596405	3.851102	3.578112	8.386623	20.292314	9.441415	99	229	149
gene1261	NC_008261.1	1455288	1456739	+	protein_coding	CPF_RS06260	CPF_1283	.	WP_041702658.	DUF3502 domai	-2.227701	1.948557	-2.334669	1.973651	1.048017	2.643806	2.610191	1.454635	2.677853	2.610191	1.454635	2.677853	3.874061	2.554024	4.991973	119	75	205
gene1262	NC_008261.1	1456736	1457077	-	protein_coding	CPF_RS06265	CPF_1282	.	WP_003476035.	hypothetical pro	-2.213369	4.676165	-1.730923	4.516498	-1.278721	5.103846	5.284278	4.138034	4.929576	5.284278	4.138034	4.929576	29.301905	25.156686	25.639561	212	174	248
gene1263	NC_008261.1	1457097	1457483	-	protein_coding	CPF_RS06270	CPF_1284	.	WP_003458613.	fluoride efflux tr	-1.285022	4.017301	-1.299133	4.024825	1.010981	4.210139	4.202268	3.840475	4.218024	4.202268	3.840475	4.218024	13.191643	20.059448	15.257751	108	157	167
gene1264	NC_008261.1	1458002	1459450	+	protein_coding	CPF_RS06275	CPF_1285	.	WP_041702659.	DUF3502 domai	-11.577678	7.851323	-12.479226	7.894477	1.077870	9.868206	9.814263	6.280989	9.922446	9.814263	6.280989	9.922446	799.089026	108.105320	1019.812136	24495	3168	41793
gene1265	NC_008261.1	1459669	1460403	+	protein_coding	CPF_RS06280	CPF_1286	.	WP_011590650.	ArsR family tran	-1.888330	7.492374	-1.845394	7.476751	-1.023267	7.948341	7.964949	7.047838	7.931767	7.964949	7.047838	7.931767	215.384237	172.354278	237.835203	3349	2562	4944
gene1266	NC_008261.1	1460615	1461913	+	protein_coding	CPF_RS06285	CPF_1287	.	WP_011590651.	amidohydrolase	-1.228803	1.083033	-1.102082	1.012248	-1.114983	1.160647	1.241810	0.944557	1.084789	1.241810	0.944557	1.084789	1.018908	1.332261	1.007112	28	35	37
gene1268	NC_008261.1	1463088	1464173	+	protein_coding	CPF_RS06295	CPF_1289	.	WP_011590652.	nicotinate-nucl	2.251063	5.891684	2.150819	5.927859	1.046607	5.368142	5.335382	6.505989	5.401102	5.335382	6.505989	5.401102	30.773415	125.663612	35.911265	707	2760	1103
gene1269	NC_008261.1	1464211	1464771	+	protein_coding	CPF_RS06300	CPF_1290	.	WP_011590653.	adenosylcobinar	2.398250	2.740772	2.717435	2.625091	-1.133091	2.089403	2.181478	3.443460	2.001215	2.181478	3.443460	2.001215	2.612071	14.807328	2.773159	31	168	44
gene1270	NC_008261.1	1464755	1465510	+	protein_coding	CPF_RS06305	CPF_1291	.	WP_011590654.	adenosylcobinar	1.696638	3.656037	1.774005	3.620166	-1.045600	3.262207	3.294531	4.057210	3.230200	3.294531	4.057210	3.230200	6.752865	24.134308	7.296050	108	369	156
gene1271	NC_008261.1	1465524	1465904	+	protein_coding	CPF_RS06310	CPF_1292	.	WP_003448817.	hypothetical pro	1.624310	2.094018	1.712647	2.048408	-1.054384	1.734517	1.773138	2.472966	1.696738	1.773138	2.472966	1.696738	1.861026	6.488963	1.948854	15	50	21
gene1272	NC_008261.1	1465909	1466553	+	protein_coding	CPF_RS06315	CPF_1293	.	WP_011590655.	histidine phosph	1.709185	4.742152	2.002324	4.616618	-1.171508	4.255520	4.371235	5.144544	4.142868	4.371235	5.144544	4.142868	15.097103	50.212502	14.362386	206	655	262
gene1273	NC_008261.1	1466556	1467503	+	protein_coding	CPF_RS06320	CPF_1294	.	WP_011590656.	cobalamin biosyr	1.999404	4.139493	2.182511	4.067568	-1.091581	3.606007	3.669770	4.669340	3.543351	3.669770	4.669340	3.543351	8.925464	35.676155	9.287012	179	684	249
gene1274	NC_008261.1	1467500	1468564	+	protein_coding	CPF_RS06325	CPF_1295	.	WP_011590657.	threonine-phos	2.398430	3.005854	2.246440	3.063470	1.067658	2.487111	2.440335	3.702425	2.534785	2.440335	3.702425	2.534785	3.373262	18.292661	4.282774	76	394	129
gene1275	NC_008261.1	1469138	1470055	+	protein_coding	CPF_RS06330	CPF_1296	.	WP_003464144.	ABC transporte	1.846415	6.504134	3.001437	6.117536	-1.625548	5.715599	6.076797	6.961523	5.375870	6.076797	6.961523	5.375870	51.904374	163.580824	35.357782	1008	3037	918
gene1276	NC_008261.1	1470042	1471073	+	protein_coding	CPF_RS06335	CPF_1297	btuC	WP_003458539.	iron ABC transp	2.051159	5.463929	3.092861	5.127919	-1.507860	4.664579	4.970229	6.006668	4.377726	4.970229	6.006668	4.377726	23.635028	92.567323	17.027924	516	1932	497
gene1277	NC_008261.1	1471078	1471833	+	protein_coding	CPF_RS06340	CPF_1298	.	WP_003472318.	ABC transporte	2.059603	4.634840	2.968348	4.329850	-1.441224	3.870251	4.142868	5.185234	3.615574	4.142868	5.185234	3.615574	12.692885	51.865871	9.681297	203	793	207
gene1278	NC_008261.1	1471843	1472496	+	protein_coding	CPF_RS06345	CPF_1299	.	WP_003458530.	ATP-binding pro	1.029124	6.023324	1.356626	5.819899	-1.318234	5.799925	6.002651	6.044068	5.604045	6.002651	6.044068	5.604045	49.366128	95.413953	40.872299	683	1262	756
gene1279	NC_008261.1	1472506	1473969	+	protein_coding	CPF_RS06350	CPF_1300	.	WP_011010197.	cobyric acid syn	-1.018449	5.978420	1.470347	5.680366	-1.497474	5.692910	5.991621	5.965247	5.409090	5.991621	5.965247	5.409090	48.690740	90.887237	36.034024	1508	2691	1492
gene1280	NC_008261.1	1474245	1480142	+	protein_coding	CPF_RS06355	CPF_1301	.	WP_011590658.	DUF4968 domai	-2.970216	1.752107	-2.310882	1.630630	-1.285317	2.517758	2.705323	1.134755	2.343198	2.705323	1.134755	2.343198	4.143543	1.735385	3.740803	517	207	624
gene1281	NC_008261.1	1480235	1480684	+	protein_coding	CPF_RS06360	CPF_1302	.	WP_011590659.	thioredoxin fami	-1.922870	0.894021	-1.470904	0.768670	-1.307271	1.274576	1.482426	0.539165	1.095868	1.482426	0.539165	1.095868	1.365579	0.659279	1.021447	13	6	13
gene1283	NC_008261.1	1481572	1482738	+	protein_coding	CPF_RS06370	CPF_1304	.	WP_004458297.	SAM-dependen	2.146325	3.927763	2.091355	3.949228	1.026284	3.433944	3.415280	4.517148	3.452710	3.415280	4.517148	3.452710	7.250505	32.158901	8.574338	179	759	283
gene1284	NC_008261.1	1483031	1483831	+	protein_coding	CPF_RS06375	CPF_1305	.	WP_003458557.	5'-methylthioad	38.667040	4.712977	19.155269	5.509662	2.018611	3.230990	2.763796	8.036828	3.777159	2.763796	8.036828	3.777159	4.367021	310.194312	10.947228	74	5025	248
gene1285	NC_008261.1	1484182	1485366	-	protein_coding	CPF_RS06380	CPF_1307	.	WP_003448915.	class I SAM-dep	2.044428	9.065600	3.690712	8.602748	-1.805254	8.127152	8.564416	9.596113	7.712214	8.564416	9.596113	7.712214	316.809095	846.797537	203.225193	7942	20294	6811
gene1286	NC_008261.1	1485699	1487054	+	protein_coding	CPF_RS06385	CPF_1308	.	WP_011590661.	thiosulfate sulfu	4.304285	3.384155	4.487500	3.343060	-1.042566	2.461022	2.491274	4.597048	2.431136	2.491274	4.597048	2.431136	3.555712	34.203727	3.963409	102	938	152
gene1288	NC_008261.1	1487787	1488026	+	protein_coding	CPF_RS06395	CPF_1310	.	WP_003448834.	DUF1292 domai	-1.165667	0.727139	-1.394114	0.830711	1.195980	0.966590	0.846077	0.624921	1.104269	0.846077	0.624921	1.104269	0.590876	0.824098	1.031269	3	4	7
gene1289	NC_008261.1	1488217	1489005	+	protein_coding	CPF_RS06400	CPF_1311	.	WP_011590662.	endonuclease	-1.243170	1.696434	-1.940310	1.967552	1.560776	2.158066	1.860697	1.546673	2.502960	1.860697	1.546673	2.502960	1.977075	2.820108	4.167647	33	45	93
gene1290	NC_008261.1	1489189	1490013	+	protein_coding	CPF_RS06405	CPF_1312	.	WP_011590663.	PHP domain-co	1.626174	4.925168	1.308341	5.090823	1.242928	4.741178	4.586900	5.288382	4.900644	4.586900	5.288382	4.900644	18.163161	55.499276	24.986166	317	926	583
gene1291	NC_008261.1	1490139	1491560	-	protein_coding	CPF_RS06410	CPF_1313	.	WP_011010208.	tetratricopeptid	1.810039	8.397105	1.419798	8.579449	1.274857	8.153282	7.979995	8.836016	8.330331	7.979995	8.836016	8.330331	218.632315	550.581133	325.878405	6577	15834	13106
gene1292	NC_008261.1	1491920	1493827	-	protein_coding	CPF_RS06415	CPF_1314	.	WP_011590664.	NAD(+) synthase	-1.399639	6.080112	-1.254437	6.003714	-1.115750	6.247968	6.327474	5.842420	6.169460	6.327474	5.842420	6.169460	64.141600	83.705584	62.561778	2589	3230	3376
gene1293	NC_008261.1	1494141	1494554	+	protein_coding	CPF_RS06420	CPF_1315	.	WP_003448822.	NfeD family prot	2.002554	8.885386	1.309455	9.203877	1.529303	8.699616	8.398575	9.400415	9.011449	8.398575	9.400415	9.011449	286.588981	773.099774	514.310540	2510	6473	6022
gene1294	NC_008261.1	1494574	1495524	+	protein_coding	CPF_RS06425	CPF_1316	.	WP_003477756.	SPFH/Band 7/F	-1.006146	9.022107	-1.369289	9.241621	1.360925	9.246150	9.026528	9.017688	9.471115	9.026528	9.017688	9.471115	438.403665	611.600541	704.255633	8820	11763	18942
gene1295	NC_008261.1	1495704	1496756	+	protein_coding	CPF_RS06430	CPF_1317	.	WP_003475565.	alanine racemas	3.345875	4.275739	2.580132	4.499408	1.296785	3.675090	3.492399	5.234782	3.867338	3.492399	5.234782	3.867338	7.721225	53.531173	11.718771	172	1140	349
gene1296	NC_008261.1	1496989	1498023	+	protein_coding	CPF_RS06435	CPF_1318	.	WP_011590665.	FMN-binding pro	-1.103639	0.685201	-1.070921	0.665342	-1.030551	0.737990	0.760017	0.617749	0.716602	0.760017	0.617749	0.716602	0.502387	0.812155	0.546594	11	17	16
gene1297	NC_008261.1	1498041	1498709	+	protein_coding	CPF_RS06440	CPF_1319	.	WP_003470400.	electron transpo	-1.339028	0.285204	-1.249272	0.258716	-1.071846	0.512636	0.565122	0.143936	0.465024	0.565122	0.143936	0.465024	0.353289	0.147820	0.317110	5	2	6
gene1298	NC_008261.1	1498740	1499315	+	protein_coding	CPF_RS06445	CPF_1320	.	WP_003448909.	electron transpo	-1.395396	0.797605	-1.096488	0.655778	-1.272605	0.882504	1.073365	0.592691	0.725581	1.073365	0.592691	0.725581	0.820661	0.772592	0.552465	10	9	9
gene1299	NC_008261.1	1499332	1500153	+	protein_coding	CPF_RS06450	CPF_1321	.	WP_003470383.	4Fe-4S dicluste	-1.566993	0.575696	-1.485989	0.552857	-1.054512	0.945544	0.984607	0.336608	0.908031	0.984607	0.336608	0.908031	0.747580	0.360919	0.774258	13	6	18
gene1300	NC_008261.1	1500217	1500717	+	protein_coding	CPF_RS06455	CPF_1322	.	WP_003470410.	cytidine deamina	4.041580	6.847913	5.762269	6.544957	-1.425747	5.652518	5.914165	7.929084	5.402447	5.914165	7.929084	5.402447	45.477378	291.641981	35.922377	482	2955	509
gene1301	NC_008261.1	1500889	1501353	+	protein_coding	CPF_RS06460	CPF_1323	.	WP_003470412.	MarR family tran	2.247509	7.958843	2.117157	8.005087	1.061569	7.439063	7.396089	8.564416	7.482287	7.396089	8.564416	7.482287	136.117427	458.198667	172.302654	1339	4309	2266
gene1302	NC_008261.1	1501355	1502329	+	protein_coding	CPF_RS06465	CPF_1324	.	WP_011590666.	3-oxoacyl-ACP	2.274177	8.780781	1.776474	8.969351	1.280164	8.384359	8.208087	9.393432	8.564416	8.208087	9.393432	8.564416	251.864550	771.863167	370.767142	5195	15220	10224
gene1303	NC_008261.1	1502426	1503370	+	protein_coding	CPF_RS06470	CPF_1325	.	WP_003466538.	[acyl-carrier-pr	2.107618	8.449793	1.639084	8.640904	1.285851	8.108418	7.929084	9.004698	8.291808	7.929084	9.004698	8.291808	206.787733	608.681637	315.226787	4134	11633	8425
gene1304	NC_008261.1	1503374	1504114	+	protein_coding	CPF_RS06475	CPF_1326	.	WP_003458601.	3-oxoacyl-[acyl	1.808771	8.622261	1.472165	8.776956	1.228647	8.352563	8.205348	9.060357	8.502418	8.205348	9.060357	8.502418	251.787999	625.647439	358.063153	3947	9376	7504
gene1305	NC_008261.1	1504129	1505370	+	protein_coding	CPF_RS06480	CPF_1327	.	WP_003458580.	beta-ketoacyl-[	1.881754	9.770947	1.572858	9.905543	1.196392	9.454005	9.325545	10.237623	9.584235	9.325545	10.237623	9.584235	554.414567	1316.964856	769.672299	14567	33080	27036
gene1306	NC_008261.1	1505382	1505873	+	protein_coding	CPF_RS06485	CPF_1328	.	WP_003458640.	acetyl-CoA carb	1.785295	8.924269	1.558284	9.026489	1.145680	8.613519	8.515975	9.352138	8.712180	8.515975	9.352138	8.712180	308.023977	748.120477	409.632841	3206	7444	5700
gene1307	NC_008261.1	1505876	1506295	+	protein_coding	CPF_RS06490	CPF_1329	.	WP_003448879.	beta-hydroxyac	1.991741	9.061918	1.548897	9.251550	1.285909	8.758039	8.578523	9.572553	8.941312	8.578523	9.572553	8.941312	320.761088	840.698017	483.138836	2850	7141	5739
gene1308	NC_008261.1	1506307	1507659	+	protein_coding	CPF_RS06495	CPF_1330	.	WP_003458585.	acetyl-CoA carb	2.058659	9.213275	1.713042	9.352501	1.201756	8.838711	8.707133	9.748838	8.972277	8.707133	9.748838	8.972277	355.625935	954.382603	496.890516	10179	26115	19014
gene1309	NC_008261.1	1507652	1508530	+	protein_coding	CPF_RS06500	CPF_1331	.	WP_003458574.	acetyl-CoA carb	2.713507	8.765108	2.013495	8.995710	1.347660	8.286991	8.074557	9.514716	8.505014	8.074557	9.514716	8.505014	234.683186	818.698088	358.646171	4364	14554	8916
gene1310	NC_008261.1	1508523	1509338	+	protein_coding	CPF_RS06505	CPF_1332	.	WP_003458527.	acetyl-CoA carb	2.029932	8.214436	1.803100	8.304894	1.125801	7.804590	7.719582	8.741013	7.890534	7.719582	8.741013	7.890534	172.686316	507.062850	232.295428	2981	8368	5361
gene1311	NC_008261.1	1509437	1509826	-	protein_coding	CPF_RS06510	CPF_1333	.	WP_003470407.	PadR family tran	1.115214	7.106594	1.300396	6.993648	-1.166051	6.916667	7.028369	7.185690	6.806739	7.028369	7.185690	6.806739	106.781842	189.035477	102.356246	881	1491	1129
gene1312	NC_008261.1	1509952	1510164	+	protein_coding	CPF_RS06515	CPF_1334	.	WP_041702661.	hypothetical pro	1.720349	5.273989	1.335843	5.466976	1.287838	5.076335	4.897138	5.679840	5.262089	4.897138	5.679840	5.262089	22.636364	74.052779	33.033796	102	319	199
gene1313	NC_008261.1	1510222	1511040	-	protein_coding	CPF_RS06520	CPF_1335	.	WP_011590668.	AP endonucleas	1.659573	6.257448	3.497843	5.658647	-2.107676	5.337848	5.902702	6.633514	4.827048	5.902702	6.633514	4.827048	44.788233	137.289347	23.571855	776	2274	546
gene1314	NC_008261.1	1511257	1511967	+	protein_coding	CPF_RS06525	CPF_1336	.	WP_003449361.	rRNA pseudouri	3.503908	5.017294	4.021799	4.896882	-1.147804	4.093041	4.193687	6.002651	3.994810	4.193687	6.002651	3.994810	13.097329	92.493736	12.631331	197	1330	254
gene1315	NC_008261.1	1511973	1512524	+	protein_coding	CPF_RS06530	CPF_1337	.	WP_003460730.	16S rRNA (cyto	3.870486	5.182542	4.410198	5.067711	-1.139443	4.202215	4.297434	6.249949	4.109105	4.297434	6.249949	4.109105	14.215270	106.326599	13.899708	166	1187	217
gene1316	NC_008261.1	1512556	1513431	+	protein_coding	CPF_RS06535	CPF_1338	.	WP_003449332.	MurR/RpiR fami	2.930067	5.652094	3.651615	5.466987	-1.246257	4.768131	4.929576	6.480509	4.611974	4.929576	6.480509	4.611974	23.149376	123.501858	20.181382	429	2188	500
gene1317	NC_008261.1	1513648	1514868	+	protein_coding	CPF_RS06540	CPF_1339	.	WP_003460716.	NAD(P)/FAD-de	3.559619	4.620851	4.255604	4.461235	-1.195522	3.663793	3.794878	5.626601	3.537237	3.794878	5.626601	3.537237	9.872125	71.232872	9.266577	255	1759	320
gene1318	NC_008261.1	1514990	1515643	+	protein_coding	CPF_RS06545	CPF_1340	.	WP_003460731.	cytidylate kinase	2.880565	5.543580	2.803051	5.566106	1.027654	4.852327	4.832690	6.359042	4.872044	4.832690	6.359042	4.872044	21.683511	112.803183	24.436877	300	1492	452
gene1319	NC_008261.1	1515645	1516493	+	protein_coding	CPF_RS06550	CPF_1341	.	WP_003460702.	4-hydroxy-3-m	2.834317	5.925769	3.130360	5.843878	-1.104449	5.149569	5.221731	6.724732	5.078404	5.221731	6.724732	5.078404	28.117053	143.562005	28.902592	505	2465	694
gene1320	NC_008261.1	1516603	1517397	+	protein_coding	CPF_RS06555	CPF_1342	.	WP_011590669.	N-acetyltransfe	1.379018	3.555508	1.095776	3.728318	1.258485	3.493146	3.331237	3.794878	3.662925	3.331237	3.794878	3.662925	6.897266	19.342987	10.006919	116	311	225
gene1321	NC_008261.1	1517783	1518355	+	protein_coding	CPF_RS06560	CPF_1344	.	WP_003449367.	hypothetical pro	1.732784	8.861718	3.598207	8.292211	-2.076547	7.929448	8.474040	9.267132	7.419854	8.474040	9.267132	7.419854	297.397118	705.790595	163.213496	3605	8179	2645
gene1323	NC_008261.1	1519004	1519474	-	protein_coding	CPF_RS06570	CPF_1346	.	WP_003460703.	DNA-binding pro	-2.562121	3.972027	-2.898440	4.046388	1.131266	4.796402	4.708258	3.350920	4.886197	4.708258	3.350920	4.886197	19.771127	13.647488	24.697899	197	130	329
gene1324	NC_008261.1	1519658	1520050	-	protein_coding	CPF_RS06575	CPF_1347	.	WP_003460714.	hypothetical pro	-1.375286	4.982479	-1.366083	4.977853	-1.006737	5.212800	5.217645	4.757913	5.207959	5.217645	4.757913	5.207959	28.025249	38.625677	31.669057	233	307	352
gene1325	NC_008261.1	1520410	1521390	+	protein_coding	CPF_RS06580	CPF_1348	.	WP_011590670.	linear amide C-N	-2.313904	10.754232	-1.308613	10.358283	-1.768211	10.957554	11.376410	10.166081	10.554119	11.376410	10.166081	10.554119	2236.630118	1233.879324	1542.370608	46417	24480	42793
gene1326	NC_008261.1	1521576	1522919	+	protein_coding	CPF_RS06585	CPF_1349	.	WP_003449335.	tRNA (N6-isope	2.719177	7.210929	3.527644	7.000360	-1.297320	6.334808	6.525358	7.968528	6.149823	6.525358	7.968528	6.149823	71.889875	296.270705	61.770887	2044	8053	2348
gene1327	NC_008261.1	1523375	1524988	+	protein_coding	CPF_RS06590	CPF_1350	.	WP_011590671.	phosphoenolpyr	3.353084	9.695591	3.779417	9.600668	-1.127147	8.775285	8.862047	10.607536	8.689372	8.862047	10.607536	8.689372	397.607353	1781.339073	402.189294	13576	58146	18359
gene1328	NC_008261.1	1525273	1526067	-	protein_coding	CPF_RS06595	CPF_1351	.	WP_011590672.	hypothetical pro	-1.089946	0.123708	-1.004785	0.079673	-1.084756	0.129170	0.200561	0.076304	0.083191	0.200561	0.076304	0.083191	0.118918	0.062196	0.044475	2	1	1
gene1331	NC_008261.1	1528044	1528631	+	protein_coding	CPF_RS06605	CPF_1354	.	WP_011590675.	hypothetical pro	-2.136815	8.391668	-3.093549	8.638098	1.447739	9.220294	8.957256	7.861794	9.491057	8.957256	7.861794	9.491057	420.365847	281.622988	714.371515	5229	3349	11880
gene1332	NC_008261.1	1528745	1529080	+	protein_coding	CPF_RS06610	CPF_1355	.	WP_003469972.	hypothetical pro	2.412397	2.056383	2.007780	2.228682	1.201524	1.644137	1.517029	2.787496	1.781895	1.517029	2.787496	1.781895	1.406847	8.535304	2.104630	10	58	20
gene1333	NC_008261.1	1529447	1531681	+	protein_coding	CPF_RS06615	CPF_1356	.	WP_003449375.	formate C-acet	1.506174	8.176589	2.072329	7.934351	-1.375890	7.652838	7.886481	8.477369	7.426116	7.886481	8.477369	7.426116	197.815134	424.548908	163.800660	9353	19190	10354
gene1334	NC_008261.1	1531761	1532468	+	protein_coding	CPF_RS06620	CPF_1357	.	WP_003477793.	pyruvate format	1.032401	5.553225	1.300451	5.383434	-1.259638	5.361182	5.530271	5.576274	5.197262	5.530271	5.576274	5.197262	35.519307	68.791258	31.462433	532	985	630
gene1335	NC_008261.1	1532731	1535463	+	protein_coding	CPF_RS06625	CPF_1358	.	WP_011590676.	DNA mismatch r	1.580583	3.810140	1.835107	3.690830	-1.161032	3.384779	3.494196	4.154652	3.278788	3.494196	4.154652	3.278788	7.731326	25.546143	7.645975	447	1412	591
gene1336	NC_008261.1	1535490	1537514	+	protein_coding	CPF_RS06630	CPF_1359	.	WP_011590677.	DNA mismatch r	1.611202	3.630716	1.653198	3.610255	-1.026065	3.284300	3.302914	3.991051	3.265792	3.302914	3.991051	3.265792	6.792882	22.830576	7.542994	291	935	432

gene1337	NC_008261.1	1537532	1538464	+		protein_coding	CPF_RS06635	CPF_1360	.	WP_003469969. tRNA (adenosin	1.894400	3.562456	1.938486	3.543526	-1.023271	3.114634	3.131273	4.053014	3.098084	3.131273	4.053014	3.098084	5.927756	24.060491	6.745643	117	454	178
gene1338	NC_008261.1	1538508	1538750	+		protein_coding	CPF_RS06640	CPF_1361	.	WP_003460455. RNA-binding pro	-1.070379	10.388407	1.058108	10.298638	-1.132577	10.347390	10.437584	10.339462	10.257975	10.437584	10.339462	10.257975	1190.505090	1442.477286	1292.814376	6120	7089	8885
gene1339	NC_008261.1	1538855	1540135	+		protein_coding	CPF_RS06645	CPF_1362	.	WP_003469951. hypothetical pro	2.108700	5.504500	2.545233	5.352770	-1.207015	4.854951	4.992569	6.068923	4.721125	4.992569	6.068923	4.721125	24.096289	96.382834	21.915752	653	2497	794
gene1340	NC_008261.1	1540394	1541299	+		protein_coding	CPF_RS06650	CPF_1363	.	WP_003469966. hypothetical pro	-1.180594	4.462045	-1.550141	4.649355	1.313018	4.775813	4.583408	4.343895	4.976295	4.583408	4.343895	4.976295	18.156710	29.252762	26.811033	348	536	687
gene1341	NC_008261.1	1541479	1542090	-		protein_coding	CPF_RS06655	CPF_1364	.	WP_003452542. XRE family trans	-1.108990	7.351114	1.087425	7.216159	-1.205943	7.289784	7.426116	7.276870	7.155954	7.426116	7.276870	7.155954	138.566142	202.308059	127.796428	1794	2504	2212
gene1342	NC_008261.1	1542298	1542690	+		protein_coding	CPF_RS06660	CPF_1365	.	WP_003477798. hypothetical pro	3.685423	5.917017	4.768996	5.695023	-1.294016	4.860964	5.050446	6.932277	4.678591	5.050446	6.932277	4.678591	25.379088	160.541902	21.232663	211	1276	236
gene1343	NC_008261.1	1542781	1544133	-		protein_coding	CPF_RS06665	CPF_1366	.	WP_011590678. tyrosine recomb	-1.641150	2.367035	-1.606558	2.353776	-1.021532	2.735801	2.751211	2.036504	2.720478	2.751211	2.036504	2.720478	4.297278	4.458536	5.122044	123	122	196
gene1344	NC_008261.1	1544649	1544867	-		protein_coding	CPF_RS06670	CPF_1367	.	WP_011590679. DUF3892 domai	-2.187546	1.269196	-1.410066	1.043317	-1.551378	1.606075	1.953792	0.824478	1.320241	1.953792	0.824478	1.320241	2.158450	1.128902	1.291608	10	5	8
gene1345	NC_008261.1	1544995	1546194	+		protein_coding	CPF_RS06675	CPF_1368	.	WP_003460673. threonine ammo	-22.212253	9.178483	-19.361780	9.100322	-1.147222	11.584216	11.683711	7.210428	11.485567	11.683711	7.210428	11.485567	2963.044618	193.415876	2766.039289	75220	4694	93876
gene1346	NC_008261.1	1546235	1547704	-		protein_coding	CPF_RS06680	CPF_1369	.	WP_003460660. amino acid perm	1.262588	5.032349	1.205462	5.066765	1.047390	4.900252	4.866966	5.203351	4.933765	4.866966	5.203351	4.933765	22.252298	52.540473	25.712564	692	1562	1069
gene1347	NC_008261.1	1548207	1549427	+		protein_coding	CPF_RS06685	CPF_1370	.	WP_011590681. dicarboxylate/ar	5.319830	7.103554	6.797428	6.891033	-1.277753	5.819969	5.999458	8.410839	5.645850	5.999458	8.410839	5.645850	49.167052	392.610970	42.394589	1270	9695	1464
gene1348	NC_008261.1	1549462	1549794	-		protein_coding	CPF_RS06690	CPF_1371	.	WP_003456465. hypothetical pro	2.850039	5.035908	2.374778	5.186466	1.200129	4.466428	4.336771	5.847753	4.599960	4.336771	5.847753	4.599960	14.621068	84.043180	19.961751	103	566	188
gene1349	NC_008261.1	1550070	1550714	+		protein_coding	CPF_RS06695	CPF_1372	.	WP_003456593. membrane prote	10.431712	6.510175	8.216548	6.729132	1.269598	5.204207	5.034868	8.417773	5.379240	5.034868	8.417773	5.379240	25.064122	393.804008	35.467419	342	5137	647
gene1350	NC_008261.1	1550747	1550965	-		protein_coding	CPF_RS06700	CPF_1373	.	WP_003453540. hypothetical pro	-1.376490	5.895849	-1.397307	5.906252	1.015123	6.141668	6.130850	5.669855	6.152504	6.130850	5.669855	6.152504	54.177095	73.604398	61.835756	251	326	383
gene1351	NC_008261.1	1551028	1551411	-		protein_coding	CPF_RS06705	CPF_1374	.	WP_003456607. DNA mismatch r	-1.102753	6.397640	1.029530	6.306446	-1.135317	6.376379	6.468584	6.327474	6.285488	6.468584	6.327474	6.285488	69.550992	111.382039	68.045315	565	865	739
gene1352	NC_008261.1	1551763	1552497	-		protein_coding	CPF_RS06710	CPF_1375	.	WP_003453545. HAD-IB family h	1.005279	6.056814	1.396022	5.814974	-1.388691	5.811329	6.053017	6.060613	5.579291	6.053017	6.060613	5.579291	50.935896	95.931772	40.072153	792	1426	833
gene1353	NC_008261.1	1552668	1553204	+		protein_coding	CPF_RS06715	CPF_1376	.	WP_003477808. ECF transporter	5.705367	5.863391	2.943607	6.426792	1.938223	5.195764	4.740280	7.252599	5.695014	4.740280	7.252599	5.695014	20.246020	197.599441	44.180767	230	2146	671
gene1354	NC_008261.1	1553235	1553987	-		protein_coding	CPF_RS06720	CPF_1377	.	WP_011590682. exodeoxyribonu	-1.694676	7.100518	-1.699326	7.102391	1.002744	7.493187	7.491211	6.730201	7.495164	7.491211	6.730201	7.495164	144.886171	144.266455	173.642866	2308	2197	3698
gene1355	NC_008261.1	1554219	1554446	+		protein_coding	CPF_RS06725	.	.	WP_011590683. N-acetyltransfe	-1.225504	3.602176	1.042214	3.428519	-1.277238	3.570976	3.751849	3.458474	3.398823	3.751849	3.458474	3.398823	9.536941	15.180759	8.374212	46	70	54
gene1356	NC_008261.1	1554567	1554989	-		protein_coding	CPF_RS06730	CPF_1379	.	WP_011590684. N-acetyltransfe	1.143512	5.183566	1.495788	4.982293	-1.308065	4.890180	5.087733	5.281205	4.700299	5.087733	5.281205	4.700299	25.814144	55.290568	21.649328	231	473	259
gene1357	NC_008261.1	1555312	1555890	-		protein_coding	CPF_RS06735	CPF_1381	.	WP_003456665. orotate phospho	267.404649	8.019419	212.804074	8.282326	1.256577	5.106374	4.944282	13.007163	5.273781	4.944282	13.007163	5.273781	23.267644	9256.118643	33.342572	285	108387	546
gene1358	NC_008261.1	1556039	1556938	-		protein_coding	CPF_RS06740	CPF_1382	.	WP_011590685. dihydroorotate c	220.662056	6.788913	128.570433	7.431114	1.716274	4.305024	3.932981	11.718676	4.712261	3.932981	11.718676	4.712261	10.819590	3876.064051	21.843252	206	70551	556
gene1359	NC_008261.1	1556958	1557698	-		protein_coding	CPF_RS06745	CPF_1383	.	WP_011590686. dihydroorotate c	195.695657	7.435128	123.189094	7.962413	1.588569	4.868953	4.546523	12.158991	5.214250	4.546523	12.158991	5.214250	17.734245	5365.046902	31.683627	278	80401	664
gene1360	NC_008261.1	1557723	1558586	-		protein_coding	CPF_RS06750	CPF_1384	.	WP_011590687. orotidine 5'-pho	180.534547	6.831931	120.417294	7.308739	1.499241	4.326715	4.044449	11.540580	4.628681	4.044449	11.540580	4.628681	11.872224	3568.174050	20.420756	217	62349	499
gene1361	NC_008261.1	1558596	1559795	-		protein_coding	CPF_RS06755	CPF_1385	.	WP_011590688. dihydroorotase	176.374932	6.900039	139.760582	7.176042	1.261925	4.277554	4.113032	11.575533	4.448658	4.113032	11.575533	4.448658	12.447781	3623.065866	17.885145	316	87928	607
gene1362	NC_008261.1	1559817	1559975	-		protein_coding	CPF_RS06760	.	.	WP_041702664. aspartate carbar	163.315540	2.646879	146.606650	2.878131	1.113971	0.928427	0.853830	8.205348	1.009541	0.853830	8.205348	1.009541	0.594592	346.121296	0.889504	2	1113	4
gene1363	NC_008261.1	1559980	1560903	-		protein_coding	CPF_RS06765	CPF_1387	.	WP_003453538. aspartate carbar	179.310582	6.670474	260.438313	6.193609	-1.452443	3.626572	3.905793	11.392110	3.367312	3.905793	11.392110	3.367312	10.640878	3269.529826	8.150658	208	61098	213
gene1364	NC_008261.1	1561294	1562073	-		protein_coding	CPF_RS06770	CPF_1388	.	WP_003456515. serine hydrolase	-15.948549	10.051241	-15.020184	10.015669	-1.061808	12.202175	12.245513	8.250160	12.158991	12.245513	8.250160	12.158991	4377.994932	353.728357	4214.466297	72241	5580	92972
gene1365	NC_008261.1	1562304	1563404	-		protein_coding	CPF_RS06775	CPF_1389	.	WP_025647569. ATP-dependent	-17.258863	8.061470	-11.710353	7.841042	-1.473812	10.090161	10.373816	6.264550	9.814263	10.373816	6.264550	9.814263	1136.198671	107.155237	931.794774	26464	2386	29015
gene1366	NC_008261.1	1563614	1564528	-		protein_coding	CPF_RS06780	CPF_1390	.	WP_011590690. undecaprenyl-d	2.616549	6.073403	2.448753	6.126750	1.068523	5.466674	5.419074	6.806739	5.514692	5.419074	6.806739	5.514692	32.753240	149.580600	38.719670	634	2768	1002
gene1367	NC_008261.1	1564788	1565765	-		protein_coding	CPF_RS06785	CPF_1391	.	WP_003462511. mannose-6-pho	2.241933	7.226047	2.318491	7.199747	-1.034148	6.642840	6.667105	7.831848	6.618662	6.667105	7.831848	6.618662	80.426760	275.794868	87.129095	1664	5455	2410
gene1368	NC_008261.1	1566013	1567371	+		protein_coding	CPF_RS06790	CPF_1392	.	WP_041702665. DUF1727 domai	3.253142	5.752373	3.445403	5.704174	-1.059100	4.922457	4.964051	6.665885	4.881212	4.964051	6.665885	4.881212	23.617636	139.387226	24.612555	679	3831	946
gene1369	NC_008261.1	1567389	1568120	+		protein_coding	CPF_RS06795	CPF_1393	.	WP_003453555. glutamine amido	3.095715	6.827260	3.476674	6.732292	-1.123060	5.976309	6.060613	7.690886	5.893178	6.060613	7.690886	5.893178	51.402956	257.496951	51.249463	796	3812	1061
gene1373	NC_008261.1	1569982	1571244	+		protein_coding	CPF_RS06815	CPF_1397	.	WP_003467177. VanW family prot	-2.376537	8.073323	-2.282929	8.046447	-1.041003	8.692830	8.721866	7.473005	8.663891	8.721866	7.473005	8.663891	360.457562	225.814685	399.097815	9631	5768	14256
gene1374	NC_008261.1	1571291	1572067	-		protein_coding	CPF_RS06820	CPF_1398	.	WP_011590693. membrane prote	1.053296	4.863044	-1.083082	4.957881	1.140807	4.919842	4.825732	4.900644	5.015787	4.825732	4.900644	5.015787	21.657837	42.382199	27.667351	356	666	608
gene1375	NC_008261.1	1572117	1572527	-		protein_coding	CPF_RS06825	CPF_1399	.	WP_003453591. hypothetical pro	-1.380193	5.719629	-1.400079	5.729529	1.014408	5.967095	5.956785	5.491915	5.977424	5.956785	5.491915	5.977424	47.155043	64.363884	54.198060	410	535	630
gene1376	NC_008261.1	1572642	1574138	-		protein_coding	CPF_RS06830	CPF_1400	.	WP_003456419. glutamate/gamm	2.061391	8.542716	1.763964	8.661366	1.168613	8.148452	8.036828	9.080447	8.261626	8.036828	9.080447	8.261626	229.845512	632.326184	303.576201	7279	19144	12853
gene1377	NC_008261.1	1574452	1575030	+		protein_coding	CPF_RS06835	CPF_1401	.	WP_003456584. hypothetical pro	-1.296036	5.906289	-1.456346	5.987236	1.123692	6.179854	6.096304	5.722197	6.264550	6.096304	5.722197	6.264550	52.658351	76.517316	66.379808	645	896	1087
gene1378	NC_008261.1	1575064	1576500	-		protein_coding	CPF_RS06840	CPF_1402	.	WP_011590694. sensor histidine	1.833402	4.132854	2.013022	4.057233	-1.097971	3.650617	3.718660	4.593183	3.583820	3.718660	4.593183	3.583820	9.276378	33.996206	9.497637	282	988	386
gene1379	NC_008261.1	1576487	1577164	-		protein_coding	CPF_RS06845	CPF_1403	.	WP_003453585. DNA-binding res	1.159008	4.644643	1.513255	4.443443	-1.305647	4.342775	4.539417	4.752308	4.154652	4.539417	4.752308	4.154652	17.639121	38.287754	14.497734	253	525	278
gene1380	NC_008261.1	1577411	1578673	-		protein_coding	CPF_RS06850	CPF_1405	.	WP_003453604. 2-hydroxyglutar	1.933027	5.282443	2.638548	5.030906	-1.364983	4.598449	4.828364	5.779226	4.379482	4.828364	5.779226	4.379482	21.670120	80.100008	17.076997	579	2046	610
gene1381	NC_008261.1	1578682	1581609	-		protein_coding	CPF_RS06855	CPF_1406	.	WP_011590695. 2-hydroxyglutar	1.408037	5.439473	1.871364	5.220333	-1.329059	4.988807	5.198228	5.691914	4.787824	5.198228	5.691914	4.787824	27.719496	74.793678	22.871461	1717	4429	1894
gene1382	NC_008261.1	1581901	1583889	-		protein_coding	CPF_RS06860	CPF_1407	.	WP_011590696. YgiQ family radic	1.586623	7.553949	2.065879	7.352281	-1.302060	7.035331	7.228305	7.894264	6.847508	7.228305	7.894264	6.847508	123.819501	287.502175	104.740096	5210	11565	5892
gene1383	NC_008261.1	1584089	1586434	-		protein_coding	CPF_RS06865	CPF_1408	.	WP_011590697. ATP-dependent	-1.209754	4.245049	-1.147480	4.207979	-1.054270	4.346338	4.384628	4.109914	4.308383	4.384628	4.109914	4.308383	15.252955	24.786180	16.216954	757	1176	1076
gene1384	NC_008261.1	1586536	1587930	-		protein_coding	CPF_RS06870	CPF_1409	.	WP_041702666. MATE family effl	-2.043956	4.866160	-1.696968	4.743892	-1.204475	5.273180	5.409090	4.377726	5.140686	5.409090	4.377726	5.140686	32.563816	29.773875	30.237874	961	840	1193
gene1385	NC_008261.1	1588092	1589198	-		protein_coding	CPF_RS06875	CPF_1410	.	WP_011590699. aminoglycoside	-2.235173	4.335912	-2.027902	4.274039	-1.102209	4.884048	4.954751	3.794365	4.814353	4.954751	3.794365	4.814353	23.485574	19.340627	23.444094	550	433	734
gene1386	NC_008261.1	1589200	1590111	-		protein_coding	CPF_RS06880	CPF_1411	.	WP_011590700. nucleotidyltrans	-1.707404	2.140934	-1.817029	2.178132	1.064205	2.605840	2.561338	1.789533	2.651114	2.561338	1.789533	2.651114	3.783678	3.578322	4.807418	73	66	124
gene1387	NC_008261.1	1590375	1592981	+		protein_coding	CPF_RS06885	CPF_1412	.	WP_011590701. calcium-transpo	-1.184944	1.017186	1.053846	0.863456	-1.248748	0.973595	1.146935	0.902116	0.826452	1.146935	0.902116	0.826452	0.906599	1.251795	0.664569	50	66	49
gene1388	NC_008261.1	1593026	1594642	-		protein_coding	CPF_RS06890	CPF_1413	.	WP_003456652. polysaccharide b	1.935661	5.849787	1.933300	5.850742	1.001221	5.393622	5.392742	6.345568	5.394502	5.392742	6.345568	5.394502	32.156500	112.101835	35.751375	1100	3666	1635
gene1389	NC_008261.1	1594664	1595638	-		protein_coding	CPF_RS06895	CPF_1414	.	WP_003452833. RluA family pseu	1.322139	6.622608	1.388750	6.585955	-1.050381	6.388679	6.424234	6.827107	6.353321	6.424234	6.827107	6.353321	68.505218	152.191943	71.585909	1413	3001	1974
gene1390	NC_008261.1	1595698	1596894	-		protein_coding	CPF_RS06900	CPF_1415	.	WP_011590702. amidohydrolase	1.000000	7.160860	1.094639	7.095333	-1.094639	7.095333	7.160860	7.160860	7.030405	7.160860	7.160860	7.030405	119.221634	187.539167	120.104212	3019	4540	4066
gene1391	NC_008261.1	1597100	1599094	-		protein_coding	CPF_RS06905	CPF_1416	.	WP_011590703. ATP-dependent	1.598961	2.152102	1.603481	2.149718	-1.002827	1.837966	1.840003	2.517138	1.835930	1.840003	2.517138	1.835930	1.942930	6.766281	2.286293	82	273	129
gene1392	NC_008261.1	1599247	1600719	+		protein_coding	CPF_RS06910	CPF_1417	.	WP_003452832. nicotinate phos	2.872412	5.947408	3.579426	5.764246	-1.246139	5.073567	5.234782	6.757045	4.917317	5.234782	6.757045	4.917317	28.432633	146.122200	25.348145	886	4353	1056
gene1393	NC_008261.1	1600979	1601314	-		protein_coding	CPF_RS06915	CPF_1419	.	WP_003452838. hypothetical pro	1.241655	10.630166	1.194833	10.658266	1.039187	10.502871	10.475180	10.787444	10.530635	10.475180	10.787444	10.530635	1221.565143	2133.384533	1522.910183	8683	14497	14472
gene1394	NC_008261.1	1601933	1602352	+		protein_coding	CPF_RS06920	CPF_1420	.	WP_003456471. transcriptional r	-1.731177	11.172646	-1.048669	10.818006	-1.650832	11.208105	11.575533	10.783781	10.852340	11.575533	10.783781	10.852340	2684.714032	2125.114118	1819.578812	23854	18051	21614
gene1395	NC_008261.1	1602430	1603827	-		protein_coding	CPF_RS06925	CPF_1421	.	WP_011590704. dipeptidase PepV	-1.917796	10.727775	-1.579889	10.593105	-1.213880	11.067082	11.207779	10.268329	10.928152	11.207779	10.268329	10.928152	1920.996880	1364.855372	1906.993395	56813	38589	75400
gene1396	NC_008261.1	1604016	1605326	-		protein_coding	CPF_RS06930	CPF_1422	.	WP_003477835. ABC transporter	17.545554	5.783459	18.801238	5.712256	-1.071567	4.024884	4.075054	8.208087	3.975332	4.075054	8.208087	3.975332	12.042867	346.573890	12.460179	334	9189	462
gene1397	NC_008261.1	1605611	1606021	-		protein_coding	CPF_RS06935	CPF_1423	.	WP_011590705. acyl-CoA thioes	1.821706	5.741459	1.450307	5.916128	1.256083	5.487094	5.325092	6.190382	5.654024	5.325092	6.190382	5.654024	30.363247	102.741601	42.756247	264	854	497
gene1398	NC_008261.1	1606212	1606967	-		protein_coding	CPF_RS06940	CPF_1424	.	WP_003456463. ABC transporter	1.572466	4.393006	1.714468	4.325311	-1.090305	4.015758	4.078609	4.731637	3.953877	4.078609	4.731637	3.953877	12.067620	37.542257	12.347162	193	574	264
gene1399	NC_008261.1	1606973	1607980	-		protein_coding	CPF_RS06945	CPF_1425	.	WP_011590706. iron ABC transp	1.270362	4.915262	1.592737	4.743290	-1.253766	4.579634	4.745673	5.090912	4.419404	4.745673	5.090912	4.419404	20.205490	47.778081	17.573660	433	974	501
gene1400	NC_008261.1	1608068	1609066	-		protein_coding	CPF_RS06950	CPF_1426	.	WP_003456650. iron ABC transp	-1.094723	6.490070	1.059704	6.383148	-1.160083	6.447678	6.555682	6.425115	6.341454	6.555682	6.425115	6.341454	73.436562	118.689957	70.857137	1552	2398	2002
gene1401	NC_008261.1	1609079	1609843	-		protein_coding	CPF_RS06955	CPF_1427	.	WP_003456657. precorrin-6A re	-1.017467	6.468006	1.142209	6.358890	-1.162160	6.371183	6.480509	6.455527	6.263700	6.480509	6.455527	6.263700	69.885532	120.738484	66.370948	1131	1868	1436
gene1402	NC_008261.1	1609862	1610584	-		protein_coding	CPF_RS06960	CPF_1428	.	WP_003477836. precorrin-3B C(	-1.253078	7.021066	-1.048519	6.894305	-1.195094	7.055957	7.185690	6.860213	6.928566	7.185690	6.860213	6.928566	120.757665	156.612876	111.452815	1847	2290	2279
gene1403	NC_008261.1	1610739	1611737	-		protein_coding	CPF_RS06965	CPF_1429	.	WP_003456627. cobalt-precorrir	-1.177719	6.344633	1.017944	6.214891	-1.198851	6.331550	6.463728	6.227733	6.202075	6.463728	6.227733	6.202075	69.177998	104.880741	63.849288	1462	2119	1804
gene1404	NC_008261.1	1611740	1612495	-		protein_coding	CPF_RS06970	CPF_1430	.	WP_003452074. precorrin-4 C(1	-1.454975	6.965365	-1.131623	6.788729	-1.285742	7.057484	7.241112	6.700118	6.878511	7.241112	6.700118	6.878511	125.240635	142.058854	106.587811	2003	2172	2279
gene1405	NC_008261.1	1612546	1613208	-		protein_coding	CPF_RS06975	CPF_1431	.	WP_003456602. cobalt-factor II	-1.292755	6.295028	-1.095527	6.177990	-1.180031	6.362445	6.482978	6.112528	6.244153	6.482978	6.112528	6.244153	70.227759	98.817220	65.595885	985	1325	1230
gene1406	NC_008261.1	1613208	1613801	-		protein_coding	CPF_RS06980	CPF_1432	.	WP_003452120. precorrin-6Y C5	-1.058701	4.113299	-1.081148	4.128255	1.021202	4.169759	4.154652	4.072357	4.184921	4.154652	4.072357	4.184921	12.732675	24.306738	14.762172	160	292	248
gene1407	NC_008261.1	1613821	1614432	-		protein_coding	CPF_RS06985	CPF_1433	.	WP_003456587. precorrin-6y C5	-1.108732	5.562924	1.020234	5.474498	-1.131166	5.548260	5.637877	5.488967	5.460067	5.637877	5.488967	5.460067	38.155894	64.231193	37.437652	494	795	648
gene1408	NC_008261.1	1614460	1615557	-		protein_coding	CPF_RS06990	CPF_1434	.	WP_003456440. cobalt-precorrir	-1.152823	7.086722	-1.062782	7.028664	-1.084722	7.131145	7.190050	6.984879	7.072724	7.190050	6.984879	7.072724	121.360915	164.819665	123.623429	2819	3660	3839
gene1409	NC_008261.1	1615575	1616204	-	cbiC	protein_coding	CPF_RS06995	CPF_1435	.	WP_003452122. precorrin-8X m	-1.212572	7.216195	1.061415	7.035373	-1.287042	7.172230	7.356570	7.078499	6.992510	7.356570	7.078499	6.992510	133.181504	175.179758	117.129668	1775	2232	2087
gene1410	NC_008261.1	1616204	1617046	-		protein_coding	CPF_RS07000	CPF_1436	.	WP_003452057. sirohydrochlorin	1.132247	6.771822	1.310892	6.663874	-1.157779	6.576291	6.682820	6.862009	6.471461	6.682820	6.862009	6.471461	81.250664	156.725318	78.978296	1449	2672	1883
gene1411	NC_008261.1	1617566	1618798	+		protein_coding	CPF_RS07005	CPF_1437	.	WP_003456592. hypothetical pro	1.336960	7.542087	1.830625	7.305296	-1.369245	7.105212	7.335518	7.754474	6.882137	7.335518	7.754474	6.882137	131.535733	265.195241	106.761575	3431	6613	3723
gene1412	NC_008261.1	1618818	1619126	-		protein_coding	CPF_RS07010	CPF_1438	.	WP_003456488. hypothetical pro	1.095110	0.750663	1.544349	0.396619	-1.410222	0.363500	0.687981	0.819056	0.192058	0.687981	0.819056	0.192058	0.458933	1.120134	0.114426	3	7	1
gene1413	NC_008261.1	1619394	1622543	-		protein_coding	CPF_RS07015	CPF_1439	.	WP_011590707. mannosyl-glyco	-1.431509	7.293915	1.055709	7.000520	-1.511257	7.253284	7.557273	7.039736	6.961523	7.557273	7.039736	6.961523	152.419666	170.815961	114.188799	10157	10882	10173
gene1414	NC_008261.1	1622926	1623927	-		protein_coding	CPF_RS07020	CPF_1440	.	WP_003452142. hypothetical pro	2.439660	11.232468	2.592066	11.186086	-1.062470	10.563735	10.607536	11.894216	10.520114	10.607536	11.894216	10.520114	1327.948882	4499.823578	1508.528126	28149	91187	42750
gene1415	NC_008261.1	1623961	1624653	-		protein_coding	CPF_RS07025	CPF_1441	.	WP_003477845. nitrogen-fixing	2.982432	11.028440	2.974294	11.030557	1.002736	10.270300	10.268329	11.844819	10.272271	10.268329	11.844819	10.272271	1049.081453	4316.491448	1314.513559	15380	60497	25764
gene1416	NC_008261.1	1625168	1628173	-		protein_coding	CPF_RS07030	CPF_1442	.	WP_003456570. O-GlcNAcase N	-1.915128	2.004679	-2.474550	2.146301	1.292107	2.706022	2.527466	1.590025	2.897192	2.527466	1.590025	2.897192	3.679705	2.960832	5.681240	234	180	483
gene1418	NC_008261.1	1628620	1629597	-		protein_coding	CPF_RS07035	.	.	WP_041702669. hypothetical pro	-1.131048	1.844260	-1.315905	1.945543	1.163438	2.041507	1.935229	1.757568	2.153623	1.935229	1.757568	2.153623	2.126669	3.488514	3.145324	44	69	87
gene1419	NC_008261.1	1629829	1630512	-		protein_coding	CPF_RS07040	CPF_1446	.	WP_003467108. superoxide dism	-1.533439	10.039047	-1.429227	9.989693	-1.072915	10.301275	10.352168	9.735397	10.250632	10.352168	9.735397	10.250632	1107.943747	948.869700	1289.938732	16032	13126	24954
gene1420	NC_008261.1	1630574	1631185	-		protein_coding	CPF_RS07045	CPF_1447	.	WP_011010339. hypothetical pro	-1.020278	0.247188	1.022832	0.216234	-1.043573	0.229272	0.262092	0.233131	0.200561	0.262092	0.233131	0.200561	0.154477	0.242382	0.115548	2	3	2
gene1421	NC_008261.1	1631495	1632943	+		protein_coding	CPF_RS07050	CPF_1448	.	WP_003467196. MFS transporter	5.056308	2.523827	4.885036	2.562441	1.035061	1.637059	1.612390	3.950474	1.662105	1.612390	3.950474	1.662105	1.533259	22.010079	1.878916	47	645	77
gene1422	NC_008261.1	1633198	1634535	-		protein_coding	CPF_RS07055	CPF_1450	.	WP_003452139. magnesium tran	-2.911125	8.165188	-3.667265	8.315388	1.259741	9.137323	8.972277	7.430700	9.305405	8.972277	7.430700	9.305405	424.794575	220.843568	628.300916	12024	5976	23776
gene1423	NC_008261.1	1635096	1635869	-		protein_coding	CPF_RS07060	CPF_1451	.	WP_011590709. YdcF family prot	-1.442650	5.201930	-2.029276	5.430825	1.406631	5.713827	5.473003	4.944282	5.965247	5.473003	4.944282	5.965247	33.956267	43.632493	53.676220	556	683	1175
gene1424	NC_008261.1	1636132	1636341	+		protein_coding	CPF_RS07065	CPF_1452	.	WP_003452066. cold-shock prot	1.455243	3.666338	1.505483	3.639885	-1.034524	3.381110	3.405683	3.946942	3.356715	3.405683	3.946942	3.356715	7.203056	21.897470	8.081779	32	93	48
gene1425	NC_008261.1	1636494	1636700	-		protein_coding	CPF_RS07070	CPF_1453	.	WP_003467361. heavy metal tran	-1.655250	2.703319	-1.124003	2.446997	-1.472638	2.798079	3.091176	2.364127	2.532773	3.091176	2.364127	2.532773	5.708944	5.971727	4.270264	25	25	25
gene1426	NC_008261.1	1636747	1637217	-		protein_coding	CPF_RS07075	CPF_1454	.	WP_003467334. murein L,D-tran	-1.060042	2.326689	-1.397258	2.514757	1.318116	2.560628	2.369129	2.285008	2.767607	2.369129	2.285008	2.767607	3.111193	5.563976	5.254872	31	53	70
gene1427	NC_008261.1	1637336	1638307	-		protein_coding	CPF_RS07080	CPF_1455	.	WP_003467193. gfo/Idh/MocA f	-2.842263	7.172132	-2.695651	7.115243	-1.091485	7.929321	7.992718	6.435792	7.866426	7.992718	6.435792	7.866426	220.836749	119.595997	228.625165	4541	2351	6285
gene1428	NC_008261.1	1638497	1641052	-		protein_coding	CPF_RS07085	CPF_1456	.	WP_011590710. bifunctional lysy	3.402947	3.898997	1.682441	4.490279	2.022825	3.586729	3.114427	4.881212	4.130656	3.114427	4.881212	4.130656	5.825535	41.823957	14.206746	315	2162	1027
gene1429	NC_008261.1	1641627	1642565	-		protein_coding	CPF_RS07090	CPF_1457	.	WP_003467356. lysylphosphatidy	1.218666	4.769200	1.107284	4.839913	1.100590	4.697311	4.628681	4.913984	4.766958	4.628681	4.913984	4.766958	18.726795	42.653012	22.557523	372	810	598
gene1430	NC_008261.1	1642704	1644195	-		protein_coding	CPF_RS07095	CPF_1458	.	WP_003462589. hypothetical pro	-2.259285	4.526096	-2.811666	4.662624	1.244450	5.307465	5.152055	3.976189	5.467563	5.152055	3.976189	5.467563	26.805580	22.612454	37.657475	279	225	524
gene1431	NC_008261.1	1643416	1644609	-		protein_coding	CPF_RS07100	CPF_1459	.	WP_011590712. hypothetical pro	-2.041800	0.476069	-1.764764	0.432943	-1.156982	1.106022	1.216195	0.186353	1.005829	1.216195	0.186353	1.005829	0.989742	0.207060	0.888386	25	5	30
gene1432	NC_008261.1	1644619	1645602	-		protein_coding	CPF_RS07105	CPF_1460	.	WP_011590713. hypothetical pro	-4.366438	2.629548	-3.757205	2.555408	-1.162151	3.789647	3.899595	1.773138	3.682798	3.899595	1.773138	3.682798	10.616547	3.517493	10.204888	221	70	284
gene1433	NC_008261.1	1645992	1646714	-		protein_coding	CPF_RS07110	CPF_1461	.	WP_003456586. membrane prote	1.998207	9.220869	1.596745	9.390100	1.251425	8.895342	8.735027	9.733733	9.058599	8.735027	9.733733	9.058599	364.103647	947.952436	527.236858	5569	13861	10781
gene1434	NC_008261.1	1646894	1647232	-		protein_coding	CPF_RS07115	CPF_1462	.	WP_011590714. hypothetical pro	-1.209221	1.058069	-1.400140	1.147244	1.157886	1.305415	1.203945	0.929867	1.415438	1.203945	0.929867	1.415438	0.976078	1.312723	1.460203	7	9	14
gene1435	NC_008261.1	1647256	1648662	-		protein_coding	CPF_RS07120	CPF_1463	.	WP_003456576. glutamate synth	-1.481319	2.485911	-1.523227	2.503807	1.028291	2.805511	2.785459	2.218577	2.825707	2.785459	2.218577	2.825707	4.468314	5.271418	5.428060	133	150	216
gene1436	NC_008261.1	1648655	1649554	-		protein_coding	CPF_RS07125	CPF_1464	.	WP_011590715. ferredoxin-NAD	-1.771666	2.612067	-1.559878	2.532382	-1.135772	2.963740	3.057000	2.231893	2.873326	3.057000	2.231893	2.873326	5.514840	5.329169	5.539386	105	97	141
gene1437	NC_008261.1	1649726	1650892	-		protein_coding	CPF_RS07130	CPF_1465	.	WP_011590716. NADPH-depend	-1.472346	3.888622	-1.676688	3.974926	1.138787	4.270400	4.177680	3.619563	4.365177	4.177680	3.619563	4.365177	12.961797	17.286998	16.845696	320	408	556
gene1438	NC_008261.1	1651004	1652455	-		protein_coding	CPF_RS07135	CPF_1466	.	WP_011590717. DUF3502 domai	-16.230097	5.919271	-13.246156	5.813324	-1.225268	8.113754	8.261626	4.241026	7.968528	8.261626	4.241026	7.968528	265.259235	27.379134	242.415097	8148	804	9955
gene1439	NC_008261.1	1652905	1655748	+		protein_coding	CPF_RS07140	CPF_1467	.	WP_011590718. enterotoxin	-1.098096	7.262644	-1.047980	7.229182	-1.047821	7.296685	7.330460	7.195456	7.263067	7.330460	7.195456	7.263067	131.156120	190.272828	139.877077	7891	10944	11251
gene1440	NC_008261.1	1655620	1656944	-		protein_coding	CPF_RS07145	CPF_1468	.	WP_011590719. HNH endonucle	1.010001	3.204932	-1.139795	3.305159	1.151195	3.297764	3.197762	3.212119	3.400894	3.197762	3.212119	3.400894	6.199351	12.266906	8.385397	120	227	217
gene1441	NC_008261.1	1657242	1657862	-		protein_coding	CPF_RS07150	CPF_1469	.	WP_011590720. hypothetical pro	-2.836990	5.224173	-4.026490	5.438591	1.419283	6.277727	6.030225	4.525864	6.535387	6.030225	4.525864	6.535387	50.238706	32.247326	82.558428	660	405	1450
gene1442	NC_008261.1	1658115	1658749	-		protein_coding	CPF_RS07155	CPF_1470	.	WP_003473455. hypothetical pro	-2.085244	3.824177	-2.989461	4.044151	1.433626	4.643422	4.390852	3.330636	4.910521	4.390852	3.330636	4.910521	15.398579	13.410327	25.179027	215	179	470
gene1443	NC_008261.1	1659824	1660288	+		protein_coding	CPF_RS07160	CPF_1471	.	WP_003452135. transcription elo	-1.526344	7.021541	-1.259854	6.887737	-1.211524	7.193461	7.333204	6.723124	7.056380	7.333204	6.723124	7.056380	131.441250	143.446276	121.509109	1293	1349	1598
gene1444	NC_008261.1	1660597	1665363	-		protein_coding	CPF_RS07165	CPF_1472	.	WP_011590722. sialidase	-3.494996	1.936910	-3.221706	1.899114	-1.084828	2.980245	3.039556	1.234265	2.922090	3.039556	1.234265	2.922090	5.473688	1.970783	5.792832	552	190	781
gene1445	NC_008261.1	1665622	1667385	+		protein_coding	CPF_RS07170	CPF_1473	.	WP_011590723. DUF4091 domai	1.091535	5.455555	-1.304595	5.707679	1.424011	5.641963	5.392742	5.519100	5.902702	5.392742	5.519100	5.902702	32.156500	65.955896	52.094601	1200	2353	2599
gene1446	NC_008261.1	1667508	1671575	-		protein_coding	CPF_RS07175	CPF_1474	.	WP_011590724. DUF4982 domai	-1.886084	2.000963	-2.261735	2.102804	1.199170	2.638105	2.510339	1.594945	2.772375	2.510339	1.594945	2.772375	3.602192	2.977936	5.275854	310	245	607
gene1447	NC_008261.1	1671824	1673158	-		protein_coding	CPF_RS07180	CPF_1475	.	WP_003462412. amidohydrolase	5.363039	2.216450	4.943976	2.313299	1.084762	1.371863	1.314428	3.737479	1.431807	1.314428	3.737479	1.431807	1.133065	18.593140	1.483173	32	502	56
gene1448	NC_008261.1	1673307	1675013	-		protein_coding	CPF_RS07185	CPF_1476	.	WP_011590725. adenine deamina	23.335462	3.363246	20.434355	3.538927	1.141972	1.879971	1.786645	6.331097	1.978173	1.786645	6.331097	1.978173	1.883048	111.608114	2.692743	68	3853	130
gene1449	NC_008261.1	1675029	1676075	-		protein_coding	CPF_RS07190	CPF_1477	.	WP_003462470. ABC transporter	35.478420	3.085095	51.314527	2.451071	-1.446359	1.147014	1.443715	6.592585	0.911289	1.443715	6.592585	0.911289	1.309295	134.830986	0.776723	29	2855	23
gene1450	NC_008261.1	1676115	1677146	-		protein_coding	CPF_RS07195	CPF_1478	.	WP_011590726. ABC transporter	26.905296	1.862788	27.954030	1.781139	-1.038979	0.615196	0.643397	5.393215	0.588231	0.643397	5.393215	0.588231	0.412239	59.986692	0.411137	9	1252	12
gene1451	NC_008261.1	1677182	1677964	-		protein_coding	CPF_RS07200	CPF_1479	.	WP_003467290. ABC transporter	24.244531	0.986383	19.744173	1.547745	1.227933	0.317911	0.202606	4.802193	0.498838	0.202606	4.802193	0.498838	0.120741	39.342012	0.361254	2	623	8
gene1452	NC_008261.1	1677966	1678796	-		protein_coding	CPF_RS07205	CPF_1480	.	WP_011590727. ABC transporter	20.704942	1.157461	18.805779	1.409412	1.100988	0.350115	0.287528	4.659431	0.426327	0.287528	4.659431	0.426327	0.170650	35.522505	0.297839	3	597	7
gene1453	NC_008261.1	1679184	1680332	-		protein_coding	CPF_RS07210	CPF_1481	.	WP_011590728. hypothetical pro	1.790639	1.796973	2.033385	1.677368	-1.135564	1.330358	1.425219	2.265694	1.241810	1.425219	2.265694	1.241810	1.275345	5.508333	1.200134	31	128	39
gene1454	NC_008261.1	1680531	1681496	-		protein_coding	CPF_RS07215	CPF_1482	.	WP_011590729. diguanylate cycl	1.813078	3.125263	2.162622	2.975862	-1.192790	2.595094	2.725379	3.583820	2.471038	2.725379	3.583820	2.471038	4.208307	16.737898	4.099453	86	327	112
gene1455	NC_008261.1	1681600	1682970	-		protein_coding	CPF_RS07220	CPF_1483	.	WP_011590730. MATE family effl	2.109503	0.633163	2.301044	0.478713	-1.090799	0.221306	0.292707	1.369611	0.167322	0.292707	1.369611	0.167322	0.172351	2.236065	0.103159	5	62	4
gene1456	NC_008261.1	1683323	1683664	-		protein_coding	CPF_RS07225	CPF_1484	.	WP_011590731. PqqD family prot	1.329592	6.536709	2.276711	6.123262	-1.712338	5.933792	6.334446	6.745430	5.558479	6.334446	6.745430	5.558479	64.408905	145.156968	39.699966	466	1004	384
gene1457	NC_008261.1	1683676	1685592	-		protein_coding	CPF_RS07230	CPF_1485	.	WP_003477897. oligopeptide tran	1.479214	8.967342	2.789798	8.481897	-1.886000	8.218975	8.689372	9.254203	7.774043	8.689372	9.254203	7.774043	353.107558	705.500386	214.692010	14320	27352	11640
gene1458	NC_008261.1	1686237	1689755	-		protein_coding	CPF_RS07235	CPF_1487	.	WP_011590733. hyaluronidase	-3.339190	3.137755	-4.485522	3.295686	1.343296	4.333478	4.125816	2.386318	4.551593	4.125816	2.386318	4.551593	12.532811	6.056034	19.381973	933	431	1929
gene1459	NC_008261.1	1690407	1695470	-		protein_coding	CPF_RS07240	CPF_1489	.	WP_011590734. DUF5011 domai	3.811302	7.063106	5.258740	6.747062	-1.456189	5.920019	6.197322	8.049841	5.655124	6.197322	8.049841	5.655124	57.575375	313.635808	42.793808	6168	32121	6129
gene1460	NC_008261.1	1695920	1697308	-		protein_coding	CPF_RS07245	CPF_1490	.	WP_003465811. MFS transporter	1.595727	0.448638	1.342564	0.652033	1.188566	0.325652	0.224068	0.898281	0.473290	0.224068	0.898281	0.473290	0.136127	1.245937	0.330922	4	35	13
gene1461	NC_008261.1	1697728	1698477	+		protein_coding	CPF_RS07250	CPF_1491	.	WP_003477901. GntR family tran	-1.244774	4.517889	-1.028281	4.382778	-1.210539	4.538674	4.678591	4.362707	4.402941	4.678591	4.362707	4.402941	19.475263	29.601612	17.396029	309	449	369
gene1462	NC_008261.1	1698591	1699772	-		protein_coding	CPF_RS07255	CPF_1492	.	WP_011590735. pyrimidine nucle	16.031503	5.647122	16.515812	5.616654	-1.030210	3.968353	3.989881	7.992718	3.946942	3.989881	7.992718	3.946942	11.397602	300.984134	12.324371	285	7195	412
gene1463	NC_008261.1	1700151	1702049	-		protein_coding	CPF_RS07260	CPF_1493	.	WP_011590736. FUSC family pro	1.431742	1.976808	1.431742	1.976808	1.000000	1.734802	1.734802	2.252573	1.734802	1.734802	2.252573	1.734802	1.792230	5.415875	1.992250	72	208	107
gene1464	NC_008261.1	1702097	1702504	-		protein_coding	CPF_RS07265	CPF_1494	.	WP_041702676. MarR family tran	2.338363	0.982734	1.646197	1.364721	1.420464	0.757347	0.545364	1.770863	1.051727	0.545364	1.770863	1.051727	0.347574	3.514537	0.953274	3	29	11
gene1465	NC_008261.1	1702642	1703739	-		protein_coding	CPF_RS07270	CPF_1495	.	WP_011590738. peptidase S41	1.565226	4.203827	1.341129	4.322511	1.167096	4.002956	3.893046	4.539417	4.115969	3.893046	4.539417	4.115969	10.547508	32.378508	13.975662	245	719	434
gene1466	NC_008261.1	1704071	1704523	+		protein_coding	CPF_RS07275	CPF_1496	.	WP_011590739. DUF1097 domai	1.096669	3.538985	1.075850	3.553046	1.019351	3.486845	3.473047	3.606175	3.500698	3.473047	3.606175	3.500698	7.617470	17.136879	8.897985	73	157	114
gene1467	NC_008261.1	1704972	1707809	-		protein_coding	CPF_RS07280	CPF_1497	.	WP_011590740. ATPase	-1.105293	4.779927	-1.114589	4.785875	1.008411	4.858725	4.852687	4.708258	4.864770	4.852687	4.708258	4.864770	21.952760	36.831794	24.157413	1318	2114	1939
gene1468	NC_008261.1	1708703	1709063	-	ssrA	tmRNA	CPF_RS14775	CPF_3016	.	transfer-messe	1.000000	17.070259	1.000000	17.070259	1.000000	17.070259	17.070259	17.070259	17.070259	17.070259	17.070259	17.070259	##########	##########	##########	1334311	741288	1499845
gene1469	NC_008261.1	1709391	1709969	-		protein_coding	CPF_RS07285	CPF_1498	.	WP_003465824. Xanthine phosph	1.093962	8.728433	1.402307	8.546074	-1.281861	8.482881	8.663891	8.793454	8.305652	8.663891	8.793454	8.305652	341.422056	529.472497	320.662718	4182	6200	5251
gene1470	NC_008261.1	1710142	1711311	+		protein_coding	CPF_RS07290	CPF_1499	.	WP_003456479. tetratricopeptid	1.003459	0.831852	-1.271598	0.994723	1.215289	0.991749	0.829264	0.834346	1.185927	0.829264	0.834346	1.185927	0.572970	1.148743	1.107163	12	23	31
gene1471	NC_008261.1	1711478	1712086	-		protein_coding	CPF_RS07295	CPF_1500	.	WP_011590742. hypothetical pro	-2.813822	4.072114	-2.481573	3.995863	-1.133886	4.794702	4.886197	3.393665	4.704920	4.886197	3.393665	4.704920	22.431931	14.208592	21.713975	289	175	374
gene1472	NC_008261.1	1712535	1713005	-		protein_coding	CPF_RS07300	CPF_1501	.	WP_003456486. SsrA-binding pr	1.000000	8.651820	1.147062	8.552276	-1.147062	8.552276	8.651820	8.651820	8.453877	8.651820	8.651820	8.453877	337.313493	483.960926	351.400802	3361	4610	4681
gene1473	NC_008261.1	1713602	1715857	-		protein_coding	CPF_RS07305	CPF_1502	.	WP_011590743. ribonuclease R	-1.128625	7.046129	-1.270335	7.129902	1.125560	7.218770	7.133953	6.959387	7.304596	7.133953	6.959387	7.304596	116.205552	163.570367	146.650162	5546	7463	9357
gene1474	NC_008261.1	1716146	1716376	-		protein_coding	CPF_RS07310	CPF_1503	.	WP_003477916. preprotein trans	1.142407	9.022093	1.109672	9.043526	1.029500	8.947513	8.926566	9.118643	8.968509	8.926566	9.118643	8.968509	410.492340	645.365310	495.315077	2006	3015	3236
gene1475	NC_008261.1	1716567	1717124	-		protein_coding	CPF_RS07315	CPF_1504	.	WP_011590744. tryptophan tran	1.379250	0.733361	1.540576	0.614862	-1.116967	0.450417	0.537224	1.001107	0.377637	0.537224	1.001107	0.377637	0.338853	1.417804	0.253461	4	16	4
gene1476	NC_008261.1	1718717	1720012	-		protein_coding	CPF_RS07320	CPF_1505	.	WP_003460354. enolase	-1.422996	12.371491	-1.130197	12.207610	-1.259069	12.461287	12.628574	12.119642	12.296216	12.628574	12.119642	12.296216	6274.698596	5364.269638	4810.950065	172033	140600	176340
gene1477	NC_008261.1	1720740	1722197	-		protein_coding	CPF_RS07325	CPF_1507	.	WP_011590745. glutamate:gamm	-5.372842	9.484496	-4.991339	9.437613	-1.076433	10.721311	10.774571	8.348886	10.668313	10.774571	8.348886	10.668313	1474.125421	379.356557	1666.641681	45468	11186	68725
gene1478	NC_008261.1	1722763	1724301	-		protein_coding	CPF_RS07330	CPF_1508	.	WP_011590746. 2,3-bisphosphog	-1.312742	9.972788	-1.006156	9.782854	-1.304710	9.977302	10.171011	9.778428	9.787282	10.171011	9.778428	9.787282	1009.226426	963.247244	911.674793	32858	29981	39682
gene1479	NC_008261.1	1724426	1725172	-		protein_coding	CPF_RS07335	CPF_1509	.	WP_003450470. triose-phosphat	-1.528368	11.398947	-1.371215	11.322495	-1.114608	11.630518	11.709049	11.097057	11.552513	11.709049	11.097057	11.552513	2993.770153	2651.213664	2895.078153	47310	40053	61164
gene1480	NC_008261.1	1725416	1726609	-		protein_coding	CPF_RS07340	CPF_1510	.	WP_003450483. phosphoglycerat	-1.502175	11.726980	-1.365376	11.659611	-1.100192	11.955104	12.024180	11.437126	11.886425	12.024180	11.437126	11.886425	3586.902452	3311.011718	3615.584923	90602	79953	122095
gene1481	NC_008261.1	1726753	1727751	-		protein_coding	CPF_RS07345	CPF_1511	.	WP_003460339. type I glyceralde	-1.080020	13.827097	-1.080020	13.827097	1.000000	13.882738	13.882738	13.771680	13.882738	13.882738	13.771680	13.882738	16379.287004	13300.699452	15265.784333	346158	268726	431320
gene1482	NC_008261.1	1727842	1728876	-		protein_coding	CPF_RS07350	CPF_1512	.	WP_003450474. sugar-binding do	1.549651	8.452921	1.894359	8.301159	-1.222442	7.996658	8.142853	8.774796	7.853087	8.142853	8.774796	7.853087	243.977424	525.416426	227.170820	5342	10998	6650
gene1483	NC_008261.1	1729103	1730488	-		protein_coding	CPF_RS07355	CPF_1513	.	WP_011590747. RNA polymerase	-1.243397	5.255332	-1.243397	5.255332	1.000000	5.414824	5.414824	5.100537	5.414824	5.414824	5.100537	5.414824	32.741164	48.125915	36.250655	960	1349	1421
gene1484	NC_008261.1	1730739	1731506	-		protein_coding	CPF_RS07360	CPF_1514	.	WP_011590748. hypothetical pro	1.248767	4.612274	1.452937	4.497757	-1.163497	4.344198	4.454805	4.775309	4.236337	4.454805	4.775309	4.236337	16.495280	38.887140	15.422991	268	604	335
gene1485	NC_008261.1	1731487	1732002	-		protein_coding	CPF_RS07365	CPF_1515	.	WP_003450471. ECF transporter	1.466777	4.224737	1.610912	4.151929	-1.098267	3.889238	3.957440	4.510089	3.822212	3.957440	4.510089	3.822212	11.084645	32.101504	11.306268	121	335	165
gene1486	NC_008261.1	1732604	1733674	-		protein_coding	CPF_RS07370	CPF_1516	.	WP_003470182. BMP family ABC	1.189007	0.788390	1.070748	0.872395	1.110445	0.745087	0.673341	0.923097	0.824478	0.673341	0.923097	0.824478	0.441364	1.292703	0.660276	10	28	20
gene1487	NC_008261.1	1733765	1734604	-		protein_coding	CPF_RS07375	CPF_1517	.	WP_003470185. DegV domain-co	3.826526	5.632221	3.045112	5.824446	1.256613	4.908790	4.746785	6.682820	5.076325	4.746785	6.682820	5.076325	20.314869	140.744221	28.875522	361	2391	686
gene1488	NC_008261.1	1734619	1735080	-		protein_coding	CPF_RS07380	CPF_1518	.	WP_003450463. tRNA (cytidine(3	3.117346	5.452954	2.884165	5.517717	1.080849	4.749879	4.694128	6.334446	4.806293	4.694128	6.334446	4.806293	19.644698	111.627864	23.342259	192	1043	305
gene1489	NC_008261.1	1735169	1736002	-		protein_coding	CPF_RS07385	CPF_1519	.	WP_003473016. Cof-type HAD-I	3.129120	4.449859	2.421373	4.666873	1.292292	3.892988	3.702425	5.348182	4.072357	3.702425	5.348182	4.072357	9.125274	58.042608	13.439349	161	979	317
gene1490	NC_008261.1	1736160	1737722	-		protein_coding	CPF_RS07390	CPF_1520	.	WP_011590749. VanW domain-c	1.221253	8.364382	1.025100	8.491904	1.191350	8.346786	8.221443	8.509805	8.474040	8.221443	8.509805	8.474040	254.919574	433.561133	353.464712	8429	13705	15625
gene1491	NC_008261.1	1737887	1738952	-		protein_coding	CPF_RS07395	CPF_1521	.	WP_003481377. muramidase	4.744842	4.961221	2.941470	5.375638	1.613085	4.294675	3.963592	6.209952	4.653415	3.963592	6.209952	4.653415	11.156907	103.959236	20.826685	228	2031	569
gene1492	NC_008261.1	1738926	1739831	-		protein_coding	CPF_RS07400	CPF_1522	.	WP_003481378. D-alanine--D-a	3.701451	4.920067	2.629266	5.210075	1.407788	4.305426	4.065773	5.953864	4.559204	4.065773	5.953864	4.559204	12.000124	90.214207	19.474098	230	1653	499
gene1493	NC_008261.1	1739951	1742416	-		protein_coding	CPF_RS07405	CPF_1523	.	WP_025647990. PAS domain-co	1.267599	3.370388	1.044866	3.513974	1.213170	3.340159	3.203676	3.545775	3.482457	3.203676	3.545775	3.482457	6.249002	16.181201	8.731910	326	807	609
gene1494	NC_008261.1	1742516	1743379	+		protein_coding	CPF_RS07410	CPF_1524	.	WP_011590751. EamA/RhaT fam	1.522486	4.242868	1.464509	4.272841	1.039588	3.978382	3.950474	4.556903	4.006486	3.950474	4.556903	4.006486	11.051564	32.849475	12.809011	202	574	313
gene1495	NC_008261.1	1743409	1744038	-		protein_coding	CPF_RS07415	CPF_1525	.	WP_011590752. endonuclease II	-1.364320	6.745727	-1.356643	6.741789	-1.005659	6.969468	6.973539	6.525358	6.965399	6.973539	6.525358	6.965399	102.268389	127.852668	114.772483	1363	1629	2045
gene1496	NC_008261.1	1744050	1744754	-		protein_coding	CPF_RS07420	CPF_1526	.	WP_003477926. 1-acyl-sn-glyce	-1.003305	6.922793	1.008080	6.914606	-1.011412	6.916984	6.925174	6.920413	6.908803	6.925174	6.920413	6.908803	98.093745	160.260823	109.935118	1463	2285	2192
gene1497	NC_008261.1	1744792	1745784	-		protein_coding	CPF_RS07425	CPF_1527	.	WP_011010387. tRNA epoxyquer	-1.410741	4.541134	-1.249429	4.457427	-1.129109	4.707732	4.796139	4.299686	4.620955	4.796139	4.299686	4.620955	21.040649	28.382843	20.224794	442	570	568
gene1498	NC_008261.1	1744929	1746438	-		protein_coding	CPF_RS07430	CPF_1528	.	WP_003450775. serine O-acetyl	53.489691	6.818582	25.477189	7.581814	2.099866	5.034317	4.527532	10.268963	5.597829	4.527532	10.268963	5.597829	17.425040	1365.967208	40.557456	188	14089	585
gene1499	NC_008261.1	1746457	1747359	-		protein_coding	CPF_RS07435	CPF_1529	.	WP_011010388. cysteine syntha	42.464082	7.326707	22.821408	7.943522	1.860713	5.535532	5.105698	10.513869	6.001553	5.105698	10.513869	6.001553	26.173895	1640.475857	54.896579	500	29959	1402
gene1500	NC_008261.1	1747698	1748969	-		protein_coding	CPF_RS07440	CPF_1530	.	WP_003477931. CBS domain-co	1.228044	4.766833	-1.113102	4.994011	1.366938	4.841181	4.620955	4.917317	5.071902	4.620955	4.917317	5.071902	18.655320	42.798691	28.797690	502	1101	1036
gene1501	NC_008261.1	1749309	1750097	+		protein_coding	CPF_RS07445	CPF_1531	.	WP_011590753. amino acid ABC	-1.170451	0.261693	-1.069325	0.214699	-1.094571	0.327179	0.398791	0.171726	0.268426	0.398791	0.171726	0.268426	0.239645	0.188007	0.179254	4	3	4
gene1502	NC_008261.1	1750111	1750752	+		protein_coding	CPF_RS07450	CPF_1532	.	WP_003481390. amino acid ABC	-1.485808	0.569905	-1.241645	0.483388	-1.196645	0.782963	0.923097	0.351850	0.664102	0.923097	0.351850	0.664102	0.662646	0.385093	0.495670	9	5	9
gene1503	NC_008261.1	1750764	1751455	+		protein_coding	CPF_RS07455	CPF_1533	.	WP_011590754. amino acid ABC	-1.338585	0.834252	-1.187784	0.764128	-1.126954	0.980718	1.070718	0.650009	0.898281	1.070718	0.650009	0.898281	0.818529	0.836206	0.765320	12	12	15
gene1504	NC_008261.1	1751625	1752305	+		protein_coding	CPF_RS07460	CPF_1534	.	WP_011590755. hypothetical pro	1.322850	0.263475	1.175950	0.400071	1.124921	0.197499	0.130067	0.533717	0.299891	0.130067	0.533717	0.299891	0.069413	0.653470	0.207682	1	9	4
gene1505	NC_008261.1	1752378	1753259	-		protein_coding	CPF_RS07465	CPF_1535	.	WP_003460604. lipid kinase	1.456077	7.006867	1.451676	7.009136	1.003031	6.743247	6.741064	7.283150	6.745430	6.741064	7.283150	6.745430	85.482696	202.492731	96.452181	1595	3612	2406
gene1506	NC_008261.1	1753574	1754095	+		protein_coding	CPF_RS07470	CPF_1536	.	WP_003453570. CDP-diacylglyce	1.692779	8.497328	1.752302	8.471220	-1.035163	8.101143	8.126110	8.885503	8.076252	8.126110	8.885503	8.076252	241.421392	572.795696	261.390193	2666	6047	3859
gene1507	NC_008261.1	1754142	1754492	-		protein_coding	CPF_RS07475	CPF_1537	.	WP_003453576. hypothetical pro	-2.854434	2.131028	-2.302481	2.018609	-1.239721	2.858751	3.017958	1.504753	2.707942	3.017958	1.504753	2.707942	5.386901	2.676559	5.036721	40	19	50
gene1508	NC_008261.1	1754514	1755050	-		protein_coding	CPF_RS07480	CPF_1538	.	WP_003460610. rubrerythrin fam	-4.405638	2.835138	-2.895184	2.617349	-1.521713	3.784947	4.099892	1.960541	3.494196	4.099892	1.960541	3.494196	12.235638	4.143511	8.822985	139	45	134
gene1509	NC_008261.1	1755102	1756451	-		protein_coding	CPF_RS07485	CPF_1539	.	WP_003460596. stage V sporulat	-4.696007	2.320807	-3.257562	2.148519	-1.441571	3.416794	3.690785	1.459350	3.163143	3.690785	1.459350	3.163143	9.033833	2.563861	7.045365	258	70	269
gene1510	NC_008261.1	1756478	1757656	-		protein_coding	CPF_RS07490	CPF_1540	.	WP_003460611. sporulation prot	-4.810312	2.078906	-3.500012	1.937902	-1.374370	3.263261	3.500698	1.234568	3.041928	3.500698	1.234568	3.041928	7.738016	1.971126	6.447772	193	47	215
gene1511	NC_008261.1	1757660	1759582	-		protein_coding	CPF_RS07495	CPF_1541	.	WP_003460607. serine protein ki	-5.527565	2.176503	-3.861332	2.020866	-1.431518	3.466553	3.734971	1.268327	3.217426	3.734971	1.268327	3.217426	9.463844	2.057032	7.244392	385	80	394
gene1512	NC_008261.1	1759672	1761450	-		protein_coding	CPF_RS07500	CPF_1542	.	WP_011590757. DNA helicase Re	-1.104957	3.823166	-1.107837	3.825009	1.002607	3.897717	3.895839	3.751849	3.899595	3.895839	3.751849	3.899595	10.601884	18.872269	11.925053	399	679	600
gene1513	NC_008261.1	1761681	1762304	-		protein_coding	CPF_RS07505	CPF_1543	.	WP_003460600. thiamine phosph	3.128038	9.030497	3.405029	8.963213	-1.088551	8.183826	8.245259	9.890517	8.122850	8.245259	9.890517	8.122850	259.076263	1035.590479	270.793013	3420	13069	4779
gene1514	NC_008261.1	1762297	1763094	-		protein_coding	CPF_RS07510	CPF_1544	.	WP_003460606. hydroxyethylthia	2.853955	8.363300	3.345199	8.236951	-1.172128	7.525525	7.640962	9.153925	7.411833	7.640962	9.153925	7.411833	162.009525	660.703791	161.679883	2735	10663	3649
gene1515	NC_008261.1	1763106	1763900	-		protein_coding	CPF_RS07515	CPF_1545	.	WP_011590758. bifunctional hyd	3.161805	8.332885	3.750325	8.195742	-1.186134	7.419627	7.543782	9.204530	7.297515	7.543782	9.204530	7.297515	152.334442	685.960785	145.255996	2562	11029	3266
gene1516	NC_008261.1	1764207	1765046	+		protein_coding	CPF_RS07520	CPF_1546	.	WP_003460605. patatin family pro	-1.146346	4.466620	-1.149159	4.468349	1.002454	4.567995	4.566227	4.369185	4.569763	4.566227	4.369185	4.569763	17.895092	29.608675	19.657243	318	503	467
gene1517	NC_008261.1	1765164	1766177	-		protein_coding	CPF_RS07525	CPF_1547	.	WP_003470312. LacI family DNA-	-1.194207	6.090506	1.361055	5.737154	-1.625381	5.859021	6.219878	5.963824	5.519100	6.219878	5.963824	5.519100	58.831173	90.748342	38.879612	1262	1861	1115
gene1518	NC_008261.1	1766395	1767459	+		protein_coding	CPF_RS07530	CPF_1548	.	WP_003477945. galactose/gluco	-6.924955	10.841207	-7.343886	10.878407	-1.060496	12.368904	12.326607	9.534802	12.411346	12.326607	9.534802	12.411346	4787.235989	828.137565	5144.706686	107857	17837	154962
gene1519	NC_008261.1	1767534	1769081	+		protein_coding	CPF_RS07535	CPF_1549	.	WP_003449162. galactose/meth	-8.825074	8.406266	-8.213376	8.363125	-1.074476	10.070623	10.122572	6.980964	10.018939	10.122572	6.980964	10.018939	975.662486	164.787723	1121.376169	31951	5159	49095
gene1520	NC_008261.1	1769053	1770081	+		protein_coding	CPF_RS07540	CPF_1550	.	WP_003449090. galactose/meth	-7.857953	8.616117	-8.809816	8.528701	-1.153916	10.126786	10.230581	7.256427	10.024043	10.230581	7.256427	10.024043	1032.545216	198.744645	1130.693077	22477	4136	32906
gene1521	NC_008261.1	1770121	1771143	-		protein_coding	CPF_RS07545	CPF_1551	.	WP_011590759. galactose-1-ep	-3.868872	5.879579	-3.738476	5.858576	-1.034880	6.911209	6.935985	4.984072	6.886522	6.935985	4.984072	6.886522	99.883595	44.467475	107.110231	2140	920	3099
gene1522	NC_008261.1	1771369	1772532	+		protein_coding	CPF_RS07550	CPF_1552	.	WP_003465923. galactokinase	-12.650070	6.246429	-8.799801	6.047161	-1.437540	8.073619	8.339664	4.678591	7.816062	8.339664	4.678591	7.816062	275.051618	35.895004	220.803881	6773	845	7269
gene1523	NC_008261.1	1772655	1774151	+		protein_coding	CPF_RS07555	CPF_1553	.	WP_011590760. galactose-1-ph	-14.813354	5.221079	-11.908306	5.110521	-1.243951	7.356655	7.515805	3.626979	7.200875	7.515805	3.626979	7.200875	147.904434	17.307716	133.991114	4684	524	5673
gene1524	NC_008261.1	1774342	1775997	-		protein_coding	CPF_RS07560	CPF_1554	.	WP_003460743. nucleoside kinas	-1.543403	5.764630	-1.940622	5.919036	1.257366	6.249201	6.086182	5.460067	6.416586	6.086182	5.460067	6.416586	52.322470	62.792709	74.580153	1833	2103	3493
gene1525	NC_008261.1	1776128	1776826	+		protein_coding	CPF_RS07565	CPF_1555	.	WP_003472738. DUF421 domain	1.035842	0.242752	1.023354	0.252278	1.012203	0.227256	0.218675	0.269479	0.236174	0.218675	0.269479	0.236174	0.135251	0.282952	0.151750	2	4	3
gene1527	NC_008261.1	1777354	1778220	+		protein_coding	CPF_RS07575	CPF_1557	.	WP_003449129. fructose-1,6-bi	1.036020	10.856161	1.280305	10.701989	-1.235792	10.676855	10.830665	10.881717	10.525229	10.830665	10.881717	10.525229	1518.966244	2334.393948	1512.391404	27860	40932	37085
gene1528	NC_008261.1	1778267	1779310	-		protein_coding	CPF_RS07580	CPF_1558	.	WP_011590762. nucleoid-associ	3.194734	2.546841	2.152210	2.914065	1.484397	2.109046	1.843269	3.518965	2.413146	1.843269	3.518965	2.413146	1.946947	15.960885	3.928642	43	337	116
gene1529	NC_008261.1	1779450	1780013	+		protein_coding	CPF_RS07585	CPF_1559	.	WP_011590763. CYTH domain-co	1.157538	5.236529	1.219787	5.197832	-1.053777	5.094137	5.132062	5.343122	5.056493	5.132062	5.343122	5.056493	26.484641	57.949893	28.524452	316	661	455
gene1530	NC_008261.1	1780263	1780865	-		protein_coding	CPF_RS07590	CPF_1561	.	WP_041702689. hypothetical pro	1.424389	4.353541	2.254370	3.986935	-1.582693	3.760093	4.105842	4.616185	3.443460	4.105842	4.616185	3.443460	12.307460	34.603930	8.560922	157	422	146
gene1531	NC_008261.1	1781006	1782667	-		protein_coding	CPF_RS07595	CPF_1562	.	WP_011590766. enterotoxin	1.627791	8.557506	2.349354	8.277146	-1.443278	7.944181	8.213263	8.916178	7.683914	8.213263	8.916178	7.683914	251.879427	578.683627	197.446194	8856	19451	9281
gene1534	NC_008261.1	1783902	1784054	-		protein_coding	CPF_RS07610	CPF_1565	.	WP_003481409. hypothetical pro	2.080241	3.318976	2.941605	3.011910	-1.414070	2.570347	2.832396	3.889146	2.332543	2.832396	3.889146	2.332543	4.634319	21.006428	3.697546	15	65	16
gene1535	NC_008261.1	1784095	1784580	-		protein_coding	CPF_RS07615	CPF_1566	.	WP_011590768. Cys-tRNA(Pro)	2.204445	4.458304	2.512543	4.349779	-1.139762	3.828884	3.924413	5.064828	3.735680	3.924413	5.064828	3.735680	10.796247	46.495424	10.549133	111	457	145
gene1536	NC_008261.1	1784747	1785040	-		protein_coding	CPF_RS07620	CPF_1567	.	WP_003460283. hypothetical pro	4.929208	1.470603	2.575108	2.233756	1.914175	1.088474	0.716602	3.017958	1.653325	0.716602	3.017958	1.653325	0.468020	10.044018	1.867078	3	64	16
gene1537	NC_008261.1	1785241	1786269	-		protein_coding	CPF_RS07625	CPF_1568	.	WP_011590769. autolytic lysozy	1.130435	1.098480	1.414399	0.906512	-1.251199	0.836462	1.013595	1.190473	0.690284	1.013595	1.190473	0.690284	0.780944	1.874043	0.515420	17	39	15
gene1538	NC_008261.1	1786323	1786805	-		protein_coding	CPF_RS07630	CPF_1569	.	WP_011590770. cobalt ABC tran	1.469722	0.859825	1.817814	0.614023	-1.236842	0.446905	0.625808	1.181351	0.319146	0.625808	1.181351	0.319146	0.391470	1.842704	0.219614	4	18	3
gene1540	NC_008261.1	1787141	1787326	-		protein_coding	CPF_RS07640	CPF_1571	.	WP_011590772. hypothetical pro	1.101926	3.071917	1.221094	2.995177	-1.108145	2.927691	3.002701	3.142728	2.854554	3.002701	3.142728	2.854554	5.336942	11.431041	5.512772	21	43	29
gene1541	NC_008261.1	1787345	1789843	-		protein_coding	CPF_RS07645	CPF_1572	.	WP_011590773. DUF2479 domai	1.576321	1.696091	1.488049	1.745751	1.059120	1.440257	1.399288	2.055849	1.482426	1.399288	2.055849	1.482426	1.248429	4.531057	1.542216	66	229	109
gene1542	NC_008261.1	1789859	1791739	-		protein_coding	CPF_RS07650	CPF_1573	.	WP_011590774. DUF2479 domai	1.230308	2.107925	1.814769	1.781708	-1.475053	1.659812	1.963710	2.262730	1.402944	1.963710	2.262730	1.402944	2.161204	5.493989	1.428597	86	209	76
gene1543	NC_008261.1	1791790	1794849	-		protein_coding	CPF_RS07655	CPF_1574	.	WP_011590775. peptidase M23	1.208233	2.242728	1.666997	1.980688	-1.379698	1.863844	2.110426	2.383325	1.646073	2.110426	2.383325	1.646073	2.425294	6.043388	1.860328	157	374	161
gene1544	NC_008261.1	1794854	1795615	-		protein_coding	CPF_RS07660	CPF_1575	.	WP_041702692. tail protein	1.139683	0.496675	1.094478	0.530765	1.041303	0.439461	0.411235	0.599867	0.469624	0.411235	0.599867	0.469624	0.248137	0.778676	0.324809	4	12	7
gene1545	NC_008261.1	1795557	1798811	-		protein_coding	CPF_RS07665	CPF_1576	.	WP_011590777. phage tail tape m	-1.098613	0.945811	1.215058	0.726422	-1.334878	0.780393	1.016082	0.880399	0.599374	1.016082	0.880399	0.599374	0.784204	1.215260	0.423642	54	80	39
gene1547	NC_008261.1	1799362	1799679	-		protein_coding	CPF_RS07675	CPF_1578	.	WP_011590779. hypothetical pro	-1.010854	1.260515	1.044114	1.221216	-1.055447	1.228783	1.268327	1.252752	1.190473	1.268327	1.252752	1.190473	1.040546	2.021373	1.111880	7	13	10
gene1548	NC_008261.1	1799682	1800284	-		protein_coding	CPF_RS07680	CPF_1579	.	WP_011590780. tail protein	-1.197839	1.105534	-1.157036	1.083078	-1.035265	1.218137	1.243393	0.982960	1.193394	1.243393	0.982960	1.193394	1.019089	1.393997	1.114093	13	17	19
gene1549	NC_008261.1	1800301	1800648	-		protein_coding	CPF_RS07685	CPF_1580	.	WP_011590781. hypothetical pro	1.039661	0.246238	1.082564	0.211040	-1.041267	0.188360	0.219775	0.275888	0.161435	0.219775	0.275888	0.161435	0.135833	0.284172	0.101603	1	2	1
gene1550	NC_008261.1	1800657	1801094	-		protein_coding	CPF_RS07690	CPF_1581	.	WP_041702697. hypothetical pro	1.083144	0.963924	1.206524	0.877438	-1.113909	0.826561	0.908031	1.023256	0.752399	0.908031	1.023256	0.752399	0.647535	1.467572	0.565079	6	13	7
gene1551	NC_008261.1	1801094	1801423	-		protein_coding	CPF_RS07695	CPF_1582	.	WP_011590783. phage head-tail	-1.056628	0.435391	1.165932	0.264476	-1.231956	0.289711	0.476934	0.397467	0.175983	0.476934	0.397467	0.175983	0.286485	0.449508	0.107145	2	3	1
gene1553	NC_008261.1	1801706	1802890	-		protein_coding	CPF_RS07705	CPF_1584	.	WP_011590785. phage major cap	-1.294515	1.448577	1.433943	0.980408	-1.856261	1.114500	1.646702	1.274290	0.754302	1.646702	1.274290	0.754302	1.595614	2.086325	0.566918	40	50	19
gene1554	NC_008261.1	1802931	1803536	-		protein_coding	CPF_RS07710	CPF_1585	.	WP_011590786. HK97 family pha	-1.188146	1.542876	1.303587	1.217349	-1.548853	1.319416	1.672236	1.423523	1.041036	1.672236	1.423523	1.041036	1.638071	2.447817	0.933539	21	30	16
gene1555	NC_008261.1	1803526	1804793	-		protein_coding	CPF_RS07715	CPF_1586	.	WP_011590787. phage portal pro	-1.051795	0.782677	-1.091603	0.807851	1.037847	0.846324	0.819951	0.747097	0.873545	0.819951	0.747097	0.873545	0.568150	1.030123	0.736620	15	26	26
gene1556	NC_008261.1	1804774	1806513	-		protein_coding	CPF_RS07720	CPF_1587	.	WP_011590788. terminase large	-1.129211	1.312362	1.157929	1.116858	-1.307546	1.193946	1.402944	1.227629	1.016082	1.402944	1.227629	1.016082	1.249668	1.960786	0.894105	46	69	44
gene1557	NC_008261.1	1806506	1807018	-		protein_coding	CPF_RS07725	CPF_1588	.	WP_011590789. phage terminase	-1.154842	0.583167	1.141495	0.381261	-1.318247	0.455152	0.696189	0.488493	0.297568	0.696189	0.488493	0.297568	0.460722	0.578315	0.206771	5	6	3
gene1558	NC_008261.1	1807109	1807525	-		protein_coding	CPF_RS14780	.	.	WP_041702752. HNH endonuclea	1.898794	3.279612	2.241430	3.138862	-1.180450	2.727235	2.849527	3.774610	2.610191	2.849527	3.774610	2.610191	4.761013	19.090623	4.578706	42	161	54
gene1559	NC_008261.1	1807521	1808342	-		protein_coding	CPF_RS07735	CPF_1589	.	WP_011590790. hypothetical pro	1.682297	3.600094	2.363831	3.316692	-1.405121	2.988978	3.244378	3.994810	2.753684	3.244378	3.994810	2.753684	6.440689	22.858202	5.204734	112	380	121
gene1560	NC_008261.1	1808517	1809065	-		protein_coding	CPF_RS07740	CPF_1590	.	WP_011590791. site-specific int	1.760248	2.841775	1.641722	2.899212	1.072196	2.512790	2.463009	3.278788	2.563578	2.463009	3.278788	2.563578	3.444084	12.789285	4.379470	40	142	68
gene1561	NC_008261.1	1809160	1809513	-		protein_coding	CPF_RS07745	CPF_1591	.	WP_011590792. hypothetical pro	1.357593	5.242401	-1.034820	5.492197	1.404864	5.266021	5.026512	5.467563	5.516943	5.026512	5.467563	5.516943	24.836809	63.273990	38.853608	186	453	389
gene1562	NC_008261.1	1809572	1809757	-		protein_coding	CPF_RS07750	CPF_1592	.	WP_041702754. hypothetical pro	-2.643042	1.992804	1.830441	0.872416	-4.837932	1.231762	2.813636	1.411436	0.539245	2.813636	1.411436	0.539245	4.574521	2.392544	0.380191	18	9	2
gene1563	NC_008261.1	1809920	1810381	-		protein_coding	CPF_RS14675	CPF_1593	.	WP_011590794. sigma-70 family	-1.441583	4.947622	-1.200742	4.820982	-1.200576	5.084905	5.218478	4.690825	4.954751	5.218478	4.690825	4.954751	28.034621	36.067680	26.097411	274	337	341
gene1564	NC_008261.1	1810917	1811306	-		protein_coding	CPF_RS07760	CPF_1595	.	WP_011590796. single-stranded	-1.698089	2.030083	-1.571058	1.983018	-1.080857	2.390913	2.447659	1.683747	2.335483	2.447659	1.683747	2.335483	3.393748	3.296393	3.717100	28	26	41
gene1565	NC_008261.1	1811479	1812762	-		protein_coding	CPF_RS07765	CPF_1597	.	WP_011590798. DNA helicase	-1.339372	1.250172	-1.174285	1.167189	-1.140585	1.380450	1.478594	1.057038	1.288820	1.478594	1.057038	1.288820	1.362143	1.540371	1.266712	37	40	46
gene1566	NC_008261.1	1812773	1813513	-		protein_coding	CPF_RS07770	CPF_1598	.	WP_011590799. hypothetical pro	-1.229913	0.440757	-1.391132	0.500434	1.131082	0.697846	0.614628	0.316072	0.792332	0.614628	0.316072	0.792332	0.382753	0.333643	0.620312	6	5	13
gene1567	NC_008261.1	1813702	1813881	-		protein_coding	CPF_RS07775	CPF_1600	.	WP_011590801. hypothetical pro	-1.399261	1.012724	1.428386	0.476850	-1.998685	0.604417	1.283646	0.798982	0.284595	1.283646	0.798982	0.284595	1.050446	1.098798	0.196432	4	4	1
gene1568	NC_008261.1	1813907	1814137	-		protein_coding	CPF_RS07780	CPF_1601	.	WP_011590802. hypothetical pro	-1.416551	0.576479	-1.047229	0.409575	-1.352686	0.625233	0.880020	0.377637	0.444214	0.880020	0.377637	0.444214	0.613897	0.428103	0.306128	3	2	2
gene1569	NC_008261.1	1814143	1814640	-		protein_coding	CPF_RS07785	CPF_1602	.	WP_011590803. hypothetical pro	-1.248870	0.421956	-1.244422	0.420177	-1.003574	0.609115	0.611694	0.291072	0.606547	0.611694	0.291072	0.606547	0.379679	0.297867	0.425997	4	3	6
gene1571	NC_008261.1	1815028	1815372	+		protein_coding	CPF_RS07795	CPF_1604	.	WP_011590805. XRE family trans	-1.495533	4.861305	-1.260806	4.743866	-1.186172	5.035635	5.160297	4.579638	4.913984	5.160297	4.579638	4.913984	26.991887	33.393897	25.314122	197	233	247
gene1574	NC_008261.1	1817080	1817934	+		protein_coding	CPF_RS07805	CPF_1606	.	WP_011590807. DNA methyltran	1.724543	4.716240	1.443446	4.853727	1.194740	4.465992	4.339487	5.125701	4.596184	4.339487	5.125701	4.596184	14.650953	49.041079	19.932691	265	848	482
gene1575	NC_008261.1	1818046	1818543	+		protein_coding	CPF_RS07810	CPF_1607	.	WP_011590808. ImmA/IrrE famil	-1.559299	2.644301	-1.944927	2.781974	1.247309	3.139460	2.984095	2.343198	3.302914	2.984095	2.343198	3.302914	5.220588	5.858048	7.738952	55	59	109
gene1576	NC_008261.1	1818556	1820064	+		protein_coding	CPF_RS07815	CPF_1608	.	WP_011590809. resolvase	-1.006682	2.466230	-1.008027	2.467191	1.001337	2.472002	2.471038	2.461431	2.472966	2.471038	2.461431	2.472966	3.477121	6.422396	4.100472	111	196	175
gene1577	NC_008261.1	1820078	1820416	-		protein_coding	CPF_RS07820	CPF_1609	.	WP_011590810. aminoacyl-tRNA	1.568256	4.851004	1.454789	4.908583	1.077995	4.591126	4.537270	5.186431	4.645621	4.537270	5.186431	4.645621	17.569401	51.925487	20.651448	126	356	198
gene1578	NC_008261.1	1820820	1821197	-		protein_coding	CPF_RS07825	CPF_1610	.	WP_011590811. hypothetical pro	2.901536	1.318327	1.792568	1.825393	1.618648	1.048875	0.757513	2.294330	1.452302	0.757513	2.294330	1.452302	0.500212	5.624798	1.496626	4	43	16
gene1579	NC_008261.1	1821673	1822233	-		protein_coding	CPF_RS07830	CPF_1612	.	WP_011590813. hypothetical pro	-2.197471	4.984329	-2.929797	5.166192	1.333258	5.788263	5.584502	4.448658	5.999458	5.584502	4.448658	5.999458	37.158813	30.672323	54.895950	441	348	871
gene1580	NC_008261.1	1822317	1823267	-		protein_coding	CPF_RS07835	CPF_1613	.	WP_011590814. ribonuclease Z	-1.090025	3.695220	-1.263293	3.798417	1.158958	3.862872	3.757923	3.633562	3.970751	3.757923	3.633562	3.970751	9.593187	17.365857	12.455160	193	334	335
gene1581	NC_008261.1	1823380	1827816	-		protein_coding	CPF_RS07840	CPF_1614	.	WP_041702699. glycosyl hydrolas	-3.979902	5.336532	-4.657493	5.429910	1.170253	6.537542	6.425115	4.432383	6.651936	6.425115	4.432383	6.651936	68.524001	30.512254	89.466265	6432	2738	11227
gene1582	NC_008261.1	1828029	1828655	+		protein_coding	CPF_RS07845	CPF_1615	.	WP_003449118. potassium trans	1.840072	5.121323	2.386427	4.912731	-1.296921	4.508855	4.700299	5.580061	4.325209	4.700299	5.580061	4.325209	19.677009	68.924587	16.466463	261	874	292
gene1583	NC_008261.1	1828707	1829459	-		protein_coding	CPF_RS07850	CPF_1616	.	WP_011590816. hypothetical pro	-1.441728	5.283825	1.222399	4.879504	-1.762367	5.129292	5.554310	5.026512	4.736796	5.554310	5.026512	4.736796	36.409870	45.374657	22.069266	580	691	470
gene1584	NC_008261.1	1829551	1830699	-		protein_coding	CPF_RS07855	CPF_1617	.	WP_011590817. YibE/F family pro	-1.679298	4.804779	1.200046	4.311827	-2.015235	4.660431	5.193237	4.445378	4.182289	5.193237	4.445378	4.182289	27.563914	30.640105	14.709330	670	712	478
gene1585	NC_008261.1	1830780	1835912	-		protein_coding	CPF_RS07860	CPF_1618	.	WP_011590818. DNA-binding pro	-4.606191	7.386860	-1.717700	6.745751	-2.681604	7.826537	8.570364	6.366790	7.147268	8.570364	6.366790	7.147268	317.150482	113.293049	127.523596	34439	11761	18513
gene1586	NC_008261.1	1836149	1836652	-		protein_coding	CPF_RS07865	CPF_1619	.	WP_003449110. N-acetyltransfe	-1.713430	5.905954	-1.517493	5.823350	-1.129119	6.218943	6.307159	5.530271	6.131961	6.307159	5.530271	6.131961	63.214320	66.516507	61.104421	674	678	871
gene1587	NC_008261.1	1836952	1837740	+		protein_coding	CPF_RS07870	CPF_1620	.	WP_080503687. PHP domain-co	-1.055302	4.237948	1.126341	4.112581	-1.188630	4.150433	4.276954	4.199298	4.027654	4.276954	4.199298	4.027654	13.839522	26.383680	12.951076	231	421	289
gene1588	NC_008261.1	1838091	1839404	+		protein_coding	CPF_RS07875	CPF_1621	.	WP_003460070. sodium-depende	2.816440	8.846328	3.219025	8.740843	-1.142941	8.033916	8.130870	9.624742	7.938118	8.130870	9.624742	7.938118	243.185367	875.425725	239.270470	6760	23264	8892
gene1589	NC_008261.1	1839695	1840390	+		protein_coding	CPF_RS07880	CPF_1622	.	WP_003449164. energy-coupled	-1.082615	9.002915	-1.010459	8.953340	-1.071409	9.010466	9.060357	8.945837	8.960849	9.060357	8.945837	8.960849	458.573867	590.864289	491.249644	6752	8317	9670
gene1590	NC_008261.1	1840499	1841575	+		protein_coding	CPF_RS07885	CPF_1623	.	WP_003460055. aminotransferas	1.056585	3.125209	1.232663	3.010498	-1.166648	2.972494	3.085757	3.165166	2.863388	3.085757	3.165166	2.863388	5.661873	11.615425	5.515420	129	253	168
gene1591	NC_008261.1	1841711	1842757	-		protein_coding	CPF_RS07890	CPF_1624	.	WP_003470420. calcium/proton	1.260848	4.517489	1.155360	4.582425	1.091303	4.415962	4.353385	4.687780	4.479438	4.353385	4.687780	4.479438	14.898871	35.986414	18.438729	330	762	546
gene1592	NC_008261.1	1843198	1844481	-		protein_coding	CPF_RS07895	CPF_1625	.	WP_041702701. alkaline phospha	-1.369126	5.158224	-1.454037	5.199597	1.062018	5.433059	5.389828	4.936572	5.476636	5.389828	4.936572	5.476636	32.065590	43.361435	38.083966	871	1126	1383
gene1593	NC_008261.1	1844550	1844885	-		protein_coding	CPF_RS07900	CPF_1626	.	WP_003460059. hypothetical pro	-1.464611	3.211422	-1.433042	3.196961	-1.022030	3.482702	3.498456	2.947938	3.467019	3.498456	2.947938	3.467019	7.737658	9.859748	8.628983	55	67	82
gene1594	NC_008261.1	1845025	1845438	-		protein_coding	CPF_RS07905	CPF_1627	.	WP_003460053. hypothetical pro	-1.267390	7.097438	-1.476974	7.204398	1.165367	7.379993	7.270427	6.928566	7.491211	7.270427	6.928566	7.491211	127.195269	160.400587	173.287294	1114	1343	2029
gene1595	NC_008261.1	1845638	1846549	-		protein_coding	CPF_RS07910	CPF_1628	.	WP_003460063. DUF368 domain	-1.012050	6.144703	1.065847	6.089894	-1.078690	6.098464	6.153349	6.136068	6.044068	6.153349	6.136068	6.044068	55.200229	99.813488	56.719775	1065	1841	1463
gene1596	NC_008261.1	1846709	1847881	-		protein_coding	CPF_RS07915	CPF_1629	.	WP_078209945. transposase	1.021654	4.378133	1.186693	4.268355	-1.161541	4.253316	4.362707	4.393614	4.146667	4.362707	4.393614	4.146667	14.991015	30.055351	14.438515	372	713	479
gene1598	NC_008261.1	1848575	1849366	-		protein_coding	CPF_RS07930	CPF_1631	.	WP_011590822. D-alanyl-D-alan	2.369212	3.530574	2.272635	3.566169	1.042496	2.992648	2.962777	4.207185	3.022819	2.962777	4.207185	3.022819	5.132860	26.533469	6.294757	86	425	141
gene1599	NC_008261.1	1849636	1849962	-		protein_coding	CPF_RS07935	CPF_1632	.	WP_003470421. hypothetical pro	1.480740	4.078918	2.556086	3.632381	-1.726222	3.388963	3.805576	4.371894	3.017958	3.805576	4.371894	3.017958	9.974415	29.637297	6.271411	69	196	58

gene1601	NC_008261.1	1850448	1851389	+	protein_coding	CPF_RS07945	CPF_1634	.	WP_003472741. hypothetical pro	-1.239712	0.502993	1.026848	0.351702	-1.272995	0.476403	0.681336	0.371332	0.333110	0.681336	0.371332	0.333110	0.451625	0.419923	0.225209
gene1602	NC_008261.1	1851427	1852296	-	protein_coding	CPF_RS07950	CPF_1635	.	WP_003466212. methionyl amino	[illegible]	[illegible]	[illegible]	[illegible]	[illegible]	[illegible]	[illegible]	[illegible]	[illegible]	[illegible]	[illegible]	[illegible]	[illegible]	[illegible]	[illegible]
gene1604	NC_008261.1	1853342	1854265	+	protein_coding	CPF_RS07960	CPF_1637	.	WP_011590823. hypothetical pro	[illegible]	[illegible]	[illegible]	[illegible]	[illegible]	[illegible]	[illegible]	[illegible]	[illegible]	[illegible]	[illegible]	[illegible]	[illegible]	[illegible]	[illegible]
gene1605	NC_008261.1	1854405	1854974	-	protein_coding	CPF_RS07965	CPF_1638	.	WP_011010422. hypothetical pro	[illegible]	[illegible]	[illegible]	[illegible]	[illegible]	[illegible]	[illegible]	[illegible]	[illegible]	[illegible]	[illegible]	[illegible]	[illegible]	[illegible]	[illegible]
gene1608	NC_008261.1	1855590	1856072	-	protein_coding	CPF_RS07975	CPF_1642	.	WP_011590826. hypothetical pro	[illegible]	[illegible]	[illegible]	[illegible]	[illegible]	[illegible]	[illegible]	[illegible]	[illegible]	[illegible]	[illegible]	[illegible]	[illegible]	[illegible]	[illegible]
gene1609	NC_008261.1	1856289	1856882	-	protein_coding	CPF_RS07980	CPF_1643	.	WP_003448636. GTP-binding pro	[illegible]	[illegible]	[illegible]	[illegible]	[illegible]	[illegible]	[illegible]	[illegible]	[illegible]	[illegible]	[illegible]	[illegible]	[illegible]	[illegible]	[illegible]
gene1610	NC_008261.1	1856882	1859212	-	protein_coding	CPF_RS07985	CPF_1644	.	WP_003460768. endopeptidase L	[illegible]	[illegible]	[illegible]	[illegible]	[illegible]	[illegible]	[illegible]	[illegible]	[illegible]	[illegible]	[illegible]	[illegible]	[illegible]	[illegible]	[illegible]
gene1611	NC_008261.1	1859334	1861046	-	protein_coding	CPF_RS07990	CPF_1645	.	WP_003460767. ATP-dependent	[illegible]	[illegible]	[illegible]	[illegible]	[illegible]	[illegible]	[illegible]	[illegible]	[illegible]	[illegible]	[illegible]	[illegible]	[illegible]	[illegible]	[illegible]
gene1612	NC_008261.1	1861157	1862464	-	protein_coding	CPF_RS07995	CPF_1646	.	WP_003448621. ATP-dependent	[illegible]	[illegible]	[illegible]	[illegible]	[illegible]	[illegible]	[illegible]	[illegible]	[illegible]	[illegible]	[illegible]	[illegible]	[illegible]	[illegible]	[illegible]
gene1613	NC_008261.1	1862504	1863088	-	protein_coding	CPF_RS08000	CPF_1647	.	WP_003448628. ATP-dependent	[illegible]	[illegible]	[illegible]	[illegible]	[illegible]	[illegible]	[illegible]	[illegible]	[illegible]	[illegible]	[illegible]	[illegible]	[illegible]	[illegible]	[illegible]
gene1614	NC_008261.1	1863279	1864565	-	protein_coding	CPF_RS08005	CPF_1648	.	WP_003460760. trigger factor	[illegible]	[illegible]	[illegible]	[illegible]	[illegible]	[illegible]	[illegible]	[illegible]	[illegible]	[illegible]	[illegible]	[illegible]	[illegible]	[illegible]	[illegible]
gene1615	NC_008261.1	1864692	1865480	-	protein_coding	CPF_RS08010	CPF_1649	.	WP_011590827. sugar phosphate	[illegible]	[illegible]	[illegible]	[illegible]	[illegible]	[illegible]	[illegible]	[illegible]	[illegible]	[illegible]	[illegible]	[illegible]	[illegible]	[illegible]	[illegible]
gene1616	NC_008261.1	1865685	1867271	+	protein_coding	CPF_RS08015	CPF_1650	.	WP_003481525. ATP-dependent	[illegible]	[illegible]	[illegible]	[illegible]	[illegible]	[illegible]	[illegible]	[illegible]	[illegible]	[illegible]	[illegible]	[illegible]	[illegible]	[illegible]	[illegible]
gene1617	NC_008261.1	1867310	1867573	-	protein_coding	CPF_RS08020	CPF_1651	.	WP_003454590. hypothetical pro	[illegible]	[illegible]	[illegible]	[illegible]	[illegible]	[illegible]	[illegible]	[illegible]	[illegible]	[illegible]	[illegible]	[illegible]	[illegible]	[illegible]	[illegible]
gene1618	NC_008261.1	1867767	1868474	+	protein_coding	CPF_RS08025	CPF_1652	.	WP_003470332. purine-nucleosi	[illegible]	[illegible]	[illegible]	[illegible]	[illegible]	[illegible]	[illegible]	[illegible]	[illegible]	[illegible]	[illegible]	[illegible]	[illegible]	[illegible]	[illegible]
gene1619	NC_008261.1	1868529	1869803	-	protein_coding	CPF_RS08030	CPF_1653	.	WP_003457229. PEGA domain-c	[illegible]	[illegible]	[illegible]	[illegible]	[illegible]	[illegible]	[illegible]	[illegible]	[illegible]	[illegible]	[illegible]	[illegible]	[illegible]	[illegible]	[illegible]
gene1620	NC_008261.1	1869814	1870158	-	protein_coding	CPF_RS08035	CPF_1654	.	WP_003457196. hypothetical pro	[illegible]	[illegible]	[illegible]	[illegible]	[illegible]	[illegible]	[illegible]	[illegible]	[illegible]	[illegible]	[illegible]	[illegible]	[illegible]	[illegible]	[illegible]
gene1621	NC_008261.1	1870342	1871250	-	protein_coding	CPF_RS08040	CPF_1655	.	WP_003457170. DNA polymerase	[illegible]	[illegible]	[illegible]	[illegible]	[illegible]	[illegible]	[illegible]	[illegible]	[illegible]	[illegible]	[illegible]	[illegible]	[illegible]	[illegible]	[illegible]
gene1622	NC_008261.1	1871497	1874418	-	protein_coding	CPF_RS08045	CPF_1656	.	WP_011590829. peptidase M16	[illegible]	[illegible]	[illegible]	[illegible]	[illegible]	[illegible]	[illegible]	[illegible]	[illegible]	[illegible]	[illegible]	[illegible]	[illegible]	[illegible]	[illegible]
gene1623	NC_008261.1	1874793	1875062	-	protein_coding	CPF_RS08050	CPF_1657	.	WP_003454555. hypothetical pro	[illegible]	[illegible]	[illegible]	[illegible]	[illegible]	[illegible]	[illegible]	[illegible]	[illegible]	[illegible]	[illegible]	[illegible]	[illegible]	[illegible]	[illegible]
gene1624	NC_008261.1	1875232	1876383	+	protein_coding	CPF_RS08055	CPF_1658	.	WP_003457302. hypothetical pro	[illegible]	[illegible]	[illegible]	[illegible]	[illegible]	[illegible]	[illegible]	[illegible]	[illegible]	[illegible]	[illegible]	[illegible]	[illegible]	[illegible]	[illegible]
gene1626	NC_008261.1	1877319	1877897	+	protein_coding	CPF_RS08065	CPF_1661	.	WP_003457263. flavin reductase	[illegible]	[illegible]	[illegible]	[illegible]	[illegible]	[illegible]	[illegible]	[illegible]	[illegible]	[illegible]	[illegible]	[illegible]	[illegible]	[illegible]	[illegible]
gene1627	NC_008261.1	1878024	1879595	-	protein_coding	CPF_RS08070	CPF_1662	.	WP_003475779. ClC family H(+)/(	[illegible]	[illegible]	[illegible]	[illegible]	[illegible]	[illegible]	[illegible]	[illegible]	[illegible]	[illegible]	[illegible]	[illegible]	[illegible]	[illegible]	[illegible]
gene1628	NC_008261.1	1879858	1881168	+	protein_coding	CPF_RS08075	CPF_1663	.	WP_011590830. electron transpo	[illegible]	[illegible]	[illegible]	[illegible]	[illegible]	[illegible]	[illegible]	[illegible]	[illegible]	[illegible]	[illegible]	[illegible]	[illegible]	[illegible]	[illegible]
gene1629	NC_008261.1	1881182	1882162	+	protein_coding	CPF_RS08080	CPF_1664	.	WP_003478008. NADH:ubiquinon	[illegible]	[illegible]	[illegible]	[illegible]	[illegible]	[illegible]	[illegible]	[illegible]	[illegible]	[illegible]	[illegible]	[illegible]	[illegible]	[illegible]	[illegible]
gene1630	NC_008261.1	1882473	1882730	-	protein_coding	CPF_RS08085	CPF_1665	.	WP_003475769. DUF1653 domai	[illegible]	[illegible]	[illegible]	[illegible]	[illegible]	[illegible]	[illegible]	[illegible]	[illegible]	[illegible]	[illegible]	[illegible]	[illegible]	[illegible]	[illegible]
gene1631	NC_008261.1	1882859	1884016	-	protein_coding	CPF_RS08090	CPF_1666	.	WP_004459380. tRNA 4-thiourid	[illegible]	[illegible]	[illegible]	[illegible]	[illegible]	[illegible]	[illegible]	[illegible]	[illegible]	[illegible]	[illegible]	[illegible]	[illegible]	[illegible]	[illegible]
gene1632	NC_008261.1	1884013	1885158	-	protein_coding	CPF_RS08095	CPF_1667	.	WP_011590831. cysteine desulfu	[illegible]	[illegible]	[illegible]	[illegible]	[illegible]	[illegible]	[illegible]	[illegible]	[illegible]	[illegible]	[illegible]	[illegible]	[illegible]	[illegible]	[illegible]
gene1633	NC_008261.1	1885332	1885769	-	protein_coding	CPF_RS08100	CPF_1668	.	WP_003454663. hypothetical pro	[illegible]	[illegible]	[illegible]	[illegible]	[illegible]	[illegible]	[illegible]	[illegible]	[illegible]	[illegible]	[illegible]	[illegible]	[illegible]	[illegible]	[illegible]
gene1635	NC_008261.1	1888964	1889803	-	protein_coding	CPF_RS08110	CPF_1670	.	WP_011590832. homocysteine S	[illegible]	[illegible]	[illegible]	[illegible]	[illegible]	[illegible]	[illegible]	[illegible]	[illegible]	[illegible]	[illegible]	[illegible]	[illegible]	[illegible]	[illegible]
gene1636	NC_008261.1	1890045	1890773	+	protein_coding	CPF_RS08115	CPF_1671	.	WP_011590833. 1-acyl-sn-glyce	[illegible]	[illegible]	[illegible]	[illegible]	[illegible]	[illegible]	[illegible]	[illegible]	[illegible]	[illegible]	[illegible]	[illegible]	[illegible]	[illegible]	[illegible]
gene1637	NC_008261.1	1890817	1892205	-	protein_coding	CPF_RS08120	CPF_1672	.	WP_011590834. sodium:alanine s	[illegible]	[illegible]	[illegible]	[illegible]	[illegible]	[illegible]	[illegible]	[illegible]	[illegible]	[illegible]	[illegible]	[illegible]	[illegible]	[illegible]	[illegible]
gene1638	NC_008261.1	1892512	1893168	-	protein_coding	CPF_RS08125	CPF_1673	.	WP_011590835. HDIG domain-co	[illegible]	[illegible]	[illegible]	[illegible]	[illegible]	[illegible]	[illegible]	[illegible]	[illegible]	[illegible]	[illegible]	[illegible]	[illegible]	[illegible]	[illegible]
[illegible]	[illegible]	[illegible]	[illegible]	[illegible]	[illegible]	[illegible]	[illegible]	[illegible]	[illegible]	[illegible]	[illegible]	[illegible]	[illegible]	[illegible]	[illegible]	[illegible]	[illegible]	[illegible]	[illegible]	[illegible]	[illegible]	[illegible]	[illegible]	[illegible]

gene1867	NC_008261.1	2142083	2142553	+	protein_coding	CPF_RS09265	CPF_1906	.	WP_003449697. transcriptional r	-1.020691	9.441957	-1.100854	9.496255	1.078539	9.511125	9.456742	9.427196	9.565820	9.456742	9.427196	9.565820	623.543210	784.205665	758.803526	6213	7470	10108
gene1868	NC_008261.1	2142660	2142863	+	protein_coding	CPF_RS09270	CPF_1907	.	WP_003459798. DUF378 domain	1.566577	3.081662	1.975339	2.889963	-1.260927	2.602208	2.774819	3.422435	2.440335	2.774819	3.422435	2.440335	4.402603	14.542912	3.986417	19	60	23
gene1869	NC_008261.1	2142911	2144656	-	protein_coding	CPF_RS09275	CPF_1908	.	WP_025648231. phosphoenolpyr	-1.152470	7.583961	-1.454266	7.747724	1.261869	7.853005	7.687017	7.482287	8.022578	7.687017	7.482287	8.022578	169.587414	227.349187	249.833309	6264	8028	12337
gene1870	NC_008261.1	2144855	2145709	+	protein_coding	CPF_RS09280	CPF_1910	.	WP_011590930. MBL fold metallo	1.085506	0.425210	1.084599	0.425902	1.000837	0.370728	0.370125	0.488493	0.371332	0.370125	0.488493	0.371332	0.221146	0.578315	0.248125	4	10	6
gene1871	NC_008261.1	2145765	2146448	-	protein_coding	CPF_RS09285	CPF_1911	.	WP_003449676. hypothetical pro	2.076274	5.668434	1.897252	5.739361	1.094359	5.230519	5.165880	6.219878	5.295966	5.165880	6.219878	5.295966	27.159549	104.674945	34.013773	393	1448	658
gene1872	NC_008261.1	2146634	2146852	+	protein_coding	CPF_RS09290	CPF_1912	.	WP_003449499. iron transporter	1.581379	7.271230	1.570381	7.276496	1.007004	6.953182	6.948149	7.609333	6.958219	6.948149	7.609333	6.958219	100.152080	247.003716	113.822997	464	1094	705
gene1873	NC_008261.1	2146890	2148653	+	protein_coding	CPF_RS09295	CPF_1913	.	WP_011590932. ferrous iron trar	1.455569	7.246698	1.706733	7.126504	-1.172553	6.865177	6.980964	7.522547	6.751310	6.980964	7.522547	6.751310	102.686423	232.597547	96.812975	3832	8298	4830
gene1874	NC_008261.1	2148668	2148817	+	protein_coding	CPF_RS14805	CPF_1914	.	WP_003459783. FeoB-associate	-1.687639	2.149250	1.484762	1.492544	-2.505741	1.777549	2.559654	1.804648	1.234417	2.559654	1.804648	1.234417	3.781604	3.626033	1.178593	12	11	5
gene1875	NC_008261.1	2148863	2150560	-	protein_coding	CPF_RS09300	CPF_1915	.	WP_011590933. arginine--tRNA	-2.892994	9.814962	-1.378692	9.307315	-2.098362	10.062286	10.611111	9.078547	9.541847	10.611111	9.078547	9.541847	1334.836201	631.585460	739.723115	47949	21689	35524
gene1876	NC_008261.1	2151042	2152550	+	protein_coding	CPF_RS09305	CPF_1916	.	WP_003478235. Ppx/GppA family	1.110884	5.396617	1.047758	5.439246	1.060248	5.363331	5.321296	5.473003	5.405698	5.321296	5.473003	5.405698	30.260353	63.372014	35.966995	966	1934	1535
gene1877	NC_008261.1	2152648	2153862	-	protein_coding	CPF_RS09310	CPF_1917	.	WP_011590934. hypothetical pro	-1.524435	2.095670	-1.372895	2.029261	-1.110380	2.345018	2.421761	1.813487	2.270708	2.421761	1.813487	2.270708	3.306959	3.662659	3.492127	85	90	120
gene1878	NC_008261.1	2154028	2154648	-	protein_coding	CPF_RS09315	CPF_1918	.	WP_003459807. site-2 protease	-1.935449	6.044936	-1.223413	5.730951	-1.582008	6.200308	6.540009	5.587340	5.878252	6.540009	5.587340	5.878252	72.389408	69.351656	50.673794	951	871	890
gene1879	NC_008261.1	2154852	2156231	-	protein_coding	CPF_RS09320	CPF_1919	.	WP_003459766. PhoH family prot	-12.773512	3.422571	-11.454382	3.375214	-1.115164	5.643022	5.722197	2.047114	5.564942	5.722197	2.047114	5.564942	40.248054	4.478795	39.764694	1175	125	1552
gene1880	NC_008261.1	2156453	2157067	+	protein_coding	CPF_RS09325	CPF_1920	.	WP_003459802. 3'-5' exonuclea	1.143644	5.586213	1.445747	5.411434	-1.264158	5.318457	5.490232	5.683871	5.152055	5.490232	5.683871	5.152055	34.434121	74.128649	30.413442	448	922	529
gene1881	NC_008261.1	2157104	2157433	-	protein_coding	CPF_RS09330	CPF_1921	.	WP_003459777. single-stranded	1.257386	1.476239	1.100413	1.580139	1.142648	1.413162	1.320241	1.650668	1.512623	1.320241	1.650668	1.512623	1.145941	3.146557	1.607172	8	21	15
gene1882	NC_008261.1	2157713	2159143	-	protein_coding	CPF_RS09335	CPF_1922	.	WP_003459773. adenylosuccinat	7.863440	7.893720	7.690880	7.913001	1.022437	6.561072	6.545085	9.520246	6.577097	6.545085	9.520246	6.577097	72.540210	820.366557	84.058123	2196	23742	3402
gene1883	NC_008261.1	2159386	2159646	-	protein_coding	CPF_RS09340	CPF_1923	.	WP_003459789. HPr family phosp	1.018872	11.035512	1.254705	10.884102	-1.231464	10.870809	11.022033	11.049007	10.721659	11.022033	11.049007	10.721659	1684.697518	2645.639788	1698.528242	9302	13965	12538
gene1884	NC_008261.1	2159741	2160934	-	protein_coding	CPF_RS09345	CPF_1924	.	WP_003459805. pyridoxal phosph	1.202849	8.017388	1.201837	8.018005	1.000842	7.885874	7.885267	8.151723	7.886481	7.885267	8.151723	7.886481	197.591997	333.035111	231.513518	4991	8042	7818
gene1885	NC_008261.1	2161087	2161347	-	protein_coding	CPF_RS09350	CPF_1925	.	WP_003459803. stage V sporulat	-3.873400	10.378857	-4.569999	10.486891	1.179842	11.520200	11.401521	9.447921	11.640115	11.401521	9.447921	11.640115	2297.759343	788.292671	3059.328704	12687	4161	22583
gene1886	NC_008261.1	2161565	2163100	-	protein_coding	CPF_RS09355	CPF_1926	.	WP_003459753. ribonuclease Y	-1.595676	11.408510	-1.459677	11.345950	-1.093171	11.686137	11.750573	11.076405	11.622054	11.750573	11.076405	11.622054	3013.065938	2649.926786	3025.829672	97907	82318	131447
gene1887	NC_008261.1	2163448	2164506	-	protein_coding	CPF_RS09360	CPF_1927	.	WP_003459804. DNA recombinat	-1.225770	9.616838	1.100839	9.401558	-1.349375	9.546211	9.764803	9.471115	9.332512	9.764803	9.471115	9.332512	766.364094	799.118572	646.623385	17169	17115	19367
gene1888	NC_008261.1	2164642	2165232	-	protein_coding	CPF_RS09365	CPF_1928	.	WP_003469140. CDP-diacylglyce	1.356964	7.887509	1.736082	7.702584	-1.279387	7.490556	7.670391	8.110773	7.314938	7.670391	8.110773	7.314938	166.125050	325.121430	147.354005	2077	3886	2463
gene1889	NC_008261.1	2165216	2166553	-	protein_coding	CPF_RS09370	CPF_1929	.	WP_003459746. 30S ribosomal p	1.423946	7.871795	1.815595	7.688621	-1.275045	7.443638	7.620975	8.130870	7.270427	7.620975	8.130870	7.270427	159.792570	329.565419	140.374094	4523	8918	5312
gene1890	NC_008261.1	2166622	2169012	-	protein_coding	CPF_RS09375	CPF_1930	.	WP_003459775. DNA translocase	2.201686	4.038409	1.834895	4.186964	1.199898	3.638117	3.509036	4.647645	3.771947	3.509036	4.647645	3.771947	7.789378	35.031934	10.883868	394	1694	736
gene1891	NC_008261.1	2169144	2169833	-	protein_coding	CPF_RS09380	CPF_1931	.	WP_003459750. Clp protease	2.052951	7.485769	1.917394	7.538394	1.070699	7.033983	6.984879	8.022578	7.083432	6.984879	8.022578	7.083432	102.898004	306.994540	123.957210	1502	4284	2419
gene1892	NC_008261.1	2169887	2171083	-	protein_coding	CPF_RS09385	CPF_1932	.	WP_003459758. aspartate kinase	1.516279	6.454258	1.461672	6.481916	1.037359	6.187372	6.160971	6.761507	6.213887	6.160971	6.761507	6.213887	55.878971	146.767987	64.335212	1415	3553	2178
gene1893	NC_008261.1	2171097	2171372	-	protein_coding	CPF_RS09390	CPF_1933	.	WP_003459765. YlmC/YmxH fam	1.778535	5.546549	1.912321	5.489878	-1.075223	5.094148	5.146734	5.977424	5.042099	5.146734	5.977424	5.042099	26.717857	91.367423	28.055631	156	510	219
gene1894	NC_008261.1	2171507	2173615	-	protein_coding	CPF_RS09395	CPF_1934	.	WP_003459742. polyribonucleoti	2.095388	9.605042	2.300083	9.533704	-1.097689	9.018760	9.086245	10.153462	8.951776	9.086245	10.153462	8.951776	466.738562	1216.922431	485.888686	20824	51905	28982
gene1895	NC_008261.1	2173707	2173970	-	protein_coding	CPF_RS09400	CPF_1935	.	WP_003449423. 30S ribosomal p	1.437091	12.417811	1.221230	12.537146	1.176757	12.275837	12.158991	12.682142	12.393807	12.158991	12.682142	12.393807	4227.267916	6862.116897	5089.913441	23609	36638	38004
gene1896	NC_008261.1	2174086	2175015	-	protein_coding	CPF_RS09405	CPF_1936	.	WP_003459763. bifunctional ribo	1.287375	5.531565	1.261504	5.546678	1.020508	5.366973	5.352349	5.716781	5.381636	5.352349	5.716781	5.381636	31.055918	75.976549	35.471839	611	1429	933
gene1897	NC_008261.1	2175019	2175903	-	protein_coding	CPF_RS09410	CPF_1937	.	WP_003459755. tRNA pseudouri	1.270991	5.656592	1.225922	5.683381	1.036764	5.512241	5.486259	5.832213	5.538346	5.486259	5.832213	5.538346	34.290820	83.192027	39.193202	642	1489	981
gene1898	NC_008261.1	2175907	2176860	-	protein_coding	CPF_RS09415	CPF_1938	.	WP_003459795. bifunctional olig	1.084868	6.094306	1.208989	6.014890	-1.114411	5.957176	6.035829	6.153349	5.879547	6.035829	6.153349	5.879547	50.342113	100.965002	50.701725	1016	1948	1368
gene1899	NC_008261.1	2176864	2177214	-	protein_coding	CPF_RS09420	CPF_1939	.	WP_003449473. ribosome-bindin	1.205527	8.183563	1.373362	8.087412	-1.139221	7.955262	8.049841	8.319506	7.861794	8.049841	8.319506	7.861794	230.290012	367.674636	227.659793	1710	2610	2260
gene1900	NC_008261.1	2177236	2179275	-	protein_coding	CPF_RS09425	CPF_1940	.	WP_003449445. translation initia	1.106672	9.153289	1.319108	9.024682	-1.191959	8.952883	9.080447	9.226674	8.827112	9.080447	9.226674	8.827112	462.551392	687.152572	442.318920	19962	28350	25520
gene1901	NC_008261.1	2179306	2179608	-	protein_coding	CPF_RS09430	CPF_1941	.	WP_003459801. 50S ribosomal p	1.762856	7.238591	1.703444	7.264711	1.034877	6.865863	6.841177	7.659092	6.890637	6.841177	7.659092	6.890637	91.887995	254.246572	107.240270	589	1558	919
gene1902	NC_008261.1	2179601	2179867	-	protein_coding	CPF_RS09435	CPF_1942	.	WP_003459790. DUF448 domain	-1.245532	6.230753	-1.085063	6.132991	-1.147889	6.290868	6.391147	6.074385	6.192164	6.391147	6.074385	6.192164	66.921651	97.039892	63.299700	378	524	478
gene1903	NC_008261.1	2179895	2180995	-	protein_coding	CPF_RS09440	CPF_1943	nusA	WP_003449452. transcription ter	1.287960	8.446777	1.350635	8.411681	-1.048662	8.231859	8.266206	8.631294	8.197655	8.266206	8.631294	8.197655	265.759892	477.887207	290.055847	6190	10641	9032
gene1904	NC_008261.1	2181015	2181479	-	protein_coding	CPF_RS09445	CPF_1944	.	WP_003459796. ribosome matura	1.693580	7.121215	1.428125	7.249770	1.185877	6.873188	6.751310	7.511387	6.997265	6.751310	7.511387	6.997265	86.204315	230.960201	117.174929	848	2172	1541
gene1905	NC_008261.1	2181712	2186061	-	protein_coding	CPF_RS09450	CPF_1945	polC	WP_003459779. PolC-type DNA	1.360269	5.666887	1.542244	5.571905	-1.133779	5.357951	5.449286	5.893178	5.268147	5.449286	5.893178	5.268147	33.556306	87.024783	33.203802	3088	7656	4085
gene1906	NC_008261.1	2186139	2187188	-	protein_coding	CPF_RS09455	CPF_1946	.	WP_003459806. flavodoxin-depe	1.368378	7.816951	1.449013	7.774320	-1.058927	7.552576	7.593990	8.046457	7.511387	7.593990	8.046457	7.511387	156.396354	312.215536	175.004184	3474	6630	5197
gene1907	NC_008261.1	2187208	2188215	-	protein_coding	CPF_RS09460	CPF_1947	.	WP_003459800. RIP metalloprote	1.023153	7.818851	1.129972	7.746734	-1.104402	7.730393	7.802357	7.835379	7.659092	7.802357	7.835379	7.659092	184.343836	276.171045	193.976721	3931	5630	5530
gene1908	NC_008261.1	2188227	2189381	-	protein_coding	CPF_RS09465	CPF_1948	.	WP_003459741. 1-deoxy-D-xylu	1.072421	7.340897	1.119769	7.309450	-1.044150	7.259403	7.290634	7.391506	7.228305	7.290634	7.391506	7.228305	128.058876	215.207391	136.349404	3129	5027	4454
gene1909	NC_008261.1	2189741	2190535	-	protein_coding	CPF_RS09470	CPF_1949	.	WP_003459799. CDP-archaeol s	2.522688	5.363482	1.981843	5.557053	1.272900	4.908349	4.737375	6.072337	5.085494	4.737375	6.072337	5.085494	20.156665	96.652739	28.997829	339	1554	652
gene1910	NC_008261.1	2190553	2191314	-	protein_coding	CPF_RS09475	CPF_1950	.	WP_003459793. isoprenyl transfe	2.355661	7.554348	2.064291	7.657004	1.141148	7.056124	6.961523	8.197655	7.152009	6.961523	8.197655	7.152009	101.115735	344.044827	127.742748	1630	5302	2753
gene1911	NC_008261.1	2191379	2191936	-	protein_coding	CPF_RS09480	CPF_1951	.	WP_003459781. ribosome-recyc	3.290552	8.262472	3.435938	8.227801	-1.044183	7.416604	7.447857	9.166187	7.385483	7.447857	9.166187	7.385483	139.692331	662.911780	158.349637	1649	7481	2499
gene1912	NC_008261.1	2191950	2192663	-	protein_coding	CPF_RS09485	CPF_1952	.	WP_003459788. UMP kinase	3.206230	8.521944	3.245782	8.512180	-1.012336	7.713998	7.722847	9.403725	7.705159	7.722847	9.403725	7.705159	172.727694	773.405888	202.737759	2609	11168	4094
gene1913	NC_008261.1	2192756	2193667	-	protein_coding	CPF_RS09490	CPF_1953	.	WP_003459744. elongation facto	1.745945	10.967040	1.656374	11.006377	1.054076	10.610323	10.572402	11.376410	10.648381	10.572402	11.376410	10.648381	1314.853900	3252.423308	1662.629915	25368	59989	42885
gene1914	NC_008261.1	2193747	2194448	-	protein_coding	CPF_RS09495	CPF_1954	.	WP_003470452. 30S ribosomal p	2.131111	11.641916	1.844742	11.750498	1.155235	11.212511	11.108901	12.200506	11.317087	11.108901	12.200506	11.317087	1779.024009	5519.979611	2511.611338	26420	78369	49866
gene1915	NC_008261.1	2194831	2195607	-	protein_coding	CPF_RS09500	CPF_1955	.	WP_003449426. GTP-sensing ple	-1.499646	9.114737	1.206852	8.690430	-1.809850	8.973601	9.411734	8.827112	8.555864	9.411734	8.827112	8.555864	587.438419	542.186696	370.050815	9656	8520	8132
gene1916	NC_008261.1	2195749	2197851	-	protein_coding	CPF_RS09505	CPF_1956	.	WP_003458449. type I DNA topo	1.143702	6.451766	1.405556	6.298993	-1.228953	6.205141	6.355637	6.549348	6.058207	6.355637	6.549348	6.058207	65.476781	131.079833	57.416465	2913	5575	3415
gene1917	NC_008261.1	2197896	2198978	-	protein_coding	CPF_RS09510	CPF_1957	.	WP_003471623. DNA-protecting	1.969487	4.350672	2.583641	4.125835	-1.311834	3.688160	3.889146	4.866966	3.497561	3.889146	4.866966	3.497561	10.475358	41.410370	8.880256	240	907	272
gene1918	NC_008261.1	2198979	2200502	-	protein_coding	CPF_RS09515	CPF_1958	.	WP_003478266. ATP-binding pro	1.034393	0.727598	1.008740	0.746096	1.025431	0.721502	0.703615	0.752399	0.739844	0.703615	0.752399	0.739844	0.465256	1.038234	0.556815	15	32	24
gene1920	NC_008261.1	2201001	2201819	-	protein_coding	CPF_RS09525	CPF_1960	.	WP_003478270. ribonuclease HII	-1.281558	5.484874	1.019342	5.295006	-1.306346	5.470578	5.666742	5.308843	5.281205	5.666742	5.308843	5.281205	38.901120	56.328479	33.630906	674	933	779
gene1921	NC_008261.1	2201835	2202680	-	protein_coding	CPF_RS09530	CPF_1961	.	WP_003458489. ribosome biogen	-1.804315	6.218860	-1.271952	5.978694	-1.418540	6.401971	6.659140	5.807689	6.154733	6.659140	5.807689	6.154733	79.845046	81.650025	61.980604	1429	1397	1483
gene1922	NC_008261.1	2202827	2203174	-	protein_coding	CPF_RS09535	CPF_1962	.	WP_003449446. 50S ribosomal p	2.322746	11.361778	2.394711	11.338535	-1.030982	10.748082	10.770114	11.985946	10.726094	10.770114	11.985946	10.726094	1473.929217	4782.043646	1701.034445	10851	33656	16742
gene1923	NC_008261.1	2203295	2204005	-	protein_coding	CPF_RS09540	CPF_1963	.	WP_003458426. tRNA (guanosine	1.892062	5.730439	2.269444	5.586489	-1.199455	5.156033	5.288891	6.208850	5.026512	5.288891	6.208850	5.026512	29.385885	103.759890	27.898334	442	1492	561
gene1924	NC_008261.1	2203995	2204492	-	protein_coding	CPF_RS09545	CPF_1964	.	WP_003458412. ribosome matura	2.409916	5.842922	2.613641	5.777313	-1.084536	5.183910	5.242779	6.511762	5.125701	5.242779	6.511762	5.125701	28.475937	125.997683	29.961815	300	1269	422
gene1925	NC_008261.1	2204548	2204775	-	protein_coding	CPF_RS09550	CPF_1965	.	WP_003458429. KH domain-con	1.960735	11.262674	1.994757	11.249711	-1.017351	10.775028	10.787444	11.758839	10.762626	10.787444	11.758839	10.762626	1490.043358	4059.985447	1714.077039	7187	18721	11053
gene1926	NC_008261.1	2204819	2205064	-	protein_coding	CPF_RS09555	CPF_1966	.	WP_003458442. 30S ribosomal p	2.179793	11.818352	2.263567	11.789790	-1.038432	11.242380	11.269616	12.393807	11.215209	11.269616	12.393807	11.215209	2000.138224	6015.917756	2414.965259	10409	29930	16802
gene1927	NC_008261.1	2205098	2206456	-	protein_coding	CPF_RS09560	CPF_1967	.	WP_003458480. signal recognitio	-1.066141	8.407551	1.054099	8.323386	-1.123818	8.369249	8.453877	8.361479	8.285468	8.453877	8.361479	8.285468	295.655238	380.576974	315.123956	8500	10460	12112
gene1928	NC_008261.1	2206468	2206806	-	protein_coding	CPF_RS09565	CPF_1968	.	WP_003458448. putative DNA-bi	1.391504	6.415040	1.375986	6.423430	1.011278	6.189227	6.181143	6.657788	6.197322	6.181143	6.657788	6.197322	56.751954	138.565205	63.831748	407	950	612
gene1929	NC_008261.1	2206981	2207895	-	protein_coding	CPF_RS09570	CPF_1969	.	WP_003466435. signal recognitio	1.053400	7.300497	-1.015454	7.349174	1.069679	7.311494	7.263067	7.338120	7.360244	7.263067	7.338120	7.360244	127.086705	209.347994	153.758049	2460	3874	3979
gene1930	NC_008261.1	2207909	2211466	-	protein_coding	CPF_RS09575	CPF_1970	.	WP_011590936. chromosome se	-1.039530	5.706319	1.058121	5.637522	-1.099949	5.665219	5.734353	5.678422	5.596917	5.734353	5.678422	5.596917	40.587414	74.002084	40.535243	3055	5325	4079
gene1931	NC_008261.1	2211671	2212744	-	protein_coding	CPF_RS09580	CPF_1971	.	WP_003458439. radical SAM prot	-1.176435	8.262316	-1.191384	8.271291	1.012707	8.389461	8.380358	8.145936	8.398575	8.380358	8.145936	8.398575	283.752370	332.217514	339.586147	6447	7216	10315
gene1932	NC_008261.1	2212723	2213448	-	protein_coding	CPF_RS09585	CPF_1972	.	WP_011590937. ribonuclease III	-1.493112	7.162505	-1.641269	7.227753	1.099227	7.525436	7.457501	6.879178	7.593990	7.457501	6.879178	7.593990	141.354393	157.804613	185.311792	2171	2317	3805
gene1933	NC_008261.1	2213535	2213765	-	protein_coding	CPF_RS09590	CPF_1973	.	WP_003458423. acyl carrier prot	2.060618	12.396998	1.872963	12.468627	1.100192	11.955104	11.886425	12.929502	12.024180	11.886425	12.929502	12.024180	3354.332218	9058.231901	4029.868768	16392	42318	26328
gene1934	NC_008261.1	2213842	2214861	-	protein_coding	CPF_RS09595	CPF_1974	.	WP_003458435. phosphate acylt	3.929907	7.486616	3.370720	7.611847	1.165896	6.673982	6.564181	8.538677	6.785620	6.564181	8.538677	6.785620	74.056420	445.497858	99.591086	1598	9190	2873
gene1935	NC_008261.1	2214919	2215101	-	protein_coding	CPF_RS09600	CPF_1975	.	WP_003449428. 50S ribosomal p	2.036502	10.290811	1.862612	10.358262	1.093358	9.854718	9.790545	10.816638	9.919311	9.790545	10.816638	9.919311	781.376593	2208.313309	1019.192898	3025	8173	5275
gene1936	NC_008261.1	2215117	2215611	-	protein_coding	CPF_RS09605	CPF_1976	.	WP_003449453. hypothetical pro	3.087174	9.844417	2.484845	10.013004	1.242401	9.220036	9.064799	10.691086	9.377930	9.064799	10.691086	9.377930	459.044757	1935.781987	664.012004	4807	19379	9296
gene1937	NC_008261.1	2215731	2216927	-	protein_coding	CPF_RS09610	CPF_1977	.	WP_003458524. acetate kinase	2.486435	9.151567	1.848461	9.378535	1.345138	8.729341	8.518084	9.832162	8.945837	8.518084	9.832162	8.945837	308.301854	978.219167	483.282099	7807	23681	16361
gene1938	NC_008261.1	2217018	2218019	-	protein_coding	CPF_RS09615	CPF_1978	.	WP_003458455. phosphate acety	2.206607	9.030255	1.871309	9.156024	1.179178	8.595438	8.477369	9.619199	8.715151	8.477369	9.619199	8.715151	298.103271	872.458584	409.790342	6319	17680	11613
gene1939	NC_008261.1	2218254	2219462	+	protein_coding	CPF_RS09620	CPF_1979	.	WP_011590938. nucleotidyltrans	-1.249341	3.542473	-1.271915	3.554796	1.018068	3.719589	3.706695	3.385527	3.732529	3.706695	3.385527	3.732529	9.149043	14.150770	10.528372	234	346	360
gene1940	NC_008261.1	2219527	2220687	+	protein_coding	CPF_RS09625	CPF_1981	.	WP_011590939. sporulation inte	-1.424776	1.030863	-1.555157	1.079157	1.091510	1.379107	1.317390	0.806655	1.443715	1.317390	0.806655	1.443715	1.140019	1.107316	1.492275	28	26	49
gene1941	NC_008261.1	2220670	2221200	-	protein_coding	CPF_RS09630	CPF_1980	.	WP_003458392. ATPase	-1.206047	7.558684	-1.181117	7.543869	-1.021107	7.679953	7.695035	7.424749	7.664901	7.695035	7.424749	7.664901	170.118786	220.038908	195.100379	1911	2363	2930
gene1942	NC_008261.1	2221209	2221703	-	protein_coding	CPF_RS09635	CPF_1982	.	WP_011590940. phosphopanteth	-1.386747	7.180129	-1.292466	7.130811	-1.072947	7.368889	7.419854	6.948149	7.318275	7.419854	6.948149	7.318275	138.085858	162.921741	148.145535	1446	1631	2074
gene1943	NC_008261.1	2221705	2222262	-	protein_coding	CPF_RS09640	CPF_1983	.	WP_003458525. 16S rRNA (guan	-1.293935	7.756944	-1.035880	7.598674	-1.249117	7.782945	7.945053	7.573288	7.624145	7.945053	7.573288	7.624145	209.157287	239.963254	189.398585	2469	2708	2989
gene1944	NC_008261.1	2222327	2224399	-	protein_coding	CPF_RS09645	CPF_1984	.	WP_011590941. DNA helicase R	1.023069	4.609605	1.361304	4.397967	-1.330608	4.382299	4.593183	4.626086	4.181097	4.593183	4.626086	4.181097	18.196577	34.824415	14.702561	798	1460	862
gene1945	NC_008261.1	2224553	2226199	-	protein_coding	CPF_RS09650	CPF_1985	.	WP_003471654. DAK2 domain-c	-1.467497	10.027273	-1.082768	9.811605	-1.355320	10.086067	10.307768	9.754411	9.869135	10.307768	9.754411	9.869135	1074.410753	959.346517	986.475678	37435	31955	45951
gene1946	NC_008261.1	2226213	2226560	-	protein_coding	CPF_RS09655	CPF_1986	.	WP_003458425. Asp23/Gls24 fa	-1.280134	9.117719	1.228424	8.791655	-1.572548	8.965109	9.297607	8.941312	8.644503	9.297607	8.941312	8.644503	547.001470	589.656558	393.507729	4027	4150	3873
gene1947	NC_008261.1	2226820	2227011	+	protein_coding	CPF_RS09660	CPF_1987	.	WP_003458496. 50S ribosomal p	2.502516	10.022406	1.968357	10.205735	1.271373	9.554160	9.382536	10.705915	9.728923	9.382536	10.705915	9.728923	579.304373	1963.671785	862.214247	2353	7625	4682
gene1948	NC_008261.1	2227254	2227883	-	protein_coding	CPF_RS09665	CPF_1988	.	WP_003449435. thiamine diphos	1.044886	6.665111	1.496014	6.399705	-1.431748	6.369366	6.633514	6.696860	6.115736	6.633514	6.696860	6.115736	78.033107	141.980368	60.164353	1040	1809	1072
gene1949	NC_008261.1	2227885	2228538	-	protein_coding	CPF_RS09670	CPF_1989	.	WP_011590942. ribulose-phosph	1.106847	7.352524	1.425713	7.165708	-1.288086	7.094697	7.279661	7.426116	6.914432	7.279661	7.426116	6.914432	127.426769	220.162781	110.020010	1763	2912	2035
gene1950	NC_008261.1	2228538	2229401	-	protein_coding	CPF_RS09675	CPF_1990	.	WP_011590943. ribosome biogen	-1.010558	5.942112	1.423066	5.674336	-1.438090	5.681575	5.949693	5.934541	5.425539	5.949693	5.934541	5.425539	46.941791	88.705028	36.421789	858	1550	890
gene1951	NC_008261.1	2229638	2231716	-	protein_coding	CPF_RS09680	CPF_1991	.	WP_003458522. serine/threonin	-1.989586	8.846823	-1.747393	8.757848	-1.138602	9.262858	9.356963	8.364495	9.169699	9.356963	8.364495	9.169699	568.241085	381.130610	578.411817	24992	16025	34010
gene1952	NC_008261.1	2231718	2232437	-	protein_coding	CPF_RS09685	CPF_1992	.	WP_003458503. serine/threonin	-2.180966	8.430221	-1.852668	8.319401	-1.177203	8.892989	9.011449	7.886481	8.776086	9.011449	7.886481	8.776086	434.490589	285.618744	427.436298	6618	4159	8704
gene1953	NC_008261.1	2232450	2233493	-	protein_coding	CPF_RS09690	CPF_1993	.	WP_003458521. 23S rRNA (aden	-1.940187	8.012666	-1.693541	7.919728	-1.145639	8.406366	8.505014	7.548819	8.308861	8.505014	7.548819	8.308861	307.662858	236.288881	321.911608	6795	4989	9505
gene1954	NC_008261.1	2233525	2234853	-	protein_coding	CPF_RS09695	CPF_1994	.	WP_011590944. 16S rRNA (cyto	-1.856530	8.191253	-1.601374	8.089625	-1.159336	8.542391	8.649706	7.757098	8.436408	8.649706	7.757098	8.436408	336.972537	265.274088	350.438454	9474	7130	13172
gene1955	NC_008261.1	2234864	2235559	-	protein_coding	CPF_RS09700	CPF_1995	.	WP_003465352. zinc metallopept	-1.927416	7.867283	-1.709254	7.785262	-1.127635	8.267739	8.354843	7.408175	8.181542	8.354843	7.408175	8.181542	278.119074	217.746669	285.554731	4095	3065	5621
gene1956	NC_008261.1	2235577	2236506	-	protein_coding	CPF_RS09705	CPF_1996	.	WP_011590945. methionyl-tRNA	-1.493268	8.375971	-1.403993	8.332900	-1.063586	8.625617	8.670200	8.091726	8.581263	8.670200	8.091726	8.581263	342.174205	319.324808	375.324757	6732	6006	9872
gene1957	NC_008261.1	2236526	2236969	-	protein_coding	CPF_RS09710	CPF_1997	.	WP_003458459. peptide deformy	-1.366004	7.633499	-1.063504	7.456113	-1.284436	7.679104	7.861794	7.411833	7.500659	7.861794	7.411833	7.500659	193.764640	218.386056	174.479506	1820	1961	2191
gene1958	NC_008261.1	2236983	2239178	-	protein_coding	CPF_RS09715	CPF_1998	.	WP_011590946. primosomal prot	3.016402	4.328185	2.331200	4.545910	1.293927	3.785751	3.604433	5.197262	3.976189	3.604433	5.197262	3.976189	8.416481	52.035279	12.462169	391	2311	774
gene1959	NC_008261.1	2239217	2240404	-	protein_coding	CPF_RS09720	CPF_1999	.	WP_003471651. bifunctional pho	2.143698	8.889690	2.136521	8.892263	1.003359	8.359059	8.356640	9.456742	8.361479	8.356640	9.456742	8.361479	278.129364	789.511166	332.178624	6990	18969	11161
gene1960	NC_008261.1	2240410	2240622	-	protein_coding	CPF_RS09725	CPF_2000	.	WP_003449449. DNA-directed R	1.909154	7.549181	1.884364	7.559203	1.013156	7.106534	7.097112	8.030046	7.115968	7.097112	8.030046	7.115968	112.959897	307.353853	126.657219	509	1324	763
gene1961	NC_008261.1	2240603	2241253	-	protein_coding	CPF_RS09730	CPF_2001	.	WP_011590947. guanylate kinase	1.907775	7.876925	1.952245	7.859272	-1.023310	7.408109	7.424749	8.356640	7.391506	7.424749	8.356640	7.391506	138.252204	380.452396	159.082863	1904	5009	2929
gene1962	NC_008261.1	2241243	2241512	-	protein_coding	CPF_RS09735	CPF_2002	.	WP_003458433. DUF370 domain	1.527543	7.545773	1.612636	7.504943	-1.055706	7.207143	7.246352	7.857566	7.168145	7.246352	7.857566	7.168145	125.878406	280.925962	129.645201	719	1534	990
gene1963	NC_008261.1	2241526	2242410	-	protein_coding	CPF_RS09740	CPF_2003	.	WP_003449417. YicC family prote	1.872828	7.196654	1.824600	7.216666	1.026432	6.777056	6.758263	7.663482	6.795901	6.758263	7.663482	6.795901	86.741886	254.883834	100.999405	1624	4562	2528
gene1964	NC_008261.1	2242630	2244027	-	protein_coding	CPF_RS09745	CPF_2004	.	WP_003471594. NCS2 family per	195.014503	3.499388	241.392913	3.079616	-1.237820	1.201119	1.364840	8.972277	1.057038	1.364840	8.972277	1.057038	1.217255	596.640112	0.961084	36	16869	38
gene1965	NC_008261.1	2244285	2245268	-	protein_coding	CPF_RS09750	CPF_2005	.	WP_003458466. asparaginase	1.730632	4.979918	1.363181	5.162936	1.269554	4.769015	4.599960	5.391259	4.944282	4.599960	5.391259	4.944282	18.254696	59.897878	25.943413	380	1192	722
gene1966	NC_008261.1	2245619	2247094	-	protein_coding	CPF_RS09755	CPF_2006	.	WP_003458520. stage IV sporula	-2.635578	7.531434	-1.577674	7.186125	-1.670546	7.884023	8.262868	6.864748	7.522547	8.262868	6.864748	7.522547	265.525763	157.181680	176.285852	8291	4692	7359
gene1967	NC_008261.1	2247427	2248422	-	protein_coding	CPF_RS09760	CPF_2007	.	WP_003458497. glycerol-3-phos	1.364164	8.226819	1.494414	8.158942	-1.095479	7.939806	8.005860	8.453877	7.874297	8.005860	8.453877	7.874297	225.054820	411.403783	229.932083	4742	8287	6477
gene1968	NC_008261.1	2248427	2249743	-	protein_coding	CPF_RS09765	CPF_2008	.	WP_003458514. ribosome biogen	1.080752	7.584738	1.360284	7.415687	-1.258647	7.361121	7.528927	7.640962	7.197054	7.528927	7.640962	7.197054	149.491100	252.035173	133.376964	4165	6713	4968
gene1969	NC_008261.1	2249746	2251083	-	protein_coding	CPF_RS09770	CPF_2009	.	WP_003458424. radical SAM prot	1.206468	6.860534	1.519390	6.688716	-1.259370	6.558015	6.726475	6.997265	6.393773	6.726475	6.997265	6.393773	84.471376	166.297868	73.384575	2391	4500	2777
gene1970	NC_008261.1	2251410	2253125	-	protein_coding	CPF_RS09775	CPF_2010	.	WP_003458438. HAMP domain-c	-1.405011	5.149974	-1.316907	5.105237	-1.066902	5.354184	5.401102	4.910521	5.307674	5.401102	4.910521	5.307674	32.422410	42.616833	34.142101	1177	1479	1657
gene1971	NC_008261.1	2253139	2253843	-	protein_coding	CPF_RS09780	CPF_2011	.	WP_003458458. peptidoglycan ec	-1.386668	5.252988	-1.396366	5.257793	1.006994	5.499115	5.494090	5.022467	5.504144	5.494090	5.022467	5.504144	34.530608	45.237738	38.517414	515	645	768
gene1972	NC_008261.1	2254009	2254458	-	protein_coding	CPF_RS09785	CPF_2012	.	WP_003449440. transcriptional r	-1.226636	9.386292	1.696693	8.850520	-2.081225	8.990553	9.534802	9.240095	8.477369	9.534802	9.240095	8.477369	668.713712	695.319225	354.677840	6366	6328	4514
gene1973	NC_008261.1	2254520	2254792	-	protein_coding	CPF_RS09790	CPF_2013	.	WP_003458476. YlmC/YmxH fam	1.831932	2.207390	-1.072251	2.736712	1.964292	2.248352	1.813487	2.686853	2.787496	1.813487	2.686853	2.787496	1.904654	7.788182	5.310143	11	43	41
gene1974	NC_008261.1	2254933	2255706	-	protein_coding	CPF_RS09795	CPF_2014	.	WP_003449454. RNA polymerase	1.519351	6.327651	-2.904238	7.365605	4.412556	7.022753	6.033113	6.636568	8.174728	6.033113	6.636568	8.174728	50.262604	137.541371	282.359756	823	2153	6181
gene1975	NC_008261.1	2255781	2256488	-	protein_coding	CPF_RS09800	CPF_2015	.	WP_003449468. RNA polymerase	1.855392	5.184640	-2.745488	6.336425	5.093958	5.814900	4.757913	5.649638	7.106700	4.757913	5.649638	7.106700	20.430278	72.632395	126.099435	306	1040	2525
gene1976	NC_008261.1	2256502	2257308	-	protein_coding	CPF_RS09805	CPF_2016	.	WP_003458523. sigma-E process	2.037740	4.846505	-2.078663	5.891346	4.235774	5.300134	4.360146	5.387115	6.442771	4.360146	5.387115	6.442771	14.936634	59.739469	76.761876	255	975	1752
gene1978	NC_008261.1	2257713	2258858	-	protein_coding	CPF_RS09815	CPF_2018	.	WP_003449411. cell division prot	1.166463	10.263931	1.260880	10.207017	-1.080943	10.097161	10.153462	10.375602	10.041172	10.153462	10.375602	10.041172	994.444814	1469.958417	1139.254888	24109	34069	36925
gene1979	NC_008261.1	2258872	2260110	-	protein_coding	CPF_RS09820	CPF_2019	.	WP_003478307. cell division prot	-1.023107	7.346920	-1.092052	7.393707	1.067388	7.410310	7.363417	7.330460	7.457501	7.363417	7.330460	7.457501	133.569381	208.199562	168.798451	3501	5217	5915
gene1980	NC_008261.1	2260188	2261240	-	protein_coding	CPF_RS09825	CPF_2020	.	WP_011590949. type IV pili twitcl	-1.014265	7.907502	-1.059001	7.938535	1.044107	7.948800	7.917726	7.897291	7.979995	7.917726	7.897291	7.979995	203.894198	287.518748	244.213816	4542	6123	7273
gene1981	NC_008261.1	2261421	2262122	-	protein_coding	CPF_RS09830	CPF_2021	.	WP_011590950. RNA polymerase	-1.092301	1.406997	1.223852	1.190151	-1.336815	1.245240	1.472123	1.344752	1.053323	1.472123	1.344752	1.053323	1.346725	2.183508	0.956977	20	31	19
gene1982	NC_008261.1	2262185	2263852	-	protein_coding	CPF_RS09835	CPF_2022	.	WP_011590951. stage V sporulat	-1.215684	0.880398	-1.250626	0.897659	1.028743	1.052726	1.032483	0.750715	1.073365	1.032483	0.750715	1.073365	0.793502	1.037534	0.975095	28	35	46
gene1983	NC_008261.1	2263965	2264591	-	protein_coding	CPF_RS09840	CPF_2023	.	WP_003459577. uridine kinase	1.190635	8.899772	1.079490	8.971183	1.102961	8.845205	8.774796	9.026528	8.916178	8.774796	9.026528	8.916178	371.676828	614.564424	473.410814	4930	7793	8395
gene1984	NC_008261.1	2264591	2265820	-	protein_coding	CPF_RS09845	CPF_2024	.	WP_003459619. peptidase U32	-1.342166	8.608239	-1.238470	8.551462	-1.083729	8.764944	8.823138	8.398575	8.707133	8.823138	8.398575	8.707133	386.884264	391.346203	407.735594	10067	9735	14184
gene1985	NC_008261.1	2265826	2266470	-	protein_coding	CPF_RS09850	CPF_2025	.	WP_003459682. O-methyltransfe	-1.241271	7.451793	-1.186134	7.419627	-1.046484	7.576487	7.609333	7.297515	7.543782	7.609333	7.297515	7.543782	158.006572	204.453044	178.597913	2156	2667	3258
gene1986	NC_008261.1	2266486	2267514	-	protein_coding	CPF_RS09855	CPF_2026	.	WP_003451288. endolytic transg	-1.081672	7.840455	-1.111790	7.860098	1.027844	7.917077	7.897291	7.784028	7.936912	7.897291	7.784028	7.936912	200.059368	268.708911	239.085955	4355	5592	6958
gene1987	NC_008261.1	2267657	2269489	-	protein_coding	CPF_RS09860	CPF_2027	.	WP_003459575. translational GTI	2.954803	6.844384	2.684076	6.921632	1.100864	6.176258	6.107329	7.670391	6.245965	6.107329	7.670391	6.245965	53.124012	255.537919	65.603828	2060	9473	3401
gene1988	NC_008261.1	2269646	2271313	-	protein_coding	CPF_RS09865	CPF_2028	.	WP_003459637. ribonuclease J	-1.215623	10.702557	-1.192511	10.688882	-1.019381	10.830476	10.844332	10.562636	10.816638	10.844332	10.562636	10.816638	1524.147542	1698.709722	1754.428555	53782	57304	82765
gene1989	NC_008261.1	2271384	2271836	-	protein_coding	CPF_RS09870	CPF_2029	.	WP_003451087. transcriptional r	-1.083012	10.577482	1.025102	10.502215	-1.110197	10.559486	10.635163	10.520114	10.484347	10.635163	10.520114	10.484347	1351.735746	1654.527467	1483.856058	12954	15158	19011
gene1990	NC_008261.1	2271857	2272111	-	protein_coding	CPF_RS09875	CPF_2030	.	WP_003459598. DUF1292 domai	-1.134149	9.644165	-1.037340	9.580195	-1.093324	9.670822	9.735397	9.553787	9.606676	9.735397	9.553787	9.606676	752.613426	836.314368	786.745315	4060	4313	5674
gene1991	NC_008261.1	2272127	2272540	-	protein_coding	CPF_RS09880	CPF_2031	.	WP_003459573. Holliday junction	-1.292259	10.172516	-1.113348	10.066404	-1.160696	10.251086	10.359144	9.989249	10.144155	10.359144	9.989249	10.144155	1123.634328	1101.903062	1211.986195	9841	9226	14191
gene1992	NC_008261.1	2272588	2272845	-	protein_coding	CPF_RS09885	CPF_2032	.	WP_003451703. IreB family regul	-2.730640	12.202163	-3.031054	12.272909	1.110016	13.023352	12.948279	11.499040	13.098859	12.948279	11.499040	13.098859	7606.081649	3511.425458	7842.438283	41514	18322	57225
gene1993	NC_008261.1	2272989	2275628	-	protein_coding	CPF_RS09890	CPF_2033	.	WP_003459676. alanine--tRNA l	1.909601	7.123967	1.858743	7.144726	1.027361	6.692042	6.672598	7.605869	6.711542	6.672598	7.605869	6.711542	80.502336	246.611423	94.300812	4496	13167	7041
gene1995	NC_008261.1	2277134	2277616	-	protein_coding	CPF_RS09900	CPF_2036	.	WP_003459697. photosystem re	4.834500	1.739245	6.506560	1.283549	-1.345860	0.694496	0.941061	3.214428	0.512533	0.941061	3.214428	0.512533	0.685073	12.284695	0.366023	7	120	5
gene1996	NC_008261.1	2277896	2278975	-	protein_coding	CPF_RS09905	CPF_2037	.	WP_003459581. tRNA(5-methyla	8.939511	10.161427	13.103868	9.834080	-1.465837	8.423069	8.703448	11.863643	8.151723	8.703448	11.863643	8.151723	355.532090	4405.675416	278.573488	8123	96229	8509
gene1997	NC_008261.1	2279000	2279428	-	protein_coding	CPF_RS09910	CPF_2038	.	WP_003451659. Fe-S cluster as	7.084884	9.979677	10.433903	9.652791	-1.472699	8.382871	8.666752	11.491496	8.108289	8.666752	11.491496	8.108289	341.688673	3470.548894	267.120213	3101	30111	3241
gene1998	NC_008261.1	2279431	2280627	-	protein_coding	CPF_RS09915	CPF_2039	.	WP_003473085. cysteine desulfu	4.558705	10.736488	8.005110	10.277007	-1.756005	9.282771	9.697801	11.886425	8.885503	9.697801	11.886425	8.885503	734.917063	4470.743728	456.224681	18610	108229	15445
gene1999	NC_008261.1	2280620	2281081	-	protein_coding	CPF_RS09920	CPF_2040	.	WP_003451273. Rrf2 family trans	1.871571	10.962920	3.494691	10.482997	-1.867250	10.059576	10.520114	11.424364	9.619199	10.520114	11.424364	9.619199	1266.162189	3305.811557	786.978512	12375	30888	10283
gene2001	NC_008261.1	2282660	2283559	-	protein_coding	CPF_RS09930	CPF_2043	.	WP_003458734. GGDEF domain-	1.116243	3.459474	1.131057	3.449730	-1.013271	3.371534	3.381058	3.539709	3.362037	3.381058	3.539709	3.362037	7.090508	16.152327	8.132290	135	294	207
gene2002	NC_008261.1	2283744	2284172	-	protein_coding	CPF_RS09935	CPF_2044	.	WP_003458736. MOSC domain-c	1.102471	4.482359	1.251167	4.388669	-1.134875	4.320310	4.412541	4.553281	4.230007	4.412541	4.553281	4.230007	15.646498	32.733416	15.412367	142	284	187
gene2003	NC_008261.1	2284175	2285170	-	protein_coding	CPF_RS09940	CPF_2045	.	WP_003458692. GTP 3',8-cyclas	-1.016548	3.720671	1.045747	3.676442	-1.063052	3.688159	3.732529	3.708851	3.644317	3.732529	3.708851	3.644317	9.444519	18.368457	9.904439	199	370	279
gene2004	NC_008261.1	2285250	2286263	-	protein_coding	CPF_RS09945	CPF_2046	.	WP_003458733. molybdopterin-b	1.003039	3.941719	1.202703	3.808441	-1.199059	3.806327	3.939530	3.943908	3.677627	3.939530	3.943908	3.677627	10.908474	21.845920	10.181925	234	448	292
gene2005	NC_008261.1	2286381	2286977	-	protein_coding	CPF_RS09950	CPF_2047	.	WP_011590955. molybdenum cof	1.392307	2.357279	1.386537	2.360592	1.004162	2.133593	2.130599	2.608077	2.136591	2.130599	2.608077	2.136591	2.454559	7.371332	3.138966	31	89	53
gene2006	NC_008261.1	2287022	2288242	-	protein_coding	CPF_RS09955	CPF_2048	.	WP_011590956. molybdopterin m	1.404121	2.671910	1.584338	2.574688	-1.128349	2.349549	2.438270	2.927937	2.264057	2.438270	2.927937	2.264057	3.368137	9.638103	3.474966	87	238	120
gene2007	NC_008261.1	2288581	2289063	-	protein_coding	CPF_RS09960	CPF_2049	.	WP_003458698. molybdopterin-g	1.253759	5.823958	1.344112	5.772104	-1.072066	5.612691	5.663112	5.989372	5.562718	5.663112	5.989372	5.562718	38.853441	91.828099	39.750053	397	897	543
gene2008	NC_008261.1	2289139	2290362	-	protein_coding	CPF_RS09965	CPF_2050	.	WP_011590958. NAD(P)/FAD-de	-1.037874	5.736876	1.167857	5.597072	-1.212088	5.623295	5.763754	5.710123	5.486259	5.763754	5.710123	5.486259	41.477156	75.623140	38.188716	1074	1872	1322
gene2009	NC_008261.1	2290364	2290777	-	protein_coding	CPF_RS09970	CPF_2051	.	WP_003458719. nitrate reductas	-1.251894	5.512023	-1.113084	5.429080	-1.124707	5.591044	5.676461	5.352349	5.506911	5.676461	5.352349	5.506911	39.049176	58.403490	38.517777	342	489	451
gene2010	NC_008261.1	2290790	2292868	-	protein_coding	CPF_RS09975	CPF_2052	.	WP_003481844. nitrate reductas	-1.145024	5.579878	-1.006167	5.487478	-1.138005	5.584390	5.678422	5.483045	5.491915	5.678422	5.483045	5.491915	39.062027	63.977619	38.334026	1718	2690	2254
gene2012	NC_008261.1	2293804	2295081	-	protein_coding	CPF_RS09985	CPF_2054	.	WP_011590960. FAD-dependent	-1.391853	0.890812	-1.443797	0.910871	1.037320	1.186827	1.160691	0.683684	1.213552	1.160691	0.683684	1.213552	0.924688	0.889871	1.134326	25	23	41
gene2013	NC_008261.1	2295113	2296093	-	protein_coding	CPF_RS09990	CPF_2055	.	WP_003463476. hypothetical pro	1.024608	0.635043	1.135834	0.553370	-1.108555	0.538300	0.617749	0.652821	0.469070	0.617749	0.652821	0.469070	0.385485	0.856861	0.324383	8	17	9
gene2014	NC_008261.1	2296338	2297213	-	protein_coding	CPF_RS09995	CPF_2056	.	WP_003451263. D-alanyl-D-alan	-1.094176	0.378542	-1.029596	0.339535	-1.062723	0.402725	0.448992	0.319146	0.361225	0.448992	0.319146	0.361225	0.269806	0.338671	0.242177	5	6	6
gene2016	NC_008261.1	2297980	2298399	+	protein_coding	CPF_RS10005	CPF_2058	.	WP_003458743. sporulation prote	-1.392886	1.212887	-1.028279	1.017096	-1.354580	1.237112	1.475256	0.997179	1.037411	1.475256	0.997179	1.037411	1.350573	1.412740	0.926037	12	12	11
gene2017	NC_008261.1	2298728	2299315	-	protein_coding	CPF_RS10010	CPF_2059	.	WP_003451108. segregation/cor	2.203606	3.034005	2.550650	2.904060	-1.157489	2.409330	2.517138	3.657004	2.306139	2.517138	3.657004	2.306139	3.617606	17.659250	3.607937	45	210	60
gene2018	NC_008261.1	2299308	2300054	-	protein_coding	CPF_RS10015	CPF_2060	.	WP_003458723. segregation/cor	3.049835	2.260462	3.049835	2.260462	1.000000	1.594945	1.594945	3.203676	1.594945	1.594945	3.203676	1.594945	1.518717	12.113252	1.751323	24	183	37
gene2019	NC_008261.1	2300325	2301533	-	protein_coding	CPF_RS10020	CPF_2061	.	WP_003469040. D-alanyl-D-alan	-1.896299	1.756587	-4.932688	2.225733	2.601219	2.886171	2.277816	1.354630	3.657004	2.277816	1.354630	3.657004	2.854189	2.208502	9.972708	73	54	341
gene2020	NC_008261.1	2301785	2303086	-	protein_coding	CPF_RS10025	CPF_2062	deoA	WP_003458658. pyrimidine-nucle	-3.430785	9.608727	-6.639045	10.033518	1.935139	11.004979	10.539059	8.760520	11.491496	10.539059	8.760520	11.491496	1282.790211	520.511134	2773.847873	35333	13706	102143
gene2021	NC_008261.1	2303333	2304205	-	protein_coding	CPF_RS10030	CPF_2063	.	WP_003451532. tyrosine recomb	-3.421696	3.165651	-2.659707	3.024726	-1.286493	3.989163	4.175022	2.400310	3.811578	4.175022	2.400310	3.811578	12.941057	6.117018	11.178405	239	108	276
gene2022	NC_008261.1	2304255	2304875	-	protein_coding	CPF_RS10035	CPF_2064	.	WP_003451331. stage II sporulat	-2.495846	1.712399	-2.429195	1.698945	-1.027437	2.475264	2.494867	1.175338	2.455816	2.494867	1.175338	2.455816	3.577605	1.831330	4.042516	47	23	71
gene2023	NC_008261.1	2305009	2305539	-	protein_coding	CPF_RS10040	CPF_2065	.	WP_003458749. NUDIX hydrolase	1.082188	5.395931	-1.039334	5.480966	1.124754	5.423398	5.339256	5.453207	5.508866	5.339256	5.453207	5.508866	30.890224	62.482483	38.620553	347	671	580
gene2024	NC_008261.1	2305601	2305906	-	protein_coding	CPF_RS10045	CPF_2066	.	WP_003458729. hypothetical pro	1.454963	3.643896	1.318054	3.719878	1.103872	3.453982	3.383431	3.924413	3.526003	3.383431	3.924413	3.526003	7.105956	21.652779	9.128316	46	134	79
gene2025	NC_008261.1	2305983	2306813	-	protein_coding	CPF_RS10050	CPF_2067	.	WP_003451549. sporulation tran	1.083837	9.664575	1.317021	9.522113	-1.215147	9.465067	9.606676	9.722824	9.325545	9.606676	9.722824	9.325545	701.599108	936.199495	641.162957	12334	15734	15069
gene2026	NC_008261.1	2306987	2308147	-	protein_coding	CPF_RS10055	CPF_2068	.	WP_003451227. SpoIVB peptidas	-1.162107	0.557408	-1.149358	0.550810	-1.011092	0.668212	0.676216	0.459474	0.660302	0.676216	0.459474	0.660302	0.447864	0.553658	0.487273	11	13	16
gene2027	NC_008261.1	2308324	2310021	-	protein_coding	CPF_RS10060	CPF_2069	.	WP_003458741. DNA repair prote	-1.014246	6.284667	-1.002743	6.276448	-1.011472	6.286647	6.294880	6.274472	6.278424	6.294880	6.274472	6.278424	62.497805	107.656943	67.425500	2245	3697	3238
gene2028	NC_008261.1	2310037	2310489	-	protein_coding	CPF_RS10065	CPF_2070	.	WP_003451580. arginine repress	-1.371214	6.666610	-1.253769	6.603892	-1.093674	6.833328	6.898225	6.442771	6.769042	6.898225	6.442771	6.769042	96.209693	120.067305	98.190044	922	1100	1258
gene2029	NC_008261.1	2310512	2311342	-	protein_coding	CPF_RS10070	CPF_2071	.	WP_003458666. NAD(+) kinase	-1.093236	6.495247	1.127612	6.344036	-1.232746	6.407152	6.559868	6.431263	6.257992	6.559868	6.431263	6.257992	73.777691	119.300876	66.247974	1297	2005	1557
gene2030	NC_008261.1	2311427	2312242	-	protein_coding	CPF_RS10075	CPF_2072	.	WP_003458690. TlyA family rRNA	1.174265	6.250873	1.389072	6.126181	-1.182929	6.013666	6.136068	6.367826	5.893705	6.136068	6.367826	5.893705	54.395321	113.434710	51.260118	939	1872	1183
gene2031	NC_008261.1	2312254	2314113	-	protein_coding	CPF_RS10080	CPF_2073	.	WP_003458746. 1-deoxy-D-xylu	1.038983	5.406756	1.204582	5.298446	-1.159385	5.271481	5.379240	5.434413	5.165880	5.379240	5.434413	5.165880	31.869166	61.727623	30.719449	1254	2322	1616
gene2032	NC_008261.1	2314134	2315006	-	protein_coding	CPF_RS10085	CPF_2074	.	WP_003458680. polyprenyl synth	1.195720	3.875402	1.185724	3.881658	1.008430	3.754658	3.748607	4.006486	3.760719	3.748607	4.006486	3.760719	9.529816	22.995458	10.732889	176	406	265
gene2033	NC_008261.1	2314996	2315223	-	protein_coding	CPF_RS10090	CPF_2075	.	WP_003451121. exodeoxyribonu	1.240606	3.121372	1.477208	2.986096	-1.190715	2.841018	2.969721	3.280766	2.717893	2.969721	3.280766	2.717893	5.183120	12.795211	5.117574	25	59	33
gene2034	NC_008261.1	2315242	2316444	-	protein_coding	CPF_RS10095	CPF_2076	.	WP_003458744. exodeoxyribonu	1.236015	3.562534	1.354625	3.492857	-1.095962	3.346212	3.412964	3.718660	3.280766	3.412964	3.718660	3.280766	7.230000	18.372666	7.671140	184	447	261
gene2035	NC_008261.1	2316446	2317279	-	protein_coding	CPF_RS10100	CPF_2077	.	WP_011590961. bifunctional met	1.279868	7.090196	1.426188	7.009669	-1.114324	6.835902	6.914432	7.270427	6.758263	6.914432	7.270427	6.758263	97.544082	202.052308	97.127912	1721	3408	2291
gene2036	NC_008261.1	2317328	2317735	-	protein_coding	CPF_RS10105	CPF_2078	.	WP_003451187. N utilization sub	1.734105	5.439112	2.141136	5.272974	-1.234721	4.902041	5.056493	5.850684	4.752308	5.056493	5.850684	4.752308	25.488755	84.227696	22.271936	220	695	257
gene2037	NC_008261.1	2317854	2318243	-	protein_coding	CPF_RS10110	CPF_2079	.	WP_003451410. Asp23/Gls24 fa	-1.128502	11.403958	1.660504	10.945165	-1.873883	11.029181	11.491496	11.317087	10.585466	11.491496	11.317087	10.585466	2476.950885	3151.478770	1600.891577	20436	24857	17658
gene2038	NC_008261.1	2318328	2318825	-	protein_coding	CPF_RS10115	CPF_2080	.	WP_003451536. stage III sporula	-3.043323	2.662595	-2.634946	2.584222	-1.154985	3.478330	3.583820	1.978173	3.375946	3.583820	1.978173	3.375946	8.258022	4.170136	8.164950	87	42	115
gene2039	NC_008261.1	2318851	2319456	-	protein_coding	CPF_RS10120	CPF_2081	.	WP_003451336. stage III sporula	-2.279418	2.174218	-2.273732	2.172843	-1.002501	2.846517	2.848320	1.659654	2.844716	2.848320	1.659654	2.844716	4.758207	3.182162	5.484540	61	39	94
gene2040	NC_008261.1	2319471	2320028	-	protein_coding	CPF_RS10125	CPF_2082	.	WP_003468981. stage III sporula	-1.303587	1.217349	-1.250188	1.191268	-1.042713	1.393025	1.423523	1.041036	1.363181	1.423523	1.041036	1.363181	1.270700	1.506416	1.394034	15	17	22
gene2041	NC_008261.1	2320036	2321208	-	protein_coding	CPF_RS10130	CPF_2083	.	WP_003458684. stage III sporula	-1.891095	1.942574	-1.690835	1.877620	-1.118439	2.373700	2.455816	1.536595	2.294330	2.455816	1.536595	2.294330	3.425366	2.782122	3.587021	85	66	119
gene2042	NC_008261.1	2321219	2321605	-	protein_coding	CPF_RS10135	CPF_2084	.	WP_003451406. stage III sporula	-1.934016	2.470051	-1.676903	2.383566	-1.153326	2.886525	2.991259	2.039659	2.785459	2.991259	2.039659	2.785459	5.252228	4.471851	5.299099	43	35	58
gene2043	NC_008261.1	2321693	2321890	-	protein_coding	CPF_RS10140	CPF_2085	.	WP_003451671. stage III sporula	-1.415580	1.675763	-1.443236	1.687744	1.019537	1.959015	1.945108	1.443715	1.973022	1.945108	1.443715	1.973022	2.148639	2.497268	2.678620	9	10	15
gene2044	NC_008261.1	2321966	2322481	-	protein_coding	CPF_RS10145	CPF_2086	.	WP_003458670. stage III sporula	-1.590915	1.184916	-1.724156	1.228023	1.083751	1.623251	1.566270	0.896414	1.682304	1.566270	0.896414	1.682304	1.465738	1.245730	1.918639	16	13	28
gene2045	NC_008261.1	2322628	2323512	-	protein_coding	CPF_RS10150	CPF_2087	.	WP_003451516. stage III sporula	-2.408486	2.381042	-2.340866	2.365201	-1.028887	3.077473	3.098084	1.829957	3.057000	3.098084	1.829957	3.057000	5.715137	3.687491	6.592129	107	66	165
gene2046	NC_008261.1	2323710	2324072	-	protein_coding	CPF_RS10155	CPF_2088	.	WP_003458718. hypothetical pro	5.576633	5.877334	3.830439	6.202398	1.455873	5.030610	4.766958	7.246352	5.308843	4.766958	7.246352	5.308843	20.574845	197.102524	34.188930	158	1447	351
gene2047	NC_008261.1	2324186	2324743	-	protein_coding	CPF_RS10160	CPF_2089	.	WP_003451649. elongation facto	3.860841	9.459833	3.860841	9.459833	1.000000	8.535432	8.535432	10.484347	8.535432	8.535432	10.484347	8.535432	311.745166	1605.751151	365.427113	3680	18121	5767
gene2054	NC_008261.1	2327604	2328032	-	protein_coding	CPF_RS10195	CPF_2096	.	WP_003458702. type IV pilin	-1.109168	0.276566	1.055852	0.168032	-1.171117	0.219468	0.361225	0.211748	0.133341	0.361225	0.211748	0.133341	0.220373	0.230517	0.082419	2	2	1
gene2055	NC_008261.1	2328025	2329044	-	protein_coding	CPF_RS10200	CPF_2097	.	WP_011590966. type II secretion	-1.174375	0.329259	-1.020278	0.247188	-1.151035	0.349112	0.465024	0.233131	0.262092	0.465024	0.233131	0.262092	0.278059	0.242382	0.173322	6	5	5
gene2056	NC_008261.1	2329031	2330419	-	protein_coding	CPF_RS10205	CPF_2098	.	WP_003458669. type II/IV secre	-1.222465	0.604713	-1.112305	0.548362	-1.099037	0.695278	0.766727	0.476934	0.630487	0.766727	0.476934	0.630487	0.510476	0.569571	0.458200	15	16	18
gene2057	NC_008261.1	2330518	2331549	-	protein_coding	CPF_RS10210	CPF_2099	.	WP_003458660. hypothetical pro	1.468906	5.987731	1.228264	6.121416	1.195921	5.844417	5.716781	6.271523	5.974903	5.716781	6.271523	5.974903	40.124582	107.611909	54.064516	876	2246	1578
gene2058	NC_008261.1	2331566	2332384	-	protein_coding	CPF_RS10215	CPF_2100	.	WP_003458678. diaminopimelate	1.094798	6.593484	1.106031	6.586043	-1.010260	6.521108	6.528475	6.659140	6.513748	6.528475	6.659140	6.513748	71.915126	138.738311	81.594882	1246	2298	1890
gene2059	NC_008261.1	2332476	2334203	+	protein_coding	CPF_RS10220	CPF_2101	.	WP_011590967. DUF814 domain	-1.158002	4.595405	1.348794	4.269517	-1.561906	4.368964	4.702442	4.490805	4.059134	4.702442	4.490805	4.059134	19.695856	31.647665	13.218245	720	1106	646
gene2060	NC_008261.1	2334314	2335441	-	protein_coding	CPF_RS10225	CPF_2102	.	WP_003451545. class I SAM-dep	1.166030	7.283343	1.112554	7.317646	1.048066	7.207168	7.173383	7.394988	7.241112	7.173383	7.394988	7.241112	119.809475	215.755954	137.983117	2859	4922	4402
gene2061	NC_008261.1	2335461	2335997	-	protein_coding	CPF_RS10230	CPF_2103	.	WP_003451681. bifunctional pyri	-1.086655	7.538053	-1.033303	7.501938	-1.051633	7.561835	7.598238	7.478344	7.525607	7.598238	7.478344	7.525607	157.126719	226.419863	176.657224	1785	2459	2683
gene2062	NC_008261.1	2336001	2336921	-	protein_coding	CPF_RS10235	CPF_2104	.	WP_003458700. RluA family pseu	1.086147	7.336237	1.017255	7.383738	1.067723	7.323986	7.276870	7.396089	7.371408	7.276870	7.396089	7.371408	127.387923	215.929867	156.748995	2482	4022	4083
gene2063	NC_008261.1	2337059	2337685	-	protein_coding	CPF_RS10240	CPF_2105	.	WP_003451394. DivIVA domain-c	-1.495850	10.574917	-1.082154	10.345220	-1.382289	10.633295	10.869389	10.288424	10.402330	10.869389	10.288424	10.402330	1551.166480	1394.894845	1412.732306	20575	17688	25052
gene2064	NC_008261.1	2337698	2338459	-	protein_coding	CPF_RS10245	CPF_2106	.	WP_003475151. RNA-binding pro	-1.186084	9.909620	1.063988	9.742439	-1.261978	9.864216	10.033488	9.787282	9.697801	10.033488	9.787282	9.697801	895.773747	967.115071	843.065016	14440	14904	18169
gene2065	NC_008261.1	2338461	2338910	-	protein_coding	CPF_RS10250	CPF_2107	.	WP_003451474. cell division prot	-1.631979	10.974769	-1.114686	10.705175	-1.464070	11.055353	11.333765	10.627143	10.783781	11.333765	10.627143	10.783781	2159.821338	1808.730999	1730.331280	20561	16461	22022
gene2066	NC_008261.1	2338929	2339588	-	protein_coding	CPF_RS10255	CPF_2108	.	WP_003458739. YggS family pyric	-1.783899	9.157205	-1.431900	9.004444	-1.245826	9.424350	9.584235	8.749201	9.267132	9.584235	8.749201	9.267132	693.437383	509.592436	615.332552	9682	6802	11486
gene2067	NC_008261.1	2339688	2340419	-	protein_coding	CPF_RS10260	CPF_2109	.	WP_003458728. DUF881 domain	1.668937	6.593732	1.564894	6.642657	1.066485	6.280871	6.234610	6.973539	6.327474	6.234610	6.973539	6.327474	59.668758	164.009076	69.749504	924	2428	1444
gene2068	NC_008261.1	2340432	2340770	-	protein_coding	CPF_RS10265	CPF_2110	.	WP_003451069. DUF1290 domai	1.204866	5.786899	1.444559	5.651358	-1.198937	5.521595	5.654024	5.922897	5.392268	5.654024	5.922897	5.392268	38.485355	88.244157	35.670683	276	605	342
gene2069	NC_008261.1	2340783	2341499	-	protein_coding	CPF_RS10270	CPF_2111	.	WP_003451626. DUF881 domain	1.394198	6.097025	1.703726	5.944695	-1.222012	5.715560	5.862018	6.341454	5.572760	5.862018	6.341454	5.572760	43.775895	111.856693	39.993251	664	1622	811
gene2070	NC_008261.1	2341513	2342259	-	protein_coding	CPF_RS10275	CPF_2112	.	WP_003458747. cell division prot	2.411506	4.684329	2.175549	4.768596	1.108459	4.165816	4.092201	5.362135	4.240756	4.092201	5.362135	4.240756	12.213013	58.845254	15.525238	193	889	328
gene2071	NC_008261.1	2342417	2343541	-	protein_coding	CPF_RS10280	CPF_2113	.	WP_003451300. stage V sporulat	1.655131	6.634512	1.346233	6.790084	1.229453	6.428270	6.280989	7.007934	6.579005	6.280989	7.007934	6.579005	61.934277	168.072106	84.230094	1474	3824	2680
gene2072	NC_008261.1	2343569	2344540	-	protein_coding	CPF_RS10285	CPF_2114	.	WP_003458662. phospho-N-ace	1.335594	6.264043	1.194480	6.346775	1.118138	6.138800	6.058779	6.476261	6.219878	6.058779	6.476261	6.219878	51.355122	122.749954	64.495214	1056	2413	1773
gene2073	NC_008261.1	2344590	2345951	-	protein_coding	CPF_RS10290	CPF_2115	.	WP_011590968. UDP-N-acetylm	1.148466	5.778831	-1.038855	5.906980	1.193089	5.805794	5.679840	5.879547	5.934541	5.679840	5.879547	5.934541	39.148768	86.366956	52.777071	1128	2379	2033
gene2074	NC_008261.1	2345964	2347418	-	protein_coding	CPF_RS10295	CPF_2116	.	WP_011590969. UDP-N-acetylm	-1.049558	5.899547	-1.075028	5.916716	1.024268	5.951812	5.934541	5.864760	5.969134	5.934541	5.864760	5.969134	46.425367	85.400375	53.704947	1429	2513	2210
gene2075	NC_008261.1	2347513	2349732	-	protein_coding	CPF_RS10300	CPF_2117	.	WP_011590970. stage V sporulat	-1.253659	6.285953	-1.156249	6.228840	-1.084247	6.392527	6.451140	6.124995	6.334446	6.451140	6.124995	6.334446	68.967436	99.448622	70.333318	3239	4465	4416
gene2076	NC_008261.1	2349920	2350348	-	protein_coding	CPF_RS10305	CPF_2118	.	WP_003458745. cell division prot	1.070650	8.036077	1.394359	7.842004	-1.302349	7.794098	7.986985	8.085471	7.605869	7.986985	8.085471	7.605869	219.601911	317.537186	187.585809	1993	2755	2276
gene2077	NC_008261.1	2350420	2351352	-	protein_coding	CPF_RS10310	CPF_2119	.	WP_003451137. ribosomal RNA s	1.044255	7.851428	1.515272	7.577010	-1.451055	7.546925	7.820253	7.882727	7.283150	7.820253	7.882727	7.283150	187.306960	285.122121	143.553353	3697	5380	3788
gene2078	NC_008261.1	2351900	2352997	-	protein_coding	CPF_RS10315	CPF_2121	.	WP_003451384. redox-regulated	3.152125	7.565811	3.199081	7.553904	-1.014897	6.772165	6.782840	8.439164	6.761507	6.782840	8.439164	6.761507	88.211605	402.817459	97.314406	2049	8945	3022
gene2079	NC_008261.1	2353156	2353953	+	protein_coding	CPF_RS10320	CPF_2122	.	WP_011590971. delta-lactam-bi	1.269589	0.188597	1.118573	0.337170	1.135008	0.148728	0.083191	0.427553	0.265894	0.083191	0.427553	0.265894	0.059236	0.495698	0.177232	1	8	4
gene2080	NC_008261.1	2354132	2354767	-	protein_coding	CPF_RS10325	CPF_2123	.	WP_011590972. methenyltetrahy	1.265075	4.043906	1.394351	3.970041	-1.102189	3.807018	3.877850	4.217073	3.737479	3.877850	4.217073	3.737479	10.405358	26.744323	10.562859	140	344	190
gene2081	NC_008261.1	2354796	2356082	-	protein_coding	CPF_RS10330	CPF_2124	.	WP_011590973. hypothetical pro	1.586319	4.690232	1.822477	4.581507	-1.148871	4.267903	4.369185	5.034868	4.168968	4.369185	5.034868	4.168968	15.095565	45.565530	14.588176	411	1186	531
gene2082	NC_008261.1	2356082	2356798	-	protein_coding	CPF_RS10335	CPF_2125	.	WP_003481897. glycosyltransfer	1.226668	4.821753	1.527562	4.655741	-1.245294	4.515617	4.676632	4.971377	4.360146	4.676632	4.971377	4.360146	19.448628	44.135810	16.766591	295	640	340
gene2083	NC_008261.1	2356814	2357911	-	protein_coding	CPF_RS10340	CPF_2126	.	WP_003460180. tetratricopeptid	1.256569	5.119141	1.937725	4.785622	-1.542077	4.634088	4.957046	5.286536	4.332172	4.957046	5.286536	4.332172	23.548925	55.480281	16.519619	547	1232	513
gene2084	NC_008261.1	2358203	2359930	-	protein_coding	CPF_RS10345	CPF_2127	.	WP_011590974. phospho-sugar	1.365055	8.459873	1.550113	8.365170	-1.135568	8.146147	8.238371	8.687330	8.054956	8.238371	8.687330	8.054956	259.000510	491.368625	255.668684	9468	17172	12495
gene2085	NC_008261.1	2360086	2360697	-	protein_coding	CPF_RS10350	CPF_2128	.	WP_003469843. 50S ribosomal p	-1.380740	5.368365	-1.352462	5.354052	-1.020908	5.591181	5.606128	5.140686	5.576274	5.606128	5.140686	5.576274	37.615224	49.930663	40.037488	487	618	693
gene2086	NC_008261.1	2360831	2365270	-	protein_coding	CPF_RS10355	CPF_2129	.	WP_011590976. hypothetical pro	-2.858097	8.197631	-3.390956	8.309318	1.186439	9.112569	8.990085	7.475030	9.236722	8.990085	7.475030	9.236722	431.498948	226.125898	607.301760	40530	20305	76261
gene2087	NC_008261.1	2365428	2368226	-	protein_coding	CPF_RS10360	CPF_2130	.	WP_011590977. calx-beta domai	-3.150622	9.408103	-2.708672	9.307714	-1.163161	10.162661	10.272271	8.616634	10.054220	10.272271	8.616634	10.054220	1050.023463	473.083667	1145.294016	62175	26780	90664
gene2088	NC_008261.1	2368450	2369166	-	protein_coding	CPF_RS10365	CPF_2131	.	WP_003478392. glycerophospho	1.273653	5.118261	1.005100	5.292050	1.267191	5.114714	4.946749	5.295721	5.288382	4.946749	5.295721	5.288382	23.272426	55.790422	33.779750	353	809	685
gene2089	NC_008261.1	2369170	2370192	-	protein_coding	CPF_RS10370	CPF_2132	.	WP_003460161. alpha-hydroxy-a	1.024380	6.040807	-1.124689	6.142385	1.152109	6.124743	6.023456	6.058207	6.227733	6.023456	6.058207	6.227733	50.134907	95.798409	65.047259	1085	1982	1882
gene2090	NC_008261.1	2370206	2370649	-	protein_coding	CPF_RS10375	CPF_2133	.	WP_011590978. DUF523 domain	1.140724	4.880396	1.224874	4.827761	-1.073769	4.734725	4.786345	4.976295	4.683662	4.786345	4.976295	4.683662	20.973425	44.323126	21.342085	197	398	268
gene2091	NC_008261.1	2370888	2373071	-	protein_coding	CPF_RS10380	CPF_2134	.	WP_011590979. stage V sporulat	-1.876689	4.428412	-1.669987	4.351762	-1.123775	4.820817	4.905728	3.997538	4.737375	4.905728	3.997538	4.737375	22.682700	22.911745	22.082424	1048	1012	1364
gene2092	NC_008261.1	2373335	2375695	-	protein_coding	CPF_RS10385	CPF_2135	.	WP_011590980. endonuclease M	1.539461	6.051679	1.185827	6.246732	1.298217	5.933743	5.748463	6.370889	6.124995	5.748463	6.370889	6.124995	41.083504	113.677400	60.726729	2052	5428	4055
gene2093	NC_008261.1	2375709	2378060	-	protein_coding	CPF_RS10390	CPF_2136	.	WP_003469841. peptidase	1.876720	4.465341	1.746474	4.522405	1.074577	4.085820	4.034265	4.942479	4.138034	4.034265	4.942479	4.138034	11.777318	43.580506	14.281417	586	2073	950
gene2094	NC_008261.1	2378459	2379247	+	protein_coding	CPF_RS10395	CPF_2137	.	WP_003469844. PHP domain-co	-1.248051	3.409795	1.000000	3.253701	-1.248051	3.409795	3.573378	3.253701	3.253701	3.573378	3.253701	3.253701	8.207855	12.659153	7.483840	137	202	167
gene2095	NC_008261.1	2379538	2380893	-	protein_coding	CPF_RS10400	CPF_2138	.	WP_011590981. cell division prot	-1.111899	6.511532	1.338196	6.221776	-1.487939	6.295314	6.588495	6.435468	6.015179	6.588495	6.435468	6.015179	75.262573	119.567186	55.461654	2159	3279	2127
gene2096	NC_008261.1	2380974	2383355	-	protein_coding	CPF_RS10405	CPF_2139	.	WP_011590982. phenylalanine--	1.995384	6.611540	2.306540	6.497856	-1.155938	6.026523	6.131961	7.128627	5.922897	6.131961	7.128627	5.922897	54.255386	182.858492	52.413236	2734	8809	3531
gene2097	NC_008261.1	2383379	2384398	-	protein_coding	CPF_RS10410	CPF_2140	.	WP_003451091. phenylalanine--	2.092466	7.000616	2.488018	6.864534	-1.189036	6.362122	6.488244	7.553449	6.238452	6.488244	7.553449	6.238452	70.580680	236.564695	65.550555	1523	4880	1891
gene2098	NC_008261.1	2385010	2385783	-	protein_coding	CPF_RS10415	CPF_2141	.	WP_011590983. RNA methyltran	-1.063475	6.196738	-1.196139	6.280368	1.124746	6.325521	6.241290	6.152504	6.410889	6.241290	6.152504	6.410889	59.912047	100.936074	74.324433	981	1580	1627
gene2099	NC_008261.1	2385809	2386474	-	protein_coding	CPF_RS10420	CPF_2142	.	WP_003451483. TrkA family pota	-1.179661	6.432542	-1.187776	6.437395	1.006879	6.557776	6.552833	6.314460	6.562724	6.552833	6.314460	6.562724	73.389245	110.176749	83.510197	1034	1484	1573
gene2100	NC_008261.1	2386497	2387837	-	protein_coding	CPF_RS10425	CPF_2143	.	WP_003451277. potassium uptak	1.275890	5.179235	1.067135	5.310890	1.195622	5.133728	5.006465	5.357968	5.264226	5.006465	5.357968	5.264226	24.463399	58.737746	33.063877	694	1593	1254
gene2101	NC_008261.1	2388066	2388425	-	protein_coding	CPF_RS10430	CPF_2144	.	WP_003451332. 50S ribosomal p	2.168793	10.330645	1.869136	10.443247	1.160319	9.893961	9.787282	10.904175	10.001803	9.787282	10.904175	10.001803	779.824603	2352.938061	1093.930490	5939	17131	11138
gene2102	NC_008261.1	2388455	2388652	-	protein_coding	CPF_RS10435	CPF_2145	.	WP_003451643. 50S ribosomal p	3.461903	9.453875	2.417237	9.734524	1.432173	8.855750	8.600437	10.392002	9.118643	8.600437	10.392002	9.118643	326.115632	1500.857854	558.581526	1366	6010	3128
gene2103	NC_008261.1	2388674	2389195	-	protein_coding	CPF_RS10440	CPF_2146	.	WP_011590984. translation initia	3.226813	10.831078	2.523163	11.021231	1.278876	10.194834	10.018939	11.709049	10.373816	10.018939	11.709049	10.373816	879.929358	3837.835361	1396.903331	9717	40516	20623
gene2104	NC_008261.1	2389476	2390354	-	protein_coding	CPF_RS10445	CPF_2147	.	WP_011590985. putative sporula	-1.086633	0.456128	1.136504	0.293650	-1.234963	0.334758	0.519981	0.400116	0.215514	0.519981	0.400116	0.215514	0.322662	0.450020	0.120675	6	8	3
gene2105	NC_008261.1	2390489	2391184	+	protein_coding	CPF_RS10450	CPF_2148	.	WP_011590986. hypothetical pro	1.340327	1.376521	1.426156	1.323323	-1.064036	1.135695	1.181351	1.603936	1.091804	1.181351	1.603936	1.091804	0.950834	2.983804	1.016028	14	42	20
gene2106	NC_008261.1	2391439	2392419	+	protein_coding	CPF_RS10455	CPF_2149	.	WP_011590987. ATPase	-2.343023	5.844090	-2.759460	5.949396	1.177735	6.607414	6.490460	5.262089	6.726475	6.490460	5.262089	6.726475	70.591876	54.687870	95.080356	1465	1085	2638
gene2107	NC_008261.1	2392552	2393106	-	protein_coding	CPF_RS10460	CPF_2150	.	WP_011590988. NUDIX hydrolase	-1.387285	5.884127	-1.422314	5.901382	1.025250	6.142957	6.124995	5.652731	6.160971	6.124995	5.652731	6.160971	53.828243	72.787928	61.987607	632	817	973
gene2108	NC_008261.1	2393271	2393825	-	protein_coding	CPF_RS10465	CPF_2151	.	WP_003451404. hypothetical pro	-1.968920	0.801938	-1.806304	0.766218	-1.090027	1.364217	1.427816	0.450411	1.303452	1.427816	0.450411	1.303452	1.277569	0.534550	1.274154	15	6	20
gene2114	NC_008261.1	2394983	2395942	-	protein_coding	CPF_RS10495	CPF_2157	.	WP_003454848. redox-regulated	-1.634543	9.046855	-1.227191	8.845799	-1.331938	9.199152	9.408239	8.699352	8.994711	9.408239	8.699352	8.994711	586.591839	497.188819	504.585014	11913	9653	13700
gene2115	NC_008261.1	2395958	2396695	-	protein_coding	CPF_RS10500	CPF_2158	.	WP_011590989. class I SAM-dep	-1.221416	5.159787	1.392918	4.772489	-1.701331	4.907801	5.306081	5.017527	4.539417	5.306081	5.017527	4.539417	29.783978	45.090908	19.212020	465	673	401
gene2116	NC_008261.1	2396747	2397019	-	protein_coding	CPF_RS10505	CPF_2159	.	WP_011590990. small acid-solub	-1.152956	0.664626	-1.454333	0.795384	1.261395	0.927686	0.775178	0.569840	1.110198	0.775178	0.569840	1.110198	0.519451	0.724482	1.036125	3	4	8
gene2117	NC_008261.1	2397221	2398198	+	protein_coding	CPF_RS10510	CPF_2160	.	WP_003468781. aspartate-semia	1.442767	8.154397	1.665651	8.046647	-1.154484	7.789951	7.894264	8.423103	7.687017	7.894264	8.423103	7.687017	198.891898	395.718869	198.589260	4115	7827	5493
gene2118	NC_008261.1	2398223	2399098	+	protein_coding	CPF_RS10515	CPF_2161	.	WP_011590991. 4-hydroxy-tetr	-1.352983	8.323505	-1.055146	8.146918	-1.282271	8.363159	8.544433	8.108289	8.185731	8.544433	8.108289	8.185731	314.324282	324.954387	286.817807	5825	5757	7106
gene2119	NC_008261.1	2399171	2399935	+	protein_coding	CPF_RS10520	CPF_2162	.	WP_003455184. 4-hydroxy-tetr	-1.467899	7.892997	-1.249905	7.780221	-1.174408	8.057926	8.174728	7.620975	7.942794	8.174728	7.620975	7.942794	247.843387	248.586835	240.294260	4011	3846	5199
gene2120	NC_008261.1	2400229	2401371	-	protein_coding	CPF_RS10525	CPF_2163	.	WP_011590992. aminotransferas	-1.036334	5.238418	1.000000	5.212737	-1.036334	5.238418	5.264226	5.212737	5.212737	5.264226	5.212737	5.212737	28.866578	52.993199	31.676613	698	1225	1024
gene2121	NC_008261.1	2401371	2401907	-	protein_coding	CPF_RS10530	CPF_2164	.	WP_003451437. cob(I)alamin ade	-1.157534	5.750409	-1.082644	5.702842	-1.069172	5.808456	5.856904	5.645850	5.760409	5.856904	5.645850	5.760409	43.749008	72.557484	46.156062	497	788	701
gene2122	NC_008261.1	2402101	2402811	+	protein_coding	CPF_RS10535	CPF_2165	.	WP_003454957. 2,3,4,5-tetrahyd	3.332450	8.578937	4.275429	8.377724	-1.282969	7.572598	7.754474	9.491057	7.394988	7.754474	9.491057	7.394988	176.182343	809.772225	159.482992	2650	11644	3207
gene2123	NC_008261.1	2402861	2403517	-	protein_coding	CPF_RS10540	CPF_2166	.	WP_003454917. single-stranded	-1.594333	8.143067	-1.964880	8.286449	1.232415	8.635921	8.486493	7.813540	8.787981	8.486493	7.813540	8.787981	299.664809	273.269499	432.365937	4165	3631	8034
gene2124	NC_008261.1	2403633	2404409	-	protein_coding	CPF_RS10545	CPF_2167	.	WP_003455022. polysaccharide c	-1.236836	0.856310	-1.088718	0.775497	-1.136048	0.926687	1.023256	0.716602	0.839232	1.023256	0.716602	0.839232	0.790876	0.954554	0.682583	13	15	15
gene2125	NC_008261.1	2404517	2405047	+	protein_coding	CPF_RS10550	CPF_2168	.	WP_003451559. DUF4364 domai	2.250032	5.955309	2.338886	5.924413	-1.039490	5.370987	5.398998	6.568943	5.343122	5.398998	6.568943	5.343122	32.403578	133.438745	34.558736	364	1433	519
gene2126	NC_008261.1	2405113	2406219	+	protein_coding	CPF_RS10555	CPF_2169	.	WP_003454728. AI-2E family trar	2.033301	6.314882	2.266972	6.229210	-1.114922	5.744677	5.823685	6.847508	5.666742	5.823685	6.847508	5.666742	42.743744	154.099686	43.183127	1001	3450	1352
gene2127	NC_008261.1	2406391	2407812	+	protein_coding	CPF_RS10560	CPF_2170	.	WP_011590993. LytR family trans	-1.444182	4.531768	-1.224955	4.418332	-1.178967	4.684376	4.804643	4.274391	4.567120	4.804643	4.274391	4.567120	21.208365	27.991525	19.643212	638	805	790
gene2128	NC_008261.1	2407867	2408508	-	protein_coding	CPF_RS10565	CPF_2171	.	WP_003454985. hemolysin D	-2.313663	2.736489	-2.453599	2.770297	1.060483	3.449778	3.407678	2.197499	3.492399	3.407678	2.197499	3.492399	7.215678	5.160242	8.811908	98	67	160
gene2129	NC_008261.1	2408634	2409128	+	protein_coding	CPF_RS10570	CPF_2172	.	WP_011590994. cupin	-1.523930	5.329704	-1.403010	5.273071	-1.086186	5.582305	5.642259	5.034462	5.522988	5.642259	5.034462	5.522988	38.198024	45.550162	39.000705	400	456	546
gene2130	NC_008261.1	2409240	2410799	-	protein_coding	CPF_RS10575	CPF_2173	.	WP_011590995. ClC family H(+)/(	1.431521	4.691140	1.173375	4.840340	1.220003	4.580695	4.439498	4.957046	4.726382	4.439498	4.957046	4.726382	16.302109	43.994172	22.007953	538	1388	971
gene2131	NC_008261.1	2410910	2412235	-	protein_coding	CPF_RS10580	CPF_2174	.	WP_011590996. bifunctional folyl	1.468162	3.009937	1.506382	2.989546	-1.026033	2.727050	2.745651	3.299662	2.708575	2.745651	3.299662	2.708575	4.277833	13.051331	5.039684	120	350	189
gene2132	NC_008261.1	2412522	2415164	-	protein_coding	CPF_RS10585	CPF_2175	.	WP_011590997. valine--tRNA lig	1.773323	7.797273	2.274816	7.605495	-1.282799	7.213104	7.394988	8.221443	7.035693	7.394988	8.221443	7.035693	136.069080	349.357822	120.508097	7608	18674	9008

gene2134	NC_008261.1	2416377	2417552	-	protein_coding	CPF_RS10595	CPF_2177	.	WP_011590998.	transglutaminase	2.926172	4.462273	2.585628	4.567116	1.131706	3.842695	3.754482	5.303496	3.932981	3.754482	5.303496	3.932981	9.566559	56.089141	12.266986	238	1334	408
gene2135	NC_008261.1	2417558	2418196	-	protein_coding	CPF_RS10600	CPF_2178	.	WP_011590999.	regulatory prote	2.222295	4.765155	1.878238	4.900130	1.183181	4.343461	4.223820	5.375870	4.466490	4.223820	5.375870	4.466490	13.315508	59.041035	18.204554	180	763	329
gene2136	NC_008261.1	2418202	2419446	-	protein_coding	CPF_RS10605	CPF_2179	.	WP_003451379.	insulinase family	1.924034	5.072854	2.312443	4.925146	-1.201873	4.488103	4.622705	5.566839	4.357421	4.622705	5.566839	4.357421	18.680215	68.231370	16.699097	492	1718	588
gene2137	NC_008261.1	2419541	2420212	+	protein_coding	CPF_RS10610	CPF_2180	.	WP_003454882.	DNA-binding res	-1.850002	6.346498	-1.303818	6.106604	-1.418911	6.548503	6.805757	5.918230	6.300973	6.805757	5.918230	6.300973	89.123750	88.075508	68.716166	1267	1197	1306
gene2138	NC_008261.1	2420212	2421735	+	protein_coding	CPF_RS10615	CPF_2181	.	WP_003468780.	sensor histidine	-1.340051	5.414988	-1.062720	5.251656	-1.260963	5.460423	5.630247	5.207959	5.295721	5.630247	5.207959	5.295721	38.057977	52.560602	34.012145	1227	1620	1466
gene2139	NC_008261.1	2421988	2422734	-	protein_coding	CPF_RS10620	CPF_2182	.	WP_003468803.	NAD(P)-depend	1.891580	3.063611	1.819314	3.096070	1.039722	2.666078	2.638127	3.557719	2.694325	2.638127	3.557719	2.694325	3.923351	16.349581	5.017302	62	247	106
gene2140	NC_008261.1	2422840	2423802	-	protein_coding	CPF_RS10625	CPF_2183	.	WP_011591000.	membrane dipep	2.149153	2.584841	2.478303	2.454499	-1.153153	1.985765	2.091216	3.194984	1.885632	2.091216	3.194984	1.885632	2.405226	12.014891	2.386558	49	234	65
gene2141	NC_008261.1	2424050	2425282	-	protein_coding	CPF_RS10630	CPF_2184	.	WP_003468731.	serine hydroxym	3.770174	8.476718	4.077398	8.413230	-1.081488	7.516567	7.573288	9.487919	7.460270	7.573288	9.487919	7.460270	155.189929	808.859522	169.591178	4048	20170	5914
gene2142	NC_008261.1	2425462	2426232	+	protein_coding	CPF_RS10635	CPF_2185	.	WP_003454759.	threonine/serin	-1.180551	6.334666	-1.256159	6.378455	1.064044	6.500152	6.455527	6.216067	6.545085	6.455527	6.216067	6.545085	68.973816	104.535429	82.868369	1125	1630	1807
gene2143	NC_008261.1	2426233	2426754	+	protein_coding	CPF_RS10640	CPF_2186	.	WP_003451569.	threonine/serin	-1.488724	6.335611	-1.518573	6.349282	1.020050	6.643452	6.629148	6.055071	6.657788	6.629148	6.055071	6.657788	77.425090	95.765909	90.291041	855	1011	1333
gene2144	NC_008261.1	2426772	2427044	-	protein_coding	CPF_RS10645	CPF_2187	.	WP_003451077.	transcriptional r	1.996495	5.498630	3.205277	5.111109	-1.605452	4.668504	5.022467	6.019936	4.339487	5.022467	6.019936	4.339487	24.760505	93.458183	16.578008	143	516	128
gene2145	NC_008261.1	2427330	2429123	-	protein_coding	CPF_RS10650	CPF_2188	.	WP_003451272.	aspartate--tRN	2.389914	8.915216	2.509586	8.877318	-1.050074	8.273541	8.308861	9.565820	8.238371	8.308861	9.565820	8.238371	270.393147	837.190186	298.550548	10262	30375	15148
gene2146	NC_008261.1	2429144	2430391	-	protein_coding	CPF_RS10655	CPF_2189	.	WP_003478447.	histidine--tRNA	1.490298	7.868650	1.773003	7.737596	-1.189697	7.459762	7.586111	8.161712	7.335518	7.586111	8.161712	7.335518	156.354797	337.206770	150.355568	4128	8511	5307
gene2147	NC_008261.1	2430423	2431865	-	protein_coding	CPF_RS10660	CPF_2190	.	WP_011591001.	coproporphyrinc	1.017053	6.987735	1.057994	6.959158	-1.040255	6.947021	6.975548	6.999943	6.918611	6.975548	6.999943	6.918611	102.565864	166.772829	110.287857	3131	4867	4501
gene2148	NC_008261.1	2431855	2432475	-	protein_coding	CPF_RS10665	CPF_2191	.	WP_011591002.	MBL fold hydrola	-1.028129	7.862691	1.138987	7.748261	-1.171026	7.768006	7.882727	7.842706	7.654954	7.882727	7.842706	7.654954	197.453338	277.725116	193.756091	2594	3488	3403
gene2149	NC_008261.1	2432635	2433084	-	protein_coding	CPF_RS10670	CPF_2192	.	WP_003451157.	D-aminoacyl-tR	1.689170	6.698589	1.970327	6.580033	-1.166446	6.219046	6.331097	7.087412	6.108977	6.331097	7.087412	6.108977	64.182230	176.027402	59.872511	611	1602	762
gene2150	NC_008261.1	2433099	2435279	-	protein_coding	CPF_RS10675	CPF_2193	.	WP_011591003.	bifunctional (p)pp	1.254416	6.230286	1.533379	6.079768	-1.222384	5.922303	6.068923	6.395939	5.779226	6.068923	6.395939	5.779226	51.604769	116.416641	46.803263	2381	5135	2887
gene2151	NC_008261.1	2435431	2435949	-	protein_coding	CPF_RS10680	CPF_2194	.	WP_003451591.	adenine phospho	1.766351	6.997444	1.636848	7.055445	1.079117	6.653781	6.599082	7.419854	6.708933	6.599082	7.419854	6.708933	75.777815	218.838595	93.878658	832	2297	1378
gene2152	NC_008261.1	2435993	2436847	-	protein_coding	CPF_RS10685	CPF_2195	.	WP_003451668.	DHH family phos	3.547782	2.840373	3.107381	2.968656	1.141727	2.163682	2.070185	3.897102	2.261403	2.070185	3.897102	2.261403	2.377324	21.050652	3.473747	43	364	84
gene2153	NC_008261.1	2437047	2437916	-	protein_coding	CPF_RS10690	CPF_2196	.	WP_011591004.	protein transloc	1.168571	7.885615	1.059828	7.956763	1.102604	7.844184	7.774043	7.998788	7.914958	7.774043	7.998788	7.914958	180.821544	300.994808	234.946365	3328	5296	5781
gene2154	NC_008261.1	2437909	2439168	-	protein_coding	CPF_RS10695	CPF_2197	.	WP_003451081.	protein transloc	1.162127	8.472186	1.087154	8.520771	1.068962	8.412463	8.364495	8.581263	8.460706	8.364495	8.581263	8.460706	280.168866	462.358402	351.725746	7468	11782	12534
gene2155	NC_008261.1	2439266	2440639	-	protein_coding	CPF_RS10700	CPF_2198	.	WP_011591005.	thioether cross	2.308944	8.439624	2.475468	8.385496	-1.072121	7.807170	7.857566	9.064799	7.757098	7.857566	9.064799	7.757098	192.864577	626.170778	212.558428	5606	17400	8260
gene2156	NC_008261.1	2440709	2440849	-	protein_coding	CPF_RS10705	CPF_2199	.	WP_003451489.	six-cysteine pep	2.964326	9.757114	3.708445	9.580461	-1.251025	8.841667	9.004698	10.572402	8.681588	9.004698	10.572402	8.681588	432.135468	1701.850706	401.473823	1289	4853	1601
gene2157	NC_008261.1	2441045	2441314	-	protein_coding	CPF_RS10710	CPF_2200	.	WP_003451165.	preprotein trans	1.267538	8.986132	1.633279	8.797773	-1.288544	8.631936	8.816744	9.158773	8.451002	8.816744	9.158773	8.451002	384.112966	661.659387	350.827771	2194	3613	2679
gene2158	NC_008261.1	2441353	2442495	-	protein_coding	CPF_RS10715	CPF_2201	.	WP_003451459.	tRNA guanosine	2.206621	7.597336	2.180615	7.606549	1.011926	7.056385	7.047838	8.189677	7.064942	7.047838	8.189677	7.064942	108.518482	343.309411	122.623139	2624	7936	3964
gene2159	NC_008261.1	2442519	2443544	-	protein_coding	CPF_RS10720	CPF_2202	.	WP_003461896.	tRNA preQ1(34)	1.379249	6.477739	1.374704	6.480206	1.003306	6.252329	6.249949	6.713831	6.254710	6.249949	6.713831	6.254710	60.630987	142.795516	65.925377	1316	2963	1913
gene2160	NC_008261.1	2443566	2444606	-	protein_coding	CPF_RS10725	CPF_2203	.	WP_003451099.	Holliday junction	1.068038	6.219203	1.155155	6.161944	-1.081567	6.115079	6.171903	6.266866	6.058779	6.171903	6.266866	6.058779	56.306310	107.251528	57.435168	1240	2258	1691
gene2161	NC_008261.1	2444620	2445225	-	protein_coding	CPF_RS10730	CPF_2204	.	WP_003481965.	Holliday junction	1.126034	5.584513	1.336609	5.457494	-1.187006	5.374458	5.499544	5.670794	5.252216	5.499544	5.670794	5.252216	34.789512	73.679286	32.790550	446	903	562
gene2162	NC_008261.1	2445454	2446047	-	protein_coding	CPF_RS10735	CPF_2205	.	WP_003451265.	hypothetical pro	1.194147	1.238144	1.067886	1.324506	1.118234	1.194646	1.116752	1.372732	1.277974	1.116752	1.372732	1.277974	0.875371	2.247541	1.250023	11	27	21
gene2163	NC_008261.1	2446345	2449890	-	protein_coding	CPF_RS10740	CPF_2206	.	WP_011591006.	2',3'-cyclic-nuc	1.051495	4.816330	1.065004	4.807043	-1.012847	4.771028	4.780245	4.852687	4.761829	4.780245	4.852687	4.761829	20.915600	41.079419	22.445112	1569	2946	2251
gene2164	NC_008261.1	2450140	2453787	-	protein_coding	CPF_RS10745	CPF_2207	.	WP_011591007.	2',3'-cyclic-nuc	-20.870456	8.102583	-27.223467	8.248009	1.304402	10.775455	10.585466	6.202075	10.968854	10.585466	6.202075	10.968854	1324.662955	103.500243	1981.160110	102229	7636	204404
gene2165	NC_008261.1	2454036	2455034	-	protein_coding	CPF_RS10750	CPF_2208	.	WP_011591008.	protein-tyrosine	-3.354109	6.351602	-3.910492	6.447414	1.165881	7.394158	7.284276	5.538346	7.505697	7.284276	5.538346	7.505697	127.520319	66.818783	174.523747	2695	1350	4931
gene2166	NC_008261.1	2455509	2455943	-	protein_coding	CPF_RS10755	CPF_2209	.	WP_003454969.	bacitracin resist	-1.845467	5.686164	1.434993	4.994011	-2.648232	5.397267	6.145309	5.261323	4.740280	6.145309	5.261323	4.740280	54.876731	54.674661	22.108774	505	481	272
gene2167	NC_008261.1	2456370	2457107	-	protein_coding	CPF_RS10760	CPF_2210	.	WP_003451771.	YebC/PmpR fam	1.538157	7.485315	2.063813	7.260915	-1.341744	6.965873	7.181154	7.802357	6.757045	7.181154	7.802357	6.757045	120.480994	271.416448	97.114125	1881	4051	2027
gene2168	NC_008261.1	2457233	2457886	-	protein_coding	CPF_RS10765	CPF_2211	.	WP_003454954.	YigZ family prote	-1.228057	5.536034	1.222039	5.243188	-1.500734	5.385416	5.686206	5.389828	5.100537	5.686206	5.389828	5.100537	39.319434	59.803832	29.356691	544	791	543
gene2169	NC_008261.1	2457892	2459298	-	protein_coding	CPF_RS10770	CPF_2212	.	WP_003454993.	PLP-dependent	1.032927	3.453541	1.359937	3.247657	-1.316586	3.225755	3.430251	3.476990	3.033450	3.430251	3.476990	3.033450	7.391196	15.462826	6.408127	220	440	255
gene2170	NC_008261.1	2459313	2460215	-	protein_coding	CPF_RS10775	CPF_2213	.	WP_011591009.	nucleotidyltrans	1.286888	2.654881	1.420050	2.577697	-1.103477	2.407089	2.479165	2.843051	2.337109	2.479165	2.843051	2.337109	3.507302	8.980208	3.719811	67	164	95
gene2171	NC_008261.1	2460257	2460730	-	protein_coding	CPF_RS10780	CPF_2214	.	WP_011591010.	hypothetical pro	1.566070	4.048142	1.575662	4.043369	-1.006125	3.733072	3.737479	4.384628	3.728670	3.737479	4.384628	3.728670	9.473956	29.938762	10.517821	95	287	141
gene2172	NC_008261.1	2460862	2462655	-	protein_coding	CPF_RS10785	CPF_2215	.	WP_003451503.	GTPase HflX	2.971319	6.262484	2.519885	6.395780	1.179148	5.643630	5.526009	7.097112	5.763754	5.526009	7.097112	5.763754	35.465716	176.698808	46.335644	1346	6411	2351
gene2173	NC_008261.1	2462762	2463286	+	protein_coding	CPF_RS10790	CPF_2216	.	WP_003451102.	hypoxanthine ph	2.620015	8.755665	3.143625	8.612220	-1.199850	7.955888	8.088401	9.477976	7.825546	8.088401	9.477976	7.825546	236.620390	808.087270	222.114219	2628	8580	3298
gene2174	NC_008261.1	2463320	2464009	-	protein_coding	CPF_RS10795	CPF_2217	.	WP_011591011.	sporulation prot	-1.105119	1.178448	-1.151612	1.206087	1.042071	1.282134	1.252752	1.108551	1.312205	1.252752	1.108551	1.312205	1.027610	1.648197	1.281079	15	23	25
gene2175	NC_008261.1	2464146	2466638	+	protein_coding	CPF_RS10800	CPF_2218	.	WP_011591012.	penicillin-binding	1.024461	7.359614	1.144314	7.279176	-1.116991	7.261955	7.342202	7.377068	7.182584	7.342202	7.377068	7.182584	132.272725	213.432540	132.269023	6976	10761	9326
gene2176	NC_008261.1	2466932	2468332	+	protein_coding	CPF_RS10805	CPF_2219	.	WP_003451357.	alanine:cation sy	7.717866	6.139441	5.389169	6.459723	1.432107	5.092313	4.839829	7.788031	5.357968	4.839829	7.788031	5.357968	21.796185	269.323096	34.903506	646	7631	1383
gene2177	NC_008261.1	2468369	2468761	-	protein_coding	CPF_RS10810	CPF_2220	.	WP_003470926.	hypothetical pro	2.438188	1.888805	2.039836	2.060709	1.195286	1.475395	1.352317	2.638127	1.609673	1.352317	2.638127	1.609673	1.202800	7.548992	1.799378	10	60	20
gene2178	NC_008261.1	2468809	2469885	-	protein_coding	CPF_RS10815	CPF_2221	.	WP_041702716.	N-acetyltransfe	1.608278	2.946131	2.147183	2.701883	-1.335082	2.405765	2.623244	3.308761	2.206316	2.623244	3.308761	2.206316	3.906253	13.176391	3.315818	89	287	101
gene2179	NC_008261.1	2470211	2470750	+	protein_coding	CPF_RS10820	CPF_2222	.	WP_003451138.	cupin domain-co	1.314578	5.130080	-1.715854	5.707363	2.255624	5.492080	4.936572	5.331172	6.110099	4.936572	5.331172	6.110099	23.197342	57.320617	59.911797	265	626	915
gene2180	NC_008261.1	2470782	2471831	+	protein_coding	CPF_RS10825	CPF_2223	.	WP_004457807.	spermidine/putr	-1.074182	5.785742	-2.520476	6.366372	2.346415	6.423425	5.837591	5.734353	7.068050	5.837591	5.734353	7.068050	43.173317	77.371060	123.011407	959	1643	3653
gene2181	NC_008261.1	2471834	2472679	+	protein_coding	CPF_RS10830	CPF_2224	.	WP_003461855.	ABC transporter	1.072147	5.730540	-2.245658	6.337770	2.407675	6.282438	5.680509	5.781011	6.948149	5.680509	5.781011	6.948149	39.168213	80.247305	112.551427	701	1373	2693
gene2182	NC_008261.1	2472673	2473473	+	protein_coding	CPF_RS10835	CPF_2225	.	WP_003451178.	ABC transporter	-1.088311	5.381995	-2.323936	5.898298	2.135360	5.965579	5.443387	5.321296	6.537866	5.443387	5.321296	6.537866	33.460825	57.038715	82.589776	567	924	1871
gene2183	NC_008261.1	2473475	2474557	+	protein_coding	CPF_RS10840	CPF_2226	.	WP_003454960.	spermidine/putr	1.009801	6.834139	-1.873010	7.279796	1.891367	7.272306	6.827107	6.841177	7.746536	6.827107	6.841177	7.746536	91.179266	152.946996	209.534852	2089	3350	6418
gene2184	NC_008261.1	2474812	2475243	-	protein_coding	CPF_RS10845	CPF_2227	.	WP_003451216.	nucleoside-diph	-2.850068	5.033270	-2.921426	5.048608	1.025037	5.862964	5.845153	4.334157	5.880830	5.845153	4.334157	5.880830	43.440305	29.072357	50.744965	397	254	620
gene2185	NC_008261.1	2475428	2475697	+	protein_coding	CPF_RS10850	CPF_2228	.	WP_003461780.	acylphosphatase	-1.631094	5.274892	-1.328870	5.134770	-1.227429	5.489795	5.639604	4.933765	5.343964	5.639604	4.933765	5.343964	38.166193	43.219379	34.572054	218	236	264
gene2186	NC_008261.1	2476160	2476726	-	protein_coding	CPF_RS10855	CPF_2230	.	WP_003454815.	hypothetical pro	1.528284	2.007977	1.652579	1.941219	-1.081330	1.667840	1.725197	2.337109	1.612390	1.725197	2.337109	1.612390	1.750743	5.842814	1.808423	21	67	29
gene2187	NC_008261.1	2476905	2478176	+	protein_coding	CPF_RS10860	CPF_2231	.	WP_011591014.	D-alanyl-D-alan	2.609270	6.660478	3.716759	6.371112	-1.424444	5.743622	6.004488	7.388135	5.494090	6.004488	7.388135	5.494090	49.388289	214.421054	38.415452	1329	5516	1382
gene2188	NC_008261.1	2478231	2478875	-	protein_coding	CPF_RS10865	CPF_2232	.	WP_011591015.	pyrophosphatase	2.648847	5.309803	3.026267	5.199006	-1.142485	4.556314	4.653415	6.058779	4.461240	4.653415	6.058779	4.461240	19.201170	95.825386	18.144846	262	1250	331
gene2189	NC_008261.1	2479465	2480475	+	protein_coding	CPF_RS10870	CPF_2233	.	WP_003461748.	asparagine synth	15.975332	6.415841	14.182269	6.531505	1.126430	4.806237	4.721125	8.718900	4.892883	4.721125	8.718900	4.892883	20.058213	502.920062	24.725966	429	10283	707
gene2190	NC_008261.1	2480601	2481419	+	protein_coding	CPF_RS10875	CPF_2234	.	WP_003461934.	tetratricopeptide	3.901514	3.210646	3.642741	3.276873	1.071038	2.424453	2.375453	4.339487	2.474463	2.375453	4.339487	2.474463	3.116707	29.160402	4.101330	54	483	95
gene2191	NC_008261.1	2481531	2481908	+	protein_coding	CPF_RS10880	CPF_2235	.	WP_003451739.	CidA/LrgA family	2.982976	3.696499	2.860494	3.733687	1.042818	3.021352	2.991259	4.568012	3.051747	2.991259	4.568012	3.051747	5.252228	33.094742	6.547737	42	253	70
gene2192	NC_008261.1	2481901	2482611	+	protein_coding	CPF_RS10885	CPF_2236	.	WP_003451174.	LrgB family prote	2.020944	4.012107	2.249704	3.923371	-1.113195	3.458346	3.536564	4.551593	3.381858	3.536564	4.551593	3.381858	7.911584	32.685756	8.255122	119	470	166
gene2194	NC_008261.1	2483377	2485539	-	protein_coding	CPF_RS10895	CPF_2238	.	WP_011591016.	type IA DNA top	1.337949	3.292333	1.444983	3.232623	-1.079998	3.032990	3.089012	3.509036	2.977984	3.089012	3.509036	2.977984	5.682022	15.796170	6.080950	260	691	372
gene2195	NC_008261.1	2485767	2486924	-	protein_coding	CPF_RS10900	CPF_2239	.	WP_011591017.	amidohydrolase	-1.702774	4.785915	-1.804325	4.824329	1.059639	5.226854	5.185234	4.417347	5.268807	5.185234	4.417347	5.268807	27.472148	30.359270	33.220438	673	711	1088
gene2196	NC_008261.1	2487087	2487584	-	protein_coding	CPF_RS10905	CPF_2240	.	WP_003454922.	tryptophan-rich	1.387991	1.592696	-1.017574	1.859182	1.412384	1.603498	1.373660	1.846658	1.871792	1.373660	1.846658	1.871792	1.233957	3.772980	2.342986	13	38	33
gene2197	NC_008261.1	2487630	2489666	-	protein_coding	CPF_RS10910	CPF_2241	.	WP_003454982.	sensor histidine	1.033059	0.574419	1.019578	0.584206	1.013222	0.560832	0.551437	0.598359	0.570387	0.551437	0.598359	0.570387	0.348086	0.776764	0.399229	15	32	23
gene2198	NC_008261.1	2489656	2491542	-	protein_coding	CPF_RS10915	CPF_2242	.	WP_003455042.	sensor histidine	-1.114925	0.576513	-1.133142	0.586631	1.016339	0.671891	0.660302	0.503356	0.683684	0.660302	0.503356	0.683684	0.425856	0.602679	0.505914	17	23	27
gene2199	NC_008261.1	2491663	2492598	-	protein_coding	CPF_RS10920	CPF_2243	.	WP_003451066.	tetratricopeptide	3.026011	1.775570	2.439019	2.001716	1.240667	1.294479	1.148234	2.745651	1.459350	1.148234	2.745651	1.459350	0.909040	8.240983	1.511016	18	156	40
gene2200	NC_008261.1	2492719	2493639	-	protein_coding	CPF_RS10925	CPF_2244	.	WP_003473705.	alpha/beta hydro	-1.536129	3.727060	-1.317994	3.623955	-1.165503	3.937524	4.049550	3.430251	3.828598	4.049550	3.430251	3.828598	11.907332	14.656602	11.402021	232	273	297
gene2201	NC_008261.1	2493762	2494358	-	protein_coding	CPF_RS10930	CPF_2245	.	WP_003454836.	hypothetical pro	1.487766	1.566610	1.964506	1.304102	-1.320440	1.087187	1.306032	1.879180	0.905013	1.306032	1.879180	0.905013	1.108511	3.892726	0.769935	14	47	13
gene2202	NC_008261.1	2494499	2494584	-	tRNA	CPF_RS10935	CPF_2246	.	.	tRNA-Leu	2.052569	2.147696	1.188382	2.600686	1.727196	2.047342	1.690734	2.728164	2.479165	1.690734	2.728164	2.479165	1.648955	8.049332	4.111370	3	14	10
gene2203	NC_008261.1	2494644	2495759	-	protein_coding	CPF_RS10940	CPF_2247	.	WP_003455006.	glycosyl hydrolas	1.550828	4.001128	1.449213	4.053687	1.070117	3.745675	3.697109	4.330147	3.794878	3.697109	4.330147	3.794878	9.064330	28.976360	11.088910	214	654	350
gene2204	NC_008261.1	2495811	2496356	-	protein_coding	CPF_RS10945	CPF_2248	.	WP_003455079.	lytic transglycos	-1.588263	6.610795	-1.557990	6.597583	-1.019431	6.939042	6.952938	6.285488	6.925174	6.952938	6.285488	6.925174	100.513798	108.219505	111.318731	1161	1195	1719
gene2205	NC_008261.1	2496366	2496965	-	protein_coding	CPF_RS10950	CPF_2249	.	WP_011591019.	dephospho-CoA	-1.307845	6.735899	-1.637327	6.891585	1.251927	7.092500	6.932277	6.545085	7.256427	6.932277	6.545085	7.256427	98.636848	131.031633	139.722169	1252	1590	2371
gene2206	NC_008261.1	2496982	2499582	-	protein_coding	CPF_RS10955	CPF_2250	.	WP_011591020.	DNA polymerase	-1.143167	6.374212	-1.248900	6.436756	1.092491	6.534960	6.471461	6.278424	6.599082	6.471461	6.278424	6.599082	69.678351	107.959732	86.334976	3834	5679	6351
gene2207	NC_008261.1	2499939	2500856	-	protein_coding	CPF_RS10960	CPF_2251	.	WP_003451262.	glutaminase 2	-1.014216	6.329791	1.027137	6.300273	-1.041738	6.310416	6.339981	6.319617	6.280989	6.339981	6.319617	6.280989	64.725990	110.633853	67.480211	1257	2054	1752
gene2209	NC_008261.1	2501693	2502928	+	protein_coding	CPF_RS10970	CPF_2253	.	WP_003455100.	aminopeptidase	-2.709922	10.397557	-1.707529	10.081840	-1.587043	10.803215	11.141522	9.703270	10.475180	11.141522	9.703270	10.475180	1845.903299	925.550427	1477.474698	48266	23136	51648
gene2210	NC_008261.1	2503141	2504190	-	protein_coding	CPF_RS10975	CPF_2254	.	WP_003455008.	hypothetical pro	4.305298	4.819009	3.150617	5.091102	1.366494	4.098724	3.879669	5.985782	4.330147	3.879669	5.985782	4.330147	10.444431	91.781007	16.500298	232	1949	490
gene2211	NC_008261.1	2504207	2505217	-	protein_coding	CPF_RS10980	CPF_2255	.	WP_003454874.	hypothetical pro	3.771944	5.890178	2.891782	6.111379	1.304367	5.198008	5.009866	6.925174	5.393215	5.009866	6.925174	5.393215	24.546764	160.271229	35.707513	525	3277	1021
gene2212	NC_008261.1	2505734	2506080	-	RNase_P_RNA	CPF_RS14695	.	rnpB	.	RNase P RNA c	1.114748	15.475793	1.000000	15.554350	1.114748	15.475793	15.397632	15.554350	15.554350	15.397632	15.554350	15.554350	49985.154383	44439.323933	49030.850332	366931	311865	481187
gene2213	NC_008261.1	2506128	2506385	-	protein_coding	CPF_RS10985	CPF_2257	.	WP_003478491.	hypothetical pro	4.300376	2.251513	4.002479	2.331456	1.074428	1.483907	1.433026	3.537488	1.536595	1.433026	3.537488	1.536595	1.282521	16.098665	1.644548	7	84	12
gene2214	NC_008261.1	2506548	2507237	-	protein_coding	CPF_RS10990	CPF_2258	.	WP_003455144.	radical SAM prot	9.219484	6.173082	7.634046	6.346606	1.207680	4.909542	4.775309	7.979995	5.047547	4.775309	7.979995	5.047547	20.826227	297.821968	28.234982	304	4156	551
gene2215	NC_008261.1	2507255	2507812	-	protein_coding	CPF_RS10995	CPF_2259	.	WP_003451567.	ECF transporter	4.770089	4.795354	4.884751	4.773677	-1.024038	3.781828	3.799001	6.053017	3.764732	3.799001	6.053017	3.764732	9.911463	95.613128	10.772084	117	1079	170
gene2216	NC_008261.1	2507831	2508811	-	protein_coding	CPF_RS11000	CPF_2260	.	WP_003454942.	nucleoside hydr	6.156132	6.698618	5.832681	6.745743	1.055455	5.553488	5.514692	8.136716	5.592557	5.514692	8.136716	5.592557	35.079103	331.605068	40.439787	728	6579	1122
gene2217	NC_008261.1	2509325	2510113	-	protein_coding	CPF_RS11005	CPF_2261	.	WP_003455195.	Nif3-like dinucle	1.417244	5.826177	1.308351	5.886082	1.083229	5.637435	5.580061	6.083149	5.695399	5.580061	6.083149	5.695399	37.085126	97.450410	44.186024	619	1555	986
gene2218	NC_008261.1	2510113	2510802	-	protein_coding	CPF_RS11010	CPF_2262	.	WP_003455116.	SAM-dependent	2.023348	4.391878	2.209566	4.320006	-1.092034	3.848798	3.912831	4.929576	3.785813	3.912831	4.929576	3.785813	10.687143	42.996434	10.966037	156	600	214
gene2219	NC_008261.1	2510816	2511703	-	protein_coding	CPF_RS11015	CPF_2263	.	WP_003454720.	membrane prote	1.434796	3.526719	1.630786	3.425843	-1.136598	3.182197	3.275898	3.796744	3.091176	3.275898	3.796744	3.091176	6.654005	19.377447	6.649493	125	348	167
gene2220	NC_008261.1	2511938	2513038	-	protein_coding	CPF_RS11020	CPF_2264	.	WP_011591021.	RNA polymerase	-1.427553	8.788454	-1.256814	8.698755	-1.135850	8.956619	9.048976	8.535432	8.865204	9.048976	8.535432	8.865204	453.509166	444.743634	451.558377	10563	9903	14061
gene2221	NC_008261.1	2513065	2514852	-	protein_coding	CPF_RS11025	CPF_2265	.	WP_003455114.	DNA primase	-1.194467	7.525679	1.088029	7.337476	-1.299615	7.463518	7.654954	7.398587	7.276870	7.654954	7.398587	7.276870	164.229073	216.809760	141.885954	6212	7840	7175
gene2222	NC_008261.1	2515021	2516049	-	protein_coding	CPF_RS11030	CPF_2266	.	WP_011591022.	deoxyguanosine	-1.574830	6.409466	-1.181699	6.209498	-1.332682	6.534981	6.745430	6.090234	6.331097	6.745430	6.090234	6.331097	85.720042	98.122961	70.097061	1866	2042	2040
gene2223	NC_008261.1	2516254	2517333	+	protein_coding	CPF_RS11035	CPF_2267	.	WP_003451193.	CotS family spor	-1.789661	1.770019	-1.433136	1.638439	-1.248773	2.072532	2.238974	1.399288	1.918463	2.238974	1.399288	1.918463	2.757420	2.380728	2.488140	63	52	76
gene2224	NC_008261.1	2517385	2520015	-	protein_coding	CPF_RS11040	CPF_2268	.	WP_003455045.	pyruvate, phosph	-16.142385	10.344936	-14.154288	10.266452	-1.140459	12.448931	12.544099	8.531318	12.354485	12.544099	8.531318	12.354485	5936.946436	443.171324	4978.055865	330444	23581	370421
gene2225	NC_008261.1	2520116	2520748	-	protein_coding	CPF_RS11045	CPF_2269	.	WP_003451073.	transcriptional r	-4.256801	9.485285	-3.073620	9.272438	-1.384947	10.349967	10.587548	8.497778	10.117717	10.587548	8.497778	10.117717	1327.892763	428.999809	1188.758795	17782	5492	21282
gene2226	NC_008261.1	2520953	2521579	-	protein_coding	CPF_RS11050	CPF_2270	.	WP_003451685.	DUF4342 domai	1.115596	7.969050	1.010957	8.040484	1.103505	7.961263	7.890534	8.048348	8.032627	7.890534	8.048348	8.032627	198.353293	312.920779	250.944386	2631	3968	4450
gene2227	NC_008261.1	2521585	2522325	-	protein_coding	CPF_RS11055	CPF_2271	.	WP_034833182.	DNA repair prote	-1.094826	6.319722	-1.218663	6.395769	1.113112	6.462248	6.385411	6.254710	6.540009	6.385411	6.254710	6.540009	66.918067	106.699046	92.692357	1049	1599	1733
gene2228	NC_008261.1	2522338	2523228	-	protein_coding	CPF_RS11060	CPF_2272	.	WP_011591023.	GTPase Era	1.120457	8.662917	1.091778	8.681778	1.026268	8.599946	8.581263	8.745349	8.618670	8.581263	8.745349	8.618670	321.234774	509.498094	385.919675	6055	9181	9725
gene2229	NC_008261.1	2523237	2523635	-	protein_coding	CPF_RS11065	CPF_2273	.	WP_003461827.	cytidine deamina	1.009504	8.611844	-1.165701	8.728568	1.176780	8.721655	8.605023	8.618670	8.839868	8.605023	8.618670	8.839868	327.573189	474.878913	442.814127	2765	3832	4997
gene2230	NC_008261.1	2523679	2524377	-	protein_coding	CPF_RS11070	CPF_2274	.	WP_003455106.	phosphatase PA	-1.138575	7.232776	-1.384692	7.370812	1.216163	7.466830	7.326997	7.139768	7.609333	7.326997	7.139768	7.609333	130.449122	185.262962	187.816087	1929	2619	3713
gene2231	NC_008261.1	2524405	2524911	-	protein_coding	CPF_RS11075	CPF_2275	.	WP_003455166.	endoribonucleas	-1.166534	7.973583	-1.322220	8.062207	1.133461	8.175339	8.085471	7.863243	8.266206	8.085471	7.863243	8.266206	236.536745	281.851379	304.341935	2537	2890	4364
gene2232	NC_008261.1	2524913	2526967	-	protein_coding	CPF_RS11080	CPF_2276	.	WP_003482023.	HD family phosph	-1.370829	7.371282	-1.457624	7.414096	1.063315	7.646473	7.602317	7.147268	7.690886	7.602317	7.147268	7.690886	157.290821	186.162007	198.949895	6838	7737	11563
gene2233	NC_008261.1	2527004	2528140	-	protein_coding	CPF_RS11085	CPF_2277	.	WP_003478501.	sporulation prot	1.000000	3.333699	1.013095	3.324301	-1.013095	3.324301	3.333699	3.333699	3.314930	3.333699	3.333699	3.314930	6.901345	13.437804	7.836553	166	309	252
gene2235	NC_008261.1	2528659	2529108	-	protein_coding	CPF_RS11095	CPF_2279	.	WP_003451322.	GatB/YqeY dom	2.629912	11.826040	2.356085	11.909870	1.116221	11.228116	11.149085	12.544099	11.307708	11.149085	12.544099	11.307708	1863.070437	6494.554062	2492.959352	17736	59106	31728
gene2236	NC_008261.1	2529158	2529334	-	protein_coding	CPF_RS11100	CPF_2280	.	WP_003451446.	30S ribosomal p	2.116775	10.729775	2.021424	10.764684	1.047170	10.235661	10.202467	11.284335	10.268963	10.202467	11.284335	10.268963	1017.240902	3127.103940	1307.239128	3809	11194	6544
gene2237	NC_008261.1	2529454	2529798	-	protein_coding	CPF_RS11105	CPF_2281	.	WP_011591025.	histidine triad nu	1.717254	9.366634	1.717254	9.366634	1.000000	8.984700	8.984700	9.764803	8.984700	8.984700	9.764803	8.984700	430.500038	960.397019	501.875532	3142	6701	4897
gene2238	NC_008261.1	2529867	2531171	-	protein_coding	CPF_RS11110	CPF_2282	.	WP_003478502.	tRNA (N(6)-L-th	1.042762	6.073458	-1.012476	6.112676	1.055772	6.082351	6.043328	6.103738	6.121627	6.043328	6.103738	6.121627	50.602503	98.550793	60.474000	1397	2601	2232
gene2239	NC_008261.1	2531171	2531923	-	protein_coding	CPF_RS11115	CPF_2283	.	WP_011591026.	16S rRNA (urac	1.017951	5.998602	-1.029231	6.032198	1.047707	6.019305	5.985782	6.011450	6.053017	5.985782	6.011450	6.053017	48.211690	93.113261	57.333137	768	1418	1221
gene2240	NC_008261.1	2531946	2532887	-	protein_coding	CPF_RS11120	CPF_2284	.	WP_011591027.	50S ribosomal p	1.101606	6.830954	1.174790	6.783918	-1.066434	6.714949	6.761507	6.901115	6.668712	6.761507	6.901115	6.668712	86.912670	158.468334	90.984356	1732	3019	2424
gene2241	NC_008261.1	2533113	2533469	-	protein_coding	CPF_RS11125	CPF_2285	.	WP_003451353.	GntR family tran	-1.399720	4.084391	-1.267848	4.016563	-1.104012	4.262181	4.334157	3.849019	4.191400	4.334157	3.849019	4.191400	14.565003	20.221572	14.856211	110	146	150
gene2242	NC_008261.1	2533471	2534649	-	protein_coding	CPF_RS11130	CPF_2286	.	WP_011591028.	hypothetical pro	-1.057222	3.009593	-1.242347	3.122334	1.175106	3.164255	3.050000	2.969721	3.282790	3.050000	2.969721	3.282790	5.492788	10.023384	7.677347	137	239	256
gene2243	NC_008261.1	2534665	2535486	-	protein_coding	CPF_RS11135	CPF_2287	.	WP_041702720.	hypothetical pro	1.467245	0.926442	-1.003893	1.246193	1.472956	0.928528	0.690284	1.243393	1.248998	0.690284	1.243393	1.248998	0.460049	1.985054	1.204401	8	33	28
gene2244	NC_008261.1	2535479	2536171	-	protein_coding	CPF_RS11140	CPF_2288	.	WP_003470891.	ABC transporter	1.195846	1.495264	-1.184823	1.747896	1.416866	1.603576	1.371804	1.629836	1.874507	1.371804	1.629836	1.874507	1.227794	3.068072	2.346981	18	43	46
gene2245	NC_008261.1	2536347	2537510	-	protein_coding	CPF_RS11145	CPF_2289	.	WP_003451159.	molecular chape	-3.377102	8.961103	-2.182194	8.670748	-1.547572	9.561705	9.881895	8.126110	9.251889	9.881895	8.126110	9.251889	820.850191	329.426933	611.683554	20213	7755	20137
gene2246	NC_008261.1	2537624	2539483	-	protein_coding	CPF_RS11150	CPF_2290	.	WP_003454824.	molecular chape	-5.254365	11.321070	-3.232188	11.001146	-1.625638	12.225382	12.580908	10.187392	11.879903	12.580908	10.187392	11.879903	6099.971879	1264.246578	3600.410709	240024	47557	189400
gene2247	NC_008261.1	2539540	2540166	-	protein_coding	CPF_RS11155	CPF_2291	.	WP_003461874.	nucleotide exch	-3.857051	10.235875	-1.880122	9.753125	-2.051490	10.724983	11.255837	9.308339	10.219165	11.255837	9.308339	10.219165	1975.239940	730.647939	1278.350177	26200	9265	22669
gene2248	NC_008261.1	2540197	2541216	-	protein_coding	CPF_RS11160	CPF_2292	.	WP_003470958.	HrcA family tran	-2.654425	10.087889	-1.414680	9.655234	-1.876343	10.352728	10.816638	9.408239	9.908715	10.816638	9.408239	9.908715	1511.714898	778.966820	1018.373452	32620	16069	29378
gene2249	NC_008261.1	2541413	2542546	-	protein_coding	CPF_RS11165	CPF_2293	.	WP_011591030.	coproporphyrinc	1.021876	3.659894	1.249324	3.511294	-1.222579	3.496350	3.644317	3.675538	3.354390	3.644317	3.675538	3.354390	8.670345	17.790059	8.075543	208	408	259
gene2250	NC_008261.1	2542816	2543085	-	protein_coding	CPF_RS11170	CPF_2294	.	WP_011591031.	hypothetical pro	-5.401242	4.515499	-3.970717	4.342109	-1.360269	5.666887	5.893178	3.459887	5.449286	5.893178	3.459887	5.449286	44.643941	15.200036	37.191149	255	83	284
gene2251	NC_008261.1	2543342	2545144	-	protein_coding	CPF_RS11175	CPF_2295	.	WP_003455062.	elongation facto	1.581722	6.205071	1.673357	6.162074	-1.057934	5.842366	5.883131	6.544628	5.801882	5.883131	6.544628	5.801882	44.359919	131.005580	47.869299	1692	4777	2441
gene2253	NC_008261.1	2545682	2546767	-	protein_coding	CPF_RS11185	CPF_2297	.	WP_004457777.	stage II sporulat	-1.658349	2.812341	-1.201462	2.600065	-1.380276	2.959190	3.200786	2.471038	2.735829	3.200786	2.471038	2.735829	6.224326	6.465302	5.176692	143	142	159
gene2254	NC_008261.1	2546916	2547893	-	protein_coding	CPF_RS11190	CPF_2298	.	WP_003455049.	GPR endopeptid	-1.045288	0.690499	-1.138228	0.746865	1.088913	0.782223	0.723188	0.659287	0.846077	0.723188	0.659287	0.846077	0.483334	0.859489	0.686910	10	17	19
gene2255	NC_008261.1	2548089	2548352	+	protein_coding	CPF_RS11195	CPF_2299	.	WP_003451389.	30S ribosomal p	1.862971	11.140730	1.799940	11.166548	1.035018	10.725763	10.700963	11.598568	10.750620	10.700963	11.598568	10.750620	1391.243666	3709.940813	1712.977395	7770	19808	12790
gene2256	NC_008261.1	2548448	2549494	-	protein_coding	CPF_RS11200	CPF_2300	.	WP_011591033.	DNA polymerase	1.613365	4.095259	2.428292	3.760771	-1.505110	3.457240	3.764732	4.454805	3.174863	3.764732	4.454805	3.174863	9.661692	30.744298	7.125589	214	651	211
gene2257	NC_008261.1	2549538	2551193	-	protein_coding	CPF_RS11205	CPF_2301	.	WP_011591034.	competence pro	1.318497	0.307799	1.404359	0.207857	-1.065121	0.112993	0.167322	0.566216	0.076304	0.167322	0.566216	0.076304	0.085634	0.716607	0.042703	3	24	2
gene2258	NC_008261.1	2551245	2551610	-	protein_coding	CPF_RS11210	CPF_2302	.	WP_011010735.	stage V sporulat	1.011751	0.445938	1.028740	0.433524	-1.016791	0.425409	0.437590	0.454445	0.413567	0.437590	0.454445	0.413567	0.258306	0.540392	0.289818	2	4	3
gene2259	NC_008261.1	2551666	2552685	-	protein_coding	CPF_RS11215	CPF_2303	.	WP_011591035.	stage V sporulat	1.005447	0.953963	-1.025384	0.975805	1.030970	0.971805	0.950053	0.957890	0.994055	0.950053	0.957890	0.994055	0.695148	1.357338	0.866612	15	28	25
gene2260	NC_008261.1	2552691	2553158	-	protein_coding	CPF_RS11220	CPF_2304	.	WP_003454910.	stage V sporulat	-1.098975	0.251664	-1.028942	0.212215	-1.068063	0.277251	0.328789	0.192630	0.233792	0.328789	0.192630	0.233792	0.202009	0.211307	0.151102	2	2	2
gene2261	NC_008261.1	2553296	2554051	-	protein_coding	CPF_RS11225	CPF_2305	.	WP_003454713.	RNA polymerase	-4.122825	4.134193	-4.133879	4.135704	1.002681	5.282346	5.280415	3.236781	5.284278	5.280415	3.236781	5.284278	29.137362	12.492284	33.674078	466	191	720
gene2262	NC_008261.1	2554069	2554491	-	protein_coding	CPF_RS11230	CPF_2306	.	WP_003451402.	anti-sigma F fac	-3.164786	4.393912	-3.166165	4.394173	1.000436	5.303182	5.302867	3.640760	5.303496	5.302867	3.640760	5.303496	29.725377	17.417113	34.103960	266	149	408
gene2263	NC_008261.1	2554505	2554840	-	protein_coding	CPF_RS11235	CPF_2307	.	WP_003451156.	anti-sigma F fac	-1.755728	3.428152	-1.744653	3.424093	-1.006348	3.853581	3.858148	3.046079	3.849019	3.858148	3.046079	3.849019	10.269982	10.595550	11.575464	73	72	110
gene2264	NC_008261.1	2554974	2557259	-	protein_coding	CPF_RS11240	CPF_2308	.	WP_011591036.	ATP-dependent	-1.536445	2.053280	-1.360282	1.976249	-1.129505	2.296793	2.386318	1.766721	2.210627	2.386318	1.766721	2.210627	3.163744	3.504040	3.340893	153	162	216
gene2265	NC_008261.1	2557460	2558134	-	protein_coding	CPF_RS11245	CPF_2309	.	WP_003463155.	2-deoxyribose-	-3.356225	9.175402	-5.142448	9.451157	1.532212	10.393550	10.090299	8.343459	10.705915	10.090299	8.343459	10.705915	949.602884	376.667873	1688.111469	13560	5142	32227
gene2266	NC_008261.1	2558391	2558798	+	protein_coding	CPF_RS11250	CPF_2310	.	WP_011591037.	hypothetical pro	1.279907	8.636891	1.804746	8.380031	-1.410060	8.209086	8.460706	8.816744	7.964949	8.460706	8.816744	7.964949	296.017134	539.420829	242.304800	2555	4451	2796
gene2267	NC_008261.1	2559002	2561551	-	protein_coding	CPF_RS11255	CPF_2311	.	WP_011591038.	calcium-translo	-1.778959	1.546598	-1.646056	1.503037	-1.080740	1.960150	2.016960	1.185927	1.904940	2.016960	1.185927	1.904940	2.280085	1.861493	2.440380	123	96	176
gene2268	NC_008261.1	2561796	2563445	+	protein_coding	CPF_RS11260	CPF_2312	.	WP_003451432.	cobalt-depende	1.121790	9.707290	1.362420	9.564858	-1.214505	9.483522	9.624742	9.790545	9.344374	9.624742	9.790545	9.344374	708.878935	971.626906	650.047466	24744	32423	30335
gene2269	NC_008261.1	2563488	2564129	-	protein_coding	CPF_RS11265	CPF_2313	.	WP_003454832.	HAD-IB family h	1.431641	3.391701	1.333366	3.446619	1.073704	3.193615	3.142728	3.660398	3.245325	3.142728	3.660398	3.245325	5.963979	17.714263	7.435048	81	230	135
gene2270	NC_008261.1	2564213	2564587	-	protein_coding	CPF_RS11270	CPF_2314	.	WP_003451221.	DUF1292 domai	-1.023844	12.775887	-1.055879	12.798063	1.031289	12.815102	12.792897	12.758901	12.837346	12.792897	12.758901	12.837346	6868.780186	7962.240210	6480.091430	54491	60386	68727
gene2271	NC_008261.1	2564797	2566191	-	protein_coding	CPF_RS11275	CPF_2315	.	WP_003455061.	glutamate decar	-3.219738	8.891957	-2.651004	8.763497	-1.214535	9.634121	9.775344	8.088401	9.494939	9.775344	8.088401	9.494939	774.212343	317.977895	714.734119	22848	8971	28199
gene2272	NC_008261.1	2566777	2567832	-	protein_coding	CPF_RS11280	CPF_2316	.	WP_011591039.	DUF2776 domai	-4.374753	6.930359	-3.890724	6.857394	-1.124406	7.991223	8.076252	5.947050	7.907089	8.076252	5.947050	7.907089	234.738796	89.761164	232.671617	5244	1917	6949
gene2273	NC_008261.1	2567952	2569370	-	protein_coding	CPF_RS11285	CPF_2317	.	WP_003454789.	glutamate:gamm	-13.163737	10.262554	-12.538062	10.233177	-1.049902	12.253684	12.288861	8.570364	12.218607	12.288861	8.570364	12.218607	4557.812687	460.136083	4515.081621	136821	13205	181202
gene2274	NC_008261.1	2570072	2573581	-	protein_coding	CPF_RS11290	CPF_2318	.	WP_025647475.	pyruvate:ferredo	2.932508	13.107598	2.677873	13.176932	1.095088	12.419836	12.354485	13.906620	12.485532	12.354485	13.906620	12.485532	4910.254405	13966.001319	5336.990252	364607	991400	529808
gene2275	NC_008261.1	2573692	2574117	-	protein_coding	CPF_RS11295	CPF_2319	.	WP_003451301.	flavodoxin	-1.493673	10.634782	-1.059149	10.390658	-1.410258	10.677294	10.928152	10.349288	10.432194	10.928152	10.349288	10.432194	1596.085615	1449.600557	1442.448108	14384	12489	17379
gene2276	NC_008261.1	2574324	2575397	-	protein_coding	CPF_RS11300	CPF_2320	.	WP_003451085.	undecaprenyldip	1.441033	3.874161	1.411236	3.890260	1.021114	3.634604	3.619563	4.146667	3.649707	3.619563	4.146667	3.649707	8.494526	25.275418	9.942319	193	549	302
gene2277	NC_008261.1	2575582	2575761	+	protein_coding	CPF_RS11305	CPF_2321	.	WP_003451068.	small, acid-solub	-4.052079	1.285314	-2.384445	1.083495	-1.699380	2.228490	2.643583	0.624921	1.878575	2.643583	0.624921	1.878575	3.939171	0.824098	2.357185	15	3	12
gene2278	NC_008261.1	2575882	2577660	-	protein_coding	CPF_RS11310	CPF_2322	.	WP_011591040.	single-stranded	-1.115597	4.302337	-1.172099	4.337188	1.050647	4.417464	4.381969	4.224153	4.453247	4.381969	4.224153	4.453247	15.251833	26.849207	17.947205	574	966	903
gene2279	NC_008261.1	2577672	2578883	-	protein_coding	CPF_RS11315	CPF_2323	.	WP_011591041.	insulinase family	-1.377863	3.200860	-1.299504	3.161321	-1.060299	3.397918	3.440416	2.977984	3.355944	3.440416	2.977984	3.355944	7.488326	10.117643	8.080945	192	248	277
gene2280	NC_008261.1	2579096	2579671	-	protein_coding	CPF_RS11320	CPF_2324	.	WP_004457853.	DJ-1 family prot	-1.405351	7.943329	-1.294955	7.885903	-1.085251	8.133358	8.192586	7.701656	8.074557	8.192586	7.701656	8.074557	250.793903	259.247597	261.254723	3056	3020	4256
gene2281	NC_008261.1	2579683	2582622	-	protein_coding	CPF_RS11325	CPF_2325	.	WP_004457707.	DNA topoisomer	-1.289430	5.302431	-1.406692	5.362740	1.090941	5.551398	5.488967	5.122233	5.614540	5.488967	5.122233	5.614540	34.407455	48.974986	41.310878	2140	2912	3435
gene2282	NC_008261.1	2582636	2584582	-	protein_coding	CPF_RS11330	CPF_2326	.	WP_003451434.	type IIA DNA to	-1.141280	4.283094	-1.145359	4.285610	1.003573	4.382054	4.379482	4.188828	4.384628	4.379482	4.188828	4.384628	15.198282	26.335592	17.125007	626	1037	943
gene2283	NC_008261.1	2584572	2585642	-	protein_coding	CPF_RS11335	CPF_2327	.	WP_003454987.	tRNA 2-selenou	1.475526	3.626489	1.749608	3.491186	-1.185753	3.231478	3.356715	3.917945	3.110912	3.356715	3.917945	3.110912	7.017683	21.421940	6.767829	159	464	205
gene2284	NC_008261.1	2585888	2587288	-	protein_coding	CPF_RS11340	CPF_2328	.	WP_003451106.	glycosyl transfer	2.512158	9.720327	6.415203	8.967047	-2.553662	8.375003	9.078547	10.407475	7.725980	9.078547	10.407475	7.725980	461.633757	1528.764152	205.913022	13682	43316	8159
gene2285	NC_008261.1	2588139	2589377	+	protein_coding	CPF_RS11345	CPF_2329	.	WP_011591042.	UV DNA damage	1.167075	0.109049	1.159362	0.120206	1.006653	0.049027	0.044476	0.267374	0.054042	0.044476	0.267374	0.054042	0.038152	0.279355	0.028537	1	7	1
gene2286	NC_008261.1	2589462	2590070	+	protein_coding	CPF_RS11350	CPF_2330	.	WP_003451397.	sporulation mem	-1.024341	0.490331	1.206380	0.309663	-1.235745	0.320813	0.507986	0.473290	0.202606	0.507986	0.473290	0.202606	0.310477	0.568344	0.116118	4	7	2
gene2287	NC_008261.1	2590240	2591409	-	protein_coding	CPF_RS11355	CPF_2331	.	WP_011591043.	enoyl-[acyl-car	-1.154410	9.132557	-1.168483	9.141195	1.012191	9.245459	9.236722	9.029567	9.254203	9.236722	9.029567	9.254203	519.283776	616.171977	612.384697	12853	14580	20264
gene2288	NC_008261.1	2591647	2593137	-	protein_coding	CPF_RS11360	CPF_2332	.	WP_003451678.	amino acid perm	2.113692	6.448287	2.008672	6.488132	1.052283	5.967614	5.930966	7.010731	6.004488	5.930966	7.010731	6.004488	46.350651	168.202281	55.159089	1462	5072	2326
gene2289	NC_008261.1	2593484	2595724	-	protein_coding	CPF_RS11365	CPF_2333	.	WP_011591044.	DUF4968 domai	-3.399227	3.203581	-6.475274	3.540027	1.904925	4.647216	4.205542	2.440335	5.135276	4.205542	2.440335	5.135276	13.225491	6.332429	30.103814	627	287	1908
gene2290	NC_008261.1	2595773	2596666	-	protein_coding	CPF_RS11370	CPF_2334	.	WP_011591045.	ROK family prote	-4.538428	2.225316	-8.266246	2.480345	1.821390	3.978586	3.569508	1.387315	4.434547	3.569508	1.387315	4.434547	8.195591	2.322962	17.639341	155	42	446
gene2291	NC_008261.1	2596807	2598255	-	protein_coding	CPF_RS11375	CPF_2335	.	WP_003454778.	DUF3502 domai	-8.664363	4.707460	-17.903391	5.071602	2.066325	7.020026	6.515987	3.400894	7.563054	6.515987	3.400894	7.563054	71.704335	14.366269	181.425196	2198	421	7435
gene2292	NC_008261.1	2598640	2599134	+	protein_coding	CPF_RS11380	CPF_2336	.	WP_003454877.	membrane prote	1.034422	2.344590	1.169963	2.253055	-1.131031	2.229718	2.320305	2.369129	2.142667	2.320305	2.369129	2.142667	2.960347	5.993442	3.142914	31	60	44
gene2293	NC_008261.1	2599202	2602336	-	protein_coding	CPF_RS11385	CPF_2337	.	WP_011591046.	alpha-mannosida	-2.739741	2.455288	-6.880630	2.909371	2.511416	3.895710	3.287683	1.833644	4.616185	3.287683	1.833644	4.616185	6.694706	3.706471	20.210892	444	235	1792
gene2294	NC_008261.1	2602643	2603599	-	protein_coding	CPF_RS11390	CPF_2338	.	WP_003451608.	carbohydrate AB	-6.929046	4.379923	-18.662525	4.874318	2.693376	6.669968	5.993443	3.200786	7.422858	5.993443	3.200786	7.422858	48.850663	12.090220	163.746802	989	234	4432
gene2295	NC_008261.1	2603617	2604543	-	protein_coding	CPF_RS11395	CPF_2339	.	WP_041702761.	sugar ABC trans	-5.868848	3.744003	-16.514120	4.244556	2.813861	5.931696	5.232181	2.679104	6.724732	5.232181	2.679104	6.724732	28.351835	7.734256	94.897693	556	145	2488
gene2296	NC_008261.1	2604813	2605289	-	protein_coding	CPF_RS11400	CPF_2340	.	WP_003470675.	cyclic pyranopte	1.556671	6.324652	1.582893	6.311966	-1.016845	6.001410	6.013472	6.651936	5.989372	6.013472	6.651936	5.989372	49.747521	138.282662	54.778618	502	1334	739
gene2297	NC_008261.1	2605328	2605984	-	protein_coding	CPF_RS11405	CPF_2341	.	WP_003455194.	molybdate ABC	1.416783	3.922938	1.261581	4.011171	1.123022	3.762432	3.679670	4.182289	3.847056	3.679670	4.182289	3.847056	8.993542	26.190522	11.570659	125	348	215
gene2298	NC_008261.1	2605988	2606758	-	protein_coding	CPF_RS11410	CPF_2342	.	WP_003478550.	molybdate ABC	1.294179	4.852516	1.340539	4.826064	-1.035822	4.644604	4.670062	5.042099	4.619285	4.670062	5.042099	4.619285	19.435289	45.790366	20.224101	317	714	441
gene2299	NC_008261.1	2606743	2607234	-	protein_coding	CPF_RS11415	CPF_2343	.	WP_003478551.	molybdenum co	-1.093202	5.984011	1.243105	5.760964	-1.358965	5.823180	6.048636	5.920076	5.606128	6.048636	5.920076	5.606128	50.920994	88.138321	41.035149	530	877	571
gene2300	NC_008261.1	2607576	2609099	-	protein_coding	CPF_RS11420	CPF_2344	.	WP_003451481.	DNA-binding res	-1.094553	3.565274	-1.244004	3.654802	1.136541	3.722220	3.631040	3.500698	3.815690	3.631040	3.500698	3.815690	8.591736	15.768181	11.205911	277	486	483
gene2301	NC_008261.1	2609104	2610840	-	protein_coding	CPF_RS11425	CPF_2345	.	WP_003451463.	sensor histidine	-1.235404	2.716559	-1.185332	2.688193	-1.042242	2.843324	2.873326	2.568344	2.813636	2.873326	2.568344	2.813636	4.898451	7.059633	5.414606	180	248	266
gene2302	NC_008261.1	2611062	2611739	+	protein_coding	CPF_RS11430	CPF_2346	.	WP_003471057.	DUF624 domain	-2.491091	1.160902	-1.977172	1.059379	-1.259926	1.818703	1.992994	0.676216	1.659654	1.992994	0.676216	1.659654	2.231035	0.875149	1.877404	32	12	36
gene2303	NC_008261.1	2612006	2613343	+	protein_coding	CPF_RS11435	CPF_2347	.	WP_003471016.	peptidoglycan br	2.631719	7.233346	2.472223	7.282837	1.064516	6.613888	6.568943	7.964949	6.659140	6.568943	7.964949	6.659140	74.084683	295.271103	90.376394	2097	7990	3420
gene2304	NC_008261.1	2613709	2614014	+	protein_coding	CPF_RS11440	CPF_2348	.	WP_003451687.	PadR family tran	1.057769	6.765123	1.534251	6.489649	-1.450459	6.450902	6.724732	6.805757	6.188223	6.724732	6.805757	6.188223	84.344608	149.468813	62.973827	546	925	545
gene2305	NC_008261.1	2614061	2614798	-	protein_coding	CPF_RS11445	CPF_2349	.	WP_003451387.	amino acid ABC	17.064232	4.421535	15.829284	4.505531	1.078017	2.879387	2.825707	6.918611	2.934087	2.825707	6.918611	2.934087	4.611713	159.861675	5.845053	72	2386	122
gene2306	NC_008261.1	2614817	2616325	-	protein_coding	CPF_RS11450	CPF_2350	.	WP_003455090.	amino acid ABC	4.076590	5.151228	8.872083	4.416768	-2.176349	3.632322	4.236337	6.263700	3.114427	4.236337	6.263700	3.114427	13.407279	107.116398	6.771636	428	3269	289
gene2307	NC_008261.1	2616498	2617277	+	protein_coding	CPF_RS11455	CPF_2351	.	WP_003455000.	iron-sulfur clust	-2.150822	6.131653	-1.830472	6.024388	-1.175010	6.591570	6.708933	5.604045	6.476261	6.708933	5.604045	6.476261	83.025610	70.175142	79.600340	1370	1107	1756
gene2308	NC_008261.1	2617410	2617826	-	protein_coding	CPF_RS11460	CPF_2352	.	WP_003454898.	DUF86 domain-	1.202083	3.031680	1.563054	2.826869	-1.300288	2.705780	2.901817	3.167354	2.522986	2.901817	3.167354	2.522986	4.987728	11.620379	4.239542	44	98	50
gene2309	NC_008261.1	2618333	2619154	-	protein_coding	CPF_RS11465	CPF_2353	.	WP_011591047.	hypothetical pro	-4.120894	4.675938	-4.112820	4.674799	-1.001963	5.806275	5.807689	3.764732	5.804860	5.807689	3.764732	5.804860	42.324526	19.008399	47.917967	736	316	1114
gene2310	NC_008261.1	2619173	2620429	-	protein_coding	CPF_RS11470	CPF_2354	.	WP_011591048.	hypothetical pro	-3.817140	4.453118	-4.228948	4.512312	1.107884	5.596403	5.522988	3.590495	5.670794	5.522988	3.590495	5.670794	35.273915	16.835994	43.346334	938	428	1541
gene2311	NC_008261.1	2620546	2621994	-	protein_coding	CPF_RS11475	CPF_2355	.	WP_011591049.	sensor histidine	-2.767165	4.084013	-2.528566	4.029252	-1.094362	4.818205	4.883688	3.415280	4.753599	4.883688	3.415280	4.753599	22.379060	14.502766	22.302976	686	425	914
gene2312	NC_008261.1	2621984	2622682	-	protein_coding	CPF_RS11480	CPF_2356	.	WP_011591050.	DNA-binding res	-2.528945	4.864197	-2.182701	4.770700	-1.158631	5.472050	5.579291	4.240756	5.366870	5.579291	4.240756	5.366870	37.058641	27.375627	35.104865	548	387	694
gene2313	NC_008261.1	2622885	2623280	-	protein_coding	CPF_RS11485	CPF_2357	.	WP_003451775.	hypothetical pro	-3.124435	5.380353	-2.734699	5.297168	-1.142515	6.167695	6.264550	4.620955	6.072337	6.264550	4.620955	6.072337	61.355577	34.712020	58.126051	514	278	651
gene2314	NC_008261.1	2623413	2623703	-	protein_coding	CPF_RS11490	CPF_2358	.	WP_003451429.	DUF503 domain	-1.802573	2.380375	-1.482821	2.259365	-1.215638	2.698521	2.843051	1.992994	2.561338	2.843051	1.992994	2.561338	4.710762	4.247929	4.374159	29	25	36
gene2315	NC_008261.1	2623715	2625472	-	protein_coding	CPF_RS11495	CPF_2359	.	WP_003451399.	RNA helicase	-1.903318	3.512489	-1.647435	3.419983	-1.155322	3.901759	4.007296	3.078779	3.799001	4.007296	3.078779	3.799001	11.535184	10.856722	11.122215	429	386	553
gene2316	NC_008261.1	2625734	2626594	+	protein_coding	CPF_RS11500	CPF_2360	.	WP_011591051.	hypothetical pro	1.501724	4.501789	2.240598	4.182410	-1.492017	3.918777	4.218024	4.804643	3.640760	4.218024	4.804643	3.640760	13.286125	39.453348	9.896894	242	687	241
gene2317	NC_008261.1	2627002	2627517	-	protein_coding	CPF_RS11505	CPF_2361	.	WP_011591052.	heptaprenyl diph	2.272332	1.718147	2.734926	1.519188	-1.203577	1.083339	1.225218	2.409392	0.957890	1.225218	2.409392	0.957890	1.007695	6.132825	0.822274	11	64	12
gene2318	NC_008261.1	2627535	2627909	-	protein_coding	CPF_RS11510	CPF_2362	.	WP_003463119.	NusG domain II-	1.843946	1.609602	1.637095	1.718456	1.126353	1.310651	1.227629	2.110426	1.399288	1.227629	2.110426	1.399288	1.008428	4.746806	1.414311	8	36	15
gene2319	NC_008261.1	2627910	2628941	-	protein_coding	CPF_RS11515	CPF_2363	.	WP_003473292.	FAD:protein FMN	1.254846	1.347233	1.338680	1.293498	-1.066808	1.145794	1.193394	1.520904	1.100093	1.193394	1.520904	1.100093	0.961891	2.731024	1.027843	21	57	30
gene2320	NC_008261.1	2629113	2632358	-	protein_coding	CPF_RS11520	CPF_2364	.	WP_011591053.	DUF1533 domai	1.106650	1.381149	1.359039	1.214898	-1.228065	1.152298	1.309982	1.456182	1.013595	1.309982	1.456182	1.013595	1.121317	2.559122	0.893203	77	168	82
gene2321	NC_008261.1	2632371	2633417	-	protein_coding	CPF_RS11525	CPF_2365	.	WP_011591054.	hypothetical pro	2.361778	2.146903	2.381953	2.138729	-1.008542	1.608533	1.614680	2.854554	1.602409	1.614680	2.854554	1.602409	1.535035	9.067443	1.789840	34	192	53
gene2322	NC_008261.1	2633617	2634066	-	protein_coding	CPF_RS11530	CPF_2366	.	WP_011591055.	GNAT family N-a	-2.702175	6.425638	-3.646715	6.616260	1.349548	7.395662	7.182584	5.748463	7.615061	7.182584	5.748463	7.615061	120.486118	77.904761	187.946255	1147	709	2392
gene2323	NC_008261.1	2634354	2635775	+	protein_coding	CPF_RS11535	CPF_2367	.	WP_003455192.	MFS transporter	-17.775967	6.217985	-13.769746	6.083831	-1.290944	8.445072	8.631294	4.479438	8.262868	8.631294	4.479438	8.262868	333.715951	31.399189	303.873034	10039	903	12221
gene2324	NC_008261.1	2635900	2637654	-	protein_coding	CPF_RS11540	CPF_2368	.	WP_003455029.	peptidase S8	1.061018	1.625240	1.126818	1.580054	-1.062016	1.539063	1.583077	1.668526	1.496272	1.583077	1.668526	1.496272	1.508332	3.240045	1.571457	56	115	78
gene2325	NC_008261.1	2637656	2639266	-	protein_coding	CPF_RS11545	CPF_2369	.	WP_003454870.	subtilase	1.674398	1.274911	1.790663	1.208621	-1.069437	0.906485	0.956203	1.699845	0.859352	0.956203	1.699845	0.859352	0.704209	3.345502	0.702327	24	109	32
gene2326	NC_008261.1	2639725	2641080	-	protein_coding	CPF_RS11550	CPF_2370	.	WP_003454771.	RNA methyltran	5.104310	7.857404	3.812424	8.098068	1.338862	6.976371	6.769042	9.120758	7.190050	6.769042	9.120758	7.190050	87.358966	646.297286	132.826358	2506	17724	5094
gene2327	NC_008261.1	2641108	2641557	-	protein_coding	CPF_RS11555	CPF_2371	.	WP_003454831.	hypothetical pro	5.789784	7.290768	4.105632	7.579788	1.410206	6.376376	6.133242	8.666752	6.629148	6.133242	8.666752	6.629148	54.308041	487.976087	88.473028	517	4441	1126
gene2328	NC_008261.1	2641744	2643651	-	protein_coding	CPF_RS11560	CPF_2372	.	WP_003454944.	selenocysteine-	-1.046582	5.104640	1.062771	5.027795	-1.112277	5.060248	5.137588	5.071902	4.984072	5.137588	5.071902	4.984072	26.558438	46.828480	26.926026	1072	1807	1453
gene2329	NC_008261.1	2643652	2645040	-	protein_coding	CPF_RS11565	CPF_2373	.	WP_003455025.	L-selenocystein	-1.122460	6.288699	-1.186887	6.328304	1.057398	6.412717	6.372583	6.205919	6.453103	6.372583	6.205919	6.453103	66.225721	103.626359	77.995856	1946	2911	3064
gene2330	NC_008261.1	2645057	2646088	-	protein_coding	CPF_RS11570	CPF_2374	.	WP_011591056.	selenide, water	-1.087473	6.716059	-1.045600	6.687930	-1.040047	6.748438	6.776821	6.655842	6.720173	6.776821	6.655842	6.720173	87.852680	138.419771	94.630033	1918	2889	2762
gene2331	NC_008261.1	2646133	2646897	-	protein_coding	CPF_RS11575	CPF_2375	.	WP_003454885.	competence pro	-1.334261	0.743495	-1.020893	0.578751	-1.306955	0.762904	0.980068	0.564027	0.593859	0.980068	0.564027	0.593859	0.741491	0.710987	0.415974	12	11	9
gene2332	NC_008261.1	2647093	2648427	+	protein_coding	CPF_RS11580	CPF_2376	.	WP_003455081.	D-alanyl-D-alan	1.422041	4.505824	2.153316	4.177196	-1.514243	3.948369	4.258995	4.766958	3.660398	4.258995	4.766958	3.660398	13.738413	38.778919	9.984932	388	1047	377
gene2333	NC_008261.1	2648459	2649355	-	protein_coding	CPF_RS11585	CPF_2377	.	WP_003455017.	RluA family pseu	-2.678298	6.369810	-2.210000	6.244550	-1.211899	6.979977	7.119988	5.698672	6.842718	7.119988	5.698672	6.842718	115.408496	75.243760	104.496633	2190	1365	2651
gene2334	NC_008261.1	2649352	2650665	-	protein_coding	CPF_RS11590	CPF_2378	.	WP_011591057.	LytR family trans	-2.900990	5.712930	-2.489077	5.615493	-1.165488	6.421212	6.532629	4.996084	6.311695	6.532629	4.996084	6.311695	72.128204	44.553992	69.235596	2005	1184	2573
gene2335	NC_008261.1	2650688	2651257	-	protein_coding	CPF_RS11595	CPF_2379	.	WP_003454731.	HD domain-con	-1.898359	5.742820	-1.426495	5.549352	-1.330786	6.014109	6.223780	5.299027	5.811501	6.223780	5.299027	5.811501	58.963174	56.038686	48.136209	711	646	776
gene2336	NC_008261.1	2651260	2651868	-	protein_coding	CPF_RS11600	CPF_2380	.	WP_003453701.	nicotinate-nico	-1.448883	4.986685	-1.123151	4.809429	-1.290016	5.074305	5.261323	4.726382	4.893934	5.261323	4.726382	4.893934	28.796700	37.267106	24.733030	371	459	426
gene2337	NC_008261.1	2651870	2652172	-	protein_coding	CPF_RS11605	CPF_2381	.	WP_003453740.	ribosome assem	1.036260	6.890393	1.118914	6.834605	-1.079761	6.809167	6.864748	6.916135	6.754036	6.864748	6.916135	6.754036	93.292056	159.760843	96.854650	598	979	830
gene2338	NC_008261.1	2652190	2653476	-	protein_coding	CPF_RS11610	CPF_2382	.	WP_003454769.	GTPase Obg	-1.204267	8.304004	1.097469	8.103641	-1.321646	8.235540	8.439164	8.171009	8.036828	8.439164	8.171009	8.036828	293.316752	339.436302	251.130913	7986	8835	9141
gene2339	NC_008261.1	2653643	2653945	-	protein_coding	CPF_RS11615	CPF_2383	.	WP_003454907.	50S ribosomal p	1.963874	12.340895	1.711190	12.443814	1.147665	11.962582	11.863643	12.837346	12.062345	11.863643	12.837346	12.062345	3289.403006	8636.876798	4078.514323	21085	52926	34951
gene2340	NC_008261.1	2653949	2654320	-	protein_coding	CPF_RS11620	CPF_2384	.	WP_003478582.	ribosomal-proce	2.096230	11.137023	1.789281	11.256200	1.171549	10.729515	10.615914	11.683711	10.844332	10.615914	11.683711	10.844332	1341.224277	3784.870926	1818.739676	10555	28475	19135
gene2341	NC_008261.1	2654330	2654641	-	protein_coding	CPF_RS11625	CPF_2385	.	WP_003452389.	50S ribosomal p	1.950708	11.804381	1.647962	11.930434	1.183710	11.453230	11.332218	12.296216	11.575533	11.332218	12.296216	11.575533	2157.453795	5751.889332	2919.736847	14240	36294	25764
gene2342	NC_008261.1	2654767	2656209	-	protein_coding	CPF_RS11630	CPF_2386	.	WP_003455169.	ribonuclease	1.973052	4.539888	1.932745	4.556441	1.020855	4.090926	4.076064	5.056493	4.105842	4.076064	5.056493	4.105842	12.055011	46.122093	13.893183	368	1346	567
gene2343	NC_008261.1	2656226	2656927	-	protein_coding	CPF_RS11635	CPF_2387	.	WP_003455013.	radical SAM prot	1.702238	4.597739	1.978910	4.478102	-1.162534	4.119939	4.230007	4.997440	4.012734	4.230007	4.997440	4.012734	13.399916	44.656268	12.894006	199	634	256
gene2344	NC_008261.1	2656911	2658764	-	protein_coding	CPF_RS11640	CPF_2388	.	WP_004457875.	B12-binding dom	1.940095	5.579855	2.326732	5.435179	-1.199288	4.989422	5.122233	6.078360	4.860055	5.122233	6.078360	4.860055	26.388623	97.238268	24.067703	1035	3646	1262
gene2345	NC_008261.1	2658896	2659750	-	protein_coding	CPF_RS11645	CPF_2389	.	WP_003455164.	peptidase M50	1.092945	0.341608	-1.043146	0.441102	1.140102	0.366020	0.283462	0.411683	0.472624	0.283462	0.411683	0.472624	0.165860	0.462652	0.330833	3	8	8
gene2346	NC_008261.1	2659747	2660589	-	protein_coding	CPF_RS11650	CPF_2390	.	WP_003454782.	peptidase M23	-2.047319	1.707335	-2.015067	1.698814	-1.016006	2.289242	2.300725	1.266989	2.277816	2.300725	1.266989	2.277816	2.915828	2.052914	3.523195	52	35	84
gene2347	NC_008261.1	2660742	2661866	-	protein_coding	CPF_RS11655	CPF_2391	.	WP_003454998.	rod shape-deter	2.767248	7.787100	3.373854	7.628396	-1.219209	6.942967	7.087412	8.555864	6.801466	7.087412	8.555864	6.801466	112.229615	453.144198	101.516121	2671	10310	3230
gene2348	NC_008261.1	2662014	2662286	-	protein_coding	CPF_RS11660	CPF_2392	.	WP_003452508.	cell division topo	2.037523	8.199005	1.957188	8.229838	1.041046	7.730618	7.701656	8.728472	7.759689	7.701656	8.728472	7.759689	170.206828	505.688465	212.664755	983	2792	1642
gene2349	NC_008261.1	2662297	2663094	-	protein_coding	CPF_RS11665	CPF_2393	.	WP_003452408.	septum site-det	1.866391	7.775227	1.876327	7.771168	-1.005324	7.334289	7.338120	8.238371	7.330460	7.338120	8.238371	7.330460	132.036282	352.627335	149.805340	2229	5691	3381
gene2350	NC_008261.1	2663123	2663758	-	protein_coding	CPF_RS11670	CPF_2394	.	WP_003452463.	septum site-det	2.093204	7.062130	2.264619	7.000639	-1.081892	6.492322	6.549348	7.615061	6.435792	6.549348	7.615061	6.435792	73.060478	247.618222	76.163776	983	3185	1370
gene2351	NC_008261.1	2664005	2667076	-	protein_coding	CPF_RS11675	CPF_2395	.	WP_003455183.	penicillin-binding	2.164580	7.615196	1.787001	7.762528	1.211292	7.215448	7.078499	8.192586	7.355046	7.078499	8.192586	7.355046	111.312363	343.497701	153.078939	7234	21341	13300
gene2352	NC_008261.1	2667085	2667582	-	protein_coding	CPF_RS11680	CPF_2396	.	WP_003455077.	rod shape-deter	1.953088	6.026282	1.456690	6.251242	1.340772	5.770374	5.562718	6.528475	5.985782	5.562718	6.528475	5.985782	36.639039	128.579196	54.740663	386	1295	771
gene2353	NC_008261.1	2667598	2668449	-	protein_coding	CPF_RS11685	CPF_2397	.	WP_003452517.	rod shape-deter	2.276931	7.452083	1.876921	7.601488	1.213121	7.020116	6.882137	8.069228	7.160860	6.882137	8.069228	7.160860	94.817517	314.666275	128.275709	1709	5422	3091
gene2354	NC_008261.1	2668453	2669481	-	protein_coding	CPF_RS11690	CPF_2398	.	WP_003452282.	rod shape-deter	1.969655	10.411414	2.041976	10.384116	-1.036718	9.907873	9.933918	10.911861	9.881895	9.933918	10.911861	9.881895	853.663200	2368.645984	1001.116598	18583	49293	29135
gene2355	NC_008261.1	2669501	2670184	-	protein_coding	CPF_RS11695	CPF_2399	.	WP_003455187.	JAB domain-co	2.635936	8.577801	2.616573	8.583568	1.007400	7.912406	7.907089	9.305405	7.917726	7.907089	9.305405	7.917726	202.763657	729.977620	235.770240	2934	10098	4561
gene2356	NC_008261.1	2670231	2670809	-	protein_coding	CPF_RS11700	CPF_2400	.	WP_003455083.	maf-like protein	2.810400	8.588773	2.971020	8.544940	-1.057152	7.835475	7.875669	9.366445	7.795486	7.875669	9.366445	7.795486	195.856411	755.437696	217.642721	2399	8846	3564
gene2357	NC_008261.1	2671035	2671838	-	protein_coding	CPF_RS11705	CPF_2401	.	WP_003454754.	SPOR domain-c	-1.065759	1.129796	1.034650	1.059933	-1.102687	1.103909	1.176670	1.084789	1.035647	1.176670	1.084789	1.035647	0.940698	1.598997	0.923524	16	26	21
gene2358	NC_008261.1	2672100	2672918	-	protein_coding	CPF_RS11710	CPF_2402	.	WP_003454845.	PHP domain-co	1.406673	5.375787	1.435811	5.360283	-1.020714	5.120465	5.135276	5.627563	5.105698	5.135276	5.627563	5.105698	26.492009	71.240734	29.443232	459	1180	682
gene2359	NC_008261.1	2673232	2674683	-	protein_coding	CPF_RS11715	CPF_2403	.	WP_011591059.	aminoacyl-histid	-1.033438	9.135016	1.188608	8.985820	-1.228354	9.009189	9.158773	9.111321	8.862047	9.158773	9.111321	8.862047	489.043228	641.809136	451.128285	15022	18847	18526
gene2360	NC_008261.1	2674816	2676240	+	protein_coding	CPF_RS11720	CPF_2404	.	WP_003452279.	pyruvate kinase	-2.013216	8.374821	-1.380823	8.114693	-1.457983	8.618492	8.894769	7.885267	8.350795	8.894769	7.885267	8.350795	403.470652	285.363562	328.368342	12163	8224	13234
gene2361	NC_008261.1	2676482	2677618	-	protein_coding	CPF_RS11725	CPF_2405	.	WP_003454971.	30S ribosomal p	-1.170649	7.711055	-1.090213	7.660289	-1.073780	7.774027	7.825546	7.598238	7.722847	7.825546	7.598238	7.722847	188.415030	245.098583	205.802816	4532	5636	6618
gene2362	NC_008261.1	2677764	2677967	-	protein_coding	CPF_RS11730	CPF_2406	.	WP_003452305.	heavy metal-bin	-3.075582	8.304042	-1.853224	7.965663	-1.659585	8.780914	9.153925	7.533065	8.423103	9.153925	7.533065	8.423103	487.993804	233.898495	348.204824	2106	965	2009
gene2363	NC_008261.1	2678072	2678836	-	protein_coding	CPF_RS11735	CPF_2407	.	WP_003455051.	peroxide stress	-1.951948	7.153409	-1.648293	7.038464	-1.184224	7.529158	7.652117	6.687203	7.408175	7.652117	6.687203	7.408175	164.178479	140.840019	161.120560	2657	2179	3486
gene2364	NC_008261.1	2679002	2679412	-	protein_coding	CPF_RS11740	CPF_2408	.	WP_003452437.	CBS domain-co	1.716334	8.217374	1.885069	8.146138	-1.098312	7.769005	7.836943	8.616273	7.701656	7.836943	8.616273	7.701656	190.000319	473.044468	201.479138	1652	3932	2342
gene2365	NC_008261.1	2679498	2680400	-	protein_coding	CPF_RS11745	CPF_2409	.	WP_003452466.	exonuclease	1.656753	4.682210	1.794345	4.619592	-1.083049	4.274235	4.332172	5.060531	4.217073	4.332172	5.060531	4.217073	14.552686	46.379487	15.231647	278	847	389
gene2366	NC_008261.1	2680655	2682400	-	protein_coding	CPF_RS11750	CPF_2410	.	WP_003454902.	DUF262 domain	4.330036	3.739697	4.778616	3.644490	-1.103597	2.757043	2.829066	4.943445	2.686853	2.829066	4.943445	2.686853	4.629541	43.583757	5.001931	171	1539	247
gene2367	NC_008261.1	2682582	2684369	-	protein_coding	CPF_RS11755	CPF_2411	.	WP_003454750.	DNA mismatch r	1.126038	5.241815	1.178728	5.208177	-1.046792	5.123793	5.156887	5.328143	5.090912	5.156887	5.328143	5.090912	26.913264	57.244414	29.227518	1018	2070	1478
gene2368	NC_008261.1	2684693	2686222	-	protein_coding	CPF_RS11760	CPF_2412	.	WP_003454995.	PTS glucose tra	-1.176551	0.618798	1.057821	0.470240	-1.244581	0.567737	0.747097	0.512533	0.431437	0.747097	0.512533	0.431437	0.494327	0.614034	0.300426	16	19	13
gene2369	NC_008261.1	2686393	2687289	-	protein_coding	CPF_RS11765	CPF_2413	.	WP_003454919.	ABC transporter	-1.543199	6.566106	-1.217591	6.401038	-1.267420	6.713399	6.886522	6.260598	6.544628	6.886522	6.260598	6.544628	95.383277	106.994973	82.856252	1810	1941	2102
gene2370	NC_008261.1	2687515	2690283	-	protein_coding	CPF_RS11770	CPF_2415	.	WP_011591062.	hypothetical pro	-1.040771	3.293976	-1.207658	3.398658	1.160350	3.428531	3.322929	3.265276	3.537488	3.322929	3.265276	3.537488	6.862608	12.714150	9.270404	402	712	726
gene2371	NC_008261.1	2690273	2691412	-	protein_coding	CPF_RS11775	CPF_2416	.	WP_003457331.	DNA repair exon	-1.582537	4.116826	-1.575494	4.113856	-1.004470	4.458021	4.461240	3.799001	4.454805	4.461240	3.799001	4.454805	16.544519	19.387997	17.989047	399	447	580
gene2372	NC_008261.1	2691692	2691874	-	protein_coding	CPF_RS11780	CPF_2417	.	WP_003452440.	Small, acid-solub	-3.313404	8.241836	-3.705123	8.314113	1.118222	9.231424	9.151172	7.422858	9.312379	9.151172	7.422858	9.312379	485.874172	219.399291	631.030143	1881	812	3266
gene2373	NC_008261.1	2692014	2694188	-	protein_coding	CPF_RS11785	CPF_2418	.	WP_003457402.	2',3'-cyclic-nuc	-6.259329	5.962685	-7.517966	6.067815	1.201082	7.561713	7.430700	4.784692	7.695035	7.430700	4.784692	7.695035	139.267362	39.102045	199.255326	6408	1720	12257
gene2375	NC_008261.1	2694450	2694525	-	tRNA	CPF_RS11795	CPF_2420	.	.	tRNA-Trp	1.753684	1.751342	2.949721	1.189404	-1.682014	0.945639	1.392410	2.202799	0.642220	1.392410	2.202799	0.642220	1.243949	5.204832	0.465234	2	8	1
gene2376	NC_008261.1	2694788	2695408	-	protein_coding	CPF_RS11800	CPF_2421	.	WP_003457372.	CBS domain-co	1.532210	9.507356	1.425834	9.560817	1.074606	9.256289	9.204530	9.820144	9.308339	9.204530	9.820144	9.308339	506.040783	978.089256	630.063150	6648	12284	11066
gene2377	NC_008261.1	2695708	2696172	+	protein_coding	CPF_RS11805	CPF_2422	.	WP_003457392.	tRNA (adenosine	1.840819	6.190882	2.387375	5.986151	-1.296909	5.575645	5.766336	6.646684	5.391259	5.766336	6.646684	5.391259	41.577317	138.023177	35.661935	409	1298	469
gene2378	NC_008261.1	2696166	2696870	+	protein_coding	CPF_RS11810	CPF_2423	.	WP_003457395.	tRNA (adenosine	1.172543	5.827553	1.194756	5.813729	-1.018944	5.700310	5.713863	5.943504	5.686788	5.713863	5.943504	5.686788	40.095735	89.213027	43.984078	598	1272	877
gene2379	NC_008261.1	2696870	2697316	+	protein_coding	CPF_RS11815	CPF_2424	.	WP_003457406.	ribosomal-prote	1.388478	6.079897	1.299317	6.129472	1.068622	5.895434	5.847753	6.321258	5.943504	5.847753	6.321258	5.943504	43.568820	110.727842	52.918023	412	1001	669
gene2380	NC_008261.1	2697410	2698006	-	protein_coding	CPF_RS11820	CPF_2425	.	WP_003468145.	ECF transporter	5.413641	7.390073	5.178416	7.427737	1.045424	6.303485	6.271523	8.708122	6.335611	6.271523	8.708122	6.335611	61.522333	500.422318	70.360209	777	6042	1188
gene2381	NC_008261.1	2698353	2700515	-	protein_coding	CPF_RS11825	CPF_2426	.	WP_011591063.	RNA-binding tra	2.336401	3.886466	1.775054	4.111809	1.316242	3.514863	3.322235	4.546523	3.718660	3.322235	4.546523	3.718660	6.862135	32.529595	10.461850	314	1423	640
gene2382	NC_008261.1	2700627	2701970	+	protein_coding	CPF_RS11830	CPF_2427	.	WP_011591064.	peptidase M28	-1.172297	0.337508	-1.124977	0.315499	-1.042062	0.440402	0.471124	0.241787	0.411683	0.471124	0.241787	0.411683	0.281369	0.257531	0.289387	8	7	11
gene2383	NC_008261.1	2702218	2703189	-	protein_coding	CPF_RS11835	.	.	WP_003452421.	peptide chain re	-1.258121	6.840294	-1.168332	6.787962	-1.076851	6.954319	7.007934	6.676664	6.901115	7.007934	6.676664	6.901115	105.239094	140.198455	109.128957	2164	2756	3000
gene2384	NC_008261.1	2703566	2706088	-	protein_coding	CPF_RS11840	CPF_2429	.	WP_003457334.	protein transloc	-1.369707	9.403376	-1.238624	9.332273	-1.105829	9.560208	9.633047	9.179180	9.487919	9.633047	9.179180	9.487919	713.622325	672.468151	710.897427	38089	34313	50727
gene2385	NC_008261.1	2706323	2706862	-	protein_coding	CPF_RS11845	CPF_2430	.	WP_003452413.	ribosome-assoc	-4.991833	12.763310	-5.344423	12.808193	1.070633	14.024827	13.975681	11.656111	14.074145	13.975681	11.656111	14.074145	17463.571691	3765.946202	17130.190623	199499	41128	261620
gene2386	NC_008261.1	2707314	2707973	-	protein_coding	CPF_RS11850	CPF_2431	.	WP_003457335.	amidophosphorib	1.439255	3.519180	1.247105	3.628844	1.154077	3.368091	3.266307	3.791629	3.473047	3.266307	3.791629	3.473047	6.589159	19.328852	8.678728	92	258	162
gene2387	NC_008261.1	2707949	2708911	-	protein_coding	CPF_RS11855	CPF_2432	.	WP_003457387.	hypothetical pro	1.305855	2.520641	1.312429	2.516728	-1.005034	2.331858	2.335483	2.720478	2.328238	2.335483	2.720478	2.328238	2.994261	8.009928	3.671628	61	156	100

gene2388	NC_008261.1	2708929	2711163	-	protein_coding	CPF_RS11860	CPF_2433	.	WP_003457380.	ATP-dependent	1.101516	4.110760	1.285799	3.995652	-1.167299	3.928435	4.041606	4.181097	3.818432	4.041606	4.181097	3.818432	11.865101	26.172035	11.248046	561	1183	711
gene2389	NC_008261.1	2711300	2712436	+	protein_coding	CPF_RS11865	CPF_2434	.	WP_003457417.	N-acetylglucosa	-1.188045	8.701930	-1.015306	8.589473	-1.170135	8.713037	8.827112	8.578523	8.600437	8.827112	8.578523	8.600437	388.055140	461.582037	380.321613	9334	10614	12230
gene2390	NC_008261.1	2712572	2713747	-	protein_coding	CPF_RS11870	CPF_2435	.	WP_003452418.	S-adenosylmeth	2.402925	8.997684	2.962479	8.834206	-1.232864	8.235093	8.387485	9.652276	8.085471	8.387485	9.652276	8.085471	285.750698	889.689822	263.860455	7109	21160	8776
gene2391	NC_008261.1	2714178	2714263	-	tRNA	CPF_RS11875	CPF_2436	.	.	tRNA-Tyr	3.174157	3.314635	1.530957	3.931734	2.073315	3.065740	2.584562	4.250935	3.636501	2.584562	4.250935	3.636501	3.847563	27.597711	9.867288	7	48	24
gene2397	NC_008261.1	2714726	2714801	-	tRNA	CPF_RS11905	CPF_2442	.	.	tRNA-Thr	1.255136	1.039427	-1.112199	1.290625	1.395962	1.103039	0.888351	1.216195	1.369611	0.888351	1.216195	1.369611	0.621974	1.951812	1.395702	1	3	3
gene2398	NC_008261.1	2714966	2715628	-	protein_coding	CPF_RS11910	CPF_2443	.	WP_003457339.	hypothetical pro	-1.162340	0.789070	1.116381	0.603362	-1.297614	0.692018	0.905013	0.687981	0.529152	0.905013	0.687981	0.529152	0.641675	0.894948	0.373310	9	12	7
gene2399	NC_008261.1	2715716	2716246	+	protein_coding	CPF_RS11915	CPF_2444	.	WP_003457351.	spore protease	1.080809	0.353089	-1.334036	0.585660	1.441838	0.500017	0.301456	0.413567	0.829364	0.301456	0.413567	0.829364	0.178042	0.465592	0.665872	2	5	10
gene2400	NC_008261.1	2716285	2717322	-	protein_coding	CPF_RS11920	CPF_2445	.	WP_003452335.	rod shape-deter	2.073003	6.817304	2.165486	6.783213	-1.044613	6.280132	6.311695	7.363417	6.248726	6.311695	7.363417	6.248726	63.345517	212.645949	65.606058	1391	4464	1926
gene2401	NC_008261.1	2717413	2717664	-	protein_coding	CPF_RS11925	CPF_2446	.	WP_003452341.	sporulation tran	-1.100312	0.237300	-1.597339	0.390023	1.451714	0.519492	0.316072	0.178160	0.853830	0.316072	0.178160	0.853830	0.187580	0.196214	0.701543	1	1	5
gene2402	NC_008261.1	2717741	2718511	-	protein_coding	CPF_RS11930	CPF_2447	.	WP_003457419.	M23 family pepti	-2.285630	1.763799	-2.066832	1.710924	-1.105862	2.384475	2.458165	1.265573	2.312994	2.458165	1.265573	2.312994	3.433363	2.052229	3.622912	56	32	79
gene2403	NC_008261.1	2718621	2719643	-	protein_coding	CPF_RS11935	CPF_2448	.	WP_003457347.	stage II sporulat	-1.273116	0.929022	-1.142734	0.861916	-1.114096	1.038534	1.119391	0.771028	0.963518	1.119391	0.771028	0.963518	0.877938	1.063353	0.829508	19	22	24
gene2404	NC_008261.1	2719755	2721020	-	protein_coding	CPF_RS11940	CPF_2449	.	WP_003457368.	UDP-N-acetylg	1.121871	2.086996	1.281746	1.984481	-1.142507	1.907169	2.005690	2.171597	1.813487	2.005690	2.171597	1.813487	2.240287	5.077383	2.206370	60	130	79
gene2405	NC_008261.1	2721043	2721687	-	protein_coding	CPF_RS11945	CPF_2450	.	WP_003457349.	hypothetical pro	1.037371	0.618591	1.071966	0.593380	-1.033348	0.568535	0.592691	0.645623	0.545364	0.592691	0.645623	0.545364	0.366435	0.843263	0.383728	5	11	7
gene2406	NC_008261.1	2721869	2722270	-	protein_coding	CPF_RS11950	CPF_2451	.	WP_003452358.	ATP synthase e	-1.116967	11.344290	-1.114772	11.342881	-1.001969	11.422945	11.424364	11.264777	11.421526	11.424364	11.264777	11.421526	2367.618305	3058.265775	2640.223635	20135	24864	30018
gene2407	NC_008261.1	2722286	2723683	-	protein_coding	CPF_RS11955	CPF_2452	.	WP_003452277.	ATP synthase s	-1.224627	11.274407	-1.098545	11.196775	-1.114772	11.342881	11.421526	11.129183	11.264777	11.421526	11.129183	11.264777	2363.536565	2679.345251	2445.377591	69901	75754	96687
gene2408	NC_008261.1	2723697	2724548	-	protein_coding	CPF_RS11960	CPF_2453	.	WP_003452288.	ATP synthase s	1.038652	10.511668	1.184816	10.416010	-1.140724	10.388938	10.484347	10.539059	10.294397	10.484347	10.539059	10.294397	1231.185202	1671.700858	1337.163257	22191	28805	32221
gene2409	NC_008261.1	2724613	2726121	-	protein_coding	CPF_RS11965	CPF_2454	.	WP_003452357.	ATP synthase s	-1.210179	11.392616	-1.009597	11.262724	-1.198676	11.399588	11.531058	11.255837	11.269616	11.531058	11.255837	11.269616	2575.450528	3030.486417	2446.528891	82216	92485	104413
gene2410	NC_008261.1	2726132	2726671	-	protein_coding	CPF_RS11970	CPF_2455	.	WP_003457416.	ATP synthase s	1.459596	9.756891	1.432735	9.770659	1.018748	9.501308	9.487919	10.033488	9.514716	9.487919	10.033488	9.514716	641.122006	1131.303864	726.275034	7324	12355	11092
gene2411	NC_008261.1	2726674	2727153	-	protein_coding	CPF_RS11975	CPF_2456	.	WP_003452454.	ATP synthase s	1.555789	9.595192	1.377993	9.685265	1.129025	9.368794	9.281665	9.919311	9.456742	9.281665	9.919311	9.456742	537.499918	1055.257902	701.778311	5458	10244	9527
gene2412	NC_008261.1	2727253	2727471	-	protein_coding	CPF_RS11980	CPF_2457	.	WP_003452423.	ATP synthase s	1.256714	10.555543	1.448206	10.451112	-1.152375	10.289188	10.392002	10.721659	10.187392	10.392002	10.721659	10.187392	1145.273571	1984.835166	1236.876567	5306	8791	7661
gene2413	NC_008261.1	2727532	2728206	-	protein_coding	CPF_RS11985	CPF_2458	.	WP_003452429.	F0F1 ATP synth	1.359581	10.733163	1.654562	10.587577	-1.216965	10.371258	10.513869	10.957030	10.230581	10.513869	10.957030	10.230581	1265.296822	2463.211573	1283.356533	18068	33626	24500
gene2414	NC_008261.1	2728272	2728628	-	protein_coding	CPF_RS11990	CPF_2459	.	WP_003482158.	hypothetical pro	2.710988	5.607212	3.324577	5.437375	-1.226334	4.784326	4.933765	6.372583	4.639413	4.933765	6.372583	4.639413	23.171596	113.850222	20.501571	175	822	207
gene2415	NC_008261.1	2729310	2730488	-	protein_coding	CPF_RS11995	CPF_2460	.	WP_003457425.	acetyl-CoA C-a	1.105426	9.339153	-1.103475	9.482495	1.219809	9.409368	9.267132	9.411734	9.553787	9.267132	9.411734	9.553787	535.967851	780.901311	747.671640	13368	18620	24931
gene2416	NC_008261.1	2730756	2731910	-	protein_coding	CPF_RS12000	CPF_2461	.	WP_003452343.	UDP-N-acetylg	1.730330	8.076731	1.898238	8.006265	-1.097038	7.623786	7.690886	8.481933	7.557273	7.690886	8.481933	7.557273	169.681081	427.632111	180.921638	4146	9989	5910
gene2417	NC_008261.1	2731974	2732603	-	protein_coding	CPF_RS12005	CPF_2462	.	WP_003452430.	uracil phosphori	2.906853	9.860490	3.840561	9.640817	-1.321209	8.917566	9.120758	10.660216	8.718900	9.120758	10.660216	8.718900	474.726410	1857.360648	411.272741	6327	23665	7328
gene2418	NC_008261.1	2732648	2733097	-	protein_coding	CPF_RS12010	CPF_2463	.	WP_003457391.	ribose 5-phosph	5.348434	8.750502	6.227557	8.623589	-1.164370	7.513568	7.624145	10.043262	7.404595	7.624145	10.043262	7.404595	159.877831	1133.629653	160.602903	1522	10317	2044
gene2419	NC_008261.1	2733361	2734413	-	protein_coding	CPF_RS12015	CPF_2464	.	WP_003457388.	threonylcarbam	2.430875	6.111385	2.964883	5.950206	-1.219677	5.358980	5.504144	6.785620	5.217645	5.504144	6.785620	5.217645	34.880183	148.431612	31.832077	777	3161	948
gene2420	NC_008261.1	2734436	2735026	-	protein_coding	CPF_RS12020	CPF_2465	.	WP_003452379.	hypothetical pro	2.399090	3.637244	2.518662	3.595303	-1.049840	3.025081	3.060370	4.322857	2.990200	3.060370	4.322857	2.990200	5.518839	28.864357	6.162185	69	345	103
gene2421	NC_008261.1	2735105	2736187	-	protein_coding	CPF_RS12025	CPF_2466	.	WP_003452315.	peptide chain re	1.645933	8.225900	1.741415	8.183296	-1.058011	7.833515	7.874297	8.593203	7.792943	7.874297	8.593203	7.792943	195.758261	465.695451	217.631556	4485	10200	6666
gene2422	NC_008261.1	2736244	2738007	-	protein_coding	CPF_RS12030	CPF_2467	.	WP_003457428.	peptide chain re	-1.252371	5.846866	-1.129145	5.773739	-1.109132	5.936264	6.011450	5.686788	5.862018	6.011450	5.686788	5.862018	49.628198	74.337032	50.090191	1852	2652	2499
gene2423	NC_008261.1	2738033	2738632	-	protein_coding	CPF_RS12035	CPF_2468	.	WP_003452353.	thymidine kinase	-1.317309	5.906836	-1.011945	5.719943	-1.301759	5.915688	6.108977	5.711384	5.728515	6.108977	5.711384	5.728515	53.178812	75.652226	45.140102	675	918	766
gene2424	NC_008261.1	2738788	2738994	+	protein_coding	CPF_RS12040	CPF_2469	.	WP_003452406.	50S ribosomal p	2.176478	12.006812	1.898417	12.109685	1.146470	11.557091	11.458912	12.580908	11.656111	11.458912	12.580908	11.656111	2449.821984	6613.329322	3086.204859	10728	27686	18068
gene2425	NC_008261.1	2739103	2740227	+	protein_coding	CPF_RS12045	CPF_2470	.	WP_003457423.	galactosyldiacyl	6.050654	3.708539	4.139404	4.076321	1.461721	2.891663	2.630765	5.227857	3.178434	2.630765	5.227857	3.178434	3.907658	53.357619	7.134415	93	1214	227
gene2426	NC_008261.1	2740270	2741730	-	protein_coding	CPF_RS12050	CPF_2471	.	WP_003452426.	transcription ter	-1.144007	8.614585	-1.042783	8.548250	-1.097071	8.645093	8.712180	8.518084	8.578523	8.712180	8.518084	8.578523	357.000539	435.672185	374.366551	11034	12873	15469
gene2427	NC_008261.1	2741994	2743601	-	protein_coding	CPF_RS12055	CPF_2472	.	WP_003452392.	CTP synthetase	10.624531	8.746686	12.260718	8.620371	-1.154001	7.102513	7.206587	10.615914	6.999943	7.206587	10.615914	6.999943	122.320086	1786.263864	117.243591	4161	58090	5332
gene2428	NC_008261.1	2743942	2744361	-	protein_coding	CPF_RS12060	CPF_2473	.	WP_003452275.	DUF1934 domai	1.294876	6.776428	1.199426	6.832968	1.079580	6.647590	6.592585	6.965399	6.703055	6.592585	6.965399	6.703055	75.406993	163.642381	93.613937	670	1390	1112
gene2429	NC_008261.1	2744384	2745364	-	protein_coding	CPF_RS12065	CPF_2474	.	WP_011591068.	tRNA 4-thiouric	1.282436	6.754158	1.205891	6.799594	1.063476	6.621342	6.577097	6.935985	6.665885	6.577097	6.935985	6.665885	74.784022	161.089801	90.755245	1552	3196	2518
gene2430	NC_008261.1	2745410	2746039	-	protein_coding	CPF_RS12070	CPF_2475	.	WP_011591069.	stage II sporulat	-1.044053	5.183060	-1.197088	5.280226	1.146578	5.312001	5.214250	5.152055	5.411584	5.214250	5.152055	5.411584	27.986874	50.466212	36.087387	373	643	643
gene2431	NC_008261.1	2746109	2746975	-	protein_coding	CPF_RS12075	CPF_2476	.	WP_003471015.	4-diphosphocyti	1.031182	4.882155	1.039001	4.876678	-1.007583	4.854603	4.860055	4.904355	4.849157	4.860055	4.904355	4.849157	22.135689	42.545145	23.938890	406	746	587
gene2432	NC_008261.1	2747111	2749735	-	protein_coding	CPF_RS12080	CPF_2477	.	WP_011591070.	cyanophycin syr	1.241326	1.600665	1.411467	1.495059	-1.137064	1.356485	1.452302	1.764185	1.266989	1.452302	1.764185	1.266989	1.314558	3.503595	1.239206	73	186	92
gene2433	NC_008261.1	2749722	2750549	-	protein_coding	CPF_RS12085	CPF_2478	.	WP_025648404.	cyanophycinase	-1.321242	1.193693	1.077304	0.954318	-1.423379	1.128397	1.411436	1.009541	0.902116	1.411436	1.009541	0.902116	1.255968	1.433214	0.768647	22	24	18
gene2434	NC_008261.1	2750649	2752211	-	protein_coding	CPF_RS12090	CPF_2479	.	WP_003457329.	DUF3794 domai	-2.152843	7.838225	-1.676260	7.668170	-1.284313	8.228360	8.410839	7.304596	8.049841	8.410839	7.304596	8.049841	288.731424	204.711718	254.720810	9547	6471	11260
gene2435	NC_008261.1	2752571	2752807	-	protein_coding	CPF_RS12095	CPF_2480	.	WP_003462688.	hypothetical pro	-1.544237	11.331146	-1.544237	11.331146	1.000000	11.648928	11.648928	11.022033	11.648928	11.648928	11.022033	11.648928	2870.907901	2601.438477	3077.768116	14394	12469	20630
gene2436	NC_008261.1	2752970	2753857	-	protein_coding	CPF_RS12100	CPF_2481	.	WP_003452445.	sporulation pept	-1.013439	0.650601	-1.381141	0.842361	1.362826	0.854922	0.660302	0.641043	1.106904	0.660302	0.641043	1.106904	0.425856	0.835235	1.035250	8	15	26
gene2437	NC_008261.1	2754056	2755060	-	protein_coding	CPF_RS12105	CPF_2482	.	WP_003457404.	CotS family spor	-5.617838	3.662421	-5.811346	3.679877	1.034445	5.137630	5.113259	2.623244	5.162117	5.113259	2.623244	5.162117	26.292498	7.478385	30.608229	559	152	870
gene2438	NC_008261.1	2755169	2756296	+	protein_coding	CPF_RS12110	CPF_2483	.	WP_011591072.	glycosyltransfer	1.146675	0.240703	1.059222	0.314665	1.082564	0.211040	0.161435	0.358892	0.275888	0.161435	0.358892	0.275888	0.083812	0.394515	0.188073	2	9	6
gene2439	NC_008261.1	2756479	2757507	-	protein_coding	CPF_RS12115	CPF_2484	.	WP_011591073.	CotS family spor	-1.372058	0.531584	-1.492018	0.570037	1.087431	0.865006	0.806655	0.350313	0.927578	0.806655	0.350313	0.927578	0.551254	0.384419	0.790310	12	8	23
gene2440	NC_008261.1	2757523	2758206	-	protein_coding	CPF_RS12120	CPF_2485	.	WP_011591074.	hypothetical pro	1.187550	0.342761	-1.010355	0.495869	1.199847	0.347936	0.240505	0.488493	0.503356	0.240505	0.488493	0.503356	0.138217	0.578315	0.361849	2	8	7
gene2441	NC_008261.1	2758214	2759221	-	protein_coding	CPF_RS12125	CPF_2486	.	WP_011591075.	CotS family spor	1.025707	0.266792	-1.380304	0.463142	1.415787	0.432447	0.249110	0.285729	0.750715	0.249110	0.285729	0.750715	0.140685	0.294321	0.561235	3	6	16
gene2442	NC_008261.1	2759350	2760495	+	protein_coding	CPF_RS12130	CPF_2487	.	WP_011591076.	glycosyltransfer	-1.033438	0.463021	1.049555	0.403508	-1.084650	0.424714	0.487354	0.439903	0.370125	0.487354	0.439903	0.370125	0.288735	0.517758	0.246826	7	12	8
gene2443	NC_008261.1	2760729	2761259	-	protein_coding	CPF_RS12135	CPF_2488	.	WP_003457337.	heptaprenyl diph	-1.139930	0.790619	-1.496794	0.949118	1.313058	1.069282	0.890716	0.701771	1.283646	0.890716	0.701771	1.283646	0.623146	0.931185	1.265156	7	10	19
gene2444	NC_008261.1	2761452	2761823	-	protein_coding	CPF_RS12140	CPF_2489	.	WP_011591077.	hypothetical pro	-1.093719	0.934254	-1.313765	1.050433	1.201191	1.125600	1.001107	0.871865	1.265573	1.001107	0.871865	1.265573	0.762420	1.196272	1.235621	6	9	13
gene2445	NC_008261.1	2761964	2762407	+	protein_coding	CPF_RS12145	CPF_2490	.	WP_003457393.	FMN-binding dor	-2.706996	1.578677	-1.924466	1.411407	-1.406622	2.192890	2.452775	1.016082	1.960541	2.452775	1.016082	1.960541	3.406851	1.447740	2.627943	32	13	33
gene2446	NC_008261.1	2762442	2763470	+	protein_coding	CPF_RS12150	CPF_2491	.	WP_011591078.	FAD:protein FMI	-2.251185	1.103851	-1.764937	0.992644	-1.275505	1.649942	1.834787	0.664102	1.483719	1.834787	0.664102	1.483719	1.929390	0.864943	1.546259	42	18	45
gene2447	NC_008261.1	2763593	2764717	-	protein_coding	CPF_RS12155	CPF_2492	.	WP_011591079.	ethanolamine ut	1.221344	2.282349	1.191806	2.301083	1.024784	2.160254	2.142667	2.431136	2.177986	2.142667	2.431136	2.177986	2.479052	6.285123	3.205772	59	143	102
gene2448	NC_008261.1	2764880	2765209	-	protein_coding	CPF_RS12160	CPF_2493	.	WP_003470977.	PTS sugar trans	-1.548449	2.766436	-1.447691	2.722776	-1.069599	3.050850	3.099771	2.468947	3.002701	3.099771	2.468947	3.002701	5.729704	6.442951	6.214398	40	43	58
gene2449	NC_008261.1	2765439	2766818	-	protein_coding	CPF_RS12165	CPF_2494	.	WP_011591080.	PDZ domain-co	4.754483	9.387850	3.097823	9.729866	1.534782	8.633819	8.330331	10.579619	8.948365	8.330331	10.579619	8.948365	273.070202	1703.948688	484.580965	7972	47556	18913
gene2450	NC_008261.1	2766935	2767954	-	protein_coding	CPF_RS12170	CPF_2495	.	WP_041702724.	alpha/beta hydr	1.715758	4.410620	1.433492	4.550025	1.196907	4.165994	4.038356	4.817201	4.297667	4.038356	4.817201	4.297667	11.817514	39.799101	16.014995	255	821	462
gene2451	NC_008261.1	2767969	2768988	-	protein_coding	CPF_RS12175	CPF_2496	.	WP_003452273.	tRNA (adenosin	1.403184	5.181072	1.776888	4.999338	-1.266325	4.769114	4.942479	5.431183	4.601831	4.942479	5.431183	4.601831	23.217939	61.419563	19.966748	501	1267	576
gene2452	NC_008261.1	2769130	2769762	+	protein_coding	CPF_RS12180	CPF_2497	.	WP_003471106.	RusA family cro	1.433954	3.796883	1.572227	3.725096	-1.096428	3.478736	3.545775	4.065773	3.412964	3.545775	4.065773	3.412964	7.990357	24.293325	8.490336	107	311	152
gene2453	NC_008261.1	2769771	2770331	+	protein_coding	CPF_RS12185	CPF_2498	.	WP_003457357.	acyl-CoA thioes	1.848876	2.418192	1.520972	2.581659	1.215588	2.151392	2.015169	2.901817	2.296823	2.015169	2.901817	2.296823	2.275029	9.430858	3.592502	27	107	57
gene2454	NC_008261.1	2770468	2771430	-	protein_coding	CPF_RS12190	CPF_2499	.	WP_003457394.	FAD-dependent	-1.392250	6.757018	-1.197740	6.651409	-1.162397	6.890536	6.999943	6.522524	6.782840	6.999943	6.522524	6.782840	104.700963	127.388657	99.280833	2133	2481	2704
gene2455	NC_008261.1	2771462	2772034	-	protein_coding	CPF_RS12195	CPF_2500	.	WP_003468263.	sulfurtransferas	-1.479293	6.077776	-1.287413	5.981344	-1.149043	6.265772	6.366790	5.801882	6.166357	6.366790	5.801882	6.166357	65.914091	81.115437	62.385197	799	940	1011
gene2456	NC_008261.1	2772216	2774105	+	protein_coding	CPF_RS12200	CPF_2501	.	WP_011591082.	LTA synthase fa	3.314954	7.158048	4.355202	6.932409	-1.313804	6.145540	6.345568	8.074557	5.951818	6.345568	8.074557	5.951818	64.852515	315.956681	53.354706	2593	12077	2852
gene2457	NC_008261.1	2774242	2775765	-	protein_coding	CPF_RS12205	CPF_2502	.	WP_011591083.	hypothetical pro	-1.744357	6.942106	-2.245802	7.112061	1.287467	7.535110	7.355046	6.552351	7.719582	7.355046	6.552351	7.719582	132.877241	131.336615	204.768887	4284	4048	8826
gene2458	NC_008261.1	2775782	2777197	-	protein_coding	CPF_RS12210	CPF_2503	.	WP_003452521.	sensor histidine	-1.763702	6.274220	-2.317089	6.456015	1.313764	6.890901	6.696860	5.878252	7.090566	6.696860	5.878252	7.090566	81.821261	86.181130	125.075657	2451	2468	5009
gene2459	NC_008261.1	2777378	2778061	-	protein_coding	CPF_RS12215	CPF_2504	.	WP_003452499.	DNA-binding res	-1.546977	7.059024	-1.974204	7.225307	1.276168	7.554624	7.380763	6.751310	7.732581	7.380763	6.751310	7.732581	134.415581	145.590704	207.856201	1945	2014	4021
gene2461	NC_008261.1	2778510	2781421	-	rRNA	CPF_RS12225	CPF_3017	.	.	23S ribosomal R	-1.201447	0.617185	-1.582113	0.760918	1.316840	0.941443	0.763611	0.498838	1.160691	0.763611	0.498838	1.160691	0.503218	0.594302	1.092789	31	35	90
gene2464	NC_008261.1	2781834	2783352	-	rRNA	CPF_RS12240	CPF_3018	.	.	16S ribosomal R	-1.162689	0.113476	-1.153654	0.111049	-1.007832	0.260206	0.265894	0.048429	0.254639	0.265894	0.048429	0.254639	0.155596	0.032552	0.162939	5	1	7
gene2465	NC_008261.1	2783830	2784399	-	protein_coding	CPF_RS12245	CPF_2508	.	WP_003476466.	chromate transp	-1.710689	3.451385	-1.929876	3.528281	1.128128	3.946342	3.860335	3.085757	4.034265	3.860335	3.085757	4.034265	10.283310	11.016893	12.964520	124	127	209
gene2466	NC_008261.1	2784386	2784937	-	protein_coding	CPF_RS12250	CPF_2509	.	WP_003462654.	chromate transp	-1.234946	3.206202	-1.132250	3.145910	-1.090701	3.298815	3.362037	3.057589	3.236781	3.362037	3.057589	3.236781	7.022001	10.659533	7.366205	82	119	115
gene2467	NC_008261.1	2785365	2786078	+	protein_coding	CPF_RS12255	CPF_2510	.	WP_011591084.	DUF4309 domai	-1.296095	5.137651	-1.546354	5.259014	1.193087	5.454005	5.328143	4.953971	5.582842	5.328143	4.953971	5.582842	30.454097	43.905743	40.309852	460	634	814
gene2468	NC_008261.1	2786410	2787861	-	protein_coding	CPF_RS12260	CPF_2512	.	WP_011591085.	aminoacyl-histic	-4.289249	5.338872	-3.352451	5.190623	-1.279437	6.311320	6.491577	4.390852	6.136068	6.491577	4.390852	6.136068	70.612086	30.001265	61.291692	2169	881	2517
gene2479	NC_008261.1	2788953	2789029	-	tRNA	CPF_RS12315	CPF_2523	.	.	tRNA-Arg	2.467556	1.386064	1.267044	2.005113	1.947491	1.273058	0.880020	2.183102	1.841636	0.880020	2.183102	1.841636	0.613897	5.137236	2.295960	1	8	5
gene2480	NC_008261.1	2789105	2789178	-	tRNA	CPF_RS12320	CPF_2524	.	.	tRNA-Gly	2.801571	2.002039	1.120091	2.724163	2.322644	1.942820	1.427816	2.807199	2.643583	1.427816	2.807199	2.643583	1.277569	8.686442	4.778079	2	13	10
gene2487	NC_008261.1	2789713	2789789	-	tRNA	CPF_RS12355	CPF_2531	.	.	tRNA-Arg	2.088546	3.366623	-1.179479	4.056858	2.463396	3.466889	2.877031	3.939530	4.177680	2.877031	3.939530	4.177680	4.911175	21.833254	14.694143	8	34	32
gene2488	NC_008261.1	2789869	2789942	-	tRNA	CPF_RS12360	CPF_2532	.	.	tRNA-Gly	3.033208	1.502651	1.371587	2.263591	2.211458	1.358946	0.902116	2.502960	2.047114	0.902116	2.502960	2.047114	0.638785	6.681878	2.866847	1	10	6
gene2489	NC_008261.1	2789947	2790022	-	tRNA	CPF_RS12365	CPF_2533	.	.	tRNA-Pro	1.255136	1.039427	-1.740943	1.565863	2.185121	1.338272	0.888351	1.216195	2.016064	0.888351	1.216195	2.016064	0.621974	1.951812	2.791404	1	3	6
gene2490	NC_008261.1	2790232	2790702	-	protein_coding	CPF_RS12370	CPF_2534	.	WP_011591086.	metallophosphoe	-1.996927	6.043275	-2.154953	6.093654	1.079134	6.617433	6.562724	5.564942	6.672598	6.562724	5.564942	6.672598	73.966095	68.132460	91.284635	737	649	1216
gene2491	NC_008261.1	2790714	2791328	-	protein_coding	CPF_RS12375	CPF_2535	.	WP_003482252.	non-canonical p	-1.951738	5.091739	-2.476685	5.245727	1.268964	5.766183	5.596917	4.632158	5.940568	5.596917	4.632158	5.940568	37.508597	34.893529	52.892942	488	434	920
gene2492	NC_008261.1	2791330	2792076	-	protein_coding	CPF_RS12380	CPF_2536	.	WP_011591087.	ribonuclease PH	-1.969158	5.499181	-2.091057	5.538685	1.061904	6.052822	6.009650	5.032071	6.096304	6.009650	5.032071	6.096304	49.548130	45.474341	59.118969	783	687	1249
gene2493	NC_008261.1	2792298	2794001	+	protein_coding	CPF_RS12385	CPF_2537	.	WP_041702726.	hypothetical pro	-1.493900	0.860910	-1.234857	0.755758	-1.209775	1.051532	1.197837	0.618753	0.923097	1.197837	0.618753	0.923097	0.970922	0.812491	0.788495	35	28	38
gene2494	NC_008261.1	2794270	2795790	-	protein_coding	CPF_RS12390	CPF_2538	.	WP_011591089.	peptide-binding	1.301098	1.691867	1.164827	1.778893	1.116988	1.590428	1.512623	1.892352	1.672236	1.512623	1.892352	1.672236	1.398522	3.901057	1.882959	45	120	81
gene2496	NC_008261.1	2797536	2797859	+	protein_coding	CPF_RS12400	CPF_2540	.	WP_003459451.	hypothetical pro	4.535326	4.817723	5.929981	4.569227	-1.307509	3.650488	3.849019	6.030225	3.462198	3.849019	6.030225	3.462198	10.212666	94.008253	8.621188	70	616	79
gene2497	NC_008261.1	2797896	2799884	-	protein_coding	CPF_RS12405	CPF_2541	.	WP_011591090.	DNA ligase (NAD	-1.542440	5.595651	-1.629077	5.632807	1.056169	5.956273	5.916983	5.291769	5.995824	5.916983	5.291769	5.995824	45.487625	55.561380	54.876558	1914	2235	3087
gene2498	NC_008261.1	2799922	2802177	-	protein_coding	CPF_RS12410	CPF_2542	.	WP_011591091.	DNA helicase Pc	-1.675521	5.260386	-1.600831	5.229648	-1.046657	5.612859	5.645850	4.901240	5.580061	5.645850	4.901240	5.580061	38.323108	42.388462	40.106633	1829	1934	2559
gene2499	NC_008261.1	2802389	2803177	-	protein_coding	CPF_RS12415	CPF_2543	.	WP_011591092.	hypothetical pro	-2.769258	8.304626	-3.477617	8.453694	1.255794	9.234654	9.071816	7.602317	9.400415	9.071816	7.602317	9.400415	459.400231	245.725439	679.057631	7668	3921	15153
gene2500	NC_008261.1	2803194	2803742	-	protein_coding	CPF_RS12420	CPF_2544	.	WP_011591093.	hypothetical pro	-3.261013	7.842135	-3.788331	7.938545	1.161704	8.848474	8.741013	7.035693	8.957256	8.741013	7.035693	8.957256	368.000392	170.133523	488.954975	4274	1889	7592
gene2501	NC_008261.1	2803744	2804415	-	protein_coding	CPF_RS12425	CPF_2545	.	WP_011591094.	anchor protein	-4.365664	6.645344	-4.449091	6.656978	1.019110	7.806586	7.792943	5.666742	7.820253	7.792943	5.666742	7.820253	183.030779	73.359467	221.459679	2602	997	4209
gene2502	NC_008261.1	2804968	2805687	-	protein_coding	CPF_RS12430	CPF_2546	.	WP_003462288.	rRNA pseudouri	2.018277	6.516196	1.711845	6.643334	1.179007	6.146932	6.029294	7.042418	6.266866	6.029294	7.042418	6.266866	50.224433	171.206427	66.541381	765	2493	1355
gene2503	NC_008261.1	2806061	2808121	-	protein_coding	CPF_RS12435	CPF_2547	.	WP_011010854.	triple tyrosine m	-1.900492	5.961618	-1.819975	5.932653	-1.044240	6.411468	6.442771	5.516398	6.380317	6.442771	5.516398	6.380317	68.921162	65.687954	72.860020	3005	2738	4247
gene2504	NC_008261.1	2808494	2808943	-	protein_coding	CPF_RS12440	CPF_2548	.	WP_003454468.	YaiI/YqxD family	-1.588290	6.024853	-1.194522	5.827158	-1.329645	6.158878	6.367826	5.700352	5.956785	6.367826	5.700352	5.956785	65.967988	75.487406	53.508110	628	687	681
gene2505	NC_008261.1	2809141	2810493	-	protein_coding	CPF_RS12445	CPF_2549	.	WP_003462349.	glucose-6-phos	1.778985	10.161446	1.332795	10.376169	1.334778	9.960533	9.754411	10.585466	10.171011	9.754411	10.585466	10.171011	766.068364	1707.290525	1225.736444	21927	46717	46904
gene2506	NC_008261.1	2810523	2810732	-	protein_coding	CPF_RS12450	CPF_2550	.	WP_003454512.	hypothetical pro	3.020256	6.040909	2.252835	6.277486	1.340647	5.503370	5.295966	6.890637	5.718896	5.295966	6.890637	5.718896	29.487511	158.226878	44.954894	131	672	267
gene2507	NC_008261.1	2810934	2811902	-	protein_coding	CPF_RS12455	CPF_2551	.	WP_003454313.	dipeptide ABC tr	22.025896	5.253642	15.423598	5.628626	1.428065	3.725163	3.476990	7.938118	3.991051	3.476990	7.938118	3.991051	7.658822	292.389064	12.588684	157	5730	345
gene2508	NC_008261.1	2811903	2812880	-	protein_coding	CPF_RS12460	CPF_2552	.	WP_003454477.	ABC transporter	26.672529	4.905500	19.757304	5.239087	1.350009	3.288145	3.078779	7.816062	3.511748	3.078779	7.816062	3.511748	5.655007	274.379239	9.002135	117	5427	249
gene2509	NC_008261.1	2812894	2813808	-	protein_coding	CPF_RS12465	CPF_2553	.	WP_003454342.	ABC transporter	24.391511	4.685558	20.659167	4.874173	1.180663	3.034735	2.917300	7.525607	3.156897	2.917300	7.525607	3.156897	5.011142	233.017178	7.032914	97	4312	182
gene2510	NC_008261.1	2813823	2814788	-	protein_coding	CPF_RS12470	CPF_2554	.	WP_003454494.	ABC transporter	29.474086	4.509403	21.785976	4.861566	1.352893	2.896683	2.686853	7.568228	3.122900	2.686853	7.568228	3.122900	4.061506	239.858680	6.808020	83	4686	186
gene2511	NC_008261.1	2814863	2816605	-	protein_coding	CPF_RS12475	CPF_2555	.	WP_003459416.	peptide ABC tra	30.469048	6.459040	19.221792	6.924723	1.585130	4.769396	4.448658	9.377930	5.113259	4.448658	9.377930	5.113259	16.407564	762.539171	29.738674	605	26880	1466
gene2512	NC_008261.1	2817237	2818529	+	protein_coding	CPF_RS12480	CPF_2556	.	WP_003479149.	MFS transporter	2.271831	2.573673	2.155102	2.621031	1.054164	2.086640	2.048938	3.232793	2.125037	2.048938	3.232793	2.125037	2.339740	12.466638	3.117391	64	326	114
gene2513	NC_008261.1	2818882	2820411	-	protein_coding	CPF_RS12485	CPF_2557	.	WP_003460104.	GMP synthase (	5.371664	10.965980	14.199229	10.152829	-2.643358	9.091960	9.820144	12.245513	8.417773	9.820144	12.245513	8.417773	806.556900	5628.268368	346.922238	26106	174155	15012
gene2514	NC_008261.1	2820518	2821972	-	protein_coding	CPF_RS12490	CPF_2558	.	WP_003454518.	IMP dehydrogen	2.729459	10.102100	8.387916	9.191024	-3.073106	8.555633	9.403725	10.852340	7.784028	9.403725	10.852340	7.784028	585.758827	2250.485081	216.447948	18030	66223	8907
gene2515	NC_008261.1	2822682	2825303	-	protein_coding	CPF_RS12495	CPF_2559	.	WP_011591095.	hypothetical pro	1.211054	3.365499	1.162910	3.395850	1.041400	3.259331	3.230200	3.506464	3.288725	3.230200	3.506464	3.288725	6.400027	15.784217	7.699959	355	837	571
gene2516	NC_008261.1	2825327	2825887	-	protein_coding	CPF_RS12500	CPF_2560	.	WP_011591096.	prepilin-type cle	1.715925	0.966806	1.554201	1.067330	1.104056	0.720699	0.652821	1.431807	0.795634	0.652821	1.431807	0.795634	0.421302	2.467888	0.630263	5	28	10
gene2517	NC_008261.1	2825913	2828021	-	protein_coding	CPF_RS12505	CPF_2561	.	WP_011591097.	type II secretion	1.780499	1.853724	1.654019	1.918984	1.076469	1.535953	1.483719	2.316001	1.590025	1.483719	2.316001	1.590025	1.367223	5.720626	1.743580	61	244	104
gene2518	NC_008261.1	2828026	2829027	-	protein_coding	CPF_RS12510	CPF_2562	.	WP_011591098.	type II secretion	1.582196	1.280274	1.591475	1.274815	-1.005865	0.987169	0.991397	1.653325	0.982960	0.991397	1.653325	0.982960	0.754811	3.158221	0.846893	16	64	24
gene2519	NC_008261.1	2829048	2830265	-	protein_coding	CPF_RS12515	CPF_2563	.	WP_011591099.	hypothetical pro	1.480732	2.920576	1.494123	2.913413	-1.009043	2.644613	2.651114	3.217426	2.638127	2.651114	3.217426	2.638127	3.958576	12.341177	4.731789	102	304	163
gene2520	NC_008261.1	2830255	2830821	-	protein_coding	CPF_RS12520	CPF_2564	.	WP_003460111.	fimbrial protein	1.673471	2.445246	1.514087	2.527844	1.105268	2.172871	2.101872	2.844716	2.246268	2.101872	2.844716	2.246268	2.417692	8.982236	3.429767	29	103	55
gene2521	NC_008261.1	2830836	2831990	-	protein_coding	CPF_RS12525	CPF_2565	.	WP_041702727.	pilus assembly p	1.854967	2.696147	1.752366	2.744106	1.058550	2.327723	2.287040	3.178434	2.369129	2.287040	3.178434	2.369129	2.864852	11.730023	3.795987	70	274	124
gene2522	NC_008261.1	2832064	2832564	-	protein_coding	CPF_RS12530	CPF_2566	.	WP_003460093.	prepilin-type N-	1.405597	6.516438	1.182056	6.644912	1.189112	6.399196	6.275473	6.766657	6.525358	6.275473	6.766657	6.525358	61.705820	146.857282	82.219194	654	1488	1165
gene2523	NC_008261.1	2832577	2833041	-	protein_coding	CPF_RS12535	CPF_2567	.	WP_003460106.	prepilin-type N-	1.362994	7.091368	1.304162	7.124139	1.045111	6.903251	6.871496	7.318275	6.935152	6.871496	7.318275	6.935152	94.235142	207.460107	112.308482	927	1951	1477
gene2524	NC_008261.1	2833099	2833617	-	protein_coding	CPF_RS12540	CPF_2568	.	WP_003460140.	type II secretion	1.517193	3.599590	1.153595	3.808368	1.315188	3.503490	3.311426	3.912831	3.706695	3.311426	3.912831	3.706695	6.830933	21.340812	10.355266	75	224	152
gene2525	NC_008261.1	2833642	2834847	-	protein_coding	CPF_RS12545	CPF_2569	.	WP_003460117.	type II secretion	1.243955	3.732258	1.051476	3.856668	1.183056	3.697383	3.578112	3.893046	3.820630	3.578112	3.893046	3.820630	8.231104	21.032957	11.287517	210	513	385
gene2526	NC_008261.1	2834874	2836562	-	protein_coding	CPF_RS12550	CPF_2570	.	WP_003462253.	type II secretion	1.272176	3.402013	1.128502	3.491798	1.127314	3.318112	3.232793	3.580091	3.405683	3.232793	3.580091	3.405683	6.409025	16.686893	8.436463	229	570	403
gene2527	NC_008261.1	2836632	2837375	-	protein_coding	CPF_RS12555	CPF_2571	.	WP_003454467.	prepilin peptidas	1.156936	1.563549	1.021334	1.656938	1.132769	1.549246	1.461927	1.672236	1.641781	1.461927	1.672236	1.641781	1.334235	3.256518	1.853432	21	49	39
gene2528	NC_008261.1	2837652	2838074	-	protein_coding	CPF_RS12560	CPF_2572	.	WP_003460119.	prepilin-type cle	-2.943383	3.019563	-1.910838	2.767588	-1.540363	3.571898	3.897102	2.339626	3.273832	3.897102	2.339626	3.273832	10.616206	5.844669	7.606520	95	50	91
gene2529	NC_008261.1	2838740	2840359	-	protein_coding	CPF_RS12565	CPF_2573	.	WP_003454479.	molecular chape	-1.312708	8.762378	1.118182	8.487343	-1.467846	8.679584	8.960849	8.568303	8.407148	8.960849	8.568303	8.407148	421.374608	459.968953	344.301859	14441	15070	15775
gene2530	NC_008261.1	2840414	2840698	-	protein_coding	CPF_RS12570	CPF_2574	.	WP_003454296.	molecular chape	1.100992	8.285150	1.627577	7.995764	-1.478282	7.929066	8.216039	8.354843	7.652117	8.216039	8.354843	7.652117	252.604540	380.299689	193.289208	1523	2192	1558
gene2532	NC_008261.1	2841870	2842175	+	protein_coding	CPF_RS12580	CPF_2576	.	WP_003454503.	HNH endonucle	-2.090605	4.644463	-1.329066	4.343230	-1.572987	4.869083	5.206788	4.142868	4.553281	5.206788	4.142868	4.553281	27.805915	25.207713	19.412116	180	156	168
gene2533	NC_008261.1	2842298	2843236	-	protein_coding	CPF_RS12585	CPF_2577	.	WP_003454265.	DNA-3-methyla	-1.244252	8.372194	-1.112598	8.292648	-1.118331	8.450259	8.531318	8.216039	8.369971	8.531318	8.216039	8.369971	311.156773	348.912172	332.641796	6181	6626	8834
gene2534	NC_008261.1	2843246	2843650	-	protein_coding	CPF_RS12590	CPF_2578	.	WP_003460091.	hypothetical pro	-1.061533	7.871765	1.193767	7.699988	-1.267223	7.742238	7.914958	7.828809	7.573288	7.914958	7.828809	7.573288	203.786460	275.554061	182.550919	1746	2257	2091
gene2535	NC_008261.1	2843892	2844392	-	protein_coding	CPF_RS12595	CPF_2579	.	WP_003460107.	signal peptidase	1.121155	10.055172	1.283048	9.956593	-1.144399	9.875244	9.973018	10.138003	9.778428	9.973018	10.138003	9.778428	864.353242	1212.559520	900.670687	9161	12286	12762
gene2536	NC_008261.1	2844534	2846234	-	protein_coding	CPF_RS12600	CPF_2580	.	WP_003460100.	ABC transporter	1.953973	4.567632	2.056745	4.526352	-1.052596	4.072771	4.109914	5.076325	4.035963	4.109914	5.076325	4.035963	12.394147	47.120401	12.970756	446	1621	624
gene2537	NC_008261.1	2846494	2847342	-	protein_coding	CPF_RS12605	CPF_2581	.	WP_003454476.	3-hydroxybutyry	1.041295	9.140463	1.002438	9.167943	1.038763	9.138713	9.111321	9.169699	9.166187	9.111321	9.169699	9.166187	472.310809	665.510356	577.177269	8483	11427	13859
gene2538	NC_008261.1	2847456	2848463	-	protein_coding	CPF_RS12610	CPF_2582	.	WP_003454260.	electron transfe	1.017865	8.694351	-1.106094	8.779569	1.125854	8.766680	8.681588	8.707133	8.852607	8.681588	8.707133	8.852607	349.554554	499.805822	443.375362	7454	10189	12640
gene2539	NC_008261.1	2848485	2849264	-	protein_coding	CPF_RS12615	CPF_2583	.	WP_003460115.	electron transfe	1.104471	8.194214	1.035385	8.241084	1.066726	8.169311	8.122850	8.266206	8.216039	8.122850	8.266206	8.216039	240.531857	357.278319	295.282811	3969	5636	6514
gene2540	NC_008261.1	2849283	2850422	-	protein_coding	CPF_RS12620	CPF_2584	.	WP_003454427.	acyl-CoA dehyd	1.125899	8.016849	1.069536	8.054206	1.052699	7.968727	7.931767	8.102844	8.005860	7.931767	8.102844	8.005860	207.283338	324.911607	247.256349	4999	7491	7972
gene2541	NC_008261.1	2850469	2851251	-	protein_coding	CPF_RS12625	CPF_2585	.	WP_003467423.	crotonase	1.252567	7.661381	1.195255	7.695812	1.047950	7.534367	7.500659	7.825546	7.568228	7.500659	7.825546	7.568228	146.519060	275.204635	181.440062	2427	4358	4018
gene2542	NC_008261.1	2851514	2852152	-	protein_coding	CPF_RS12630	CPF_2586	.	WP_003454253.	transcriptional r	-1.402268	6.463061	-1.420522	6.472039	1.013018	6.720865	6.711542	6.223780	6.730201	6.711542	6.223780	6.730201	83.073978	104.772688	95.338746	1123	1354	1723
gene2543	NC_008261.1	2852618	2854834	-	protein_coding	CPF_RS12635	CPF_2587	.	WP_011591103.	cadmium-transl	-1.088251	7.243333	-1.494193	7.466650	1.373022	7.529801	7.304596	7.182584	7.761950	7.304596	7.182584	7.761950	129.081152	188.349400	213.007910	6054	8445	13356
gene2544	NC_008261.1	2854850	2855209	-	protein_coding	CPF_RS12640	CPF_2588	.	WP_003454170.	ArsR family tran	-1.184392	8.729681	-1.068056	8.655824	-1.108924	8.777711	8.852607	8.608461	8.703448	8.852607	8.608461	8.703448	397.462380	470.422793	405.632333	3027	3425	4130
gene2545	NC_008261.1	2855344	2855964	-	protein_coding	CPF_RS12645	CPF_2589	.	WP_003454289.	GntR family tran	2.536590	7.287262	6.179283	6.545317	-2.436059	5.969960	6.646684	7.989575	5.362135	6.646684	7.989575	5.362135	79.620737	299.462202	35.073098	1046	3761	616
gene2546	NC_008261.1	2856054	2856632	-	protein_coding	CPF_RS12650	CPF_2590	.	WP_003457835.	hypothetical pro	2.169640	5.546444	5.515426	4.744216	-2.542093	4.290313	5.015787	6.133242	3.669770	5.015787	6.133242	3.669770	24.655538	99.574989	10.076052	302	1166	165
gene2547	NC_008261.1	2857017	2857583	-	protein_coding	CPF_RS12655	.	.	WP_003454445.	DUF3867 domai	-1.614401	6.580589	-1.528706	6.543150	-1.056057	6.895696	6.935152	6.244153	6.856465	6.935152	6.244153	6.856465	98.791914	105.781086	104.763799	1185	1213	1680
gene2548	NC_008261.1	2857812	2858234	-	protein_coding	CPF_RS12660	CPF_2592	.	WP_078209998.	mannose-6-pho	1.530356	0.782614	1.389190	0.879042	1.101618	0.599478	0.533717	1.147584	0.673341	0.533717	1.147584	0.673341	0.335249	1.753401	0.501529	3	15	6
gene2550	NC_008261.1	2858899	2859681	-	protein_coding	CPF_RS12670	CPF_2594	.	WP_011591107.	hypothetical pro	1.180698	2.460102	1.237400	2.424318	-1.048024	2.309114	2.343198	2.582838	2.275527	2.343198	2.582838	2.275527	3.018522	7.135871	3.522231	50	113	78
gene2551	NC_008261.1	2859714	2860430	-	protein_coding	CPF_RS12675	CPF_2595	.	WP_011591108.	M48 family pepti	-1.185868	3.646903	-1.171598	3.638450	-1.012180	3.763204	3.771947	3.526003	3.754482	3.771947	3.526003	3.754482	9.691350	15.999231	10.701030	147	232	217
gene2552	NC_008261.1	2860476	2863655	-	protein_coding	CPF_RS12680	CPF_2596	.	WP_011591109.	type I restrictior	-1.382819	4.211052	-1.275206	4.155398	-1.084389	4.392514	4.451344	3.983732	4.334462	4.451344	3.983732	4.334462	16.440466	22.701576	16.533655	1106	1460	1487
gene2553	NC_008261.1	2864026	2865114	-	protein_coding	CPF_RS12685	CPF_2597	.	WP_011591110.	hypothetical pro	1.166760	0.539811	-1.074986	0.712662	1.254250	0.580763	0.439903	0.662410	0.766727	0.439903	0.662410	0.766727	0.260441	0.862692	0.584426	6	19	18
gene2554	NC_008261.1	2865161	2866414	-	protein_coding	CPF_RS14690	CPF_2598	.	WP_011591111.	restriction endo	-1.392934	4.434893	-1.630364	4.541197	1.170453	4.792585	4.680396	4.202268	4.907462	4.680396	4.202268	4.907462	19.488531	26.418463	25.122635	517	670	891
gene2555	NC_008261.1	2866431	2867948	-	protein_coding	CPF_RS12695	CPF_2599	.	WP_011591112.	type I restrictior	-1.624051	5.740253	-1.675053	5.761202	1.031404	6.122964	6.100700	5.401102	6.145309	6.100700	5.401102	6.145309	52.906340	60.162435	61.352041	1699	1847	2634
gene2558	NC_008261.1	2869975	2871759	+	protein_coding	CPF_RS12710	CPF_2603	.	WP_080503690.	hypothetical pro	-3.225437	2.532268	-2.200867	2.325123	-1.465530	3.226731	3.514200	1.824705	2.962777	3.514200	1.824705	2.962777	7.838620	3.684204	6.041526	296	133	305
gene2559	NC_008261.1	2871704	2873401	+	protein_coding	CPF_RS12715	CPF_2604	.	WP_041702729.	recombinase fan	-3.571767	2.207013	-2.522887	2.032866	-1.415746	3.047680	3.308761	1.472123	2.807199	3.308761	1.472123	2.807199	6.820473	2.591687	5.393207	245	89	259
gene2560	NC_008261.1	2873655	2875259	+	protein_coding	CPF_RS12720	CPF_2606	.	WP_011591119.	serine recombin	-2.497659	3.096363	-2.129788	3.001915	-1.172726	3.709558	3.826271	2.505695	3.596405	3.826271	2.505695	3.596405	10.043046	6.685209	9.516861	341	217	432
gene2561	NC_008261.1	2875271	2876059	+	protein_coding	CPF_RS12725	CPF_2608	.	WP_011591120.	zinc metalloprot	-1.392235	0.920398	-1.207866	0.837367	-1.152641	1.082237	1.189549	0.712146	0.984607	1.189549	0.712146	0.984607	0.958582	0.940036	0.851455	16	15	19
gene2563	NC_008261.1	2877009	2878388	-	protein_coding	CPF_RS12735	CPF_2609	.	WP_003467493.	glycoside hydrol	-2.770427	5.182208	-3.697078	5.359862	1.334479	6.173765	5.969134	4.499025	6.385411	5.969134	4.499025	6.385411	47.783860	31.996513	72.995885	1395	893	2849
gene2564	NC_008261.1	2879085	2880461	-	protein_coding	CPF_RS12740	CPF_2610	.	WP_011591122.	aldehyde dehydr	-3.427480	4.996859	-3.200025	4.955184	-1.071079	5.914085	5.963824	4.186676	5.864760	5.963824	4.186676	5.864760	47.476025	26.320875	50.122288	1383	733	1952
gene2565	NC_008261.1	2880612	2881322	-	protein_coding	CPF_RS12745	CPF_2611	.	WP_011591123.	DUF3298/DUF4	1.333426	0.617848	1.032056	0.836281	1.292009	0.601261	0.444214	0.859352	0.813830	0.444214	0.859352	0.813830	0.265936	1.182251	0.646485	4	17	13
gene2566	NC_008261.1	2881431	2882372	+	protein_coding	CPF_RS12750	CPF_2612	.	WP_003462281.	alpha/beta hydr	1.162149	3.387430	1.291406	3.307950	-1.111223	3.203789	3.280766	3.497561	3.128618	3.280766	3.497561	3.128618	6.674010	15.694611	6.831334	133	299	182
gene2567	NC_008261.1	2882619	2883515	-	protein_coding	CPF_RS12755	CPF_2613	.	WP_003467491.	23S rRNA pseu	1.939878	5.431994	1.691262	5.538961	1.147000	5.072968	4.975000	5.930966	5.172866	4.975000	5.930966	5.172866	23.714074	88.528556	30.864151	450	1606	783
gene2568	NC_008261.1	2883537	2884550	-	protein_coding	CPF_RS12760	CPF_2614	.	WP_003467523.	hypothetical pro	1.709478	4.429056	1.551208	4.504876	1.102030	4.128622	4.059134	4.832690	4.199298	4.059134	4.832690	4.199298	11.934057	40.473468	14.959062	256	830	429
gene2569	NC_008261.1	2884611	2885288	-	protein_coding	CPF_RS12765	CPF_2615	.	WP_003467493.	class D sortase	1.352915	6.979435	1.174133	7.084131	1.152267	6.866281	6.764805	7.200875	6.969279	6.764805	7.200875	6.969279	87.219526	190.928266	115.356068	1251	2618	2212
gene2570	NC_008261.1	2885638	2886108	+	protein_coding	CPF_RS12770	CPF_2616	.	WP_003457926.	2-C-methyl-D-	-3.006606	6.440314	-2.649001	6.359028	-1.134996	7.191226	7.283150	5.695014	7.100463	7.283150	5.695014	7.100463	127.458535	74.956204	125.741582	1270	714	1675
gene2571	NC_008261.1	2886284	2887597	-	protein_coding	CPF_RS12775	CPF_2617	.	WP_003457854.	sodium:proton a	3.513220	6.575205	5.357458	6.216189	-1.524942	5.418068	5.730988	7.543782	5.122233	5.730988	7.543782	5.122233	40.578860	234.924467	29.868446	1128	6243	1110
gene2572	NC_008261.1	2887935	2888777	-	protein_coding	CPF_RS12780	CPF_2618	.	WP_003457860.	pyridoxamine kin	7.094039	4.966622	3.798352	5.531260	1.867663	4.176870	3.750490	6.577097	4.651724	3.750490	6.577097	4.651724	9.532514	134.025955	20.803630	170	2285	496
gene2573	NC_008261.1	2888912	2888987	-	tRNA	CPF_RS12785	CPF_2619	.	.	tRNA-Lys	1.931761	1.805956	1.962532	1.791110	-1.015929	1.380963	1.392410	2.342326	1.369611	1.392410	2.342326	1.369611	1.243949	5.855435	1.395702	2	9	3
gene2574	NC_008261.1	2889032	2889105	-	tRNA	CPF_RS12790	CPF_2620	.	.	tRNA-Gly	-2.164390	3.641698	-1.259474	3.289269	-1.718488	3.830597	4.241026	3.127065	3.459887	4.241026	3.127065	3.459887	13.414475	11.359193	8.600542	21	17	18
gene2575	NC_008261.1	2889171	2889245	-	tRNA	CPF_RS12795	CPF_2621	.	.	tRNA-Gln	-3.029532	6.346297	-2.246825	6.153215	-1.348361	6.977078	7.196012	5.596917	6.764805	7.196012	5.596917	6.764805	121.641608	69.883538	97.587478	193	106	207
gene2576	NC_008261.1	2889249	2889325	-	tRNA	CPF_RS12800	CPF_2622	.	.	tRNA-Arg	-2.720627	7.084041	-2.296555	6.972758	-1.184656	7.719504	7.842706	6.398767	7.598238	7.842706	6.398767	7.598238	190.921911	116.872126	185.972750	311	182	405
gene2577	NC_008261.1	2889451	2890143	-	protein_coding	CPF_RS12805	CPF_2623	.	WP_003454455.	hypothetical pro	-2.522761	9.453115	-2.788081	9.520099	1.105171	10.216034	10.144155	8.809151	10.288424	10.144155	8.809151	10.288424	983.121650	536.199039	1327.779105	14413	7515	26024
gene2578	NC_008261.1	2890333	2890737	+	protein_coding	CPF_RS12810	CPF_2624	.	WP_003454383.	HNH endonucle	-1.210606	3.446535	1.335763	3.095558	-1.617083	3.221859	3.587156	3.311426	2.893762	3.587156	3.311426	2.893762	8.286849	13.185573	5.674706	71	108	65
gene2579	NC_008261.1	2890796	2892205	-	protein_coding	CPF_RS12815	CPF_2625	.	WP_003467540.	SGNH/GDSL hy	-4.202808	6.239193	-7.170028	6.557689	1.706009	7.735967	7.360244	5.288891	8.130870	7.360244	5.288891	8.130870	133.462475	55.512666	271.853699	3981	1583	10841
gene2585	NC_008261.1	2893473	2895404	+	protein_coding	CPF_RS12845	CPF_2631	.	WP_003457830.	threonine--tRN	1.661034	8.296088	2.313844	8.042319	-1.393014	7.695300	7.938118	8.670200	7.459908	7.938118	8.670200	7.459908	208.262272	488.495798	169.559964	8512	19087	9265
gene2586	NC_008261.1	2895873	2897567	-	protein_coding	CPF_RS12850	CPF_2632	.	WP_003457828.	M3 family oligor	-2.628598	9.386445	-1.751343	9.110419	-1.500905	9.812158	10.109445	8.715151	9.523613	10.109445	8.715151	9.523613	961.520329	501.372668	728.724931	34478	17187	34934
gene2587	NC_008261.1	2897663	2898241	-	protein_coding	CPF_RS12855	CPF_2633	.	WP_004459316.	5-formyltetrahy	-2.264971	6.167743	-1.922816	6.059413	-1.177945	6.666437	6.785620	5.606128	6.549348	6.785620	5.606128	6.549348	88.335405	70.197805	82.990027	1082	822	1359
gene2588	NC_008261.1	2898467	2899279	-	protein_coding	CPF_RS12860	CPF_2634	.	WP_003462312.	class I SAM-dep	-1.065414	3.321291	-1.106910	3.348365	1.038948	3.394762	3.367312	3.275898	3.422435	3.367312	3.275898	3.422435	7.035273	12.772004	8.524139	121	210	196
gene2589	NC_008261.1	2899683	2901515	+	protein_coding	CPF_RS12865	CPF_2636	.	WP_003457837.	glutamine--fruc	-4.161258	4.671100	-10.368576	5.173422	2.491692	6.436461	5.811501	3.754482	7.128627	5.811501	3.754482	7.128627	42.447633	18.882777	126.944661	1646	700	6581
gene2590	NC_008261.1	2902307	2902831	+	protein_coding	CPF_RS12870	CPF_2637	.	WP_003457864.	rubrerythrin	1.460809	0.670179	1.119483	0.912136	1.304896	0.613024	0.450411	0.997179	0.834346	0.450411	0.997179	0.834346	0.270115	1.412740	0.673482	3	15	10
gene2591	NC_008261.1	2902981	2904327	-	protein_coding	CPF_RS12875	CPF_2638	.	WP_003457908.	phosphoglucosa	5.215086	8.217686	10.513999	7.610898	-2.016074	6.587085	7.112248	9.494939	6.100700	7.112248	9.494939	6.100700	114.613214	810.663134	59.244628	3266	22084	2257
gene2592	NC_008261.1	2904800	2906092	+	protein_coding	CPF_RS12880	CPF_2639	.	WP_003457833.	DUF349 domain	1.705636	6.919071	1.388725	7.074093	1.228204	6.691260	6.544628	7.314938	6.841177	6.544628	7.314938	6.841177	72.495374	207.420385	104.405268	1983	5424	3818
gene2595	NC_008261.1	2908599	2909281	-	protein_coding	CPF_RS12895	CPF_2642	.	WP_003454391.	DUF1540 domai	-1.903698	7.822036	-1.598746	7.702445	-1.190745	8.173310	8.300213	7.371408	8.048348	8.300213	7.371408	8.048348	269.647421	212.710661	254.620506	1814	1368	2290
gene2596	NC_008261.1	2908982	2909281	-	protein_coding	CPF_RS12900	CPF_2643	.	WP_003454163.	hypothetical pro	-1.487618	6.859527	-1.509822	6.869769	1.014926	7.162688	7.152009	6.579005	7.173383	7.152009	6.579005	7.173383	118.017571	134.163207	130.823794	749	814	1110
gene2597	NC_008261.1	2909421	2910260	-	protein_coding	CPF_RS12905	CPF_2644	.	WP_003454256.	transcriptional r	-2.386640	5.961427	-2.040708	5.858854	-1.169516	6.507915	6.621851	5.366870	6.395939	6.621851	5.366870	6.395939	77.151483	58.981894	73.577861	1371	1002	1748
gene2598	NC_008261.1	2910377	2912395	-	protein_coding	CPF_RS12910	CPF_2645	.	WP_003467507.	glycogen debran	-20.626719	5.026609	-15.447624	4.887894	-1.335268	7.451999	7.663482	3.297039	7.246352	7.663482	3.297039	7.246352	165.152535	13.004345	138.261362	7054	531	7895
gene2599	NC_008261.1	2912754	2915117	-	protein_coding	CPF_RS12915	CPF_2646	.	WP_011591125.	glycogen/starch	-30.006561	6.397423	-18.875987	6.163265	-1.589668	8.964809	9.305405	4.398199	8.636679	9.305405	4.398199	8.636679	549.624367	30.077497	390.266754	27487	1438	26093
gene2600	NC_008261.1	2915110	2916603	-	protein_coding	CPF_RS12920	CPF_2647	.	WP_011591126.	4-alpha-glucano	-22.602908	5.663991	-16.555531	5.509413	-1.365278	8.115755	8.343459	3.845023	7.894264	8.343459	3.845023	7.894264	275.836908	20.188754	232.499900	8718	610	9824
gene2601	NC_008261.1	2916625	2918772	-	protein_coding	CPF_RS12925	CPF_2648	.	WP_003457922.	DUF5110 domai	-13.429009	5.905487	-12.321502	5.859872	-1.089884	8.006900	8.069228	4.321947	7.945053	8.069228	4.321947	7.945053	233.489425	28.820422	240.360490	10610	1252	14602
gene2602	NC_008261.1	2918796	2919809	-	protein_coding	CPF_RS12930	CPF_2649	.	WP_003457851.	LacI family trans	-11.425969	5.062583	-11.457853	5.064013	1.002790	7.117978	7.115968	3.601723	7.119988	7.115968	3.601723	7.119988	114.865301	17.067125	126.716154	2464	350	3634
gene2603	NC_008261.1	2920104	2920997	-	protein_coding	CPF_RS12935	CPF_2650	.	WP_003454394.	sugar ABC trans	-14.833479	7.999641	-9.857576	7.772154	-1.484491	9.876986	10.166081	6.294880	9.596113	10.166081	6.294880	9.596113	1004.250733	108.847349	782.023964	18993	1968	19773
gene2604	NC_008261.1	2920999	2921952	-	protein_coding	CPF_RS12940	CPF_2651	.	WP_003457841.	sugar ABC trans	-12.648197	7.372949	-9.522574	7.211035	-1.328233	9.220169	9.427196	5.766336	9.017688	9.427196	5.766336	9.017688	592.709013	78.937217	517.468923	11962	1523	13962
gene2605	NC_008261.1	2922039	2923265	-	protein_coding	CPF_RS12945	CPF_2652	.	WP_003457886.	maltose ABC tra	-12.755711	10.107448	-9.045064	9.898310	-1.410240	11.858917	12.109479	8.436408	11.613539	12.109479	8.436408	11.613539	3904.961141	401.007453	3008.927080	101362	9951	104417
gene2606	NC_008261.1	2923723	2924664	-	protein_coding	CPF_RS12950	CPF_2653	.	WP_003467462.	hypothetical pro	-6.038145	5.199449	-9.860635	5.468844	1.633057	7.000695	6.655842	4.061736	7.363417	6.655842	4.061736	7.363417	79.787036	24.250537	154.305565	1590	462	4111
gene2607	NC_008261.1	2924699	2925808	-	protein_coding	CPF_RS12955	CPF_2654	.	WP_011591127.	ABC transporter	-9.268651	8.910430	-15.494283	9.215037	1.671687	11.024640	10.660216	7.447857	11.401521	10.660216	7.447857	11.401521	1367.169232	222.372910	2618.482664	32104	4992	82203
gene2608	NC_008261.1	2926027	2927745	-	protein_coding	CPF_RS12960	CPF_2655	.	WP_011591128.	4Fe-4S dicluste	2.849857	8.735564	3.056409	8.680362	-1.072478	7.962089	8.012723	9.523613	7.911775	8.012723	9.523613	7.911775	225.955813	821.912233	233.990767	8217	28574	11376
gene2609	NC_008261.1	2927971	2929041	-	protein_coding	CPF_RS12965	CPF_2656	.	WP_003454444.	butyrate kinase	1.784302	8.956232	1.684537	8.999611	1.059224	8.589690	8.548286	9.383646	8.631294	8.548286	9.383646	8.631294	314.427518	769.343106	387.978202	7124	16664	11752
gene2610	NC_008261.1	2929065	2929973	-	protein_coding	CPF_RS12970	CPF_2657	.	WP_003454254.	phosphate butyr	1.617686	8.485811	1.552421	8.516701	1.042041	8.175588	8.145936	8.839868	8.205348	8.145936	8.839868	8.205348	244.462628	553.260993	292.024054	4701	10171	7508
gene2611	NC_008261.1	2930156	2931757	-	protein_coding	CPF_RS12975	CPF_2658	.	WP_003457870.	hypothetical pro	-1.212107	6.160652	-1.349169	6.235751	1.113078	6.377782	6.300973	6.023456	6.455527	6.300973	6.023456	6.455527	62.879206	93.521270	78.352139	2131	3030	3550
gene2612	NC_008261.1	2931833	2933158	-	protein_coding	CPF_RS12980	CPF_2659	.	WP_003467458.	hypothetical pro	-1.218826	7.828461	-1.182687	7.807112	-1.030557	7.950767	7.972509	7.687017	7.929084	7.972509	7.687017	7.929084	217.313918	256.029823	237.078462	6096	6866	8891
gene2613	NC_008261.1	2933125	2933982	-	protein_coding	CPF_RS12985	CPF_2660	.	WP_003454389.	TIGR00159 fami	-1.188629	6.540048	-1.189635	6.540646	1.000846	6.666495	6.665885	6.416586	6.667105	6.665885	6.416586	6.667105	80.270944	118.485745	90.784608	1457	2056	2203
gene2615	NC_008261.1	2935176	2935778	+	protein_coding	CPF_RS12995	CPF_2662	.	WP_003457891.	cell wall hydrolas	-1.525038	3.917915	-1.525038	3.917915	1.000000	4.234146	4.234146	3.625301	4.234146	4.234146	3.625301	4.234146	13.404941	17.301965	15.421388	171	211	263
gene2616	NC_008261.1	2935810	2936670	-	protein_coding	CPF_RS13000	CPF_2663	.	WP_003457940.	NAD(P)/FAD-de	-2.400134	10.031944	-1.961225	9.894201	-1.223793	10.536675	10.683362	9.420247	10.392002	10.683362	9.420247	10.392002	1374.455085	781.428981	1408.274587	25035	13607	34293
gene2617	NC_008261.1	2936697	2937011	-	protein_coding	CPF_RS13005	CPF_2664	.	WP_003457895.	thioredoxin	-2.555091	9.031731	-2.004563	8.867825	-1.274637	9.557087	9.733733	8.380358	9.383646	9.733733	8.380358	9.383646	751.218716	384.108304	665.624278	5006	2447	5930
gene2618	NC_008261.1	2937128	2938297	-	protein_coding	CPF_RS13010	CPF_2665	.	WP_003457933.	beta-aspartyl-pe	1.090465	4.481441	1.259407	4.374813	-1.154927	4.314252	4.419404	4.544348	4.211603	4.419404	4.544348	4.211603	15.797087	32.499057	15.110163	391	769	500
gene2619	NC_008261.1	2938462	2940081	-	protein_coding	CPF_RS13015	CPF_2666	.	WP_003457916.	phosphoenolpyr	6.231289	10.052886	9.431382	9.706122	-1.513552	8.515166	8.819382	11.458912	8.221443	8.819382	11.458912	8.221443	384.317219	3348.219919	295.543041	13171	109698	13541
gene2620	NC_008261.1	2940556	2942562	+	protein_coding	CPF_RS13020	CPF_2667	.	WP_003457867.	PAS domain S-l	-1.053244	4.071513	1.334880	3.820233	-1.405954	3.855505	4.109105	4.034265	3.617556	4.109105	4.034265	3.617556	12.388664	23.651252	9.689477	526	960	550
gene2621	NC_008261.1	2942733	2943326	-	protein_coding	CPF_RS13025	CPF_2668	.	WP_003457936.	30S ribosomal p	6.013967	9.139903	12.354839	8.520880	-2.054358	7.399365	7.936912	10.525229	6.898225	7.936912	10.525229	6.898225	208.179232	1654.606299	108.573391	2616	19877	1824
gene2622	NC_008261.1	2943727	2944761	-	protein_coding	CPF_RS13030	CPF_2670	.	WP_003454288.	ribonucleotide-c	1.680472	3.633982	2.026063	3.481252	-1.205651	3.140986	3.278788	4.027654	3.008975	3.278788	4.027654	3.008975	6.668046	23.504717	6.251666	146	492	183
gene2623	NC_008261.1	2944857	2947088	-	protein_coding	CPF_RS13035	CPF_2671	.	WP_003482361.	ribonucleoside-	-1.111110	5.062992	1.578728	4.646520	-1.754140	4.716793	5.139563	4.987561	4.328799	5.139563	4.987561	4.328799	26.578817	44.483587	16.490794	1255	2008	1041
gene2624	NC_008261.1	2947372	2947728	-	protein_coding	CPF_RS13040	CPF_2672	.	WP_004460474.	ArsC family tran	-1.288939	10.115353	1.157566	9.827804	-1.492032	10.007301	10.300102	9.933918	9.722824	10.300102	9.933918	9.722824	1066.025807	1061.078529	858.094744	8051	7661	8664
gene2625	NC_008261.1	2947900	2949726	-	protein_coding	CPF_RS13045	CPF_2673	.	WP_003457943.	sensor histidine	2.798258	4.537300	5.061093	4.002739	-1.808658	3.401132	3.855348	5.339878	3.000428	3.855348	5.339878	3.000428	10.245726	57.592159	6.212287	396	2128	321
gene2626	NC_008261.1	2949704	2950402	-	protein_coding	CPF_RS13050	CPF_2674	.	WP_003454428.	DNA-binding res	2.633574	4.592637	3.500109	4.347329	-1.329034	3.736123	3.946942	5.343964	3.536564	3.946942	5.343964	3.536564	11.022917	58.005204	9.256758	163	820	183
gene2627	NC_008261.1	2950656	2951513	+	protein_coding	CPF_RS13055	CPF_2675	.	WP_003454377.	YitT family prote	1.692069	4.357720	4.146279	3.583739	-2.450419	3.285286	3.994810	4.753599	2.701782	3.994810	4.753599	2.701782	11.459407	38.323453	5.027563	208	665	122
gene2628	NC_008261.1	2951774	2952445	-	protein_coding	CPF_RS13060	CPF_2676	.	WP_011591131.	N-acetylmuram	1.146779	0.992168	1.312442	0.878105	-1.144460	0.795012	0.898281	1.095868	0.703615	0.898281	1.095868	0.703615	0.633081	1.618765	0.526157	9	22	10
gene2629	NC_008261.1	2952636	2954249	-	protein_coding	CPF_RS13065	CPF_2677	.	WP_003454505.	Na/Pi cotranspo	2.343199	5.152826	2.126367	5.231121	1.101973	4.644584	4.575067	5.803547	4.715156	4.575067	5.803547	4.715156	18.011824	81.306950	21.885021	615	2654	999
gene2630	NC_008261.1	2954640	2955032	-	protein_coding	CPF_RS13070	CPF_2678	.	WP_003454434.	30S ribosomal p	2.603322	10.709132	2.117062	10.867034	1.229687	10.189225	10.041172	11.421526	10.339462	10.041172	11.421526	10.339462	903.784208	3303.564833	1372.655676	7514	26257	15257
gene2631	NC_008261.1	2955059	2955493	-	protein_coding	CPF_RS13075	CPF_2679	.	WP_003454462.	50S ribosomal p	3.233424	10.326608	2.427894	10.548538	1.331782	9.719197	9.514716	11.207779	9.928073	9.514716	11.207779	9.928073	655.586764	2858.654996	1028.139276	6033	25149	12649
gene2632	NC_008261.1	2955640	2956374	-	protein_coding	CPF_RS13080	CPF_2680	.	WP_003460461.	tRNA pseudouri	1.660116	3.493124	1.610380	3.517397	1.030885	3.168432	3.146566	3.877850	3.190449	3.146566	3.877850	3.190449	5.981109	20.787460	7.167768	93	309	149
gene2633	NC_008261.1	2956532	2957338	-	protein_coding	CPF_RS13085	CPF_2681	.	WP_003454368.	energy-coupling	2.194088	5.897598	2.056387	5.948832	1.066953	5.404515	5.357968	6.491577	5.451465	5.357968	6.491577	5.451465	31.161920	124.319368	37.197964	532	2029	849
gene2634	NC_008261.1	2957354	2958211	-	protein_coding	CPF_RS13090	CPF_2682	.	WP_003454522.	energy-coupling	1.784916	6.659537	1.664315	6.713052	1.072463	6.304972	6.254710	7.090566	6.355637	6.254710	7.090566	6.355637	60.767915	176.057369	71.704593	1103	3055	1740
gene2635	NC_008261.1	2958196	2959041	-	protein_coding	CPF_RS13095	CPF_2683	.	WP_003454376.	energy-coupling	1.793967	6.462913	1.838193	6.444135	-1.024653	6.037478	6.055071	6.898225	6.019936	6.055071	6.898225	6.019936	51.069539	158.332082	55.711493	914	2709	1333
gene2636	NC_008261.1	2959464	2959805	-	protein_coding	CPF_RS13100	CPF_2684	.	WP_003454231.	50S ribosomal p	1.531420	11.479045	1.497771	11.495494	1.022466	11.191741	11.175726	11.790596	11.207779	11.175726	11.790596	11.207779	1910.290734	4107.334956	2406.500270	13821	28409	23277
gene2637	NC_008261.1	2959822	2960769	-	protein_coding	CPF_RS13105	CPF_2685	.	WP_003454404.	DNA-directed R	1.591439	11.625195	1.487697	11.675135	1.069733	11.343381	11.294859	11.965191	11.392110	11.294859	11.965191	11.392110	2034.606884	4762.871040	2596.820226	40804	91316	69625
gene2638	NC_008261.1	2960854	2961474	-	protein_coding	CPF_RS13110	CPF_2686	.	WP_003470276.	30S ribosomal p	1.474919	11.643455	1.373285	11.696093	1.074007	11.417897	11.366511	11.927146	11.469515	11.366511	11.927146	11.469515	2218.723937	4538.990240	2744.412966	29148	57006	48201
gene2639	NC_008261.1	2961498	2961893	-	protein_coding	CPF_RS13115	CPF_2687	.	WP_003454436.	30S ribosomal p	1.450149	11.529761	1.430622	11.539766	1.013649	11.274552	11.264777	11.800978	11.284335	11.264777	11.800978	11.284335	1989.520221	4167.814835	2464.865987	16667	33379	27606
gene2640	NC_008261.1	2961910	2962275	-	protein_coding	CPF_RS13120	CPF_2688	.	WP_003454489.	30S ribosomal p	2.047160	10.412709	1.829683	10.497500	1.118861	9.989401	9.908715	10.942338	10.070745	9.908715	10.942338	10.070745	827.484270	2435.548289	1160.237602	6407	18028	12010
gene2641	NC_008261.1	2962512	2962625	-	protein_coding	CPF_RS13125	CPF_2689	.	WP_003373491.	50S ribosomal p	2.635387	10.166061	1.938786	10.400536	1.359297	9.709963	9.491057	10.889072	9.933918	9.491057	10.889072	9.933918	641.462960	2334.800669	1030.028019	1547	5383	3321
gene2642	NC_008261.1	2962638	2962856	-	protein_coding	CPF_RS13130	CPF_2690	.	WP_003454491.	translation initia	2.662277	9.649022	1.937201	9.892691	1.374290	9.194993	8.968509	10.381170	9.427196	8.968509	10.381170	9.427196	423.919580	1482.022299	698.921637	1964	6564	4329
gene2643	NC_008261.1	2962864	2963142	-	protein_coding	CPF_RS13135	CPF_2691	.	WP_003460478.	hypothetical pro	2.349997	10.281962	1.901974	10.442706	1.235557	9.835485	9.684088	10.916747	9.989249	9.684088	10.916747	9.989249	733.787127	2389.176234	1083.671664	4331	13481	8551
gene2644	NC_008261.1	2963231	2963980	-	protein_coding	CPF_RS13140	CPF_2692	.	WP_003454388.	methionine amir	2.158404	10.916487	1.771966	11.065199	1.218084	10.516947	10.375602	11.485567	10.660216	10.375602	11.485567	10.660216	1137.380551	3464.443421	1665.728684	18046	52549	35333
gene2645	NC_008261.1	2963980	2964630	-	protein_coding	CPF_RS13145	CPF_2693	.	WP_003454337.	adenylate kinase	2.465500	10.882121	1.899171	11.080172	1.298198	10.437190	10.250632	11.552513	10.627143	10.250632	11.552513	10.627143	1038.852039	3613.880018	1619.505906	14307	47580	29818
gene2646	NC_008261.1	2964657	2965934	-	protein_coding	CPF_RS13150	CPF_2694	.	WP_003460476.	preprotein trans	2.402024	10.963205	2.008338	11.099145	1.196025	10.477617	10.349288	11.613539	10.607536	10.349288	11.613539	10.607536	1104.817327	3715.561424	1602.471122	29870	96034	57921
gene2647	NC_008261.1	2965937	2966377	-	protein_coding	CPF_RS13155	CPF_2695	.	WP_003454379.	50S ribosomal p	2.157903	11.120603	1.813258	11.251771	1.190070	10.704406	10.579619	11.689250	10.830665	10.579619	11.689250	10.830665	1318.845255	3792.309873	1795.229246	12304	33823	22391
gene2648	NC_008261.1	2966411	2966590	-	protein_coding	CPF_RS13160	CPF_2696	.	WP_034824315.	50S ribosomal p	2.427625	10.674838	1.954384	10.839637	1.242143	10.209438	10.054220	11.333765	10.367052	10.054220	11.333765	10.367052	910.211171	3156.021877	1389.167970	3466	11489	7072
gene2649	NC_008261.1	2966596	2967093	-	protein_coding	CPF_RS13165	CPF_2697	.	WP_003454272.	30S ribosomal p	2.173122	11.536016	1.945842	11.619364	1.116803	11.069111	10.989710	12.109479	11.149085	10.989710	12.109479	11.149085	1676.093639	5286.243229	2313.449739	17658	53241	32584
gene2650	NC_008261.1	2967114	2967473	-	protein_coding	CPF_RS13170	CPF_2698	.	WP_003454424.	50S ribosomal p	2.482699	10.889399	1.989893	11.057613	1.247655	10.411569	10.253183	11.565093	10.572402	10.253183	11.565093	10.572402	1042.961242	3622.873576	1574.599893	7943	26377	16032
gene2651	NC_008261.1	2967504	2968043	-	protein_coding	CPF_RS13175	CPF_2699	.	WP_003454268.	50S ribosomal p	2.397322	11.171203	1.876239	11.356715	1.277728	10.733616	10.558281	11.819705	10.911861	10.558281	11.819705	10.911861	1309.030376	4271.210040	1893.474705	14954	46646	28918
gene2652	NC_008261.1	2968140	2968538	-	protein_coding	CPF_RS13180	CPF_2700	.	WP_003454446.	30S ribosomal p	2.560803	11.570294	1.963733	11.771597	1.304049	11.101709	10.911861	12.268458	11.294859	10.911861	12.268458	11.294859	1592.017543	5681.941067	2485.146966	13438	45850	28044
gene2653	NC_008261.1	2968572	2968757	-	protein_coding	CPF_RS13185	CPF_2701	.	WP_003470274.	30S ribosomal p	2.713524	10.056996	2.068256	10.265243	1.311987	9.556527	9.362658	10.802826	9.754411	9.362658	10.802826	9.754411	571.306902	2181.999688	873.679388	2248	8208	4596
gene2654	NC_008261.1	2968774	2969313	-	protein_coding	CPF_RS13190	CPF_2702	.	WP_003454431.	50S ribosomal p	2.320008	10.777373	1.950625	10.908911	1.189366	10.311729	10.187392	11.401521	10.437584	10.187392	11.401521	10.437584	1011.229030	3286.687244	1444.692783	11552	35894	22064
gene2655	NC_008261.1	2969336	2969650	-	protein_coding	CPF_RS13195	CPF_2703	.	WP_003454257.	50S ribosomal p	2.402349	10.656747	1.900709	10.834539	1.263923	10.210818	10.043262	11.307708	10.381170	10.043262	11.307708	10.381170	906.534611	3134.242135	1402.076366	6041	19967	12491
gene2656	NC_008261.1	2969674	2970042	-	protein_coding	CPF_RS13200	CPF_2704	.	WP_003454425.	50S ribosomal p	2.049187	10.824294	1.768112	10.935355	1.158969	10.425011	10.319133	11.354184	10.531975	10.319133	11.354184	10.531975	1084.905410	3206.881473	1522.971782	8469	23932	15894
gene2657	NC_008261.1	2970073	2970327	-	protein_coding	CPF_RS13205	CPF_2705	.	WP_003454312.	30S ribosomal p	2.601387	10.215259	1.923128	10.445779	1.352686	9.764353	9.548871	10.928152	9.984698	9.548871	10.928152	9.984698	672.161646	2426.727179	1083.334710	3626	12515	7813
gene2658	NC_008261.1	2970349	2970558	-	protein_coding	CPF_RS13210	CPF_2706	.	WP_003454396.	50S ribosomal p	2.310383	8.557725	1.901120	8.707329	1.215264	8.114375	7.974958	9.183078	8.256228	7.974958	9.183078	8.256228	217.667349	675.525168	303.403444	967	2869	1802
gene2659	NC_008261.1	2970558	2970992	-	protein_coding	CPF_RS13215	CPF_2707	.	WP_003454381.	50S ribosomal p	2.389013	10.528794	1.916007	10.696395	1.246871	10.077210	9.919311	11.175726	10.237623	9.919311	11.175726	10.237623	841.515647	2767.265333	1287.836089	7744	24345	15844
gene2660	NC_008261.1	2971023	2971691	-	protein_coding	CPF_RS13220	CPF_2708	.	WP_003454406.	30S ribosomal p	2.433089	11.385969	1.887181	11.578239	1.289272	10.944370	10.762626	12.045415	11.129183	10.762626	12.045415	11.129183	1464.806444	5171.272408	2294.503362	20731	69967	43414
gene2661	NC_008261.1	2971712	2972047	-	protein_coding	CPF_RS13225	CPF_2709	.	WP_003454177.	50S ribosomal p	2.832589	10.863127	2.085647	11.097206	1.358125	10.356458	10.138003	11.640115	10.579619	10.138003	11.640115	10.579619	977.195840	3740.964904	1591.626349	6946	25421	15125
gene2662	NC_008261.1	2972075	2972356	-	protein_coding	CPF_RS13230	CPF_2710	.	WP_003454422.	50S ribosomal p	2.906100	10.980232	2.111138	11.224763	1.376556	10.465616	10.237623	11.776707	10.698687	10.237623	11.776707	10.698687	1035.080106	4063.506091	1686.014521	6175	23175	13447
gene2663	NC_008261.1	2972427	2973260	-	protein_coding	CPF_RS13235	CPF_2711	.	WP_003460473.	50S ribosomal p	2.781397	12.032387	2.047776	12.264975	1.358253	11.535849	11.317087	12.792897	11.758839	11.317087	12.792897	11.758839	2095.865822	8264.105410	3384.087383	36978	139390	79822
gene2664	NC_008261.1	2973315	2973608	-	protein_coding	CPF_RS13240	CPF_2712	.	WP_003454311.	50S ribosomal p	3.284853	10.376296	2.244449	10.670511	1.463545	9.824681	9.553787	11.269616	10.103255	9.553787	11.269616	10.103255	676.251196	3071.363994	1177.991412	4206	18262	9795
gene2665	NC_008261.1	2973608	2974222	-	protein_coding	CPF_RS13245	CPF_2713	.	WP_003454270.	50S ribosomal p	3.167619	11.492731	2.390691	11.708904	1.324981	10.892180	10.691086	12.354485	11.097057	10.691086	12.354485	11.097057	1384.359287	5872.162848	2241.453412	18011	73037	38987
gene2666	NC_008261.1	2974247	2974876	-	protein_coding	CPF_RS13250	CPF_2714	.	WP_003476250.	50S ribosomal p	3.460911	12.170377	2.426967	12.442856	1.426023	11.560872	11.307708	13.098859	11.819705	11.307708	13.098859	11.819705	2082.133377	9726.714775	3499.410199	27750	123930	62352
gene2667	NC_008261.1	2974959	2975267	-	protein_coding	CPF_RS13255	CPF_2715	.	WP_003479233.	30S ribosomal p	4.337931	12.227984	2.939374	12.530347	1.475801	11.492529	11.215209	13.332216	11.776707	11.215209	13.332216	11.776707	1939.907987	11518.174227	3481.540053	12681	71980	30426
gene2668	NC_008261.1	2975963	2977156	-	protein_coding	CPF_RS13260	CPF_2716	.	WP_003452162.	elongation facto	1.287002	9.128558	1.250318	9.149813	1.029340	8.969200	8.948365	9.312379	8.990085	8.948365	9.312379	8.990085	417.591743	733.571743	502.204879	10548	17714	16959
gene2669	NC_008261.1	2977229	2979295	-	protein_coding	CPF_RS13265	CPF_2717	.	WP_003460612.	elongation facto	1.613474	11.953644	1.427127	12.044420	1.130575	11.701732	11.613539	12.303709	11.790596	11.613539	12.303709	11.790596	2749.255674	5833.707105	3490.635878	120218	243868	204061
gene2670	NC_008261.1	2979376	2979846	-	protein_coding	CPF_RS13270	CPF_2718	.	WP_003452182.	30S ribosomal p	1.994786	12.706964	1.501357	12.918382	1.328655	12.421899	12.218607	13.214840	12.628574	12.218607	13.214840	12.628574	4326.163467	10372.405934	6060.218744	43106	98803	80728
gene2671	NC_008261.1	2980006	2980386	-	protein_coding	CPF_RS13275	CPF_2719	.	WP_003452165.	30S ribosomal p	1.703117	12.423568	1.391837	12.572836	1.223647	12.190139	12.045415	12.813593	12.336603	12.045415	12.813593	12.336603	3600.588629	8464.333339	4912.411639	29021	65221	52934
gene2672	NC_008261.1	2980444	2980683	-	protein_coding	CPF_RS13280	CPF_2720	.	WP_003452175.	50S ribosomal p	1.483496	9.983718	1.411205	10.020728	1.051227	9.739240	9.703270	10.272271	9.775344	9.703270	10.272271	9.775344	737.412859	1366.973093	900.295725	3744	6635	6111
gene2673	NC_008261.1	2980796	2984332	-	protein_coding	CPF_RS13285	CPF_2721	.	WP_003460614.	DNA-directed R	1.385424	9.981231	1.307674	10.023796	1.059457	9.790411	9.748838	10.219165	9.832162	9.748838	10.219165	9.832162	764.793941	1292.457375	955.519818	57226	92453	95585
gene2674	NC_008261.1	2984412	2988116	-	protein_coding	CPF_RS13290	CPF_2722	.	WP_003460611.	DNA-directed R	1.944536	10.062978	1.663215	10.180519	1.169143	9.706764	9.594693	10.554119	9.820144	9.594693	10.554119	9.820144	697.249260	1685.204332	933.283210	54650	126273	97795

gene2675	NC_008261.1	2988421	2988786	-	protein_coding	CPF_RS13295	CPF_2723	.	WP_003452170.	50S ribosomal p	1.744145	11.122562	1.488674	11.240380	1.171610	10.842182	10.728538	11.531058	10.957030	10.728538	11.531058	10.957030	1433.212571	3523.898467	1950.087946	11097	26084	20186
gene2676	NC_008261.1	2988841	2989341	-	protein_coding	CPF_RS13300	CPF_2724	.	WP_003452177.	50S ribosomal p	2.125782	12.072326	1.663656	12.255896	1.277777	11.716064	11.540580	12.628574	11.894216	11.540580	12.628574	11.894216	2577.114339	6674.999025	3643.051315	27314	67633	51620
gene2677	NC_008261.1	2989558	2990247	-	protein_coding	CPF_RS13305	CPF_2725	.	WP_003452172.	50S ribosomal p	1.867348	12.040639	1.680612	12.119281	1.111112	11.674323	11.598568	12.499559	11.750573	11.598568	12.499559	11.750573	2708.231618	6331.368262	3346.793421	39532	88352	65312
gene2678	NC_008261.1	2990305	2990730	-	protein_coding	CPF_RS13310	CPF_2726	.	WP_003452181.	50S ribosomal p	1.943632	12.022337	1.767878	12.093276	1.099416	11.620680	11.552513	12.511268	11.689250	11.552513	12.511268	11.689250	2587.536183	6440.734638	3185.188365	23319	55490	38376
gene2679	NC_008261.1	2990805	2991326	-	protein_coding	CPF_RS13315	CPF_2727	.	WP_003452167.	transcription ter	1.749556	10.921272	1.675591	10.953556	1.044142	10.556343	10.525229	11.332218	10.587548	10.525229	11.332218	10.587548	1276.382042	3152.318170	1601.734620	14095	33279	23647
gene2680	NC_008261.1	2991364	2991588	-	protein_coding	CPF_RS13320	CPF_2728	.	WP_003479237.	preprotein trans	1.950212	9.782337	1.795090	9.844941	1.086415	9.371976	9.312379	10.276011	9.431954	9.312379	10.276011	9.431954	552.534421	1368.442735	699.141210	2630	6227	4449
gene2681	NC_008261.1	2991642	2991791	-	protein_coding	CPF_RS13325	CPF_2729	.	WP_003482374.	50S ribosomal p	1.774690	11.905635	1.656386	11.957048	1.071423	11.548697	11.499040	12.326607	11.598568	11.499040	12.326607	11.598568	2478.211420	5842.527469	2965.339320	7864	17724	12580
gene2682	NC_008261.1	2991974	2993167	-	protein_coding	CPF_RS13330	CPF_2730	.	WP_003452162.	elongation facto	1.105424	10.575834	1.273041	10.472794	-1.151631	10.401443	10.503781	10.648381	10.300102	10.503781	10.648381	10.300102	1254.556763	1827.427678	1338.916882	31689	44128	45214
gene2683	NC_008261.1	2993234	2993309	-	tRNA	CPF_RS13335	CPF_2731	.	.	tRNA-Thr	1.728972	13.095837	1.404042	13.249694	1.231425	12.856121	12.706834	13.496749	13.007163	12.706834	13.496749	13.007163	6604.124271	11679.641913	7231.596895	10618	17952	15544
gene2684	NC_008261.1	2993392	2994039	-	protein_coding	CPF_RS13340	CPF_2732	.	WP_003459372.	RNA polymerase	1.290550	7.704344	1.248015	7.729064	1.034082	7.546684	7.522547	7.890534	7.570898	7.522547	7.890534	7.570898	149.104927	286.297862	182.299923	2044	3752	3341
gene2685	NC_008261.1	2994120	2994629	-	protein_coding	CPF_RS13345	CPF_2733	.	WP_003459316.	NYN domain-co	1.351875	7.785342	1.297420	7.815780	1.041971	7.600498	7.570898	8.005860	7.630214	7.570898	8.005860	7.630214	154.693572	301.619986	189.961417	1669	3111	2740
gene2686	NC_008261.1	2994645	2995514	-	protein_coding	CPF_RS13350	CPF_2734	.	WP_003459298.	23S rRNA (guan	1.011247	7.098628	1.207480	6.969374	-1.194051	6.961458	7.090566	7.106700	6.834701	7.090566	7.106700	6.834701	112.415798	178.005238	104.000649	2069	3132	2559
gene2687	NC_008261.1	2995537	2996274	-	protein_coding	CPF_RS13355	CPF_2735	.	WP_003452247.	thymidylate synt	-1.058742	7.376578	1.210776	7.196229	-1.281899	7.236510	7.417868	7.335518	7.059585	7.417868	7.335518	7.059585	137.903020	209.039575	121.883736	2153	3120	2544
gene2688	NC_008261.1	2996290	2996700	-	protein_coding	CPF_RS13360	CPF_2736	.	WP_003459297.	hypothetical pro	1.313671	7.041561	1.653044	6.868988	-1.258339	6.679691	6.847508	7.241112	6.515987	6.847508	7.241112	6.515987	92.124852	196.339922	81.641205	801	1632	949
gene2689	NC_008261.1	2996927	2998324	-	protein_coding	CPF_RS13365	CPF_2737	.	WP_025648643.	cysteine--tRNA	1.328445	7.755612	1.414487	7.708990	-1.064769	7.508042	7.553449	7.963187	7.462908	7.553449	7.963187	7.462908	152.393518	295.048421	170.086612	4507	8342	6725
gene2690	NC_008261.1	2998432	3000144	-	protein_coding	CPF_RS13370	CPF_2738	.	WP_003459370.	proline--tRNA l	2.051171	7.168227	2.101829	7.149285	-1.024697	6.651089	6.668712	7.705159	6.633514	6.668712	7.705159	6.633514	80.494309	259.266239	88.549261	2917	8982	4290
gene2691	NC_008261.1	3000499	3001176	-	protein_coding	CPF_RS13375	CPF_2739	.	WP_003459324.	2-C-methyl-D-	1.244112	8.022437	1.080961	8.125191	1.150932	7.967182	7.866426	8.181542	8.069228	7.866426	8.181542	8.069228	194.448648	341.891410	260.750605	2789	4688	5000
gene2692	NC_008261.1	3001190	3002296	-	protein_coding	CPF_RS13380	CPF_2740	.	WP_003459365.	PIN/TRAM dom	1.270121	8.341709	1.206406	8.379524	1.052814	8.208050	8.171009	8.515975	8.245259	8.171009	8.515975	8.245259	247.836856	434.873782	301.930545	5804	9736	9453
gene2693	NC_008261.1	3002608	3003009	+	protein_coding	CPF_RS13385	CPF_2741	.	WP_003459340.	hypothetical pro	-3.875162	7.010055	-3.220605	6.892927	-1.203241	7.920369	8.054956	6.100700	7.788031	8.054956	6.100700	7.788031	231.529200	98.522799	216.632381	1969	801	2463
gene2695	NC_008261.1	3004125	3005486	-	protein_coding	CPF_RS13395	CPF_2743	.	WP_003479244.	DNA repair prot	2.708163	6.979476	3.265767	6.828162	-1.205897	6.161187	6.297720	7.735035	6.027613	6.297720	7.735035	6.027613	62.783796	264.038198	56.229777	1809	7273	2166
gene2696	NC_008261.1	3005737	3006465	-	protein_coding	CPF_RS13400	CPF_2744	.	WP_011591132.	glucosamine-6-	1.448582	10.944624	2.180880	10.637895	-1.505527	10.381239	10.680568	11.215209	10.090299	10.680568	11.215209	10.090299	1371.285493	2864.809947	1171.365973	21148	42237	24151
gene2697	NC_008261.1	3006462	3007184	-	protein_coding	CPF_RS13405	CPF_2745	.	WP_003469605.	GntR family tran	1.168980	8.487065	2.805365	7.821026	-2.399841	7.717928	8.375187	8.600437	7.112248	8.375187	8.600437	7.112248	282.116580	467.786932	126.270807	4315	6840	2582
gene2698	NC_008261.1	3007642	3007869	+	protein_coding	CPF_RS13410	CPF_2747	.	WP_003452255.	NrdH-redoxin	-1.104371	12.156903	-1.163288	12.194117	1.053349	12.266160	12.228726	12.085501	12.303709	12.228726	12.085501	12.303709	4342.832646	5277.048549	4817.652881	20947	24333	31066
gene2699	NC_008261.1	3007991	3009439	-	protein_coding	CPF_RS13415	CPF_2748	.	WP_003459369.	NADP-depende	-4.893884	10.612574	-4.532554	10.562780	-1.079719	11.764247	11.819705	9.528725	11.709049	11.819705	9.528725	11.709049	3157.535285	824.405440	3263.091375	96790	24159	133725
gene2700	NC_008261.1	3009456	3009689	-	protein_coding	CPF_RS13420	CPF_2749	.	WP_003452243.	NrdH-redoxin	-5.303221	9.084252	-4.237105	8.941264	-1.251614	10.203872	10.367052	7.960183	10.043262	10.367052	7.960183	10.043262	1132.259225	294.562324	1139.910717	5605	1394	7544
gene2701	NC_008261.1	3009692	3010630	-	protein_coding	CPF_RS13425	CPF_2750	.	WP_003452248.	thioredoxin-disu	-3.242635	9.545967	-2.543145	9.384210	-1.275049	10.255419	10.432194	8.735027	10.081641	10.432194	8.735027	10.081641	1180.794900	506.201736	1165.677166	23456	9613	30957
gene2702	NC_008261.1	3010907	3013351	-	protein_coding	CPF_RS13430	CPF_2751	.	WP_003459331.	ATP-dependent	-1.735734	4.480041	-1.629864	4.438301	-1.064956	4.849828	4.895438	4.099892	4.804643	4.895438	4.099892	4.804643	22.620026	24.692615	23.297091	1170	1221	1611
gene2703	NC_008261.1	3013376	3014389	-	protein_coding	CPF_RS13435	CPF_2752	.	WP_003459295.	protein arginine	-1.048234	2.176382	1.130562	2.052238	-1.185094	2.084530	2.210627	2.142667	1.965626	2.210627	2.142667	1.965626	2.657192	4.973848	2.650090	57	102	76
gene2704	NC_008261.1	3014373	3014900	-	protein_coding	CPF_RS13440	CPF_2753	.	WP_003459366.	hypothetical pro	1.210885	2.305500	1.175281	2.328243	1.030293	2.193019	2.171597	2.447659	2.214653	2.171597	2.447659	2.214653	2.596272	6.368032	3.348275	29	68	50
gene2705	NC_008261.1	3014920	3015375	-	protein_coding	CPF_RS13445	CPF_2754	.	WP_003452250.	CtsR family tran	-1.466259	4.633029	-1.373994	4.588645	-1.067150	4.870210	4.917317	4.365177	4.823553	4.917317	4.365177	4.823553	22.909391	29.602479	23.494316	221	273	303
gene2706	NC_008261.1	3015592	3016245	+	protein_coding	CPF_RS13450	CPF_2755	.	WP_003479254.	hydrolase	-1.203423	0.178989	-1.063188	0.126460	-1.131900	0.252163	0.356906	0.089763	0.178160	0.356906	0.089763	0.178160	0.216835	0.075605	0.108128	3	1	2
gene2707	NC_008261.1	3016296	3017978	-	protein_coding	CPF_RS13455	CPF_2756	.	WP_011591134.	ligase	1.226495	0.598487	-1.017112	0.775754	1.247483	0.608004	0.469070	0.763611	0.788090	0.469070	0.763611	0.788090	0.280868	1.057666	0.609255	10	36	29
gene2708	NC_008261.1	3018096	3018266	-	protein_coding	CPF_RS13460	CPF_2757	.	WP_003452217.	ferredoxin	1.177772	13.764202	1.080020	13.827097	1.090510	13.709036	13.646677	13.882738	13.771680	13.646677	13.882738	13.771680	13201.337714	13780.369742	12964.313310	47756	47657	62699
gene2709	NC_008261.1	3018435	3019340	-	protein_coding	CPF_RS13465	CPF_2758	.	WP_003452261.	stage 0 sporulat	-1.262285	7.790352	-1.208440	7.759516	-1.044557	7.928675	7.960183	7.624145	7.897291	7.960183	7.624145	7.897291	214.854400	249.685419	232.596447	4118	4575	5960
gene2710	NC_008261.1	3019343	3020266	-	protein_coding	CPF_RS13470	CPF_2759	.	WP_011591135.	DNA polymerase	-1.394931	5.453292	-1.259545	5.382357	-1.107488	5.624544	5.698672	5.218478	5.551381	5.698672	5.218478	5.551381	39.596345	53.084775	39.605308	774	992	1035
gene2711	NC_008261.1	3020288	3020617	-	protein_coding	CPF_RS13475	CPF_2760	.	WP_003459310.	hypothetical pro	-1.695087	7.720714	-1.243208	7.504805	-1.363478	7.883956	8.110773	7.349414	7.663482	8.110773	7.349414	7.663482	240.361068	211.568515	195.003525	1678	1412	1820
gene2712	NC_008261.1	3020946	3021536	-	protein_coding	CPF_RS13480	CPF_2762	.	WP_003459338.	guanylate kinase	1.973609	2.401393	2.113041	2.340301	-1.070648	1.910664	1.960541	2.941377	1.862056	1.960541	2.941377	1.862056	2.159546	9.788782	2.333255	27	117	39
gene2713	NC_008261.1	3021783	3023213	-	protein_coding	CPF_RS13485	CPF_2763	.	WP_011591136.	aminotransferas	1.763333	2.648442	2.440555	2.359193	-1.384058	2.022713	2.270708	3.089012	1.801803	2.270708	3.089012	1.801803	2.840828	11.057084	2.174343	86	320	88
gene2714	NC_008261.1	3023231	3023407	-	protein_coding	CPF_RS13490	CPF_2764	.	WP_003452260.	carnitine-CoA li	2.278697	1.256349	1.675179	1.568129	1.360271	0.993081	0.795634	1.983843	1.239528	0.795634	1.983843	1.239528	0.534125	4.190330	1.198569	2	15	6
gene2715	NC_008261.1	3023563	3024732	+	protein_coding	CPF_RS13495	CPF_2765	.	WP_011591137.	amidohydrolase	-1.178731	0.388438	-1.049264	0.320344	-1.123388	0.432771	0.524762	0.287528	0.356906	0.524762	0.287528	0.356906	0.323214	0.295830	0.241763	8	7	8
gene2716	NC_008261.1	3024761	3025258	-	protein_coding	CPF_RS13500	CPF_2766	.	WP_003459375.	hypothetical pro	2.306529	4.305380	4.703748	3.667017	-2.039319	3.189317	3.744521	4.950245	2.716434	3.744521	4.950245	2.716434	9.491979	43.786429	5.111968	100	441	72
gene2717	NC_008261.1	3025533	3026477	+	protein_coding	CPF_RS13505	CPF_2767	.	WP_003459363.	magnesium tran	1.281036	4.257095	1.574806	4.098841	-1.229322	3.930434	4.082186	4.439498	3.784324	4.082186	4.439498	3.784324	12.105136	30.609366	10.962783	242	585	293
gene2718	NC_008261.1	3026569	3027450	+	protein_coding	CPF_RS13510	CPF_2768	.	WP_011591138.	LD-carboxypept	2.190142	0.922241	1.682862	1.215191	1.301438	0.680312	0.516307	1.647331	0.896414	0.516307	1.647331	0.896414	0.321565	3.139422	0.761676	6	56	19
gene2719	NC_008261.1	3027823	3027897	-	tRNA	CPF_RS13515	CPF_2769	.	.	tRNA-Cys	6.644913	2.854977	3.449746	3.526506	1.926204	2.221988	1.798869	4.531120	2.744630	1.798869	4.531120	2.744630	1.890802	32.304654	5.185808	3	49	11
gene2724	NC_008261.1	3029408	3032318	-	rRNA	CPF_RS13540	CPF_3020	.	.	23S ribosomal R	-1.742073	6.001601	-2.088003	6.122614	1.198573	6.544701	6.415346	5.614540	6.676664	6.415346	5.614540	6.676664	68.006524	70.831123	91.813629	4188	4170	7559
gene2727	NC_008261.1	3032731	3034249	-	rRNA	CPF_RS13555	CPF_3021	.	.	16S ribosomal R	-1.098307	0.094323	1.002743	0.046410	-1.101320	0.090392	0.183710	0.048429	0.044476	0.183710	0.048429	0.044476	0.093358	0.032552	0.023277	3	1	1
gene2728	NC_008261.1	3034751	3036127	-	protein_coding	CPF_RS13560	CPF_2774	.	WP_003470204.	UDP-N-acetylm	-1.211970	6.499979	-1.085025	6.421375	-1.116998	6.559837	6.640135	6.362781	6.480509	6.640135	6.362781	6.480509	78.783425	112.896084	79.676977	2295	3144	3103
gene2729	NC_008261.1	3036438	3037481	+	protein_coding	CPF_RS13565	CPF_2775	.	WP_003450326.	[FeFe] hydroger	1.067268	5.648243	1.175316	5.577658	-1.101239	5.531476	5.601477	5.695399	5.462350	5.601477	5.695399	5.462350	37.535321	74.974003	37.457574	829	1583	1106
gene2730	NC_008261.1	3037723	3038964	-	protein_coding	CPF_RS13570	CPF_2776	.	WP_011010917.	dihydropyrimidine	1.305178	5.734620	1.633798	5.564590	-1.251782	5.381285	5.545714	5.929961	5.221731	5.545714	5.929961	5.221731	35.966346	88.421371	31.970040	945	2221	1123
gene2731	NC_008261.1	3039056	3039925	-	protein_coding	CPF_RS13575	CPF_2777	.	WP_011591139.	LD-carboxypept	1.143426	3.001532	1.581727	2.749196	-1.383323	2.662068	2.906408	3.099771	2.438270	2.906408	3.099771	2.438270	4.998672	11.082702	3.982831	92	195	98
gene2732	NC_008261.1	3040098	3041216	-	protein_coding	CPF_RS13580	CPF_2778	.	WP_003470196.	hypothetical pro	1.796600	4.356057	1.684036	4.407178	1.066842	4.000278	3.953877	4.799146	4.047224	3.953877	4.799146	4.047224	11.067698	39.282757	13.081431	262	889	414
gene2733	NC_008261.1	3041404	3042807	-	protein_coding	CPF_RS13585	CPF_2779	.	WP_003450306.	glycine--tRNA l	1.761562	8.446908	1.646346	8.497963	1.069983	8.096995	8.048348	8.865204	8.145936	8.048348	8.865204	8.145936	230.189007	561.126435	276.843375	6837	15933	10993
gene2735	NC_008261.1	3043495	3045000	-	protein_coding	CPF_RS13595	CPF_2781	.	WP_011591140.	lysine--tRNA lig	2.019977	9.423598	2.595661	9.230746	-1.284995	8.747314	8.930066	9.944405	8.568303	8.930066	9.944405	8.568303	411.776794	1062.298854	371.984528	13119	32355	15844
gene2736	NC_008261.1	3045026	3045502	-	protein_coding	CPF_RS13600	CPF_2782	.	WP_003450338.	transcription elo	2.038703	8.293410	2.625218	8.097042	-1.287691	7.610908	7.795486	8.823138	7.430700	7.795486	8.823138	7.430700	183.629795	541.106068	164.039354	1853	5220	2213
gene2737	NC_008261.1	3045781	3046761	-	protein_coding	CPF_RS13605	CPF_2783	.	WP_003459360.	tRNA dihydrouri	1.868909	6.328747	1.919809	6.307899	-1.027235	5.874290	5.893705	6.795901	5.854939	5.893705	6.795901	5.854939	44.668034	148.841734	49.594606	927	2953	1376
gene2738	NC_008261.1	3046768	3047547	-	protein_coding	CPF_RS13610	CPF_2784	.	WP_003450343.	type III pantothe	2.570614	6.159920	2.355857	6.229795	1.091159	5.578974	5.516398	6.878511	5.642259	5.516398	6.878511	5.642259	35.088925	157.783133	42.384008	579	2489	935
gene2739	NC_008261.1	3047750	3049420	-	protein_coding	CPF_RS13615	CPF_2785	.	WP_003470195.	formate--tetrah	6.469027	7.218029	7.153693	7.130131	-1.105838	5.922809	5.995824	8.689372	5.850684	5.995824	8.689372	5.850684	48.910787	491.766249	49.555910	1729	16619	2342
gene2740	NC_008261.1	3049622	3051427	-	protein_coding	CPF_RS13620	CPF_2786	.	WP_011591141.	ATP-dependent	-1.744931	9.459029	-1.448713	9.329520	-1.204469	9.734011	9.869135	9.065965	9.600737	9.869135	9.065965	9.600737	815.212145	626.917056	784.586442	31146	22898	40075
gene2741	NC_008261.1	3051502	3052053	-	protein_coding	CPF_RS13625	CPF_2787	.	WP_003450331.	hypoxanthine ph	-1.032908	6.237199	1.057490	6.173433	-1.092290	6.196593	6.260598	6.213887	6.133242	6.260598	6.213887	6.133242	61.057154	104.445505	61.171525	713	1166	955
gene2742	NC_008261.1	3052056	3053465	-	protein_coding	CPF_RS13630	CPF_2788	.	WP_003479285.	tRNA(Ile)-lysidin	1.110338	3.803420	1.088051	3.818311	1.020484	3.743268	3.728670	3.879669	3.757923	3.728670	3.879669	3.757923	9.420486	20.795332	10.707640	281	593	427
gene2743	NC_008261.1	3053682	3056072	-	protein_coding	CPF_RS13635	CPF_2789	.	WP_011591142.	stage II sporulat	-1.022416	0.956298	-1.454579	1.180183	1.422687	1.200083	0.972424	0.940441	1.481042	0.972424	0.940441	1.481042	0.731490	1.323521	1.537938	37	64	104
gene2750	NC_008261.1	3056931	3057019	-	tRNA	CPF_RS13670	CPF_2796	.	.	tRNA-Leu	1.020826	0.798824	1.187242	0.678825	-1.163021	0.666308	0.784094	0.813830	0.566216	0.784094	0.813830	0.566216	0.531124	1.111144	0.397278	1	2	1
gene2751	NC_008261.1	3057379	3057783	-	protein_coding	CPF_RS13675	CPF_2797	.	WP_003450336.	RNA-binding pro	1.321624	10.127232	1.496454	10.035400	-1.132284	9.838047	9.928073	10.330386	9.748838	9.928073	10.330386	9.748838	847.126073	1432.099752	869.539529	7258	11730	9960
gene2752	NC_008261.1	3057843	3058163	-	protein_coding	CPF_RS13680	CPF_2798	.	WP_003450312.	cell division prot	1.074957	9.292088	1.465019	9.064730	-1.362863	9.014009	9.240095	9.344374	8.793454	9.240095	9.344374	8.793454	519.823347	744.923269	434.316930	3530	4836	3943
gene2754	NC_008261.1	3058643	3058942	-	protein_coding	CPF_RS13690	CPF_2800	.	WP_003479288.	sporulation prot	-1.379571	1.045896	-1.231562	0.978003	-1.120180	1.218840	1.303452	0.839232	1.139721	1.303452	0.839232	1.139721	1.102968	1.153738	1.060733	7	7	9
gene2755	NC_008261.1	3059073	3059333	-	protein_coding	CPF_RS13695	CPF_2801	.	WP_003450572.	RNA-binding S4	1.217409	5.439083	1.040180	5.554352	1.170384	5.411328	5.299027	5.582842	5.526009	5.299027	5.582842	5.526009	29.521145	68.959032	39.015484	163	364	288
gene2756	NC_008261.1	3059441	3059716	-	protein_coding	CPF_RS13700	CPF_2802	.	WP_003450698.	HU family DNA-b	3.405694	11.143364	3.369318	11.151746	1.010796	10.302140	10.294397	12.062345	10.309889	10.294397	12.062345	10.309889	1057.239311	5202.568542	1351.282192	6173	29040	10548
gene2757	NC_008261.1	3059933	3061384	-	protein_coding	CPF_RS13705	CPF_2803	.	WP_003467940.	nucleoside triph	-1.010650	5.118054	-1.084366	5.168514	1.072940	5.176236	5.125701	5.110418	5.227270	5.125701	5.110418	5.227270	26.467324	48.390236	32.046034	813	1421	1316
gene2758	NC_008261.1	3061414	3062946	-	protein_coding	CPF_RS13710	CPF_2804	.	WP_003467866.	polysaccharide b	1.433922	0.857396	1.137653	1.058820	1.260422	0.785366	0.635962	1.155929	0.969869	0.635962	1.155929	0.969869	0.400855	1.773989	0.830320	13	55	36
gene2759	NC_008261.1	3063139	3063687	-	protein_coding	CPF_RS13715	CPF_2805	.	WP_003450655.	stage V sporulat	1.605702	1.307248	1.096176	1.625157	1.464821	1.255051	1.009541	1.692746	1.560266	1.009541	1.692746	1.560266	0.774919	3.332419	1.674503	9	37	26
gene2760	NC_008261.1	3063880	3064908	-	protein_coding	CPF_RS13720	CPF_2806	.	WP_011591143.	foldase	4.951165	8.942994	3.521446	9.218308	1.406003	8.105317	7.863243	10.171011	8.354843	7.863243	10.171011	8.354843	193.903695	1235.426698	328.700236	4221	25710	9566
gene2761	NC_008261.1	3065009	3068515	-	protein_coding	CPF_RS13725	CPF_2807	.	WP_003467883.	transcription-re	-1.021478	5.299584	-1.306383	5.473672	1.278914	5.489528	5.314935	5.284278	5.669855	5.314935	5.284278	5.669855	29.976790	55.325266	43.312527	2224	3924	4296
gene2762	NC_008261.1	3068546	3069112	-	protein_coding	CPF_RS13730	CPF_2808	.	WP_003450588.	peptidyl-tRNA h	1.001147	5.810674	1.028235	5.791382	-1.027056	5.790557	5.809847	5.811501	5.771332	5.809847	5.811501	5.771332	42.351302	81.973801	46.520115	508	940	746
gene2763	NC_008261.1	3069238	3070395	-	protein_coding	CPF_RS13735	CPF_2809	.	WP_011591145.	PDZ domain-co	-2.867570	10.063776	-2.466818	9.962569	-1.162457	10.743203	10.852340	9.332512	10.635163	10.852340	9.332512	10.635163	1540.562934	740.706404	1635.526420	37740	17347	53565
gene2765	NC_008261.1	3071824	3072510	-	protein_coding	CPF_RS13745	CPF_2811	.	WP_011591146.	DNA-binding res	-2.727745	11.132540	-2.697176	11.124919	-1.011334	11.871770	11.879903	10.432194	11.863643	11.879903	10.432194	11.863643	3326.999959	1545.202348	3568.253838	48353	21469	69331
gene2766	NC_008261.1	3072782	3073741	-	protein_coding	CPF_RS13750	CPF_2812	.	WP_003456820.	ribose-phosphat	2.656649	9.253091	2.706469	9.238617	-1.018753	8.561678	8.575090	9.984698	8.548286	8.575090	9.984698	8.548286	319.072872	1096.823361	368.899526	6480	21295	10016
gene2767	NC_008261.1	3073798	3075162	-	protein_coding	CPF_RS13755	CPF_2813	.	WP_003456738.	bifunctional N-a	1.775854	8.839228	1.792592	8.232108	-1.009426	7.828609	7.835379	8.663891	7.821844	7.835379	8.663891	7.821844	189.807452	486.018778	221.808562	5481	13417	8563
gene2768	NC_008261.1	3075372	3075644	-	protein_coding	CPF_RS13760	CPF_2814	.	WP_003450704.	septation protei	-1.328867	11.569791	-1.351411	11.581707	1.016965	11.788836	11.776707	11.366511	11.800978	11.776707	11.366511	11.800978	3050.563480	3248.577476	3497.182530	17618	17936	27002
gene2769	NC_008261.1	3075755	3076558	-	protein_coding	CPF_RS13765	CPF_2815	.	WP_003456678.	pur operon repr	2.850908	6.293341	1.814602	6.650561	1.571093	5.899732	5.582842	7.094263	6.234610	5.582842	7.094263	6.234610	37.098762	176.566141	65.306351	631	2871	1485
gene2770	NC_008261.1	3076919	3078292	+	protein_coding	CPF_RS13770	CPF_2816	.	WP_003463013.	UDP-N-acetylm	2.592234	7.669671	2.477673	7.705245	1.046237	7.045818	7.013289	8.387485	7.078499	7.013289	8.387485	7.078499	105.789970	384.555226	123.777971	3075	10686	4810
gene2771	NC_008261.1	3078504	3079523	+	protein_coding	CPF_RS13775	CPF_2817	.	WP_003450734.	LacI family trans	1.138349	8.091720	-1.540523	8.491722	1.753653	8.394196	7.998788	8.185731	8.809151	7.998788	8.185731	8.809151	223.559554	342.194709	436.356628	4824	7059	12588
gene2772	NC_008261.1	3079639	3080457	+	protein_coding	CPF_RS13780	CPF_2818	.	WP_003456857.	purine-nucleosi	-2.639490	8.935611	-4.036606	9.214625	1.529313	9.964858	9.663127	8.262868	10.276011	9.663127	8.262868	10.276011	724.518927	355.660309	1319.246769	12553	5891	30558
gene2773	NC_008261.1	3080537	3081763	+	protein_coding	CPF_RS13785	CPF_2819	.	WP_003456809.	NupC/NupG fam	-2.646388	9.161648	-4.375421	9.491674	1.653356	10.246798	9.890517	8.486493	10.615914	9.890517	8.486493	10.615914	822.082889	428.611724	1610.061415	21339	10636	55873
gene2774	NC_008261.1	3081839	3082915	-	protein_coding	CPF_RS13790	CPF_2820	.	WP_003456773.	aminopeptidase	-1.437908	9.242973	-1.299502	9.171732	-1.106507	9.435382	9.508671	8.984700	9.362658	9.508671	8.984700	9.362658	648.876967	598.171423	660.931168	14784	13029	20132
gene2775	NC_008261.1	3083083	3083781	+	protein_coding	CPF_RS13795	CPF_2821	.	WP_003456816.	metallophospho	1.101488	6.016051	1.247580	5.924467	-1.132632	5.856200	5.946729	6.086182	5.767050	5.946729	6.086182	5.767050	46.931929	97.618514	46.384959	694	1380	917
gene2776	NC_008261.1	3083918	3085132	-	protein_coding	CPF_RS13800	CPF_2822	.	WP_011591148.	hypothetical pro	-1.099379	0.639010	-1.115021	0.648104	1.014228	0.721117	0.710999	0.574310	0.731380	0.710999	0.574310	0.731380	0.466865	0.732532	0.552920	12	18	19
gene2777	NC_008261.1	3085544	3086941	+	protein_coding	CPF_RS13805	CPF_2823	.	WP_003450738.	asparagine--tRN	3.694905	8.669247	4.246371	8.556665	-1.149251	7.676587	7.777590	9.663127	7.576896	7.777590	9.663127	7.576896	181.066627	899.363612	182.960082	5355	25428	7234
gene2778	NC_008261.1	3087255	3088736	-	protein_coding	CPF_RS13810	CPF_2824	.	WP_003456695.	leucyl aminopep	1.597684	8.566807	1.674455	8.531517	-1.048051	8.201556	8.235480	8.911462	8.167771	8.235480	8.911462	8.167771	258.677562	577.536112	281.406909	8110	17310	11795
gene2779	NC_008261.1	3088885	3089436	-	protein_coding	CPF_RS13815	CPF_2825	.	WP_003456686.	hypothetical pro	3.116428	4.871946	5.312862	4.393480	-1.704792	3.715847	4.120516	5.760409	3.350920	4.120516	5.760409	3.350920	12.502587	78.737220	8.070798	146	879	126
gene2780	NC_008261.1	3089566	3090075	-	protein_coding	CPF_RS13820	CPF_2826	.	WP_003450666.	anaerobic ribonu	2.585200	4.782983	3.535829	4.514922	-1.367720	3.914268	4.146667	5.516943	3.694894	4.146667	5.516943	3.694894	12.698034	65.733960	10.260690	137	678	148
gene2781	NC_008261.1	3090184	3090792	+	protein_coding	CPF_RS13825	CPF_2827	.	WP_003456742.	HD domain-con	-1.049568	0.345702	-1.309676	0.468344	1.247824	0.518002	0.382356	0.312561	0.701771	0.382356	0.312561	0.701771	0.232857	0.324768	0.522529	3	4	9
gene2782	NC_008261.1	3090850	3092241	-	protein_coding	CPF_RS13830	CPF_2828	.	WP_011591149.	MATE family effl	1.119817	0.358176	1.006320	0.444424	1.112784	0.354532	0.285729	0.448992	0.439903	0.285729	0.448992	0.439903	0.169792	0.532822	0.304808	5	15	12
gene2783	NC_008261.1	3092624	3093622	-	protein_coding	CPF_RS13835	CPF_2829	.	WP_011591150.	adenosine deam	-4.572334	5.526006	-4.042032	5.452503	-1.131197	6.640681	6.730201	4.537270	6.552351	6.730201	4.537270	6.552351	84.508827	32.369988	83.032389	1786	654	2346
gene2784	NC_008261.1	3094013	3096130	-	protein_coding	CPF_RS13840	CPF_2830	.	WP_003482449.	anaerobic ribonu	-1.119666	7.289417	1.358188	6.984007	-1.520716	7.062562	7.371408	7.208339	6.766657	7.371408	7.208339	6.766657	133.664004	192.997757	97.876618	5989	8267	5863
gene2785	NC_008261.1	3096390	3097457	+	protein_coding	CPF_RS13845	CPF_2831	.	WP_011591151.	DUF3048 domai	-1.044895	0.338172	-1.234324	0.434034	1.181290	0.476594	0.371332	0.307973	0.611694	0.371332	0.307973	0.611694	0.221302	0.324084	0.430385	5	7	13
gene2786	NC_008261.1	3097717	3098562	+	protein_coding	CPF_RS13850	CPF_2832	.	WP_011591152.	DegV domain-co	-2.468288	8.502481	-1.919959	8.332933	-1.285595	8.996137	9.179180	7.875669	8.816744	9.179180	7.875669	8.816744	497.453073	283.758678	439.548219	8903	4855	10517
gene2787	NC_008261.1	3098565	3099005	+	protein_coding	CPF_RS13855	CPF_2833	.	WP_003450702.	PadR family tran	-3.453048	7.377842	-2.470563	7.160626	-1.397677	8.080613	8.325736	6.537866	7.842706	8.325736	6.537866	7.842706	272.687120	129.949654	225.135265	2544	1159	2808
gene2788	NC_008261.1	3099057	3099245	-	protein_coding	CPF_RS13860	CPF_2834	.	WP_003450562.	ferredoxin	4.837889	1.063707	1.379129	2.451504	3.507930	0.967843	0.419947	2.694325	2.230567	0.419947	2.694325	2.230567	0.250106	7.848555	3.367408	1	30	18
gene2789	NC_008261.1	3099389	3100243	-	protein_coding	CPF_RS13865	CPF_2835	.	WP_003450706.	protein mrp	1.769528	7.883227	2.036358	7.775753	-1.150792	7.380279	7.482287	8.305652	7.279661	7.482287	8.305652	7.279661	143.468764	362.834588	142.589044	2595	6274	3448
gene2790	NC_008261.1	3100264	3101103	-	protein_coding	CPF_RS13870	CPF_2836	.	WP_003456826.	hypothetical pro	1.602974	5.618457	1.842163	5.510840	-1.149215	5.187088	5.288382	5.969134	5.087733	5.288382	5.969134	5.087733	29.374963	91.121729	29.085985	522	1548	691
gene2791	NC_008261.1	3101093	3101863	-	protein_coding	CPF_RS13875	CPF_2837	.	WP_003456764.	prolipoprotein di	1.382274	5.315662	1.382274	5.315662	1.000000	5.087267	5.087267	5.554310	5.087267	5.087267	5.554310	5.087267	25.811535	67.210509	29.075011	421	1048	634
gene2792	NC_008261.1	3101966	3102538	-	protein_coding	CPF_RS13880	CPF_2838	.	WP_041702733.	hypothetical pro	-1.726319	1.305607	-1.491541	1.224773	-1.157406	1.648752	1.757568	0.969869	1.546673	1.757568	0.969869	1.546673	1.814906	1.380688	1.666073	22	16	27
gene2793	NC_008261.1	3102687	3104309	-	protein_coding	CPF_RS13885	CPF_2839	.	WP_011591154.	hypothetical pro	-1.366519	2.562777	-1.083038	2.404258	-1.261747	2.624847	2.797910	2.347405	2.462488	2.797910	2.347405	2.462488	4.485267	5.879888	4.073879	154	193	187
gene2794	NC_008261.1	3104409	3105266	-	protein_coding	CPF_RS13890	CPF_2840	.	WP_003450598.	ribosomal RNA s	2.483575	5.205293	3.063340	5.030745	-1.233440	4.436359	4.590283	5.902702	4.287596	4.590283	5.902702	4.287596	18.180790	87.077802	15.989300	330	1511	388
gene2795	NC_008261.1	3105278	3105826	-	protein_coding	CPF_RS13895	CPF_2841	.	WP_011591155.	ribonuclease M5	2.704628	5.268421	3.109321	5.151916	-1.149630	4.497659	4.599368	6.034798	4.398199	4.599368	6.034798	4.398199	18.253646	94.568660	17.324669	212	1050	269
gene2796	NC_008261.1	3105967	3106989	-	protein_coding	CPF_RS13900	CPF_2842	.	WP_003456850.	DUF348 domain	-1.263716	5.393464	-1.110060	5.302054	-1.138421	5.470626	5.564942	5.227270	5.377908	5.564942	5.227270	5.377908	36.624379	53.312636	35.392345	793	1103	1024
gene2797	NC_008261.1	3107233	3108003	-	protein_coding	CPF_RS13905	CPF_2843	.	WP_011591156.	TatD family deox	1.103317	6.098124	1.050544	6.133789	1.050233	6.062865	6.027613	6.169460	6.098323	6.027613	6.169460	6.098323	50.151628	101.392953	59.204795	818	1581	1291
gene2798	NC_008261.1	3108022	3109521	-	protein_coding	CPF_RS13910	CPF_2844	.	WP_003462961.	DUF445 domain	-1.235249	4.311397	-1.098483	4.228902	-1.124505	4.381028	4.466490	4.161689	4.297201	4.466490	4.161689	4.297201	16.576033	25.777796	16.005289	526	782	679
gene2799	NC_008261.1	3109623	3110303	+	protein_coding	CPF_RS13915	CPF_2845	.	WP_003456830.	Crp/Fnr family t	-1.301459	3.590721	-1.081686	3.461860	-1.203176	3.649951	3.785813	3.405683	3.518965	3.785813	3.405683	3.518965	9.787192	14.376341	9.034147	141	198	174
gene2800	NC_008261.1	3110567	3112111	-	protein_coding	CPF_RS13920	CPF_2846	.	WP_003456732.	methionine--tR	1.278065	3.172476	1.114679	3.275140	1.146577	3.097524	3.000428	3.354390	3.197762	3.000428	3.354390	3.197762	5.323618	13.729638	7.208868	174	429	315
gene2801	NC_008261.1	3112212	3114149	-	protein_coding	CPF_RS13925	CPF_2847	.	WP_011591157.	methionine--tR	2.264287	7.361863	2.674514	7.230580	-1.181173	6.674711	6.795901	7.974958	6.555682	6.795901	7.974958	6.555682	88.759407	297.568382	83.304248	3639	11663	4566
gene2802	NC_008261.1	3114638	3115219	+	protein_coding	CPF_RS13930	CPF_2848	.	WP_003482466.	membrane prote	1.532479	3.943110	1.937623	3.755717	-1.264372	3.473853	3.647183	4.263049	3.308761	3.647183	4.263049	3.308761	8.690543	27.781459	7.776282	107	327	128
gene2803	NC_008261.1	3115254	3115763	+	protein_coding	CPF_RS13935	CPF_2849	.	WP_003456858.	membrane prote	2.521765	4.242044	2.510283	4.245892	1.004574	3.630269	3.626979	4.961413	3.633562	3.626979	4.961413	3.633562	8.527147	44.016546	9.844716	92	454	142
gene2804	NC_008261.1	3115893	3117608	-	protein_coding	CPF_RS13940	CPF_2850	.	WP_003456822.	sodium:proton a	1.635913	2.533085	1.593736	2.554660	1.026464	2.221560	2.202799	2.912895	2.240481	2.202799	2.912895	2.240481	2.644479	9.595271	3.420392	96	333	166
gene2805	NC_008261.1	3118034	3119647	-	protein_coding	CPF_RS13945	CPF_2852	.	WP_003463008.	AarF/ABC1/Ub	-1.399440	6.859993	-1.229918	6.769486	-1.137832	7.012938	7.106700	6.621851	6.920413	7.106700	6.621851	6.920413	114.074885	136.971881	110.410918	3895	4471	5040
gene2806	NC_008261.1	3119787	3120125	-	protein_coding	CPF_RS13950	CPF_2853	.	WP_003456770.	hypothetical pro	-3.729513	9.894124	-2.570561	9.647117	-1.450856	10.617227	10.889072	8.990085	10.352168	10.889072	8.990085	10.352168	1570.369794	603.123286	1386.880318	11262	4135	13297
gene2807	NC_008261.1	3120233	3121477	-	protein_coding	CPF_RS13955	CPF_2854	.	WP_003456838.	Xaa-Pro aminop	-2.704341	8.924311	-2.214614	8.790318	-1.221134	9.525556	9.670757	8.235480	9.382536	9.670757	8.235480	9.382536	726.971682	352.277211	664.215105	19147	8870	23388
gene2808	NC_008261.1	3121625	3124222	-	protein_coding	CPF_RS13960	CPF_2855	.	WP_011591158.	bifunctional ace	5.290834	12.046126	5.346512	12.037781	-1.010523	10.896621	10.904175	13.307670	10.889072	10.904175	13.307670	10.889072	1589.605708	11306.038986	1857.222620	87366	594045	136464
gene2809	NC_008261.1	3124651	3125169	-	protein_coding	CPF_RS13965	CPF_2856	.	WP_003479331.	spore maturatio	-1.030159	0.566392	-1.122552	0.623201	1.089688	0.647230	0.588231	0.545364	0.712146	0.588231	0.545364	0.712146	0.364316	0.666900	0.545014	4	7	8
gene2810	NC_008261.1	3125181	3125759	-	protein_coding	CPF_RS13970	CPF_2857	.	WP_011591159.	spore maturatio	-1.440782	1.334588	1.078798	1.040763	-1.554312	1.266273	1.623764	1.096912	0.987488	1.623764	1.096912	0.987488	1.551176	1.622577	0.854938	19	19	14
gene2811	NC_008261.1	3125874	3126053	+	protein_coding	CPF_RS13975	CPF_2858	.	WP_003450573.	hypothetical pro	-2.969159	4.534639	1.001946	3.815658	-2.974936	4.532972	5.387115	3.817061	3.814257	5.387115	3.817061	3.814257	32.038593	19.778360	11.196631	122	72	57
gene2812	NC_008261.1	3126093	3127460	-	protein_coding	CPF_RS13980	CPF_2859	.	WP_003450630.	MATE family effl	2.003497	3.419637	2.179900	3.348452	-1.088048	2.893408	2.954919	3.957440	2.833177	2.954919	3.957440	2.833177	5.114012	22.228968	5.479422	148	615	212
gene2813	NC_008261.1	3127649	3128281	-	protein_coding	CPF_RS13985	CPF_2860	.	WP_003463058.	hypothetical pro	1.019418	1.211266	1.392929	0.956743	-1.366395	0.945848	1.197472	1.225218	0.747097	1.197472	1.225218	0.747097	0.970791	1.952840	0.558575	13	25	10
gene2814	NC_008261.1	3128394	3129086	-	protein_coding	CPF_RS13990	CPF_2861	.	WP_003456899.	cytochrome c b	1.026854	2.398014	-1.424772	2.660343	1.463033	2.639221	2.378975	2.417206	2.927937	2.378975	2.417206	2.927937	3.137695	6.207494	5.816432	46	87	114
gene2815	NC_008261.1	3129090	3129518	-	protein_coding	CPF_RS13995	CPF_2862	.	WP_003456683.	thioredoxin fami	1.115530	2.597792	-1.016724	2.689791	1.134186	2.609373	2.520124	2.677853	2.701782	2.520124	2.677853	2.701782	3.636158	7.722320	5.027563	33	67	61
gene2816	NC_008261.1	3129623	3130621	-	protein_coding	CPF_RS14000	CPF_2863	.	WP_003450596.	LacI family trans	-1.195957	9.096676	-1.195957	9.096676	1.000000	9.226674	9.226674	8.968509	9.226674	9.226674	8.968509	9.226674	515.853994	595.083131	603.595210	10902	12023	17054
gene2817	NC_008261.1	3130953	3131282	+	protein_coding	CPF_RS14005	CPF_2864	.	WP_003462958.	rhodanese-like	1.428362	5.176017	1.919271	4.947001	-1.343687	4.707302	4.925222	5.439583	4.499025	4.925222	5.439583	4.499025	23.062057	61.732456	18.536049	161	412	173
gene2818	NC_008261.1	3131328	3134447	-	protein_coding	CPF_RS14010	CPF_2865	.	WP_011591160.	isoleucine--tRN	2.917174	7.649535	2.877655	7.717586	1.089451	6.977662	6.916135	8.460706	7.039736	6.916135	8.460706	7.039736	97.615694	416.407374	120.533772	6443	26275	10636
gene2819	NC_008261.1	3135114	3135329	+	protein_coding	CPF_RS14015	CPF_2866	.	WP_003456903.	(2Fe-2S)-bindin	2.237811	13.382029	3.265317	13.094276	-1.459157	12.538063	12.813593	13.975681	12.268458	12.813593	13.975681	12.268458	6971.457749	14501.612401	4670.173711	31856	63349	28530
gene2820	NC_008261.1	3135494	3136102	-	protein_coding	CPF_RS14020	CPF_2867	.	WP_003450594.	DUF1643 domai	2.407930	5.637083	2.020539	5.776901	1.191727	5.163692	5.038716	6.306509	5.291769	5.038716	6.306509	5.291769	25.148601	109.527945	33.964372	324	1349	585
gene2821	NC_008261.1	3136266	3138194	-	protein_coding	CPF_RS14025	CPF_2868	.	WP_011591161.	ABC transporte	1.701432	5.448307	1.841782	5.386617	-1.082490	5.020902	5.078404	5.845153	4.964051	5.078404	5.845153	4.964051	25.730201	83.870909	26.394612	1050	3272	1440
gene2822	NC_008261.1	3138325	3138930	-	protein_coding	CPF_RS14030	CPF_2869	.	WP_003472112.	energy-coupled	4.939219	7.483911	5.358879	7.415021	-1.084965	6.360839	6.419935	8.724219	6.302287	6.419935	8.724219	6.302287	68.408974	504.821418	68.790140	877	6187	1179
gene2823	NC_008261.1	3138930	3139595	-	protein_coding	CPF_RS14035	CPF_2870	.	WP_003463023.	thiaminase II	4.920005	7.057915	5.205860	7.009842	-1.058100	5.960675	6.001553	8.300213	5.920076	6.001553	8.300213	5.920076	49.328361	361.118400	52.399596	695	4864	987
gene2824	NC_008261.1	3140020	3140334	-	protein_coding	CPF_RS14040	CPF_2871	.	WP_003456890.	hypothetical pro	1.014763	0.509300	1.260178	0.311288	-1.241845	0.304894	0.498838	0.519981	0.186353	0.498838	0.519981	0.186353	0.300127	0.627884	0.112247	2	4	1
gene2825	NC_008261.1	3140496	3141671	+	protein_coding	CPF_RS14045	CPF_2872	.	WP_011591162.	class I SAM-dep	2.780730	6.335428	4.153292	6.001582	-1.493598	5.343277	5.640505	7.115968	5.061713	5.640505	7.115968	5.061713	38.185844	179.451614	28.532768	950	4268	949
gene2826	NC_008261.1	3142044	3142391	-	protein_coding	CPF_RS14050	CPF_2873	.	WP_003450634.	DUF1667 domai	-29.319556	6.424816	-28.782452	6.415604	-1.018661	9.294992	9.308339	4.434547	9.281665	9.308339	4.434547	9.281665	550.940642	30.548472	622.012476	4056	215	6122
gene2827	NC_008261.1	3142394	3143647	-	protein_coding	CPF_RS14055	CPF_2874	.	WP_003450714.	FAD-dependent	-25.922259	6.019329	-26.409488	6.028500	1.018796	8.822573	8.809151	4.113032	8.836016	8.809151	4.113032	8.836016	383.136236	24.801811	442.789959	10164	629	15704
gene2828	NC_008261.1	3143660	3145090	-	protein_coding	CPF_RS14060	CPF_2875	.	WP_011591163.	FAD/NAD(P)-bi	-11.086788	6.342072	-11.155449	6.345470	1.006193	8.315051	8.310599	4.839829	8.319506	8.310599	4.839829	8.319506	270.572342	40.496572	323.309978	8191	1172	13085
gene2829	NC_008261.1	3145256	3146758	-	protein_coding	CPF_RS14065	CPF_2876	.	WP_003456800.	glycerol kinase	-21.112880	7.805752	-18.542129	7.734517	-1.138644	10.215801	10.309889	5.909837	10.122572	10.309889	5.909837	10.122572	1074.536727	87.509043	1189.696443	34166	2660	50572
gene2830	NC_008261.1	3146778	3147338	-	protein_coding	CPF_RS14070	CPF_2877	.	WP_003456768.	glycerol-3-phos	-10.500079	7.002534	-10.430053	6.998736	-1.006714	8.896366	8.901194	5.508866	8.891541	8.901194	5.508866	8.891541	405.797834	65.222755	461.163799	4816	740	7317
gene2831	NC_008261.1	3147380	3148087	-	protein_coding	CPF_RS14075	CPF_2878	.	WP_003456873.	aquaporin family	-7.558542	7.885066	-7.939254	7.914501	1.050369	9.513357	9.477976	6.559868	9.548871	9.477976	6.559868	9.548871	634.073041	132.204926	745.609726	9497	1893	14930
gene2832	NC_008261.1	3148353	3148796	-	protein_coding	CPF_RS14080	CPF_2880	.	WP_003472067.	hypothetical pro	-6.171304	8.417620	-5.234697	8.315347	-1.178923	9.712702	9.832162	7.206587	9.594693	9.832162	7.206587	9.594693	807.636571	192.549459	780.180610	7586	1729	9797
gene2833	NC_008261.1	3149057	3149800	-	protein_coding	CPF_RS14085	CPF_2881	.	WP_003456794.	2-phosphosulfol	-1.012648	4.703186	-1.074576	4.745728	1.061154	4.754885	4.712261	4.694128	4.797895	4.712261	4.694128	4.797895	19.822926	36.153991	23.191663	312	544	488
gene2834	NC_008261.1	3149827	3150762	-	protein_coding	CPF_RS14090	CPF_2882	.	WP_003450751.	TIGR01212 fami	1.022397	4.833153	1.052520	4.812092	-1.029463	4.796210	4.817201	4.849157	4.775309	4.817201	4.849157	4.775309	21.463433	40.887955	22.589694	425	774	598
gene2835	NC_008261.1	3150855	3151940	-	protein_coding	CPF_RS14095	CPF_2883	.	WP_011591164.	hypothetical pro	1.088385	0.930957	1.034904	0.968990	1.051677	0.907484	0.871865	0.994055	0.944557	0.871865	0.994055	0.944557	0.609375	1.411439	0.813945	14	31	25
gene2836	NC_008261.1	3152100	3153158	+	protein_coding	CPF_RS14100	CPF_2884	.	WP_003456735.	transporter	-1.111288	1.060877	-1.644745	1.297696	1.480035	1.394140	1.139721	0.987488	1.705352	1.139721	0.987488	1.705352	0.892730	1.400734	1.969886	20	30	59
gene2837	NC_008261.1	3153204	3154520	-	protein_coding	CPF_RS14105	CPF_2885	.	WP_011591165.	spore cortex-ly	1.149388	2.815076	1.140081	2.821147	1.008164	2.722293	2.716434	2.917300	2.728164	2.716434	2.917300	2.728164	4.163498	9.611352	5.154665	116	256	192
gene2838	NC_008261.1	3154643	3156391	-	protein_coding	CPF_RS14110	CPF_2886	.	WP_011591166.	peptidase S8	-1.133535	1.890056	-1.089760	1.862776	-1.040170	1.954016	1.982632	1.801803	1.925813	1.982632	1.801803	1.925813	2.216206	3.618682	2.506784	82	128	124
gene2839	NC_008261.1	3156394	3158091	-	protein_coding	CPF_RS14115	CPF_2887	.	WP_011591167.	peptidase S8	-1.162168	1.292497	-1.162168	1.292497	1.000000	1.405445	1.405445	1.188626	1.405445	1.405445	1.188626	1.405445	1.252740	1.863685	1.436800	45	64	69
gene2840	NC_008261.1	3158107	3159843	-	protein_coding	CPF_RS14120	CPF_2888	.	WP_011591168.	peptidase S8	-1.096811	1.144956	-1.195655	1.202248	1.090120	1.274276	1.213552	1.080237	1.338039	1.213552	1.080237	1.338039	0.979690	1.594111	1.343474	36	56	66
gene2841	NC_008261.1	3159984	3160655	-	protein_coding	CPF_RS14125	CPF_2889	.	WP_011591169.	helix-turn-helix	1.204037	8.204631	1.154565	8.235337	1.042850	8.101993	8.071784	8.339664	8.132315	8.071784	8.339664	8.132315	234.240005	375.553378	272.076028	3330	5104	5171
gene2842	NC_008261.1	3160657	3161031	-	protein_coding	CPF_RS14130	CPF_2890	.	WP_003456740.	DUF3783 domai	-1.312958	6.679881	-1.098469	6.553754	-1.195262	6.749288	6.879178	6.486357	6.621851	6.879178	6.486357	6.621851	94.414057	123.680676	87.593012	749	938	929
gene2843	NC_008261.1	3161060	3161572	-	protein_coding	CPF_RS14135	CPF_2891	.	WP_003456782.	peptidylprolyl iso	1.065433	9.768436	1.235925	9.660259	-1.160022	9.615151	9.722824	9.814263	9.508671	9.722824	9.814263	9.508671	748.396466	976.773380	724.937917	8122	10134	10518
gene2844	NC_008261.1	3161813	3162082	-	protein_coding	CPF_RS14140	CPF_2892	.	WP_003450538.	DUF4325 domai	-1.228438	5.283917	-1.523170	5.432653	1.239924	5.587386	5.434413	5.137588	5.744665	5.434413	5.137588	5.744665	33.264113	49.812165	45.703207	190	272	349
gene2845	NC_008261.1	3162318	3163100	-	protein_coding	CPF_RS14145	CPF_2893	.	WP_003456846.	glutamate racem	-1.984692	6.710876	-1.942196	6.696356	-1.021880	7.207895	7.223525	6.234610	7.192299	7.223525	6.234610	7.192299	123.638663	105.143579	133.031962	2048	1665	2946
gene2846	NC_008261.1	3163406	3165307	+	protein_coding	CPF_RS14150	CPF_2894	.	WP_003456676.	glutamine synth	43.879265	6.642737	47.872719	6.548342	-1.091010	4.389965	4.453247	9.908715	4.327583	4.453247	9.908715	4.327583	16.477417	1050.582379	16.470555	663	40412	886
gene2847	NC_008261.1	3165397	3165681	-	protein_coding	CPF_RS14155	CPF_2895	.	WP_003456707.	hypothetical pro	-1.968620	3.865514	-2.298585	3.962830	1.167613	4.495254	4.384862	3.407678	4.608424	4.384862	3.407678	4.608424	15.259105	14.400034	20.098108	92	83	162
gene2848	NC_008261.1	3165921	3166562	-	protein_coding	CPF_RS14160	CPF_2896	.	WP_003450676.	VanZ family prot	-1.210196	4.423076	-1.428595	4.537605	1.180465	4.680985	4.562837	4.287596	4.802193	4.562837	4.287596	4.802193	17.891937	28.265802	23.296483	243	367	423
gene2849	NC_008261.1	3167173	3170376	-	protein_coding	CPF_RS14165	CPF_2897	.	WP_003456883.	carbamoyl-phos	100.000832	6.397665	146.301727	5.928700	-1.463005	3.601857	3.886767	10.530635	3.337832	3.886767	10.530635	3.337832	10.474950	1656.962335	8.011782	710	107368	726
gene2850	NC_008261.1	3170389	3171438	-	protein_coding	CPF_RS14170	CPF_2898	.	WP_003450627.	carbamoyl-phos	113.343959	6.115163	164.855661	5.636127	-1.454472	3.309231	3.590495	10.415059	3.050000	3.590495	10.415059	3.050000	8.328534	1528.820117	6.532771	185	32465	194
gene2851	NC_008261.1	3172096	3172752	+	protein_coding	CPF_RS14175	CPF_2899	.	WP_003462951.	hypothetical pro	1.211251	0.472425	1.066505	0.582192	1.135720	0.436237	0.353989	0.630487	0.537596	0.353989	0.630487	0.537596	0.215845	0.827861	0.376719	3	11	7
gene2852	NC_008261.1	3173007	3174479	-	protein_coding	CPF_RS14180	CPF_2900	.	WP_011591170.	iron hydrogenas	-1.151908	2.915548	1.027825	2.795452	-1.183960	2.894973	3.019345	2.815319	2.775724	3.019345	2.815319	2.775724	5.391289	8.727722	5.280864	168	260	220
gene2853	NC_008261.1	3174548	3175165	-	protein_coding	CPF_RS14185	CPF_2901	.	WP_011591171.	DNA-3-methyla	-1.012707	1.974714	1.155132	1.858688	-1.169811	1.867281	1.983843	1.965626	1.757568	1.983843	1.965626	1.757568	2.218174	4.160496	2.059677	29	52	36
gene2854	NC_008261.1	3175478	3175789	-	protein_coding	CPF_RS14190	CPF_2902	.	WP_003462970.	PTS lactose/ce	1.269969	5.629825	1.127761	5.717472	1.126097	5.545071	5.460067	5.804860	5.631399	5.460067	5.804860	5.631399	33.785969	81.458950	42.044029	223	514	371
gene2855	NC_008261.1	3175890	3177137	-	protein_coding	CPF_RS14195	CPF_2903	.	WP_011591172.	gamma-glutamyl	1.052746	5.318154	-1.157873	5.460077	1.218946	5.422141	5.281205	5.355362	5.566839	5.281205	5.355362	5.566839	29.165018	58.558525	39.805836	770	1478	1405
gene2856	NC_008261.1	3177140	3177949	-	protein_coding	CPF_RS14200	CPF_2904	.	WP_003450599.	glutamate 5-kin	1.344203	4.184128	1.090780	4.339808	1.232332	4.124133	3.976189	4.402941	4.277580	3.976189	4.402941	4.277580	11.263112	30.155894	15.889176	193	494	364
gene2857	NC_008261.1	3178036	3178914	-	protein_coding	CPF_RS14205	CPF_2905	.	WP_011591173.	NAD(P)-depend	1.517298	2.558664	1.732218	2.448860	-1.141646	2.177874	2.275527	2.877031	2.084411	2.275527	2.877031	2.084411	2.850185	9.225401	2.976651	53	164	74
gene2858	NC_008261.1	3178978	3179751	-	protein_coding	CPF_RS14210	CPF_2906	.	WP_011591174.	NAD(P)-depend	1.644551	1.522697	1.533921	1.584877	1.072122	1.254792	1.205563	1.923256	1.306032	1.205563	1.923256	1.306032	0.977159	4.024666	1.279093	16	63	28
gene2859	NC_008261.1	3179937	3181229	+	protein_coding	CPF_RS14215	CPF_2907	.	WP_003450694.	NCS2 family per	-2.577295	3.514381	-6.517350	4.028468	2.528756	4.886653	4.263049	2.897192	5.601477	4.263049	2.897192	5.601477	13.745971	9.407340	40.854235	376	246	1494
gene2860	NC_008261.1	3181320	3181547	+	protein_coding	CPF_RS14220	CPF_2908	.	WP_003450671.	hypothetical pro	1.141954	0.426515	1.223570	0.358948	-1.071470	0.287299	0.341378	0.532883	0.241787	0.341378	0.532883	0.241787	0.207325	0.650604	0.155078	1	3	1
gene2861	NC_008261.1	3181613	3183262	-	protein_coding	CPF_RS14225	CPF_2909	.	WP_041702734.	hydroxylamine re	-1.904109	3.305398	-1.067194	2.919861	-1.784221	3.358930	3.802443	2.873326	2.967148	3.802443	2.873326	2.967148	9.912387	9.199934	6.042966	346	307	282
gene2862	NC_008261.1	3183567	3184916	+	protein_coding	CPF_RS14230	CPF_2910	.	WP_011591176.	membrane prote	2.230940	7.514785	1.302096	7.923165	1.713346	7.336352	6.958219	8.115870	7.735035	6.958219	8.115870	7.735035	100.772754	327.807999	208.087095	2878	8950	7945
gene2863	NC_008261.1	3184958	3185746	-	protein_coding	CPF_RS14235	CPF_2911	.	WP_003479364.	DUF3888 domai	-1.365761	0.938127	-1.215168	0.869127	-1.123928	1.102057	1.189549	0.739844	1.021000	1.189549	0.739844	1.021000	0.958582	1.002705	0.896268	16	16	20
gene2864	NC_008261.1	3185879	3186466	+	protein_coding	CPF_RS14240	CPF_2912	.	WP_011591177.	sugar O-acetylt	1.181408	5.893699	1.288365	5.829544	-1.090533	5.711812	5.774671	6.015179	5.649638	5.774671	6.015179	5.649638	41.723059	93.257657	42.633788	519	1109	709
gene2865	NC_008261.1	3186518	3186991	+	protein_coding	CPF_RS14245	CPF_2913	.	WP_003479367.	peptide-methior	-1.982999	5.550319	-1.312392	5.270857	-1.510980	5.760656	6.066088	5.078404	5.470603	6.066088	5.078404	5.470603	51.458541	47.255258	37.819400	516	453	507
gene2866	NC_008261.1	3186993	3188477	-	protein_coding	CPF_RS14250	CPF_2914	.	WP_011591178.	DNA methyltran	-1.215750	2.966644	-1.262339	2.992401	1.038322	3.137922	3.110912	2.829066	3.165166	3.110912	2.829066	3.165166	5.825199	8.890273	7.047746	183	267	296
gene2867	NC_008261.1	3188606	3190762	-	protein_coding	CPF_RS14255	CPF_2915	.	WP_003456789.	YhgE/Pip domai	1.535448	4.360107	1.194372	4.550466	1.285569	4.239069	4.061736	4.680396	4.424144	4.061736	4.680396	4.424144	11.943523	35.966906	17.621519	545	1569	1075
gene2868	NC_008261.1	3190785	3192950	-	protein_coding	CPF_RS14260	CPF_2916	.	WP_011591179.	YhgE/Pip domai	1.486562	3.909105	1.184326	4.081685	1.255197	3.793978	3.633562	4.205542	3.961476	3.633562	4.205542	3.961476	8.598523	26.503550	12.373591	394	1161	758
gene2869	NC_008261.1	3193302	3193640	-	protein_coding	CPF_RS14265	CPF_2917	.	WP_003456718.	hypothetical pro	-1.149491	8.675006	1.066702	8.528384	-1.226165	8.627756	8.776086	8.575090	8.481933	8.776086	8.575090	8.481933	371.746215	460.765770	355.246624	2666	3159	3406
gene2870	NC_008261.1	3193854	3194660	-	protein_coding	CPF_RS14270	CPF_2918	.	WP_003456862.	hypothetical pro	-1.106555	6.956984	-1.093504	6.948510	-1.011935	7.021842	7.030405	6.884330	7.013289	7.030405	6.884330	7.013289	106.840868	157.957284	118.165970	1824	2578	2697
gene2871	NC_008261.1	3194797	3195828	-	protein_coding	CPF_RS14275	CPF_2919	.	WP_003456679.	DNA methylase	1.630586	3.525546	1.567272	3.556974	1.040398	3.218890	3.190449	3.895839	3.247584	3.190449	3.895839	3.247584	6.183583	21.033672	7.468989	135	439	218
gene2872	NC_008261.1	3195952	3197385	-	protein_coding	CPF_RS14280	CPF_2920	.	WP_003482523.	potassium/proto	-1.419974	4.073685	-1.438722	4.082568	1.013203	4.343913	4.334462	3.828598	4.353385	4.334462	3.828598	4.353385	14.569989	19.895595	16.618651	442	577	674
gene2873	NC_008261.1	3197638	3198006	-	protein_coding	CPF_RS14285	CPF_2921	.	WP_003456828.	phage holin fami	-2.294058	1.805656	-1.714188	1.646953	-1.338276	2.281504	2.501354	1.303452	2.080978	2.501354	1.303452	2.080978	3.586888	2.143996	2.970437	28	16	31
gene2874	NC_008261.1	3198543	3198941	+	protein_coding	CPF_RS14290	CPF_2922	.	WP_003456879.	DUF2871 domai	1.171351	0.291968	-1.042734	0.456742	1.221407	0.311901	0.199380	0.427553	0.487924	0.199380	0.427553	0.487924	0.118471	0.495698	0.354464	1	4	4
gene2875	NC_008261.1	3199025	3201265	-	protein_coding	CPF_RS14295	CPF_2923	.	WP_011591180.	cell wall anchor	-6.067040	5.971360	-4.144418	5.745609	-1.463906	7.131624	7.411833	4.810840	6.862009	7.411833	4.810840	6.862009	137.359482	39.605261	104.937353	6512	1795	6651
gene2876	NC_008261.1	3201879	3202769	+	protein_coding	CPF_RS14300	CPF_2925	.	WP_011591181.	NAD(P)-depend	-1.579336	1.720012	-1.315937	1.607552	-1.200160	1.944916	2.080978	1.421660	1.817751	2.080978	1.421660	1.817751	2.387377	2.441773	2.222262	45	44	56
gene2877	NC_008261.1	3202927	3203079	-	protein_coding	CPF_RS14305	CPF_2926	.	WP_003479385.	cytochrome c55	5.439483	13.678550	3.340114	14.057897	1.628532	12.858243	12.511268	14.954737	13.214840	12.511268	14.954737	13.214840	5747.791531	22179.232859	8337.503662	18604	68629	36078
gene2878	NC_008261.1	3203450	3203929	-	protein_coding	CPF_RS14310	CPF_2927	.	WP_003456814.	23S rRNA (pseu	2.010397	2.696058	2.793880	2.393187	-1.389715	1.987451	2.238974	3.246455	1.764185	2.238974	3.246455	1.764185	2.757420	12.567499	2.062537	28	122	28
gene2879	NC_008261.1	3204175	3204546	-	protein_coding	CPF_RS14315	CPF_2928	.	WP_011591182.	TIGR03987 fami	-1.326209	4.443405	-1.027844	4.264180	-1.290282	4.464096	4.651724	4.244416	4.284037	4.651724	4.244416	4.284037	19.187576	27.381331	15.968031	151	206	168
gene2880	NC_008261.1	3204776	3205417	-	protein_coding	CPF_RS14320	CPF_2929	.	WP_003456852.	lactate utilizatio	-1.011563	4.902221	-1.122407	4.976535	1.109577	4.984962	4.910521	4.893934	5.060531	4.910521	4.893934	5.060531	22.825104	42.283176	28.528553	310	549	518
gene2881	NC_008261.1	3205524	3206024	-	protein_coding	CPF_RS14325	CPF_2930	.	WP_003456869.	hypothetical pro	1.776021	9.422908	1.718962	9.447490	1.033194	9.041213	9.017688	9.846337	9.064799	9.017688	9.846337	9.064799	437.413124	982.305462	531.284198	4636	9953	7528
gene2882	NC_008261.1	3206168	3206722	-	protein_coding	CPF_RS14330	CPF_2931	.	WP_003456832.	hypothetical pro	2.021212	7.530257	1.638930	7.690326	1.233251	7.189378	7.039736	8.054956	7.342202	7.039736	8.054956	7.342202	108.082342	314.137371	150.796162	1269	3526	2367
gene2883	NC_008261.1	3206738	3207529	-	protein_coding	CPF_RS14335	CPF_2932	.	WP_011591183.	MBL fold metallo	2.168144	7.677731	1.743105	7.845166	1.243840	7.295470	7.139768	8.256228	7.454569	7.139768	8.256228	7.454569	116.503974	354.986596	168.663763	1952	5686	3778
gene2884	NC_008261.1	3207552	3208805	-	protein_coding	CPF_RS14340	CPF_2933	.	WP_003456772.	UDP-N-acetylgl	1.870823	7.826940	1.761468	7.872834	1.062082	7.431455	7.388135	8.291808	7.475030	7.388135	8.291808	7.475030	135.439639	360.158568	172.080178	3593	9134	6103
gene2885	NC_008261.1	3209145	3209690	+	protein_coding	CPF_RS14345	CPF_2934	.	WP_011591184.	hypothetical pro	-1.830763	5.924328	-1.702174	5.875318	-1.075544	6.323838	6.376589	5.504144	6.271523	6.376589	5.504144	6.271523	66.403173	64.931703	66.894851	767	717	1033
gene2886	NC_008261.1	3209885	3210364	+	protein_coding	CPF_RS14350	CPF_2935	.	WP_003456877.	dUTPase	1.040004	4.935676	1.161292	4.854987	-1.116623	4.827235	4.907462	4.964051	4.748320	4.907462	4.964051	4.748320	22.748714	44.089260	22.245938	231	428	302
gene2887	NC_008261.1	3210420	3210851	+	protein_coding	CPF_RS14355	CPF_2936	.	WP_003475850.	EamA-like trans	1.886332	1.388279	2.237813	1.205871	-1.186330	0.872100	1.004019	1.919603	0.757513	1.004019	1.919603	0.757513	0.765950	4.006034	0.572927	7	35	7
gene2888	NC_008261.1	3210918	3212114	+	protein_coding	CPF_RS14360	CPF_2937	.	WP_004456928.	peptidase	1.282343	0.791942	1.264508	0.804490	1.014104	0.642639	0.632615	0.991397	0.652821	0.632615	0.991397	0.652821	0.394904	1.404478	0.472619	10	34	16
gene2889	NC_008261.1	3212117	3213169	-	protein_coding	CPF_RS14365	CPF_2938	.	WP_011591185.	M23 family pepti	-1.151084	0.630227	1.011762	0.528349	-1.164623	0.620246	0.739844	0.536851	0.519981	0.739844	0.536851	0.519981	0.493799	0.657400	0.369360	11	14	11
gene2890	NC_008261.1	3213399	3214400	+	protein_coding	CPF_RS14370	CPF_2939	.	WP_003456897.	DnaD domain pro	1.745663	6.140657	1.467164	6.273075	1.189821	5.875941	5.751906	6.555682	6.002651	5.751906	6.555682	6.002651	41.231565	131.658343	54.977469	874	2668	1558
gene2891	NC_008261.1	3214397	3215392	+	protein_coding	CPF_RS14375	CPF_2940	.	WP_003456704.	DNA replication	1.784009	3.607906	1.527429	3.731509	1.167982	3.324550	3.214428	4.049550	3.438445	3.214428	4.049550	3.438445	6.312166	23.978283	8.555447	133	483	241
gene2901	NC_008261.1	3216239	3216313	-	tRNA	CPF_RS14425	CPF_2950	.	.	tRNA-Glu	4.050757	2.620380	2.102974	3.236730	1.926204	2.221988	1.798869	3.817061	2.744630	1.798869	3.817061	2.744630	1.890802	19.778360	5.185808	3	30	11
gene2902	NC_008261.1	3216512	3217081	-	protein_coding	CPF_RS14430	CPF_2951	.	WP_011591186.	hypothetical pro	1.706674	2.586248	1.335695	2.783814	1.277743	2.399535	2.229241	3.000428	2.582838	2.229241	3.000428	2.582838	2.736687	10.236169	4.466246	33	118	72
gene2903	NC_008261.1	3217277	3218524	+	protein_coding	CPF_RS14435	CPF_2952	.	WP_003456905.	NAD(P)/FAD-de	1.765828	4.181734	1.759580	4.184553	1.003551	3.794186	3.791629	4.611974	3.796744	3.791629	4.611974	3.796744	9.847928	34.509117	11.105970	260	871	392
gene2904	NC_008261.1	3218641	3219399	-	protein_coding	CPF_RS14440	CPF_2953	.	WP_003456854.	acyl-ACP thioes	-1.535919	6.076342	-1.376696	6.000847	-1.115656	6.314334	6.393773	5.774671	6.235881	6.393773	5.774671	6.235881	67.074900	79.673696	65.358324	1077	1223	1403
gene2905	NC_008261.1	3219422	3220162	-	protein_coding	CPF_RS14445	CPF_2954	.	WP_011591187.	acyl-ACP thioes	1.195855	4.889148	1.253310	4.854268	-1.048045	4.727857	4.761829	5.019871	4.694128	4.761829	5.019871	4.694128	20.477311	45.108540	21.615486	321	676	453
gene2906	NC_008261.1	3220295	3220837	-	protein_coding	CPF_RS14450	CPF_2955	.	WP_011591188.	NADH peroxidas	1.187564	14.197607	1.618499	13.970506	-1.362873	13.849019	14.074145	14.322151	13.627495	14.074145	14.322151	13.627495	18952.158150	16756.423067	12024.836558	217707	184014	184669
gene2907	NC_008261.1	3221152	3221343	-	protein_coding	CPF_RS14455	CPF_2956	.	WP_003451010.	DUF1858 domai	1.954490	11.256802	4.188648	10.667408	-2.143090	10.219153	10.783781	11.750573	9.684088	10.783781	11.750573	9.684088	1487.529545	4053.276094	827.224776	6042	15739	4492
gene2908	NC_008261.1	3221453	3221932	-	protein_coding	CPF_RS14460	CPF_2957	.	WP_003472888.	NUDIX domain-c	1.553284	3.847922	3.295524	3.204975	-2.121649	2.951295	3.543351	4.178673	2.458165	3.543351	4.178673	2.458165	7.976822	26.062109	4.051413	81	253	55
gene2909	NC_008261.1	3222060	3223346	-	protein_coding	CPF_RS14465	CPF_2958	.	WP_003450991.	adenylosuccinat	16.639327	7.382264	18.905072	7.260457	-1.136168	5.534709	5.627563	9.684088	5.443387	5.627563	9.684088	5.443387	37.940922	913.731021	37.006163	1033	23783	1347
gene2910	NC_008261.1	3223827	3224507	+	protein_coding	CPF_RS14470	CPF_2959	.	WP_003459996.	L-serine ammor	-3.082256	7.790306	-2.427879	7.633595	-1.269526	8.470609	8.644503	7.020516	8.300213	8.644503	7.020516	8.300213	335.263386	169.466563	318.583481	4830	2334	6136
gene2911	NC_008261.1	3224508	3225389	+	protein_coding	CPF_RS14475	CPF_2960	.	WP_003451046.	L-serine ammor	-2.681363	8.825705	-2.312229	8.726573	-1.159644	9.458375	9.565820	8.142853	9.352138	9.565820	8.142853	9.352138	682.521713	331.825712	654.559938	12735	5919	16328
gene2912	NC_008261.1	3225625	3226797	+	protein_coding	CPF_RS14480	CPF_2961	.	WP_011591189.	SIS domain-cor	-3.980599	4.622840	-13.941802	5.302868	3.502438	6.567748	5.725515	3.732529	7.533874	5.725515	3.732529	7.533874	40.459621	18.505328	178.416634	1004	439	5919
gene2913	NC_008261.1	3226817	3227674	+	protein_coding	CPF_RS14485	CPF_2962	.	WP_011010998.	tagatose bisphos	-6.036823	5.318743	-18.347442	5.915137	3.039255	7.530758	6.771469	4.177680	8.375187	6.771469	4.177680	8.375187	87.433074	26.048423	335.239577	1587	452	8135
gene2914	NC_008261.1	3227731	3228456	-	protein_coding	CPF_RS14490	CPF_2963	.	WP_003466360.	GntR family tran	2.040350	5.136358	3.105563	4.789783	-1.522074	4.334045	4.647645	5.676461	4.041606	4.647645	5.676461	4.041606	19.142419	73.896420	13.003482	294	1085	267
gene2915	NC_008261.1	3228593	3228856	-	protein_coding	CPF_RS14495	CPF_2964	.	WP_011010999.	preprotein trans	-4.496858	1.265707	-2.554312	1.061542	-1.760497	2.307428	2.751211	0.582294	1.935229	2.751211	0.582294	1.935229	4.297278	0.749180	2.544689	24	4	19
gene2916	NC_008261.1	3228873	3229277	-	protein_coding	CPF_RS14500	CPF_2965	.	WP_003451031.	PTS sugar trans	-4.060376	3.540105	-3.664379	3.483812	-1.108066	4.617777	4.692392	2.670779	4.544348	4.692392	2.670779	4.544348	19.608319	7.691584	19.294000	168	63	221
gene2917	NC_008261.1	3229450	3230256	-	protein_coding	CPF_RS14505	CPF_2966	.	WP_003460011.	mannose perme	-4.316651	4.119791	-3.214469	3.951274	-1.342881	5.090568	5.307674	3.197762	4.882343	5.307674	3.197762	4.882343	29.873269	12.070436	24.623387	510	197	562
gene2918	NC_008261.1	3230256	3231047	-	protein_coding	CPF_RS14510	CPF_2967	.	WP_003451051.	PTS sugar trans	-4.744347	4.499248	-3.213239	4.274063	-1.476500	5.472104	5.760409	3.514200	5.198228	5.760409	3.514200	5.198228	41.361298	15.857650	31.473783	693	254	705
gene2919	NC_008261.1	3231094	3231582	-	protein_coding	CPF_RS14515	CPF_2968	.	WP_003451042.	PTS sugar trans	-3.073577	4.771421	-2.155270	4.550059	-1.426075	5.387533	5.649638	4.029719	5.137588	5.649638	4.029719	5.137588	38.376711	23.560112	30.151728	397	233	417
gene2920	NC_008261.1	3231860	3232660	-	protein_coding	CPF_RS14520	CPF_2969	.	WP_003459984.	pyrroline-5-carl	1.181750	5.558741	1.401792	5.431411	-1.186200	5.314983	5.439583	5.680509	5.193237	5.439583	5.680509	5.193237	33.283784	74.076254	31.340855	564	1200	710
gene2921	NC_008261.1	3232838	3234172	-	protein_coding	CPF_RS14525	CPF_2970	.	WP_003451036.	replicative DNA	1.766769	6.115687	1.845752	6.081857	-1.044704	5.687261	5.718896	6.540009	5.655801	5.718896	6.540009	5.655801	40.152991	130.189016	42.826617	1134	3515	1617
gene2922	NC_008261.1	3234588	3236486	-	protein_coding	CPF_RS14530	CPF_2971	.	WP_003459992.	ATP-dependent	-1.114671	8.722460	-1.069184	8.692624	-1.042543	8.771015	8.801120	8.644503	8.741013	8.801120	8.644503	8.741013	381.520871	482.091009	416.808483	15327	18515	22386
gene2923	NC_008261.1	3236502	3236948	-	protein_coding	CPF_RS14535	CPF_2972	.	WP_003451030.	50S ribosomal p	-1.178493	7.314566	-1.247251	7.354703	1.058344	7.474789	7.433997	7.197054	7.515805	7.433997	7.197054	7.515805	139.483675	190.482861	175.602407	1319	1722	2220
gene2924	NC_008261.1	3236950	3238908	-	protein_coding	CPF_RS14540	CPF_2973	.	WP_011591190.	delta-lactam-bio	-1.243485	6.924453	-1.182376	6.888827	-1.051684	7.046987	7.083432	6.769042	7.010731	7.083432	6.769042	7.010731	111.913484	147.025198	117.949518	4638	5825	6535
gene2925	NC_008261.1	3238941	3239936	-	protein_coding	CPF_RS14545	CPF_2974	.	WP_011591191.	DUF2232 domai	1.159688	4.312729	-1.049632	4.455726	1.217245	4.346682	4.207185	4.420922	4.490805	4.207185	4.420922	4.490805	13.241311	30.481709	18.495386	279	614	521
gene2926	NC_008261.1	3239954	3240271	-	protein_coding	CPF_RS14550	CPF_2975	.	WP_003459975.	hypothetical pro	1.150656	5.368420	1.343298	5.253401	-1.167419	5.155276	5.268147	5.470603	5.044824	5.268147	5.470603	5.044824	28.986355	63.284532	28.130562	195	407	253
gene2927	NC_008261.1	3240554	3240796	-	protein_coding	CPF_RS14555	CPF_2976	.	WP_003451020.	30S ribosomal p	2.574444	9.759356	2.080683	9.922701	1.237307	9.253362	9.101035	10.465296	9.408239	9.101035	10.465296	9.408239	469.393592	1577.181753	683.438281	2413	7751	4697
gene2928	NC_008261.1	3240827	3241279	-	protein_coding	CPF_RS14560	CPF_2977	.	WP_003451037.	single-stranded	3.096049	11.863644	2.259144	12.104678	1.370453	11.301446	11.076405	12.706834	11.531058	11.076405	12.706834	11.531058	1763.809582	7206.003055	2852.740785	16903	66018	36549
gene2929	NC_008261.1	3241293	3241580	-	protein_coding	CPF_RS14565	CPF_2978	.	WP_003479405.	30S ribosomal p	3.345112	12.277294	2.286649	12.568411	1.462888	11.708321	11.437126	13.179181	11.985946	11.437126	13.179181	11.985946	2410.608676	10266.548229	3932.202781	14687	59798	32029
gene2930	NC_008261.1	3241724	3241936	-	protein_coding	CPF_RS14570	CPF_2979	.	WP_003459977.	DUF951 domain	-1.218288	4.142721	1.044789	3.971010	-1.272853	4.109880	4.287596	4.002741	3.939530	4.287596	4.002741	3.939530	13.981284	22.981897	12.283924	63	99	74
gene2931	NC_008261.1	3241973	3242833	-	protein_coding	CPF_RS14575	CPF_2980	.	WP_003466073.	mechanosensiti	-1.004884	4.680146	1.226599	4.526904	-1.232590	4.530305	4.683662	4.676632	4.381969	4.683662	4.676632	4.381969	19.544878	35.835356	17.083435	356	624	416
gene2932	NC_008261.1	3242866	3243099	-	protein_coding	CPF_RS14580	CPF_2981	.	WP_003460020.	DUF3343 domai	-1.550984	3.442179	1.833274	2.667310	-2.843378	2.923892	3.773300	3.140116	2.265694	3.773300	3.140116	2.265694	9.696422	11.410592	3.475338	48	54	23
gene2933	NC_008261.1	3243259	3243906	+	protein_coding	CPF_RS14585	CPF_2982	.	WP_003459974.	translocator pro	1.384527	5.402467	1.474445	5.354853	-1.064945	5.127275	5.172866	5.642259	5.082087	5.172866	5.642259	5.082087	27.355356	72.261214	28.973738	375	947	531
gene2934	NC_008261.1	3243918	3245078	+	protein_coding	CPF_RS14590	CPF_2983	.	WP_011591192.	aminotransferas	1.146876	6.388624	1.158912	6.380972	-1.010494	6.282999	6.290534	6.488244	6.275473	6.290534	6.488244	6.275473	62.171726	123.721221	67.182831	1527	2905	2206
gene2936	NC_008261.1	3246204	3246794	+	protein_coding	CPF_RS14600	CPF_2985	.	WP_011591194.	spore protease	-1.380433	2.168149	-1.178296	2.062984	-1.171551	2.296098	2.413146	1.948025	2.184727	2.413146	1.948025	2.184727	3.279310	4.099575	3.230660	41	49	54
gene2937	NC_008261.1	3246944	3247441	-	protein_coding	CPF_RS14605	CPF_2986	.	WP_003451034.	DUF4446 domai	1.456825	5.477430	1.381919	5.517296	1.054204	5.250676	5.212737	5.755564	5.288891	5.212737	5.755564	5.288891	27.906418	78.041118	33.795792	294	786	476
gene2938	NC_008261.1	3247490	3248350	-	protein_coding	CPF_RS14610	CPF_2987	.	WP_003451039.	chromosome pa	1.263590	6.239843	-1.056704	6.450552	1.335241	6.278448	6.073361	6.410889	6.490460	6.073361	6.410889	6.490460	51.771965	117.843189	80.119666	943	2052	1951
gene2939	NC_008261.1	3248351	3249124	-	protein_coding	CPF_RS14615	CPF_2988	.	WP_003451027.	ParA family prot	1.237173	5.407015	-1.186843	5.684940	1.468330	5.525816	5.255671	5.562718	5.809847	5.255671	5.562718	5.809847	28.642966	67.844374	48.103029	469	1062	1053
gene2940	NC_008261.1	3249374	3250093	-	protein_coding	CPF_RS14620	CPF_2989	.	WP_003459994.	ribosomal RNA s	3.594349	4.673086	2.403745	5.013808	1.495312	4.120490	3.840475	5.686206	4.420922	3.840475	5.686206	4.420922	10.110540	74.306199	17.580675	154	1082	358
gene2941	NC_008261.1	3250270	3252162	-	protein_coding	CPF_RS14625	CPF_2991	.	WP_003451018.	tRNA uridine-5-	2.675573	7.327550	2.462826	7.393094	1.086383	6.711437	6.651936	8.071784	6.771469	6.651936	8.071784	6.771469	79.707351	315.038031	98.583399	3192	12061	5278
gene2942	NC_008261.1	3252177	3253553	-	protein_coding	CPF_RS14630	CPF_2992	.	WP_003451045.	tRNA uridine-5-	1.567733	6.269820	1.868903	6.134886	-1.192106	5.825729	5.953864	6.602544	5.700352	5.953864	6.602544	5.700352	47.029757	135.231123	44.267840	1370	3766	1724
gene2943	NC_008261.1	3253895	3254518	-	protein_coding	CPF_RS14635	CPF_2993	.	WP_011591195.	protein jag	1.308478	8.730468	1.170573	8.812227	1.117810	8.618640	8.538677	8.926566	8.699352	8.538677	8.926566	8.699352	313.164115	579.246033	404.631285	4134	7310	7141
gene2944	NC_008261.1	3254560	3255276	-	protein_coding	CPF_RS14640	CPF_2994	.	WP_003460013.	protein transloc	1.227442	8.986479	1.244995	8.976060	-1.014301	8.829619	8.839868	9.135522	8.819382	8.839868	9.135522	8.819382	390.093327	652.244513	440.418900	5917	9458	8931
gene2945	NC_008261.1	3255310	3255519	-	protein_coding	CPF_RS14645	CPF_2995	.	WP_003451050.	membrane prote	1.298344	7.646288	1.176602	7.718738	1.103469	7.530959	7.460270	7.836943	7.602317	7.460270	7.836943	7.602317	141.359974	276.190666	186.891134	628	1173	1110
gene2946	NC_008261.1	3255491	3255868	-	protein_coding	CPF_RS14650	CPF_2996	.	WP_003450986.	ribonuclease P p	1.130947	8.285946	1.174734	8.258203	-1.038717	8.170208	8.197655	8.375187	8.142853	8.197655	8.375187	8.142853	250.981483	383.532743	275.940362	2007	2932	2950
gene2947	NC_008261.1	3255929	3256063	-	protein_coding	CPF_RS14655	CPF_2997	.	WP_003451019.	50S ribosomal p	1.362404	12.186227	1.362404	12.186227	1.000000	11.965191	11.965191	12.411346	11.965191	11.965191	12.411346	11.965191	3503.936599	6160.226529	3827.283473	10007	16819	14613

Supplementary Table 2 Primers

name	Base sequence (5'-3')	Fragment length
F-CPF_0394	5'-AAGGAGATATACATATGAAAGAATCTAGAAAAAAAC-3'	3039 bp
R-CPF_0394	5'-GGTGGTGGTGCTCGAGTTTTTTTCTTTTCTTAGTAAAC-3'	
